# Synthesis and Antiproliferative Activity of Fluorinated *N*‑Acetylmannosamine Analogs

**DOI:** 10.1021/acs.joc.5c03084

**Published:** 2026-03-19

**Authors:** Aleš Krčil, Lucia Šutvajová, Vojtěch Hamala, Ivana Císařová, Martin Kurfiřt, Lucie Červenková Št’astná, Jana Bernášková, Roman Hrstka, Jindřich Karban

**Affiliations:** † Institute of Chemical Process Fundamentals of the CAS, V. V. I., Rozvojová 1/135, 165 00 Praha, Czech Republic; ‡ Department of Organic Chemistry, University of Chemistry and Technology, Technická 5, 166 28 Praha, Czech Republic; § Department of Experimental Biology, Faculty of Science, Masaryk University, Kotlářská 2, 611 37 Brno, Czech Republic; ∥ Research Centre for Applied Molecular Oncology, Masaryk Memorial Cancer Institute, Žlutý Kopec 7, 656 53 Brno, Czech Republic; ⊥ Department of Inorganic Chemistry, Faculty of Science, 37740 Charles University, Hlavova 8, CZ-128 43 Praha 2, Czech Republic

## Abstract

Introducing fluorine into monosaccharides, or their incomplete acylation that leaves the anomeric group unprotected, can impart antitumor properties to the resulting glycomimetics. This property could be exploited in the development of new antiproliferative agents. Herein, we report the synthesis and antiproliferative activity of a complete series of deoxyfluorinated analogs of acylated *N*-acetylmannosamine (ManNAc) hemiacetals, which combine both aforementioned structural features. In addition, retentive deoxyfluorination at C4 of a 1,6-anhydro-β-d-mannopyranose derivative provided access to 3,4-difluoro and 3,4,6-trifluoro talosazides (TalN_3_). Attempted conversion of the fluorinated talosazides to fluorinated *N*-acetyltalosamine (TalNAc) analogs was prevented by unwanted reactions, partly arising from an elimination-addition mechanism. The *in vitro* antitumor activity of the fluoroanalogs against MDA-MB-231 breast cancer cells was investigated using an MTT proliferation assay, a colony forming assay, a wound healing assay, and cell cycle analysis. The trifluorinated ManNAc analog showed the most pronounced antiproliferative activity. In addition, Western blot analysis indicated the induction of programmed cell death in MDA-MB-231 cells.

## Graphical abstract

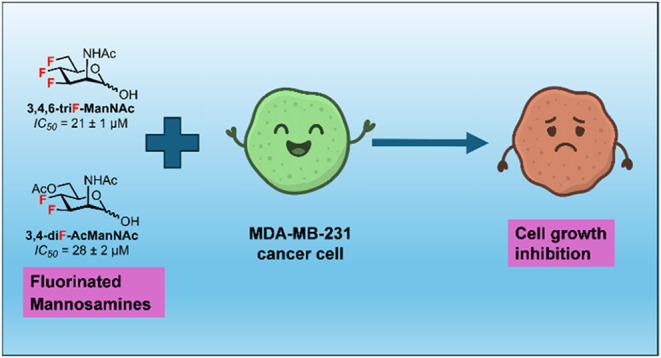

## Introduction

Fluorinated carbohydrates are valuable probes and inhibitors of carbohydrate-processing enzymes and carbohydrate-binding proteins.^1^ In addition, some fluorinated monosaccharides can cross the cytoplasmic membrane, enter metabolic pathways, and disrupt the biosynthesis of cell surface and extracellular glycans in a controlled and targeted manner.^2–9^ These fluorinated monosaccharide-based metabolic inhibitors of glycosylation are typically acetylated prior to administration to facilitate diffusion across the plasma membrane. The antiproliferative activity of some acetylated fluorinated monosaccharides^10,11^ limits their use as metabolic inhibitors at higher concentrations, however, this property can be exploited in the development of new antitumor therapeutics.^11,12^ For example, fully acetylated 3-fluoro and 4-fluoro analogs of *N*-acetyl-d-glucosamine (GlcNAc), and 4-fluoro and 4,6-difluoro analogs of *N*-acetyl-d-galactosamine (GalNAc) were cytotoxic against leukemia cells (*IC*_50_ 24–35 μM),^13,14^ while fully acetylated 6,6-difluoro-l-fucose **1** and 6,6,6-trifluoro-l-fucose **2** (Figure 1) reduced the viability of the human colon cancer cells HCT116 (*IC*_50_ 43 μM and 58 μM, respectively).^11^

**Figure 1:**
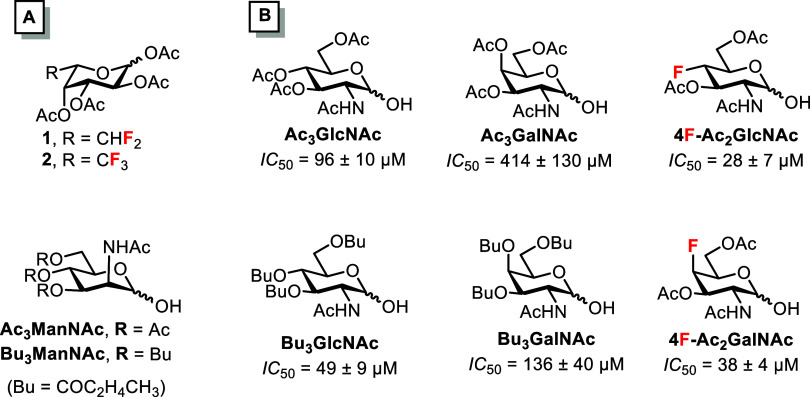
(A) Structures of fucose analogs **1** and **2**, and O-acylated ManNAc hemiacetals.^15^ (B) Structures and cytotoxicity against MDA-MB-231 cells of O-acylated GlcNAc and GalNAc hemiacetals and their 4-fluoro analogs.^19^

In addition to fluorinated monosaccharides, other types of monosaccharide glycomimetics may have antitumor activity. For example, 2-acetamido-3,4,6-tri-*O*-acetyl-d-mannopyranose (Ac_3_ManNAc) and 2-acetamido-3,4,6-tri-*O*-butyryl-d-mannopyranose (Bu_3_ManNAc, Figure 1A) have demonstrated promising cytotoxic and antimigratory activity against the cell line MDA-MB-231,^15–17^ a human cell line derived from highly aggressive triple-negative breast cancer with limited treatment options.^18^ These two compounds are structurally hemiacetals (also termed lactols) of O-acylated *N*-acetylmannosamine (ManNAc), characterized by an unprotected anomeric hydroxyl group and O-acyl groups at the 3-, 4-, and 6-positions. The GlcNAc hemiacetal Ac_3_GlcNAc (a C2-epimer of Ac_3_ManNAc) also showed cytotoxicity, albeit weak, but the GalNAc hemiacetal Ac_3_GalNAc (a C4-epimer of Ac_3_GlcNAc) was virtually noncytotoxic (Figure 1B).^19^ Consistent with the previously observed trend for ManNAc hemiacetals, the cytotoxicity against MDA-MB-231 cells increased after the O-acetyl groups were replaced with O-butyryl groups to give Bu_3_GlcNAc and Bu_3_GalNAc (Figure 1, Bu = butyryl).^19^

Introducing fluorine at the 4-position of Ac_3_GlcNAc and Ac_3_GalNAc—thereby combining the cytotoxicity of hemiacetals with that of fluorosugars—produced fluorinated hemiacetals 4F-Ac_2_GlcNAc and 4F-Ac_2_GalNAc exhibiting approximately 3-fold and 10-fold increased cytotoxicity to MDA-MB-231 cells, respectively (Figure 1B).^19^ Hemiacetal 4F-Ac_2_GlcNAc also inhibited growth of human prostate cancer cells PC-3 *in vitro*.^12^ Introducing an additional fluorine substituent at the 3- and 6-positions, or replacing the 2-acetamido group with more lipophilic azido or phenyl-triazole groups, further enhanced the antiproliferative effect against MDA-MB-231 cells.^19,20^ In contrast to nonfluorinated hexosamine hemiacetals, however, the substitution of butyryl esters for acetyl esters at the nonfluorinated positions of these fluorinated hemiacetals mostly decreased cytotoxicity.^19^

The confirmed positive role of the fluorine substituent in enhancing the cytotoxicity of acylated GlcNAc and GalNAc hemiacetals,^19^ suggests that replacing an acetoxy group with fluorine could also significantly increase the cytotoxicity of acylated ManNAc hemiacetals. This motivated us to synthesize a complete series of acetylated mono-, di-, and trifluorinated ManNAc hemiacetal analogs **3**, **6**–**11** (Figure 2) and to determine their *in vitro* antitumor properties. The 3-fluoro ManNAc analog was prepared with two different O-acyl groups—acetyl (compound **3**) and propionyl (compound **4**)—to evaluate the effect of alkyl chain length on cytotoxicity. The 2-azido-4-fluoro analog **5** was included for comparison. During the synthesis of 3,4-difluorinated and 3,4,6-trifluorinated ManNAc derivatives, we unexpectedly obtained precursors of fluorinated *N*-acetyltalosamine with an amino group masked as an azide. Evaluation of antitumor activity comprised the determination of the cytotoxic activity by an MTT assay, a cell proliferation assay, and a colony-forming assay. The antimigratory activity of selected compounds was evaluated using a wound healing assay. To elucidate the mechanisms underlying the cytotoxicity of the most cytotoxic fluoro analogs, we also conducted cell cycle arrest analysis and Western blotting to detect proteins characteristic for cell apoptosis and autophagy. The trifluorinated mannosamine hemiacetal **11** displayed the strongest antiproliferative and cytotoxic activity in all assays, placing it among the most cytotoxic fluorinated monosaccharides reported.

**Figure 2:**
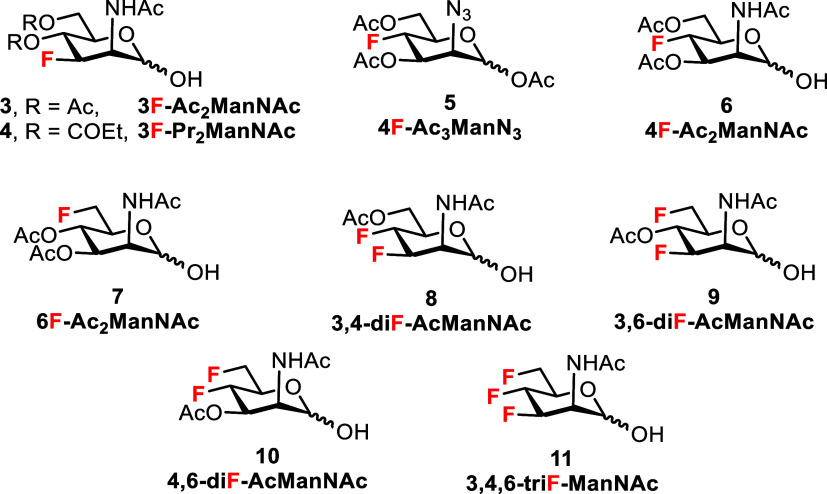
Structures of the target fluorinated ManNAc analogs.

## Results

### Synthesis

Initially, we planned to prepare the 3-fluoro ManNAc analog from the known d-*altro*-configured 2-azido intermediate **12** ^21^ by analogy with a recently described fluorination of its d-*allo*-configured counterpart **13**, which upon reaction with diethylaminosulfur trifluoride (DAST) yielded the desired 3-fluoro product **14** with an inversion of the configuration at C3 in a usable yield of 41%, together with 5% of the elimination products **15** and **16**.^22^ However, fluorination of **12** predominantly yielded the d-*altro-*configured product **17** with retention of the configuration at C3, containing traces of the desired inseparable product **18** (Scheme 1). The axial orientation of the fluorine substituent in **17** was evidenced by a large vicinal coupling between *trans*-diaxially disposed fluorine and H-4 (^3^ *J*_F,H‑4_ = 29.6 Hz). The addition of triethylamine trihydrofluoride^23^ improved the yield but **17** remained the main reaction product. Conducting the reaction in toluene at higher temperatures led predominantly to the elimination product **19**,^24^ while the desired **18** was formed in a disappointing 18% yield. The microwave-assisted reaction in dichloromethane at 80 °C also gave the elimination product **19** along with the low yield (19%) of an inseparable mixture of both C3-configurational isomers **17** and **18**.

**Scheme 1:**
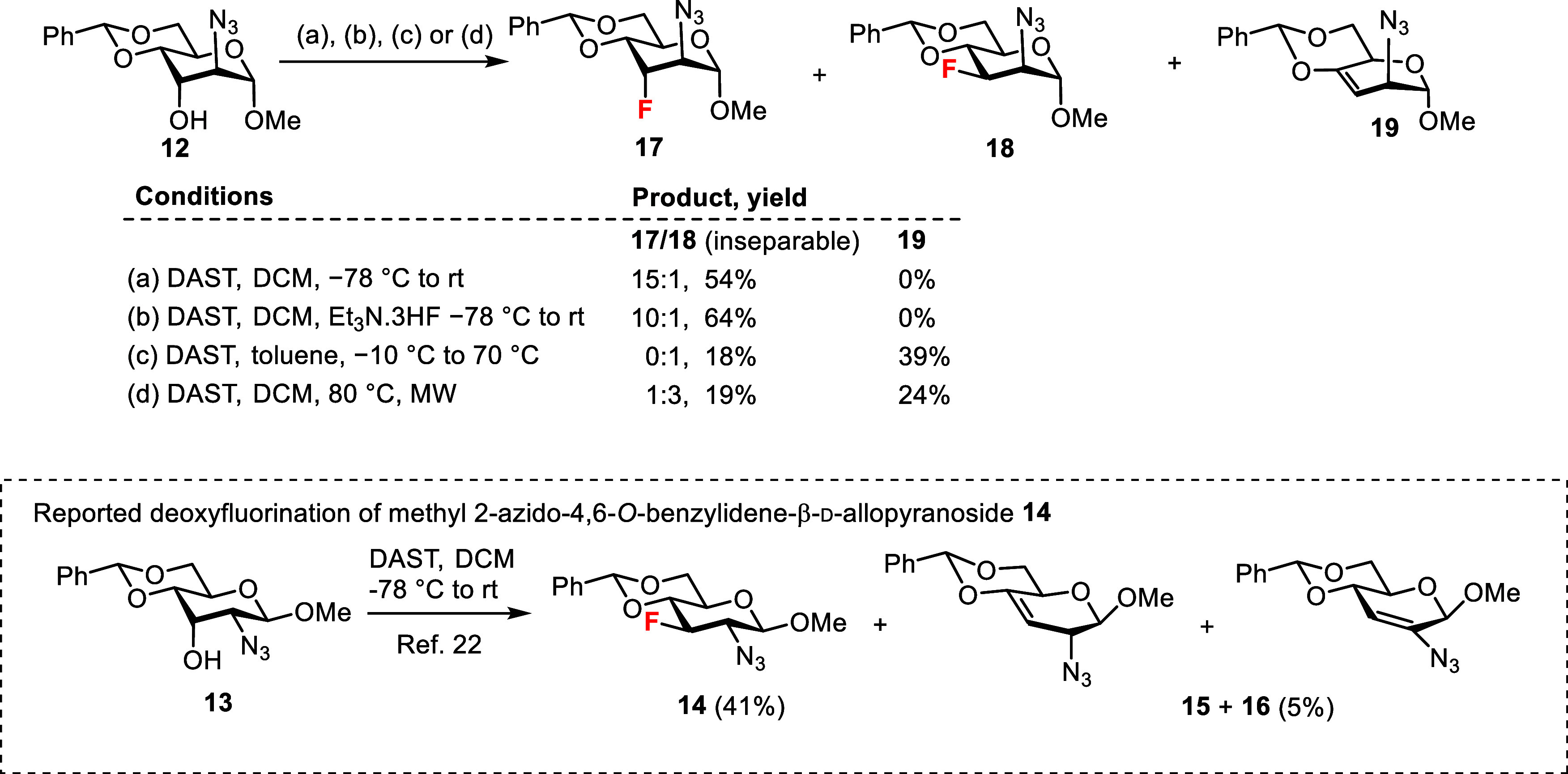
Fluorination of 2-Azido-altropyranoside **12** and Methyl 2-Azido-allopyranoside **13**.^22^

An alternative route to the required 3-fluoro analogs was developed starting from 3-fluorinated 1,6-anhydro-β-d-glucopyranose **20** (Scheme 2), available in six steps from levoglucosan.^25^ The C2 hydroxyl group was activated as trifluoromethanesulfonate and subsequent nucleophilic substitution by reaction with sodium azide afforded 1,6-anhydro-2-azido-3-fluoro-mannopyranose **21**. The equatorial position of the 2-azido group was indicated by the large vicinal coupling constant (^3^ *J*_H‑2,F_ = 28.2 Hz). Oxidative de-O-benzylation with the NaBrO_3_/Na_2_S_2_O_4_ system^26^ liberated the hydroxyl at the 4-position, giving alcohol **22**. Subsequent reaction with trimethylsilylthiophenol (TMSSPh) effected cleavage of the internal acetal^27,28^ and introduced a stable thiophenyl group at the anomeric position to give compound **23** as a separable pair of anomers. Protection of the anomeric position as a thioglycoside enabled us to manipulate the remaining positions.^28^ Then, we could regioselectively and chemoselectively introduce the unprotected anomeric hydroxyl to obtain the desired hemiacetals (vide infra). In addition, thioglycosides are highly useful as versatile glycosyl donors.

**Scheme 2:**
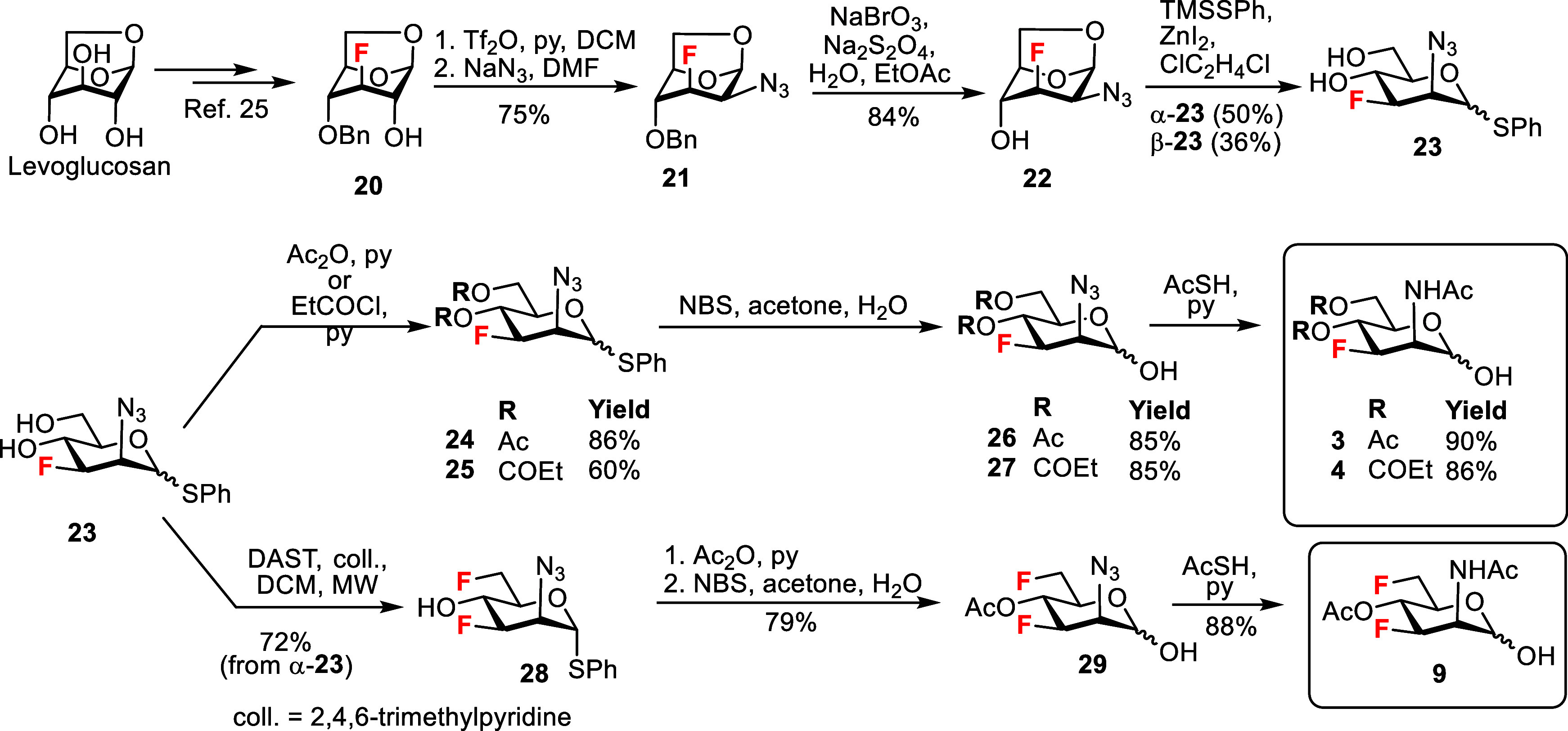
Synthesis of the Target 3-Fluoro and 3,6-Difluoro ManNAc Analogs

The two unprotected 4- and 6-hydroxyl groups in **23** were either acetylated with acetic anhydride or propionylated with propionyl chloride in pyridine to give the acylated products **24** and **25**. Hydrolysis of the thiophenyl glycoside (NBS/acetone/water) yielded the hemiacetals **26** and **27**, which were converted to the desired acylated 3-fluoro ManNAc hemiacetals **3** and **4** on reaction with thioacetic acid.^29^ The microwave-assisted deoxyfluorination of diol **23** by reaction with DAST (1.3 equiv) proceeded regioselectively at the primary 6-hydroxyl group,^22,30^ yielding 3,6-difluoro intermediate **28**. To prevent migration of the anomeric thiophenyl group to the 6- or 4-positions during the reaction with DAST, α-thioglycoside α-**23** was used as the starting material.^28,31^ Acetylation of the 4-OH group and subsequent hydrolysis of the thiophenyl group yielded the 2-azido-hemiacetal **29**, which was converted to the desired acetylated 3,6-difluoro ManNAc hemiacetal **9** by reaction with thioacetic acid (Scheme 2).

To prepare the 4-fluoro and 4,6-difluoro analogs, the 4-fluoro-mannosazide intermediate **30**, previously used in the chemoenzymatic synthesis of 7-fluoro-neuraminic acid, was obtained in six steps from methyl α-d-mannoside as reported^32^ (Scheme 3). Zemplén deacylation liberated the hydroxyl group at the 6-position to give the alcohol **31**. The sulfuric acid-catalyzed acetolysis with Ac_2_O acetolysed both the benzyl ether and the methyl glycoside group,^33^ producing compound **5**, which was included in the cytotoxicity assays for comparison. The azide was converted to an acetamide by reaction with AcSH to give the intermediate **32**. The following regioselective hydrolysis of the anomeric acetate by treatment with hydrazine acetate yielded the target 4-fluoro hemiacetal **6**.

**Scheme 3:**
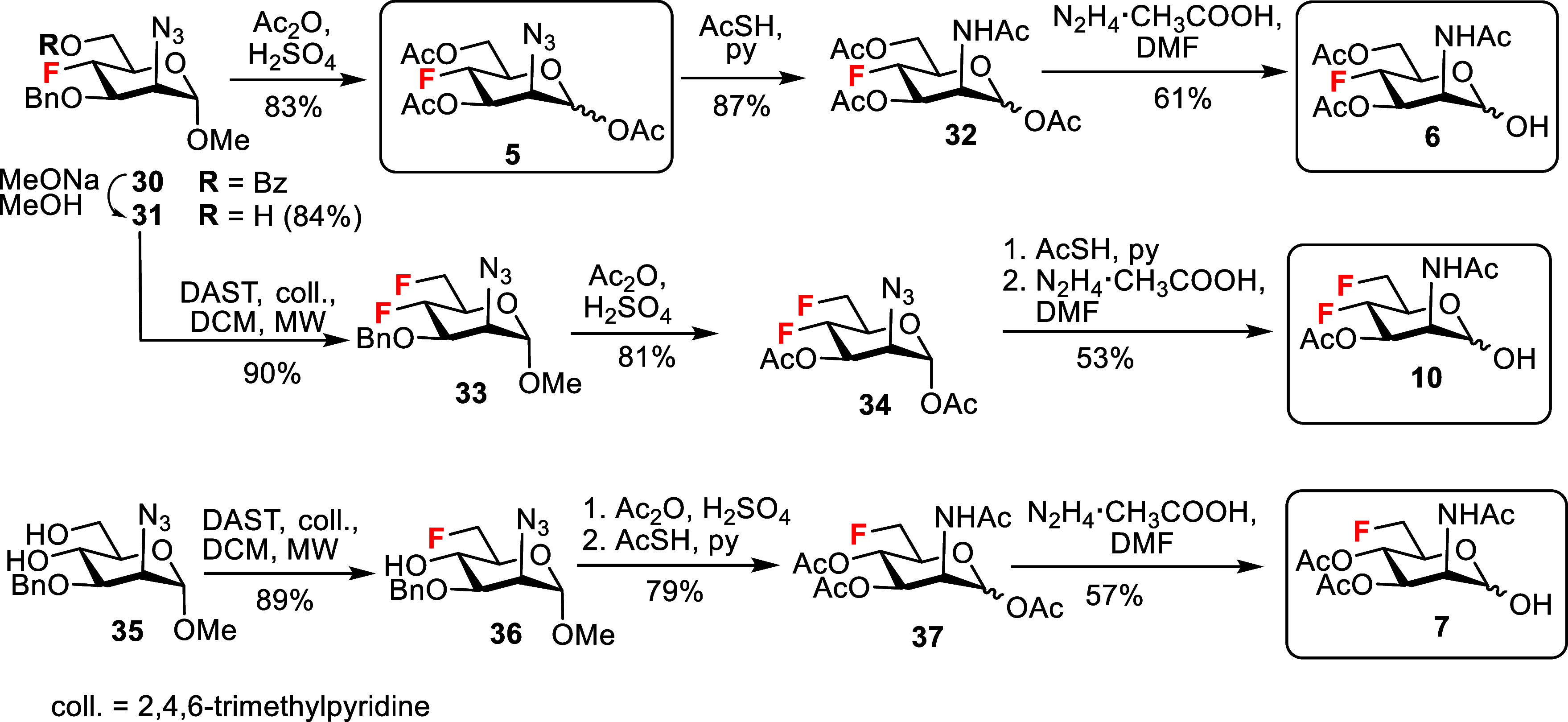
Synthesis of the Target 4-Fluoro, 4,6-Difluoro and 6-Fluoro ManNAc Analogs

The C6-deoxyfluorination of alcohol **31** using a microwave-assisted reaction with DAST afforded the 4,6-difluoro-mannosazide **33**. Sulfuric acid-catalyzed acetolysis yielded the intermediate **34**, which was isolated as an α-acetate, and the subsequent azide-to-acetamide conversion followed by anomeric deprotection resulted in the desired acetylated 4,6-difluoro ManNAc hemiacetal **10**. The target acetylated 6-fluoro ManNAc hemiacetal **7** was prepared from diol **35** (Scheme 3), which was synthesized from methyl α-d-mannopyranoside in five steps using the published route.^34^ Microwave-assisted deoxyfluorination using 1.2 equiv of DAST resulted in regioselective C6-deoxyfluorination to produce 6-fluoro-mannosazide **36**. The intermediate **36** was then subjected to acetolysis followed by an azide-to-acetamide conversion to give the acetate **37**. Anomeric deprotection afforded the target 6-fluoro ManNAc analog **7**.

We have originally intended to prepare the 3,4-difluoro ManNAc analogs from the 3-fluoro analog **22** using Latrell-Dax inversion^38^ at C4 followed by DAST-mediated fluorination as the key reactions. Accordingly, the C4 hydroxyl in **22** was converted to trifluoromethanesulfonate ester and then reacted with KNO_2_ to give the expected d-talosazide derivative **38** (Scheme 4A). However, the subsequent reaction with DAST proceeded with complete retention of the configuration at the 4-position, giving 3,4-difluorotalosazide **39** as the sole product in good yield of 85%. The equatorial position of the fluorine substituent at the 4-position is evidenced from the large coupling between H-4 and F-3 (^3^ *J*_H‑3,F‑4_ = 24.4 Hz). In addition, the magnitude of the geminal coupling constant ^2^ *J*_F‑4,C‑5_ = 27.3 Hz indicates the *anti*-periplanar relationship between bonds C4–F4 and C5–O5.^39^ Retentive DAST-mediated fluorination at C4 of a 1,6-anhydrotalopyranose scaffold has previously been reported for 1,6-anhydro-2,3-dideoxy-2,3-difluorotalose^35^ **40** and 1,6:2,3-dianhydrotalopyranose^36^ **42** to give the corresponding products **41** and **43**, respectively (Scheme 4B). On the other hand, DAST-mediated fluorination of galactosan **44** was less stereoselective and afforded, in addition to the major product **45** with inversion of the configuration at C4, an inseparable minor product **46** with retention of the configuration.^37^ The available data suggest that deoxyfluorination of the C4 equatorial hydroxyl in 1,6-anhydro-β-d-hexopyranoses is highly stereoselective for *talo*-configured substrates, favoring configurational retention. Retention of configuration at C4 in these reactions is most likely due to the participation of the endocyclic O5 oxygen of the pyranose ring and involves an epoxonium intermediate **47** (Scheme 4C).^36,37^

**Scheme 4:**
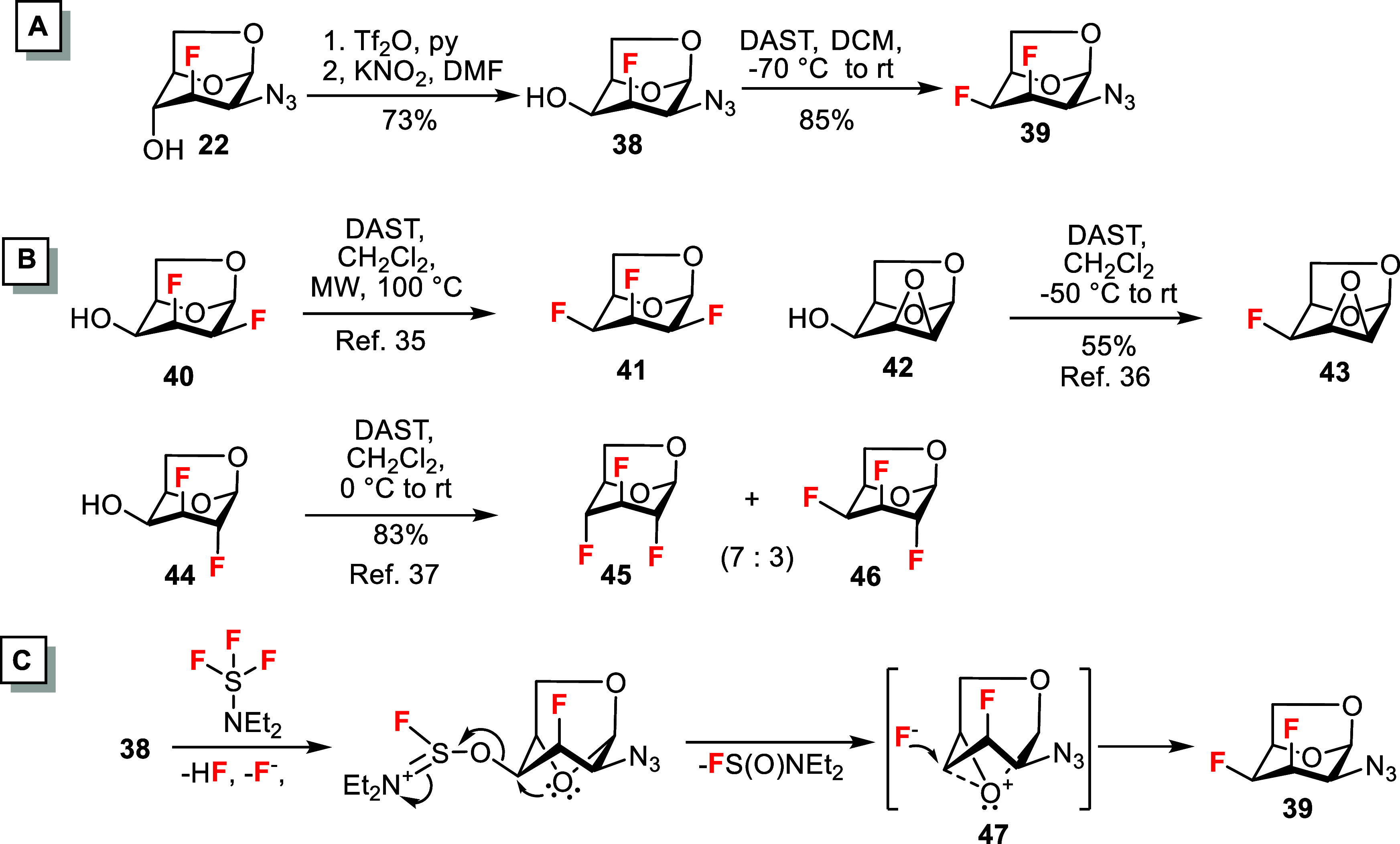
(A) Formation of d-*Talo*-product **39**. (B) Reported Reactions of 1,6-Anhydropyranoses **40**, **42** and **46** with DAST.^35–37^ (C) Suggested Mechanism for Retentive Fluorination of **38**

Having access to 3,4-difluoro-talosazide **39**, we attempted to prepare 3,4-difluoro and 3,4,6-trifluoro *N*-acetyltalosamine, however our efforts were thwarted by complications during azide to acetamide conversion. The reaction of **39** with TMSSPh afforded separable anomers of the thioglycoside **48** (Scheme 5). The β-anomer β-**48** was acetylated to yield the 6-acetate **49**, while DAST deoxyfluorination of the α-anomer α-**48** afforded the trifluoro compound **50**. Only the α-anomer was used in the reaction with DAST to prevent thioaglycone migration.^31^ Hydrolysis of the thiophenyl group in compounds **49** and **50** yielded the corresponding hemiacetals **51** and **52** in moderate yields of around 40%. Hemiacetal **52** was obtained in about 90% purity due to partial decomposition during chromatography. Hemiacetals **51** and **52** showed sluggish and irreproducible reaction under the conditions for azide-to-acetamide conversion that were successful for hemiacetals **26**, **27** and **29** (Scheme 2) and previously for fluorinated GlcNAc and GalNAc hemiacetals.^19^ The addition of an excess of AcSH to 2-azidotaloses **51** and **52** led to mixtures of compounds and further attempts at azide reduction were abandoned. However, a difluorinated analog was surprisingly isolated from the product mixture after the reaction of hemiacetal **52** with AcSH (Scheme 5). Its HRMS spectrum indicated the substitution of one fluorine with a thioacetyl group, and the NMR spectrum suggested the *galacto*-configuration (^3^ *J*_H‑2,H‑3_ = 12.4 Hz, ^3^ *J*_H‑3,F‑4_ = 35.3 Hz, ^3^ *J*_H‑3,H‑4_ = 2.2 Hz, ^3^ *J*_H‑4,H‑5_ < 1 Hz) and an unprotected α-configured anomeric hydroxyl. There was also no fluorine or oxygen substituent at C3 (the chemical shift of C-3 carbon δ_C‑3_ = 43.4 ppm). The product was assigned the structure of 3-thioester of 4,6-difluorinated GalNAc analog **53** and was obtained as a crystalline α-anomer (Scheme 5). The structure of the thioacetate **53** was eventually confirmed by an X-ray diffraction analysis. Interestingly, the compound **53** crystallizes with two independent molecules, one of which has the fluorinated exocyclic side chain C(5)–C(6)­H_2_F in the *gg* conformation (Figure 3).^40^ The other independent molecule adopts the *gt* conformation, which is typical with galactosides. The *gg* rotamer is strongly disfavored in galactosides due to destabilizing 1,3-diaxial interactions, here between the fluorine substituents F(4) and F(6).^41^ Although signal overlap in the ^1^H NMR spectrum prevented a detailed conformational analysis, the low magnitude of the vicinal coupling constant ^3^ *J*_(H5–F6)_ = 12.3 Hz indicates the minimal population of the *gg* rotamer in a CDCl_3_ solution,^40,42,43^ as is typical for galactosides.

**Figure 3:**
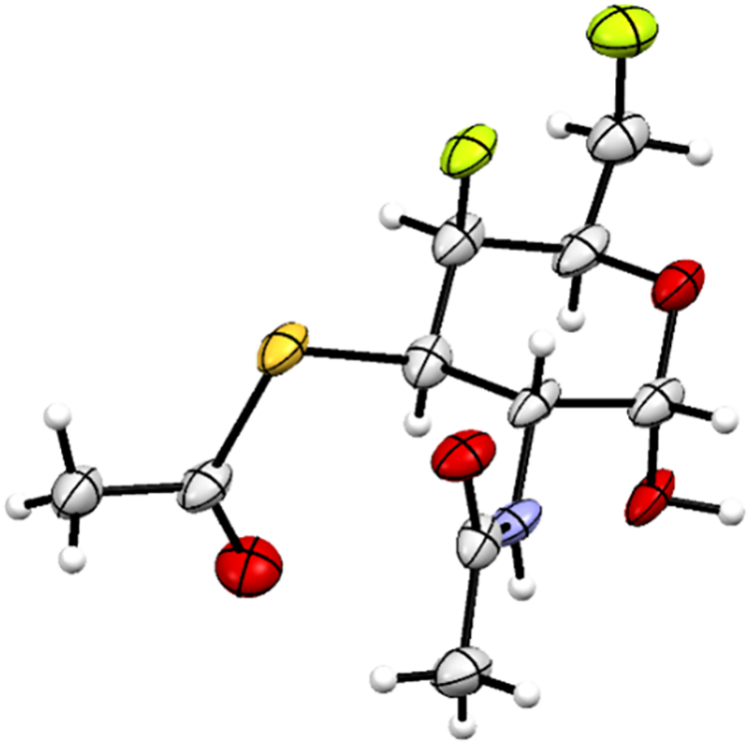
ORTEP projection showing the *gg* conformation of the exocyclic C(6)­H_2_F group adopted by one of the two independent molecules in the solid state of **53**.

**Scheme 5:**
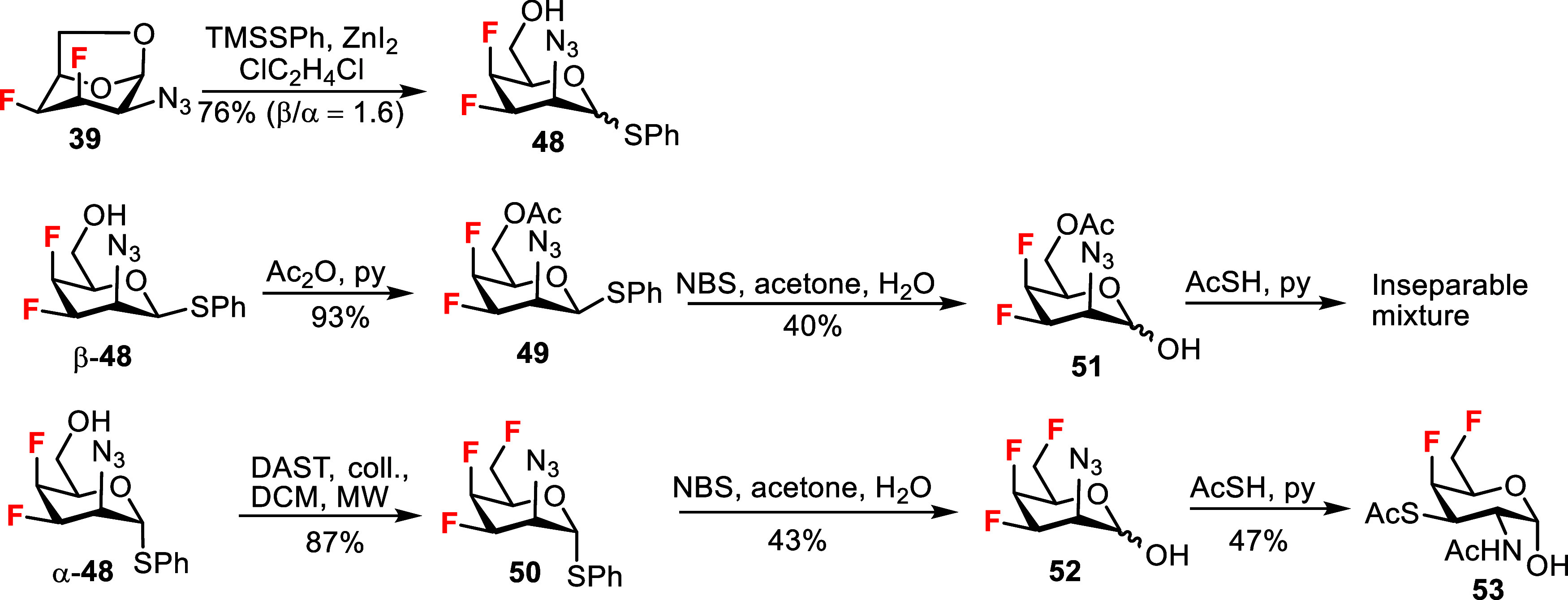
Synthesis of 3,4-Difluoro and 3,4,6-Trifluoro 2-Azido-taloses

Because the planned route to the desired 3,4-difluoro ManNAc analog from the intermediate **22** failed (Scheme 4), we decided to introduce the 3- and 4-fluorine substituents prior to the installation of the 2-azido group. Advantageously, we used the known 3,4-difluorinated 1,6-anhydroglucopyranose **54** available from levoglucosan in six steps as described by Linclau and Giguère (Scheme 6).^44^ Catalytic hydrogenation of compound **54** liberated the hydroxyl group at the 2-position to give the alcohol **55**. The hydroxyl group was activated as a trifluoromethanesulfonate ester and then substituted with azide, producing the desired d-*manno-*configured 1,6-anhydro-2-azido-3,4-difluoropyranose intermediate **56**. Reaction with TMSSPh afforded a mixture of α- and β-thioglycosides **57**. Only the β-anomer β-**57** was obtained as a pure compound by column chromatography in 42% yield. Acetylation of β-**57** with acetic anhydride produced thioglycoside **58**. NBS-promoted hydrolysis of the thiophenyl glycoside yielded hemiacetal **59**, and the final azide-to-acetamide conversion afforded the target 3,4-difluoro ManNAc analog **8**. Deoxyfluorination of β-**57** using a microwave-assisted reaction with DAST yielded trifluorinated compound **60**. No significant migration of the thiophenyl group was observed on TLC despite the β-configuration of the aglycone, which is favorable for migration.^31^ Subsequent NBS-promoted hydrolysis of the thiophenyl glycoside produced hemiacetal **61**. The azide-to-acetamide conversion by reaction with AcSH resulted in the target 3,4,6-trifluoro-ManNAc hemiacetal **11**. All final acylated deoxyfluorinated hemiacetals were obtained as a colorless gel-like mixture of both anomers, stable in air or in alcoholic or DMSO-*d*_6_ solution, with only limited solubility in water, but sufficient for biological assays.

**Scheme 6:**
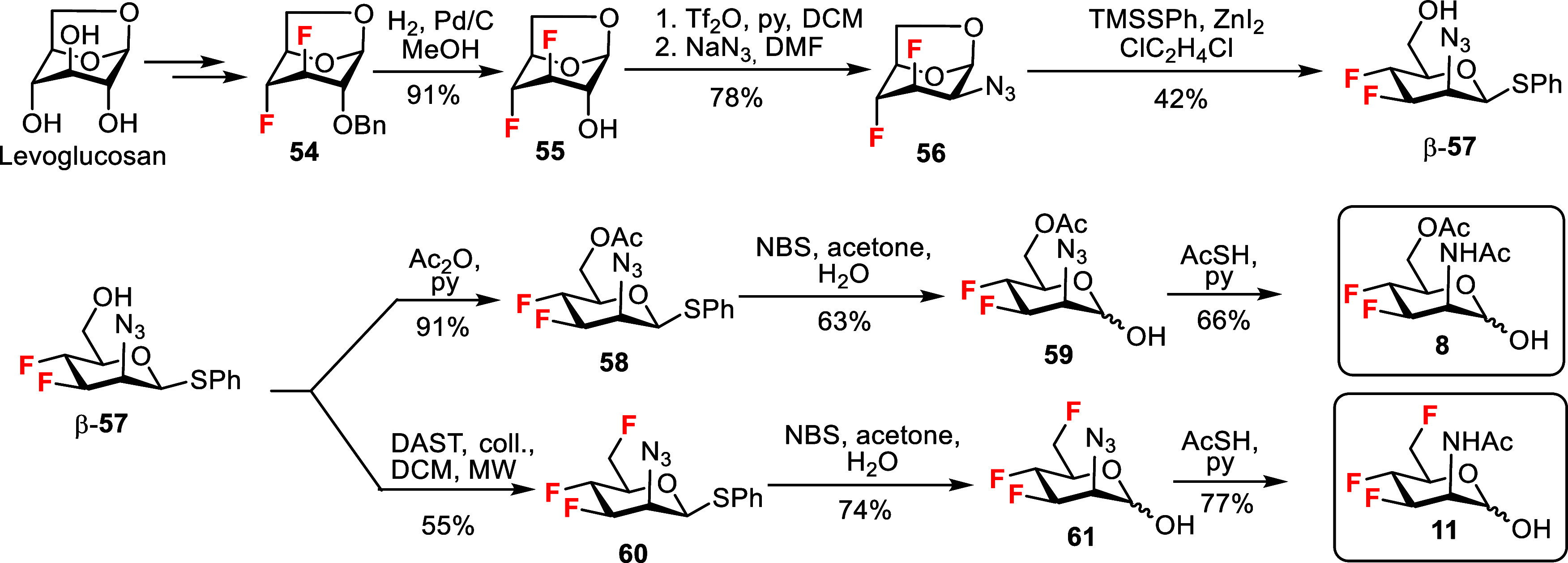
Synthesis of 3,4-Difluoro and 3,4,6-Trifluoro Analogs of Acetylated ManNAc Hemiacetals

### MTT Cytotoxicity Assay

The *in vitro* cytotoxicity of the prepared fluorinated hemiacetals was determined using the MTT cell viability assay after 72 h of treatment (Table 1) and expressed as *IC*_50_ values. We used two cell lines, the MDA-MB-231 cell line derived from human triple-negative breast adenocarcinoma, and the human mammary gland epithelial cell line MCF-10A, which is a nontumorigenic epithelial cell line. The MDA-MB-231 cell line was selected because it has previously shown sensitivity to nonfluorinated acylated mannosamines^15–17^ and fluorinated acylated GlcNAc and GalNAc.^19,20^ The MCF-10A cell line was selected as a control to evaluate the effects of the compounds on noncancerous cells. Selected cytotoxicity values against MDA-MB-231 cells for acetylated difluorinated and trifluorinated GlcNAc and GalNAc hemiacetals available in the literature^19^ are shown in Table 1 for comparison.

**Table 1:** Cytotoxicity Towards MDA-MB-231 and MCF-10A Cell Lines Expressed as *IC*_50_ [μM], Obtained after 72 h Treatment using the MTT Assay

entry	compound (substitution pattern)^a^	MDA-MB-231	MCF-10A
1	Ac_3_ManNAc	94 ± 9	>200
2	Bu_3_ManNAc	79 ± 13	>200
3	**3** (3F-Ac_2_ManNAc)	48 ± 2	57 ± 1
4	**4** (3F-Pr_2_ManNAc)	27 ± 1	37 ± 3
5	**5** (4F-Ac_3_ManN_3_)	>200	58 ± 4
6	**6** (4F-Ac_2_ManNAc)	46 ± 3	40 ± 1
7	**7** (6F-Ac_2_ManNAc)	>200	>200
8	**8** (3,4-diF-AcManNAc)	28 ± 2	38 ± 2
9	**9** (3,6-diF-AcManNAc)	>200	>200
10	**10** (4,6-diF-AcManNAc)	155 ± 20	>200
11	**11** (3,4,6-triF-ManNAc)	**21 ± 1**	**24 ± 2**
12	3,6-diF-AcGlcNAc	48 ± 9^b^	n.a.^c^
13	4,6-diF-AcGlcNAc	25 ± 10^b^	n.a.
14	4,6-diF-AcGalNAc	61 ± 5^b^	n.a.
15	4,6-diF-PrGalNAc	20 ± 4^b^	n.a.
16	2,3,6-triF-GlcNAc	29 ± 7^b^	n.a.
17	2,3,6-triF-GalNAc	28 ± 3^b^	n.a.
18	cisPt	10 ± 1	21 ± 1

a Bu = butyryl, Pr = propionyl.

b values taken from ref 19

c n.a., not available.

Nonfluorinated **Ac**_**3**_ **ManNAc** and **Bu**_**3**_ **ManNAc** showed moderate cytotoxicity with the butyryl analog showing slightly increased activity. Mono- and difluorinated analogs of ManNAc that contained a 6-fluoro substituent exhibited low or no antiproliferative activity. Thus, the 6-fluoro-ManNAc analog **7**, and the 3,6-difluoro-ManNAc analog **9** were completely ineffective (*IC*_50_ > 200 μM, Table 1, entries 7 and 9), while the 4,6-difluoro-mannosamine **10** showed weak cytotoxicity only against the MDA-MB-231 cells (entry 10). This contrasts with the corresponding GlcNAc and GalNAc hemiacetals, which all exhibited measurable cytotoxicity (see entries 12–15 for difluorinated GlcNAc).^19^ The azido analog **5** was cytotoxic only to the noncancerous MCF-10A cells (entry 5).

The remaining fluorinated ManNAc hemiacetals **3**, **4**, **6**, **8**, and **11** exhibited cytotoxic activity to both tested cell lines with the *IC*_50_ values ranging from 21 μM to 57 μM. The most cytotoxic compound to both cell lines was the 3,4,6-trifluoro-ManNAc analog **11** (entry 11), which performed slightly better than the corresponding GlcNAc and GalNAc analogs (entries 16 and 17). Its cytotoxicity toward MCF-10A cells was comparable to cisplatin. The 3,4-difluoro ManNAc analog **8** was only slightly less cytotoxic than trifluoro ManNAc **11**. No significant selectivity for the MDA-MB-231 cancer cell line was observed.

### Cell Proliferation Assay

In addition to assessing the metabolic activity and viability by the MTT assay, the cytotoxic compounds **4**, **6**, **8**, and **11** were tested in a cell proliferation assay at different concentrations based on the *IC*_50_ values obtained (10 μM, 25 μM, and 50 μM for compounds **4** and **6**; 10 μM, 20 μM, and 30 μM for compounds **8** and **11**). This assay served as a complementary experiment to the MTT assay that would also allow us to assess potential morphological changes. As no detectable morphological changes were observed and the results were consistent with the MTT assay, the experiment was performed in a single biological replicate. The obtained data (Figure 4) largely aligns with the *IC*_50_ values from the MTT assay, with one exception: the propionylated 3-fluoro ManNAc analog **4** was the least effective of all compounds tested in the cell proliferation assay, despite its relatively high cytotoxicity in the MTT assay (*IC*_50_ = 27 ± 1 μM). Conversely, the trifluorinated analog **11** exhibited the strongest antiproliferative effect among the tested compounds, consistent with the results of the MTT assay.

**Figure 4:**
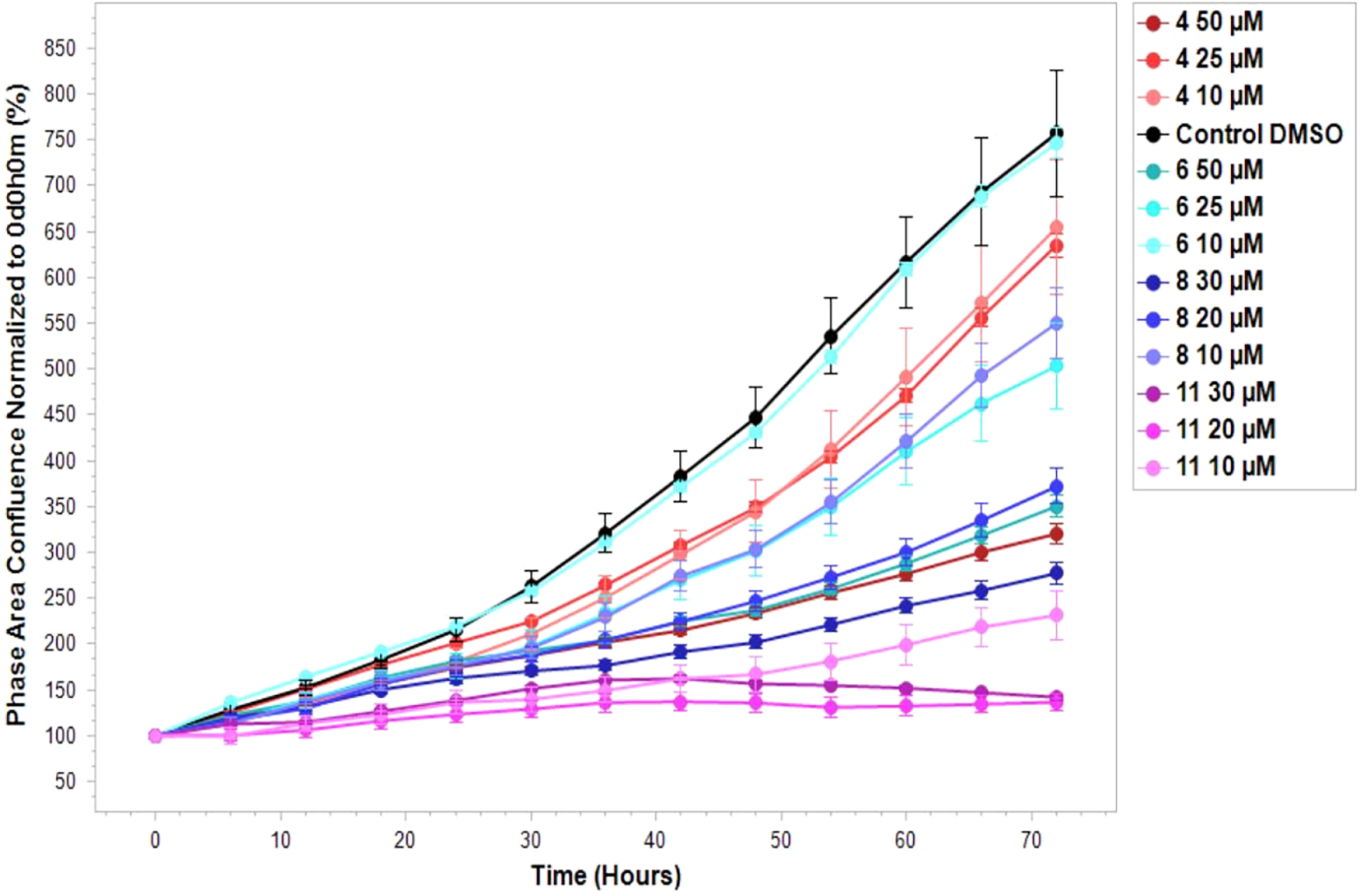
Cell proliferation assay of MDA-MB-231 treated with the cytotoxic compounds **4**, **6**, **8**, and **11**.

### Colony Forming Assay

We also evaluated the ability of MDA-MB-231 cells to survive and form colonies after treatment with the cytotoxic fluorinated hemiacetals **3**, **4**, **6**, **8**, and **11** at a concentration of 2.5 μM. The average number of colonies formed from at least ten separate wells for each treatment is displayed in the box plot graph in Figure 5. Control cells treated with DMSO formed an average of 34 colonies per well. Treatment with all compounds, except for 3-fluoro ManNAc analog **4**, resulted in a significant reduction in the number of colonies formed. The 3,4,6-trifluoro-ManNAc analog **11** was the most effective in inhibiting colony formation, reducing the average number of colonies to 18. This was followed by 3,4-difluoromannosamine **8**, which reduced the average number of colonies to 22.

**Figure 5:**
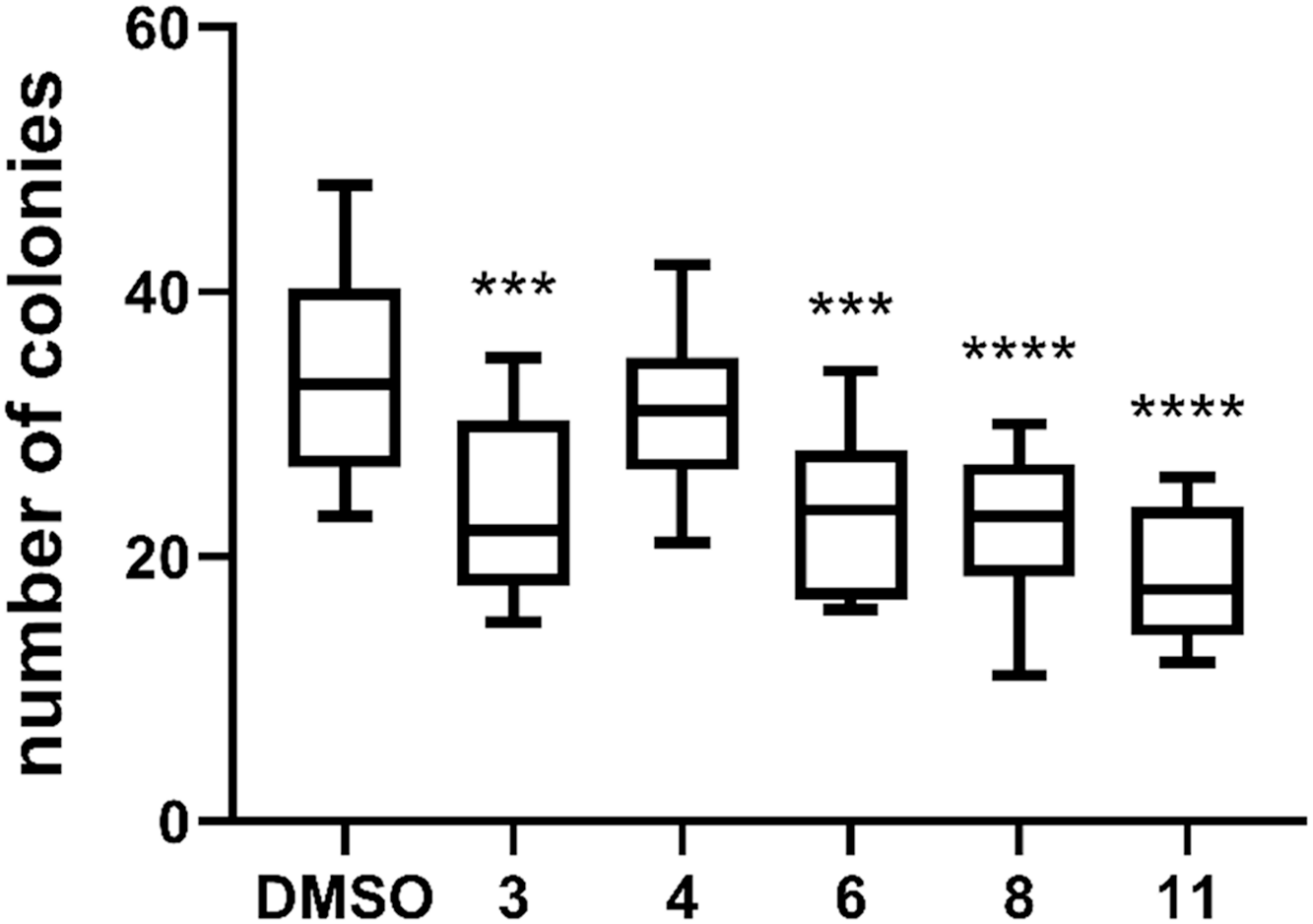
Colony forming assay of MDA-MB-231 cells treated with the cytotoxic compounds **3**, **4**, **6**, **8**, and **11**, with DMSO as control. Data presented in the box plot graph shows the number of colonies determined from five biological replicates for each compound. One-way ANOVA was performed, comparing the means of each compound applied in 2.5 μM concentration with respect to control (DMSO only); ** *P* ≤ 0.01; *** *P* ≤ 0.001; **** *P* ≤ 0.0001.

### Cell Cycle Analysis

Flow cytometry was performed to analyze the cell cycle of MDA-MB-231 cells treated with compounds **3**, **4**, **6**, **8**, and **11**; DMSO was used as the control. The data obtained (Figure S1) were analyzed using two-tailed *t* tests to determine if there were significant differences in the percentage of cells in each phase between the control and treated cells. None of the ManNAc analogs tested significantly disrupted the cell cycle.

### Western Blotting Analysis

To elucidate the mechanisms underlying the cytotoxic effects of the tested compounds, immunodetection using Western blotting (Figure 6) was performed to analyze selected proteins involved in apoptosis (PARP), DNA damage (γH2AX), cell cycle arrest (p21, cyclin B1, and cyclin D), monitoring of cellular energy balance and response to metabolic stress (AMPK and p-AMPK), and autophagy (p62 and LC3B). Treatment with the most cytotoxic 3,4,6-trifluoro-ManNAc analog **11** caused a distinct increase in γH2AX phosphorylation, indicating DNA damage, and a slight induction of PARP cleavage, suggesting the initiation of apoptotic processes. Compound **11** also showed upregulation of the autophagy markers p62 and LC3B.

**Figure 6:**
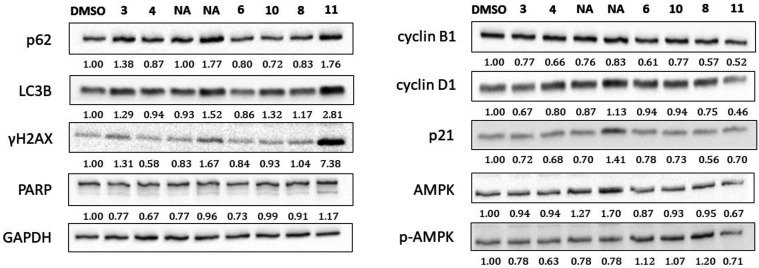
Representative Western blotting analysis of MDA-MB-231 cells treated with compounds **3**, **4**, **6**, **8**, **10**, **11** and DMSO serving as a control. The experiment was performed in three independent biological replicates; NA = Not Applicable.

### Wound Healing Assay

Compounds **5**, **7**, and **9** that were inactive against MDA-MB-231 cells in the MTT assay were tested for their antimigratory properties using a wound healing assay, as nonfluorinated ManNAc hemiacetals exhibited antimigratory properties in earlier studies.^15^ None of the tested compounds showed reduced cell migration. On the contrary, cells treated with these hexosamine analogs appeared to migrate even more than control cells treated with DMSO (Figure S2).

## Discussion

Motivated by the previous finding that fluorine introduction enhances the cytotoxicity of acylated GlcNAc and GalNAc hemiacetals, in this study, we synthesized fluorinated acetylated ManNAc hemiacetals and evaluated their *in vitro* antitumor activity. Previously, only two monofluorinated nonacetylated ManNAc analogs were reported: 4-fluoro-ManNAc^32^ and 6-fluoro-ManNAc.^45^ The synthesis of the fluorinated ManNAc hemiacetals was more complicated than we initially expected. Our experience, as well as that of the other groups,^46^ emphasizes the importance of gathering empirical results from which useful trends and patterns can emerge to guide future synthetic attempts. For example, a *trans*-diaxially disposed vicinal azido group appears to increase the likelihood of retentive deoxyfluorination on reaction with DAST as shown here for compound **12** (Scheme 1) and previously for methyl 3-azido-4,6-*O*-benzylidene-3-deoxy-α-d-altropyranoside^47^ and 1,6-anhydro-3-azido-3-deoxy-2-*O*-tosyl-β-d-glucopyranose.^48^ A possible explanation is neighboring group participation of the azido substituent^47–49^ in spite of the fact that an azide behaves as an inert and nonparticipating group in glycosylation with 2-azido-donors.^50–52^

Similarly, the deoxyfluorination of compound **38** corroborates the propensity of 1,6-anhydrotaloses to undergo retentive deoxyfluorination at the 4-position on reaction with DAST (Scheme 4A,B). If the proposed O5 participation is the correct reaction mechanism (Scheme 4C), this tendency of 1,6-anhydrotaloses is surprising because an antiperiplanar electronegative substituent at C2 (with respect to the endocyclic pyranose O5 oxygen)—for example an azide in **38** or fluorine in **40**—should reduce the electron-donating capacity of the pyranose O5 oxygen and its ability to form a cationic intermediate such as **47** (Scheme 4C), as previously noted by Linclau.^37^

Unexpected formation of product **53** from hemiacetal **52** in reaction with AcSH involves epimerization at the 2-position, suggesting that **53** is formed through an elimination-addition mechanism involving an unsaturated intermediate **62** (Scheme 7). A 1,4-conjugate addition of thioacetic acid and concomitant reduction of the azido group would then yield the GalNAc analog **53**. The formation of the 3-thiolated products **64** and **65**, which was previously reported for the reaction of the acetylated 3-fluoro-GlcNAc hemiacetal **63** with 2-phenylethanethiol, followed by acetylation, probably proceeds through the same mechanism with the elimination of hydrogen fluoride and addition of the thiol.^19^ Noteworthy, it was demonstrated that O-acetylated (nonfluorinated) GlcNAc hemiacetal can eliminate acetic acid and add the thiol group of cysteine at the 3-position via an elimination-addition mechanism. This reaction with cysteine residues has been identified as the cause of undesired S-glyco-modification of proteins.^53^

**Scheme 7:**
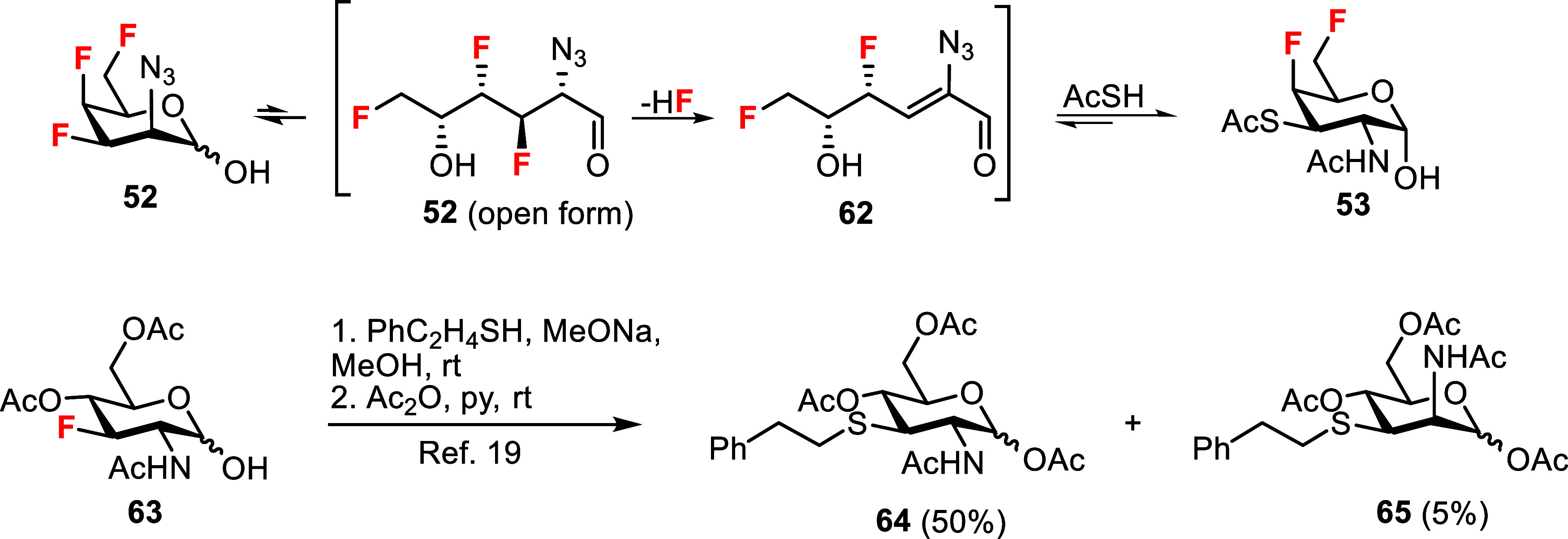
Suggested Mechanism for the Formation of 3-Thiolated Product **53** and a Related Reaction of **63** with 2-Phenylethanethiol Reported Previously^19^

Interestingly, no product resulting from migration of the β-linked thiophenyl aglycone from the anomeric to the 6-position was isolated after the microwave-assisted DAST deoxyfluorination of the thioglycoside β-**57**, indicating that this side reaction occurred to a negligible degree with this starting compound (Scheme 6). We have previously observed a significant migration with phenyl 2-azido-6-hydroxy-1-thio-β-galactoside β-**66**, which predominantly produced migration product **68**, in addition to desired 6-fluorothiogalactoside **67**, in microwave-assisted deoxyfluorination with DAST (Scheme 8).^28^ However, thioglucoside β-**69** was much less prone to migration, giving predominantly 6-fluorothioglucoside **70** together with a low quantity of the inseparable migration product **71**.^28^ Taken together, thioaglycone migration does not appear to be a general reaction mechanism with 2-azido-β-thioglycosides, and the extent of migration depends considerably on the configuration and substitution pattern of the starting thioglycoside.

**Scheme 8:**
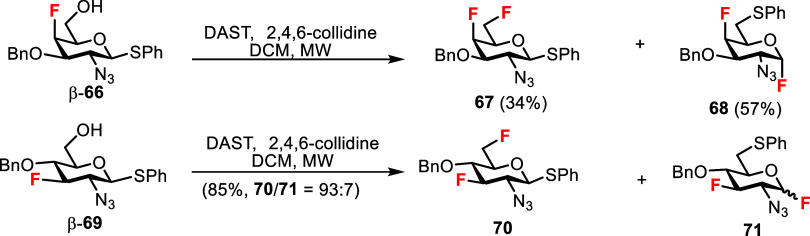
Previously Observed 1→6 Thioaglycone Migration of Phenyl 2-Azio-1-thio-β-Glycosides^28^

In contrast to acylated GlcNAc and GalNAc hemiacetals, where deoxyfluorination consistently increased cytotoxicity, the effect of deoxyfluorination on acetylated ManNAc hemiacetal was more variable and very sensitive to the fluorination pattern. Introducing fluorine at the 6-position mostly reduced the cytotoxicity compared to the nonfluorinated **Ac**_**3**_ **ManNAc** hemiacetal. For example, 6F–Ac_2_ManNAc **7** was not cytotoxic, however, 6-fluorination increased cytotoxicity of propionylated GlcNAc and GalNAc hemiacetals, albeit to a lesser extent than 3- and 4-fluorination.^19^ Strikingly, 3,6- and 4,6-diF-AcManNAc **9** and **10** were virtually noncytotoxic, while the corresponding acetylated GlcNAc and GalNAc counterparts had potent antiproliferative effects on MDA-MB-231 cells.^19^ The common trend for all fluorinated *N*-acetylhexosamine hemiacetals is that the trifluorinated analogs are among the most cytotoxic fluoro analogs. This applies to both GlcNAc and GalNAc analogs^19^ and is consistent with the finding that 3,4,6-triF-ManNAc **11** was the most cytotoxic of the compounds tested in this study. Together with 4,6-diF-PrGalNAc (Table 1, entry 15), it is one of the most cytotoxic fluorinated monosaccharides reported.

Direct molecular targets in cells responsible for the observed antiproliferative effects of fluorinated hemiacetals have not yet been identified. It has been hypothesized that nonspecific S-glyco-modification of cellular proteins contributes to the cytotoxic effects of hexosamine hemiacetals.^19^ It is possible that deoxyfluorination increases the levels of damaged S-glyco-modified proteins in cells due to higher tendency of fluorinated hexosamine hemiacetals to react by elimination-addition mechanism, but confirming this hypothesis is beyond the scope of this study. Additionally, certain fluorinated monosaccharides can act as metabolic inhibitors of specific cell-surface glycan structures.^5,54^ According to a previous report,^12^ however, this metabolic perturbation of the cell-surface glycome did not correlate with the antiproliferative activity of the hemiacetal 4F-Ac_2_GlcNAc in human prostate cancer PC-3 cells, suggesting that the glycome remodeling alone is not responsible for the antiproliferative activity of this fluoro analog.

Western blot analysis of selected stress and death-related proteins revealed that compound **11**, which exhibited the highest cytotoxicity, antiproliferative effect, and inhibition of the formation of colonies induced pronounced molecular changes consistent with multifactorial cell stress responses. Notably, γH2AX, a marker of DNA double-strand breaks, was strongly upregulated in response to compound **11**, with a ∼7.4-fold increase compared to control, indicating extensive DNA damage. This was accompanied by an increase in LC3B and p62 levels, which is consistent with the activation of autophagy, as well as PARP cleavage, a hallmark of apoptosis. Although changes in AMPK and p-AMPK expression were also observed, suggesting metabolic stress modulation, it does not seem to be a dominant mechanism.

Furthermore, Western blot analysis revealed a downregulation of cyclin B1 and cyclin D1. However, these changes were not manifested by a detectable shift in cell cycle distribution in flow cytometric analysis of DNA content (Figure S1), indicating that compound **11** does not induce a significant cell cycle arrest under the tested conditions. This indicates that the observed antiproliferative activity is likely mediated through mechanisms other than cell cycle blockade, such as DNA damage, apoptosis, and autophagy, as supported by γH2AX accumulation, PARP cleavage, and LC3B upregulation.

Taken together, these findings suggest that the antiproliferative activity of compound **11** is mediated through a multimodal cellular response, involving DNA damage, apoptosis, and autophagy, rather than through direct cell cycle arrest or modulation of the surface glycome. While the precise molecular targets remain unidentified, the consistent activation of stress and death-related pathways supports the notion that this trifluorinated hexosamine analog elicits a complex and multifactorial mechanism of action.

## Conclusion

Although the synthesis of fluorinated analogs of *N*-acetylmannosamine posed a greater challenge than the synthesis of *N*-acetylglucosamine and galactosamine analogs reported previously,^19^ a complete set of deoxyfluorinated O-acetylated ManNAc hemiacetals has been prepared. The synthesis involves the preparation of advanced polyfluorinated intermediates, including polyfluorinated phenyl 2-azido-thiomannosides and -talosides. These thioglycosides are fluorinated glycosyl donors for challenging 1,2-*cis*-glycosylation. Retentive deoxyfluorination of 2-azido-altropyranoside **12** suggests that the *trans-*diaxially positioned azido group provides anchimeric assistance in the nucleophilic deoxyfluorination with DAST. Retentive deoxyfluorination with DAST at the equatorial 4-position in 1,6-anhydro-talopyranose derivatives likely proceeds via participation of the pyranose ring oxygen and is a valuable route to 4-fluoro-talopyranoses. Introducing fluorine at the 3-position makes hexosamine-derived hemiacetals susceptible to the elimination of hydrogen fluoride and subsequent conjugate addition, as demonstrated by compound **53**. Our results also indicate that the migration of thioaglycones from β-thioglycosides considerably depends on the substitution pattern and configuration. It can occur to an insignificant extent with some 2-azidohexosamine β-thioglycosides, not compromising the yield of the desired fluorinated sugars.

A common trend for ManNAc, GalNAc and GlcNAc hemiacetals is that their 3,4,6-trideoxyfluorinated analogs showed significant cytotoxic effects. In contrast, monodeoxy- and dideoxyfluorinated analogs displayed variable activity likely influenced by the position and relative configuration of the fluorine substituents. The potent but unselective antiproliferative activity exhibited by the trifluoro ManNAc analog **11** was confirmed by three independent cell-based assays. This cytotoxicity was consistently associated with DNA damage, apoptosis, and autophagy, supporting a multifactorial mechanism of action. These findings highlight the promise of trifluorinated amino sugar hemiacetals as cytotoxic scaffolds for further biomedical research.

## Safety Statement

Reactions with DAST are potentially hazardous. Appropriate personal protective equipment, including gloves and eye protection, should be worn, and reaction should be conducted with caution. According to the empirical safety rule for organic azides, a compound is considered safe to handle if the number of nitrogen atoms does not exceed the number of carbon atoms, and if (*N*_C_ + *N*_O_)/*N*_N_ ≥ 3. In this formula, *N* is the number of atoms. All synthesized azide-containing compounds fulfill these criteria and are therefore not expected to exhibit explosive or impact-sensitive behavior.

## Supplementary Material





## Data Availability

The data underlying this study are available in the published article and its Supporting Information.

## Notes

Experimental procedures (Synthesis and characterization of new compounds, MTT assay, cell proliferation assay, colony forming assay, cell cycle analysis, wound healing assay, SDS-PAGE and western blotting, and X-ray) (PDF)
Copies of NMR spectra (PDF)

## References

[1] Linclau B., Arda A., Reichardt N. C., Sollogoub M., Unione L., Vincent S. P., Jimenez-Barbero J. (2020). Fluorinated
carbohydrates as chemical probes for molecular recognition studies.
Current status and perspectives. Chem. Soc.
Rev..

[2] Stephenson E. L., Zhang P., Ghorbani S., Wang A., Gu J., Keough M. B., Rawji K. S., Silva C., Yong V. W., Ling C.-C. (2019). Targeting the Chondroitin Sulfate Proteoglycans: Evaluating
Fluorinated Glucosamines and Xylosides in Screens Pertinent to Multiple
Sclerosis. ACS Cent. Sci..

[3] Keough M. B., Rogers J. A., Zhang P., Jensen S. K., Stephenson E. L., Chen T., Hurlbert M. G., Lau L. W., Rawji K. S., Plemel J. R., Koch M., Ling C.-C., Yong V. W. (2016). An inhibitor
of chondroitin sulfate proteoglycan synthesis promotes central nervous
system remyelination. Nat. Commun..

[4] Pijnenborg J. F. A., Rossing E., Merx J., Noga M. J., Titulaer W. H. C., Eerden N., Veizaj R., White P. B., Lefeber D. J., Boltje T. J. (2021). Fluorinated rhamnosides
inhibit cellular fucosylation. Nat. Commun..

[5] Rossing E., Pijnenborg J. F. A., Boltje T. J. (2022). Chemical tools to track and perturb
the expression of sialic acid and fucose monosaccharides. Chem. Commun..

[6] Moons S. J., Rossing E., Heming J. J. A., Janssen M. A. C. H., van Scherpenzeel M., Lefeber D. J., de Jonge M. I., Langereis J. D., Boltje T. J. (2021). Structure–Activity Relationship of Fluorinated
Sialic Acid Inhibitors for Bacterial Sialylation. Bioconjugate Chem..

[7] Barthel S. R., Antonopoulos A., Cedeno-Laurent F., Schaffer L., Hernandez G., Patil S. A., North S. J., Dell A., Matta K. L., Neelamegham S., Haslam S. M., Dimitroff C. J. (2011). Peracetylated
4-Fluoro-glucosamine Reduces the Content and Repertoire of N- and
O-Glycans without Direct Incorporation. J. Biol.
Chem..

[8] Marathe D. D., Buffone A., Chandrasekaran E. V., Xue J., Locke R. D., Nasirikenari M., Lau J. T. Y., Matta K. L., Neelamegham S. (2010). Fluorinated
per-acetylated GalNAc metabolically alters glycan structures on leukocyte
PSGL-1 and reduces cell binding to selectins. Blood.

[9] Liu Y., Sweet I. R., Boons G.-J. (2024). 2,2-Difluoro Derivatives of Fucose
Can Inhibit Cell Surface Fucosylation without Causing Slow Transfer
to Acceptors. JACS Au.

[10] Goon S., Bertozzi C. R. (2002). METABOLIC SUBSTRATE
ENGINEERING AS A TOOL FOR GLYCOBIOLOGY. J. Carbohydr.
Chem..

[11] Dai Y., Hartke R., Li C., Yang Q., Liu J. O., Wang L. X. (2020). Synthetic Fluorinated l-Fucose Analogs Inhibit
Proliferation of Cancer Cells and Primary Endothelial Cells. ACS Chem. Biol..

[12] Nishimura S.-I., Hato M., Hyugaji S., Feng F., Amano M. (2012). Glycomics
for Drug Discovery: Metabolic Perturbation in Androgen-Independent
Prostate Cancer Cells Induced by Unnatural Hexosamine Mimics. Angew. Chem., Int. Ed..

[13] Sharma M., Bernacki R. J., Hillman M. J., Korytnyk W. (1993). Fluorinated carbohydrates
as potential plasma membrane modifiers. Synthesis of 3-deoxy-3-fluoro
derivatives of 2-acetamido-2-deoxy-d-hexopyranoses. Carbohydr. Res..

[14] Sharma M., Bernacki R. J., Paul B., Korytnyk W. (1990). Fluorinated
carbohydrates
as potential plasma membrane modifiers. Synthesis of 4- and 6-fluoro
derivatives of 2-acetamido-2-deoxy-d-hexopyranoses. Carbohydr. Res..

[15] Campbell C. T., Aich U., Weier C. A., Wang J. J., Choi S. S., Wen M. M., Maisel K., Sampathkumar S.-G., Yarema K. J. (2008). Targeting Pro-Invasive Oncogenes
with Short Chain Fatty
Acid-Hexosamine Analogues Inhibits the Mobility of Metastatic MDA-MB-231
Breast Cancer Cells. J. Med. Chem..

[16] Aich U., Campbell C. T., Elmouelhi N., Weier C. A., Sampathkumar S. G., Choi S. S., Yarema K. J. (2008). Regioisomeric
SCFA Attachment to
Hexosamines Separates Metabolic Flux from Cytotoxicity and MUC1 Suppression. ACS Chem. Biol..

[17] Elmouelhi N., Aich U., Paruchuri V. D. P., Meledeo M. A., Campbell C. T., Wang J. J., Srinivas R., Khanna H. S., Yarema K. J. (2009). Hexosamine
Template. A Platform for Modulating Gene Expression and for Sugar-Based
Drug Discovery. J. Med. Chem..

[18] Obidiro O., Battogtokh G., Akala E. O. (2023). Triple Negative Breast Cancer Treatment
Options and Limitations: Future Outlook. Pharmaceutics.

[19] Hamala V., Št’astná L. C., Kurfiřt M., Cuřínová P., Balouch M., Hrstka R., Voňka P., Karban J. (2021). The effect of deoxyfluorination and
O-acylation on the cytotoxicity of N-acetyl-d-gluco- and d-galactosamine hemiacetals. Org. Biomol.
Chem..

[20] Hamala V., Ondrásková K., Stastná L. C., Krcil A., Müllerová M., Kurfirt M., Hirsová K., Holcaková J., Gyepes R., Císarova I., Bernásková J., Hrstka R., Karban J. (2024). Improving
the anticancer activity of fluorinated glucosamine and galactosamine
analogs by attachment of a ferrocene or ruthenium tetrazene motif. Appl. Organomet. Chem..

[21] Guthrie R. D., Murphy D. (1963). 1009. Nitrogen-containing carbohydrate derivatives.
Part IV. Some azido- and epimino-sugars. J.
Chem. Soc..

[22] Kurfiřt M., Dračínský M., Št’astná L. C., Cuřínová P., Hamala V., Hovorková M., Bojarová P., Karban J. (2021). Selectively Deoxyfluorinated N-Acetyllactosamine
Analogues as 19F NMR Probes to Study Carbohydrate-Galectin Interactions. Chem. - Eur. J..

[23] Lainé D., Denavit V., Lessard O., Carrier L., Fecteau C.-É., Johnson P. A., Giguère D. (2020). Fluorine effect in nucleophilic fluorination
at C4 of 1,6-anhydro-2,3-dideoxy-2,3-difluoro-β-D-hexopyranose. Beilstein J. Org. Chem..

[24] Krist P., Kuzma M., Pelyvás I. F., Simerská P., Křen V. (2003). Synthesis of 4-nitrophenyl 2-acetamido-2-deoxy-β-d-mannopyranoside and 4-nitrophenyl 2-acetamido-2-deoxy-α-d-mannopyranoside. Collect. Czech. Chem.
Commun..

[25] Lainé D., Denavit V., Giguère D. (2017). Synthesis of Protected 3-Deoxy-3-fluoro-
and 4-Deoxy-4-fluoro-d-galactopyranosides from Levoglucosan. J. Org. Chem..

[26] Niemietz M., Perkams L., Hoffman J., Eller S., Unverzagt C. (2011). Selective
oxidative debenzylation of mono- and oligosaccharides in the presence
of azides. Chem. Commun..

[27] Wang L.-X., Sakairi N., Kuzuhara H. (1990). 1,6-Anhydro-β-d-glucopyranose
derivatives as glycosyl donors for thioglycosidation reactions. J. Chem. Soc., Perkin Trans. 1.

[28] Hamala V., Št’astná L. C., Kurfiřt M., Cuřínová P., Dračínský M., Karban J. (2021). Synthesis of multiply
fluorinated N-acetyl-D-glucosamine
and D-galactosamine analogs via the corresponding deoxyfluorinated
glucosazide and galactosazide phenyl thioglycosides. Beilstein J. Org. Chem..

[29] Kolakowski R. V., Shangguan N., Sauers R. R., Williams L. J. (2006). Mechanism of Thio
Acid/Azide Amidation. J. Am. Chem. Soc..

[30] Mersch C., Wagner S., Hoffmann-Röder A. (2009). Synthesis
of Fluorinated
Analogues of Tumor-Associated Carbohydrate and Glycopeptide Antigens. Synlett.

[31] Lin P.-C., Adak A. K., Ueng S.-H., Huang L.-D., Huang K.-T., Ho J. A., Lin C.-C. (2009). DAST-Mediated
Regioselective Anomeric
Group Migration in Saccharides. J. Org. Chem..

[32] Hartlieb S., Günzel A., Gerardy-Schahn R., Münster-Kühnel A. K., Kirschning A., Dräger G. (2008). Chemoenzymatic synthesis of CMP-N-acetyl-7-fluoro-7-deoxy-neuraminic
acid. Carbohydr. Res..

[33] Wheatley D. E., Fontenelle C. Q., Kuppala R., Szpera R., Briggs E. L., Vendeville J.-B., Wells N. J., Light M. E., Linclau B. (2021). Synthesis
and Structural Characteristics of all Mono- and Difluorinated 4,6-Dideoxy-d-xylo-hexopyranoses. J. Org. Chem..

[34] Sugawara T., Igarashi K. (1988). Synthesis of a trisaccharide component of the capsular
polysaccharide of Streptococcus pneumoniae type 19F. Carbohydr. Res..

[35] Denavit V., Lainé D., St-Gelais J., Johnson P. A., Giguère D. (2018). A Chiron approach
towards the stereoselective synthesis of polyfluorinated carbohydrates. Nat. Commun..

[36] Karban J., Cisarova I., Strasak T., Stastna L. C., Sykora J. (2012). Skeletal rearrangements
resulting from reactions of 1,6:2,3- and 1,6:3,4-dianhydro-β-d-hexopyranoses with diethylaminosulphur trifluoride. Org. Biomol. Chem..

[37] Quiquempoix L., Wang Z., Graton J., Latchem P. G., Light M., Le Questel J.-Y., Linclau B. (2019). Synthesis of 2,3,4-Trideoxy-2,3,4-trifluoroglucose. J. Org. Chem..

[38] Albert R., Dax K., Link R. W., Stütz A. E. (1983). Carbohydrate triflates: reaction
with nitrite, leading directly to epi-hydroxy compounds. Carbohydr. Res..

[39] Wray V. (1976). The carbon-13
nuclear magnetic resonance spectra of the deoxyfluoro-d-glucoses,
2-deoxy-2-fluoro-d-mannose, and 4-deoxy-4-fluoro-d-galactose. Orientational and substituent effects upon JFC. J. Chem. Soc., Perk. Trans..

[40] Denavit V., Lainé D., Bouzriba C., Shanina E., Gillon É., Fortin S., Rademacher C., Imberty A., Giguère D. (2019). Stereoselective
Synthesis of Fluorinated Galactopyranosides as Potential Molecular
Probes for Galactophilic Proteins: Assessment of Monofluorogalactoside–LecA
Interactions. Chem. - Eur. J..

[41] Bock K., Duus J. (1994). A Conformational Study
of Hydroxymethyl Groups in Carbohydrates Investigated
by 1H NMR Spectroscopy. J. Carbohydr. Chem..

[42] Fernández P., Jiménez-Barbero J. (1994). The Conformation of Some Halodeoxy
Analogues of Methyl β-Lactoside in D_2_O and DMSO-d_6_ Solutions. J. Carbohydr. Chem..

[43] Kurfiřt M., Št’astná L. Č., Dračínský M., Pohl R., Císařová I., Sýkora J., Balouch M., Baka M., Hamala V., Cañada F. J., Ardá A., Jiménez-Barbero J., Karban J. (2024). Influence of Selective Deoxyfluorination on the Molecular
Structure of Type-2 *N*-Acetyllactosamine. J. Org. Chem..

[44] Fontenelle C. Q., Shishmarev D., Kuchel P. W., Linclau B. (2017). The Synthesis of 3,4-dideoxy-3,4-difluoro-d-glucose. Trends Carbohydr. Res..

[45] Khedri Z., Muthana M. M., Li Y., Muthana S. M., Yu H., Cao H., Chen X. (2012). Probe sialidase
substrate specificity using chemoenzymatically
synthesized sialosides containing C9-modified sialic acid. Chem. Commun..

[46] Willén D., Bengtsson D., Clementson S., Tykesson E., Manner S., Ellervik U. (2018). Synthesis
of Double-Modified Xyloside Analogues for
Probing the β4GalT7 Active Site. J. Org.
Chem..

[47] Vera-Ayoso Y., Borrachero P., Cabrera-Escribano F., Carmona A. T., Gómez-Guillén M. (2004). Fluorination
of 2-hydroxy-hexopyranosides by DAST: towards formyl C-glycofuranosides
from equatorial-2-OH methyl hexopyranosides. Tetrahedron: Asymmetry.

[48] Karban J., Sýkora J., Kroutil J., Císařová I., Padělková Z., Buděšínský M. (2010). Synthesis
of All Configurational Isomers of 1,6-Anhydro-2,3,4-trideoxy-2,3-epimino-4-fluoro-β-d-hexopyranoses. J. Org. Chem..

[49] Hanessian S., Saavedra O. M., Vilchis-Reyes M. A., Llaguno-Rueda A. M. (2014). Synthesis
of 4′-deoxy-4′-fluoro neamine and 4′-deoxy-4′-fluoro
4′-epi neamine. MedChemComm.

[50] van
der Vorm S., Overkleeft H. S., van der Marel G. A., Codée J. D. C. (2017). Stereoselectivity of Conformationally Restricted Glucosazide
Donors. J. Org. Chem..

[51] Hagen B., Ali S., Overkleeft H. S., van der Marel G. A., Codée J. D. C. (2017). Mapping the Reactivity and Selectivity
of 2-Azidofucosyl
Donors for the Assembly of N-Acetylfucosamine-Containing Bacterial
Oligosaccharides. J. Org. Chem..

[52] Kurfiřt M., Št’astná L. C., Dračínský M., Müllerová M., Hamala V., Cuřínová P., Karban J. (2019). Stereoselectivity in Glycosylation with Deoxofluorinated
Glucosazide and Galactosazide Thiodonors. J.
Org. Chem..

[53] Qin K., Zhang H., Zhao Z., Chen X. (2020). Protein S-Glyco-Modification
through an Elimination–Addition Mechanism. J. Am. Chem. Soc..

[54] Costa A. F., Teixeira A., Reis C. A., Gomes C. (2025). Novel anticancer drug
discovery efforts targeting glycosylation: the emergence of fluorinated
monosaccharides analogs. Expert. Opin. Drug.
Discovery.

