# Supplementary material for: Synthesis and Antiproliferative Activity of Fluorinated N‑Acetylmannosamine Analogs
Source: J Org Chem. 2026 Mar 19;91(13):4645–56. doi: 10.1021/acs.joc.5c03084 (PMC13054866; doi:10.1021/acs.joc.5c03084)
Supplement: Supplementary file 1 [file jo5c03084_si_001.pdf]

Synthesis and Antiproliferative Activity of Fluorinated *N*-Acetylmannosamine Analogs

Aleš Krčil,[a,b] Lucia Šutvajová,[c,d] Vojtěch Hamala,[a] Ivana Císařová,[e] Martin Kurfiřt,[a,b] Lucie Červenková Šťastná,[a] Jana Bernášková,[a] Roman Hrstka,[*,d] Jindřich Karban,[*,a]

[a] *Institute of Chemical Process Fundamentals of the CAS, v. v. i., Rozvojová 1/135, 165 00 Praha, Czech Republic. E-mail: karban@icpf.cas.cz.*
[b] *Department of Organic Chemistry, University of Chemistry and Technology, Technická 5, 166 28 Praha, Czech Republic.*
[c] *Department of Experimental Biology, Faculty of Science, Masaryk University, Kotlářská 2, 611 37, Brno, Czech Republic.*
[d] *Research Centre for Applied Molecular Oncology, Masaryk Memorial Cancer Institute, Žlutý kopec 7, 656 53 Brno, Czech Republic.*
[e] *Department of Inorganic Chemistry, Faculty of Science, Charles University, Hlavova 8, CZ-128 43 Praha 2, Czech Republic.*
[*]E-mail: *roman.hrstka@mou.cz*, *karban@icpf.cas.cz*

Supporting Information File I

Experimental procedures

Table of Contents

A. General procedures S2

B. Synthesis and compound characterization S4

C. *In vitro* biological activity S36

C1. MTT assay S36

C2. Cell proliferation assay S36

C3. Colony forming assay S36

C4. Cell cycle analysis S36

C5. Wound healing assay S37

C6. SDS-PAGE and western blotting S38

D. X-Ray S40

E. References: S43

A. General procedures

Chemicals were used as received. Petroleum ether fraction (with boiling point 40–65 °C) was distilled before use. TLC was carried out with Sigma-Aldrich TLC Silica gel 60 F_{254} and spots were detected by a UV detection at 254 nm or visualized with an anisaldehyde solution ($EtOH/AcOH/H_2SO_4$). Column chromatography was performed with silica gel 60 (70–230 mesh, Material Harvest). If not specified, solutions were concentrated under reduced pressure at temperatures below 45 °C. Anhydrous sodium sulfate was used to dry solutions after aqueous workup. Crystalline samples for characterization were obtained by slow cooling from solvent mixtures. The composition of these solvent mixtures is specified under individual syntheses. Microwave heating of reaction mixtures was conducted in a Monowave 300 reactor (Anton Paar) with sealed glass reaction vessels. An external infrared sensor monitored the reaction temperature, which was controlled by the instrument software. The reaction temperature and the time period during which it was held are reported under individual syntheses. NMR spectra were recorded using Bruker Avance 400 (^{1}H at 400.1 MHz, ^{19}F at 376.4 MHz, ^{13}C at 100.6 MHz) at 25 °C. The ^{1}H and ^{13}C NMR spectra were referenced to the solvent (δ/ppm; δH/δC: $CDCl_3$, 7.26/77.16, MeOH-d_4, 3.31/49.00, DMSO-d_6, 2.50/39.52). The ^{19}F NMR spectra were referenced to the line of the internal or external standard hexafluorobenzene (δ/ppm; −163.00 in $CDCl_3$, −166.62 in MeOH-d_4, −163.86 in DMSO-d_6). The structural assignment of proton and carbon NMR spectra was made by a combination of 1D and 2D NMR measurements: ^{1}H-^{1}H gCOSY, ^{1}H-^{13}C gHSQC, ^{1}H-^{13}C gHMBC, and ^{1}H-^{13}C gHSQC TOCSY. HRMS analyses were done using Bruker MicrOTOF-QIII, using APCI or ESI ionization in the positive mode. The m/z value of an $[M – N_2 + H]^+$ adduct is usually reported for azide-containing sugars because the molecular ion adducts were usually undetectable or extremely weak in abundance. EtOAc stands for ethyl acetate, PE for petroleum ether, DCM for dichloromethane, MeOH for methanol, MTBE for methyl *tert*-butyl ether, DMSO for dimethyl sulfoxide, DMF for dimethylformamide and DAST for diethylaminosulfur trifluoride. **CAUTION**: Reactions with DAST are potentially hazardous. Appropriate personal protective equipment, including gloves and eye protection, should be worn, and reaction should be conducted with caution. According to the empirical safety rule for organic azides, a compound is considered safe to handle if the number of nitrogen atoms does not exceed the number of carbon atoms, and if $(N_C + N_O)/N_N \geq 3$. In this formula, N is the number of atoms. All synthesized azide-containing compounds fulfill these criteria and are therefore not expected to exhibit explosive or impact-sensitive behavior.

General procedure for reactions of 1,6-anhydropyranoses with phenyl trimethylsilyl sulfide

To a solution of the starting deoxyfluorinated 1,6-anhydrohexopyranose (1 equiv) in dry 1,2-dichloroethane (c ≈ 0.2–0.3 mol/L) phenyl trimethylsilyl sulfide (TMSSPh, 3.1–3.4 equiv) and ZnI_2 (1.7–1.8 equiv) were added sequentially under argon atmosphere and the reaction was stirred vigorously

with the exclusion of light and moisture at rt for 48–72 h until TLC analysis indicated complete consumption of the starting compound. During TLC analysis, varying intensity spots of C6-OH products were observed near the origin. Subsequently, the reaction mixture was diluted with DCM, filtered, and washed with water. The water phase was then extracted with DCM (3×). The organic extracts were combined, dried, and concentrated. The resulting crude product was dissolved in methanol (with a concentration of approximately 0.1 mmol/L) and acidified with a few drops of AcOH. The solution was stirred at room temperature for 1–2 h to remove the 6-O-trimethylsilyl group, as indicated by TLC analysis. Finally, the solution was concentrated and subjected to purification by column chromatography on silica gel.

General procedure for C6 deoxyfluorination

DAST (1.16–1.33 equiv per reacting hydroxyl group) and 2,4,6-collidine (2.07–2.65 equiv per reacting hydroxyl group) were added dropwise in sequence to a solution of the initial alcohol (1 equiv) in DCM (*c* 0.05–0.11 mol/L). The reaction mixture was then heated to 80 °C for 1 hour using microwave irradiation (**CAUTION**: Reactions with DAST in microwave reactors are potentially hazardous. Appropriate personal protective equipment, including gloves and eye protection, should be worn, and reaction should be conducted with caution). TLC analysis using a solvent system of ethyl acetate and petroleum ether (1:3) indicated the complete consumption of all starting material and the formation of less polar products. The reaction mixture was quenched by adding methanol, followed by dilution with dichloromethane and washing with a 1% aqueous solution of HCl. The aqueous phase was then extracted with DCM (3×). The organic extracts were combined, dried, and concentrated. The resulting crude product was purified by column chromatography on silica gel.

General procedure for thioglycoside hydrolysis

The starting phenyl thioglycoside (1 equiv) was dissolved in a mixture of acetone and water (9:1, v/v, *c* 0.03–0.05 mol/L). NBS was added in excess (3.5–4.5 equiv), and the reaction mixture was stirred at room temperature for approximately 1 h. Initially, the reaction mixture turned red and then gradually became colorless. Once TLC analysis indicated complete consumption of the starting compound, the reaction was quenched by adding an aqueous solution of $Na_2S_2O_3$. The mixture was then diluted with DCM and washed with water. The aqueous phase was extracted with DCM (3×). The organic extracts were combined, dried, and concentrated. The resulting crude product was purified by column chromatography on silica gel.

General procedure for azide to acetamide conversion

2-Azidohexose was dissolved in pyridine (0.8–1.1 mL per 1 mmol of the 2-azidohexose) and thioacetic acid (0.8–1.1 mL per 1 mmol of the 2-azidohexose) and the resulting solution (final c = 0.45–0.65

mol/L) was stirred overnight. The reaction mixture may turn into a thick paste during this period. It was concentrated, co-distilled with toluene and dry-loaded onto a chromatographic column for purification.

General procedure for acid catalyzed acetolysis

A stock solution for acid catalyzed acetolysis was prepared by mixing 15 mL of Ac_2O and 0.6 mL H_2SO_4. Starting methyl glycoside was dissolved in a prepared stock solution (the volume is specified for each reaction, c ≈ 0.2–0.4 mol/L) and the reaction mixture was stirred at rt overnight until TLC indicated an absence of the starting material and a presence of one slightly more polar compound. The reaction mixture was diluted with EtOAc and quenched by an addition of saturated solution of $NaHCO_3$. The phases were separated, the aqueous phase was extracted with EtOAc (3×). The organic extracts were combined, dried, and concentrated. The resulting crude product was purified by column chromatography on silica gel.

B. Synthesis and compound characterization

2-Acetamido-3,4,6-tri-*O*-acetyl-D-mannopyranose Ac_3ManNAc

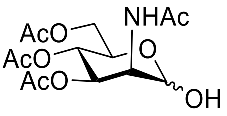

Compound **Ac_3ManNAc** was prepared according to the known procedure.[1]

2-Acetamido-3,4,6-tri-*O*-butyryl-D-mannopyranose Bu_3ManNAc

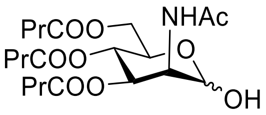

Compound **Bu_3ManNAc** was prepared according to the known procedure.[1]

2-Acetamido-4,6-di-*O*-acetyl-2,3-dideoxy-3-fluoro-D-mannopyranose (3)

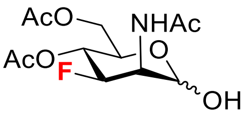

Compound **3** was prepared from compound **26** (82 mg, 0.28 mmol) according to the general procedure for azide to acetamide conversion using pyridine (0.3 mL, 3.7 mmol) and thioacetic acid (0.3 mL, 4.2 mmol). Column chromatography on silica gel in EtOAc/PE (3:1) afforded **3** (78 mg, 90%) as a colorless syrupy mixture of anomers in 1:0.2 (α/β) ratio according to NMR analysis in $CDCl_3$, R_f 0.35

(EtOAc). NMR data for the α-anomer: ^{1}H NMR (MeOH-d_4, 400 MHz, ^{1}H{^{19}F}, H-H COSY): δ 5.30 (dt, 1H, J = 12.5, 9.5 Hz, H-4), 5.06 (dd, 1H, J = 5.0, 1.8 Hz, H-1), 5.02 (ddd, 1H, J = 49.2, 9.5, 5.0 Hz, H-3), 4.57 (ddd, 1H, J = 5.4, 5.0, 1.8 Hz, H-2), 4.28 (dd, 1H, J = 11.4, 5.4 Hz, H-6), 4.16−4.08 (m, 2H, H-5, H-6′), 2.10, 2.05, 2.03 (3×s, 3×3H, *Me*). ^{13}C{^{1}H} NMR (MeOH-d_4, 101 MHz, proton-coupled-HSQC, HSQC, HMBC): δ 173.9 (CO_{NHAc}), 172.5 ($CO_{O\text{-}6}$), 171.7 ($CO_{O\text{-}4}$), 94.9 (d, $^3J_{(C\text{-}F)}$ = 7.4 Hz, $^1J_{(C\text{-}H)}$ = 175.4 Hz, C-1), 89.3 (d, $^1J_{(C\text{-}F)}$ = 187.8 Hz, C-3), 69.1 (d, $^2J_{(C\text{-}F)}$ = 19.5 Hz, C-4), 68.8 (d, $^3J_{(C\text{-}F)}$ = 6.6 Hz, C-5), 64.3 (d, $^4J_{(C\text{-}F)}$ = 2.2 Hz, C-6), 53.2 (d, $^2J_{(C\text{-}F)}$ = 15.4 Hz, C-2), 22.4, 20.71, 20.67 (3×*Me*). ^{19}F NMR (MeOH-d_4, 376 MHz): δ −205.90 (m). Resolved signals for the β-anomer: ^{1}H NMR (MeOH-d_4, 400 MHz, ^{1}H{^{19}F}, H-H COSY): δ 5.20 (dt, 1H, J = 11.7, 9.6 Hz, H-4), 4.93 (d, 1H, J = 1.8 Hz, H-1. ^{13}C{^{1}H} NMR (MeOH-d_4, 101 MHz, proton-coupled-HSQC, HSQC, HMBC): δ 94.2 (d, $^3J_{(C\text{-}F)}$ = 10.6 Hz, $^1J_{(C\text{-}H)}$ = 166.3 Hz, C-1). ^{19}F NMR (MeOH-d_4, 376 MHz): δ −200.71 (m, $^2J_{(H\text{-}F)}$ = 49.2 Hz is observed). HRMS ESI [M + Na]$^+$ calcd for $C_{12}H_{18}FNO_7Na$ 330.0960; found 330.0969.

2-Acetamido-4,6-di-*O*-propionyl-2,3-dideoxy-3-fluoro-D-mannopyranose (4)

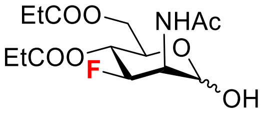

Compound **4** was prepared from compound **27** (43 mg, 0.13 mmol) according to the general procedure for azide to acetamide conversion using pyridine (0.1 mL, 1.2 mmol) and thioacetic acid (0.1 mL, 1.4 mmol). Chromatography on silica gel in EtOAc/PE (3:1) afforded **4** (39 mg, 86%) as a colorless syrup of the α-anomer with about 10% of the β-anomer according to NMR analysis in MeOH-d_4, R_f 0.35 (EtOAc). ^{1}H NMR (MeOH-d_4, 400 MHz, ^{1}H{^{19}F}, H-H COSY): δ 5.31 (ddd, 1H, J = 12.4, 9.9, 9.5 Hz, H-4), 5.06 (dd, 1H, J = 4.9, 1.8 Hz, H-1), 4.96 (ddd, 1H, J = 49.2, 9.5, 5.2 Hz, H-3), 4.56 (ddd, 1H, J = 5.4, 5.2, 1.8 Hz, H-2), 4.28 (dd, 1H, J = 11.2, 5.8 Hz, H-6), 4.16−4.09 (m, 2H, H-5, H-6′), 2.44−2.33 (m, 4H, COCH_2), 2.03 (s, 3H, Me_{Ac}), 1.16−1.10 (m, 6H, CH$_2$CH_3). ^{13}C{^{1}H} NMR (MeOH-d_4, 101 MHz, proton-coupled-HSQC, HSQC, HMBC, HSQC TOCSY): δ 175.9, 175.1, 173.9 (3×*C*O), 94.9 (d, $^3J_{(C\text{-}F)}$ = 7.4 Hz, $^1J_{(C\text{-}H)}$ = 176.0 Hz, C-1), 89.4 (d, $^1J_{(C\text{-}F)}$ = 187.8 Hz, C-3), 69.0 (d, $^2J_{(C\text{-}F)}$ = 19.5 Hz, C-4), 68.9 (d, $^3J_{(C\text{-}F)}$ = 6.7 Hz, C-5), 64.2 (d, $^4J_{(C\text{-}F)}$ = 2.2 Hz, C-6), 53.2 (d, $^2J_{(C\text{-}F)}$ = 15.2 Hz, C-2), 28.3, 28.1 (2×COCH_2), 22.4 (Me_{Ac}), 9.34, 9.31 (2×CH$_2$CH_3). ^{19}F NMR (MeOH-d_4, 376 MHz): δ −205.91 (m, $^2J_{(H\text{-}F)}$ = 49.2 Hz is observed). HRMS ESI [M + Na]$^+$ calcd for $C_{14}H_{22}FNO_7Na$ 358.1273; found 358.1280.

1,3,6-Tri-*O*-acetyl-2-azido-2,4-dideoxy-4-fluoro-D-mannopyranose (5)

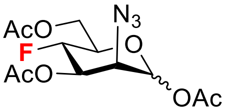

Compound **5** was prepared according to the general procedure for acid-catalyzed acetolysis starting from **31** (134 mg, 0.43 mmol) and 1 ml of Ac_2O/H_2SO_4 solution. Column chromatography on silica gel in EtOAc/PE (2:5) provided **5** (119 mg, 83%) as a colorless syrupy mixture of anomers in 1:0.1 (α/β) ratio, R_f 0.15 (EtOAc/PE 1:3) according to NMR analysis in $CDCl_3$. NMR data for the α-anomer: ^{1}H NMR ($CDCl_3$, 400 MHz, ^{1}H{^{19}F}, H-H COSY): δ 6.08 (dd, 1H, J = 2.9, 1.9 Hz, H-1), 5.45 (ddd, 1H, J = 13.3, 9.5, 4.0 Hz, H-3), 4.81 (ddd, 1H, J = 51.0, 9.7, 9.5 Hz, H-4), 4.39 (ddd, 1H, J = 12.4, 2.4, 1.9 Hz, H-6), 4.25 (dd, 1H, J = 12.4, 4.6 Hz, H-6′), 4.10−4.05 (m, 2H, H-2, H-5), 2.20, 2.18, 2.10 (3×s, 3×3H, *Me*). ^{13}C{^{1}H} NMR ($CDCl_3$, 101 MHz, proton-coupled-HSQC, HSQC, HMBC): δ 170.7, 170.1, 168.3 (3×*C*O), 91.4 (d, $^4J_{(C\text{-}F)}$ = 1.4 Hz, $^1J_{(C\text{-}H)}$ = 180.5 Hz, C-1), 84.6 (d, $^1J_{(C\text{-}F)}$ = 183.8 Hz, C-4), 70.6 (d, $^2J_{(C\text{-}F)}$ = 19.1 Hz, C-3), 70.4 (d, $^2J_{(C\text{-}F)}$ = 24.1 Hz, C-5), 62.0 (C-6), 61.0 (d, $^3J_{(C\text{-}F)}$ = 7.5 Hz, C-2), 21.0, 20.9, 20.7 (3×*Me*). ^{19}F NMR ($CDCl_3$, 376 MHz): δ −205.92 (ddddd, $^2J_{(H\text{-}F)}$ = 51.0 Hz, $^3J_{(H\text{-}F)}$ = 13.3, 4.7 Hz, $^4J_{(H\text{-}F)}$ = 2.9, 1.9 Hz). Resolved signals for the β-anomer: ^{1}H NMR ($CDCl_3$, 400 MHz, ^{1}H{^{19}F}, H-H COSY): δ 5.86 (d, 1H, J = 1.4 Hz, H-1), 5.15 (ddd, 1H, J = 13.2, 9.3, 3.9 Hz, H-3), 4.70 (ddd, 1H, J = 51.0, 9.7, 9.3 Hz, H-4), 4.41 (ddd, 1H, J = 12.3, 2.4, 2.2 Hz, H-6), 4.19 (ddd, 1H, J = 3.9, 2.0, 1.4 Hz, H-2), 3.80 (dddd, 1H, J = 9.7, 5.1, 4.3, 2.4 Hz, H-5), 2.19, 2.11 (2×s, 2×3H, *Me*). ^{13}C{^{1}H} NMR ($CDCl_3$, 101 MHz, proton-coupled-HSQC, HSQC, HMBC): δ 91.3 (d, $^4J_{(C\text{-}F)}$ = 1.3 Hz, $^1J_{(C\text{-}H)}$ = 166.7 Hz, C-1), 84.6 (d, $^1J_{(C\text{-}F)}$ = 183.7 Hz, C-4), 71.9 (d, $^2J_{(C\text{-}F)}$ = 19.3 Hz, C-3), 62.1 (C-6), 61.5 (d, $^3J_{(C\text{-}F)}$ = 7.7 Hz, C-2), 20.83, 20.77, 20.68 (3×*Me*). ^{19}F NMR ($CDCl_3$, 376 MHz): δ −205.52 (m, $^2J_{(H\text{-}F)}$ = 51.0 Hz is observed). HRMS ESI $[M + Na]^+$ calcd for $C_{12}H_{16}FN_3O_7Na$ 356.0865; found 356.0865.

2-Acetamido-3,6-di-*O*-acetyl-2,4-dideoxy-4-fluoro-D-mannopyranose (6)

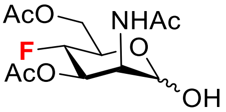

Compound **32** (65 mg, 0.19 mmol) was dissolved in dry DMF under an argon atmosphere and cooled to 0 °C. Hydrazine acetate (20 mg, 0.22 mmol) was added, and the reaction mixture was stirred for 3 hours at 0 °C. Reaction mixture was diluted with dichloromethane, washed with water, saturated solution of $NaHCO_3$ and brine, dried and concentrated. Column chromatography on silica gel in EtOAc/PE (3:1) provided product **6** (35 mg, 61%) as a yellowish syrupy mixture of anomers in 1:0.1 (α/β) ratio according to NMR analysis in MeOH-d_4, R_f 0.20 (EtOAc). NMR data for the α-anomer: ^{1}H NMR (MeOH-d_4, 400 MHz, ^{1}H{^{19}F}, H-H COSY): δ 5.40 (ddd, 1H, J = 14.6, 9.6, 4.8 Hz, H-3), 5.00 (dd, 1H, J = 3.3, 1.6 Hz, H-1), 4.62 (ddd, 1H, J = 51.2, 9.6, 9.4 Hz, H-4), 4.51 (ddd, 1H, J = 4.8, 2.5, 1.6 Hz, H-2), 4.37–

4.27 (m, 3H, H-5, H-6, H-6′), 2.08, 2.02, 2.00 (3×s, 3×3H, Me_{Ac}). $^{13}C\{^1H\}$ NMR (MeOH-d_4, 101 MHz, proton-coupled-HSQC, HSQC, HMBC): δ 173.8, 172.6, 171.7 (3×*C*O), 94.6 (d, $^4J_{(C\text{-}F)}$ = 1.2 Hz, $^1J_{(C\text{-}H)}$ = 173.3 Hz, C-1), 87.2 (d, $^1J_{(C\text{-}F)}$ = 181.7 Hz, C-4), 71.1 (d, $^2J_{(C\text{-}F)}$ = 18.3 Hz, C-3), 68.7 (d, $^2J_{(C\text{-}F)}$ = 23.1 Hz, C-5), 64.3 (C-6), 53.0 (d, $^3J_{(C\text{-}F)}$ = 7.7 Hz, C-2), 22.4, 20.8, 20.6 (3×*Me*). ^{19}F NMR (MeOH-d_4, 376 MHz): δ –205.54 (m,). NMR data for the β-anomer: ^1H NMR (MeOH-d_4, 400 MHz, $^1H\{^{19}F\}$, H-H COSY): δ 5.15 (ddd, 1H, *J* = 14.4, 9.5, 4.5 Hz, H-3), 5.05 (d, 1H, *J* = 1.7 Hz, H-1), 4.63 (ddd, 1H, *J* = 4.5, 2.3, 1.7 Hz, H-2), 4.54 (ddd, 1H, *J* = 51.2, 9.6, 9.5 Hz, H-4), 4.39–4.26 (m, 2H, H-6, H-6′), 3.86 (dddd, 1H, *J* = 9.6, 6.8, 4.0, 2.6 Hz, H-5), 2.08, 2.04, 2.01 (3×s, 3×3H, Me_{Ac}). $^{13}C\{^1H\}$ NMR (MeOH-d_4, 101 MHz, proton-coupled-HSQC, HSQC, HMBC): δ 174.7, 172.5, 171.7 (3×*C*O), 94.4 (d, $^4J_{(C\text{-}F)}$ = 1.4 Hz, $^1J_{(C\text{-}H)}$ = 162.2 Hz, C-1), 87.1 (d, $^1J_{(C\text{-}F)}$ = 183.0 Hz, C-4), 73.5 (d, $^2J_{(C\text{-}F)}$ = 18.1 Hz, C-3), 73.1 (d, $^2J_{(C\text{-}F)}$ = 23.5 Hz, C-5), 64.2 (C-6), 53.2 (d, $^3J_{(C\text{-}F)}$ = 8.0 Hz, C-2), 22.5, 20.7, 20.6 (3×*Me*). ^{19}F NMR (MeOH-d_4, 376 MHz): δ −209.53 (m). HRMS ESI $[M + Na]^+$ calcd for $C_{12}H_{18}FNO_7Na$ 330.0960; found 330.0960.

2-Acetamido-3,4-di-*O*-acetyl-2,6-dideoxy-6-fluoro-D-mannopyranose (7)

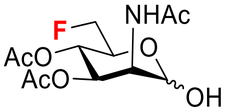

Compound **37** (72 mg, 0.21 mmol) was dissolved in dry DMF under an argon atmosphere and cooled to 0 °C. Hydrazine acetate (20 mg, 0.22 mmol) was added, and the reaction mixture was stirred for 3 hours at 0 °C. Reaction mixture was diluted with dichloromethane, washed with water, saturated solution of $NaHCO_3$ and brine, dried and concentrated. Column chromatography on silica gel in EtOAc/PE (3:1) provided **7** (36 mg, 57%) as a yellowish syrupy mixture of anomers in 1:0.1 (α/β) ratio according to NMR analysis in MeOH-d_4, R_f 0.35 (EtOAc). NMR data for the α-anomer: ^1H NMR (MeOH-d_4, 400 MHz, $^1H\{^{19}F\}$, H-H COSY): δ 5.34 (dd, 1H, *J* = 10.1, 4.5 Hz, H-3), 5.22 (dd, 1H, *J* = 10.1, 10.2 Hz, H-4), 5.04 (d, 1H, *J* = 1.8 Hz, H-1), 4.51 (ddd, 1H, *J* = 47.8, 10.3, 5.0 Hz, H-6), 4.50 (dd, 1H, *J* = 4.5, 1.8 Hz, H-2), 4.45 (ddd, 1H, *J* = 47.5, 10.3, 2.5 Hz, H-6′), 4.21 (dddd, 1H, *J* = 23.0, 10.2, 5.0, 2.5 Hz, H-5), 2.06, 2.02, 1.95 (3×s, 3×3H, *Me*). $^{13}C\{^1H\}$ NMR (MeOH-d_4, 101 MHz, proton-coupled-HSQC, HSQC, HMBC): δ 173.9, 171.8, 171.6 (3×*C*O), 94.7 ($^1J_{(C\text{-}H)}$ = 171.5 Hz, C-1), 83.2 (d, $^1J_{(C\text{-}F)}$ = 172.1 Hz, C-6), 71.0 (d, $^4J_{(C\text{-}F)}$ = 1.4 Hz, C-3), 69.9 (d, $^2J_{(C\text{-}F)}$ = 19.1 Hz, C-5), 67.1 (d, $^3J_{(C\text{-}F)}$ = 7.3 Hz, C-4), 52.4 (C-2), 22.4, 20.8, 20.6 (3×*Me*). ^{19}F NMR (MeOH-d_4, 376 MHz, $^{19}F\{^1H\}$): δ –234.10 (ddd, $^2J_{(F\text{-}H)}$ = 47.8, 47.5 Hz, $^3J_{(F\text{-}H)}$ = 23.0 Hz). Resolved signals for the β-anomer: ^1H NMR (MeOH-d_4, 400 MHz, $^1H\{^{19}F\}$, H-H COSY): δ 5.13 (dd, 1H, *J* = 10.1, 9.5 Hz, H-4), 5.08 (dd, 1H, *J* = 10.1, 4.0 Hz, H-3), 4.61 (dd, 1H, *J* = 4.0, 1.8 Hz, H-2), 3.80 (dddd, 1H, *J* = 20.8, 9.5, 5.2, 2.8 Hz, H-5), 2.05 (2×s, 2×3H, *Me*), 1.95 (s, 3H, *Me*). $^{13}C\{^1H\}$ NMR (MeOH-d_4, 101 MHz, proton-coupled-HSQC, HSQC, HMBC): δ 174.8, 171.6 (2×*C*O), 94.4 ($^1J_{(C\text{-}H)}$ = 162.6 Hz, C-1), 83.0 (d, $^1J_{(C\text{-}F)}$ = 163.7 Hz, C-6), 74.4 (d, $^2J_{(C\text{-}F)}$ = 19.4 Hz, C-5),

73.6 (d, $^4J_{(C-F)}$ = 1.4 Hz, C-3), 66.8 (d, $^3J_{(C-F)}$ = 7.1 Hz, C-4), 52.8 (C-2), 22.5, 20.7, 20.6 (3×*Me*). ^{19}F NMR (MeOH-d_4, 376 MHz): δ −233.54 (td, $^2J_{(H-F)}$ = 47.4 Hz, $^3J_{(H-F)}$ = 20.8 Hz). HRMS ESI [M + Na]$^+$ = 330.0960, calcd for $C_{12}H_{18}FNO_7Na$; found 330.0961.

2-Acetamido-6-*O*-acetyl-2,3,4-trideoxy-3,4-difluoro-D-mannopyranose (8)

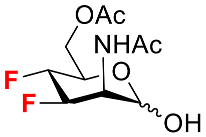

Compound **8** was prepared from compound **59** (54 mg, 0.21 mmol) according to the general procedure for azide to acetamide conversion using pyridine (0.2 mL, 2.5 mmol) and thioacetic acid (0.2 mL, 2.8 mmol). Chromatography on silica gel in EtOAc afforded **8** (38 mg, 66%) as a colorless syrupy mixture of anomers in 1:0.1 (α/β) ratio according to NMR analysis in MeOH-d_4, R_f 0.30 (EtOAc). NMR data for the α-anomer: ^{1}H NMR (MeOH-d_4, 400 MHz, ^{1}H{^{19}F}, H-H COSY): δ 5.05 (dddd, 1H, J = 50.1, 16.0, 8.7, 5.4 Hz, H-3), 5.04 (ddd, 1H, J = 4.9, 3.1, 1.7 Hz, H-1), 4.75 (dddd, 1H, J = 50.9, 13.5, 9.3, 8.7 Hz, H-4), 4.57 (td, 1H, J = 5.4, 1.7 Hz, H-2), 4.37 (ddd, 1H, J = 11.9, 2.6, 2.1 Hz, H-6), 4.29 (ddd, 1H, J = 11.9, 6.3, 1.2 Hz, H-6′), 4.21 (dddd, 1H, J = 9.3, 6.3, 5.9, 2.6 Hz, H-5), 2.07, 2.01 (2×s, 2×3H, *Me*). ^{13}C{^{1}H} NMR (MeOH-d_4, 101 MHz, proton-coupled-HSQC, HSQC, HMBC): δ 173.8 (CO_{NHAc}), 172.5 (CO_{OAc}), 94.8 (dd, $^3J_{(C-F)}$ = 7.2 Hz, $^4J_{(C-F)}$ = 1.1 Hz, $^1J_{(H-C)}$ = 175.1 Hz, C-1), 89.2 (dd, $^1J_{(C-F)}$ = 187.2 Hz, $^2J_{(C-F)}$ = 18.8 Hz, C-3), 88.1 (dd, $^1J_{(C-F)}$ = 181.1 Hz, $^2J_{(C-F)}$ = 19.4 Hz, C-4), 68.1 (dd, $^2J_{(C-F)}$ = 23.0 Hz, $^3J_{(C-F)}$ = 6.9 Hz, C-5), 64.1 (d, $^3J_{(C-F)}$ = 2.0 Hz, C-6), 53.8 (dd, $^2J_{(C-F)}$ = 15.4 Hz, $^3J_{(C-F)}$ = 8.0 Hz, C-2), 22.4, 20.6 (2×*Me*). ^{19}F NMR (MeOH-d_4, 376 MHz): δ –205.76 (dddd, $^2J_{(H-F)}$ = 50.9 Hz, $^3J_{(H-F)}$ = 16.0, 5.9 Hz, $^3J_{(F-F)}$ = 13.4 Hz, F-4), –206.81 (ddddd, $^2J_{(H-F)}$ = 49.8 Hz, $^3J_{(H-F)}$ = 13.5, 5.4 Hz, $^3J_{(F-F)}$ = 13.4 Hz, $^4J_{(H-F)}$ = 4.9 Hz, F-3). Resolved signals for the β-anomer: ^{1}H{^{19}F} NMR (MeOH-d_4, 400 MHz, H-H COSY): δ 4.95 (d, 1H, J = 1.7 Hz, H-1), 4.62 (dd, 1H, J = 9.7, 8.5 Hz, H-4), 4.38 (ddd, 1H, J = 12.0, 2.7 Hz H-6), 3.75 (ddd, 1H, J = 9.7, 7.0, 2.7 Hz, H-5). ^{13}C{^{1}H} NMR (MeOH-d_4, 101 MHz, proton-coupled-HSQC, HSQC, HMBC): δ 94.2 (d, $^3J_{(C-F)}$ = 10.1 Hz, $^1J_{(H-C)}$ = 162.3 Hz, C-1), 71.9 (dd, $^2J_{(C-F)}$ = 23.2 Hz, $^3J_{(C-F)}$ = 7.9 Hz, C-5), 64.2 (d, $^3J_{(C-F)}$ = 2.7 Hz, C-6). ^{19}F NMR (MeOH-d_4, 376 MHz): δ –201.51 (dddd, $^2J_{(H-F)}$ = 48.6 Hz, $^3J_{(H-F)}$ = 12.5, 5.5 Hz, $^3J_{(F-F)}$ = 13.9 Hz, F-3), –209.37 (dddd, $^2J_{(H-F)}$ = 51.8 Hz, $^3J_{(H-F)}$ = 15.2, 5.9 Hz, $^3J_{(F-F)}$ = 13.9 Hz, F-4). HRMS ESI [M + Na]$^+$ calcd for $C_{10}H_{15}F_2NO_5Na$ 290.0811; found 290.0811.

2-Acetamido-4-*O*-acetyl-2,3,6-trideoxy-3,6-difluoro-D-mannopyranose (9)

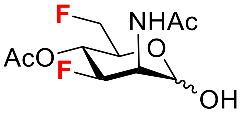

Compound **9** was prepared from compound **29** (46 mg, 0.18 mmol) according to the general procedure for azide to acetamide conversion using pyridine (0.2 mL, 2.5 mmol) and thioacetic acid (0.2 mL, 2.8 mmol). Chromatography on silica gel in EtOAc/PE 3:1 afforded **9** (43 mg, 88%) as a colorless syrupy mixture of anomers in 1:0.1 (α/β) ratio according to NMR analysis in MeOH-d_4, R_f 0.40 (EtOAc). NMR data for the α-anomer: ^{1}H NMR (MeOH-d_4, 400 MHz, ^{1}H{^{19}F}, H-H COSY): δ 5.33 (ddd, 1H, J = 12.4, 10.1, 9.4 Hz, H-4), 5.08 (dd, 1H, J = 4.9, 1.8 Hz, H-1), 4.99 (ddd, 1H, J = 49.1, 9.4, 5.1 Hz, H-3), 4.57 (ddd, 1H, J = 5.2, 5.1, 1.8 Hz, H-2), 4.51 (ddd, 1H, J = 48.0, 10.4, 5.0 Hz, H-6), 4.46 (dddd, 1H, J = 47.2, 10.4, 2.6, 1.0 Hz, H-6′), 4.13 (dddd, 1H, J = 22.7, 10.1, 5.0, 2.6 Hz, H-5), 2.11, 2.02 (2×s, 2×3H, *Me*). ^{13}C{^{1}H} NMR (MeOH-d_4, 101 MHz, proton-coupled-HSQC, HSQC, HMBC): δ 173.9, 171.6 (2×*C*O), 95.0 (d, $^3J_{(C\text{-}F)}$ = 7.4 Hz, $^1J_{(C\text{-}H)}$ = 175.0 Hz, C-1), 89.4 (dd, $^1J_{(C\text{-}F)}$ = 187.9 Hz, $^4J_{(C\text{-}F)}$ = 1.3 Hz, C-3), 83.0 (dd, $^1J_{(C\text{-}F)}$ = 172.4 Hz, $^4J_{(C\text{-}F)}$ = 2.1 Hz, C-6), 69.5 (dd, $^2J_{(C\text{-}F)}$ = 19.4 Hz, $^3J_{(C\text{-}F)}$ = 6.7 Hz, C-5), 68.2 (dd, $^2J_{(C\text{-}F)}$ = 19.5 Hz, $^3J_{(C\text{-}F)}$ = 7.0 Hz, C-4), 53.2 (d, $^2J_{(C\text{-}F)}$ = 15.3 Hz, C-2), 22.4, 20.7 (2×*Me*). ^{19}F NMR (MeOH-d_4, 376 MHz, ^{19}F{^{1}H}): δ –205.90 (dddd, $^2J_{(H\text{-}F)}$ = 49.1 Hz, $^3J_{(H\text{-}F)}$ = 12.4, 5.2 Hz, $^5J_{(F\text{-}F)}$ = 2.0 Hz, F-3), –233.92 (dddd, $^2J_{(H\text{-}F)}$ = 48.0, 47.2 Hz, $^3J_{(H\text{-}F)}$ = 22.7 Hz, $^5J_{(F\text{-}F)}$ = 2.0 Hz, F-6). Resolved signals for the β-anomer: ^{1}H NMR (MeOH-d_4, 400 MHz, ^{1}H{^{19}F}, H-H COSY): δ 5.21 (ddd, 1H, J = 11.6, 10.0, 9.4 Hz, H-4), 4.95 (dd, 1H, J = 1.7, 1.6 Hz, H-1). ^{13}C{^{1}H} NMR (MeOH-d_4, 101 MHz, proton-coupled-HSQC, HSQC, HMBC): δ 94.2 (d, $^3J_{(C\text{-}F)}$ = 10.1 Hz, $^1J_{(C\text{-}H)}$ = 165.0 Hz, C-1), 91.1 (d, $^1J_{(C\text{-}F)}$ = 182.0 Hz, C-3). ^{19}F NMR (MeOH-d_4, 376 MHz, ^{19}F{^{1}H}): δ –200.63 (from ^{19}F{^{1}H}: d, $^5J_{(F\text{-}F)}$ = 2.2 Hz, F-3), –232.92 (dddd, $^2J_{(H\text{-}F)}$ = 48.0, 47.2 Hz, $^3J_{(H\text{-}F)}$ = 20.5 Hz, $^5J_{(F\text{-}F)}$ = 2.2 Hz, F-6). HRMS ESI [M + Na]$^+$ calcd for $C_{10}H_{15}F_2NO_5Na$ 290.0811; found 290.0820.

2-Acetamido-3-*O*-acetyl-2,4,6-trideoxy-4,6-difluoro-D-mannopyranose (10)

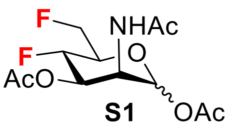

S1

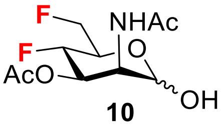

10

Compound **S1** was prepared from compound **34** (115 mg, 0.39 mmol) according to the general procedure for azide to acetamide conversion using pyridine (0.4 mL, 5.0 mmol) and thioacetic acid (0.4 mL, 5.6 mmol). Chromatography on silica gel in EtOAc/PE 3:2 afforded 1,4-di-*O*-acetyl-2-acetamido-2,4,6-trideoxy-4,6-difluoro-D-mannopyranose **S1** (104 mg, 86%) as a colorless syrupy mixture of anomers in 1:0.1 (α/β) ratio, R_f 0.30 (EtOAc/PE 3:2). ^{1}H NMR (CDCl$_3$, 400 MHz, ^{1}H{^{19}F}, H-H COSY): δ 6.01 (dd, 1H, J = 3.1, 1.8 Hz, H-1), 5.77 (d, 1H, J = 9.7 Hz, N*H*), 5.43 (ddd, 1H, J = 14.3, 9.8, 4.6 Hz, H-3), 4.75 (ddd, 1H, J = 50.6, 10.0, 9.8 Hz, H-4), 4.72–4.62 (m, 2H, H-2, H-6), 4.63 (dddd, 1H, J = 47.3, 10.7,

2.1, 1.8 Hz, H-6′), 3.98 (ddddd, 1H, J = 28.7, 10.0, 5.7, 2.1, 1.6 Hz, H-5), 2.17, 2.08, 2.04 (3×s, 3×3H, *Me*). $^{13}C\{^{1}H\}$ NMR ($CDCl_3$, 101 MHz, proton-coupled-HSQC, HSQC, HMBC): δ 170.3 ($CO_{O\text{-}NHAc}$), 170.2 ($CO_{O\text{-}3Ac}$), 168.3 ($CO_{O\text{-}1Ac}$), 91.9 (d, $^{4}J_{(C\text{-}F)}$ = 1.4 Hz, $^{1}J_{(C\text{-}H)}$ = 175.6 Hz, C-1), 83.5 (dd, $^{1}J_{(C\text{-}F)}$ = 184.3 Hz, $^{3}J_{(C\text{-}F)}$ = 7.5 Hz, C-4), 80.5 (d, $^{1}J_{(C\text{-}F)}$ = 175.4 Hz, C-6), 70.9 (dd, $^{2}J_{(C\text{-}F)}$ = 24.8, 17.8 Hz, C-5), 69.1 (d, $^{2}J_{(C\text{-}F)}$ = 18.3 Hz, C-3), 49.8 (d, $^{3}J_{(C\text{-}F)}$ = 8.0 Hz, C-2), 23.3, 21.0, 20.9 (3×*Me*). ^{19}F NMR ($CDCl_3$, 376 MHz, $^{19}F\{^{1}H\}$): δ –205.53 (dddd, $^{2}J_{(H\text{-}F)}$ = 50.6 Hz, $^{3}J_{(H\text{-}F)}$ = 14.3, 5.7 Hz, $^{5}J_{(H\text{-}F)}$ = 3.1 Hz, F-4), –238.61 (ddd, $^{2}J_{(H\text{-}F)}$ = 47.3, 46.6 Hz, $^{3}J_{(H\text{-}F)}$ = 28.7 Hz, F-6). HRMS-ESI $[M + Na]^{+}$ calcd for $C_{12}H_{17}F_2NO_6Na$ 332.0916; found 332.0920.

Compound **S1** (60 mg, 0.19 mmol) was dissolved in dry DMF under an argon atmosphere and cooled to 0 °C. Hydrazine acetate (18 mg, 0.20 mmol) was added, and the reaction mixture was stirred for 3 hours at 0 °C. Reaction mixture was diluted with dichloromethane, washed with water, saturated solution of $NaHCO_3$ and brine, dried and concentrated. Column chromatography on silica gel in EtOAc/PE 3:1 provided **10** (32 mg, 62%, 53% over two steps) as a yellowish syrupy mixture of anomers in 1:0.1 (α/β) ratio according to NMR analysis in MeOH-d_4, R_f 0.30 (EtOAc). NMR data for the α-anomer: ^{1}H NMR (MeOH-d_4, 400 MHz, $^{1}H\{^{19}F\}$, H-H COSY): δ 5.42 (ddd, 1H, J = 14.7, 9.7, 4.8 Hz, H-3), 5.04 (dd, 1H, J = 3.3, 1.5 Hz, H-1), 4.75 (ddd, 1H, J = 51.1, 9.8, 9.7 Hz, H-4), 4.67 (dddd, 1H, J = 47.6, 10.5, 4.3, 1.4 Hz, H-6), 4.60 (ddt, 1H, J = 47.9, 10.5, 1.9 Hz, H-6′), 4.52 (ddd, 1H, J = 4.8, 2.0, 1.6 Hz, H-2), 4.24 (ddddd, 1H, J = 25.9, 9.8, 5.0, 4.3, 1.9 Hz, H-5), 2.02, 2.00 (2×s, 2×3H, *Me*). $^{13}C\{^{1}H\}$ NMR (MeOH-d_4, 101 MHz, proton-coupled-HSQC, HSQC, HMBC): δ 173.8 (CO_{NHAc}), 171.7 ($CO_{O\text{-}3Ac}$), 94.7 (d, $^{4}J_{(C\text{-}F)}$ = 1.4 Hz, $^{1}J_{(C\text{-}H)}$ = 173.2 Hz, C-1), 85.8 (dd, $^{1}J_{(C\text{-}F)}$ = 181.4 Hz, $^{3}J_{(C\text{-}F)}$ = 8.2 Hz, C-4), 82.7 (d, $^{1}J_{(C\text{-}F)}$ = 172.6 Hz, C-6), 71.1 (dd, $^{2}J_{(C\text{-}F)}$ = 18.1 Hz, $^{4}J_{(C\text{-}F)}$ = 1.0 Hz, C-3), 69.7 (dd, $^{2}J_{(C\text{-}F)}$ = 23.5, 18.7 Hz, C-5), 53.1 (d, $^{3}J_{(C\text{-}F)}$ = 7.7 Hz, C-2), 22.3, 20.8 (2×*Me*). ^{19}F NMR (MeOH-d_4, 376 MHz, $^{19}F\{^{1}H\}$): δ –206.14 (dddd, $^{2}J_{(H\text{-}F)}$ = 51.1 Hz, $^{3}J_{(H\text{-}F)}$ = 14.7, 5.0 Hz, $^{5}J_{(H\text{-}F)}$ = 3.3 Hz, F-4), –237.09 (ddd, $^{2}J_{(H\text{-}F)}$ = 47.9, 47.6 Hz, $^{3}J_{(H\text{-}F)}$ = 25.9 Hz, F-6). Resolved signals for the β-anomer: ^{1}H NMR (MeOH-d_4, 400 MHz, $^{1}H\{^{19}F\}$, H-H COSY): δ 5.17 (ddd, 1H, J = 14.4, 9.6, 4.6 Hz, H-3), 5.08 (d, 1H, J = 1.8 Hz, H-1), 2.03, 2.01 (2×s, 2×3H, *Me*). $^{13}C\{^{1}H\}$ NMR (MeOH-d_4, 101 MHz, proton-coupled-HSQC, HSQC, HMBC): δ 94.5 (d, $^{4}J_{(C\text{-}F)}$ = 1.2 Hz, $^{1}J_{(C\text{-}H)}$ = 161.3 Hz, C-1), 73.8 (d, $^{2}J_{(C\text{-}F)}$ = 20.6 Hz, C-3), 53.3 (d, $^{3}J_{(C\text{-}F)}$ = 7.5 Hz, C-2). ^{19}F NMR (MeOH-d_4, 376 MHz, $^{19}F\{^{1}H\}$): δ –210.08 (dd, $^{2}J_{(H\text{-}F)}$ = 51.6 Hz, $^{3}J_{(H\text{-}F)}$ = 14.8 Hz, F-4), –236.23 (td, $^{2}J_{(H\text{-}F)}$ = 47.5 Hz, $^{3}J_{(H\text{-}F)}$ = 23.2 Hz, F-6). HRMS-ESI $[M + Na]^{+}$ calcd for $C_{10}H_{15}F_2NO_5Na$ 290.0811; found 290.0815.

2-Acetamido-2,3,4,6-tetradeoxy-3,4,6-trifluoro-D-mannopyranose (11)

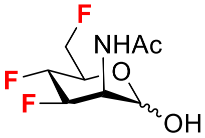

Compound **11** was prepared from compound **61** (59 mg, 0.28 mmol) according to the general procedure for azide to acetamide conversion using pyridine (0.3 mL, 3.7 mmol) and thioacetic acid (0.3 mL, 4.2 mmol). Chromatography on silica gel in DCM/MeOH 25:1 afforded **11** (49 mg, 77%) as a colorless syrupy mixture of anomers in 1:0.1 (α/β) ratio according to NMR analysis in MeOH-d_4, R_f 0.20 (DCM/MeOH 25:1). NMR data for the α-anomer: ^{1}H NMR (MeOH-d_4, 400 MHz, ^{1}H{^{19}F}, H-H COSY): δ 5.08 (dddd, 1H, J = 50.1, 16.4, 9.1, 5.4 Hz, H-3), 5.07 (dd, 1H, J = 3.8, 1.9 Hz, H-1), 4.85 (dddd, 1H, J = 52.9, 14.0, 9.9, 9.1 Hz, H-4), 4.66 (dddd, 1H, J = 47.6, 10.4, 4.3, 1.5 Hz, H-6), 4.61 (dddd, 1H, J = 47.6, 10.4, 1.9, 1.7 Hz, H-6′), 4.58—4.56 (m, 1H, H-2), 4.15 (ddddd, 1H, J = 25.5, 9.9, 4.7, 4.3, 1.9 Hz, H-5), 2.00 (s, 3H, *Me*). ^{13}C{^{1}H} NMR (MeOH-d_4, 101 MHz, proton-coupled-HSQC, HSQC, HMBC): δ 173.9 (*C*O), 94.9 (dd, $^3J_{(C\text{-}F)}$ = 7.0 Hz, $^4J_{(C\text{-}F)}$ = 1.4 Hz, $^1J_{(C\text{-}H)}$ = 176.1 Hz, C-1), 89.3 (ddd, $^1J_{(C\text{-}F)}$ = 187.2 Hz, $^2J_{(C\text{-}F)}$ = 18.4 Hz, $^4J_{(C\text{-}F)}$ = 0.9 Hz, C-3), 86.7 (ddd, $^1J_{(C\text{-}F)}$ = 180.3 Hz, $^2J_{(C\text{-}F)}$ = 18.9 Hz, $^3J_{(C\text{-}F)}$ = 8.1 Hz, C-4), 82.6 (dd, $^1J_{(C\text{-}F)}$ = 172.9 Hz, $^3J_{(C\text{-}F)}$ = 1.8 Hz, C-6), 69.2 (ddd, $^2J_{(C\text{-}F)}$ = 23.3, 18.8 Hz, $^3J_{(C\text{-}F)}$ = 7.1 Hz, C-5), 53.9 (dd, $^2J_{(C\text{-}F)}$ = 15.5 Hz, $^3J_{(C\text{-}F)}$ = 8.1 Hz, C-2), 22.3 (*Me*). ^{19}F NMR (MeOH-d_4, 376 MHz, ^{19}F{^{1}H}): δ –206.34 (dddd, $^2J_{(H\text{-}F)}$ = 52.9 Hz, $^3J_{(F\text{-}F)}$ = 13.3 Hz, $^3J_{(H\text{-}F)}$ = 16.4, 4.7 Hz, F-4), –206.82 (dddddd, $^2J_{(H\text{-}F)}$ = 50.1 Hz, $^3J_{(H\text{-}F)}$ = 14.0, 4.9 Hz, $^3J_{(F\text{-}F)}$ = 13.3 Hz, $^4J_{(H\text{-}F)}$ = 3.8 Hz, $^5J_{(F\text{-}F)}$ = 1.8 Hz, F-3), –237.11 (tdd, $^2J_{(H\text{-}F)}$ = 47.6 Hz, $^3J_{(H\text{-}F)}$ = 25.5 Hz, $^5J_{(F\text{-}F)}$ = 1.8 Hz, F-6). Resolved signals for β-anomer: ^{13}C{^{1}H} NMR (MeOH-d_4, 101 MHz, proton-coupled-HSQC, HSQC, HMBC): δ 94.3 (dd, ^3J = 9.4 Hz, ^4J = 1.6 Hz, $^1J_{(H\text{-}C)}$ = 162.5 Hz, C-1), 22.6 (*Me*). ^{19}F NMR (MeOH-d_4, 376 MHz, ^{19}F{^{1}H}): δ –201.51 (dd, $^3J_{(F\text{-}F)}$ = 13.8 Hz, $^5J_{(F\text{-}F)}$ = 1.8 Hz, F-3), –209.84 (dd, $^3J_{(F\text{-}F)}$ = 13.8 Hz, F-4), –235.79 (tdd, $^2J_{(H\text{-}F)}$ = 47.4 Hz, $^3J_{(H\text{-}F)}$ = 22.8 Hz, $^5J_{(F\text{-}F)}$ = 1.8 Hz, F-6). HRMS ESI [M + Na]$^+$ calcd for $C_8H_{12}F_3NO_3Na$ 250.0662; found 250.0659.

Methyl 2-azido-4,6-*O*-benzylidene-2-deoxy-α-D-altropyranoside (12)

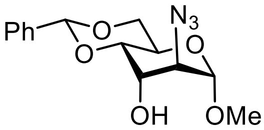

Compound **12** was prepared according to the known procedure.[2]

Methyl 2-azido-4,6-*O*-benzylidene-2,4-dideoxy-4-fluoro-α-D-altropyranoside (17)
Methyl 2-azido-4,6-*O*-benzylidene-2,4-dideoxy-4-fluoro-α-D-mannopyranoside (18)
Methyl 2-azido-4,6-*O*-benzylidene-2,3-dideoxy-D-*threo*-hex-3-enopyranoside (19)

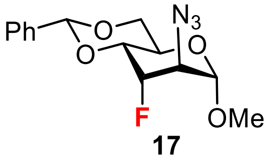
17
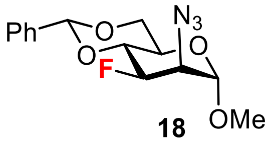
18
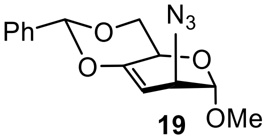
19

A: DAST (120 µL, 0.91 mmol) was added to a solution of **12** (50 mg, 0.16 mmol) in dry DCM (2 mL) at −78 °C under argon atmosphere and the reaction was allowed to warm to rt during 5 hours and then stirred at rt overnight until TLC indicated complete consumption of the starting material and a presence of one less polar product. The reaction mixture was diluted with DCM, quenched by an addition of saturated solution of $NaHCO_3$ and washed with water. The aqueous phase was extracted with DCM (3×). The organic extracts were combined, dried, and concentrated. Column chromatography on silica gel in EtOAc/PE 1:7 afforded **17** (27 mg, 54%, contaminated with traces of the inseparable epimer **18**, **17**/**18** ≈ 15:1) as a colorless syrup, R_f 0.20 (EtOAc/PE 1:7). ^{1}H NMR ($CDCl_3$, 400 MHz, H-H COSY): δ 7.52–7.49 (m, 2H, CH_{Ph}), 7.41–7.37 (m, 3H, CH_{Ph}), 5.61 (s, 1H, C*H*Ph), 4.88 (ddd, 1H, J = 49.6, 3.2, 2.3 Hz, H-3), 4.74 (d, 1H, J = 0.9 Hz, H-1), 4.35 (dd, 1H, J = 9.8, 5.3 Hz, H-6), 4.29 (ddd, 1H, J = 9.5, 5.3, 1.4 Hz, H-5), 4.02 (ddd, 1H, J = 8.3, 3.2, 0.9 Hz, H-2), 3.88 (ddd, 1H, J = 29.6, 9.5, 2.3 Hz, H-4), 3.78 (dd, 1H, J = 9.8, 1.4 Hz, H-6′), 3.45 (s, 3H, *Me*). ^{13}C{^{1}H} NMR ($CDCl_3$, 101 MHz, proton-coupled-HSQC, HSQC, HMBC): δ 137.1 (C_q), 129.4 (CH_{Ph}), 128.5, 126.4 (2×2CH_{Ph}), 102.7 (*C*HPh), 99.0 ($^{1}J_{(C-H)}$ = 173.4 Hz, C-1), 85.8 (d, $^{1}J_{(C-F)}$ = 188.8 Hz, C-3), 74.6 (d, $^{2}J_{(C-F)}$ = 16.8 Hz, C-4), 69.1 (C-6), 60.7 (d, $^{2}J_{(C-F)}$ = 26.1 Hz, C-2), 58.3 (d, $^{3}J_{(C-F)}$ = 3.0 Hz, C-5), 55.9 (*Me*). ^{19}F NMR ($CDCl_3$, 376 MHz): δ −202.32 (ddd, $^{2}J_{(H-F)}$ = 49.6 Hz, $^{3}J_{(H-F)}$ = 29.6, 8.3 Hz). HRMS-ESI [M + Na]$^+$ calcd for $C_{14}H_{16}FN_3O_4Na$, 332.1017; found 332.1021.

B: DAST (120 µL, 0.91 mmol) and triethylamine trihydrofluoride (50 µL, 0.31 mmol) were added to a solution of **12** (50 mg, 0.16 mmol) in dry DCM (2 mL) at −78 °C under argon atmosphere and the reaction was allowed to warm to rt during 5 hours and then stirred at rt overnight until TLC indicated complete consumption of the starting material and a presence of one less polar product. The reaction mixture was diluted with DCM, quenched by an addition of saturated solution of $NaHCO_3$ and washed with water. The aqueous phase was extracted with DCM (3×). The organic extracts were combined, dried, and concentrated. Column chromatography on silica gel in EtOAc/PE 1:7 afforded **17** (32 mg, 64%, containing traces of the epimeric **18**, **17**/**18** ≈ 10:1) as a colorless syrup.

C: DAST (200 µL, 1.51 mmol) and was added to a solution of **12** (100 mg, 0.32 mmol) in dry toluene (1.5 mL) at −10 °C under argon atmosphere and the reaction was stirred at −10 °C for 30 min. The reaction was then slowly heated to 70 °C when TLC indicated complete consumption of the starting material and a presence of two less polar products. The reaction mixture was diluted with DCM,

quenched by an addition of MeOH and saturated solution of $NaHCO_3$ and washed with water. The aqueous phase was extracted with DCM (3×). The organic extracts were combined, dried, and concentrated. Column chromatography in EtOAc/PE 1:10 first afforded **19**[3] (37 mg, 39%) as a yellowish syrup followed by **18** (18 mg, 18%) as a yellow crystalline solid.

Data for **18**: R_f 0.10 (EtOAc/PE 1:10). ^{1}H NMR ($CDCl_3$, 400 MHz, H-H COSY): δ 7.52–7.49 (m, 2H, CH_{Ph}), 7.41–7.35 (m, 3H, CH_{Ph}), 5.62 (s, 1H, C*H*Ph), 5.07 (ddd, 1H, J = 50.0, 9.7, 4.2 Hz, H-3), 4.74 (dd, 1H, J = 4.2, 1.6 Hz, H-1), 4.29 (ddd, 1H, J = 9.9, 4.5, 2.5 Hz, H-6), 4.24–4.16 (m, 2H, H-2, H-4), 3.87 (dd, 1H, J = 10.3, 9.9 Hz, H-6′), 3.79 (tdd, 1H, J = 10.3, 4.5, 1.3 Hz, H-5), 3.39 (s, 3H, *Me*). ^{13}C{^{1}H} NMR ($CDCl_3$, 101 MHz, proton-coupled-HSQC, HSQC, HMBC): δ 137.0 (C_q), 129.4 (CH_{Ph}), 128.5, 126.3 (2×2CH_{Ph}), 102.1 (*C*HPh), 100.5 (d, $^3J_{(C\text{-}F)}$ = 6.7 Hz, $^1J_{(C\text{-}H)}$ = 174.2 Hz, C-1), 88.7 (d, $^1J_{(C\text{-}F)}$ = 191.1 Hz, C-3), 77.1 (d, $^2J_{(C\text{-}F)}$ = 17.6 Hz, C-4), 68.7 (d, $^4J_{(C\text{-}F)}$ = 1.7 Hz, C-6), 63.2 (d, $^3J_{(C\text{-}F)}$ = 7.4 Hz, C-5), 62.5 (d, $^2J_{(C\text{-}F)}$ = 15.5 Hz, C-2), 55.4 (*Me*). ^{19}F NMR ($CDCl_3$, 376 MHz): δ −202.50 (m, $^2J_{(H\text{-}F)}$ = 50.0 Hz is observed). HRMS-APCI $[M + H]^+$ calcd for $C_{14}H_{17}FN_3O_4$ 310.1198; found 310.1201

Data for **19**: R_f 0.15 (EtOAc/PE 1:10). ^{1}H NMR ($CDCl_3$, 400 MHz, H-H COSY): δ 7.54–7.52 (m, 2H, CH_{Ph}), 7.42–7.37 (m, 3H, CH_{Ph}), 5.60 (s, 1H, C*H*Ph), 5.36 (ddd, 1H, J = 5.5, 1.8, 0.8 Hz, H-3), 4.84 (dd, 1H, J = 1.8, 0.8 Hz, H-1), 4.47 (dd, 1H, J = 10.3, 6.4 Hz, H-6), 4.39 (ddt, 1H, J = 10.3, 6.4, 1.8 Hz, H-5), 3.80 (t, 1H, J = 10.3 Hz, H-6′), 3.60 (ddd, 1H, J = 5.5, 1.8, 0.8 Hz, H-2), 3.50 (s, 3H, *Me*). ^{13}C{^{1}H} NMR ($CDCl_3$, 101 MHz, HSQC, HMBC): δ 156.5 (C-4), 136.4 (C_q), 129.8 (CH_{Ph}), 128.6, 126.4 (2×2CH_{Ph}), 104.3 (*C*HPh), 100.2 (C-1), 97.9 (C-3), 70.3 (C-6), 60.8 (C-5), 56.7 (C-2), 56.3 (*Me*). HRMS-APCI $[M + H]^+$ calcd for $C_{14}H_{16}N_3O_4$ 290.1135; found 290.1135.

D: DAST (200 μL, 1.51 mmol) and 2,4,6-collidine (175 μL, 1.32 mmol) were added to a solution of **12** (100 mg, 0.32 mmol) in dry DCM (1.5 mL) and the reaction was heated to 80 °C for 1 hour using microwave irradiation. The reaction mixture was diluted with DCM, quenched by an addition of MeOH and saturated solution of $NaHCO_3$ and washed with water. The aqueous phase was extracted with DCM (3×). The organic extracts were combined, dried, and concentrated. Column chromatography on silica gel in EtOAc/PE 1:10 first afforded **19** (23 mg, 24%) as a yellowish syrup followed by a mixture of **17** and **18** (19 mg, 19%) in approximately 1:3 ratio (based on ^{19}F NMR) as a yellow crystalline solid.

1,6-Anhydro-4-*O*-benzyl-3-deoxy-3-fluoro-β-D-glucopyranose (20)

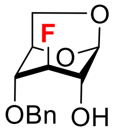

Compound **20** was prepared according to the published procedure.[4]

1,6-Anhydro-2-azido-4-*O*-benzyl-2,3-dideoxy-3-fluoro-β-D-mannopyranose (21)

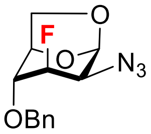

Triflic anhydride (200 μL, 1.19 mmol) was added into a solution of **20** (200 mg, 0.79 mmol) in dry DCM (4 mL) dry pyridine (1 mL) at −40 °C under argon atmosphere and the reaction was slowly allowed to warm to 0 °C when TLC indicated complete consumption of the starting material and a presence of one less polar product. The reaction mixture was poured onto ice, and the two phases were separated. The aqueous phase was extracted with DCM (3×), the organic extracts were combined, dried, and concentrated. The crude intermediate was dissolved in dry DMF (2 mL), NaN_3 (0.6 g, 9.23 mmol) was added and the reaction was stirred at rt overnight. The reaction was diluted with EtOAc and washed with water. The aqueous phase was extracted with EtOAc (3×). The organic extracts were combined, washed with brine, dried, and concentrated. Column chromatography on silica gel in EtOAc/PE 1:5 afforded **21** (165 mg, 75%) as a yellowish oil, R_f 0.25 (EtOAc/PE 1:5), $[\alpha]_D^{20}$ −123 (*c* 0.86, $CHCl_3$). ^{1}H NMR ($CDCl_3$, 400 MHz, H-H COSY): δ 7.41–7.31 (m, 5H, CH_{Ph}), 5.57 (d, 1H, J = 1.7 Hz, H-1), 4.91 (ddd, 1H, J = 47.8, 4.4, 1.9 Hz, H-3), 4.69 (s, 2H, CH_2 Bn), 4.63 (ddd, 1H, J = 5.9, 1.6, 1.3 Hz, H-5), 4.10 (dt, 1H, J = 8.8, 1.3 Hz, H-6^{en}), 3.84 (ddd, 1H, J = 8.8, 5.9, 3.9 Hz, H-6^{ex}), 3.72 (ddd, 1H, J = 12.9, 1.9, 1.6 Hz, H-4), 3.15 (ddd, 1H, J = 28.2, 4.4, 1.7 Hz, H-2). ^{13}C{^{1}H} NMR ($CDCl_3$, 101 MHz, HSQC, HMBC): δ 136.9 (C_q), 128.9 (2CH_{Ph}), 128.5 (CH_{Ph}), 128.0 (2CH_{Ph}), 100.8 (C-1), 89.5 (d, $^1J_{(C-F)}$ = 184.6 Hz, C-3), 75.7 (d, $^2J_{(C-F)}$ = 25.4 Hz, C-4), 73.6 (C-5), 72.1 (CH_2 Bn), 65.1 (d, $^4J_{(C-F)}$ = 5.6 Hz, C-6), 57.1 (d, $^2J_{(C-F)}$ = 15.7 Hz, C-2). ^{19}F NMR ($CDCl_3$, 376 MHz): δ −197.64 (dddd, $^2J_{(H-F)}$ = 47.8 Hz, $^3J_{(H-F)}$ = 28.2, 12.9 Hz, $^5J_{(H-F)}$ = 3.9 Hz). HRMS-ESI $[M + Na]^+$ calcd for $C_{13}H_{14}FN_3O_3Na$ 302.0911; found 302.0923.

1,6-Anhydro-2-azido-2,3-dideoxy-3-fluoro-β-D-mannopyranose (22)

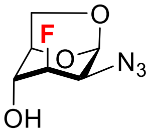

A solution of $Na_2S_2O_4$ (990 mg, 5.69 mmol) in water (6 mL) was slowly added dropwise into a two-phase solution of $NaBrO_3$ (864 mg, 5.73 mmol) in water (8 mL) and **21** (532 mg, 1.90 mmol) in EtOAc (16 mL). The reaction mixture was stirred at rt for two hours until TLC indicated an absence of the starting material and a presence of more polar product. A change of color from colorless to orange and back to colorless occurred. The reaction mixture was diluted with EtOAc, the water phase was saturated with NaCl and the phases were separated. The aqueous phase was extracted with EtOAc (3×). The organic extracts were combined, washed with saturated solution of $NaHCO_3$ and brine, dried, and concentrated. Column chromatography on silica gel in EtOAc/PE 2:3 afforded **22** (303 mg, 84%) as a

yellowish oil, R_f 0.30 (EtOAc/PE 1:1), $[\alpha]_D^{20}$ −157 (*c* 1.46, $CHCl_3$). ^{1}H NMR ($CDCl_3$, 400 MHz, H-H COSY): δ 5.57 (dd, 1H, J = 1.7, 1.8 Hz, H-1), 4.87 (ddddd, 1H, J = 47.8, 4.4, 2.2, 1.8, 0.9 Hz, H-3), 4.58 (dddd, 1H, J = 5.9, 1.8, 1.1, 0.9 Hz, H-5), 4.21 (dt, 1H, J = 7.9, 1.1 Hz, H-6^{en}), 4.01 (ddd, 1H, J = 10.9, 2.2, 1.8 Hz, H-4), 3.89 (ddd, 1H, J = 7.9, 5.9, 4.0 Hz, H-6^{ex}), 3.11 (ddd, 1H, J = 27.7, 4.4, 1.7 Hz, H-2), 2.57 (br s, 1H, O*H*). ^{13}C{^{1}H} NMR ($CDCl_3$, 101 MHz, HSQC, HMBC): δ 101.0 (C-1), 91.1 (d, $^1J_{(C\text{-}F)}$ = 185.5 Hz, C-3), 75.5 (C-5), 69.5 (d, $^2J_{(C\text{-}F)}$ = 26.4 Hz, C-4), 65.1 (d, $^4J_{(C\text{-}F)}$ = 6.0 Hz, C-6), 56.4 (d, $^2J_{(C\text{-}F)}$ = 16.0 Hz, C-2). ^{19}F NMR ($CDCl_3$, 376 MHz): δ −197.33 (ddddd, $^2J_{(H\text{-}F)}$ = 47.8 Hz, $^3J_{(H\text{-}F)}$ = 27.7, 10.9 Hz, $^5J_{(H\text{-}F)}$ = 4.0, 1.1 Hz). HRMS-ESI [M + Na]$^+$ calcd for $C_6H_8FN_3O_3Na$ 212.0442; found 212.0449.

Phenyl 2-azido-2,3-dideoxy-3-fluoro-1-thio-α-D-mannopyranoside (α-23)

Phenyl 2-azido-2,3-dideoxy-3-fluoro-1-thio-β-D-mannopyranoside (β-23)

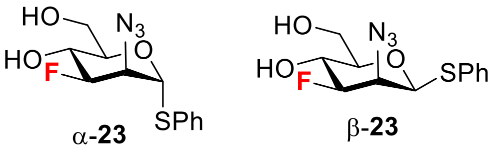

Thioglycosides α- and β-**23** were prepared by reaction of **22** (200 mg, 1.06 mmol) with PhSTMS (0.68 mL, 3.59 mmol) and ZnI_2 (0.60 g, 1.88 mmol) in 1,2-dichloroethane (4.5 mL) according to the general procedure. The reaction was completed in 48 h when TLC (EtOAc/PE 1:3) showed the absence of the starting material and the presence of less polar product. Column chromatography on silica gel in EtOAc/PE 1:5 → EtOAc/PE 1:2 first afforded the α-anomer α-**23** (157 mg, 50%) as a white crystalline solid, followed by the β-anomer β-**23** (113 mg, 36%) as a white crystalline solid.

Data for α-**23**: mp 109–111 °C (PE/EtOAc), R_f 0.20 (EtOAc/PE 1:3), $[\alpha]_D^{20}$ +109 (*c* 0.38, $CHCl_3$). ^{1}H NMR ($CDCl_3$, 400 MHz, ^{1}H{^{19}F}, H-H COSY): δ 7.47–7.45 (m, 2H, CH_{Ph}), 7.37–7.33 (m, 3H, CH_{Ph}), 5.48 (dd, 1H, J = 5.1, 1.5 Hz, H-1), 4.90 (ddd, 1H, J = 48.6, 9.0, 4.0 Hz, H-3), 4.36 (ddd, 1H, J = 5.7, 4.0, 1.5 Hz, H-2), 4.24 (ddd, 1H, J = 12.2, 9.8, 9.0 Hz, H-4), 4.14 (ddd, 1H, J = 9.8, 3.6, 3.3 Hz, H-5), 3.90 (dd, 1H, J = 11.7, 3.6 Hz, H-6), 3.86 (ddd, 1H, J = 11.7, 3.2, 1.3 Hz, H-6′), 2.83 (br s, 1H, O*H*-4), 2.02 (br s, 1H, O*H*-6). ^{13}C{^{1}H} NMR ($CDCl_3$, 101 MHz, proton-coupled-HSQC, HSQC): δ 132.50 (C_q), 132.46, 129.5 (2×2CH_{Ph}), 128.6 (CH_{Ph}), 92.5 (d, $^1J_{(C\text{-}F)}$ = 188.2 Hz, C-3), 86.3 (d, $^3J_{(C\text{-}F)}$ = 6.5 Hz, $^1J_{(C\text{-}H)}$ = 173.8 Hz, C-1), 72.9 (d, $^3J_{(C\text{-}F)}$ = 6.5 Hz, C-5), 66.5 (d, $^2J_{(C\text{-}F)}$ = 18.7 Hz, C-4), 63.1 (d, $^2J_{(C\text{-}F)}$ = 16.2 Hz, C-2), 61.8 (d, $^4J_{(C\text{-}F)}$ = 1.6 Hz, C-6). ^{19}F NMR ($CDCl_3$, 376 MHz): δ −198.63 (dddd, $^2J_{(H\text{-}F)}$ = 48.6 Hz, $^3J_{(H\text{-}F)}$ = 12.2, 5.7 Hz, $^4J_{(H\text{-}F)}$ = 5.1 Hz). HRMS ESI [M + Na]$^+$ calcd for $C_{12}H_{14}FN_3O_3SNa$ 322.0632; found 322.0633.

Data for β-**23**: mp 140–142 °C (PE/EtOAc), R_f 0.10 (EtOAc/PE 1:3), $[\alpha]_D^{20}$ +12 (*c* 0.41, $CHCl_3$). ^{1}H NMR ($CDCl_3$, 400 MHz, ^{1}H{^{19}F}, H-H COSY): δ 7.49–7.46 (m, 2H, CH_{Ph}), 7.36–7.30 (m, 3H, CH_{Ph}),

4.84 (dd, 1H, J = 1.6, 1.4 Hz, H-1), 4.66 (ddd, 1H, J = 48.5, 9.1, 4.1 Hz, H-3), 4.34 (ddd, 1H, J = 5.5, 4.1, 1.4 Hz, H-2), 4.20 (ddd, 1H, J = 12.3, 9.6, 9.1 Hz, H-4), 3.95 (ddd, 1H, J = 12.1, 3.3, 1.6 Hz, H-6), 3.86 (dd, 1H, J = 12.1, 4.7 Hz, H-6′), 3.33 (dddd, 1H, J = 9.6, 4.7, 3.3, 1.2 Hz, H-5), 2.74 (br s, 1H, O*H*-4), 2.17 (br s, 1H, O*H*-6). $^{13}C\{^1H\}$ NMR ($CDCl_3$, 101 MHz, proton-coupled-HSQC, HSQC): δ 133.5 (C_q), 131.7, 129.4 (2×2CH_{Ph}), 128.3 (CH_{Ph}), 94.7 (d, $^1J_{(C\text{-}F)}$ = 189.9 Hz, C-3), 85.4 (d, $^3J_{(C\text{-}F)}$ = 6.5 Hz, $^1J_{(C\text{-}H)}$ = 156.8 Hz, C-1), 79.2 (d, $^3J_{(C\text{-}F)}$ = 6.4 Hz, C-5), 66.3 (d, $^2J_{(C\text{-}F)}$ = 18.8 Hz, C-4), 64.0 (d, $^2J_{(C\text{-}F)}$ = 16.2 Hz, C-2), 62.2 (d, $^4J_{(C\text{-}F)}$ = 1.9 Hz, C-6). ^{19}F NMR ($CDCl_3$, 376 MHz): δ −193.25 (dddd, $^2J_{(H\text{-}F)}$ = 48.5 Hz, $^3J_{(H\text{-}F)}$ = 12.3, 5.5 Hz, $^4J_{(H\text{-}F)}$ = 1.6 Hz). HRMS ESI $[M + Na]^+$ calcd for $C_{12}H_{14}FN_3O_3SNa$ 322.0632; found 322.0647.

Phenyl 4,6-di-*O*-acetyl-2-azido-2,3-dideoxy-3-fluoro-1-thio-β-D-mannopyranoside (24)

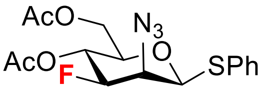

Compound **24** was prepared from compound β-**23** (100 mg, 0.33 mmol) according to the general acetylation procedure. Column chromatography on silica gel in EtOAc/PE (2:5) afforded **24** (110 mg, 86%) as a white crystalline solid, mp 136–138 °C (PE/EtOAc), R_f 0.30 (EtOAc/PE 1:2), $[\alpha]_D^{20}$ −31 (c 0.53, $CHCl_3$). ^1H NMR ($CDCl_3$, 400 MHz, $^1H\{^{19}F\}$, H-H COSY): δ 7.54–7.50 (m, 2H, CH_{Ph}), 7.35–7.30 (m, 3H, CH_{Ph}), 5.42 (ddd, 1H, J = 11.1, 10.1, 9.3 Hz, H-4), 4.77 (dd, 1H, J = 1.6, 1.4 Hz, H-1), 4.73 (ddd, 1H, J = 48.3, 9.3, 4.1 Hz, H-3), 4.37 (ddd, 1H, J = 5.5, 4.1, 1.4 Hz, H-2), 4.23 (dd, 1H, J = 12.3, 6.0 Hz, H-6), 4.17 (ddd, 1H, J = 12.3, 2.7, 0.9 Hz, H-6′), 3.54 (dddd, 1H, J = 10.1, 6.0, 2.7, 1.2 Hz, H-5), 2.11, 2.09 (2×s, 2×3H, *Me*). $^{13}C\{^1H\}$ NMR ($CDCl_3$, 101 MHz, proton-coupled-HSQC, HSQC, HMBC): δ 170.8 ($CO_{O\text{-}6}$), 169.5 ($CO_{O\text{-}4}$), 133.5 (C_q), 132.2, 129.3 (2×2CH_{Ph}), 128.4 (CH_{Ph}), 91.9 (d, $^1J_{(C\text{-}F)}$ = 194.7 Hz, C-3), 85.5 (d, $^3J_{(C\text{-}F)}$ = 6.0 Hz, $^1J_{(C\text{-}H)}$ = 156.0 Hz, C-1), 75.9 (d, $^3J_{(C\text{-}F)}$ = 6.7 Hz, C-5), 66.5 (d, $^2J_{(C\text{-}F)}$ = 18.9 Hz, C-4), 64.0 (d, $^2J_{(C\text{-}F)}$ = 16.2 Hz, C-2), 62.6 (d, $^4J_{(C\text{-}F)}$ = 2.2 Hz, C-6), 20.89, 20.86 (2×*Me*). ^{19}F NMR ($CDCl_3$, 376 MHz): δ −192.47 (ddd, $^2J_{(H\text{-}F)}$ = 48.3 Hz, $^3J_{(H\text{-}F)}$ = 11.1, 5.5 Hz). HRMS ESI $[M + Na]^+$ calcd for $C_{16}H_{18}FN_3O_5SNa$ 406.0843; found 406.0844.

Phenyl 2-azido-2,3-dideoxy-3-fluoro-4,6-di-*O*-propionyl-1-thio-β-D-mannopyranoside (25)

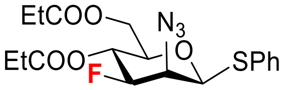

Propionyl chloride (120 µl, 1,38 mmol) was added to a solution of β-**23** (100 mg, 0.33 mmol) in pyridine (8 mL) at 0 °C and the reaction was allowed to warm to rt and then stirred for additional 18 hours. The reaction mixture was concentrated, co-distilled with toluene (3×), diluted with DCM, washed with water and the water phase was extracted with DCM (3×). Organic extracts were combined, dried and

concentrated. Column chromatography on silica gel in EtOAc/PE (1:5) provided **25** (82 mg, 60%) as a white crystalline solid, mp 149–151 °C (PE/EtOAc), R_f 0.45 (EtOAc/PE 1:3), $[\alpha]_D^{20}$ −24 (*c* 0.39, $CHCl_3$). ^{1}H NMR ($CDCl_3$, 400 MHz, ^{1}H{^{19}F}, H-H COSY): δ 7.54–7.50 (m, 2H, CH_{Ph}), 7.33–7.29 (m, 3H, CH_{Ph}), 5.42 (ddd, 1H, J = 11.1, 10.1, 9.3 Hz, H-4), 4.77 (t, 1H, J = 1.4 Hz, H-1), 4.73 (ddd, 1H, J = 48.2, 9.3, 4.1 Hz, H-3), 4.37 (ddd, 1H, J = 5.5, 4.1, 1.4 Hz, H-2), 4.21 (m, 2H, H-6, H-6′), 3.55 (dtd, 1H, J = 10.1, 4.4, 1.3 Hz, H-5), 2.44−2.29 (m, 4H, CH_2CH$_3$), 1.18−1.12 (m, 6H, CH$_2$CH_3). ^{13}C{^{1}H} NMR ($CDCl_3$, 101 MHz, proton-coupled-HSQC, HSQC, HMBC): δ 174.2 ($CO_{O\text{-}6}$), 173.1 ($CO_{O\text{-}4}$), 133.6 (C_q), 132.0, 129.3 (2×2CH_{Ph}), 128.4 (CH_{Ph}), 91.9 (d, $^1J_{(C\text{-}F)}$ = 194.5 Hz, C-3), 85.5 (d, $^3J_{(C\text{-}F)}$ = 6.2 Hz, $^1J_{(C\text{-}H)}$ = 156.6 Hz, C-1), 76.0 (d, $^3J_{(C\text{-}F)}$ = 6.7 Hz, C-5), 66.3 (d, $^2J_{(C\text{-}F)}$ = 18.7 Hz, C-4), 64.0 (d, $^2J_{(C\text{-}F)}$ = 16.2 Hz, C-2), 62.6 (d, $^4J_{(C\text{-}F)}$ = 1.9 Hz, C-6), 27.54, 27.51 (2×CH$_2$CH$_3$), 9.1 (2×CH$_2$$CH_3$). ^{19}F NMR ($CDCl_3$, 376 MHz): δ −192.51 (ddd, $^2J_{(H\text{-}F)}$ = 48.2 Hz, $^3J_{(H\text{-}F)}$ = 11.1, 5.3 Hz). HRMS ESI [M + Na]$^+$ calcd for $C_{18}H_{22}FN_3O_5SNa$ 434.1156; found 434.1155.

4,6-Di-*O*-acetyl-2-azido-2,3-dideoxy-3-fluoro-D-mannopyranose (26)

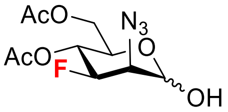

Thioglycoside **24** (95 mg, 0.25 mmol) was dissolved in a solution of acetone and water (9:1, v/v, mL) and hydrolyzed according to the general procedure using NBS (176 mg, 0.99 mmol). Chromatography on silica gel in EtOAc/PE 1:2 afforded **26** (61 mg, 85%) as a colorless syrupy mixture of anomers in 1:0.2 (α/β) ratio according to NMR analysis in $CDCl_3$, R_f 0.35 (EtOAc/PE 1:2). NMR data for the α-anomer: ^{1}H NMR ($CDCl_3$, 400 MHz, ^{1}H{^{19}F}, H-H COSY): δ 5.43 (ddd, 1H, J = 11.5, 10.0, 9.4 Hz, H-4), 5.30 (dd, 1H, J = 3.7, 1.8 Hz, H-1), 5.03 (ddd, 1H, J = 48.9, 9.4, 4.0 Hz, H-3), 4.25−4.12 (m, 3H, H-2, H-6, H-6′), 4.08 (dddd, 1H, J = 10.0, 4.3, 2.6, 1.1 Hz, H-5), 3.37 (d, 1H, J = 3.7 Hz, OH), 2.12, 2.11 (2×s, 2×3H, *Me*). ^{13}C{^{1}H} NMR ($CDCl_3$, 101 MHz, proton-coupled-HSQC, HSQC, HMBC): δ 171.1 ($CO_{O\text{-}6}$), 169.8 ($CO_{O\text{-}4}$), 93.3 (d, $^3J_{(C\text{-}F)}$ = 7.5 Hz, $^1J_{(C\text{-}H)}$ = 176.8 Hz, C-1), 89.5 (d, $^1J_{(C\text{-}F)}$ = 189.4 Hz, C-3), 68.5 (d, $^3J_{(C\text{-}F)}$ = 6.8 Hz, C-5), 66.7 (d, $^2J_{(C\text{-}F)}$ = 19.1 Hz, C-4), 62.3 (d, $^4J_{(C\text{-}F)}$ = 1.9 Hz, C-6), 62.1 (d, $^2J_{(C\text{-}F)}$ = 16.4 Hz, C-2), 20.94, 20.90 (2×*Me*). ^{19}F NMR ($CDCl_3$, 376 MHz): δ −202.22 (m, $^2J_{(H\text{-}F)}$ = 48.9 Hz is observed). NMR data for the β-anomer: ^{1}H NMR ($CDCl_3$, 400 MHz, ^{1}H{^{19}F}, H-H COSY): δ 5.35 (ddd, 1H, J = 11.2, 10.2, 9.4 Hz, H-4), 4.84 (br d, 1H, J = 10.0 Hz, H-1), 4.73 (ddd, 1H, J = 47.9, 9.4, 3.9 Hz, H-3), 4.25−4.12 (m, 3H, H-2, H-6, H-6′), 3.78 (d, 1H, J = 10.0 Hz, OH), 3.54 (dddd, 1H, J = 10.0, 5.1, 2.5, 1.1 Hz, H-5), 2.12, 2.10 (2×s, 2×3H, *Me*). ^{13}C{^{1}H} NMR ($CDCl_3$, 101 MHz, proton-coupled-HSQC, HSQC, HMBC): δ 171.0 ($CO_{O\text{-}6}$), 169.6 ($CO_{O\text{-}4}$), 92.5 (d, $^3J_{(C\text{-}F)}$ = 9.3 Hz, $^1J_{(C\text{-}H)}$ = 163.9 Hz, C-1), 91.1 (d, $^1J_{(C\text{-}F)}$ = 192.6 Hz, C-3), 71.5 (d, $^3J_{(C\text{-}F)}$ = 7.6 Hz, C-5), 66.1 (d, $^2J_{(C\text{-}F)}$ = 19.0 Hz, C-4), 63.9 (d, $^2J_{(C\text{-}F)}$ = 15.0 Hz, C-2), 62.2 (d, $^4J_{(C\text{-}F)}$ = 3.0 Hz, C-6), 20.92, 20.86 (2×*Me*). ^{19}F NMR

($CDCl_3$, 376 MHz): δ −195.75 (m, $^2J_{(H\text{-}F)}$ = 47.9 Hz is observed). HRMS ESI $[M + Na]^+$ calcd for $C_{10}H_{14}FN_3O_6Na$ 314.0759; found 314.0752.

2-Azido-2,3-dideoxy-3-fluoro-4,6-di-*O*-propionyl-D-mannopyranose (27)

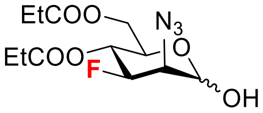

Thioglycoside **25** (65 mg, 0.16 mmol) was dissolved in a solution of acetone and water (9:1, v/v, 5 mL) and hydrolyzed according to the general procedure using NBS (122 mg, 0.69 mmol). Chromatography on silica gel in EtOAc/PE 1:2 afforded **27** (43 mg, 85%) as a colorless syrupy mixture of anomers in 1:0.2 (α/β) ratio according to NMR analysis in $CDCl_3$, R_f 0.30 (EtOAc/PE 1:2). NMR data for the α-anomer: ^{1}H NMR ($CDCl_3$, 400 MHz, $^1H\{^{19}F\}$, H-H COSY): δ 5.46 (ddd, 1H, J = 11.5, 10.0, 9.4 Hz, H-4), 5.30 (ddd, 1H, J = 4.6, 3.7, 1.8 Hz, H-1), 5.03 (ddd, 1H, J = 49.0, 9.4, 4.0 Hz, H-3), 4.24−4.18 (m, 2H, H-6, H-6′), 4.16 (ddd, 1H, J = 6.2, 4.0, 1.8 Hz, H-2), 4.08 (dt, 1H, J = 10.0, 3.7 Hz, H-5), 3.18 (d, 1H, J = 3.7 Hz, O*H*), 2.46−2.34 (m, 4H, $COCH_2$), 1.18−1.12 (m, 6H, CH_2CH_3). $^{13}C\{^1H\}$ NMR ($CDCl_3$, 101 MHz, proton-coupled-HSQC, HSQC, HMBC): δ 174.5 ($CO_{O\text{-}6}$), 173.2 ($CO_{O\text{-}4}$), 93.3 (d, $^3J_{(C\text{-}F)}$ = 7.5 Hz, $^1J_{(C\text{-}H)}$ = 176.3 Hz, C-1), 89.6 (d, $^1J_{(C\text{-}F)}$ = 189.5 Hz, C-3), 68.8 (d, $^3J_{(C\text{-}F)}$ = 6.9 Hz, C-5), 66.5 (d, $^2J_{(C\text{-}F)}$ = 19.1 Hz, C-4), 62.1 (d, $^2J_{(C\text{-}F)}$ = 15.3 Hz, C-2), 62.0 (d, $^4J_{(C\text{-}F)}$ = 2.0 Hz, C-6), 27.6, 27.5 (2×$COCH_2$), 9.14, 9.11 (2×CH_2CH_3). ^{19}F NMR ($CDCl_3$, 376 MHz): δ −202.30 (m, $^2J_{(H\text{-}F)}$ = 49.0 Hz is observed). NMR data for the β-anomer: ^{1}H NMR ($CDCl_3$, 400 MHz, $^1H\{^{19}F\}$, H-H COSY): δ 5.37 (ddd, 1H, J = 11.0, 9.9, 9.4 Hz, H-4), 4.84 (dt, 1H, J = 10.8, 1.6 Hz, H-1), 4.73 (ddd, 1H, J = 48.0, 9.4, 3.9 Hz, H-3), 4.24−4.18 (m, 3H, H-2, H-6, H-6′), 3.67 (d, 1H, J = 10.8 Hz, O*H*), 3.54 (dddd, 1H, J = 9.9, 5.0, 2.5, 1.3 Hz, H-5), 2.46−2.34 (m, 4H, $COCH_2$), 1.18−1.12 (m, 6H, CH_2CH_3). $^{13}C\{^1H\}$ NMR ($CDCl_3$, 101 MHz, proton-coupled-HSQC, HSQC, HMBC): δ 174.4 ($CO_{O\text{-}6}$), 173.1 ($CO_{O\text{-}4}$), 92.4 (d, $^3J_{(C\text{-}F)}$ = 8.9 Hz, $^1J_{(C\text{-}H)}$ = 167.8 Hz, C-1), 91.2 (d, $^1J_{(C\text{-}F)}$ = 192.6 Hz, C-3), 71.7 (d, $^3J_{(C\text{-}F)}$ = 6.9 Hz, C-5), 65.9 (d, $^2J_{(C\text{-}F)}$ = 18.9 Hz, C-4), 63.9 (d, $^2J_{(C\text{-}F)}$ = 15.1 Hz, C-2), 62.0 (d, $^4J_{(C\text{-}F)}$ = 2.6 Hz, C-6), 27.6, 27.5 (2×$COCH_2$), 9.11, 9.06 (2×CH_2CH_3). ^{19}F NMR ($CDCl_3$, 376 MHz): δ −195.81 (m, $^2J_{(H\text{-}F)}$ = 48.0 Hz is observed). HRMS ESI $[M + Na]^+$ calcd for $C_{12}H_{18}FN_3O_6Na$ 342.1072; found 342.1074.

Phenyl 2-azido-2,3,6-trideoxy-3,6-difluoro-1-thio-α-D-mannopyranoside (28)

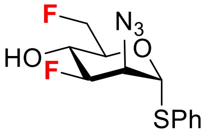

Thioglycoside (α-**23**) (100 mg, 0.33 mmol) was subjected to reaction with DAST (57 µl, 0.43 mmol) and 2,4,6-collidine (100 µl, 0.76 mmol) in dichloromethane (5 mL) according to the general procedure for C6 deoxyfluorination. Column chromatography on silica gel in EtOAc/PE 1:4 afforded product **28** (72 mg, 72%) as a white crystaline solid, mp 70–72 °C (PE/EtOAc), R_f 0.20 (EtOAc/PE 1:3), $[\alpha]_D^{20}$+95

(c 0.62, $CHCl_3$), ^{1}H NMR ($CDCl_3$, 400 MHz, $^1H\{^{19}F\}$, H-H COSY): δ 7.47–7.45 (m, 2H, CH_{Ph}), 7.37–7.31 (m, 3H, CH_{Ph}), 5.53 (dd, 1H, J = 5.3, 1.5 Hz, H-1), 4.89 (ddd, 1H, J = 48.6, 8.6, 3.9 Hz, H-3), 4.75 (ddd, 1H, J = 47.2, 10.3, 3.7 Hz, H-6), 4.63 (ddd, 1H, J = 47.9, 10.3, 1.7 Hz, H-6′), 4.37 (ddd, 1H, J = 5.6, 3.9, 1.5 Hz, H-2), 4.32–4.19 (m, 2H, H-4, H-5), 2.47 (s, 1H, OH). $^{13}C\{^1H\}$ NMR ($CDCl_3$, 101 MHz, proton-coupled-HSQC, HSQC): δ 132.6 (C_q Ph), 132.0, 129.5 (2×2CH$_{Ph}$), 128.5 (CH$_{Ph}$), 92.5 (dd, $^1J_{(C-F)}$ = 187.9 Hz, $^4J_{(C-F)}$ = 0.8 Hz, C-3), 86.2 (d, $^3J_{(C-F)}$ = 6.6 Hz, $^1J_{(C-H)}$ = 173.0 Hz, C-1), 81.5 (dd, $^1J_{(C-F)}$ = 174.1 Hz, $^4J_{(C-F)}$ = 1.9 Hz, C-6), 71.9 (dd, $^2J_{(C-F)}$ = 18.3 Hz, $^3J_{(C-F)}$ = 7.0 Hz, C-5), 65.6 (dd, $^2J_{(C-F)}$ = 19.4 Hz, $^3J_{(C-F)}$ = 7.6 Hz, C-4), 63.1 (d, $^2J_{(C-F)}$ = 16.1 Hz, C-2). ^{19}F NMR ($CDCl_3$, 376 MHz, $^{19}F\{^1H\}$): δ –198.63 (m, $^2J_{(H-F)}$ = 48.6 Hz is observed, F-3), –235.58 (m, F-6). HRMS ESI [M + Na]$^+$ calcd for $C_{12}H_{13}F_2N_3O_2SNa$ 324.0589; found 324.0596.

4-*O*-acetyl-2-azido-2,3,6-trideoxy-3,6-difluoro-D-mannopyranose (29)

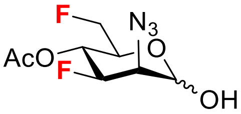

Ac_2O (1.5 mL) was added into a solution of **28** (70 mg, 0.23 mmol) in pyridine (2 mL) and the reaction mixture was stirred overnight, concentrated and co-distilled with toluene (3×). The crude acetylated intermediate was then dissolved in a solution of acetone and water (9:1, v/v, 5 mL) and hydrolyzed according to the general procedure using NBS (144 mg, 0.81 mmol). Chromatography on silica gel in EtOAc/PE 1:2 afforded **29** (46 mg, 79%) as a colorless syrupy mixture of anomers in 1:0.2 (α/β) ratio according to NMR analysis in $CDCl_3$, R_f 0.40 (EtOAc/PE 1:2). NMR data for the α-anomer: ^{1}H NMR ($CDCl_3$, 400 MHz, $^1H\{^{19}F\}$, H-H COSY): δ 5.37 (ddd, 1H, J = 11.7, 10.2, 9.3 Hz, H-4), 5.31 (dd, 1H, J = 4.7, 1.8 Hz, H-1), 5.06 (ddd, 1H, J = 48.8, 9.3, 3.9 Hz, H-3), 4.49 (ddd, 1H, J = 47.5, 10.3, 5.2 Hz, H-6), 4.44 (dddd, 1H, J = 46.6, 10.3, 2.6, 0.9 Hz, H-6′), 4.19 (ddd, 1H, J = 6.0, 3.9, 1.8 Hz, H-2), 4.11 (dddd, 1H, J = 21.7, 10.2, 5.2, 2.6 Hz, H-5), 3.25 (br s, 1H, OH), 2.14 (s, 3H, Me). $^{13}C\{^1H\}$ NMR ($CDCl_3$, 101 MHz, proton-coupled-HSQC, HSQC, HMBC): δ 169.8 (CO), 93.3 (d, $^3J_{(C-F)}$ = 7.4 Hz, $^1J_{(C-H)}$ = 176.5 Hz, C-1), 89.4 (dd, $^1J_{(C-F)}$ = 189.5 Hz, $^4J_{(C-F)}$ = 1.5 Hz, C-3), 81.8 (dd, $^1J_{(C-F)}$ = 174.4 Hz, $^4J_{(C-F)}$ = 2.2 Hz, C-6), 69.3 (dd, $^2J_{(C-F)}$ = 19.3 Hz, $^3J_{(C-F)}$ = 6.8 Hz, C-5), 66.4 (dd, $^2J_{(C-F)}$ = 19.2 Hz, $^3J_{(C-F)}$ = 7.2 Hz, C-4), 62.0 (d, $^2J_{(C-F)}$ = 15.4 Hz, C-2), 20.9 (Me). ^{19}F NMR ($CDCl_3$, 376 MHz, $^{19}F\{^1H\}$): δ –202.29 (m, F-3), –231.87 (dddd, $^2J_{(H-F)}$ = 47.5, 46.6 Hz, $^3J_{(H-F)}$ = 21.7 Hz, $^5J_{(F-F)}$ = 2.2 Hz, F-6). NMR data for the β-anomer: ^{1}H NMR ($CDCl_3$, 400 MHz, $^1H\{^{19}F\}$, H-H COSY): δ 5.35 (ddd, 1H, J = 11.5, 10.0, 9.4 Hz, H-4), 4.88 (dd, 1H, J = 1.7, 1.6 Hz, H-1), 4.76 (ddd, 1H, J = 47.9, 9.4, 3.9 Hz, H-3), overlapped with α-anomer (H-6, H-6′), 4.23 (ddd, 1H, J = 5.7, 4.0, 1.7 Hz, H-2), 3.57 (dddd, 1H, J = 20.6, 10.0, 6.1, 4.0 Hz, H-5), 3.25 (br s, 1H, OH), 2.14 (s, 3H, Me). $^{13}C\{^1H\}$ NMR ($CDCl_3$, 101 MHz, proton-coupled-HSQC, HSQC, HMBC): δ 169.7 (CO), 92.4 (d, $^3J_{(C-F)}$ = 9.5 Hz, $^1J_{(C-H)}$ = 165.6 Hz, C-1), 91.0 (dd, $^1J_{(C-F)}$ = 192.6 Hz, $^4J_{(C-F)}$ = 1.3 Hz, C-3), 81.4 (dd, $^1J_{(C-F)}$ = 175.6 Hz, $^4J_{(C-F)}$ = 2.6 Hz, C-6),

72.2 (dd, $^2J_{(C\text{-}F)}$ = 19.8 Hz, $^3J_{(C\text{-}F)}$ = 7.4 Hz, C-5), 65.8 (dd, $^2J_{(C\text{-}F)}$ = 19.2 Hz, $^3J_{(C\text{-}F)}$ = 6.9 Hz, C-4), 63.8 (d, $^2J_{(C\text{-}F)}$ = 15.4 Hz, C-2), 20.8 (*Me*). ^{19}F NMR ($CDCl_3$, 376 MHz, ^{19}F{^{1}H}): δ –195.65 (m, F-3), –232.19 (dddd, $^2J_{(H\text{-}F)}$ = 47.5, 46.9 Hz, $^3J_{(H\text{-}F)}$ = 20.6 Hz, $^5J_{(F\text{-}F)}$ = 2.2 Hz, F-6). HRMS ESI $[M + Na]^+$ calcd for $C_8H_{11}F_2N_3O_4Na$ 274.0610; found 274.0603.

Methyl 2-azido-6-*O*-benzoyl-3-*O*-benzyl-2,4-dideoxy-4-fluoro-α-D-mannopyranoside (30)

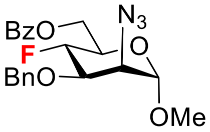

Compound **30** was prepared according to the published procedure.[5]

Methyl 2-azido-3-*O*-benzyl-2,4-dideoxy-4-fluoro-α-D-mannopyranoside (31)

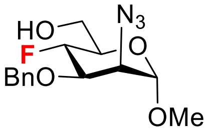

Ten drops of 0.5 M solution of MeONa in MeOH was added to a solution of **30** (182 mg, 0.44 mmol) in MeOH (8 mL) and the reaction mixture was stirred overnight until TLC indicated an absence of the starting material and a presence of one more polar product. The reaction was neutralized with DOWEX 50W ion exchange resin, filtered and concentrated. Column chromatography on silica gel in EtOAc/PE 1:2 afforded **31** (114 mg, 84%) as a colorless syrup, R_f 0.20 (EtOAc/PE 1:2), $[\alpha]_D^{20}$ +40 (*c* 0.82, $CHCl_3$). ^{1}H NMR ($CDCl_3$, 400 MHz, ^{1}H{^{19}F}, H-H COSY, HSQC): δ 7.40–7.28 (m, 5H, CH_{Ph}), 4.83 (d, 1H, *J* = 12.0 Hz, C*H*H Bn), 4.81 (ddd, 1H, *J* = 51.2, 9.7, 8.9 Hz, H-4), 4.71 (d, 1H, *J* = 12.0 Hz, C*H*H Bn), 4.66 (dd, 1H, *J* = 3.2, 1.7 Hz, H-1), 4.08 (ddd, 1H, *J* = 13.9, 8.9, 3.9 Hz, H-3), 3.94 (ddd, 1H, *J* = 3.9, 2.5, 1.7 Hz, H-2), 3.87 (dt, 1H, *J* = 12.1, 2.5 Hz, H-6), 3.80 (dd, 1H, *J* = 12.1, 4.6 Hz, H-6′), 3.71 (dddd, 1H, *J* = 9.7, 5.3, 4.6, 2.5 Hz, H-5), 3.36 (s, 3H, *Me*), 2.11 (br s, 1H, O*H*). ^{13}C{^{1}H} NMR ($CDCl_3$, 101 MHz, proton-coupled-HSQC, HSQC): δ 137.8 (C_q), 128.6 ($2CH_{Ph}$), 128.0 (CH_{Ph}), 127.7 ($2CH_{Ph}$), 99.3 (d, $^4J_{(C\text{-}F)}$ = 1.3 Hz, $^1J_{(C\text{-}H)}$ = 175.1 Hz, C-1), 88.0 (d, $^1J_{(C\text{-}F)}$ = 179.3 Hz, C-4), 76.7 (d, $^2J_{(C\text{-}F)}$ = 18.0 Hz, C-3), 73.1 (d, $^4J_{(C\text{-}F)}$ = 1.9 Hz, CH_2 Bn), 70.3 (d, $^2J_{(C\text{-}F)}$ = 25.6 Hz, C-5), 62.2 (d, $^3J_{(C\text{-}F)}$ = 8.9 Hz, C-2), 61.4 (C-6), 55.3 (*Me*). ^{19}F NMR ($CDCl_3$, 376 MHz): δ –203.29 (m, $^2J_{(H\text{-}F)}$ = 51.2 Hz is observed). HRMS ESI $[M + Na]^+$ calcd for $C_{14}H_{18}FN_3O_4Na$ 334.1174; found 334.1176.

2-Acetamido-1,3,6-tri-*O*-acetyl-2,4-dideoxy-4-fluoro-D-mannopyranose (32)

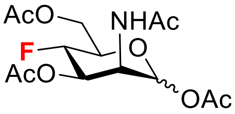

Compound **32** was prepared from compound **5** (119 mg, 0.36 mmol) according to the general procedure for azide to acetamide conversion using pyridine (0.4 mL, 5.0 mmol) and thioacetic acid (0.4 mL, 5.6 mmol). Column chromatography on silica gel in EtOAc/PE 3:2 afforded product **32** (109 mg, 87%) as a colorless syrupy mixture of anomers in 1:0.1 (α/β) ratio according to NMR analysis, R_f 0.25 (EtOAc/PE 3:2) NMR data for the α-anomer: ^{1}H NMR ($CDCl_3$, 400 MHz, ^{1}H{^{19}F}, H-H COSY): δ 6.00 (dd, 1H, J = 3.0, 1.8 Hz, H-1), 5.67 (d, 1H, J = 9.2 Hz, N*H*), 5.40 (ddd, 1H, J = 14.2, 9.7, 4.6 Hz, H-3), from ^{1}H{^{19}F} 5.67 (ddd, 1H, J = 9.2, 4.6, 1.8 Hz, H-2), 4.59 (ddd, 1H, J = 50.5, 9.9, 9.7 Hz, H-4), 4.38–4.29 (m, 2H, H-6, H-6′), 4.11 (dddd, 1H, J = 9.9, 4.8, 4.7, 3.1 Hz, H-5), 2.18, 2.11, 2.07, 2.05 (4×s, 4×3H, Me_{Ac}). ^{13}C{^{1}H} NMR ($CDCl_3$, 101 MHz, proton-coupled-HSQC, HSQC, HMBC): δ 170.6 (*C*O), 170.1 (2×*C*O), 168.3 (*C*O), 91.7 ($^1J_{(C\text{-}H)}$ = 178.9 Hz, C-1), 85.0 (d, $^1J_{(C\text{-}F)}$ = 185.0 Hz, C-4), 69.8 (d, $^2J_{(C\text{-}F)}$ = 24.2 Hz, C-5), 69.1 (d, $^2J_{(C\text{-}F)}$ = 18.4 Hz, C-3), 62.3 (C-6), 49.9 (d, $^3J_{(C\text{-}F)}$ = 7.8 Hz, C-2), 23.3, 21.0, 20.91, 20.90 (4×Me_{Ac}). ^{19}F NMR ($CDCl_3$, 376 MHz): δ –204.75 (m). Resolved signals for the β-anomer: ^{1}H NMR ($CDCl_3$, 400 MHz, ^{1}H{^{19}F}, H-H COSY): δ 5.87 (d, 1H, J = 1.8 Hz, H-1), 5.76 (d, 1H, J = 8.7 Hz, N*H*), 5.13 (ddd, 1H, J = 13.9, 9.4, 4.2 Hz, H-3), 4.78 (ddd, 1H, J = 8.7, 4.2, 1.8 Hz, H-2), 4.54 (ddd, 1H, J = 50.4, 9.7, 9.4 Hz, H-4), 4.38–4.29 (m, 2H, H-6, H-6′), 3.88 (dtd, 1H, J = 9.7, 4.7, 3.0 Hz, H-5). ^{13}C{^{1}H} NMR ($CDCl_3$, 101 MHz, proton-coupled-HSQC, HSQC, HMBC): δ 90.6 ($^1J_{(C\text{-}H)}$ = 165.9 Hz, C-1). ^{19}F NMR ($CDCl_3$, 376 MHz): δ −207.90 (m). HRMS ESI [M + Na]$^+$ calcd for $C_{14}H_{20}FNO_8Na$ 372.1065; found 372.1062.

Methyl 2-azido-3-*O*-benzyl-2,4,6-trideoxy-4,6-difluoro-α-D-mannopyranoside (33)

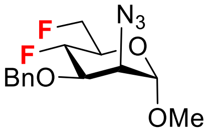

Compound **31** (178 mg, 0.57 mmol) was subjected to reaction with DAST (100 µl, 0.76 mmol) and 2,4,6-collidine (200 µl, 1,51 mmol) in dichloromethane (5 mL) according to the general procedure for C6 deoxyfluorination. Chromatography on silica gel of the crude product in EtOAc/PE 1:4 afforded compound **33** (161 mg, 90%) as a yellowish syrup, R_f 0.55 (EtOAc/PE 1:3), $[\alpha]_D^{20}$ +25 (c 1.03, $CHCl_3$). ^{1}H NMR ($CDCl_3$, 400 MHz, ^{1}H{^{19}F}, H-H COSY): δ 7.40–7.29 (m, 5H, CH_{Ph}), 4.83 (d, 1H, J = 11.9 Hz, CH*H* Bn), 4.77 (ddd, 1H, J = 51.3, 10.0, 8.9 Hz, H-4), 4.72 (d, 1H, J = 11.9 Hz, CH*H* Bn), 4.68 (dd, 1H, J = 2.5, 1.7 Hz, H-1), 4.69–4.57 (m, 2H, H-6, H-6′), 4.08 (ddd, 1H, J = 13.4, 8.9, 3.9 Hz, H-3), 3.95 (ddd, 1H, J = 3.9, 3.0, 1.7 Hz, H-2), 3.83 (ddddd, 1H, J = 24.5, 10.0, 5.9, 3.2, 1.3 Hz, H-5), 3.38 (s, 3H, *Me*). ^{13}C{^{1}H} NMR ($CDCl_3$, 101 MHz, proton-coupled-HSQC, HSQC): δ 137.7 (C_q), 128.7 (2CH_{Ph}),

128.1 (CH_{Ph}), 127.7 ($2CH_{Ph}$), 99.4 (d, $^{4}J_{(C\text{-}F)}$ = 1.3 Hz, $^{1}J_{(C\text{-}H)}$ = 174.9 Hz, C-1), 87.2 (dd, $^{1}J_{(C\text{-}F)}$ = 180.6 Hz, $^{3}J_{(C\text{-}F)}$ = 7.6 Hz, C-4), 81.3 (d, $^{1}J_{(C\text{-}F)}$ = 174.3 Hz, C-6), 76.7 (dd, $^{2}J_{(C\text{-}F)}$ = 17.6 Hz, $^{4}J_{(C\text{-}F)}$ = 1.0 Hz, C-3), 73.2 (d, $^{4}J_{(C\text{-}F)}$ = 2.0 Hz, CH_2 Bn), 69.2 (dd, $^{2}J_{(C\text{-}F)}$ = 24.2, 18.7 Hz, C-5), 62.1 (d, $^{3}J_{(C\text{-}F)}$ = 8.8 Hz, C-2), 55.5 (*Me*). ^{19}F NMR ($CDCl_3$, 376 MHz, $^{19}F\{^{1}H\}$): δ –203.46 (ddddd, $^{2}J_{(H\text{-}F)}$ = 51.3 Hz, $^{3}J_{(H\text{-}F)}$ = 13.4, 5.9 Hz, $^{4}J_{(H\text{-}F)}$ = 3.0 Hz, $^{5}J_{(H\text{-}F)}$ = 2.5 Hz, F-4), –235.20 (td, $^{2}J_{(H\text{-}F)}$ = 47.3 Hz, $^{3}J_{(H\text{-}F)}$ = 24.5 Hz, F-6). HRMS ESI $[M + Na]^+$ calcd for $C_{14}H_{17}F_2N_3O_3Na$ 336.1130; found 336.1136.

1,3-Di-*O*-acetyl-2-azido-2,4,6-trideoxy-4,6-difluoro-α-D-mannopyranose (34)

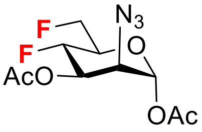

Compound **34** was prepared according to the general procedure for acid catalyzed acetolysis starting from **33** (157 mg, 0.50 mmol) and 2 mL of Ac_2O/H_2SO_4 solution. Column chromatography on silica gel in EtOAc/PE 1:2 provided **34** (119 mg, 81%) as a white crystalline α-anomer, R_f 0.40 (EtOAc/PE 1:2), mp. 116–118 °C (EtOAc/PE). ^{1}H NMR ($CDCl_3$, 400 MHz, $^{1}H\{^{19}F\}$, H-H COSY): δ 6.10 (dd, 1H, *J* = 3.0, 1.8 Hz, H-1), 5.47 (ddd, 1H, *J* = 13.3, 9.5, 4.0 Hz, H-3), 4.88 (ddd, 1H, *J* = 51.1, 9.5, 10.0 Hz, H-4), 4.79–4.56 (m, 2H, H-6, H-6′), 4.09 (ddd, 1H, *J* = 4.0, 3.0, 1.8 Hz, H-2), 4.00 (dddt, 1H, *J* = 25.7, 10.0, 5.2, 2.5 Hz, H-5), 2.19, 2.16 (2×s, 2×3H, *Me*). $^{13}C\{^{1}H\}$ NMR ($CDCl_3$, 101 MHz, proton-coupled-HSQC, HSQC, HMBC): δ 170.1 ($CO_{O\text{-}3Ac}$), 168.3 ($CO_{O\text{-}1Ac}$), 91.3 ($^{1}J_{(C\text{-}H)}$ = 175.5 Hz, C-1), 83.6 (dd, $^{1}J_{(C\text{-}F)}$ = 183.4 Hz, $^{3}J_{(C\text{-}F)}$ = 7.9 Hz, C-4), 80.5 (d, $^{1}J_{(C\text{-}F)}$ = 176.3 Hz, C-6), 71.4 (dd, $^{2}J_{(C\text{-}F)}$ = 24.3, 18.6 Hz, C-5), 70.6 (d, $^{2}J_{(C\text{-}F)}$ = 18.9 Hz, C-3), 61.0 (d, $^{3}J_{(C\text{-}F)}$ = 7.7 Hz, C-2), 21.0, 20.7 (2×*Me*). ^{19}F NMR ($CDCl_3$, 376 MHz, $^{19}F\{^{1}H\}$): δ –206.48 (m, $^{2}J_{(H\text{-}F)}$ = 51.1 Hz is observed, F-4), –237.17 (td, $^{2}J_{(H\text{-}F)}$ = 47.2 Hz, $^{3}J_{(H\text{-}F)}$ = 25.7 Hz, F-6). HRMS-ESI $[M + Na]^+$ calcd for $C_{10}H_{13}F_2N_3O_5Na$ 316.0716; found 316.0724.

Methyl 2-azido-3-*O*-benzyl-2-deoxy-α-D-mannopyranoside (35)

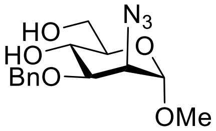

Compound **35** was prepared according to the known procedure.[5,6]

Methyl 2-azido-3-*O*-benzyl-2,6-dideoxy-6-fluoro-α-D-mannopyranoside (36)

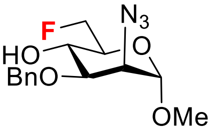

Methyl glycoside **35** (157 mg, 0.51 mmol) was subjected to reaction with DAST (79 µl, 0.60 mmol) and 2,4,6-collidine (165 µl, 1.25 mmol) in dichloromethane (6 mL) according to the general procedure for C6 deoxyfluorination. Column chromatography on silica gel in EtOAc/PE 1:5 afforded product **36** (140 mg, 89%) as a colorless syrup, R_f 0.15 (EtOAc/PE 1:5), $[\alpha]_D^{20}$+19 (*c* 1.31, $CHCl_3$), ^1H NMR ($CDCl_3$, 400 MHz, $^1H\{^{19}F\}$, H-H COSY): δ 7.39−7.32 (m, 5H, CH_{Ph}), 4.77 (d, 1H, J = 11.5 Hz, CH*H* Bn), 4.72 (d, 1H, J = 1.7 Hz, H-1), 4.63 (m, 2H, H-6), 4.58 (d, 1H, J = 11.5 Hz, CH*H* Bn), 3.96 (dd, 1H, J = 3.2, 1.7 Hz, H-2), 3.93–3.86 (m, 2H, H-3, H-4), 3.71 (ddt, 1H, J = 24.5, 9.0, 3.3 Hz, H-5), 3.37 (s, 3H, *Me*), 2.41 (s, 1H, O*H*). $^{13}C\{^1H\}$ NMR ($CDCl_3$, 101 MHz, HSQC, HMBC): δ 137.4 (C_q), 128.9 (2*C*H), 128.5 (*C*H), 128.2 (2*C*H), 99.5 (C-1), 82.3 (d, $^1J_{(C\text{-}F)}$ = 172.5 Hz, C-6), 79.6 (C-3), 72.4 (CH_2), 71.3 (d, $^2J_{(C\text{-}F)}$ = 18.2 Hz, C-5), 65.6 (d, $^3J_{(C\text{-}F)}$ = 7.7 Hz, C-4), 60.1 (C-2), 55.3 (*Me*). ^{19}F NMR ($CDCl_3$, 376 MHz): δ −235.14 (td, $^2J_{(H\text{-}F)}$ = 47.4 Hz, $^3J_{(H\text{-}F)}$ = 24.5 Hz). HRMS ESI $[M + Na]^+$ calcd for $C_{14}H_{18}FN_3O_4Na$ 334.1174; found 334.1182.

1,3,4-Tri-*O*-acetyl-2-acetamido-2,6-dideoxy-6-fluoro-D-mannopyranose (37)

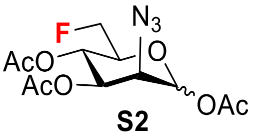

S2

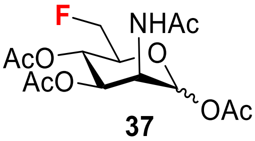

37

Compound **S2** was prepared according to the general procedure for acid catalyzed acetolysis starting from **36** (110 mg, 0.35 mmol) and 2 ml of Ac_2O/H_2SO_4 solution. Column chromatography on silica gel in EtOAc/PE 1:2 provided the intermediate 1,3,4-tri-*O*-acetyl-2-azido-2,6-dideoxy-6-fluoro-D-mannopyranose (**S2**, 114 mg, 97%) as a yellowish oily mixture of anomers in 1:0.1 (α/β) ratio according to NMR analysis, R_f 0.35 (EtOAc/PE 1:2). NMR data for the α-anomer: ^1H NMR ($CDCl_3$, 400 MHz, $^1H\{^{19}F\}$, H-H COSY): δ 6.12 (d, 1H, J = 1.9 Hz, H-1), 5.42–5.35 (m, 2H, H-3, H-4), 4.53–4.35 (m, 2H, H-6, H-6′), 4.04 (dd, 1H, J = 3.4, 1.9 Hz, H-2), 4.00 (dddd, 1H, J = 22.1, 10.3, 3.5, 3.2 Hz, H-5), 2.16, 2.12, 2.07 (3×s, 3×3H, *Me*). $^{13}C\{^1H\}$ NMR ($CDCl_3$, 101 MHz, proton-coupled-HSQC, HSQC): δ 170.2, 169.5, 168.3 (3×*C*O), 91.4 ($^1J_{(C\text{-}H)}$ = 180.9 Hz, C-1), 81.3 (d, $^1J_{(C\text{-}F)}$ = 176.0 Hz, C-6), 71.6 (d, $^2J_{(C\text{-}F)}$ = 19.6 Hz, C-5), 70.6 (C-3), 65.3 (d, $^3J_{(C\text{-}F)}$ = 6.7 Hz, C-4), 60.6 (C-2), 21.0, 20.7, 20.6 (3×*Me*). ^{19}F NMR ($CDCl_3$, 376 MHz): δ −233.32 (td, $^2J_{(H\text{-}F)}$ = 47.3 Hz, $^3J_{(H\text{-}F)}$ = 22.1 Hz). NMR data for the β-anomer: ^1H NMR ($CDCl_3$, 400 MHz, $^1H\{^{19}F\}$, H-H COSY): δ 5.86 (d, 1H, J = 1.4 Hz, H-1), 5.28 (dd, 1H, J = 9.9, 9.8 Hz, H-4), 5.10 (dd, 1H, J = 9.8, 3.6 Hz, H-3), 4.53–4.35 (m, 2H, H-6, H-6′), 4.15 (dd, 1H, J = 3.6, 1.4 Hz, H-2), 3.75 (dddd, 1H, J = 20.8, 9.9, 4.3, 2.9 Hz, H-5), 2.19, 2.12, 2.06 (3×s, 3×3H, *Me*). $^{13}C\{^1H\}$

NMR ($CDCl_3$, 101 MHz, proton-coupled-HSQC, HSQC): δ 170.2, 169.5, 168.5 (3×*C*O), 91.2 ($^1J_{(C-H)}$ = 167.0 Hz, C-1), 81.0 (d, $^1J_{(C-F)}$ = 176.4 Hz, C-6), 73.9 (d, $^2J_{(C-F)}$ = 19.7 Hz, C-5), 71.9 (C-3), 64.9 (d, $^3J_{(C-F)}$ = 6.8 Hz, C-4), 61.2 (C-2), 20.8, 20.73, 20.69 (3×*Me*). ^{19}F NMR ($CDCl_3$, 376 MHz): δ −233.94 (td, $^2J_{(H-F)}$ = 47.0 Hz, $^3J_{(H-F)}$ = 20.8 Hz).

Compound **37** was prepared from intermediate **S2** (129 mg, 0.39 mmol) according to the general procedure for azide to acetamide conversion using pyridine (0.4 mL, 5.0 mmol) and thioacetic acid (0.4 mL, 5.6 mmol). Chromatography on silica gel in EtOAc/PE (3:2) afforded product **37** (110 mg, 81%, 79% over two steps) as a colorless syrupy mixture of anomers in 1:0.03 (α/β) ratio according to NMR analysis, R_f 0.20 (EtOAc/PE 3:2). NMR data for the α-anomer: ^{1}H NMR ($CDCl_3$, 400 MHz, ^{1}H{^{19}F}, H-H COSY): δ 6.05 (d, 1H, J = 1.9 Hz, H-1), 5.91 (d, 1H, J = 9.3 Hz, N*H*), 5.36 (dd, 1H, J = 10.2, 4.4 Hz, H-3), 5.26 (dd, 1H, J = 10.2, 10.0 Hz, H-4), 4.64 (ddd, 1H, J = 9.3, 4.4, 1.9 Hz, H-2), 4.50 (ddd, 1H, J = 47.6, 10.7, 2.2 Hz, H-6), 4.43 (ddd, 1H, J = 46.8, 10.7, 3.2 Hz, H-6′), 3.96 (dddd, 1H, J = 25.8, 10.2, 3.2, 2.2 Hz, H-5), 2.17, 2.08, 2.06, 2.01 (4×s, 4×3H, *Me*). ^{13}C{^{1}H} NMR ($CDCl_3$, 101 MHz, proton-coupled-HSQC, HSQC, HMBC): δ 170.4, 170.3, 169.7, 168.3 (4×*C*O), 91.9 ($^1J_{(C-H)}$ = 179.5 Hz, C-1), 80.9 (d, $^1J_{(C-F)}$ = 176.1 Hz, C-6), 71.0 (d, $^2J_{(C-F)}$ = 18.7 Hz, C-5), 69.0 (C-3), 64.9 (d, $^3J_{(C-F)}$ = 6.5 Hz, C-4), 49.4 (C-2), 23.4, 21.0, 20.9, 20.8 (4×*Me*). ^{19}F NMR ($CDCl_3$, 376 MHz, ^{19}F{^{1}H}): δ –235.18 (ddd, $^2J_{(H-F)}$ = 47.6, 46.8 Hz, $^3J_{(H-F)}$ = 25.8 Hz, F-6). Resolved signals for β-anomer: ^{1}H NMR ($CDCl_3$, 400 MHz, ^{1}H{^{19}F}, H-H COSY): δ 5.87 (d, 1H, J = 1.8 Hz, H-1), 5.86 (d, 1H, J = 9.0 Hz, N*H*), 5.21 (dd, 1H, J = 10.0, 9.8 Hz, H-4), 5.07 (dd, 1H, J = 10.0, 4.1 Hz, H-3), 4.77 (ddd, 1H, J = 9.0, 4.1, 1.8 Hz, H-2), 3.75 (ddt, 1H, J = 24.1, 9.8, 2.9 Hz, H-5). ^{13}C{^{1}H} NMR ($CDCl_3$, 101 MHz, proton-coupled-HSQC, HSQC, HMBC): δ 90.7 ($^1J_{(C-H)}$ = 166.2 Hz, C-1), 74.0 (d, $^2J_{(C-F)}$ = 18.9 Hz, C-5), 71.6 (C-3), 64.7 (d, $^3J_{(C-F)}$ = 6.2 Hz, C-4), 49.7 (C-2). ^{19}F NMR ($CDCl_3$, 376 MHz, ^{19}F{^{1}H}): δ −234.61 (td, $^2J_{(H-F)}$ = 47.2 Hz, $^3J_{(H-F)}$ = 24.1 Hz). HRMS-ESI $[M + Na]^+$ calcd for $C_{14}H_{20}FNO_8Na$ 372.1065; found 372.1072.

1,6-Anhydro-2-azido-2,3-dideoxy-3-fluoro-β-D-talopyranose (38)

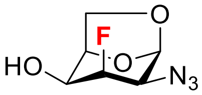

A solution of **22** (400 mg, 2.11 mmol) in DCM/pyridine (4:1, 7.5 mL) was cooled to −40 °C and trifluoromethanesulfonic anhydride (0.49 mL, 2.92 mmol) was added dropwise. The reaction mixture was allowed to warm up to 0 °C when TLC (EtOAc/PE 1:2) showed the absence of the starting material and the presence of less polar product. A few pieces of ice were added, and the reaction mixture was diluted with DCM. The aqueous phase was extracted with DCM (3×). The organic extracts were combined, dried, concentrated and co-distilled with toluene (3×). The crude intermediate was dissolved in DMF (8 mL) and potassium nitrite (0.75 g, 8.81 mmol) was added. The reaction mixture was stirred at 60 °C for 18 h, filtered and concentrated. Column chromatography on silica gel in EtOAc/PE 2:3

afforded **38** (290 mg, 73%) as a white crystalline solid, mp 134–136 °C (PE/EtOAc), R_f 0.10 (EtOAc/PE 1:2), $[\alpha]_D^{20}$ −151 (*c* 1.33, $CHCl_3$). ^1H NMR ($CDCl_3$, 400 MHz, $^1H\{^{19}F\}$, H-H COSY): δ 5.54 (dd, 1H, J = 1.8, 1.6 Hz, H-1), 5.10 (dddd, 1H, J = 52.8, 5.3, 3.9, 1.4 Hz, H-3), 4.51 (tdd, 1H, J = 5.3, 1.4, 1.1 Hz, H-5), 4.32 (dt, 1H, J = 8.1, 1.1 Hz, H-6^{en}), 4.03 (ddt, 1H, J = 24.9, 6.0, 5.3 Hz, H-4), 3.84 (ddt, 1H, J = 8.1, 5.3, 1.6 Hz, H-6^{ex}), 3.04 (ddd, 1H, J = 27.6, 3.9, 1.8 Hz, H-2), 2.47 (d, 1H, J = 6.0 Hz, O*H*). $^{13}C\{^1H\}$ NMR ($CDCl_3$, 101 MHz, HSQC): δ 100.2 (C-1), 91.3 (d, $^1J_{(C\text{-}F)}$ = 181.7 Hz, C-3), 74.3 (C-5), 67.2 (d, $^2J_{(C\text{-}F)}$ = 17.3 Hz, C-4), 65.2 (d, $^4J_{(C\text{-}F)}$ = 4.1 Hz, C-6), 59.2 (d, $^2J_{(C\text{-}F)}$ = 15.7 Hz, C-2). ^{19}F NMR ($CDCl_3$, 376 MHz): δ −215.60 (ddd, $^2J_{(H\text{-}F)}$ = 52.8 Hz, $^3J_{(H\text{-}F)}$ = 27.6, 24.9 Hz). HRMS ESI [M + Na]$^+$ calcd for $C_6H_8FN_3O_3Na$ 212.0442; found 212.0457.

1,6-Anhydro-2-azido-2,3,4-trideoxy-3,4-difluoro-β-D-talopyranose (39)

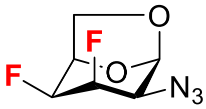

Solution of **38** (145 mg, 0.77 mmol) in DCM (5 ml) was cooled to −78 °C under an argon atmosphere and DAST (253 µl, 1.91 mmol) was added dropwise. The reaction was allowed to warm up to room temperature and stirred overnight. It was then quenched with saturated aqueous solution of $NaHCO_3$, diluted with DCM, washed with water and the aqueous phase was extracted with DCM (3×). The organic extracts were combined, dried and concentrated. Column chromatography on silica gel in EtOAc/PE 1:3 afforded **39** (124 mg, 85%) as a white crystalline solid, mp 95–97 °C (PE/EtOAc), R_f 0.30 (EtOAc/PE 1:3), $[\alpha]_D^{20}$ −43 (*c* 0.65, $CHCl_3$). ^1H NMR ($CDCl_3$, 400 MHz, $^1H\{^{19}F\}$, H-H COSY): δ 5.57 (ddd, 1H, J = 5.0, 1.9, 1.3 Hz, H-1), 5.22 (ddddd, 1H, J = 53.1, 5.2, 4.1, 3.3, 1.3 Hz, H-3), 4.73 (ddddd, 1H, J = 44.0, 24.4, 4.1, 4.0, 1.4 Hz, H-4), 4.67 (ddt, 1H, J = 5.1, 4.0, 0.9 Hz, H-5), 4.52 (dt, 1H, J = 8.0, 0.9 Hz, H-6^{en}), 3.89 (dddd, 1H, J = 8.0, 5.1, 3.1, 1.4 Hz, H-6^{ex}), 3.00 (ddd, 1H, J = 25.5, 3.3, 1.9 Hz, H-2). $^{13}C\{^1H\}$ NMR ($CDCl_3$, 101 MHz, HSQC): δ 100.5 (d, $^3J_{(C\text{-}F)}$ = 1.4 Hz, C-1), 88.6 (dd, $^1J_{(C\text{-}F)}$ = 190.4 Hz, $^2J_{(C\text{-}F)}$ = 15.2 Hz, C-3), 83.8 (dd, $^1J_{(C\text{-}F)}$ = 197.5 Hz, $^2J_{(C\text{-}F)}$ = 15.6 Hz, C-4), 71.9 (dd, $^2J_{(C\text{-}F)}$ = 27.3 Hz, $^3J_{(C\text{-}F)}$ = 1.0 Hz, C-5), 65.8 (dd, $^3J_{(C\text{-}F)}$ = 5.2 Hz, $^4J_{(C\text{-}F)}$ = 1.9 Hz, C-6), 59.2 (dd, $^2J_{(C\text{-}F)}$ = 16.0 Hz, $^3J_{(C\text{-}F)}$ = 1.2 Hz, C-2). $^{19}F\{^1H\}$ NMR ($CDCl_3$, 376 MHz, ^{19}F): δ −204.50 (d, $^3J_{(F\text{-}F)}$ = 7.4 Hz, F-4), −215.26 (d, $^3J_{(F\text{-}F)}$ = 7.4 Hz, F-3). HRMS-ESI [M + Na]$^+$ calcd for $C_6H_7F_2N_3O_2Na$ 214.0399; found 214.0404.

Phenyl 2-azido-2,3,4-trideoxy-3,4-difluoro-1-thio-α-D-talopyranoside (α-48)

Phenyl 2-azido-2,3,4-trideoxy-3,4-difluoro-1-thio-β-D-talopyranoside (β-48)

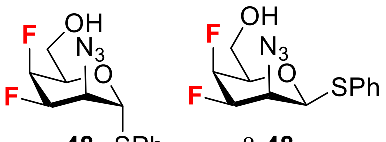

Thioglycosides **48** were prepared by reaction of the compound **39** (244 mg, 1.28 mmol) with PhSTMS (0.78 mL, 4.12 mmol) and ZnI_2 (0.71 g, 2.22 mmol) in 1,2-dichloroethane (4.5 mL) according to the general procedure. The reaction was completed in 48 h when TLC (EtOAc/PE 1:3) showed the absence of the starting material and the presence of a less polar product. Column chromatography on silica gel in EtOAc/PE 1:5 → EtOAc/PE 1:2 first afforded α-**48** (111 mg, 29%) as a colorless syrup followed by β-**48** (181 mg, 47%) as a white crystalline solid.

Data for α-**48**: R_f 0.30 (EtOAc/PE 1:3), $[\alpha]_D^{20}$+196 (*c* 0.42, $CHCl_3$). ^{1}H NMR ($CDCl_3$, 400 MHz, ^{1}H{^{19}F}, H-H COSY): δ 7.49–7.47 (m, 2H, CH_{Ph}), 7.36–7.34 (m, 3H, CH_{Ph}), 5.59 (dd, 1H, J = 6.2, 1.4 Hz, H-1), 5.00 (dddd, 1H, J = 51.6, 7.2, 3.0, 1.1 Hz, H-4), 4.95 (dddd, 1H, J = 43.1, 28.3, 4.3, 3.0 Hz, H-3), 4.46 (dddd, 1H, J = 29.0, 7.8, 5.2, 1.1 Hz, H-5), 4.24 (dddd, 1H, J = 5.5, 4.3, 1.4, 1.3 Hz, H-2), 3.94 (ddd, 1H, J = 11.6, 7.8, 4.0 Hz, H-6), 3.85 (ddd, 1H, J = 11.6, 8.5, 5.2 Hz, H-6′), 1.67 (dd, 1H, J = 8.5, 4.0 Hz, OH). ^{13}C{^{1}H} NMR ($CDCl_3$, 101 MHz, proton-coupled-HSQC, HSQC, HMBC): δ 132.8 (2CH$_{Ph}$), 131.7 (C_q), 129.6 (2CH$_{Ph}$), 128.9 (CH$_{Ph}$), 86.3 (dd, $^1J_{(C\text{-}F)}$ = 195.4 Hz, $^2J_{(C\text{-}F)}$ = 16.5 Hz, C-3), 86.1 (d, $^3J_{(C\text{-}F)}$ = 6.2 Hz, $^1J_{(C\text{-}H)}$ = 172.3 Hz, C-1), 85.3 (dd, $^1J_{(C\text{-}F)}$ = 191.6 Hz, $^2J_{(C\text{-}F)}$ = 17.0 Hz, C-4), 70.9 (dd, $^2J_{(C\text{-}F)}$ = 18.2 Hz, $^3J_{(C\text{-}F)}$ = 4.7 Hz, C-5), 61.1 (dd, $^3J_{(C\text{-}F)}$ = 6.5 Hz, $^4J_{(C\text{-}F)}$ = 2.4 Hz, C-6), 59.8 (d, $^2J_{(C\text{-}F)}$ = 17.4 Hz, C-2). ^{19}F NMR ($CDCl_3$, 376 MHz): δ –200.72 (m, F-3), –216.68 (m, F-4). HRMS-ESI [M + Na]$^+$ calcd for $C_{12}H_{13}F_2N_3O_2SNa$ 324.0589; found 324.0582.

Data for β-**48**: mp 124–126 °C (PE/EtOAc), R_f 0.15 (EtOAc/PE 1:3), $[\alpha]_D^{20}$+10 (*c* 0.19, $CHCl_3$). ^{1}H NMR ($CDCl_3$, 400 MHz, ^{1}H{^{19}F}, H-H COSY): δ 7.51–7.49 (m, 2H, CH_{Ph}), 7.36–7.32 (m, 3H, CH_{Ph}), 4.92 (dddd, 1H, J = 50.7, 6.2, 3.0, 1.8 Hz, H-4), 4.76 (t, 1H, J = 1.7 Hz, H-1), 4.75 (dddd, 1H, J = 42.7, 28.2, 4.3, 3.0 Hz, H-3), 4.32 (dddd, 1H, J = 4.5, 4.3, 1.7, 1.3 Hz, H-2), 4.04 (ddd, 1H, J = 11.7, 7.6, 4.0 Hz, H-6), 3.84 (ddd, 1H, J = 11.7, 8.7, 5.2 Hz, H-6′), 3.58 (dddd, 1H, J = 27.0, 7.6, 5.2, 1.8 Hz, H-5), 1.87 (dd, 1H, J = 8.7, 4.0 Hz, OH). ^{13}C{^{1}H} NMR ($CDCl_3$, 101 MHz, proton-coupled-HSQC, HSQC): δ 133.6 (C_q), 131.9, 129.4 (2×2CH$_{Ph}$), 128.4 (CH$_{Ph}$), 88.6 (dd, $^1J_{(C\text{-}F)}$ = 197.9 Hz, $^2J_{(C\text{-}F)}$ = 16.8 Hz, C-3), 86.0 (d, $^3J_{(C\text{-}F)}$ = 6.2 Hz, $^1J_{(C\text{-}H)}$ = 155.3 Hz, C-1), 84.0 (dd, $^1J_{(C\text{-}F)}$ = 191.7 Hz, $^2J_{(C\text{-}F)}$ = 17.0 Hz, C-4), 77.9 (dd, $^2J_{(C\text{-}F)}$ = 18.6 Hz, $^3J_{(C\text{-}F)}$ = 4.6 Hz, C-5), 61.2 (dd, $^3J_{(C\text{-}F)}$ = 6.3 Hz, $^4J_{(C\text{-}F)}$ = 2.9 Hz, C-6), 60.8 (dd, $^2J_{(C\text{-}F)}$ = 17.3 Hz, $^3J_{(C\text{-}F)}$ = 0.9 Hz, C-2). ^{19}F NMR ($CDCl_3$, 376 MHz): δ –195.25 (m, F-3), –217.64 (m, F-4). HRMS-ESI [M + Na]$^+$calcd for $C_{12}H_{13}F_2N_3O_2SNa$ 324.0589; found 324.0590.

Phenyl 6-*O*-acetyl-2-azido-2,3,4-trideoxy-3,4-difluoro-1-thio-β-D-talopyranoside (49)

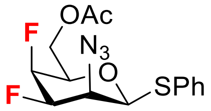

Compound **49** was prepared from compound β-**48** (80 mg, 0.27 mmol) according to the general acetylation procedure. Column chromatography on silica gel in EtOAc/PE 1:3 afforded **49** (85 mg, 93%) as a yellowish syrup R_f 0.30 (EtOAc/PE 1:3), $[\alpha]_D^{20}$+4 (*c* 0.48, $CHCl_3$). ^{1}H NMR ($CDCl_3$, 400 MHz, ^{1}H{^{19}F}, H-H COSY): δ 7.55–7.52 (m, 2H, CH_{Ph}), 7.33–7.31 (m, 3H, CH_{Ph}), 4.92 (ddddd, 1H, J = 50.6, 6.1, 3.0, 1.6, 1.0 Hz, H-4), 4.73 (dddd, 1H, J = 42.6, 28.1, 4.4, 3.0 Hz, H-3), 4.70 (dd, 1H, J = 2.2, 1.6 Hz, H-1), 4.42 (ddt, 1H, J = 11.6, 7.3, 1.1 Hz, H-6), 4.35 (dd, 1H, J = 11.6, 5.7 Hz, H-6′), 4.31 (dddd, 1H, J = 4.8, 4.4, 1.6, 1.0 Hz, H-2), 3.69 (dddd, 1H, J = 27.1, 7.3, 5.7, 1.6 Hz, H-5), 2.08 (s, 3H, *Me*). ^{13}C{^{1}H} NMR ($CDCl_3$, 101 MHz, proton-coupled-HSQC, HSQC, HMBC): δ 170.6 (*C*O), 133.5 (C_q), 132.4, 129.3 (2×2CH_{Ph}), 128.5 (CH_{Ph}), 88.4 (dd, $^1J_{(C\text{-}F)}$ = 198.1 Hz, $^2J_{(C\text{-}F)}$ = 16.8 Hz, C-3), 86.0 (d, $^3J_{(C\text{-}F)}$ = 6.2 Hz, $^1J_{(C\text{-}H)}$ = 156.5 Hz, C-1), 83.9 (dd, $^1J_{(C\text{-}F)}$ = 192.9 Hz, $^2J_{(C\text{-}F)}$ = 17.2 Hz, C-4), 74.9 (dd, $^2J_{(C\text{-}F)}$ = 18.6 Hz, $^3J_{(C\text{-}F)}$ = 5.4 Hz, C-5), 62.0 (dd, $^3J_{(C\text{-}F)}$ = 6.6 Hz, $^4J_{(C\text{-}F)}$ = 3.1 Hz, C-6), 60.5 (dd, $^2J_{(C\text{-}F)}$ = 17.2 Hz, $^3J_{(C\text{-}F)}$ = 0.9 Hz, C-2), 20.8 (*Me*). ^{19}F NMR ($CDCl_3$, 376 MHz): δ –195.35 (m, F-3), –218.21 (m, F-4). HRMS ESI [M + Na]$^+$ calcd for $C_{14}H_{15}F_2N_3O_3SNa$ 366.0694; found 366.0713.

Phenyl 2-azido-2,3,4,6-tetradeoxy-3,4,6-trifluoro-1-thio-α-D-talopyranoside (50)

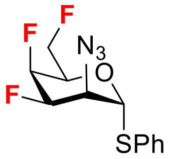

Thioglycoside α-**48** (80 mg, 0.27 mmol) was subjected to reaction with DAST (46 µl, 0.35 mmol) and 2,4,6-collidine (74 µl, 0.56 mmol) in dichloromethane (5 mL) according to the general procedure for C6 deoxyfluorination. Chromatography of the crude product on silica gel in EtOAc/PE 1:6 afforded compound **50** (70 mg, 87%) as a yellowish syrup, R_f 0.55 (EtOAc/PE 1:3), $[\alpha]_D^{20}$+52 (*c* 0.69, $CHCl_3$). ^{1}H NMR (MeOH-d_4, 400 MHz, ^{1}H{^{19}F}, H-H COSY): δ 7.54–7.52 (m, 2H, CH_{Ph}), 7.37–7.34 (m, 3H, CH_{Ph}), 5.59 (dd, 1H, J = 6.0, 1.6 Hz, H-1), 5.09 (dddd, 1H, J = 42.4, 28.2, 4.4, 3.0 Hz, H-3), 5.06 (dddd, 1H, J = 51.2, 9.1, 3.0, 1.4 Hz, H-4), 4.67 (ddd, 1H, J = 45.9, 9.7, 4.3 Hz, H-6), 4.56 (dddt, 1H, J = 47.6, 9.7, 7.2, 1.0 Hz, H-6′), 4.79–4.60 (m, 1H, H-5), 4.37 (dddd, 1H, J = 5.6, 4.4, 1.6, 1.0 Hz, H-2). ^{13}C{^{1}H} NMR (MeOH-d_4, 101 MHz, proton-coupled-HSQC, HSQC, HMBC): δ 133.7 (2*C*H, C_q), 130.3 (2*C*H), 129.5 (*C*H), 87.7 (ddd, $^1J_{(C\text{-}F)}$ = 192.4 Hz, $^2J_{(C\text{-}F)}$ = 16.1 Hz, $^4J_{(C\text{-}F)}$ = 1.5 Hz, C-3), 87.3 (d, $^3J_{(C\text{-}F)}$ = 6.4 Hz, $^1J_{(C\text{-}H)}$ = 175.7 Hz, C-1), 86.5 (ddd, $^1J_{(C\text{-}F)}$ = 190.1 Hz, $^2J_{(C\text{-}F)}$ = 17.5 Hz, $^3J_{(C\text{-}F)}$ = 7.5 Hz, C-4), 82.3 (ddd, $^1J_{(C\text{-}F)}$ = 169.5 Hz, $^3J_{(C\text{-}F)}$ = 7.0 Hz, $^4J_{(C\text{-}F)}$ = 3.4 Hz, C-6), 70.5 (ddd, $^2J_{(C\text{-}F)}$ = 23.4, 17.9 Hz, $^3J_{(C\text{-}F)}$ = 5.6 Hz, C-5), 60.8 (d, $^2J_{(C\text{-}F)}$ = 17.4 Hz, C-2). ^{19}F NMR (MeOH-d_4, 376 MHz): δ –202.87 (m, F-3), –218.76 (m, F-4), –233.98 (m, F-6). HRMS ESI [M + Na]$^+$ calcd for $C_{12}H_{12}F_3N_3OSNa$ 326.0545; found 326.0546.

6-*O*-Acetyl-2-azido-2,3,4-trideoxy-3,4-difluoro-D-talopyranose (51)

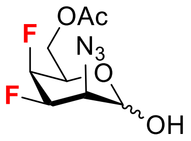

Thioglycoside **49** (62 mg, 0.18 mmol) was dissolved in a solution of acetone and water (9:1, v/v, 5 mL) and hydrolyzed according to the general procedure using NBS (130 mg, 0.73 mmol). Chromatography on silica gel in EtOAc/PE 1:2 afforded **51** (18 mg, 40%) as a colorless syrupy mixture of anomers (α/β = 1:0.2) according to NMR analysis in $CDCl_3$, R_f 0.30 (EtOAc/PE 1:2). NMR data for the α-anomer: ^{1}H NMR (MeOH-d_4, 400 MHz, H-H COSY): δ 5.17 (dd, 1H, J = 6.5, 1.9 Hz, H-1), 5.08 (dddd, 1H, J = 42.6, 29.7, 4.3, 3.0 Hz, H-3), 4.98 (dddd, 1H, J = 52.5, 7.6, 3.0, 1.6 Hz, H-4), 4.36–4.21 (m, 3H, H-5, H-6, H-6′), 4.04 (ddd, 1H, J = 5.6, 4.2, 1.9 Hz, H-2), 2.06 (s, 3H, *Me*). ^{13}C{^{1}H} NMR (MeOH-d_4, 101 MHz, HSQC, HMBC): δ 172.3 (*C*O), 94.6 (d, $^3J_{(C\text{-}F)}$ = 7.8 Hz, C-1), 87.7 (dd, $^1J_{(C\text{-}F)}$ = 189.4 Hz, $^2J_{(C\text{-}F)}$ = 16.1 Hz, C-3), 87.0 (dd, $^1J_{(C\text{-}F)}$ = 188.2 Hz, $^2J_{(C\text{-}F)}$ = 16.8 Hz, C-4), 67.8 (dd, $^2J_{(C\text{-}F)}$ = 18.0 Hz, $^3J_{(C\text{-}F)}$ = 5.9 Hz, C-5), 63.4 (dd, $^3J_{(C\text{-}F)}$ = 7.2 Hz, $^4J_{(C\text{-}F)}$ = 3.3 Hz, C-6), 60.7 (d, $^2J_{(C\text{-}F)}$ = 15.7 Hz, C-2), 20.6 (*Me*). ^{19}F NMR (MeOH-d_4, 376 MHz): δ –206.40 (m, F-3), –221.22 (m, F-4). NMR data for the β-anomer: ^{1}H NMR (MeOH-d_4, 400 MHz, H-H COSY): δ 4.87 (t, 1H, J = 1.4 Hz, H-1), 4.87 (dddd, 1H, J = 50.7, 4.2, 3.1, 2.1 Hz, H-4), 4.86 (dddd, 1H, J = 41.9, 30.1, 4.2, 3.1 Hz, H-3), 4.32–4.21 (m, 2H, H-6, H-6′), 4.09 (ddd, 1H, J = 5.8, 4.2, 1.4 Hz, H-2), 3.81 (ddddd, 1H, J = 27.6, 7.6, 5.4, 1.3, 1.0 Hz, H-5), 2.06 (s, 3H, *Me*). ^{13}C{^{1}H} NMR (MeOH-d_4, 101 MHz, HSQC, HMBC): δ 172.2 (*C*O), 94.7 (d, $^3J_{(C\text{-}F)}$ = 10.8 Hz, C-1), 88.8 (dd, $^1J_{(C\text{-}F)}$ = 192.0 Hz, $^2J_{(C\text{-}F)}$ = 16.0 Hz, C-3), 85.9 (dd, $^1J_{(C\text{-}F)}$ = 189.3 Hz, $^2J_{(C\text{-}F)}$ = 17.1 Hz, C-4), 71.9 (dd, $^2J_{(C\text{-}F)}$ = 17.9 Hz, $^3J_{(C\text{-}F)}$ = 6.8 Hz, C-5), 63.2 (dd, $^3J_{(C\text{-}F)}$ = 7.3 Hz, $^4J_{(C\text{-}F)}$ = 3.7 Hz, C-6), 62.4 (dd, $^2J_{(C\text{-}F)}$ = 16.1 Hz, $^4J_{(C\text{-}F)}$ = 0.7 Hz, C-2), 20.6 (*Me*). ^{19}F NMR (MeOH-d_4, 376 MHz): δ –201.93 (m, F-3), –220.14 (m, F-4). HRMS APCI [M + Na]$^+$ calcd for $C_8H_{11}F_2N_3O_4Na$ 274.0610; found 274.0615.

2-Azido-2,3,4,6-tetradeoxy-3,4,6-trifluoro-D-talopyranose (52)

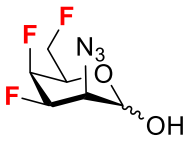

Thioglycoside **50** (64 mg, 0.21 mmol) was dissolved in a solution of acetone and water (9:1, v/v, 5 mL) and hydrolyzed according to the general procedure using NBS (163 mg, 0.92 mmol). Chromatography on silica gel in EtOAc/PE 1:2 followed by recrystallization in EtOAc/PE afforded **52** (19 mg, 43%) as a white crystalline solid in about 90% purity, mp 126-128 °C (PE/EtOAc), R_f 0.35 (EtOAc/PE 1:2), data for the α-anomer ^{1}H NMR ($CDCl_3$, 400 MHz, ^{1}H{^{19}F}, H-H COSY): δ 5.36 (ddd, 1H, J = 4.8, 3.8,

1.9 Hz, H-1), 5.07 (dddd, 1H, J = 43.4, 29.4, 4.2, 3.3 Hz, H-3), 5.08–4.91 (m, 1H, H-4), 4.69 (ddd, 1H, J = 46.0, 9.7, 5.8 Hz, H-6), 4.64 (dddt, 1H, J = 46.7, 9.7, 6.7, 1.3 Hz, H-6′), 4.35 (dddd, 1H, J = 28.9, 12.3, 6.7, 5.8 Hz, H-5), 4.11 (td, 1H, J = 4.2, 1.9 Hz, H-2), 3.00 (d, 1H, J = 3.8 Hz, O*H*). ^{13}C{^{1}H} NMR ($CDCl_3$, 101 MHz, proton-coupled-HSQC, HSQC, HMBC): δ 93.9 (d, $^{3}J_{(C\text{-}F)}$ = 7.6 Hz, $^{1}J_{(C\text{-}H)}$ = 178.4 Hz, C-1), 85.7 (ddd, $^{1}J_{(C\text{-}F)}$ = 192.5 Hz, $^{2}J_{(C\text{-}F)}$ = 16.3 Hz, $^{4}J_{(C\text{-}F)}$ = 1.3 Hz, C-3), 84.8 (ddd, $^{1}J_{(C\text{-}F)}$ = 191.3 Hz, $^{2}J_{(C\text{-}F)}$ = 17.5 Hz, $^{3}J_{(C\text{-}F)}$ = 6.2 Hz, C-4), 80.9 (ddd, $^{1}J_{(C\text{-}F)}$ = 169.9 Hz, $^{3}J_{(C\text{-}F)}$ = 7.4 Hz, $^{4}J_{(C\text{-}F)}$ = 3.0 Hz, C-6), 68.0 (ddd, $^{2}J_{(C\text{-}F)}$ = 24.1, 18.4 Hz, $^{3}J_{(C\text{-}F)}$ = 5.7 Hz, C-5), 58.7 (d, $^{2}J_{(C\text{-}F)}$ = 16.4 Hz, C-2). ^{19}F NMR ($CDCl_3$, 376 MHz, ^{19}F{^{1}H}): δ –205.13 (dd, $^{3}J_{(F\text{-}F)}$ = 13.1 Hz, $^{5}J_{(F\text{-}F)}$ = 2.2 Hz, F-3), –219.15 (dddd, $^{2}J_{(H\text{-}F)}$ = 51.2 Hz, $^{3}J_{(H\text{-}F)}$ = 29.4, 28.9 Hz, $^{3}J_{(F\text{-}F)}$ = 13.1 Hz, F-4), –233.01 (dddd, $^{2}J_{(H\text{-}F)}$ = 46.7, 46.0 Hz, $^{3}J_{(H\text{-}F)}$ = 12.3 Hz, $^{5}J_{(F\text{-}F)}$ = 2.2 Hz, F-6). HRMS ESI [M + Na]$^{+}$ = 234.0461, calcd for $C_6H_8F_3N_3O_2Na$; found 234.0463.

2-Acetamido-3-*S*-acetyl-2,3,4,6-tetradeoxy-4,6-difluoro-3-thio-α-D-galactopyranose (53)

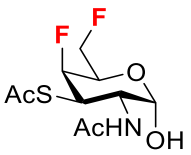

The reaction of compound **52** (11 mg, 0.05 mmol) with AcSH (6 µL, 0.09 mmol) and pyridine (6 µL, 0.08 mmol) in DCM (1 mL, added to increase solubility) according to the general procedure for azide-to-acetamide conversion overnight did not result in any conversion of the starting material, as confirmed by TLC analysis. Complete conversion was achieved by three subsequent additions of AcSH (25 µL, 0.35 mmol) and pyridine (25 µL, 0.31 mmol) over the course of 5 days, resulting in the formation of a more polar product. Chromatography on silica gel (EtOAc/PE 3:1) followed by recrystallization from EtOAc by slow cooling afforded compound **53** (7 mg, 47%) as a white crystalline α-anomer, mp > 170 °C (EtOAc, decomp.), R_f 0.50 (EtOAc). ^{1}H NMR ($CDCl_3$, 400 MHz, ^{1}H{^{19}F}, H-H COSY): δ 5.78 (d, 1H, 1H, J = 9.7 Hz, N*H*), 5.32 (d, 1H, J = 3.3 Hz, H-1), 4.75 (dd, 1H, J = 48.2, 2.2 Hz, H-4), 4.66–4.36 (m, 4H, H-2, H-5, H-6, H-6′), 4.12 (ddd, 1H, J = 35.3, 12.4, 2.2 Hz, H-3), 3.37 (br s, 1H, O*H*), 2.39 (s, 3H, Me_{AcS}), 1.96 (s, 3H, Me_{NHAc}). ^{13}C{^{1}H} NMR ($CDCl_3$, 101 MHz, HSQC): δ 196.4 (CO_{AcS}), 170.3 (CO_{NHAc}), 91.7 (C-1), 88.4 (dd, $^{1}J_{(C\text{-}F)}$ = 181.0 Hz, $^{3}J_{(C\text{-}F)}$ = 6.3 Hz, C-4), 81.4 (dd, $^{1}J_{(C\text{-}F)}$ = 169.4, $^{3}J_{(C\text{-}F)}$ = 6.6 Hz, C-6), 68.1 (dd, $^{2}J_{(C\text{-}F)}$ = 23.6, 18.7 Hz, C-5), 47.9 (d, $^{3}J_{(C\text{-}F)}$ = 1.7 Hz, C-2), 43.4 (d, $^{2}J_{(C\text{-}F)}$ = 19.4 Hz, C-3), 30.7 (Me_{AcS}), 23.3 (Me_{NHAc}). ^{19}F NMR ($CDCl_3$, 376 MHz, ^{19}F{^{1}H}): δ –207.96 (m, F-4), –232.76 (m, F-6). HRMS ESI [M + Na]$^{+}$ calcd for $C_{10}H_{15}F_2NO_4SNa$ 306.0582; found 306.0583. The structure of the α-anomer was confirmed by an X-ray single crystal diffraction analysis (Cambridge Crystallographic Data Centre deposition number CCDC 2513915).

1,6-Anhydro-2-O-benzyl-3,4-dideoxy-3,4-difluoro-β-D-glucopyranose (54)

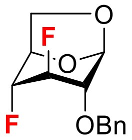

Compound **54** was prepared according to the known procedure.[7,8]

1,6-Anhydro-3,4-dideoxy-3,4-difluoro-β-D-glucopyranose (55)

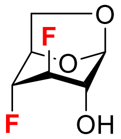

Compound **54** (290 mg, 1.13 mmol) was dissolved in MeOH (5 mL), Pd/C (40 mg) was added and the reaction mixture was stirred under H_2 atmosphere overnight, until TLC indicated an absence of the starting material and the presence of one polar product. The reaction mixture was diluted with EtOAc, filtered through celite and concentrated. Column chromatography on silica gel in EtOAc/PE 1:2 afforded **55** (171 mg, 91%) as white crystalline solid, mp 129–131 °C (EtOAc/heptane), R_f 0.20 (EtOAc/PE 1:2), $[\alpha]_D^{20}$ −64 (*c* 1.08, $CHCl_3$). ^1H NMR ($CDCl_3$, 400 MHz, $^1H\{^{19}F\}$, H-H COSY): δ 5.49 (t, 1H, J = 1.8 Hz, H-1), 4.74 (ddddd, 1H, J = 6.7, 5.9, 1.8, 1.1, 1.0 Hz, H-5), 4.69 (ddt, 1H, J = 42.8, 11.7, 1.8 Hz, H-3), 4.57 (ddq, 1H, J = 43.5, 12.2, 1.8 Hz, H-4), 4.03 (dtd, 1H, J = 8.0, 1.3, 1.1 Hz, H-6^{en}), 3.85 (dddd, 1H, J = 8.0, 5.9, 5.5, 1.8 Hz, H-6^{ex}), 3.69 (ddq, 1H, J = 13.2, 11.5, 1.8 Hz, H-2), 2.30 (d, 1H, J = 11.5 Hz, O*H*). $^{13}C\{^1H\}$ NMR ($CDCl_3$, 101 MHz, HSQC, HMBC): δ 101.3 (C-1), 88.2 (dd, $^1J_{(C\text{-}F)}$ = 181.4 Hz, $^2J_{(C\text{-}F)}$ = 31.2 Hz, C-3), 86.6 (dd, $^1J_{(C\text{-}F)}$ = 179.3 Hz, $^2J_{(C\text{-}F)}$ = 31.6 Hz, C-4), 73.6 (d, $^2J_{(C\text{-}F)}$ = 20.6 Hz, C-5), 67.2 (dd, $^2J_{(C\text{-}F)}$ = 24.7 Hz, $^3J_{(C\text{-}F)}$ = 1.4 Hz, C-2), 64.1 (dd, $^3J_{(C\text{-}F)}$ = 8.7 Hz, $^4J_{(C\text{-}F)}$ = 3.7 Hz, C-6). $^{19}F\{^1H\}$ NMR ($CDCl_3$, 376 MHz, ^{19}F): δ −189.13 (d, $^3J_{(F\text{-}F)}$ = 12.7 Hz, F-4), −190.50 (d, $^3J_{(F\text{-}F)}$ = 12.7 Hz, F-3). HRMS-APCI $[M + H]^+$calcd for $C_6H_9F_2O_3$ 167.0514; found 167.0506.

1,6-Anhydro-2-azido-2,3,4-trideoxy-3,4-difluoro-β-D-mannopyranose (56)

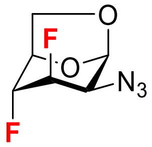

A solution of **55** (150 mg, 0.90 mmol) in dry DCM (4 mL) and pyridine (1 mL) was cooled to −40 °C under an argon atmosphere and triflic anhydride (228 µl, 1.36 mmol) was added dropwise. The reaction was allowed to warm up to 0 °C for two hours when TLC indicated an absence of the starting material and a presence of a less polar product, R_f 0.50 (EtOAc/PE 1:2). The reaction mixture was diluted with DCM and washed with iced water. The aqueous phase was extracted with DCM (3×). The organic

extracts were combined, dried, and concentrated and co-distilled with toluene (3×) affording a yellow crystalline intermediate, which was immediately dissolved in DMF (2.5 mL), NaN_3 (0.60 g, 9.23 mmol) was added and the reaction mixture was stirred at rt overnight until TLC indicated an absence of the intermediate and a presence of a more polar product. The reaction mixture was diluted with water and extracted with EtOAc (3×). Combined organic phases were dried and concentrated. Column chromatography on silica gel in EtOAc/PE 1:6 afforded **56** (135 mg, 78%) as a white crystalline solid, mp 89–90 °C (sublim., MTBE/heptane), R_f 0.20 (EtOAc/PE 1:2), $[\alpha]_D^{20}$ −145 (c 1.43, $CHCl_3$). ^{1}H NMR ($CDCl_3$, 400 MHz, ^{1}H{^{19}F}, H-H COSY): δ 5.61 (t, 1H, J = 1.9 Hz, H-1), 5.02 (dddt, 1H, J = 46.4, 8.2, 4.5, 1.9 Hz, H-3), 4.76 (ddt, 1H, J = 43.6, 10.8, 2.0 Hz, H-4), 4.76 (dddtd, 1H, J = 9.7, 5.8, 2.0, 1.9, 1.3 Hz, H-5), 4.14 (dtd, 1H, J = 8.1, 1.3, 0.8 Hz, H-6^{en}), 3.91 (dtd, 1H, J = 8.1, 5.8, 3.9 Hz, H-6^{ex}), 3.14 (dddd, 1H, J = 28.0, 4.5, 1.9, 1.4 Hz, H-2). ^{13}C{^{1}H} NMR ($CDCl_3$, 101 MHz, HSQC, HMBC): δ 100.8 (C-1), 88.3 (dd, $^1J_{(C\text{-}F)}$ = 182.4 Hz, $^2J_{(C\text{-}F)}$ = 34.5 Hz, C-3), 87.0 (dd, $^1J_{(C\text{-}F)}$ = 182.0 Hz, $^2J_{(C\text{-}F)}$ = 30.4 Hz, C-4), 73.3 (d, $^2J_{(C\text{-}F)}$ = 19.4 Hz, C-5), 64.1 (dd, $^3J_{(C\text{-}F)}$ = 7.8 Hz, $^4J_{(C\text{-}F)}$ = 5.1 Hz, C-6), 56.5 (d, $^2J_{(C\text{-}F)}$ = 16.1 Hz, C-2). ^{19}F{^{1}H} NMR ($CDCl_3$, 376 MHz, ^{19}F): δ −192.91 (d, $^3J_{(F\text{-}F)}$ = 13.2 Hz, F-4), −202.18 (d, $^3J_{(F\text{-}F)}$ = 13.3 Hz, F-3). HRMS-ESI $[M + Na]^+$ calcd for $C_6H_7F_2N_3O_2Na$ 214.0399; found 214.0407.

Phenyl 2-azido-2,3,4-trideoxy-3,4-difluoro-1-thio-β-D-mannopyranoside (β-57)

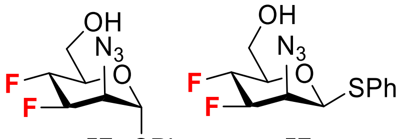

α-**57** β-**57**

Thioglycosides **57** were prepared by reaction of the compound **56** (127 mg, 0.66 mmol) with PhSTMS (0.39 mL, 2.06 mmol) and ZnI_2 (0.35 g, 1.10 mmol) in 1,2-dichloroethane (2 mL) according to the general procedure. The reaction was completed in 72 h, when TLC (EtOAc/PE 1:5) showed the absence of the starting material and the presence of less polar products. Column chromatography on silica gel in EtOAc/PE 1:5 first afforded a mixture of α-**57** and β-**57** (1:0.2, 36 mg, 18%) as a colorless syrup in 90% purity, followed by β-**57** (85 mg, 42%) as a white crystalline solid.

Data for α-**57**: R_f 0.20 (EtOAc/PE 1:5) ^{1}H NMR ($CDCl_3$, 400 MHz, ^{1}H{^{19}F}, H-H COSY): δ 7.48–7.45 (m, 2H, CH_{Ph}), 7.36–7.34 (m, 3H, CH_{Ph}), 5.48 (ddd, 1H, J = 4.4, 2.4, 1.6 Hz, H-1), 5.09 (dddd, 1H, J = 49.5, 14.7, 8.8, 4.1 Hz, H-3), 4.97 (dddd, 1H, J = 51.5, 13.2, 9.5, 8.8 Hz, H-4), 4.41 (dddd, 1H, J = 6.3, 4.1, 2.0, 1.6 Hz, H-2), 4.29 (ddddd, 1H, J = 9.5, 4.8, 4.1, 2.6, 0.6 Hz, H-5), 3.89 (dddd, 1H, J = 12.4, 2.6, 2.1, 1.6 Hz, H-6), 3.84 (ddd, 1H, J = 12.4, 4.1, 1.6 Hz, H-6′), 1.89 (br s, 1H, OH). ^{13}C{^{1}H} NMR ($CDCl_3$, 101 MHz, proton-coupled-HSQC, HSQC, HMBC): δ 132.5 (2CH$_{Ph}$), 132.0 (C_q), 129.6 (2CH$_{Ph}$), 128.8 (CH$_{Ph}$), 89.8 (dd, $^1J_{(C\text{-}F)}$ = 192.2 Hz, $^2J_{(C\text{-}F)}$ = 19.5 Hz, C-3), 86.0 (dd, $^3J_{(C\text{-}F)}$ = 6.0 Hz, $^4J_{(C\text{-}F)}$ = 1.1 Hz, $^1J_{(C\text{-}H)}$ = 170.0 Hz, C-1), 85.6 (dd, $^1J_{(C\text{-}F)}$ = 181.8 Hz, $^2J_{(C\text{-}F)}$ = 18.8 Hz, C-4), 71.2 (dd, $^2J_{(C\text{-}F)}$

= 25.4 Hz, $^3J_{(C-F)}$ = 6.0 Hz, C-5), 63.6 (dd, $^2J_{(C-F)}$ = 16.5 Hz, $^3J_{(C-F)}$ = 8.1 Hz, C-2), 60.9 (d, $^3J_{(C-F)}$ = 1.6 Hz, C-6). $^{19}F\{^1H\}$ NMR ($CDCl_3$, 376 MHz, ^{19}F): δ –198.14 (d, $^3J_{(F-F)}$ = 13.5 Hz, F-3), –206.51 (d, $^3J_{(F-F)}$ = 13.5 Hz, F-4). HRMS-ESI [M + Na]$^+$ calcd for $C_{12}H_{13}F_2N_3O_2SNa$ 324.0589; found 324.0590.

Data for β-**57**: mp 135–138 °C (MTBE), R_f 0.15 (EtOAc/PE 1:5), $[\alpha]_D^{20}$ −26 (*c* 1.29, $CHCl_3$). ^1H NMR ($CDCl_3$, 400 MHz, $^1H\{^{19}F\}$, H-H COSY): δ 7.49–7.47 (m, 2H, CH_{Ph}), 7.37–7.33 (m, 3H, CH_{Ph}), 4.91 (dddd, 1H, J = 50.3, 13.4, 9.4, 8.8 Hz, H-4), 4.86 (dddd, 1H, J = 50.7, 12.9, 8.8, 4.1 Hz, H-3), 4.85 (t, 1H, J = 1.4 Hz, H-1), 4.40 (dddd, 1H, J = 5.6, 4.1, 2.2, 1.4 Hz, H-2), 3.94 (ddddd, 1H, J = 12.5, 6.3, 2.4, 2.1, 1.6 Hz, H-6), 3.82 (dddd, 1H, J = 12.5, 7.6, 5.0, 1.6 Hz, H-6′), 3.46 (dddd, 1H, J = 9.4, 5.0, 4.0, 2.4 Hz, H-5), 2.03 (dd, 1H, J = 7.6, 6.3 Hz, O*H*). $^{13}C\{^1H\}$ NMR ($CDCl_3$, 101 MHz, proton-coupled-HSQC, HSQC, HMBC): δ 133.2 ($C_{q(Ph)}$), 131.9, 129.5 (2×2CH_{Ph}), 128.5 (CH_{Ph}), 92.0 (dd, $^1J_{(C-F)}$ = 194.0 Hz, $^2J_{(C-F)}$ = 19.1 Hz, C-3), 85.5 (d, $^3J_{(C-F)}$ = 5.8 Hz, $^1J_{(C-H)}$ = 158.2 Hz, C-1), 85.3 (dd, $^1J_{(C-F)}$ = 182.6 Hz, $^2J_{(C-F)}$ = 19.0 Hz, C-4), 77.6 (dd, $^2J_{(C-F)}$ = 24.2 Hz, $^3J_{(C-F)}$ = 5.9 Hz, C-5), 64.5 (dd, $^2J_{(C-F)}$ = 16.5 Hz, $^3J_{(C-F)}$ = 8.0 Hz, C-2), 61.4 (d, $^3J_{(C-F)}$ = 1.6 Hz, C-6). $^{19}F\{^1H\}$ NMR ($CDCl_3$, 376 MHz, ^{19}F): δ –192.79 (d, $^3J_{(F-F)}$ = 13.6 Hz, F-3), –208.88 (d, $^3J_{(F-F)}$ = 13.6 Hz, F-4). HRMS-ESI [M + Na]$^+$calcd for $C_{12}H_{13}F_2N_3O_2SNa$ 324.0589; found 324.0582.

Phenyl 6-*O*-acetyl-2-azido-2,3,4-trideoxy-3,4-difluoro-1-thio-β-D-mannopyranoside (58)

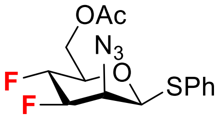

Compound **58** was prepared from compound β-**57** (130 mg, 0.43 mmol) according to the general acetylation procedure. Column chromatography on silica gel in EtOAc/PE 1:3 afforded **58** (135 mg, 91%) as a white crystalline solid, mp 109–111 °C (MTBE/heptane), R_f 0.20 (EtOAc/PE 1:3), $[\alpha]_D^{20}$ −30 (*c* 1.22, $CHCl_3$). ^1H NMR ($CDCl_3$, 400 MHz, $^1H\{^{19}F\}$, H-H COSY): δ 7.53–7.50 (m, 2H, CH_{Ph}), 7.34–7.32 (m, 3H, CH_{Ph}), 4.93–4.75 (m, 2H, H-3, H-4), from $^1H\{^{19}F\}$ 4.78 (d, 1H, J = 1.4 Hz, H-1), 4.46 (dt, 1H, J = 12.2, 2.4 Hz, H-6), 4.39 (dddd, 1H, J = 6.2, 3.4, 2.0, 1.4 Hz, H-2), 4.26 (ddd, 1H, J = 12.2, 6.2, 1.3 Hz, H-6′), 3.60 (dddd, 1H, J = 9.6, 6.2, 4.5, 2.4 Hz, H-5), 2.11 (s, 3H, *Me*). $^{13}C\{^1H\}$ NMR ($CDCl_3$, 101 MHz, proton-coupled-HSQC, HSQC, HMBC): δ 170.5 (*C*O), 133.1 (C_q), 132.2, 129.2 (2×CH_{Ph}), 128.4 (CH_{Ph}), 91.7 (dd, $^1J_{(C-F)}$ = 194.2 Hz, $^2J_{(C-F)}$ = 19.9 Hz, C-3), 85.6 (dd, $^1J_{(C-F)}$ = 184.6 Hz, $^2J_{(C-F)}$ = 19.1 Hz, C-4), 85.4 (dd, $^3J_{(C-F)}$ = 5.8 Hz, $^4J_{(C-F)}$ = 1.1 Hz, $^1J_{(C-H)}$ = 159.2 Hz, C-1), 75.0 (dd, $^2J_{(C-F)}$ = 22.9 Hz, $^3J_{(C-F)}$ = 6.8 Hz, C-5), 64.2 (dd, $^2J_{(C-F)}$ = 16.5 Hz, $^3J_{(C-F)}$ = 7.7 Hz, C-2), 62.4 (d, $^3J_{(C-F)}$ = 2.2 Hz, C-6), 20.8 (*Me*). $^{19}F\{^1H\}$ NMR ($CDCl_3$, 376 MHz, ^{19}F): δ –193.13 (d, $^3J_{(F-F)}$ = 13.5 Hz, F-3), –208.40 (d, $^3J_{(F-F)}$ = 13.5 Hz, F-4). HRMS-ESI [M + Na]$^+$ calcd for $C_{14}H_{15}F_2N_3O_3SNa$ 366.0694; found 366.0690.

6-*O*-acetyl-2-azido-2,3,4-trideoxy-3,4-difluoro-D-mannopyranose (59)

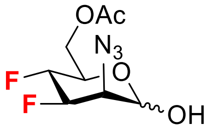

Thioglycoside **58** (135 mg, 0.39 mmol) was dissolved in a solution of acetone and water (in a ratio of 9:1, 10 mL) and hydrolyzed according to the general procedure using NBS (272 mg, 1.53 mmol). Chromatography on silica gel in EtOAc/PE 1:3 afforded **59** (62 mg, 63%) as a colorless syrupy mixture of anomers in 1:0.2 (α/β) ratio according to NMR analysis in $CDCl_3$, R_f 0.45 (EtOAc/PE 1:2). NMR data for the α-anomer: ^{1}H NMR ($CDCl_3$, 400 MHz, ^{1}H{^{19}F}, H-H COSY): δ 5.26 (ddd, 1H, J = 4.8, 2.5, 1.8 Hz, H-1), 5.14 (dddd, 1H, J = 49.7, 14.6, 8.8, 4.2 Hz, H-3), 4.85 (dddd, 1H, J = 52.1, 13.1, 9.8, 8.8 Hz, H-4), 4.47 (dddd, 1H, J = 12.2, 2.3, 1.9, 1.5 Hz, H-6), 4.23 (ddd, 1H, J = 12.2, 4.8, 1.5 Hz, H-6′), 4.19 (ddd, 1H, J = 6.1, 4.2, 1.8 Hz, H-2), 4.14 (ddddd, 1H, J = 9.8, 4.8, 3.1, 2.3, 0.7 Hz, H-5), 2.12 (s, 3H, *Me*). ^{13}C{^{1}H} NMR ($CDCl_3$, 101 MHz, proton-coupled-HSQC, HSQC, HMBC): δ 171.1 (CO_{Ac}), 93.2 (dd, $^3J_{(C\text{-}F)}$ = 7.3 Hz, $^4J_{(C\text{-}F)}$ = 1.1 Hz, $^1J_{(C\text{-}H)}$ = 176.6 Hz, C-1), 89.5 (dd, $^1J_{(C\text{-}F)}$ = 188.9 Hz, $^2J_{(C\text{-}F)}$ = 19.1 Hz, C-3), 85.9 (dd, $^1J_{(C\text{-}F)}$ = 182.7 Hz, $^2J_{(C\text{-}F)}$ = 19.1 Hz, C-4), 67.9 (dd, $^2J_{(C\text{-}F)}$ = 23.8 Hz, $^3J_{(C\text{-}F)}$ = 6.8 Hz, C-5), 62.4 (dd, $^2J_{(C\text{-}F)}$ = 15.4 Hz, $^3J_{(C\text{-}F)}$ = 7.7 Hz, C-2), 62.2 (d, $^3J_{(C\text{-}F)}$ = 2.4 Hz, C-6), 21.0 (*Me*). ^{19}F{^{1}H} NMR ($CDCl_3$, 376 MHz, ^{19}F): δ –202.85 (d, $^3J_{(F\text{-}F)}$ = 13.5 Hz, F-3), –206.61 (d, $^3J_{(F\text{-}F)}$ = 13.5 Hz, F-4). Resolved signals for the β-anomer: ^{1}H NMR ($CDCl_3$, 400 MHz, ^{1}H{^{19}F}, H-H COSY): δ 4.77 (ddd, 1H, J = 41.0, 9.6, 8.8 Hz, H-4), 4.45 (dddd, 1H, J = 12.2, 2.3, 1.8, 1.4 Hz, H-6), from ^{1}H{^{19}F-4} 3.61 (ddd, 1H, J = 9.6, 5.4, 2.3 Hz, H-5), 2.12 (s, 3H, *Me*). ^{13}C{^{1}H} NMR ($CDCl_3$, 101 MHz, proton-coupled-HSQC, HSQC, HMBC): δ 171.0 (CO_{Ac}), 92.5 (dd, $^3J_{(C\text{-}F)}$ = 8.8 Hz, $^4J_{(C\text{-}F)}$ = 1.2 Hz, $^1J_{(C\text{-}H)}$ = 164.7 Hz, C-1), 90.9 (dd, $^1J_{(C\text{-}F)}$ = 192.4 Hz, $^2J_{(C\text{-}F)}$ = 19.2 Hz, C-3), 85.5 (dd, $^1J_{(C\text{-}F)}$ = 184.1 Hz, $^2J_{(C\text{-}F)}$ = 19.3 Hz, C-4), 70.9 (dd, $^2J_{(C\text{-}F)}$ = 23.9 Hz, $^3J_{(C\text{-}F)}$ = 7.4 Hz, C-5), 64.1 (dd, $^2J_{(C\text{-}F)}$ = 15.4 Hz, $^3J_{(C\text{-}F)}$ = 8.1 Hz, C-2), 62.2 (d, $^3J_{(C\text{-}F)}$ = 1.8 Hz, C-6), 20.9 (*Me*). ^{19}F{^{1}H} NMR ($CDCl_3$, 376 MHz, ^{19}F): δ –196.36 (d, $^3J_{(F\text{-}F)}$ = 13.7 Hz, F-3), –209.85 (d, $^3J_{(F\text{-}F)}$ = 13.7 Hz, F-4). HRMS-ESI [M + Na]$^+$ calcd for $C_8H_{11}F_2N_3O_4Na$ 274.0610; found 274.0614.

Phenyl 2-azido-2,3,4,6-tetradeoxy-3,4,6-trifluoro-1-thio-β-D-mannopyranoside (60)

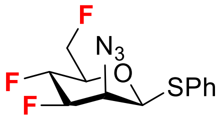

Thioglycoside β-**57** (74 mg, 0.25 mmol) was subjected to reaction with DAST (38 µl, 0.29 mmol) and 2,4,6-collidine (79 µl, 0.60 mmol) in dichloromethane (3 mL) according to the general procedure for C6 deoxyfluorination. Chromatography on silica gel of the crude product in EtOAc/PE 1:15 → EtOAc/PE 1:12 afforded compound **60** (41 mg, 55%) as a yellowish crystalline material, R_f 0.10 (EtOAc/PE 1:15), mp 119–121 °C (MTBE/heptane), $[\alpha]_D^{20}$ –14 (c 1.69, $CHCl_3$). ^{1}H NMR ($CDCl_3$, 400 MHz, ^{1}H{^{19}F}, H-

H COSY): δ 7.53–7.50 (m, 2H, CH_{Ph}), 7.37–7.33 (m, 3H, CH_{Ph}), 4.98–4.78 (m, 2H, H-3, H-4), 4.81 (t, 1H, J = 1.8 Hz, H-1), 4.67 (ddddd, 1H, J = 47.0, 10.5, 1.9, 1.8, 1.5 Hz, H-6), 4.64 (dddd, 1H, J = 47.1, 10.5, 4.8, 1.8 Hz, H-6′), 4.41 (ddd, 1H, J = 6.3, 3.3, 1.8 Hz, H-2), from $^{1}H\{^{19}F\text{-}4\}$ 3.59 (dddd, 1H, J = 22.0, 9.2, 4.8, 1.9 Hz, H-5). $^{13}C\{^{1}H\}$ NMR ($CDCl_3$, 101 MHz, proton-coupled-HSQC, HSQC, HMBC): δ 133.2 ($C_{q(Ph)}$), 132.3, 129.4 (2×2CH_{Ph}), 128.6 (CH_{Ph}), 91.8 (ddd, $^{1}J_{(C\text{-}F)}$ = 194.3 Hz, $^{2}J_{(C\text{-}F)}$ = 18.8 Hz, $^{4}J_{(C\text{-}F)}$ = 1.2 Hz, C-3), 85.7 (dd, $^{3}J_{(C\text{-}F)}$ = 6.9 Hz, $^{4}J_{(C\text{-}F)}$ = 1.3 Hz, $^{1}J_{(C\text{-}H)}$ = 156.4 Hz, C-1), 84.6 (ddd, $^{1}J_{(C\text{-}F)}$ = 184.6 Hz, $^{2}J_{(C\text{-}F)}$ = 19.3 Hz, $^{3}J_{(C\text{-}F)}$ = 8.2 Hz, C-4), 80.9 (dd, $^{1}J_{(C\text{-}F)}$ = 176.4 Hz, $^{3}J_{(C\text{-}F)}$ = 2.1 Hz, C-6), 76.2 (ddd, $^{2}J_{(C\text{-}F)}$ = 23.1, 19.5 Hz, $^{3}J_{(C\text{-}F)}$ = 6.7 Hz, C-5), 64.4 (dd, $^{2}J_{(C\text{-}F)}$ = 16.5 Hz, $^{3}J_{(C\text{-}F)}$ = 7.7 Hz, C-2). $^{19}F\{^{1}H\}$ NMR ($CDCl_3$, 376 MHz, ^{19}F): δ –192.90 (dd, $^{3}J_{(F\text{-}F)}$ = 13.4 Hz, $^{5}J_{(F\text{-}F)}$ = 1.8 Hz, F-3), –208.73 (d, $^{3}J_{(F\text{-}F)}$ = 13.4 Hz, F-4), –234.19 (d, $^{5}J_{(F\text{-}F)}$ = 1.8 Hz, F-6). HRMS APCI [M + Na]$^{+}$ calcd for $C_{12}H_{12}F_3N_3OSNa$ 326.0545; found 326.0567.

2-Azido-2,3,4,6-tetradeoxy-3,4,6-trifluoro-1-thio-β-D-mannopyranose (61)

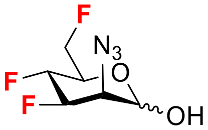

Thioglycoside **60** (140 mg, 0.46 mmol) was dissolved in a solution of acetone and water (9:1, v/v, 10 mL) and hydrolyzed according to the general procedure using NBS (280 mg, 1.57 mmol). Chromatography on silica gel in EtOAc/PE 2:5 afforded **61** (72 mg, 74%) as a colorless syrupy mixture of anomers in 1:0.2 (α/β) ratio according to NMR analysis in $CDCl_3$, R_f 0.35 (EtOAc/PE 1:2). NMR data for the α-anomer: ^{1}H NMR ($CDCl_3$, 400 MHz, $^{1}H\{^{19}F\}$, H-H COSY): δ 5.30 (td, 1H, J = 3.8, 1.8 Hz, H-1), 5.16 (dddd, 1H, J = 49.7, 14.6, 8.8, 4.2 Hz, H-3), 4.89 (dddd, 1H, J = 52.2, 12.9, 10.0, 8.8 Hz, H-4), 4.75–4.56 (m, 2H, H-6, H-6′), 4.22 (dddd, 1H, J = 6.2, 4.2, 2.4, 1.8 Hz, H-2), 4.13 (ddddd, 1H, J = 24.8, 10.0, 5.6, 3.1, 2.6 Hz, H-5), 3.10 (d, 1H, J = 3.8 Hz, OH). $^{13}C\{^{1}H\}$ NMR ($CDCl_3$, 101 MHz, proton-coupled-HSQC, HSQC, HMBC): δ 93.3 (dd, $^{3}J_{(C\text{-}F)}$ = 7.3 Hz, $^{4}J_{(C\text{-}F)}$ = 1.2 Hz, $^{1}J_{(C\text{-}H)}$ = 176.4 Hz, C-1), 89.5 (ddd, $^{1}J_{(C\text{-}F)}$ = 189.2 Hz, $^{2}J_{(C\text{-}F)}$ = 19.1 Hz, $^{4}J_{(C\text{-}F)}$ = 1.0 Hz, C-3), 85.0 (ddd, $^{1}J_{(C\text{-}F)}$ = 182.5 Hz, $^{2}J_{(C\text{-}F)}$ = 19.2 Hz, $^{3}J_{(C\text{-}F)}$ = 8.0 Hz, C-4), 81.0 (ddd, $^{1}J_{(C\text{-}F)}$ = 175.0 Hz, $^{3}J_{(C\text{-}F)}$ = 1.7 Hz, $^{4}J_{(C\text{-}F)}$ = 0.5 Hz, C-6), 69.0 (ddd, $^{2}J_{(C\text{-}F)}$ = 23.8, 18.7 Hz, $^{3}J_{(C\text{-}F)}$ = 6.9 Hz, C-5), 62.4 (dd, $^{2}J_{(C\text{-}F)}$ = 15.5 Hz, $^{3}J_{(C\text{-}F)}$ = 7.7 Hz, C-2). $^{19}F\{^{1}H\}$ NMR ($CDCl_3$, 376 MHz, ^{19}F): δ –202.87 (dd, $^{3}J_{(F\text{-}F)}$ = 13.3 Hz, $^{5}J_{(F\text{-}F)}$ = 1.6 Hz, F-3), –206.90 (d, $^{3}J_{(F\text{-}F)}$ = 13.3 Hz, F-4), –235.90 (d, $^{5}J_{(F\text{-}F)}$ = 1.6 Hz, F-6). Resolved signals for the β-anomer: ^{1}H NMR ($CDCl_3$, 400 MHz, $^{1}H\{^{19}F\}$, H-H COSY): δ 4.25 (dddd, 1H, J = 10.7, 5.9, 3.7, 2.0 Hz, H-2), 3.68 (d, 1H, J = 10.7 Hz, OH), 3.57 (ddtd, 1H, J = 25.7, 7.7, 3.8, 1.9 Hz, H-5). $^{13}C\{^{1}H\}$ NMR ($CDCl_3$, 101 MHz, proton-coupled-HSQC, HSQC, HMBC): δ 92.5 (dd, $^{3}J_{(C\text{-}F)}$ = 8.8 Hz, $^{4}J_{(C\text{-}F)}$ = 1.2 Hz, $^{1}J_{(H\text{-}C)}$ = 163.7 Hz, C-1), 91.0 (ddd, $^{1}J_{(C\text{-}F)}$ = 192.5 Hz, $^{2}J_{(C\text{-}F)}$ = 19.3 Hz, $^{4}J_{(C\text{-}F)}$ = 1.0 Hz, C-3), 84.4 (ddd, $^{1}J_{(C\text{-}F)}$ = 183.5 Hz, $^{2}J_{(C\text{-}F)}$ = 19.6 Hz, $^{3}J_{(C\text{-}F)}$ = 7.8 Hz, C-4), 80.6 (dd, $^{1}J_{(C\text{-}F)}$ = 176.2 Hz, $^{3}J_{(C\text{-}F)}$ = 2.2 Hz, C-6), 71.9 (ddd, $^{2}J_{(C\text{-}F)}$ = 24.2, 19.0 Hz, $^{3}J_{(C\text{-}F)}$ = 7.4 Hz, C-5), 64.1 (dd, $^{2}J_{(C\text{-}F)}$ = 15.6 Hz, $^{3}J_{(C\text{-}F)}$ = 8.1 Hz, C-2). $^{19}F\{^{1}H\}$ NMR

($CDCl_3$, 376 MHz, ^{19}F): δ –196.22 (dd, $^{3}J_{(F\text{-}F)}$ = 13.6 Hz, $^{5}J_{(F\text{-}F)}$ = 0.9 Hz, F-3), –210.30 (d, $^{3}J_{(F\text{-}F)}$ = 13.6 Hz, F-4), –235.97 (d, $^{5}J_{(F\text{-}F)}$ = 0.9 Hz, F-6). HRMS-APCI $[M - N_2 + H]^+$ calcd for $C_6H_8F_3NO_2$ 184.0580; found 184.0569.

C. *In vitro* biological activity

C1. MTT assay

The cells were seeded in a 96-well plate, 5,000 cells/well (100 µl) for MDA-MB-231 and 8,000 cells/well for MCF-10A. The plates were then incubated for 24 hours and consequently treated with tested compounds with a concentration gradient of 300 µM, 75 µM, 30 µM, 15 µM, 7.5 µM, 3 µM, 0.6 µM and 0.15 µM with 5 replicates per condition. Plates were then incubated for 72 hours. Afterwards, 20 µl of MTT (Serva) dissolved in PBS was added per well at a final concentration of 2.5 mg/ml. After 3-hour incubation and medium removal, 50 µl of DMSO (Serva) was added per well to dissolve the formazan crystals. Absorbance was measured at 570 nm using Microplate Reader: Infinite® M1000 PRO (Tecan).

C2. Cell proliferation assay

The MDA-MB-231 cells were seeded in a 96-well plate, at a density of 5,000 cells/well. On the next day, the cells were treated with tested compounds with a concentration gradient of 50 µM, 25 µM, and 10 µM, and alternatively with a concentration gradient of 30 µM, 20 µM, and 10 µM, with three replicates per condition and DMSO as the negative control. The plate was then placed into the Incucyte Live-Cell Analysis system (Sartorius), creating 4 scans per well every 6 hours for 3 days, with a 10 × objective and phase contrast.

C3. Colony forming assay

The MDA-MB-231 cells were seeded into 6-well plates in initial number of 100 cells/well. The next day, the seeded cells were treated with 2.5 µM of the tested compounds, using at least two replicates per condition, with DMSO as the negative control. Cells were then further incubated for 10 days until the colonies were visible by eye. Afterwards, the medium was aspirated, cells were washed with PBS and stained with fixing solution (0.05 mg/ml crystal violet (Merck), 37 % formaldehyde in 2.7 % final concentration (Dr. Kulich Pharma), 10 % PBS, 1 % methanol (Penta) and distilled water) for 20 minutes at room temperature. Plates were then washed with tap water and counted manually after drying.

C4. Cell cycle analysis

The MDA-MB-231 cells were grown on 6-well plates (250,000 cells/well). On the next day, the cells were treated with tested compounds at 50 µM, 30 µM (**8**), and 20 µM (**11**) concentrations. DMSO served as the negative control. Plates were then incubated for 48 hours. Cells were harvested using trypsin into 2 ml microtubes and washed with PBS. The pellets were resuspended in 100 µl of PBS. 400 µl of 70 % ethanol (Fagron) (not denatured) was added to fixate the cells at 4 °C overnight. Afterwards, the cells were centrifuged and washed with PBS. The cells were then stained with 1 ml of propidium iodide staining solution (0.1 % Triton-X (Sigma-Aldrich), 10 ug/ml PI (Sigma-Aldrich), 100 ug/ml DNase free RNAse A (Goldbio) in PBS) per sample and incubated for 30 minutes, room temperature, in the dark.

The fluorescence signal was detected at the flow cytometer FACS Verse (BD Biosciences). In total, 10,000 events were recorded per sample.

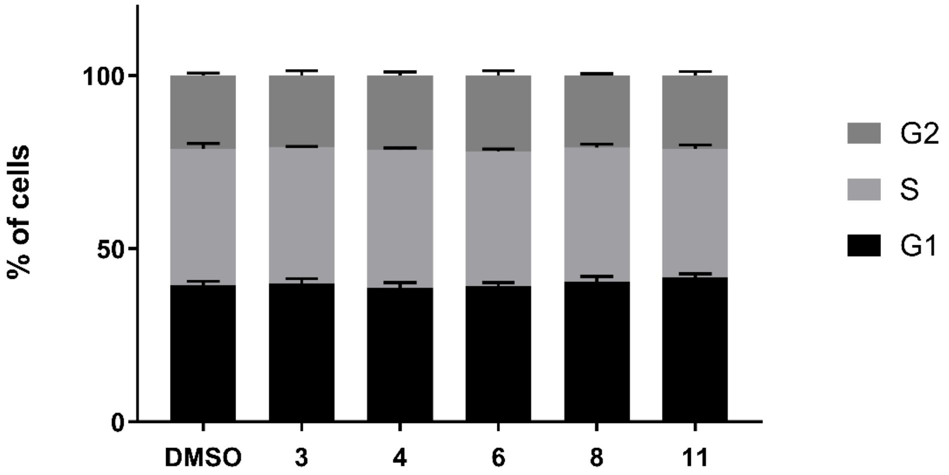

Figure S1. Cell cycle analysis of MDA-MB-231 treated with **3**, **4**, **6**, **8**, **11**, and **13**, using DMSO as control.

C5. Wound healing assay

The MDA-MB-231 cells were seeded in an Incucyte Imagelock 96-well plate (Sartorius) at a density of 80,000 cells/well. After a 24-hour incubation, a wound was created in each well using the Incucyte 96-Well Woundmaker Tool (Sartorius). The medium was aspirated, and all wells were washed twice with PBS to create truly cell-free zones. After washing, 100 µl of media without FBS was added, containing the tested compounds at 100 µM concentration. The plate was placed into the Incucyte Live-Cell Analysis System (Sartorius). Scans were acquired every 4 hours for 2 days, with a 10 × objective and phase contrast.

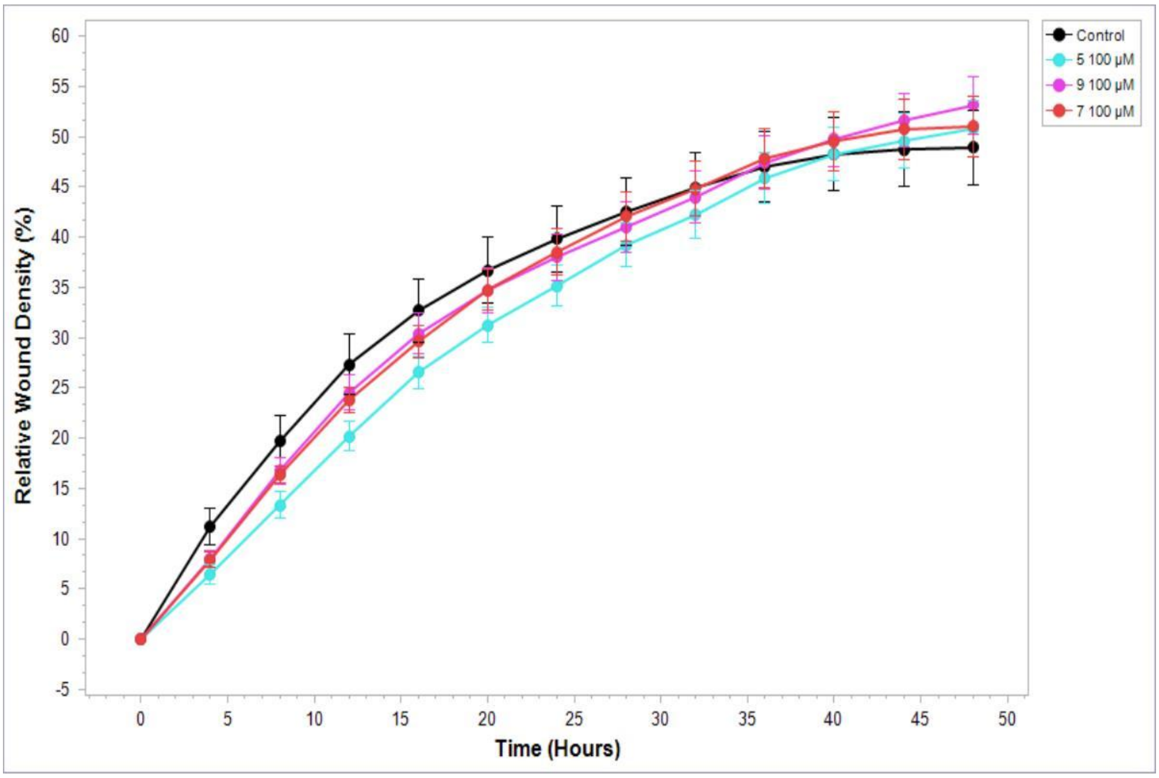

Figure S2. Scratch assay of MDA-MB-231 cells treated with **5**, **7**, and **9** with DMSO as control. The metric used to analyse and interpret the collected data is relative wound density, which is a measure (%) of the density of the wound region relative to the density of the cell region.

C6. SDS-PAGE and western blotting

The MDA-MB-231 cells were seeded in tissue culture dishes at 800,000 cells per dish and treated with a 50 μM concentration of tested compounds the next day. After 48-hour incubation, cells were harvested on ice, washed twice with cold PBS and scraped into NET lysis buffer (150 mM NaCl; 1 % NP-40; 50 mM Tris pH 8.0; 50 mM NaF; 5 mM EDTA pH 8.0) supplemented with 1:100 protease and phosphatase inhibitor cocktail (Thermo Scientific). The cells were then incubated on ice for 30 minutes and vortexed at regular intervals. Afterwards, they were centrifuged for 30 minutes at 14,000 rpm and the supernatant was transferred into a new microtube. The samples were diluted to the same concentration using NET lysis buffer and 4× CSB (29.41 % glycerol; 23.53 % Tris (1 M pH 6.8); 0.157 % bromophenol blue (2 % in 1 M Tris, pH 6.8); 39.22 % SDS (20 %)) and 20 % mercaptoethanol), to obtain the final concentration of 1× CSB. The samples were heated up at 105 °C in a thermoblock (Bioer) for 10 minutes, and stored in a freezer at -20 °C.

Prior to the electrophoresis, the 10 % polyacrylamide gel was prepared, poured, and left to solidify (30 % Acrylamide (Serva); 1 M Tris (pH 6.8); distilled water; APS (Sigma-Aldrich); TEMED (Sigma-Aldrich)). The samples were loaded into the polyacrylamide gel, and electrophoresis was performed in

1× MOPS buffer (20× MOPS: 1 M MOPS; 1M Tris base; 70 mM SDS; 20 mM EDTA acid) at 80 V for about 15 minutes and then 120 V.

The gels were then equilibrated in blotting buffer (25 mM Tris base; 192 mM glycin; 15% methanol) and transferred to a membrane (Pall Life Sciences) at 100 V for 90 minutes. The membranes were stained using the Ponceau solution (Sigma-Aldrich) (0.1 % Ponceau S in 5% acetic acid) to confirm the presence of the proteins of interest, washed in PBS-Tween 20 (1× PBS; 0.1% Tween 20 (Sigma-Aldrich)) and blocked in 5% skimmed milk or 3-5% BSA (Sigma-Aldrich) dissolved in PBS-Tween 20 solution.

Primary antibodies diluted in milk or BSA were prepared (Table 1) and incubated with the membranes overnight at 4°C. The membranes were then washed with PBS-Tween 20 (4× 10 minutes) and incubated with their corresponding peroxidase conjugated secondary antibodies (Table X) for 1 hour at room temperature. Afterwards, the membranes were washed with PBS-Tween 20 (4× 10 minutes) and incubated in the ECL A (200 mM Tris pH 9.4; 10 mM luminol; 405 mM p-coumaric acid; 0.5 mM EDTA pH 8.0) and ECL B (0.5 mM EDTA; 8 mM sodium perborate tetrahydrate; 50 mM sodium acetate pH 5.0) solutions, 1:1, for 5 minutes. The signal was captured using G:BOX Chem XX6 (Syngene).

Table 1. Antibodies and their dilutions

antibody	dilution	supplier
PARP	1:1000	Cell Signalling (9542)
p62	1:1000	Cell Signalling (39749)
GAPDH	1:5000	Abcam (ab110305)
LC3B	1:1000	Cell Signalling (2775)
p-AMPKα	1:500	Cell Signalling (2535)
AMPKα 1/2	1:500	Santa Cruz (sc-74461)
p21	1:1000	Cell Signalling (2947)
γH2AX	1:1000	Invitrogen (MA1-2022)
cyclin B1	1:1000	Cell Signalling (12231)
cyclin D1	1:1000	Thermo Scientific (RM-9104-S1)
GAR	1:1000	Jackson (111-035-003)
GAM	1:1000	Jackson (115-035-003)

GAR (peroxidase-conjugated goat anti-rabbit)

D. X-Ray

The single crystal of compound **53** was mounted on Bruker D8 VENTURE Kappa Duo PHOTONIII diffractometer by IμS micro-focus sealed tube MoKα (λ= 0.71073 Å). During the measurement of diffraction data, the crystal was kept on low temperature preserved by Cryostream 1000. The structure was solved by direct methods (XT)[9] and refined by full matrix least squares based on F^2 (SHELXL2019).[9] The hydrogen atoms on carbon were fixed into idealized positions (riding model) and assigned temperature factors $H_{iso}(H) = 1.2\ U_{eq}$(pivot atom) or 1.5 U_{eq} for methyl moiety. The hydrogen atoms in –OH and >N-H moieties were found on difference Fourier map and refined under rigid-body assumption with assigned temperature factor $H_{iso}(H) = 1.2\ U_{eq}$(pivot atoms)). The determination of absolute structure was based on anomalous dispersion of oxygen, nitrogen and sulfur atoms. Absolute structure parameter: -0.04(4).[10]

The crystallization of **53** did not supply good quality of single crystals. They are thin plates as in the direction [1 0 0] only moieties of $-CH_3$ and -F could be involved in molecular packing (See Fig. S3 in SI). Inefficiency of such stacking is witnessed by the orientation of longest axes of displacive ellipsoids in one direction (See Fig. S4). Furthermore, the precision of structural parameters is hampered also by non-merohedral twinning. The twin matrix being: -1 0 -0.367; 0 -1 0; 0 0 1, with the ratio of mosaic block 0.542:0.458. Nevertheless, the resulting absolute structure is in the agreement with known chirality of atoms of the ring.

Crystal data for **53**, $C_{10}H_{15}F_2NO_4S$, $M_r = 283.29$; Monoclinic, $P2_1$ (No 4), $a = 9.9454$ (9) Å, $b = 12.7005$ (11) Å, $c = 10.2351$ (9) Å, $\beta = 101.048$ (4)° , $V = 1268.85$ (19) Å^3, Z = 4. The unit cell contains two symmetrical molecules differing in the orientation of -F atom (See Fig. S5), $D_x = 1.483$ Mg m^{-3}, temperature of sample 100(2) K, colourless plate of dimensions 0.49 × 0.31 × 0.08 mm, multi-scan absorption correction (μ = 0.29 mm^{-1}), $T_{min} = 0.874$, $T_{max} = 0.979$; a total of 59665 measured reflections ($\theta_{max} = 28.3$), from which 6352 were unique ($R_{int} = 0.067$) and 5915 observed according to the $I > 2\sigma(I)$ criterion. The refinement converged ($\Delta/\sigma_{max} < 0.001$) to $R = 0.066$ for observed reflections and $wR(F^2) = 0.172$, $GOF = 1.06$ for 331 parameters and all 6352 reflections. The final difference map displayed no peaks of chemical significance ($\Delta\rho_{max} = 0.80$, $\Delta\rho_{min}$ -0.81 e.Å^{-3}). The unit cell contains two symmetrically independent molecules differing in the orientation of -F atom (See Fig. S5)

X-ray crystallographic data of **53** have been deposited with the Cambridge Crystallographic Data Centre under deposition number CCDC 2513915 and can be obtained free of charge from the Centre via its website (https://www.ccdc.cam.ac.uk/structures/).

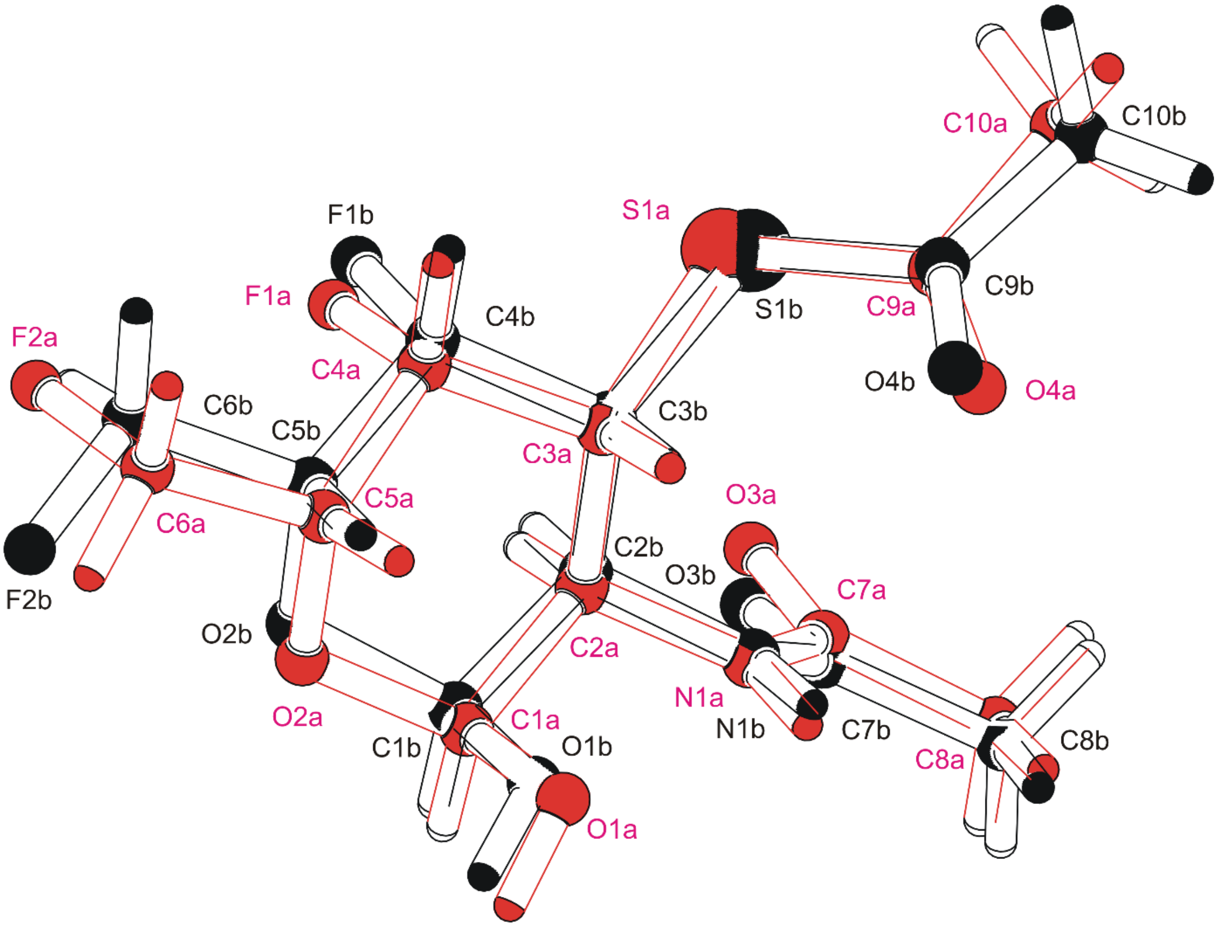

Figure S3. Results of the fit of molecule **a** on molecule **b** of compound **53**. Two molecules are differing mainly in the orientation of -CH_2-F moiety.

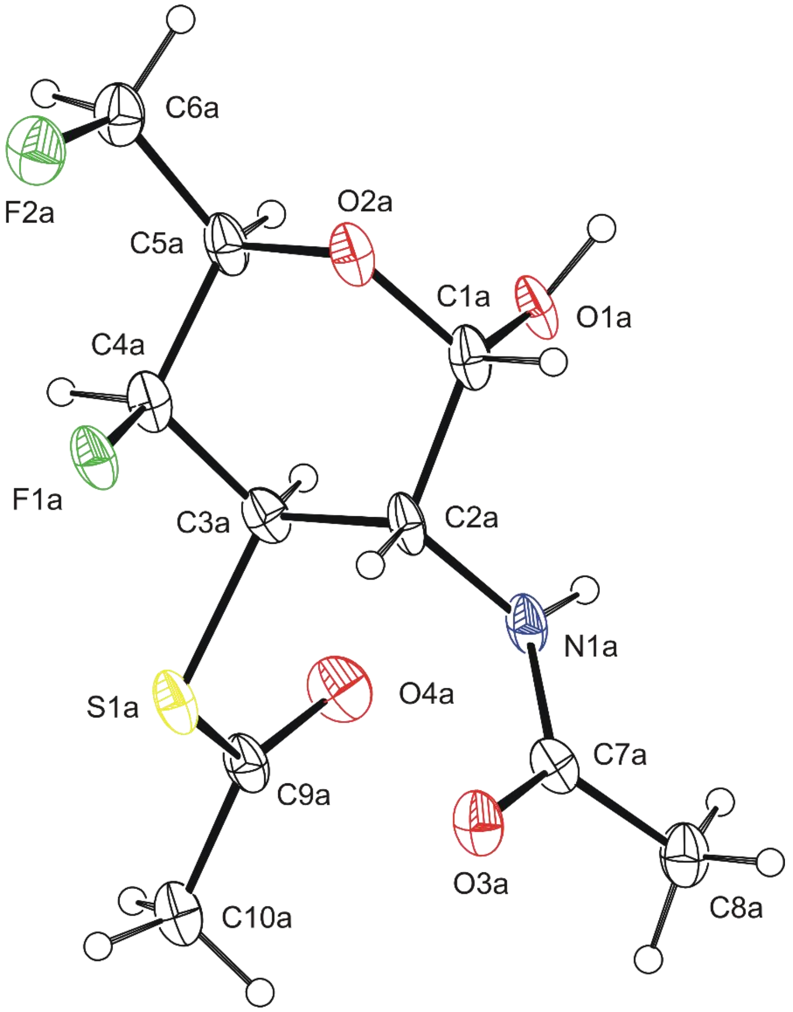

Figure S4. View on the one of symmetrically independent molecule of **53** with atom numbering schema displaying. Displacement ellipsoids are drawn on 30% probability level.

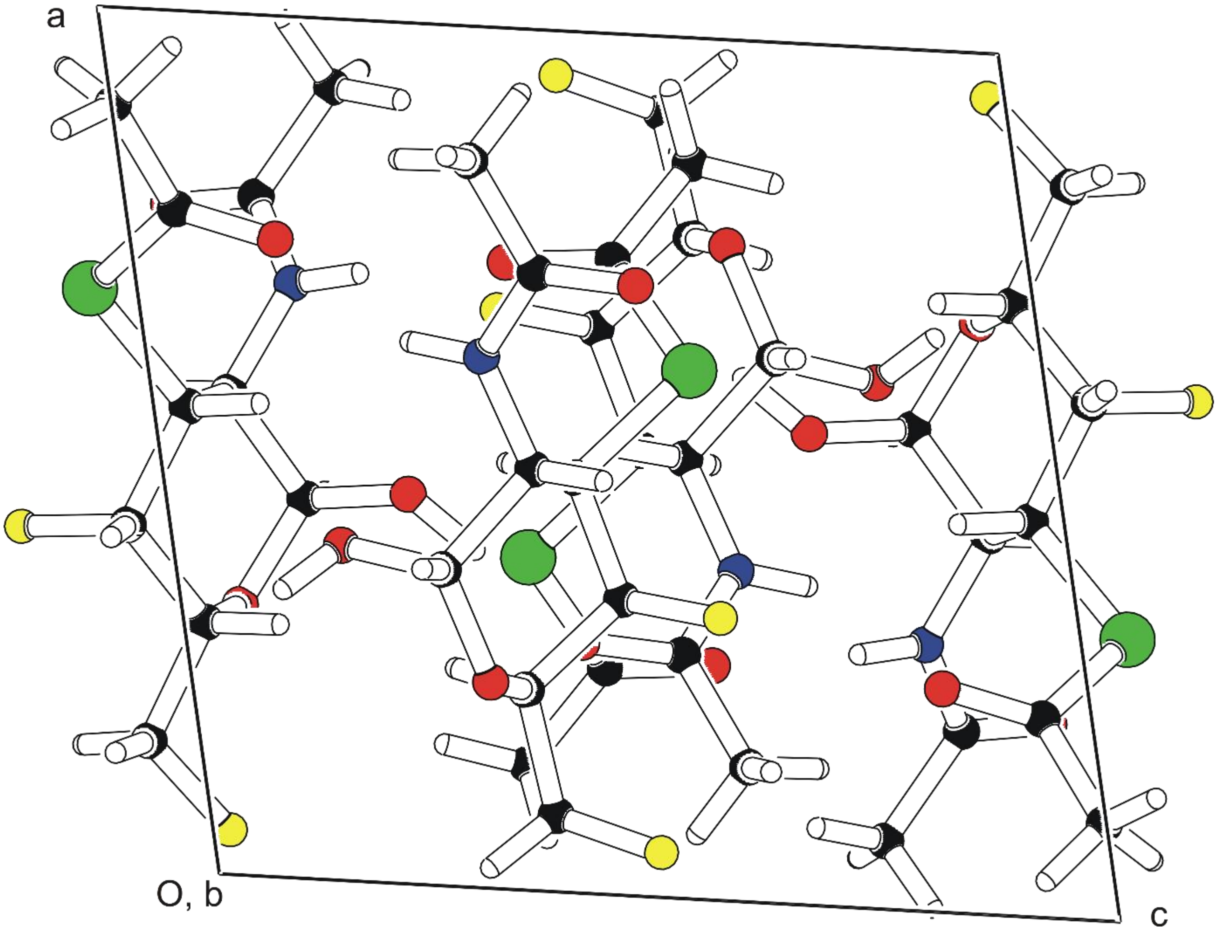

Figure S5. View on the unit cell of compound **53** along b axis.

E. References:

(1) Aich, U.; Campbell, C. T.; Elmouelhi, N.; Weier, C. A.; Sampathkumar, S. G.; Choi, S. S.; Yarema, K. J. Regioisomeric SCFA Attachment to Hexosamines Separates Metabolic Flux from Cytotoxicity and MUC1 Suppression. *ACS Chem. Biol.* **2008**, *3* (4), 230–240. https://doi.org/10.1021/cb7002708

(2) Guthrie, R.; Murphy, D. 1009. Nitrogen-containing carbohydrate derivatives. Part IV. Some azido-and epimino-sugars. *J. Chem. Soc. (Resumed)* **1963**, 5288–5294. http://dx.doi.org/10.1039/JR9630005288

(3) Krist, P.; Kuzma, M.; Pelyvás, I. F.; Simerská, P.; Křen, V. Synthesis of 4-nitrophenyl 2-acetamido-2-deoxy-β-D-mannopyranoside and 4-nitrophenyl 2-acetamido-2-deoxy-α-D-mannopyranoside. *Collect. Czech. Chem. Commun.* **2003**, *68* (4), 801–811. https://doi.org/10.1135/cccc20030801

(4) Laine, D.; Denavit, V.; Giguere, D. Synthesis of Protected 3-Deoxy-3-fluoro-and 4-Deoxy-4-fluoro-d-galactopyranosides from Levoglucosan. *J. Org. Chem.* **2017**, *82* (9), 4986–4992. https://doi.org/10.1021/acs.joc.7b00543

(5) Hartlieb, S.; Günzel, A.; Gerardy-Schahn, R.; Münster-Kühnel, A. K.; Kirschning, A.; Dräger, G. Chemoenzymatic synthesis of CMP-N-acetyl-7-fluoro-7-deoxy-neuraminic acid. *Carbohydr. Res.* **2008**, *343* (12), 2075–2082. https://doi.org/10.1016/j.carres.2008.02.003
(6) Sugawara, T.; Igarashi, K. Synthesis of a trisaccharide component of the capsular polysaccharide of Streptococcus pneumoniae type 19F. *Carbohydr. Res.* **1988**, *172* (2), 195–207. https://doi.org/10.1016/S0008-6215(00)90854-1
(7) Fontenelle, C. Q.; Shishmarev, D.; Kuchel, P. W.; Linclau, B. The Synthesis of 3,4-dideoxy-3,4-difluoro-D-glucose. *Trends Carbohydr. Res.* **2017**, *9* (1), 28–33.
(8) St-Gelais, J.; Bouchard, M.; Denavit, V.; Giguère, D. Synthesis and Lipophilicity of Trifluorinated Analogues of Glucose. *J. Org. Chem.* **2019**, *84* (13), 8509–8522. https://doi.org/10.1021/acs.joc.9b00795
(9) Sheldrick, G.M. (2015). SHELXT – Integrated space-group and crystal-structure determination. *Acta Cryst. A*, **2015**, *71* 3–8. https://doi.org/10.1107/S2053273314026370
(10) Parsons, S., Flack, H.D. and Wagner, T., Use of intensity quotients and differences in absolute structure refinement *Acta Cryst. B*, **2013**, *69,* 249–259. https://doi.org/10.1107/S2052519213010014
