# Supplementary material for: Synthesis and Antiproliferative Activity of Fluorinated N‑Acetylmannosamine Analogs
Source: J Org Chem. 2026 Mar 19;91(13):4645–56. doi: 10.1021/acs.joc.5c03084 (PMC13054866; doi:10.1021/acs.joc.5c03084)

Synthesis and Antiproliferative Activity of Fluorinated *N*-Acetylmannosamine Analogs

Aleš Krčil,[a,b] Lucia Šutvajová,[c,d] Vojtěch Hamala,[a] Ivana Císařová,[e] Martin Kurfiřt,[a,b] Lucie Červenková Šťastná,[a] Jana Bernášková,[a] Roman Hrstka,[*,d] Jindřich Karban,[*,a]

[a] *Institute of Chemical Process Fundamentals of the CAS, v. v. i., Rozvojová 1/135, 165 00 Praha, Czech Republic. E-mail: karban@icpf.cas.cz.*
[b] *Department of Organic Chemistry, University of Chemistry and Technology, Technická 5, 166 28 Praha, Czech Republic.*
[c] *Department of Experimental Biology, Faculty of Science, Masaryk University, Kotlářská 2, 611 37, Brno, Czech Republic.*
[d] *Research Centre for Applied Molecular Oncology, Masaryk Memorial Cancer Institute, Žlutý kopec 7, 656 53 Brno, Czech Republic.*
[e] *Department of Inorganic Chemistry, Faculty of Science, Charles University, Hlavova 8, CZ-128 43 Praha 2, Czech Republic*
[*]E-mail: roman.hrstka@mou.cz, karban@icpf.cas.cz

Supporting Information File II

Copies of NMR

Table of contents

NMR COMPOUND **3** S3
NMR COMPOUND **4** S6
NMR COMPOUND **5** S9
NMR COMPOUND **6** S12
NMR COMPOUND **7** S15
NMR COMPOUND **8** S18
NMR COMPOUND **9** S21
NMR COMPOUND **10** S24
NMR COMPOUND **11** S27
NMR COMPOUND **17** (containing ca 10% of compound **18**) S30
NMR COMPOUND **18** S33
NMR COMPOUND **19** S36
NMR COMPOUND **21** S39
NMR COMPOUND **22** S42
NMR COMPOUND α-**23** S45
NMR COMPOUND β-**23** S48
NMR COMPOUND **24** S51

NMR COMPOUND **25**......S54
NMR COMPOUND **26**......S57
NMR COMPOUND **27**......S60
NMR COMPOUND **28**......S63
NMR COMPOUND **29**......S66
NMR COMPOUND **31**......S69
NMR COMPOUND **32**......S72
NMR COMPOUND **33**......S75
NMR COMPOUND **34**......S78
NMR COMPOUND **36**......S81
NMR COMPOUND **37**......S84
NMR COMPOUND **38**......S87
NMR COMPOUND **39**......S90
NMR COMPOUND α-**48**......S93
NMR COMPOUND β-**48**......S96
NMR COMPOUND **49**......S99
NMR COMPOUND **50**......S102
NMR COMPOUND **51**......S105
NMR COMPOUND **53**......S108
NMR COMPOUND **55**......S111
NMR COMPOUND **56**......S114
NMR COMPOUND β-**57**......S117
NMR COMPOUND **58**......S120
NMR COMPOUND **59**......S123
NMR COMPOUND **60**......S126
NMR COMPOUND **61**......S129

NMR COMPOUND 3

^{1}H NMR (400 MHz, CD_3OD) 3

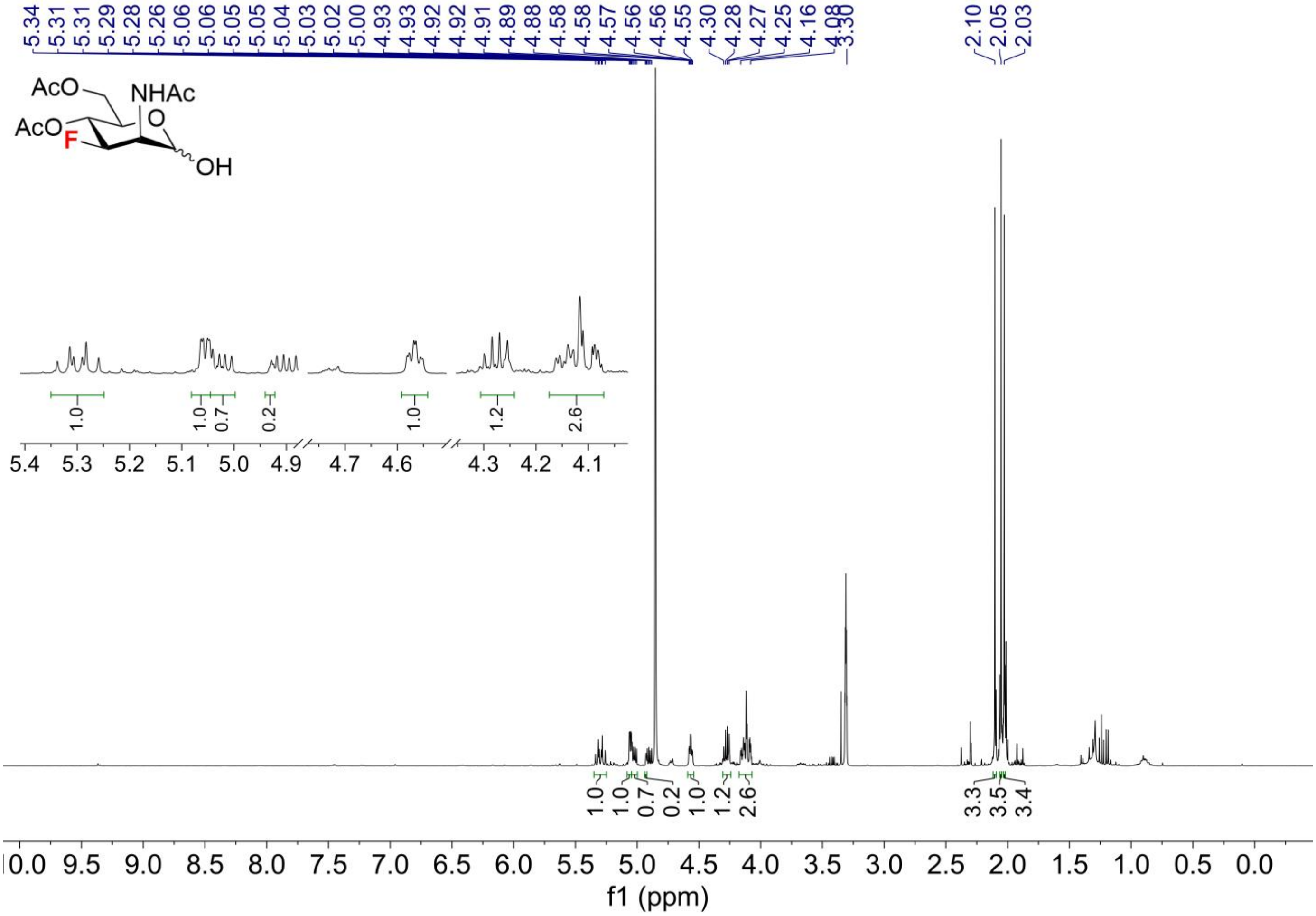

^{13}C{^{1}H} NMR (101 MHz, CD_3OD) 3

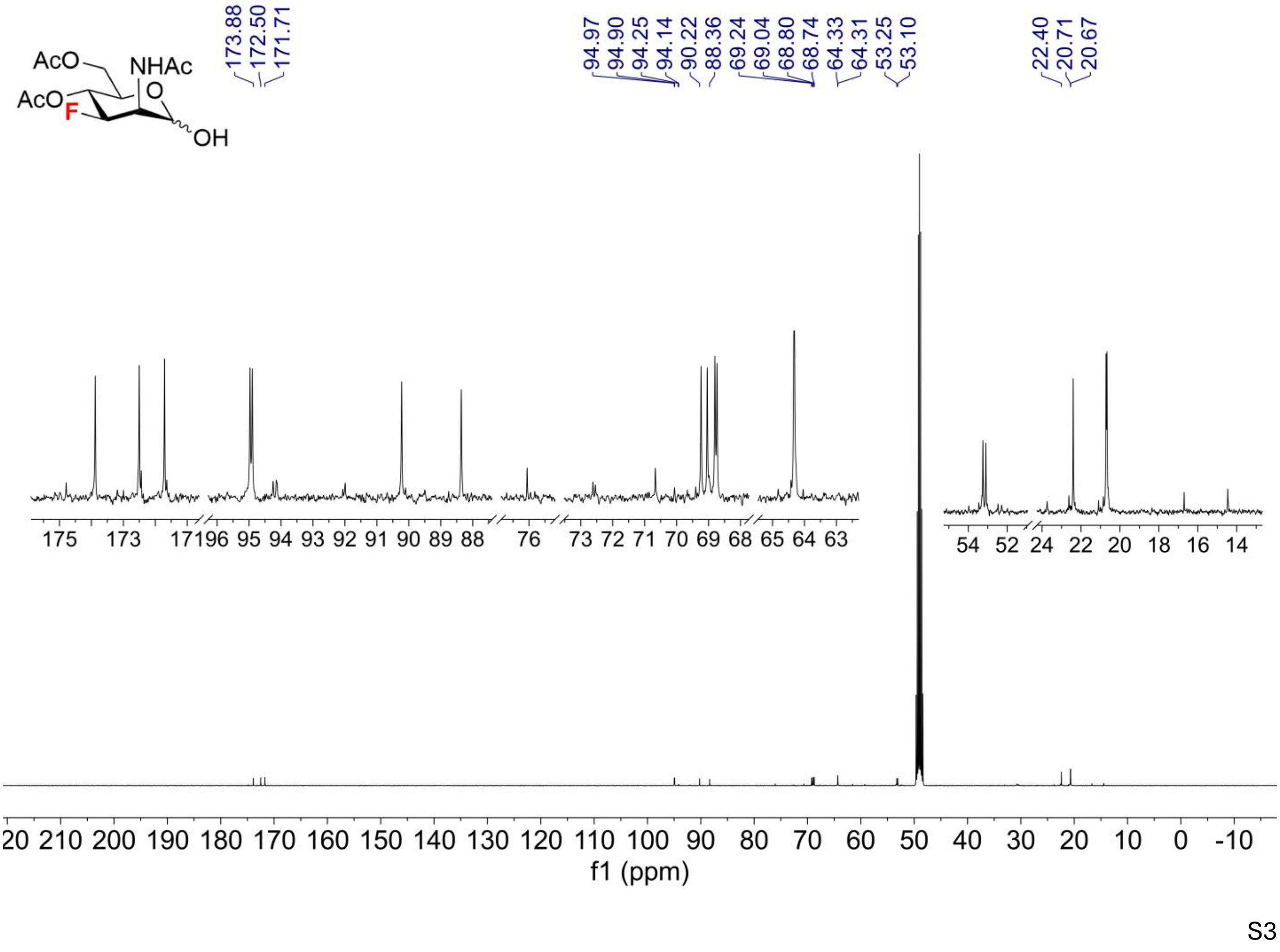

^{19}F NMR (376 MHz, CD_3OD) 3

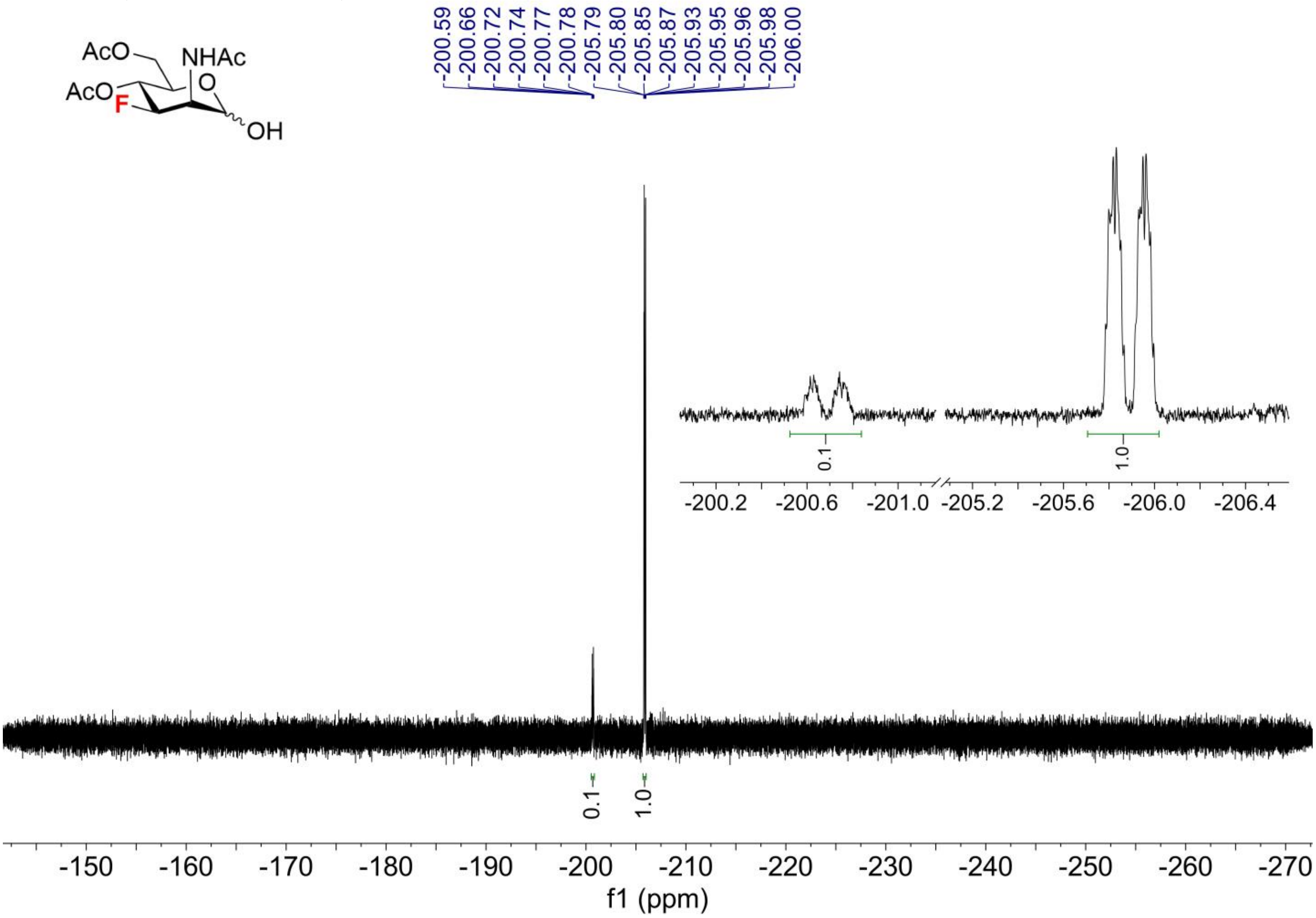

^{1}H-^{1}H COSY (400 MHz, CD_3OD) 3

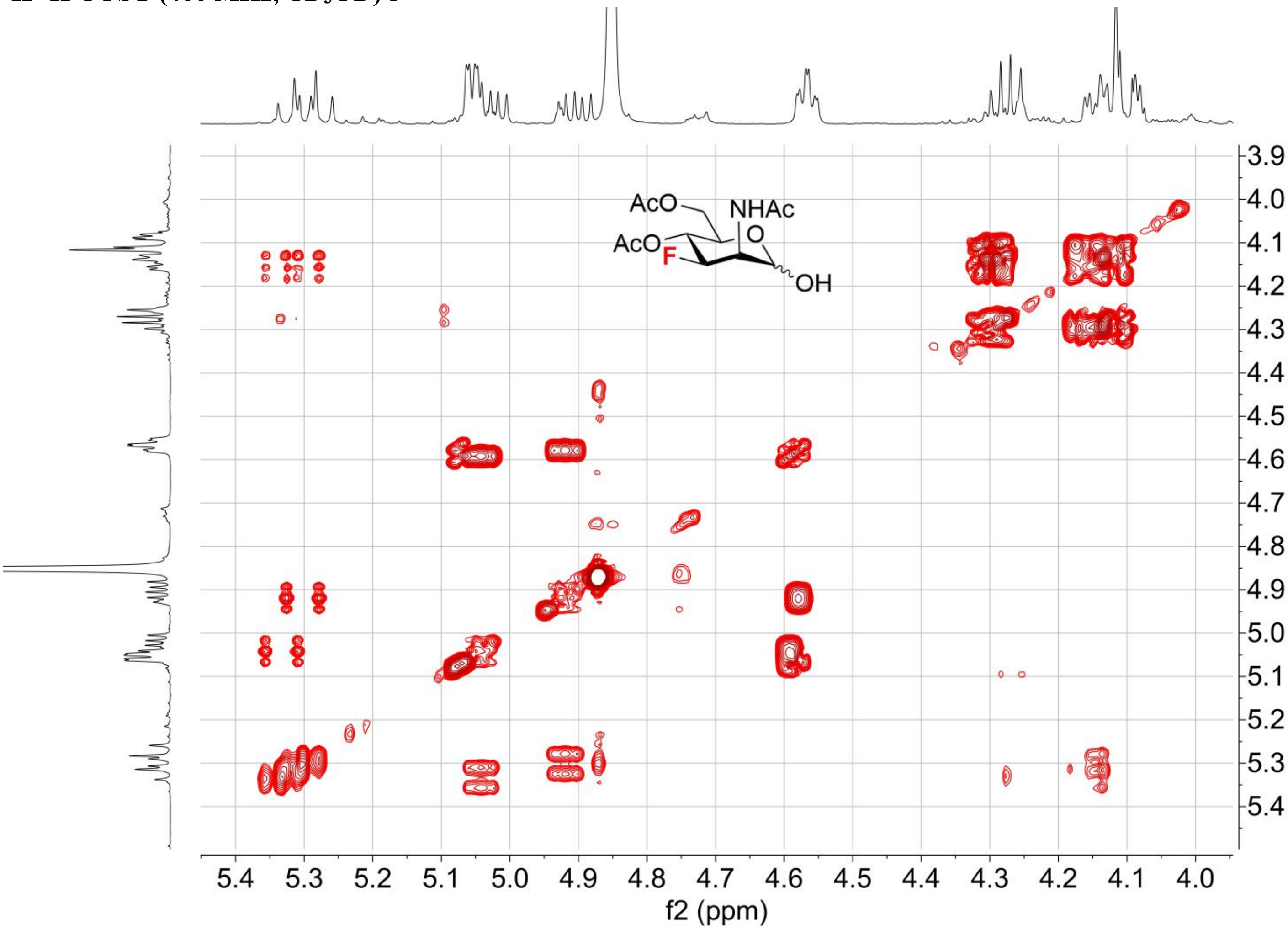

^{1}H-^{13}C HSQC (^{1}H/^{13}C 400/101 MHz, CD_3OD) 3

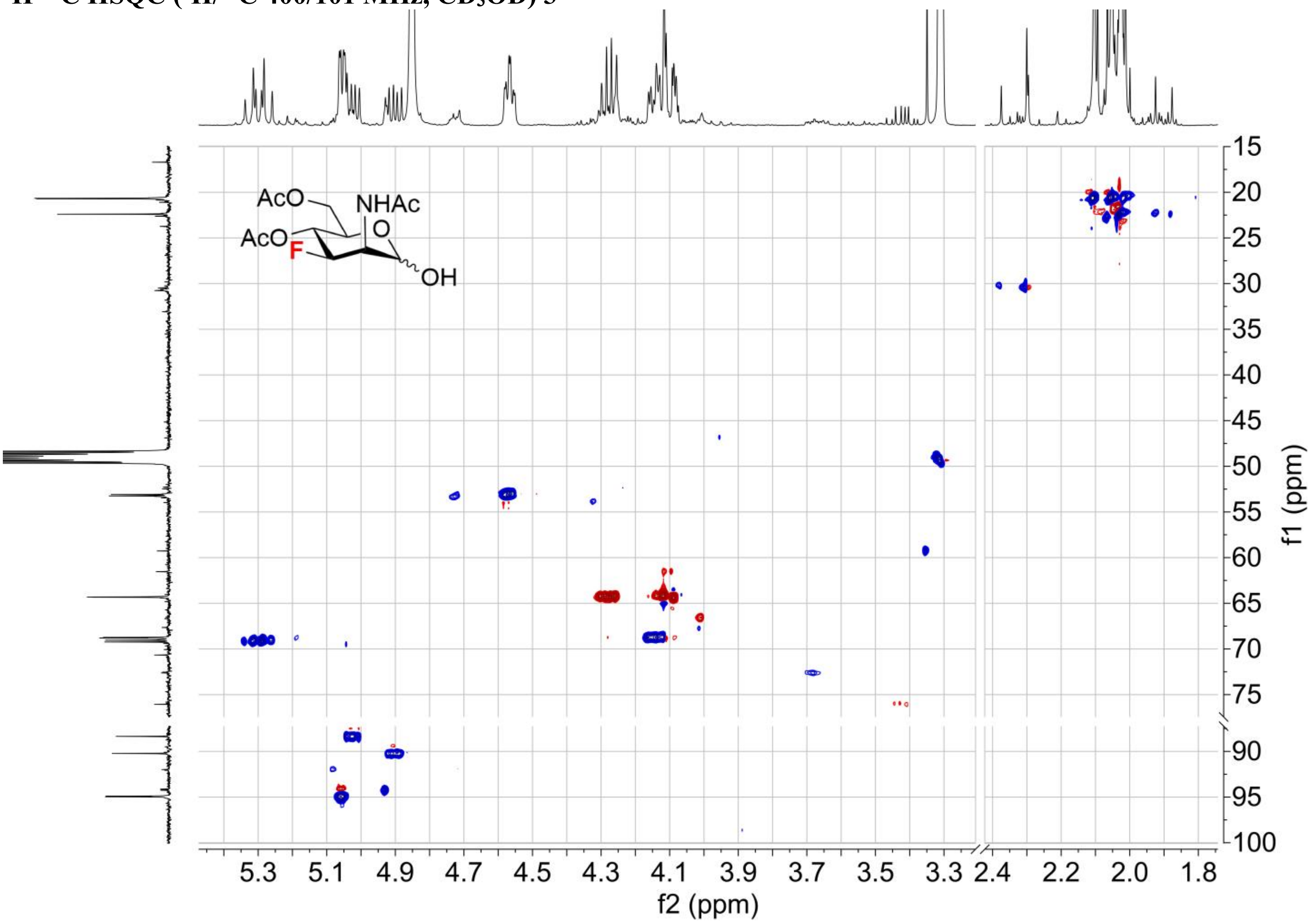

^{1}H-^{13}C HMBC (^{1}H/^{13}C 400/101 MHz, CD_3OD) 3

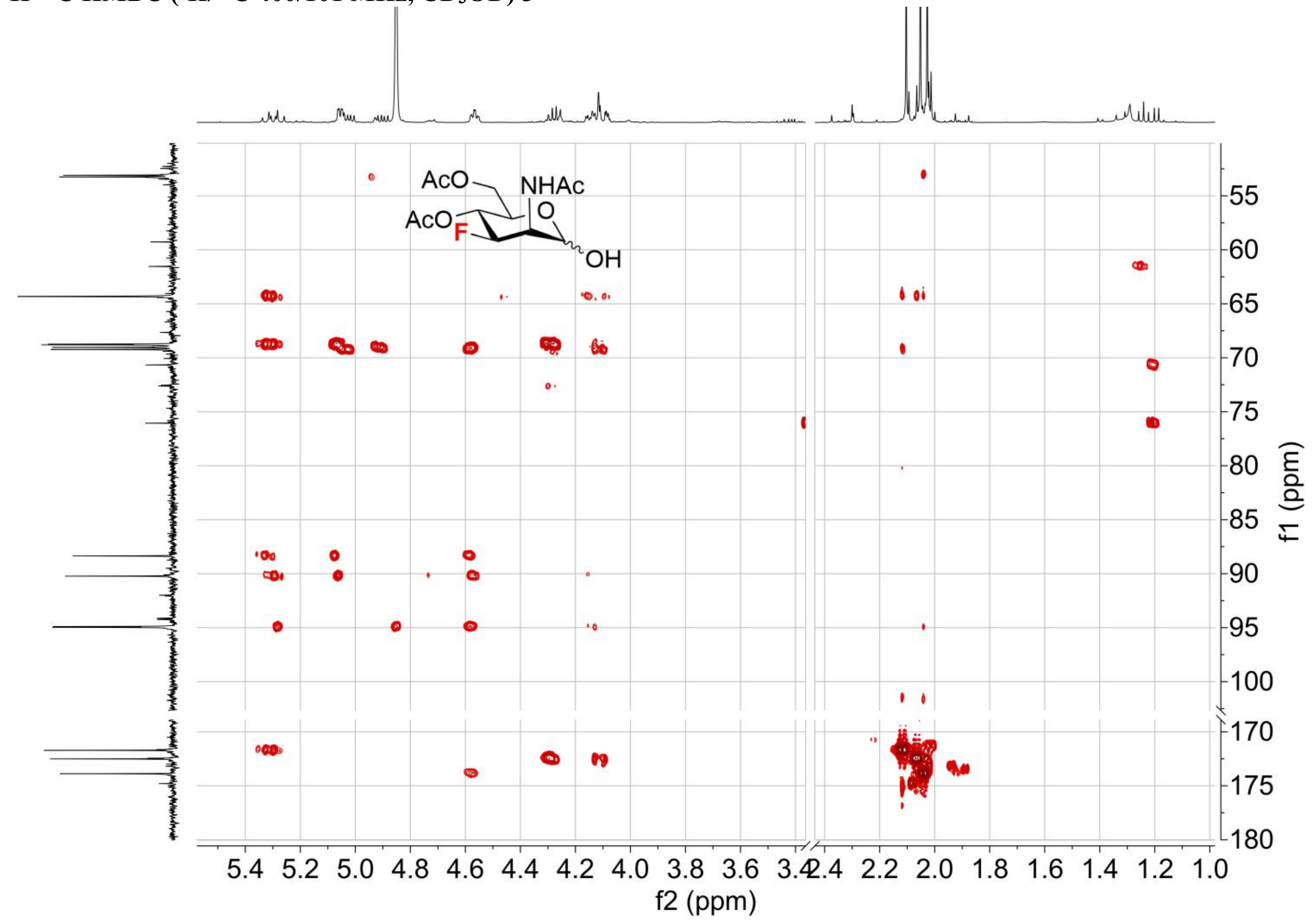

NMR COMPOUND 4

^{1}H NMR (400 MHz, CD_3OD) 4

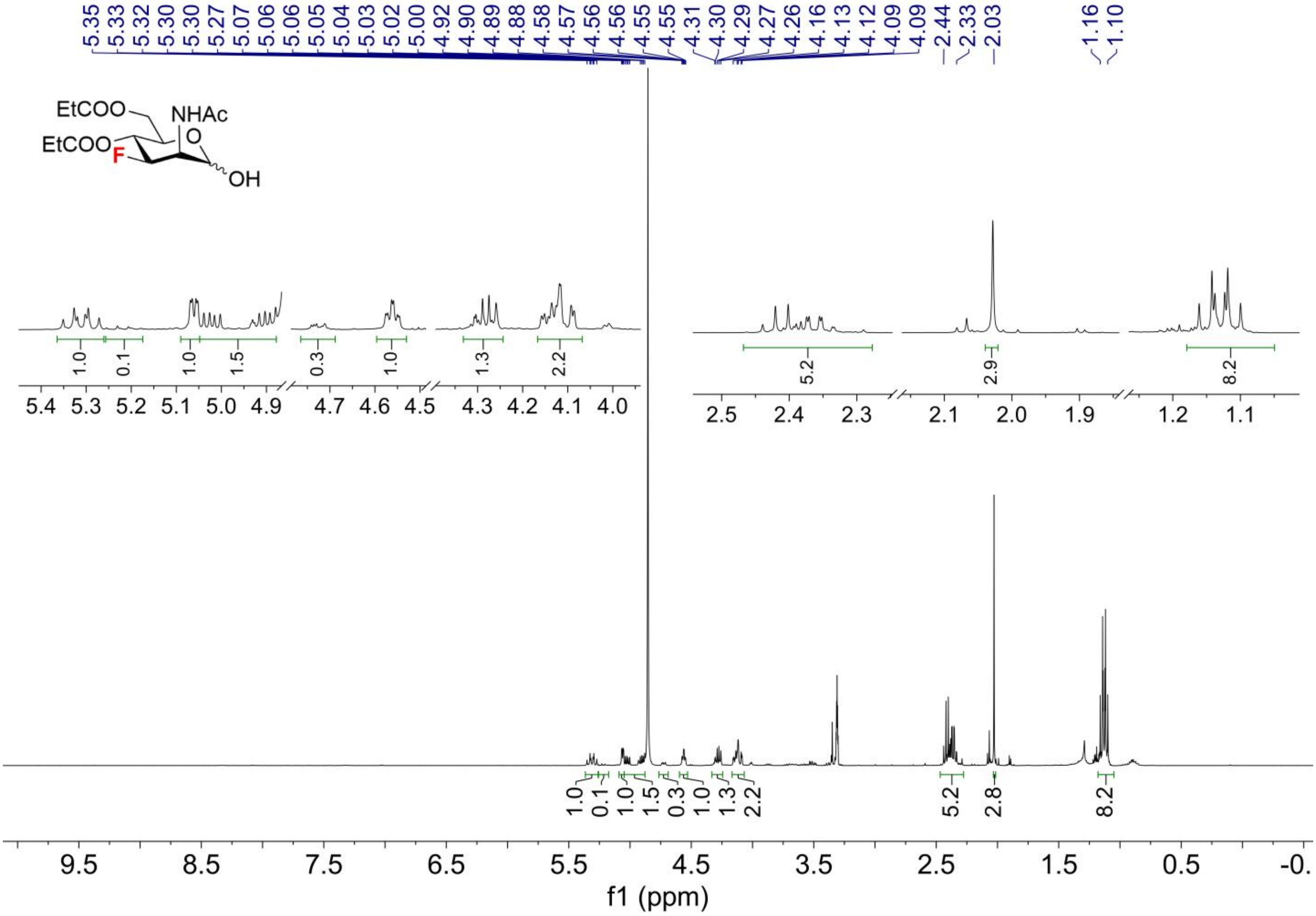

^{13}C{^{1}H} NMR (101 MHz, CD_3OD) 4

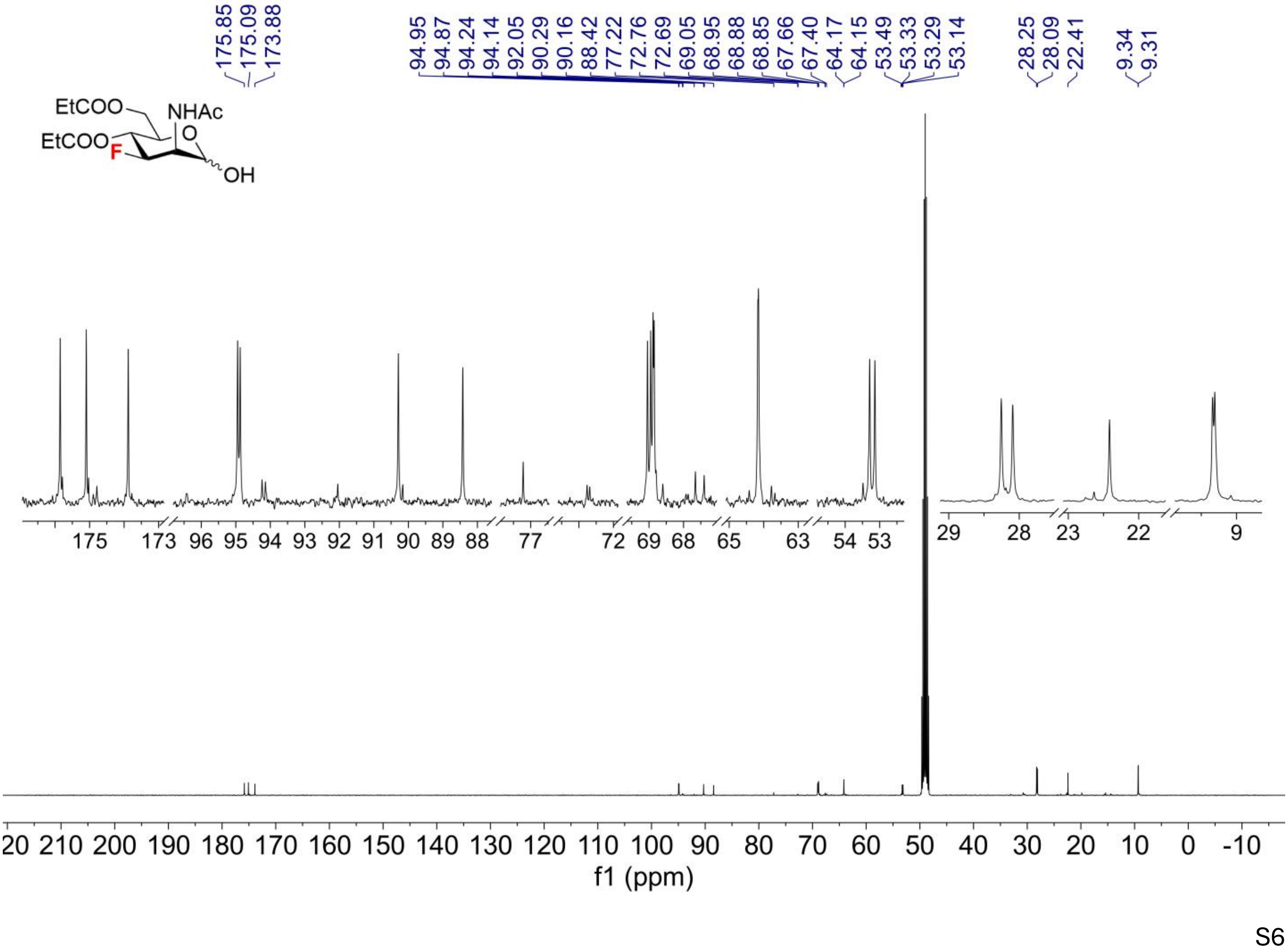

^{19}F NMR (376 MHz, CD_3OD) 4

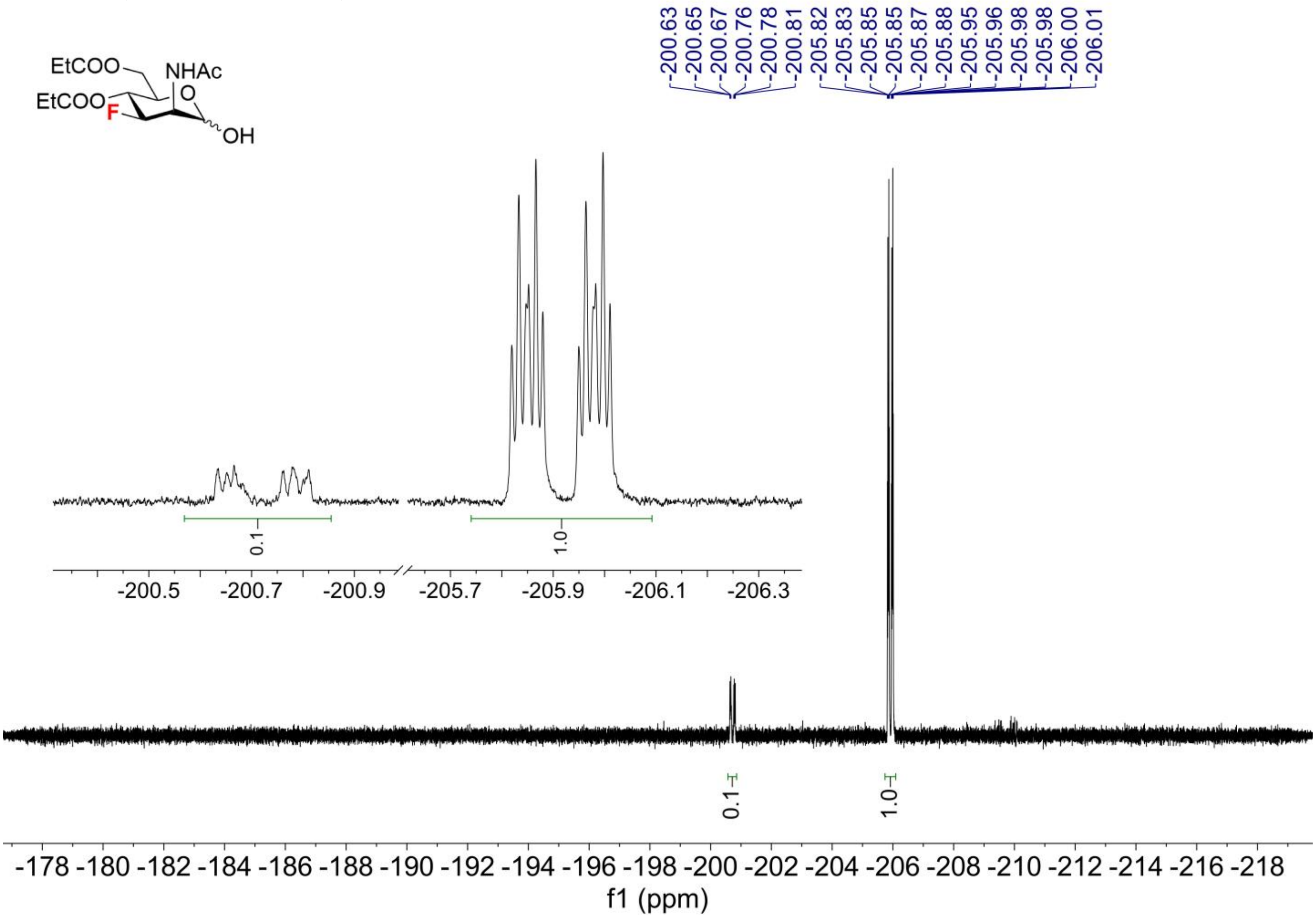

^{1}H-^{1}H COSY (400 MHz, CD_3OD) 4

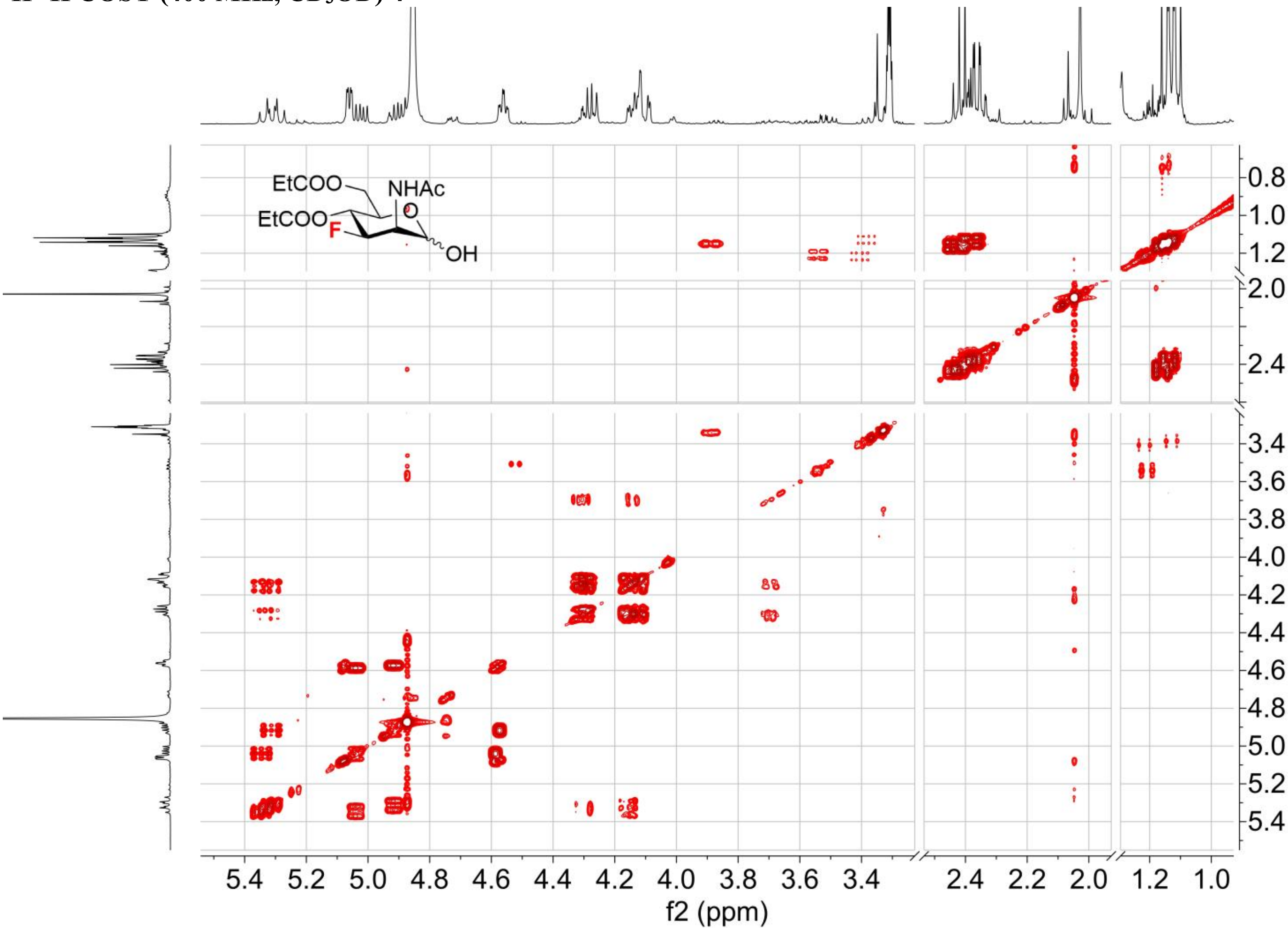

^{1}H-^{13}C HSQC ($^{1}H/^{13}C$ 400/101 MHz, CD_3OD) 4

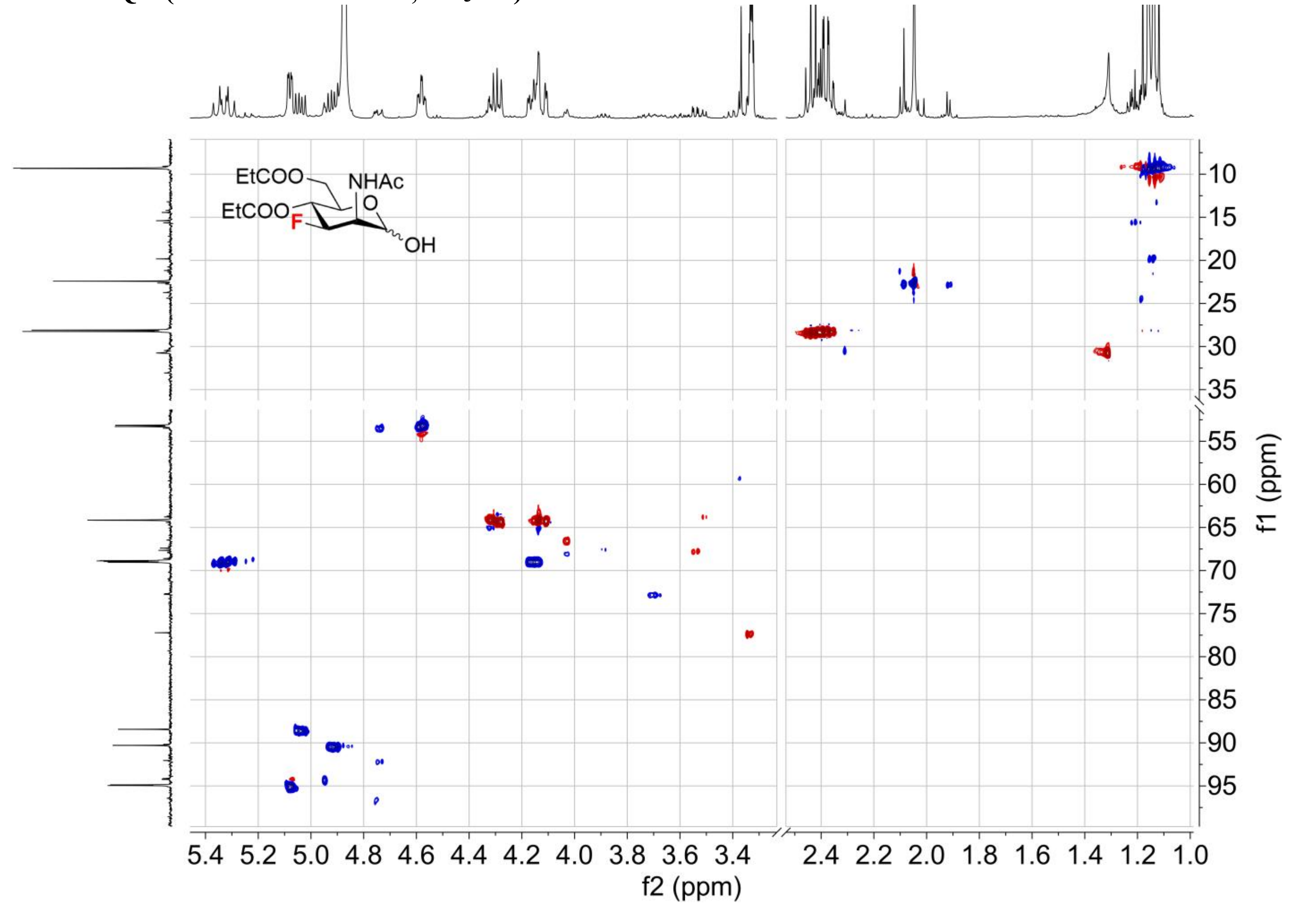

^{1}H-^{13}C HMBC ($^{1}H/^{13}C$ 400/101 MHz, CD_3OD) 4

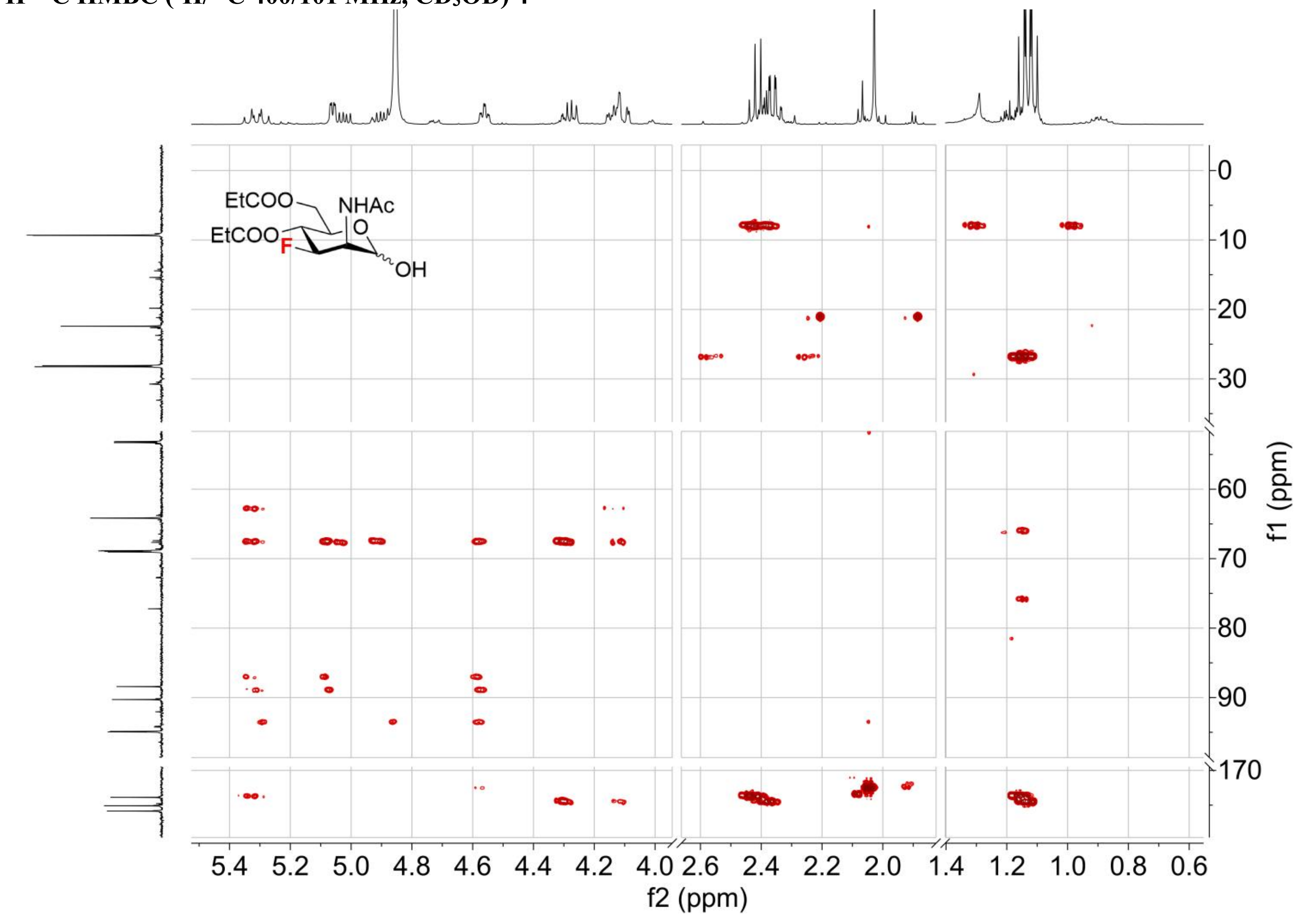

NMR COMPOUND 5

^{1}H NMR (400 MHz, CDCl$_3$) 5

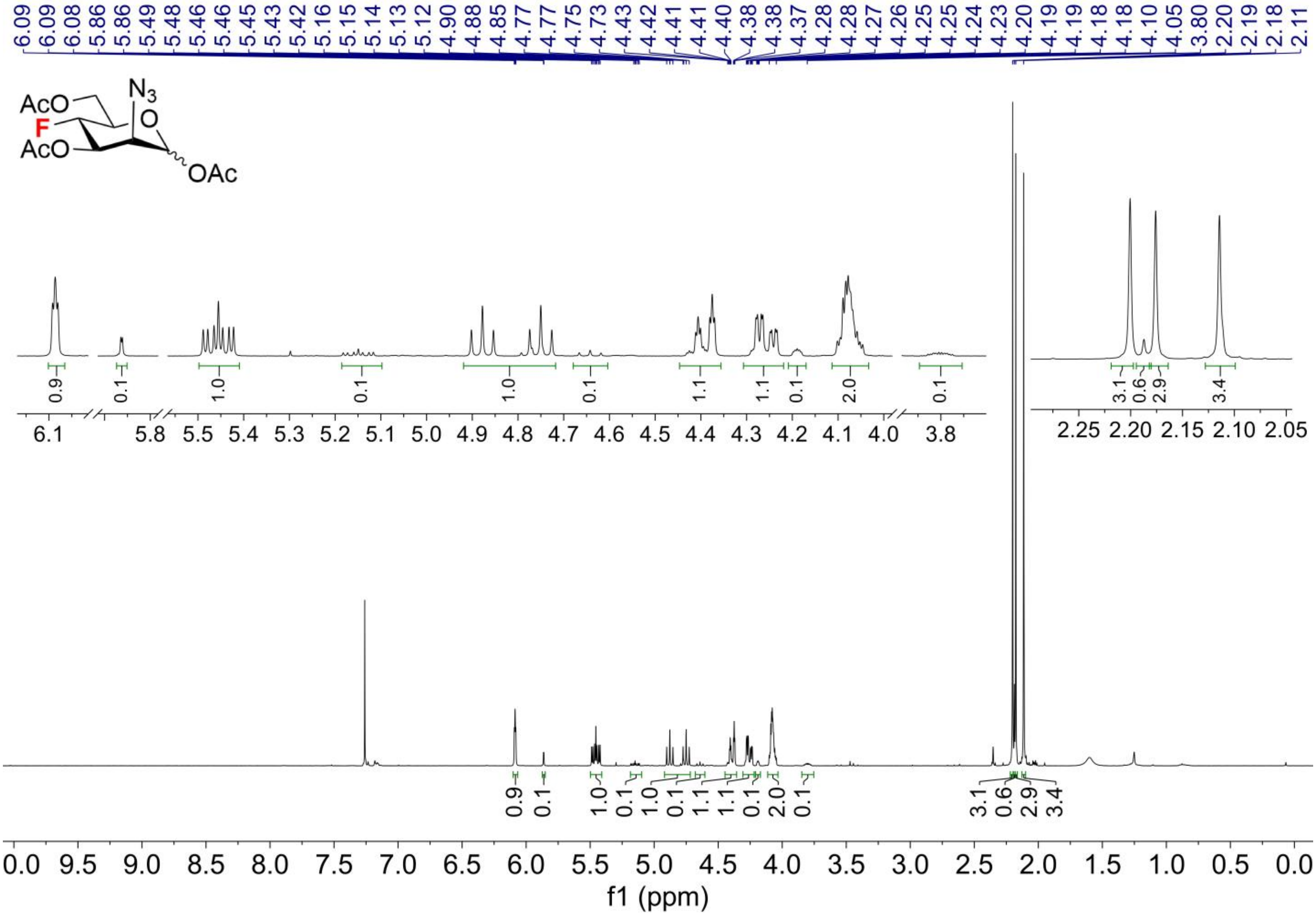

^{13}C{^{1}H} NMR (101 MHz, CDCl$_3$) 5

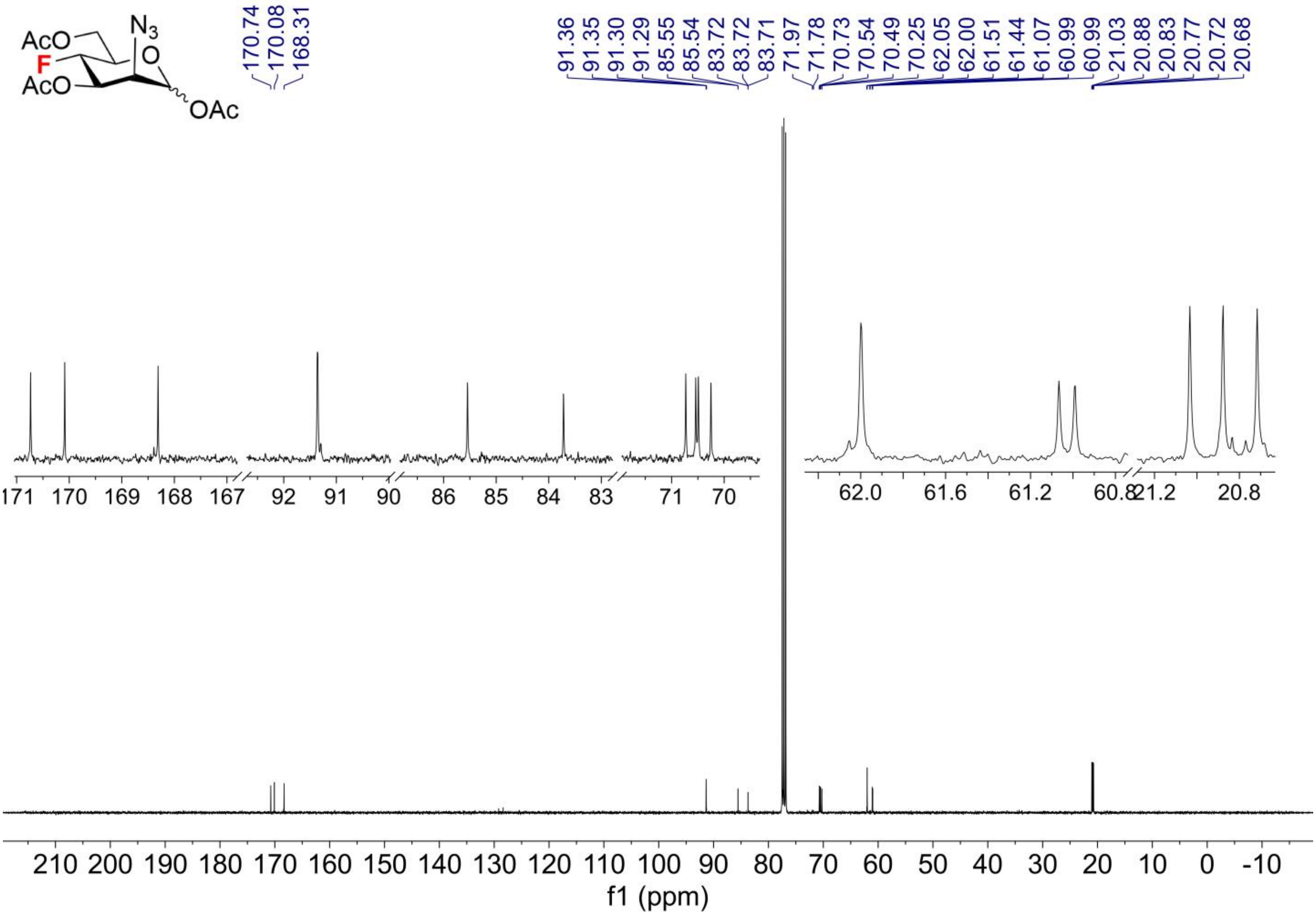

^{19}F NMR (376 MHz, CDCl$_3$) 5

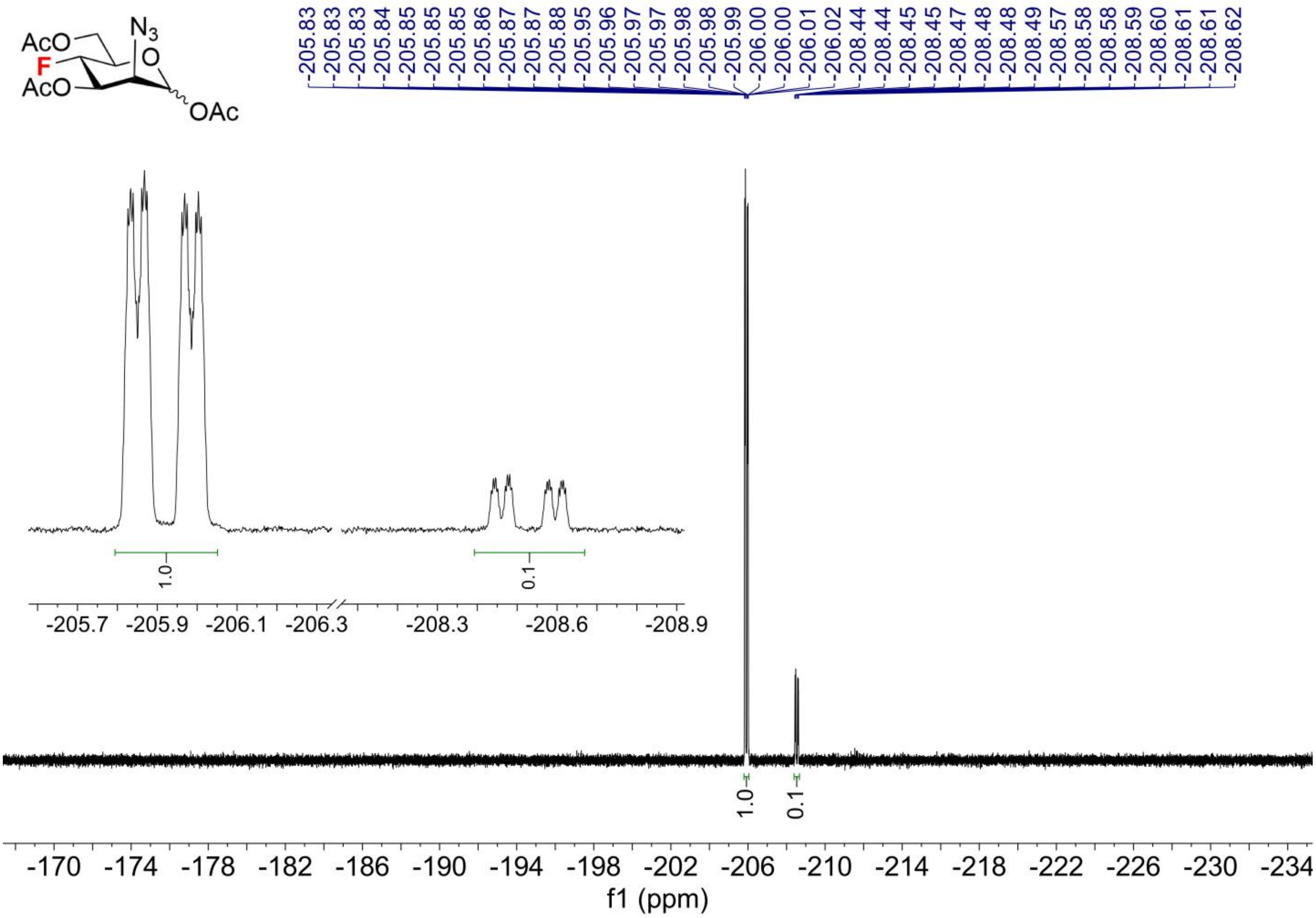

^{1}H-^{1}H COSY (400 MHz, CDCl$_3$) 5

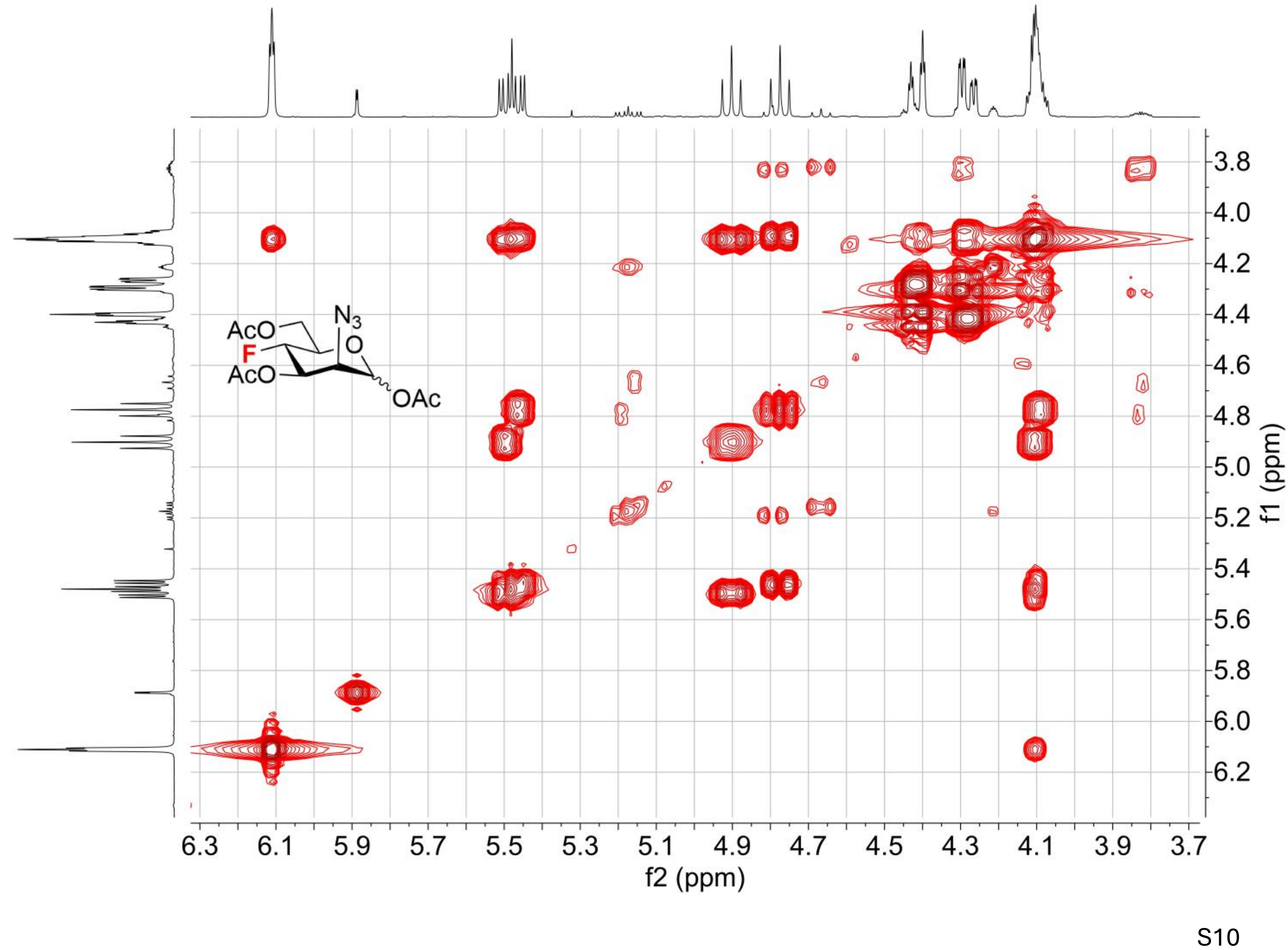

^{1}H-^{13}C HSQC (^{1}H/^{13}C 400/101 MHz, CDCl$_3$) 5

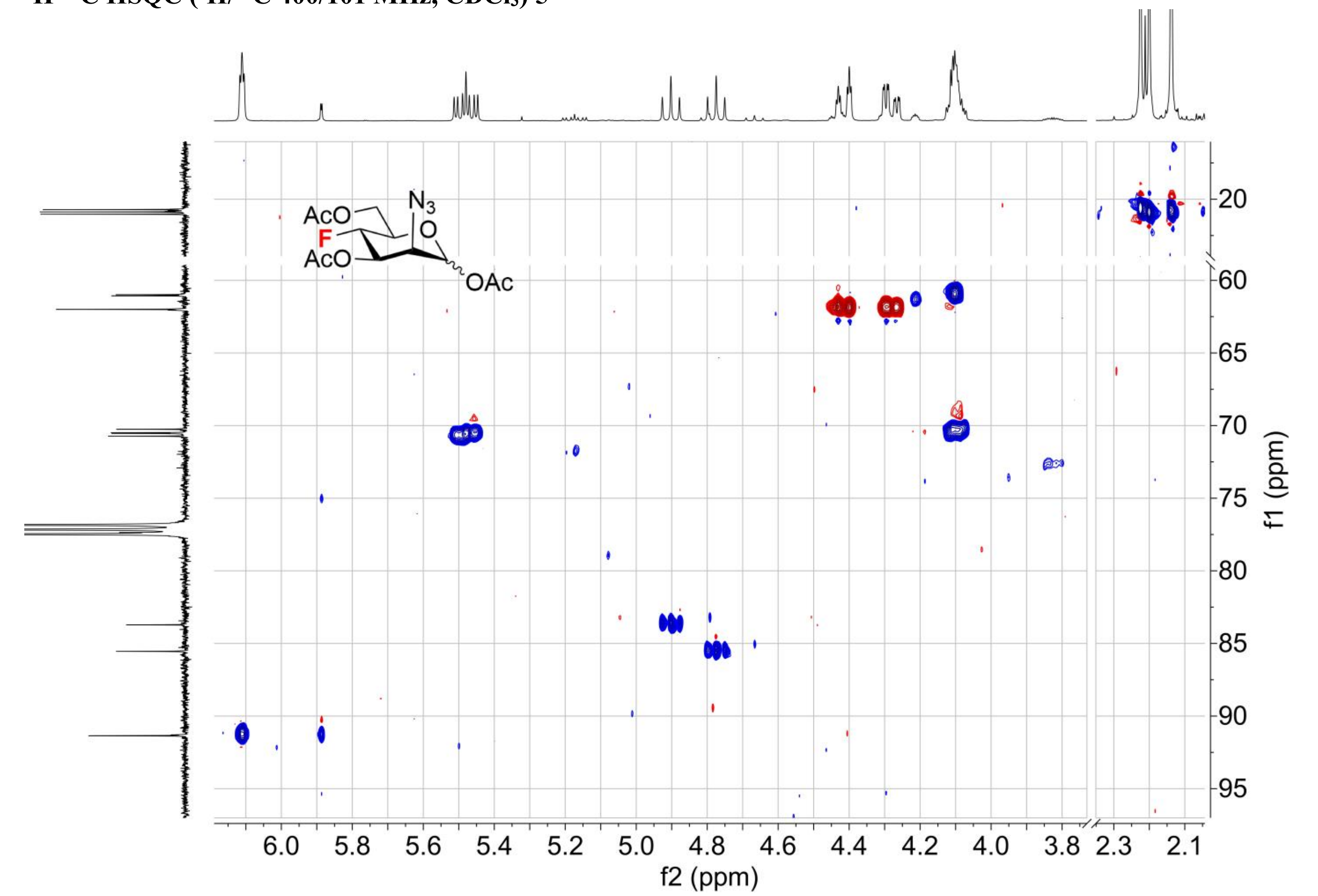

^{1}H-^{13}C HMBC (^{1}H/^{13}C 400/101 MHz, CDCl$_3$) 5

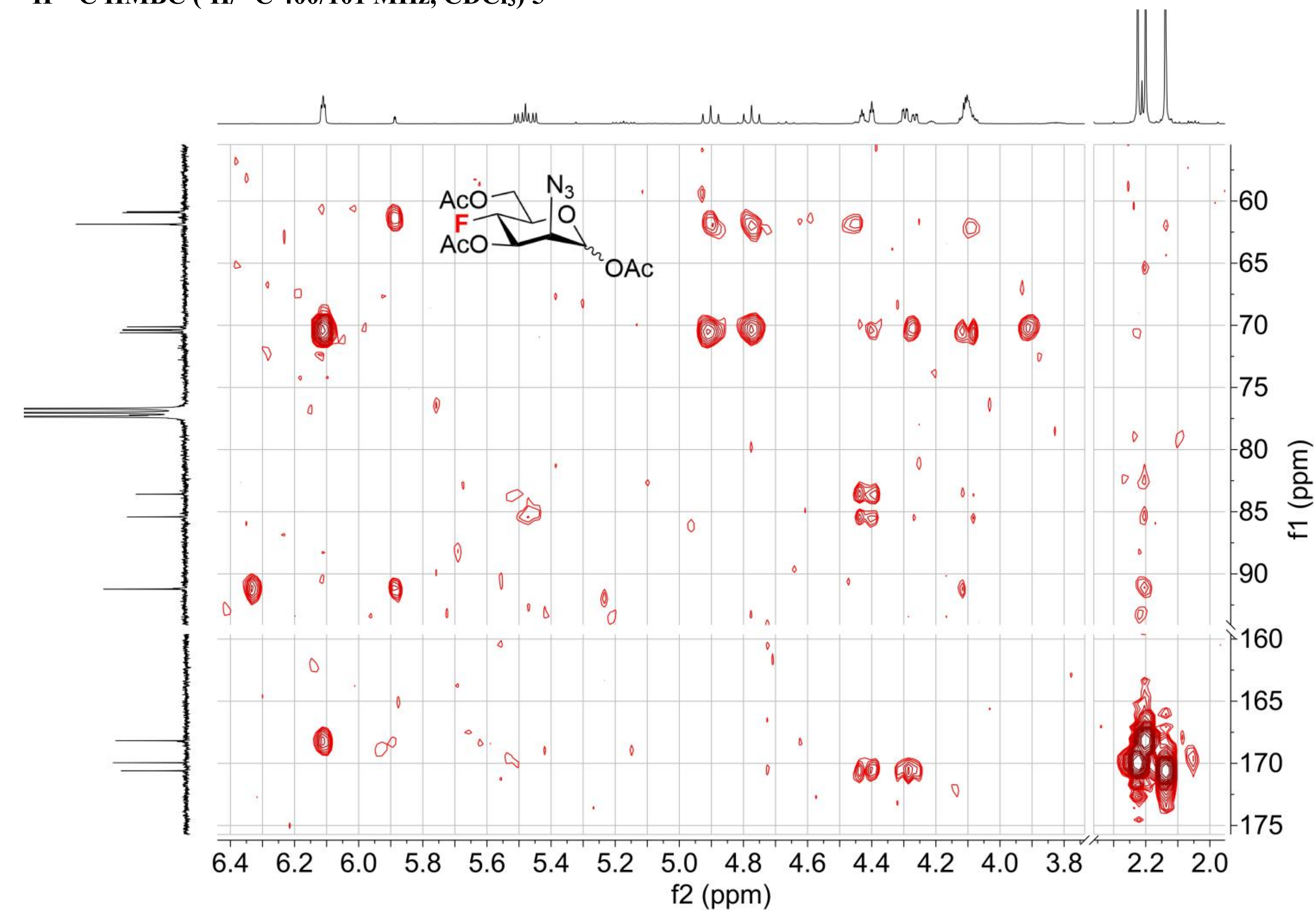

NMR COMPOUND 6

^{1}H NMR (400 MHz, CD_3OD) 6

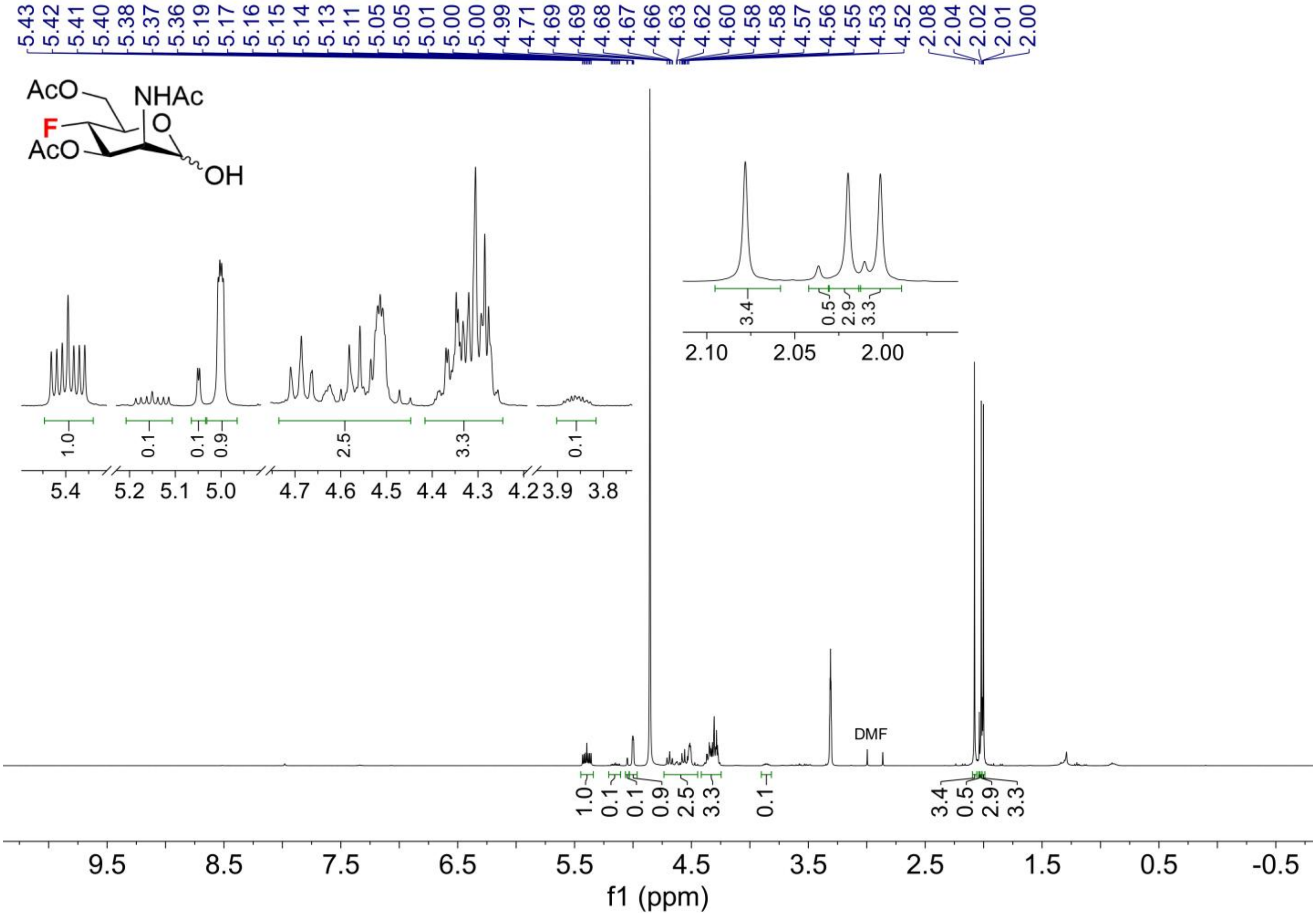

$^{13}C\{^{1}H\}$ NMR (101 MHz, CD_3OD) 6

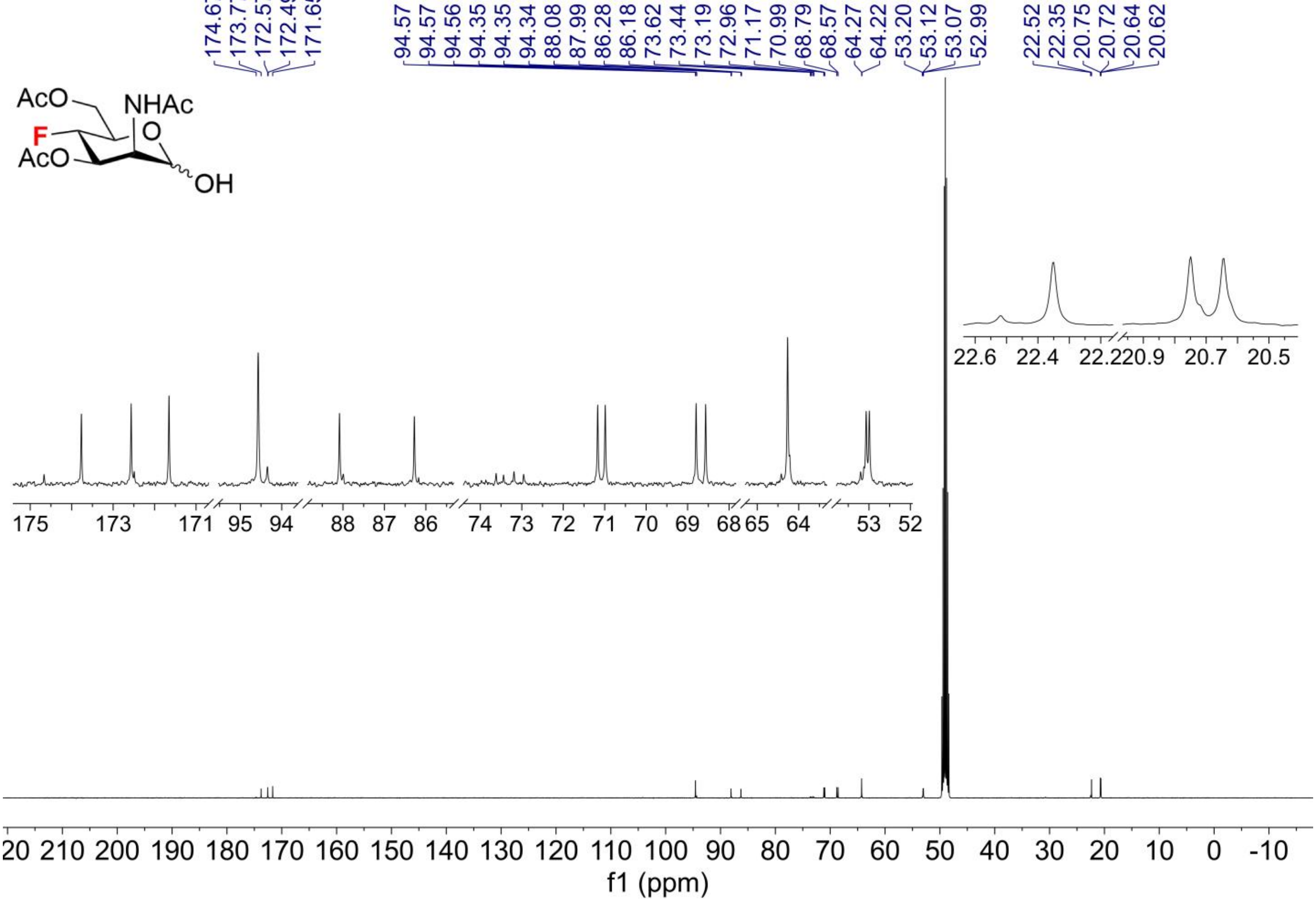

^{19}F NMR (376 MHz, CD_3OD) 6

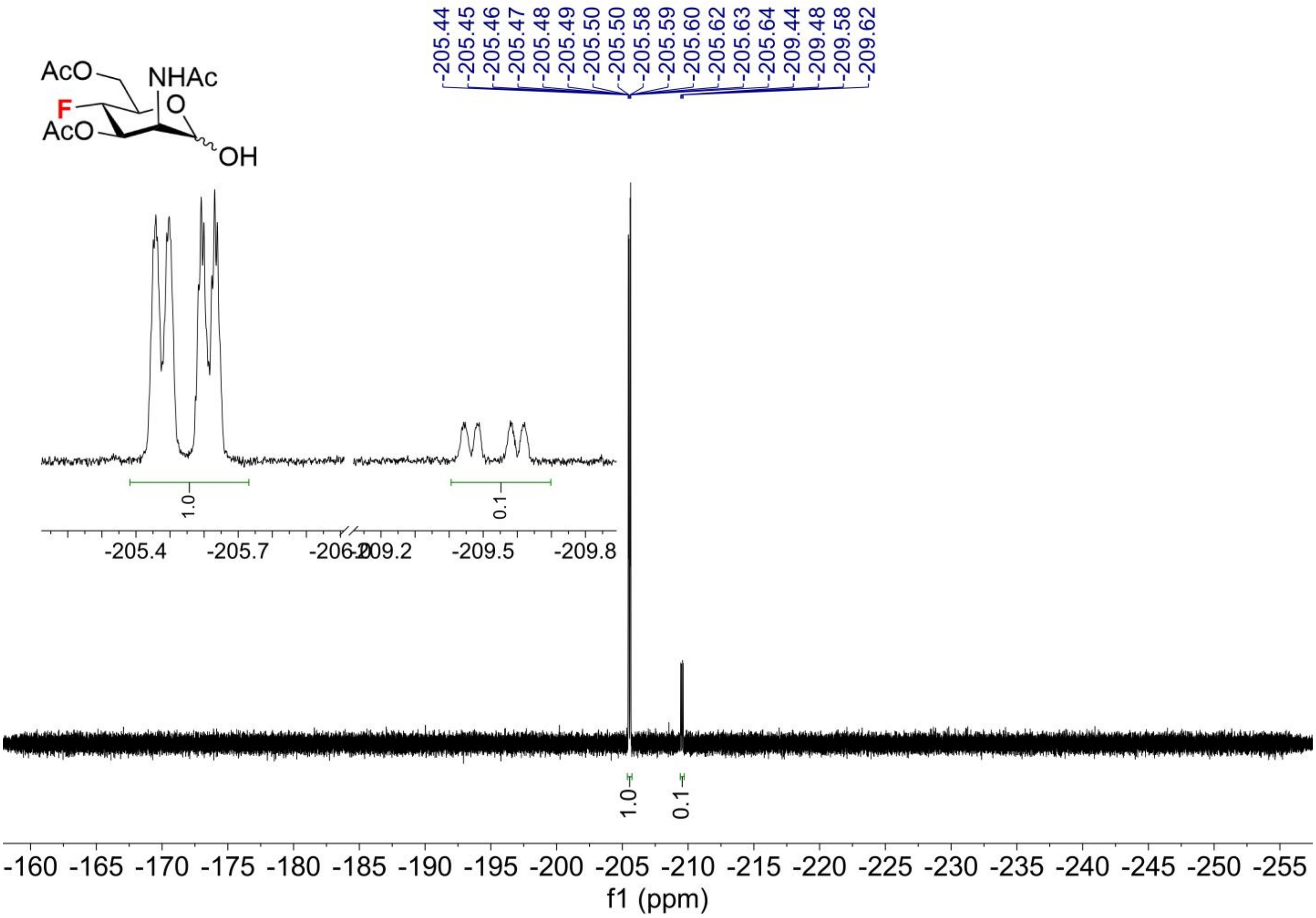

^{1}H-^{1}H COSY (400 MHz, CD_3OD) 6

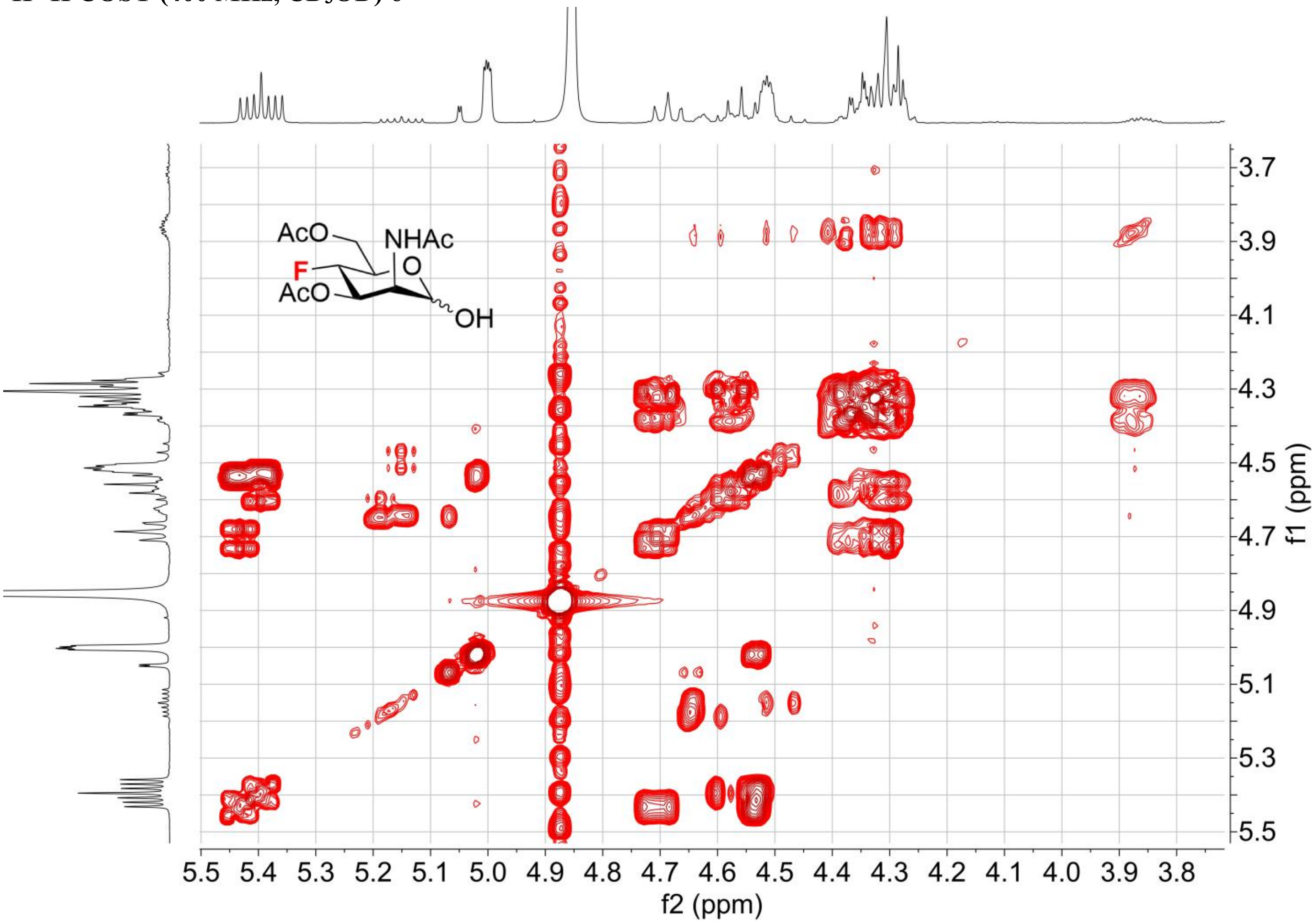

^1H-^{13}C HSQC ($^1H/^{13}C$ 400/101 MHz, CD_3OD) 6

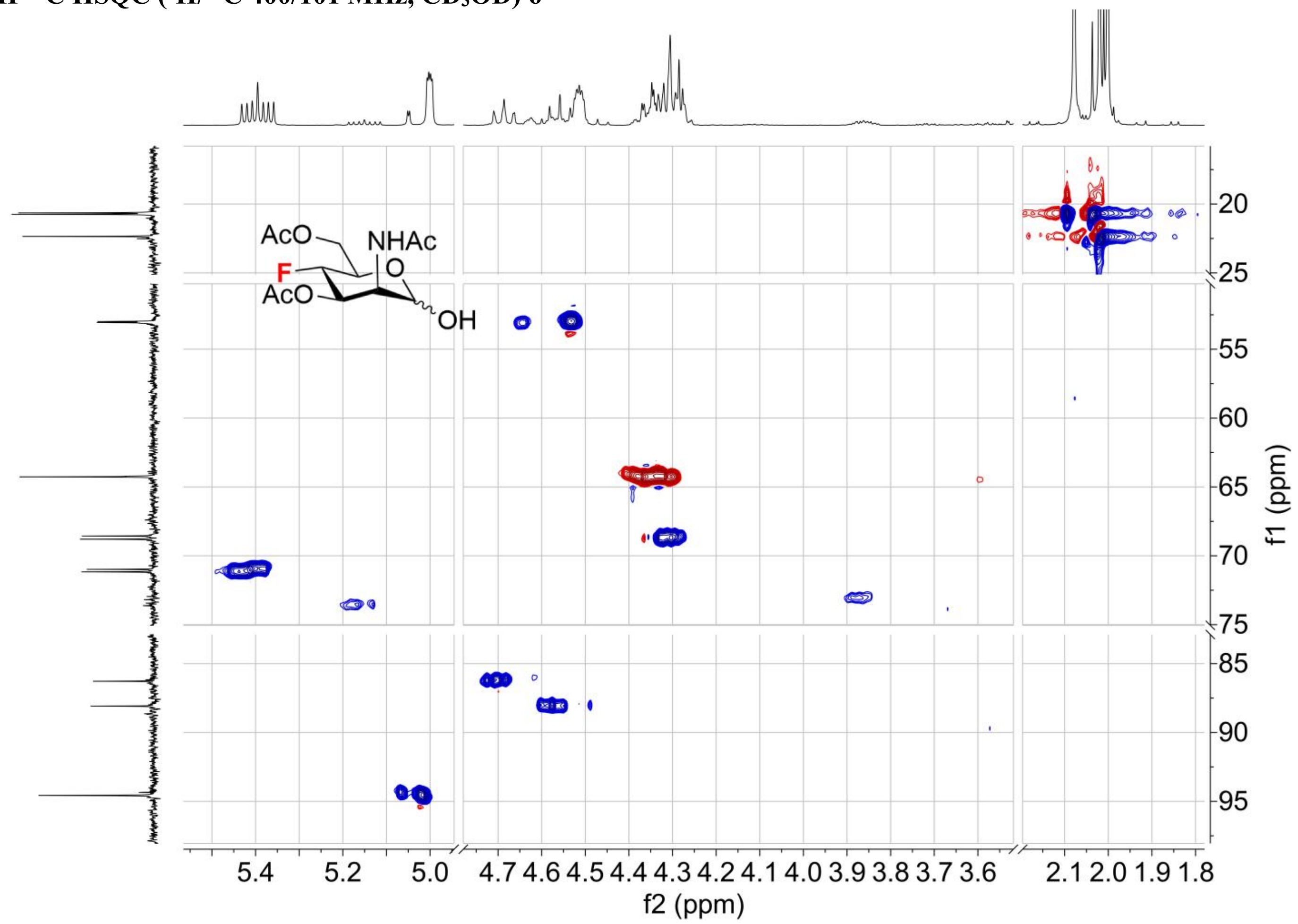

^1H-^{13}C HMBC ($^1H/^{13}C$ 400/101 MHz, CD_3OD) 6

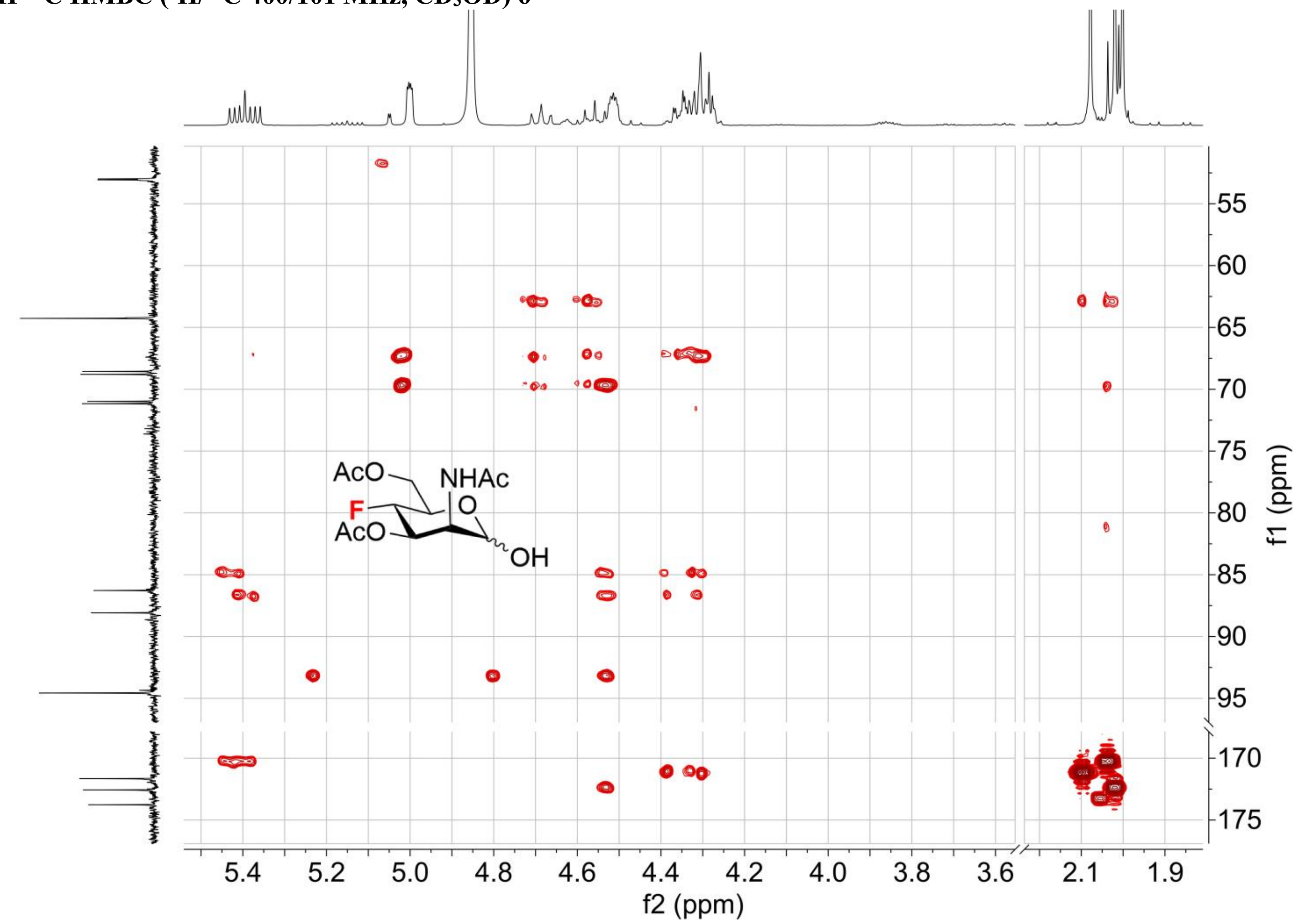

NMR COMPOUND 7

^{1}H NMR (400 MHz, CD_3OD) 7

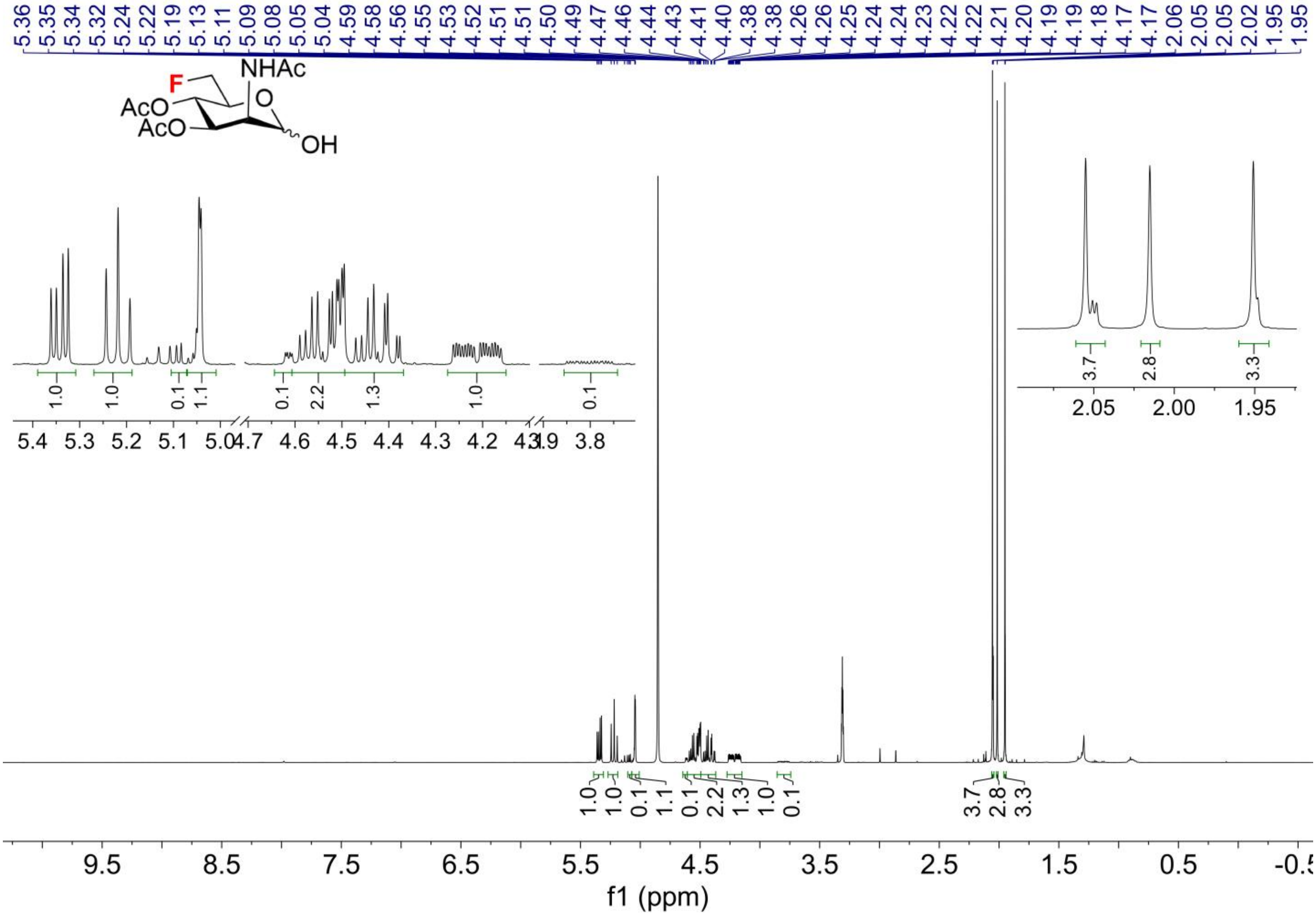

$^{13}C\{^1H\}$ NMR (101 MHz, CD_3OD) 7

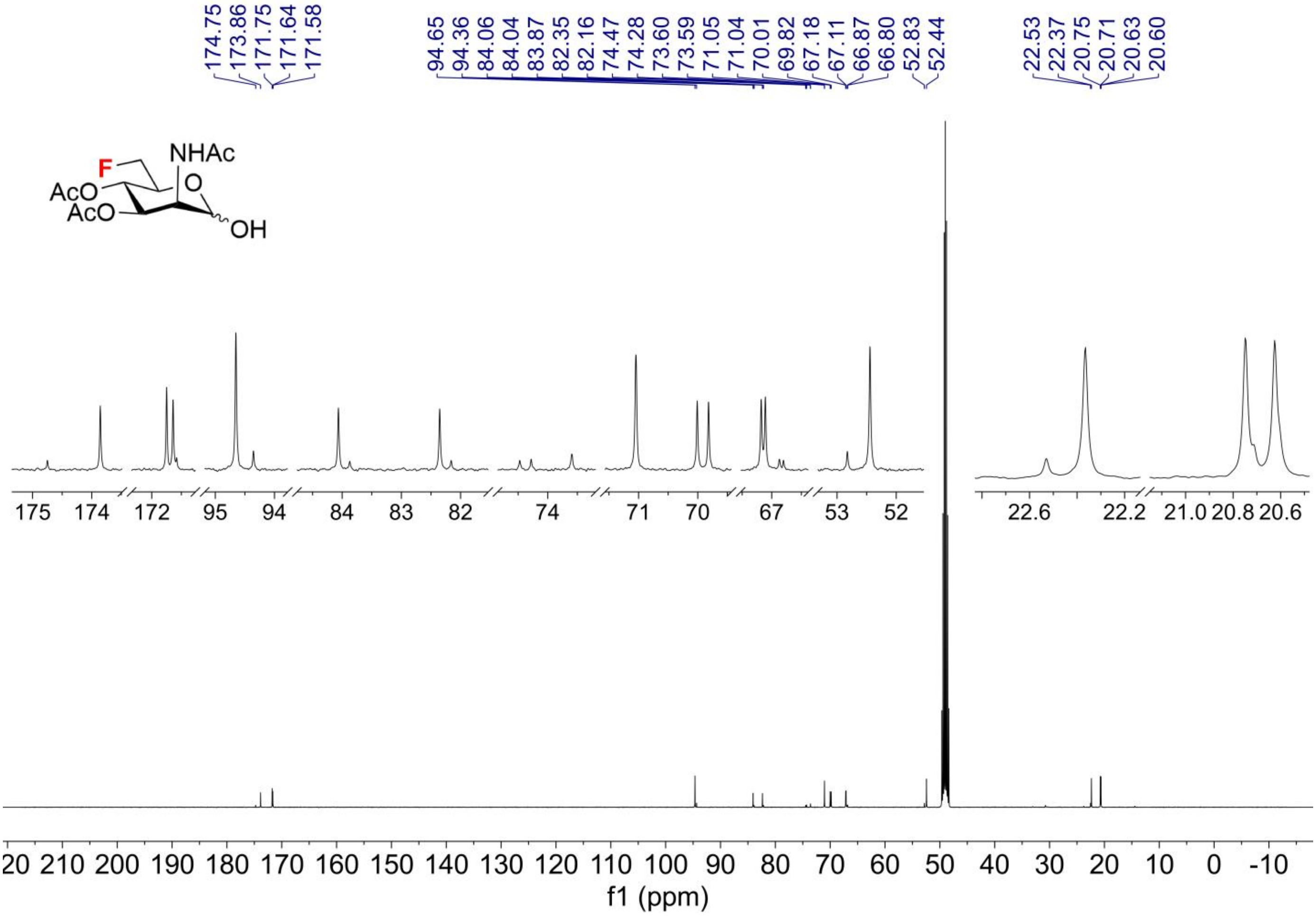

^{19}F NMR (376 MHz, CD_3OD) 7

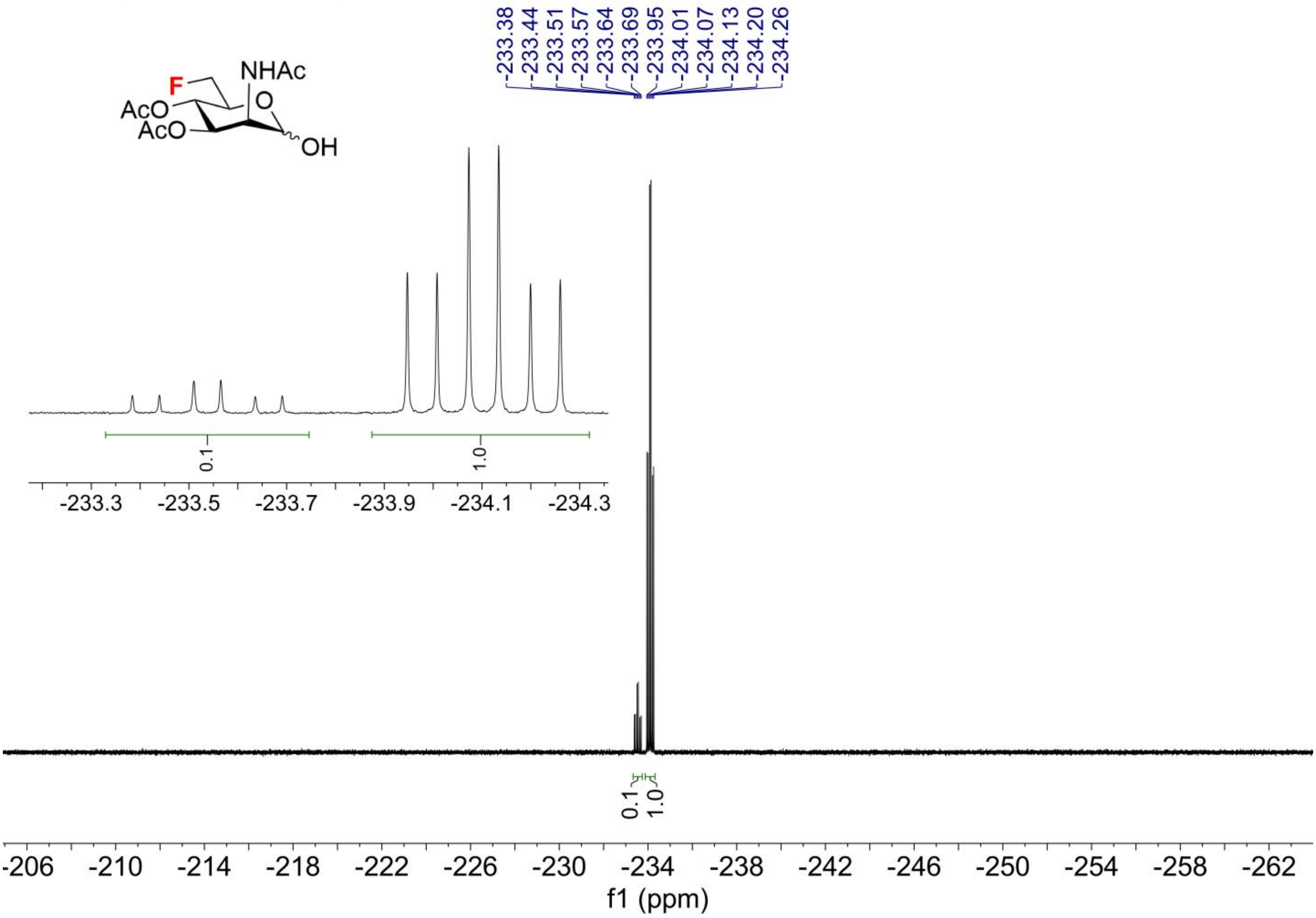

^{1}H-^{1}H COSY (400 MHz, CD_3OD) 7

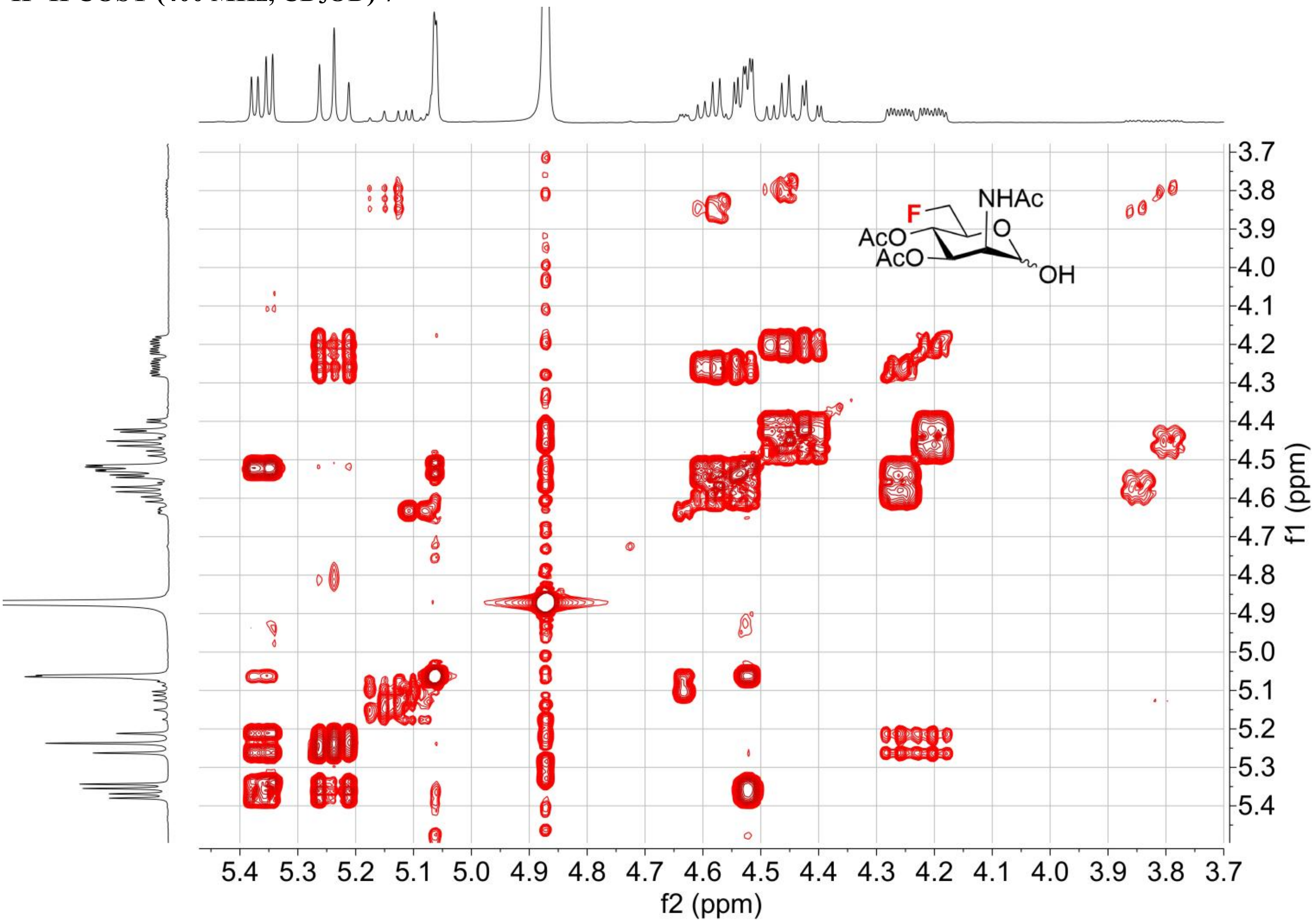

^{1}H-^{13}C HSQC (^{1}H/^{13}C 400/101 MHz, CD_3OD) 7

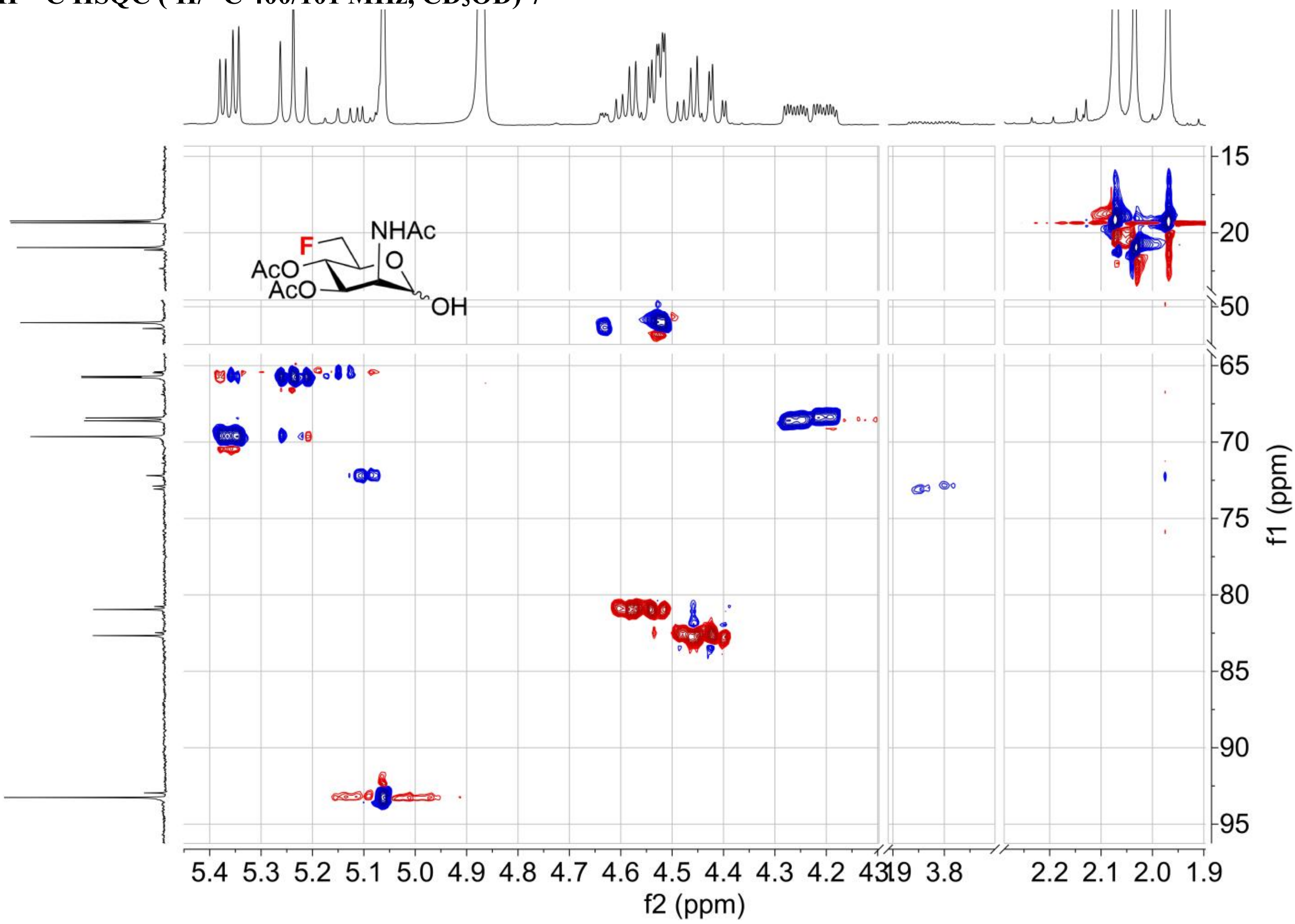

^{1}H-^{13}C HMBC (^{1}H/^{13}C 400/101 MHz, CD_3OD) 7

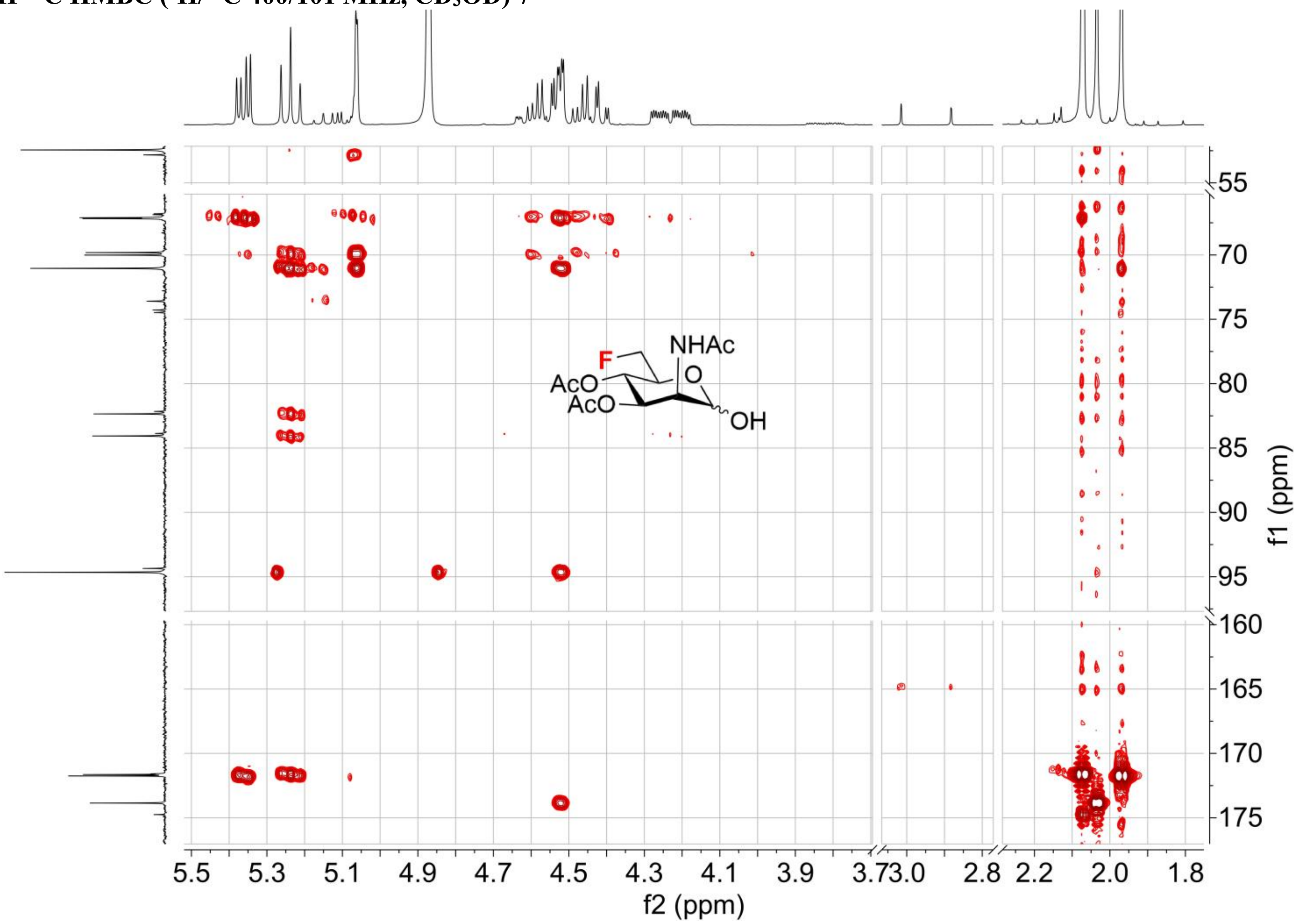

NMR COMPOUND 8

^{1}H NMR (400 MHz, CD_3OD) 8

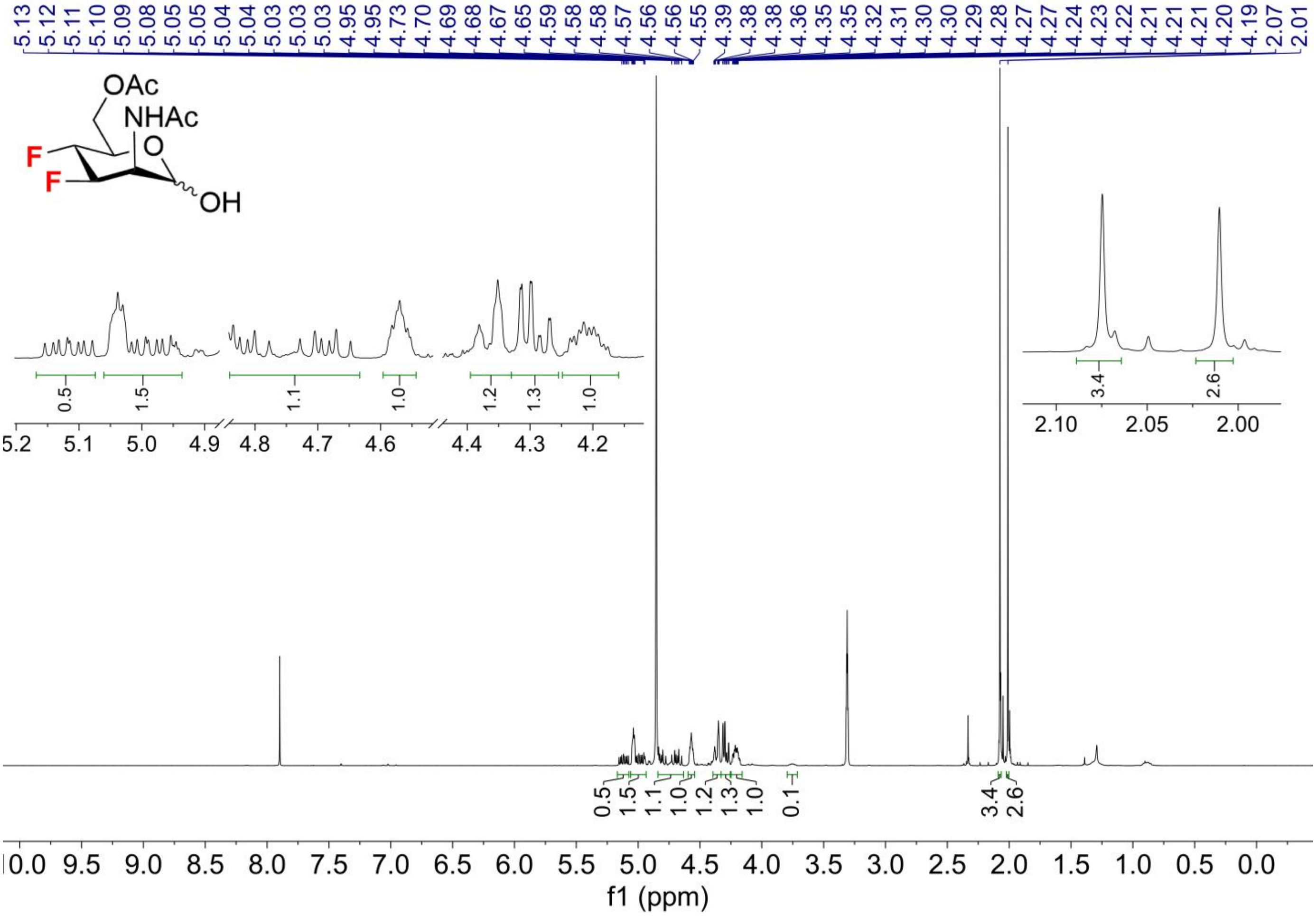

^{13}C{^{1}H} NMR (101 MHz, CD_3OD) 8

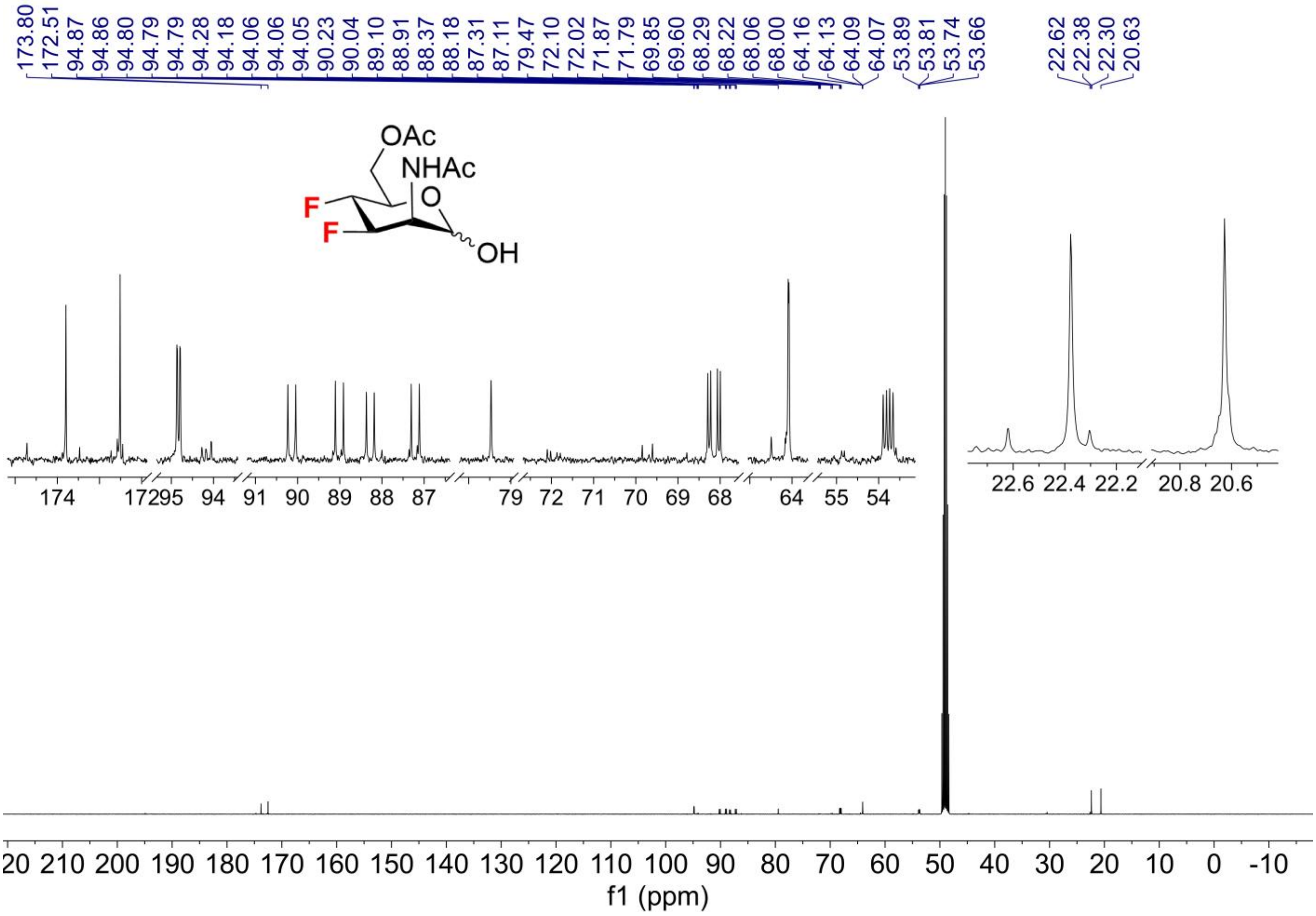

^{19}F NMR (376 MHz, CD_3OD) 8

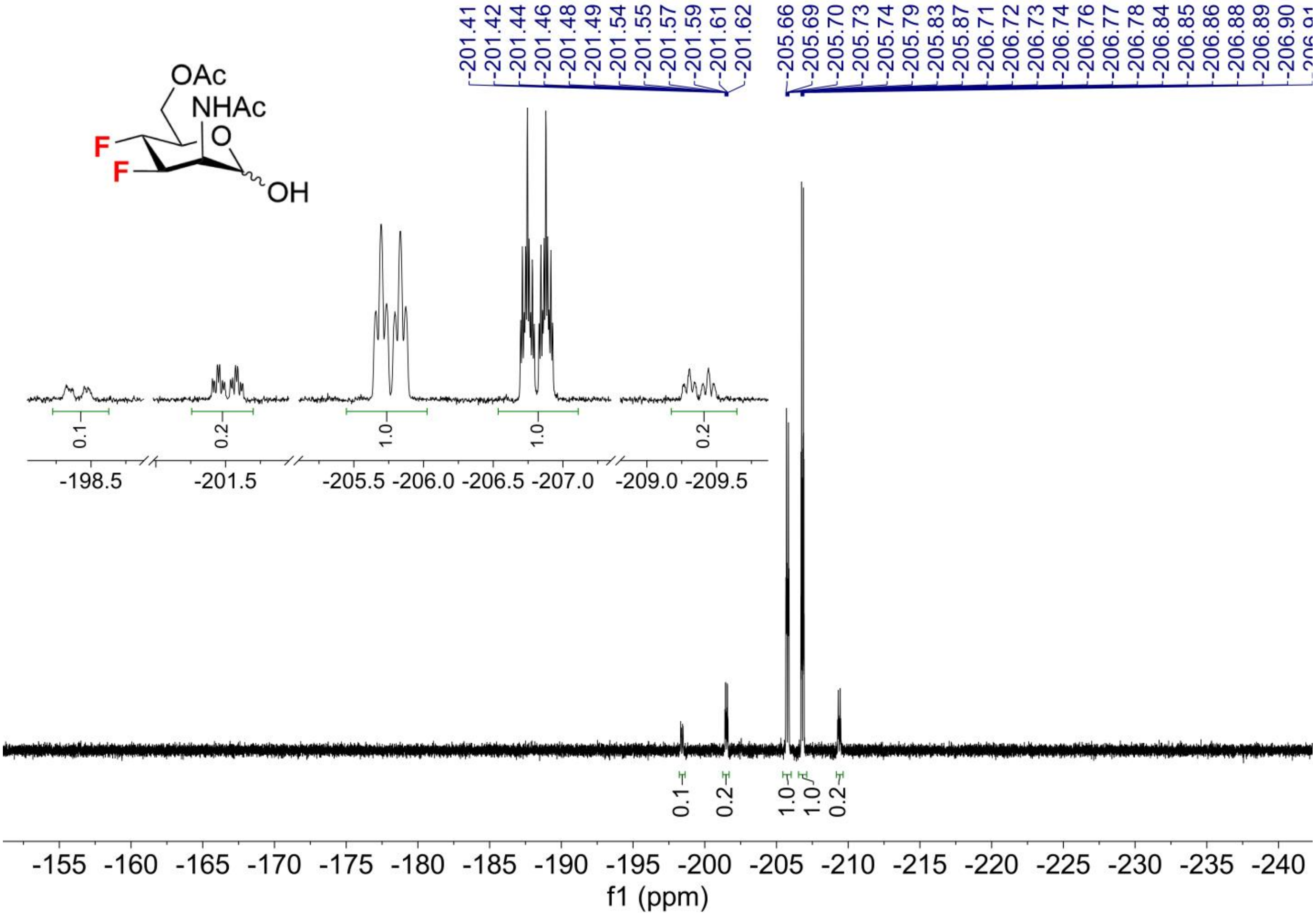

^{1}H-^{1}H COSY (400 MHz, CD_3OD) 8

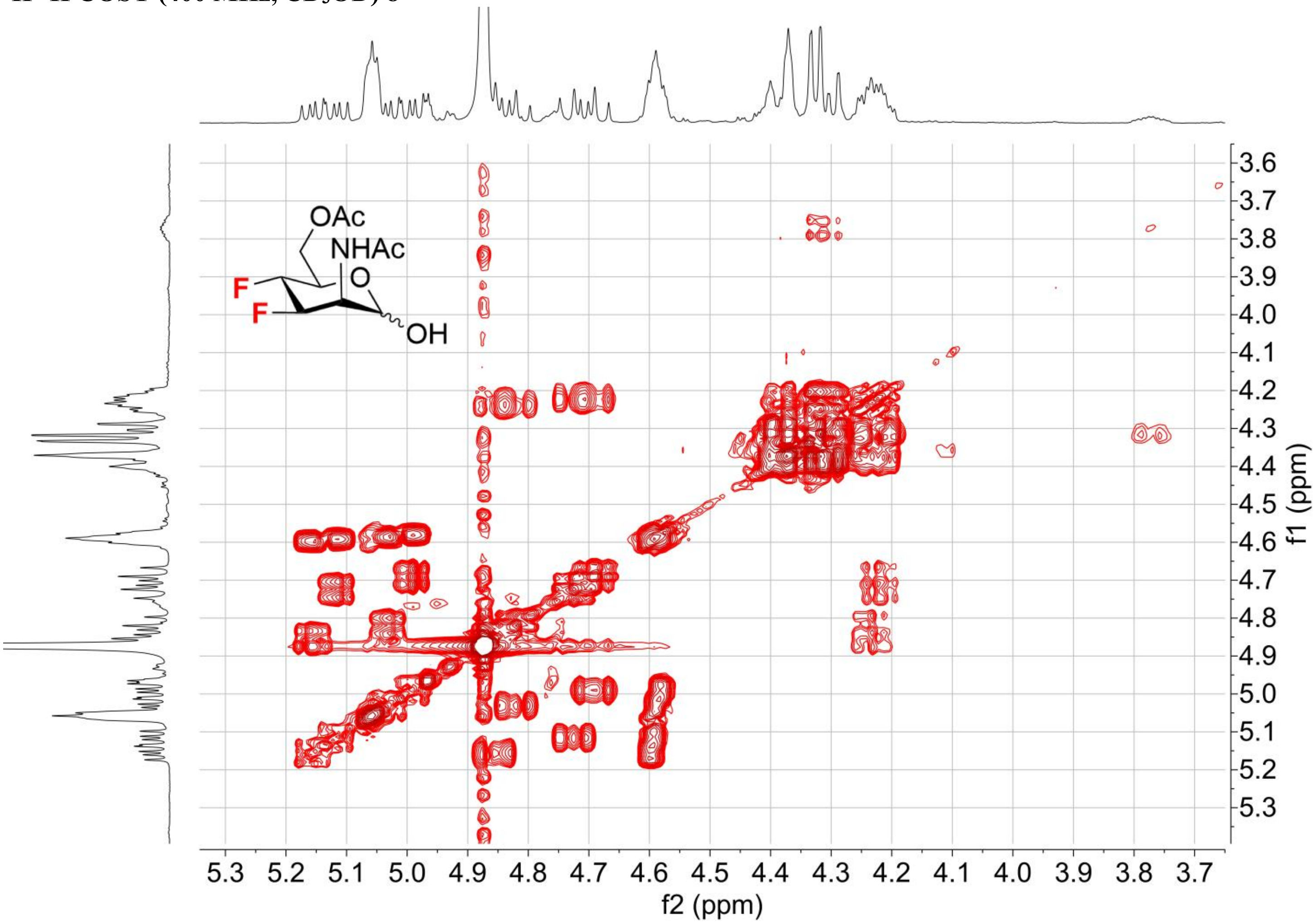

^{1}H-^{13}C HSQC (^{1}H/^{13}C 400/101 MHz, CD_3OD) 8

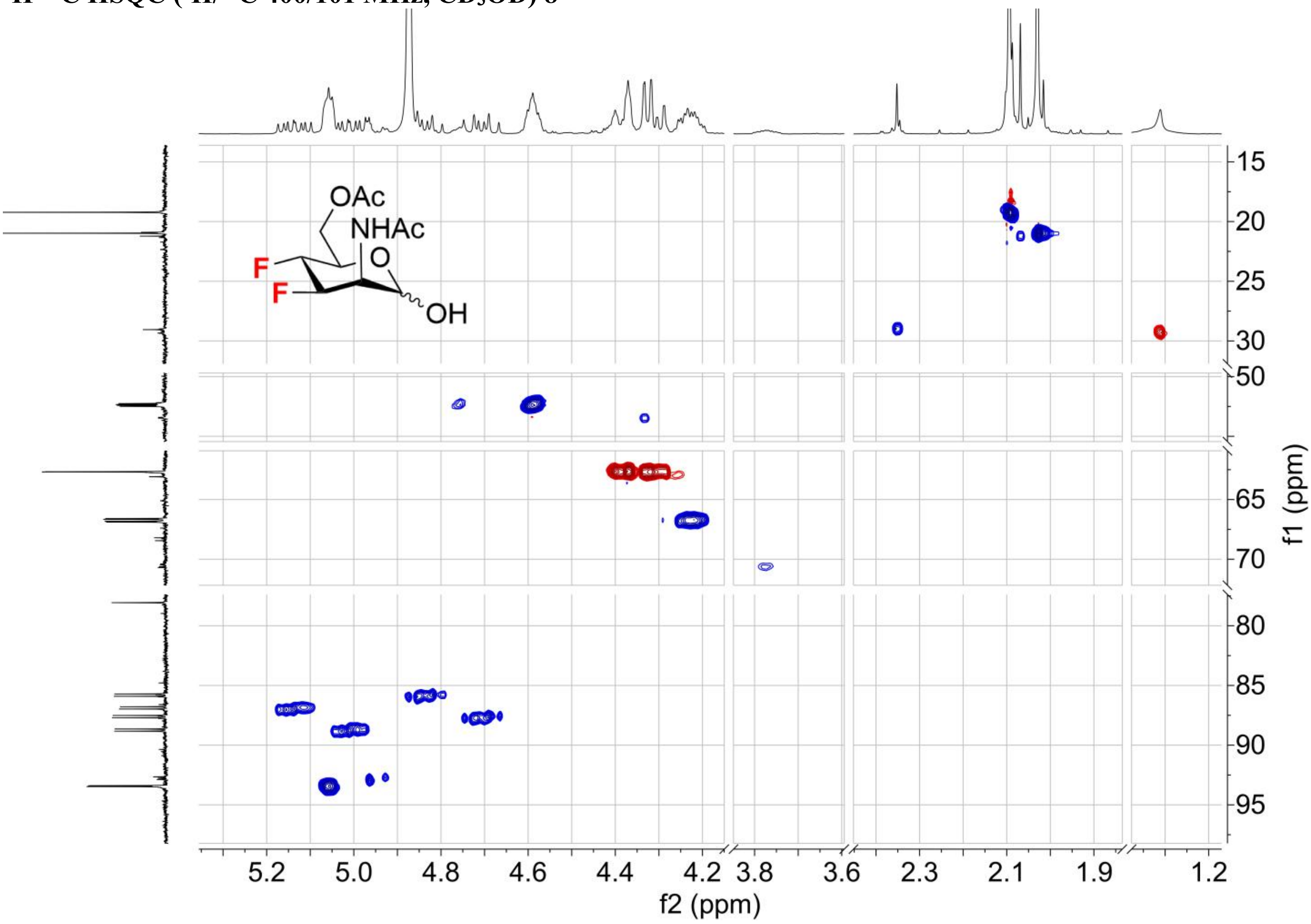

^{1}H-^{13}C HMBC (^{1}H/^{13}C 400/101 MHz, CD_3OD) 8

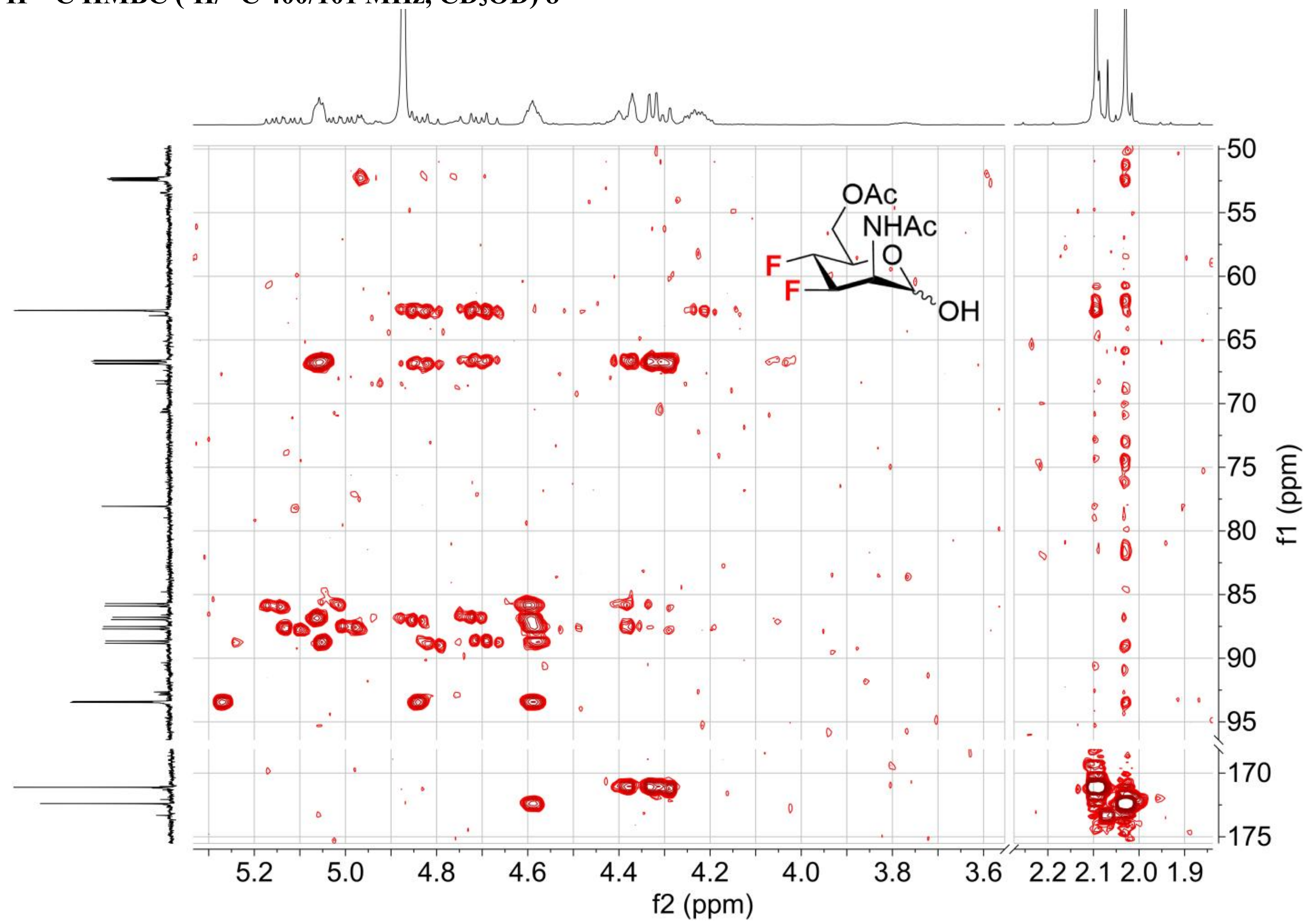

NMR COMPOUND 9

^{1}H NMR (400 MHz, CD_3OD) 9

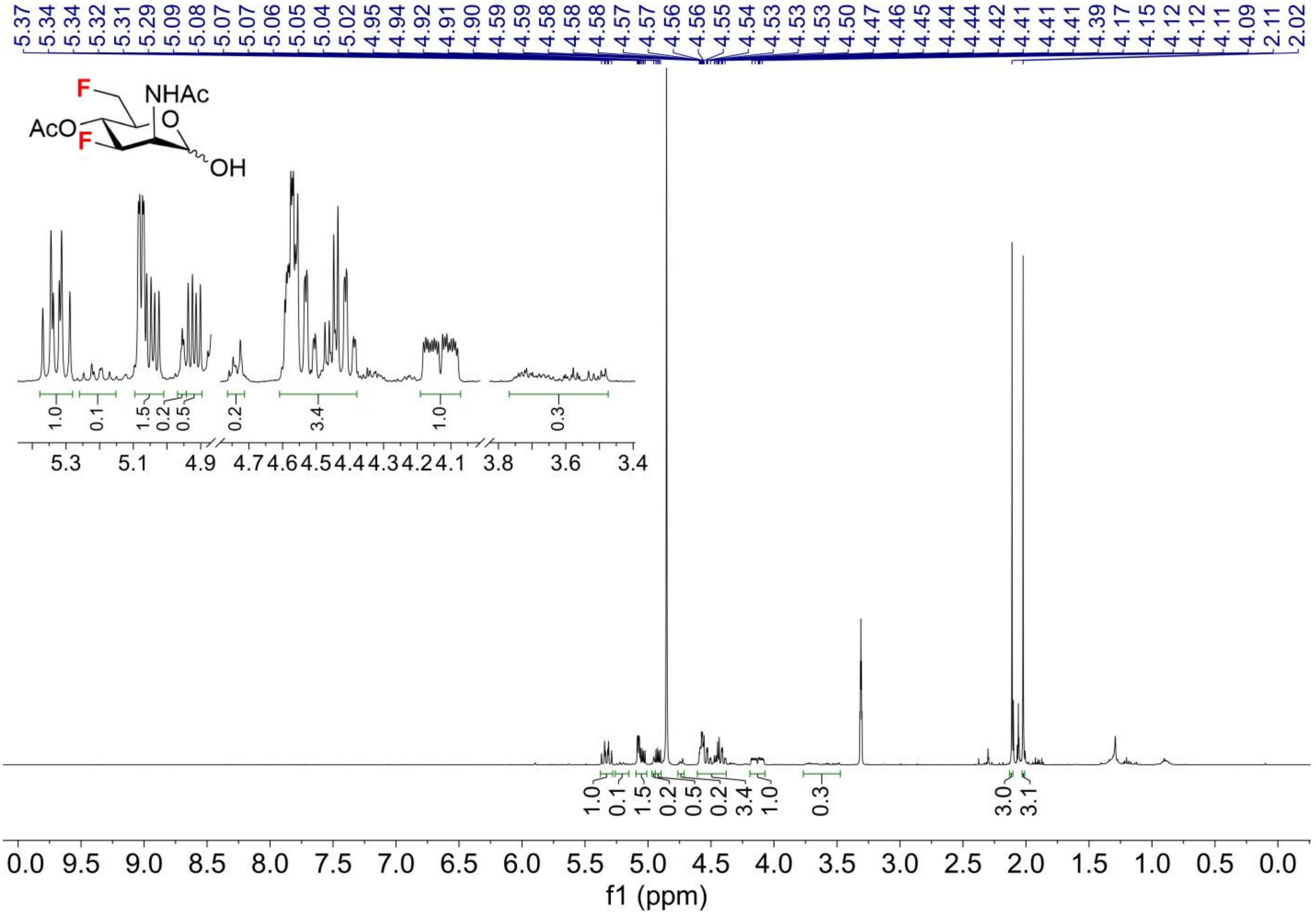

^{13}C{^{1}H} NMR (101 MHz, CD_3OD) 9

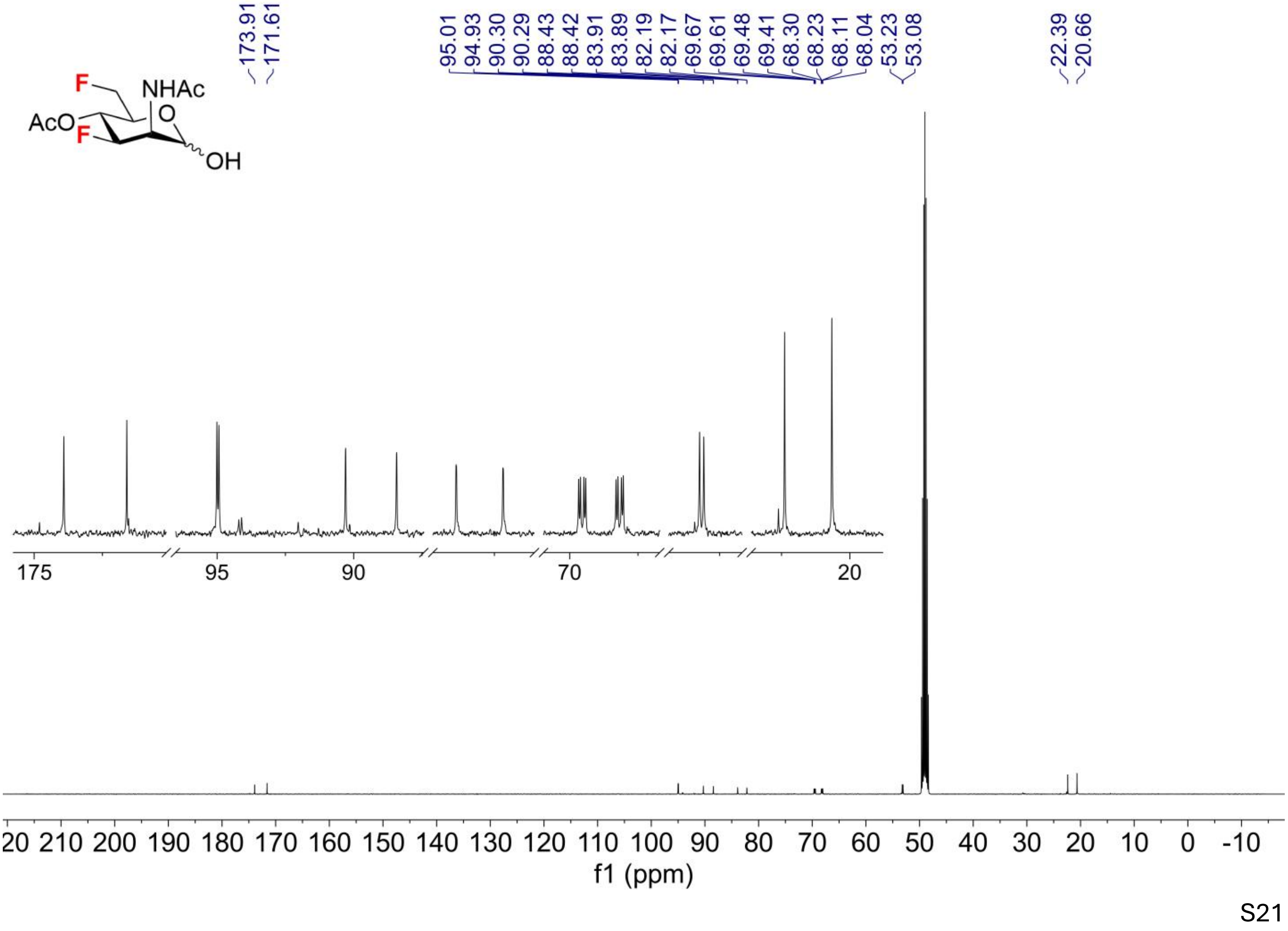

^{19}F NMR (376 MHz, CD_3OD) 9

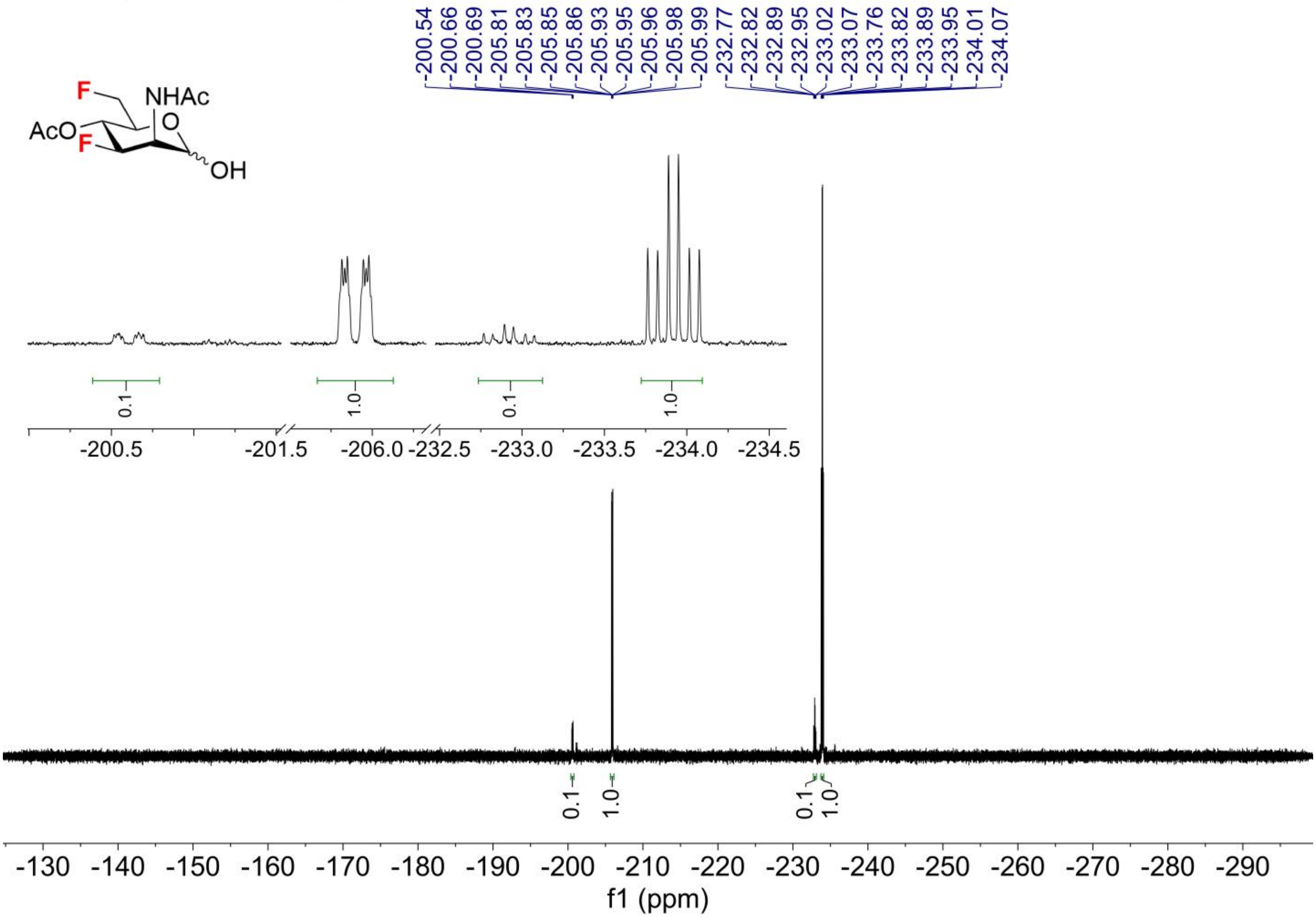

^{1}H-^{1}H COSY (400 MHz, CD_3OD) 9

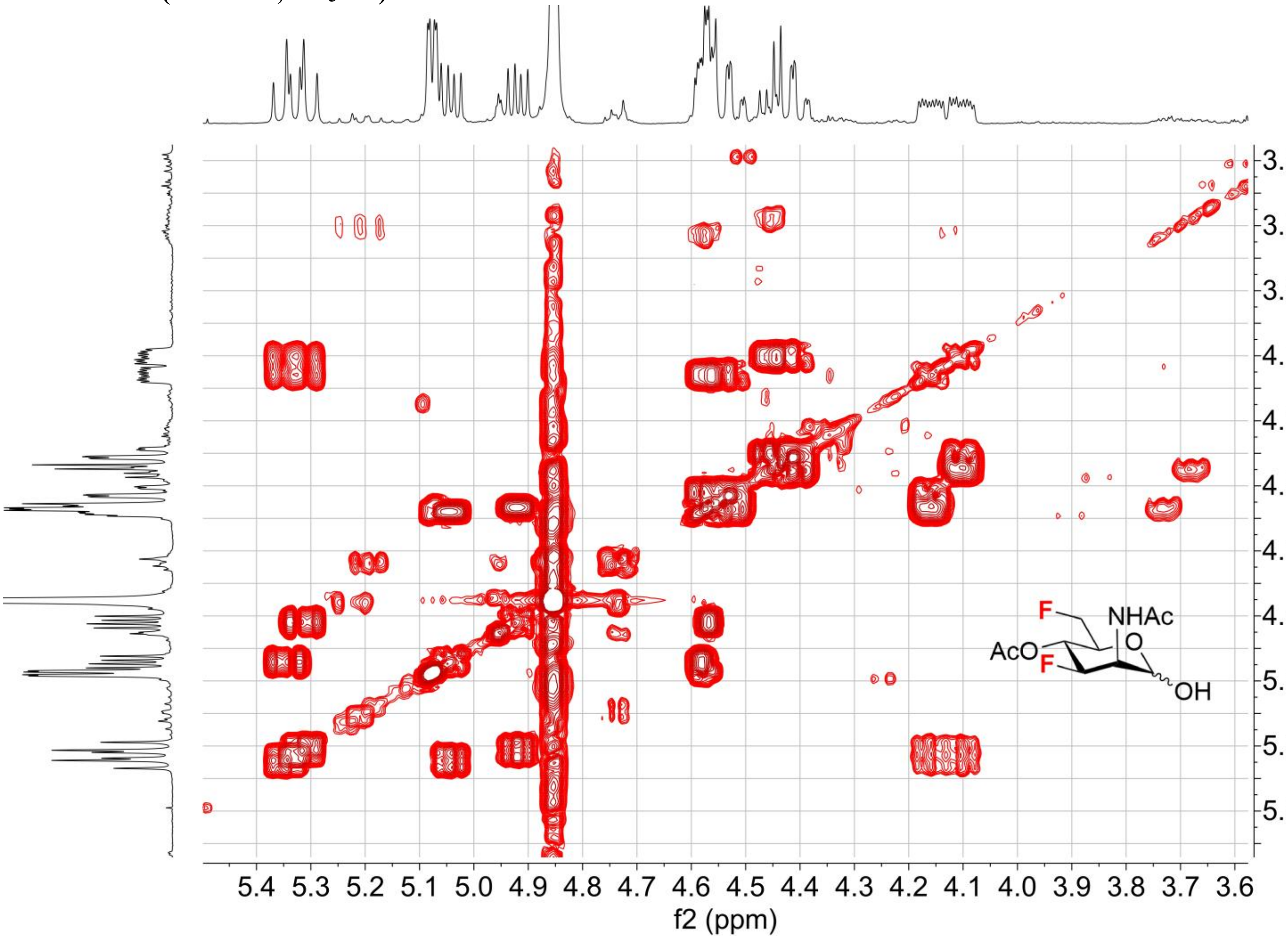

^{1}H-^{13}C HSQC ($^{1}H/^{13}C$ 400/101 MHz, CD_3OD) 9

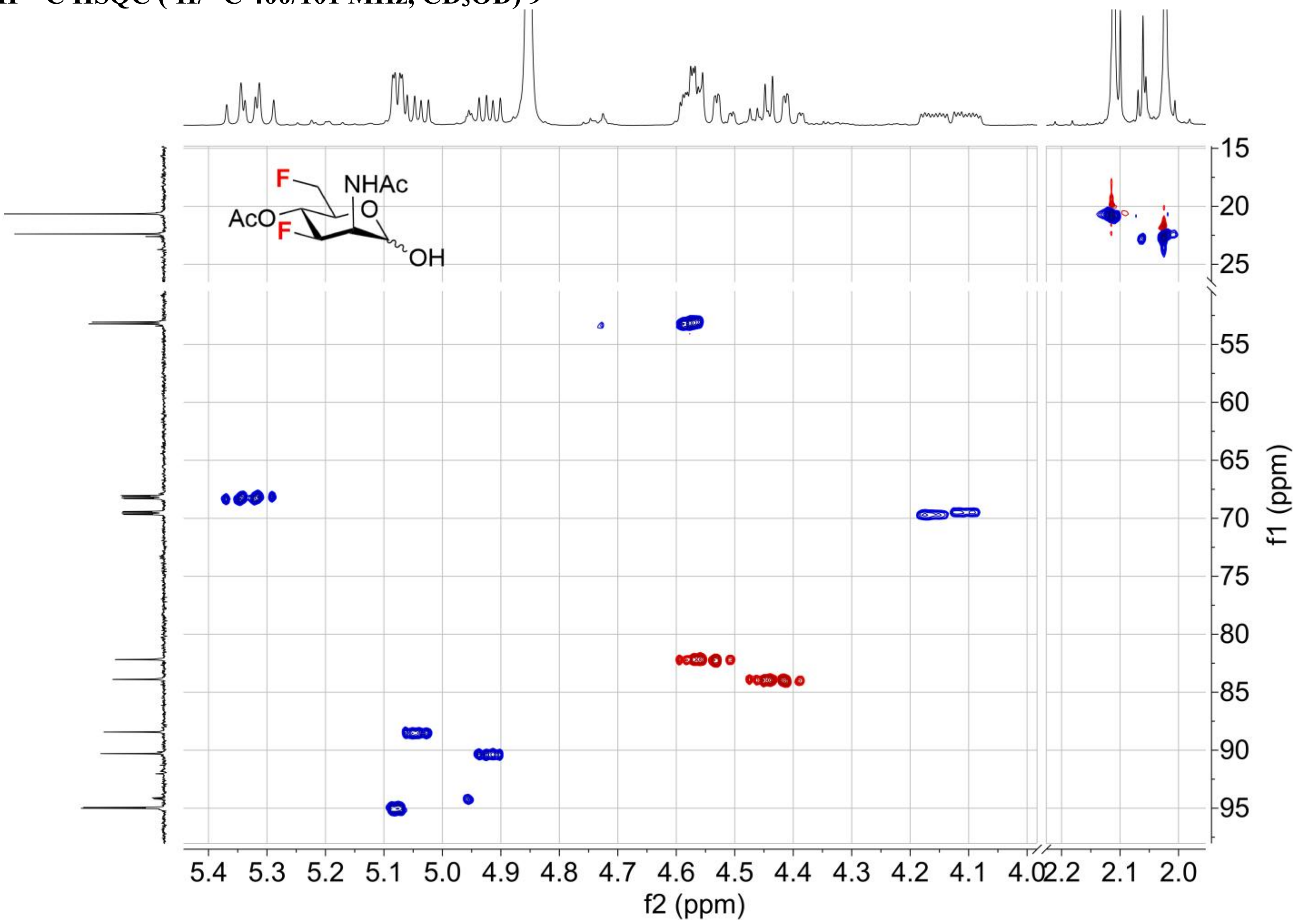

^{1}H-^{13}C HMBC ($^{1}H/^{13}C$ 400/101 MHz, CD_3OD) 9

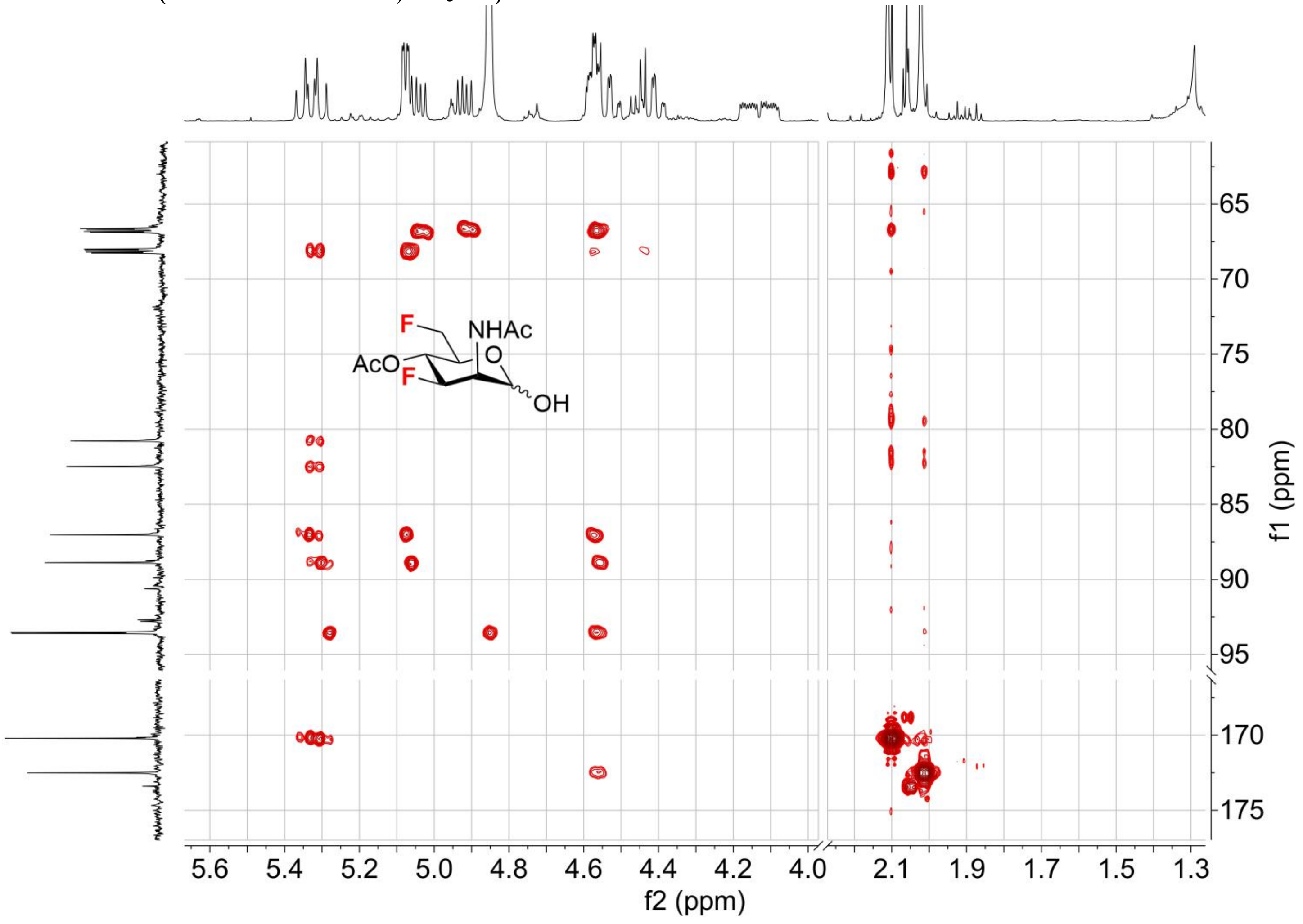

NMR COMPOUND 10

^{1}H NMR (400 MHz, CD_3OD) 10

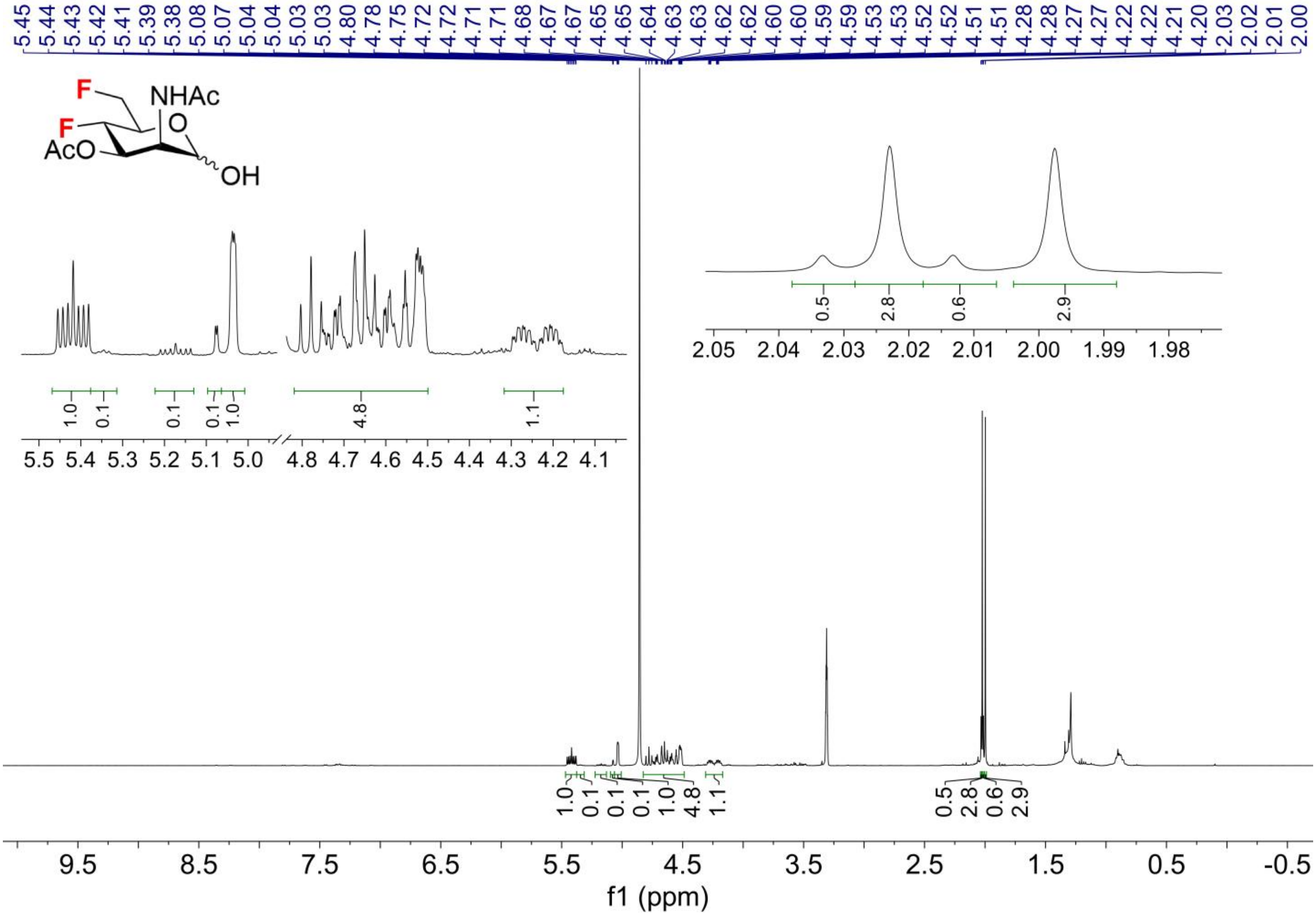

$^{13}C\{^{1}H\}$ NMR (101 MHz, CD_3OD) 10

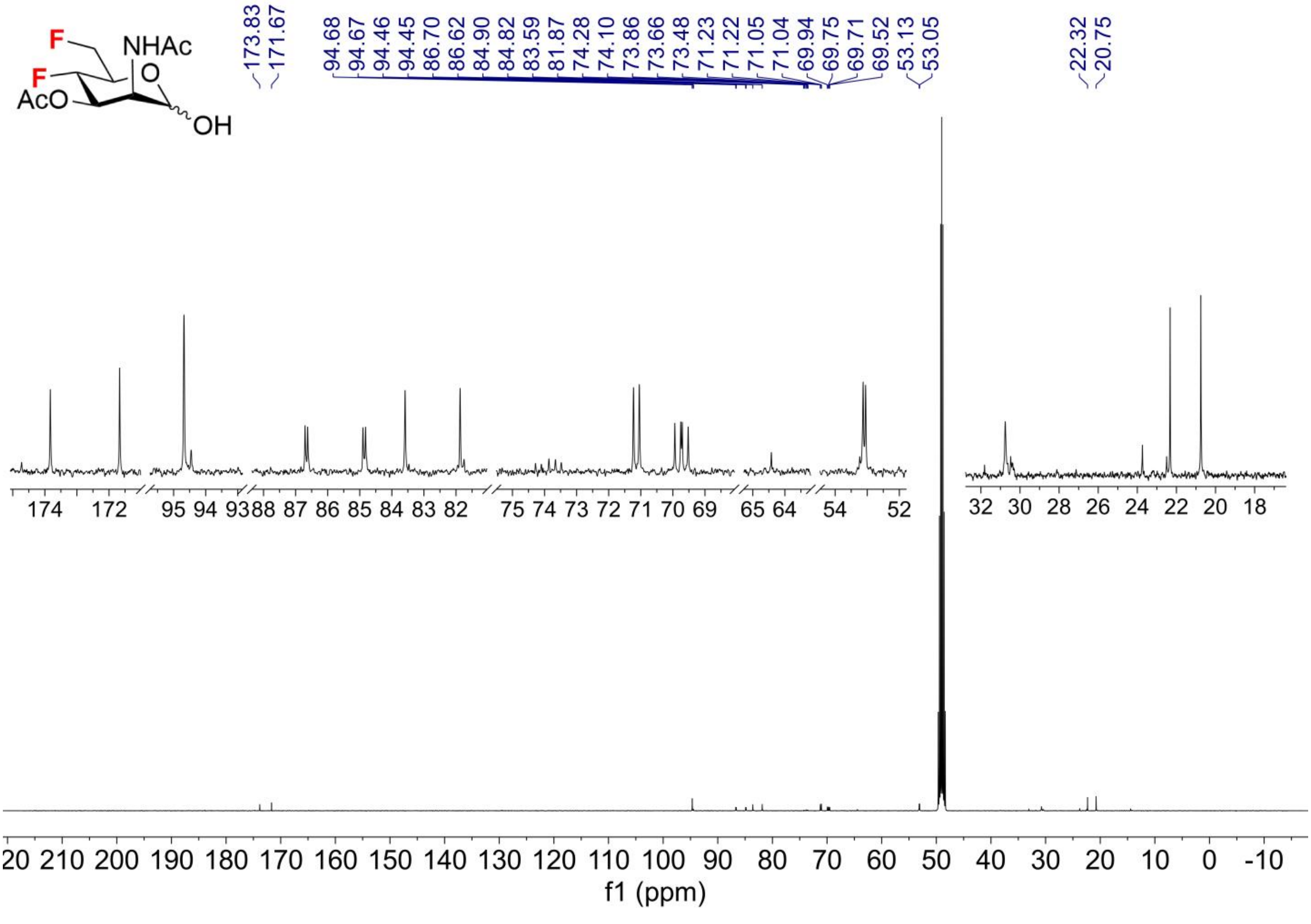

^{19}F NMR (376 MHz, CD$_3$OD) 10

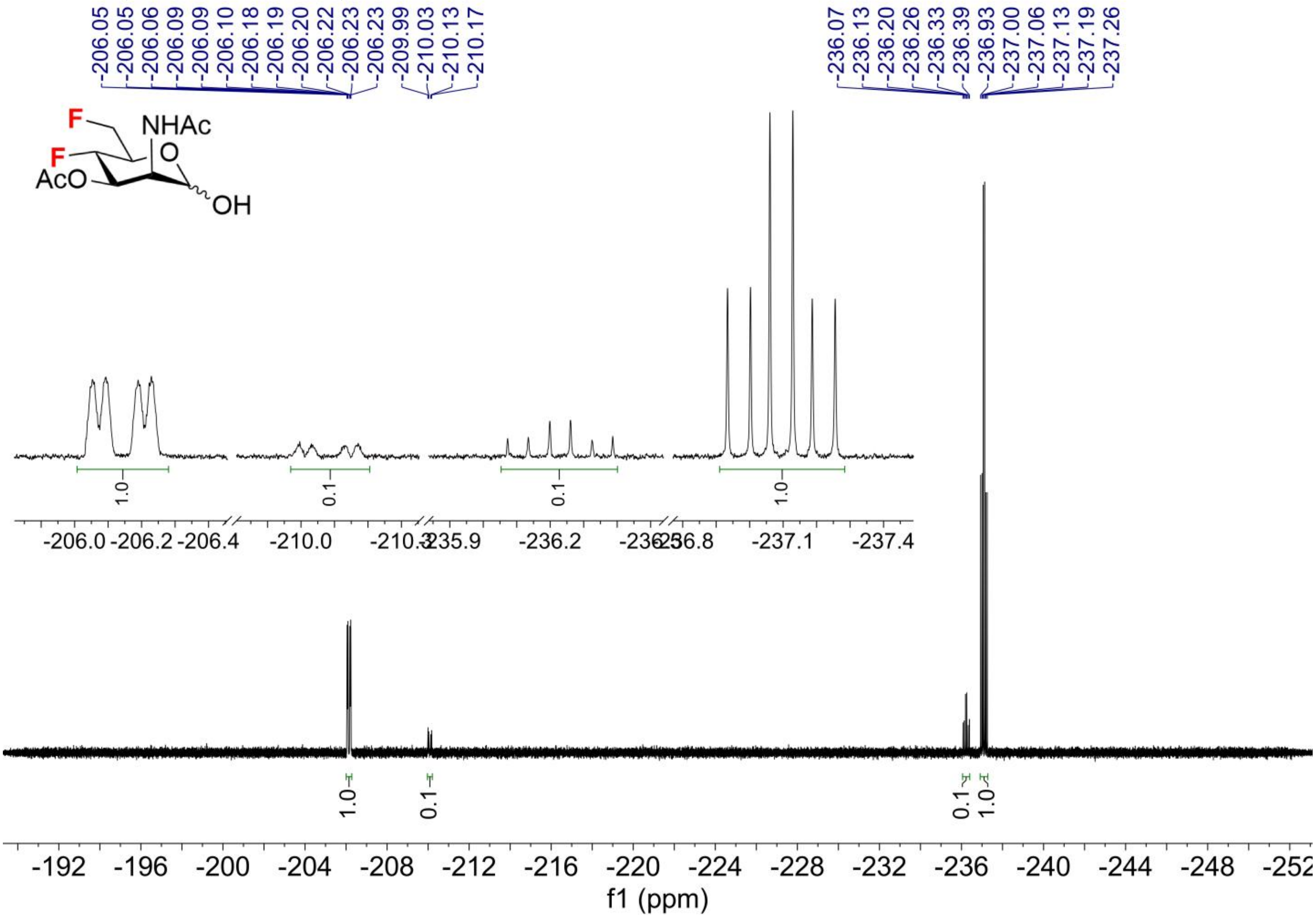

^{1}H-^{1}H COSY (400 MHz, CD$_3$OD) 10

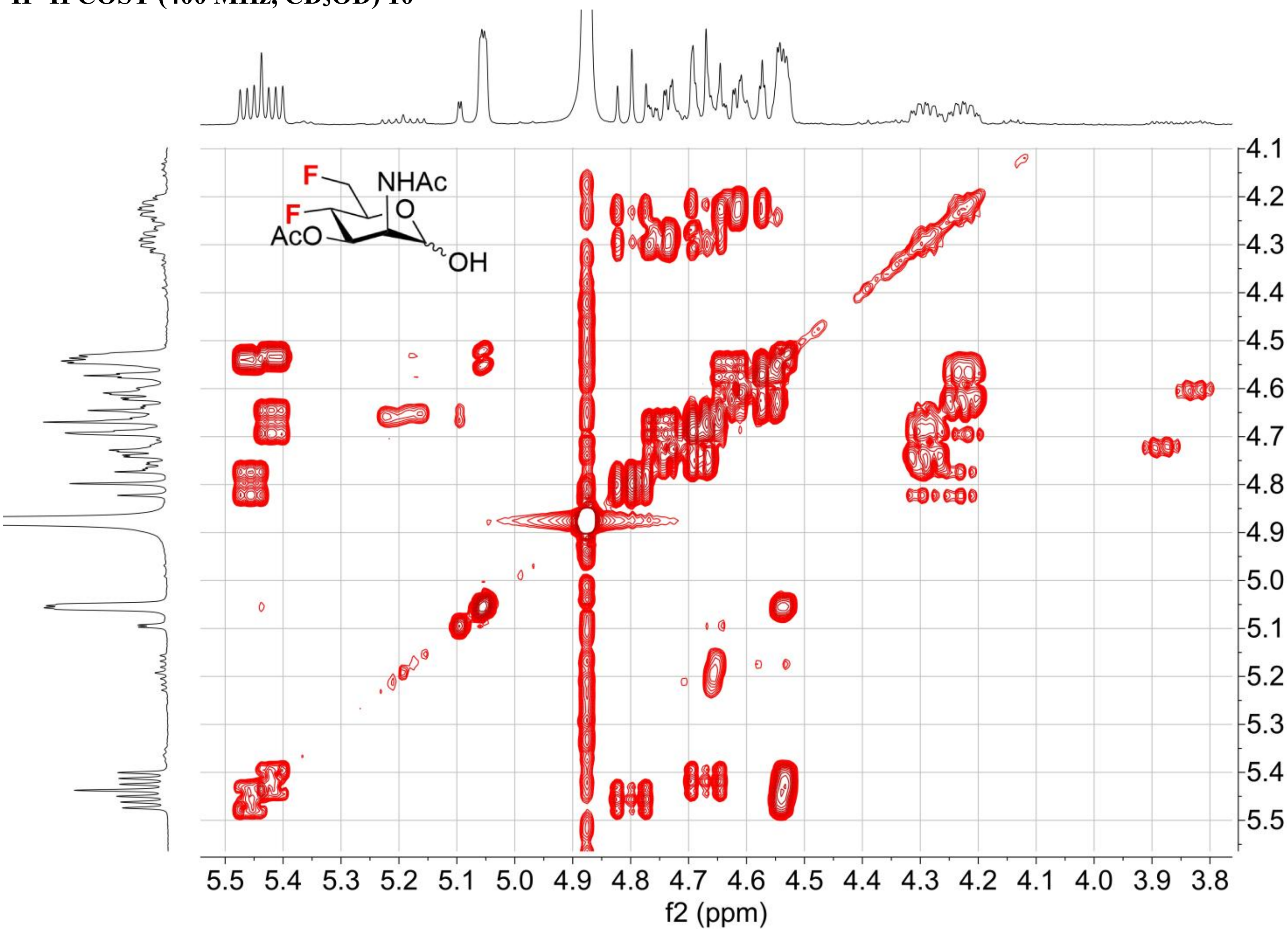

^{1}H-^{13}C HSQC (^{1}H/^{13}C 400/101 MHz, CD_3OD) 10

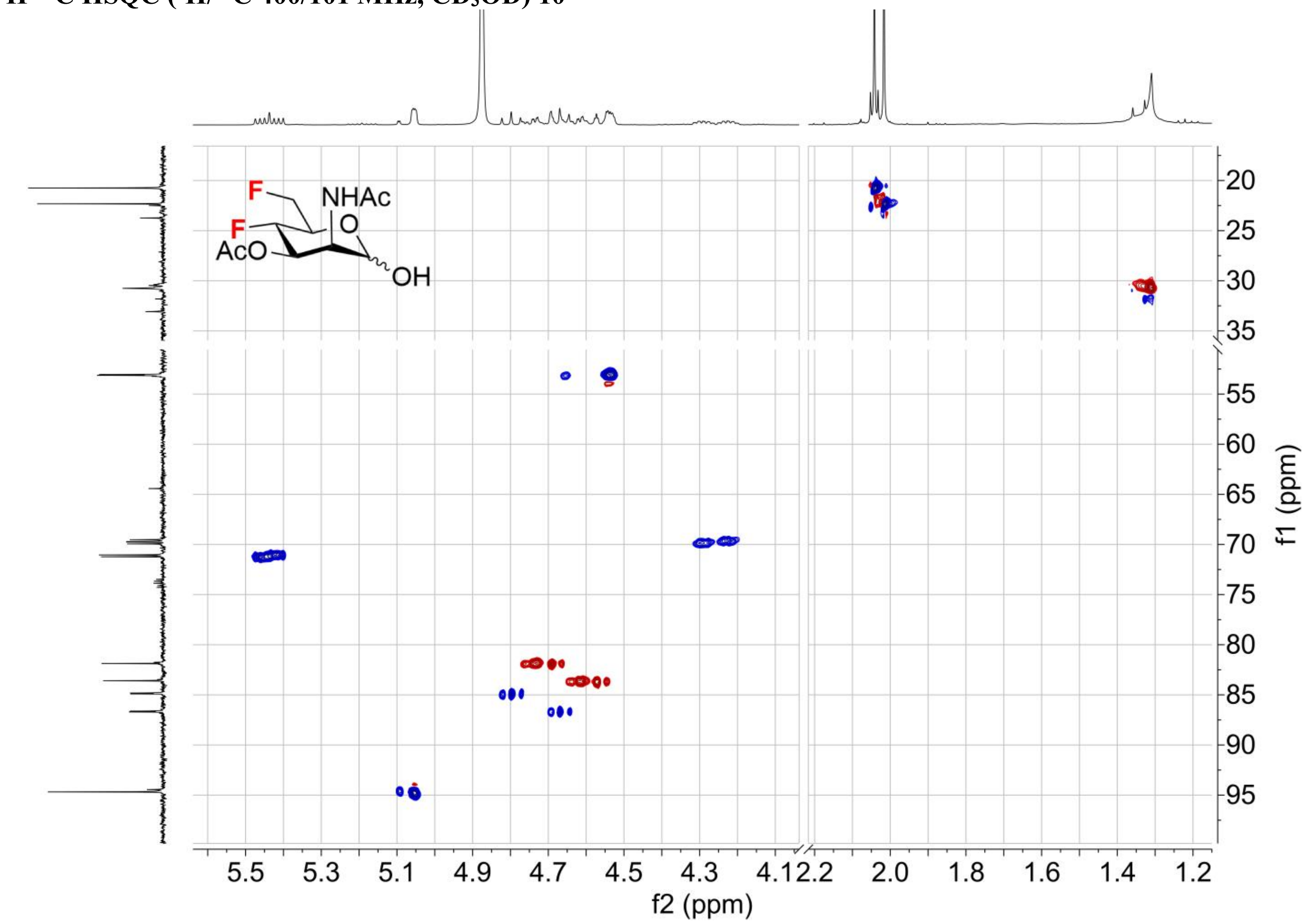

^{1}H-^{13}C HMBC (^{1}H/^{13}C 400/101 MHz, CD_3OD) 10

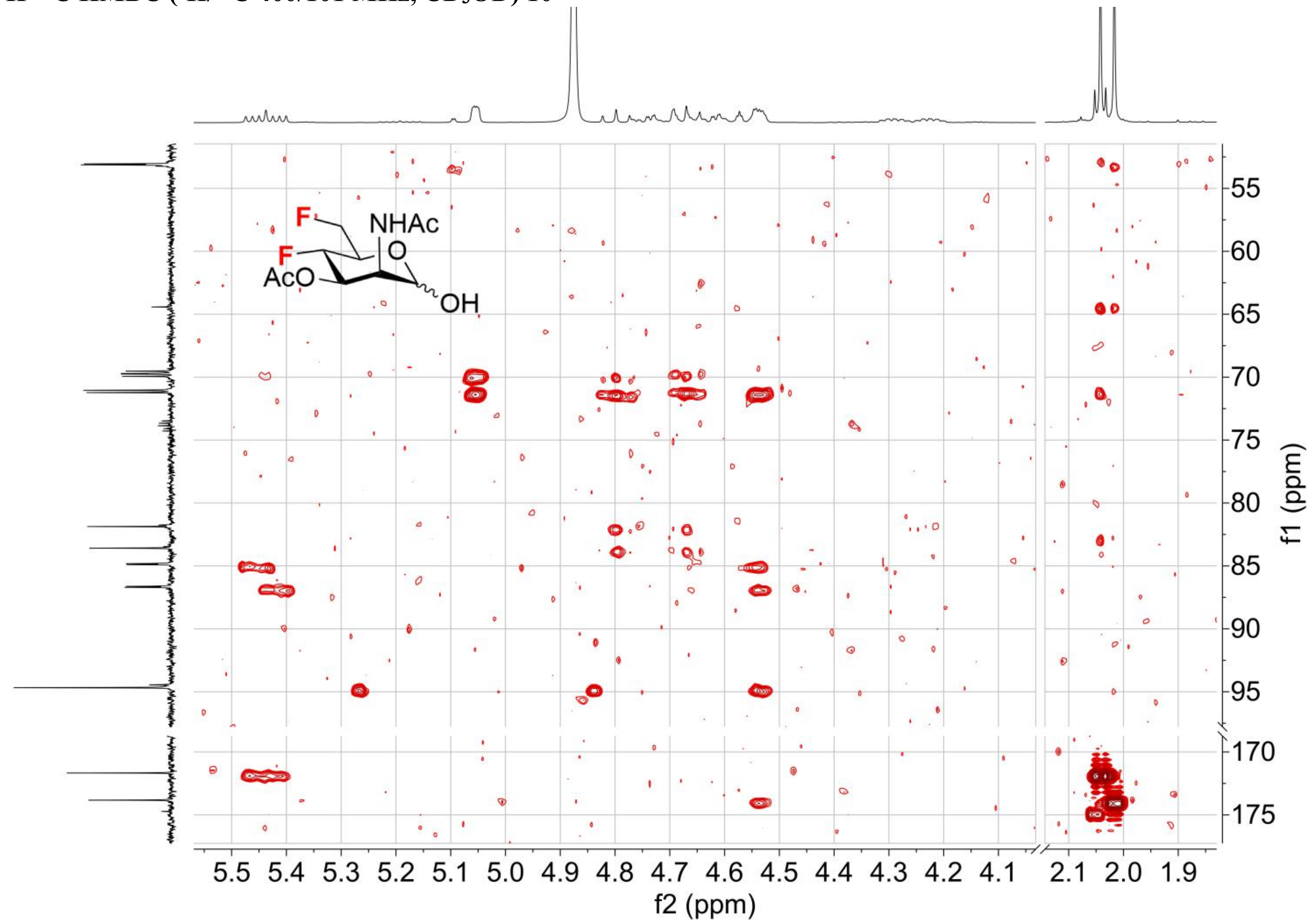

NMR COMPOUND 11

^{1}H NMR (400 MHz, CD$_3$OD) 11

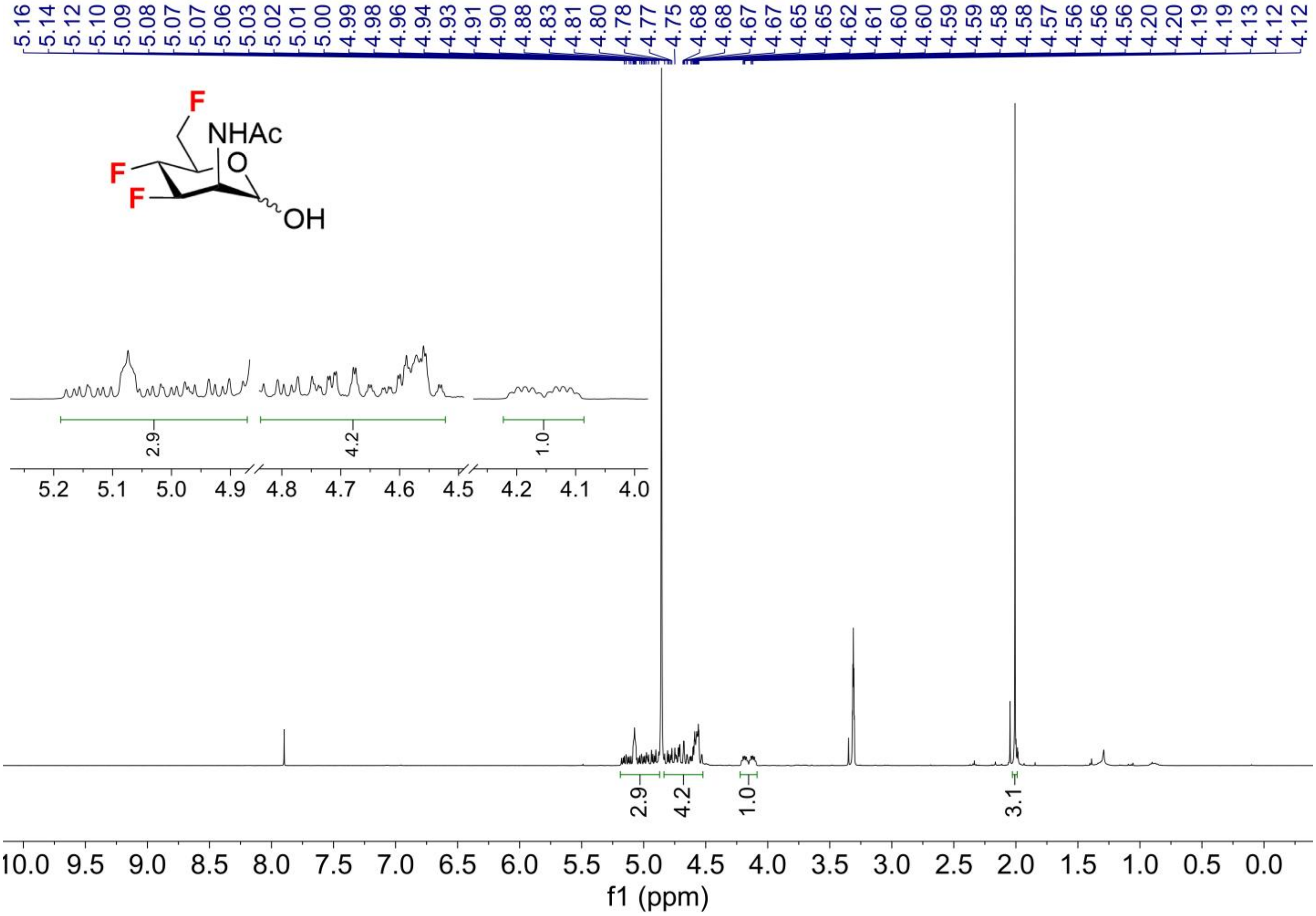

^{13}C{^{1}H} NMR (101 MHz, CD$_3$OD) 11

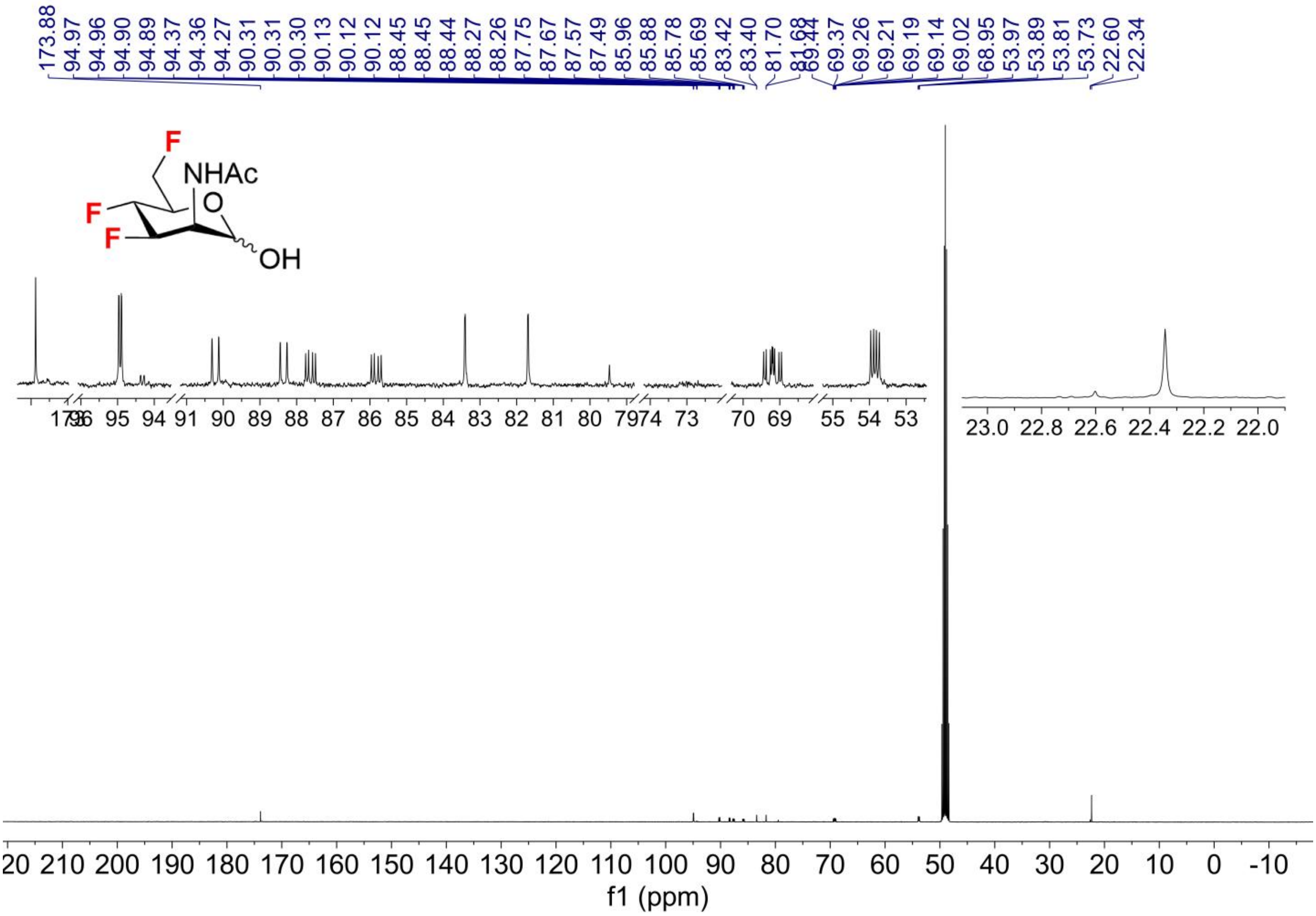

^{19}F NMR (376 MHz, CD3OD) 11

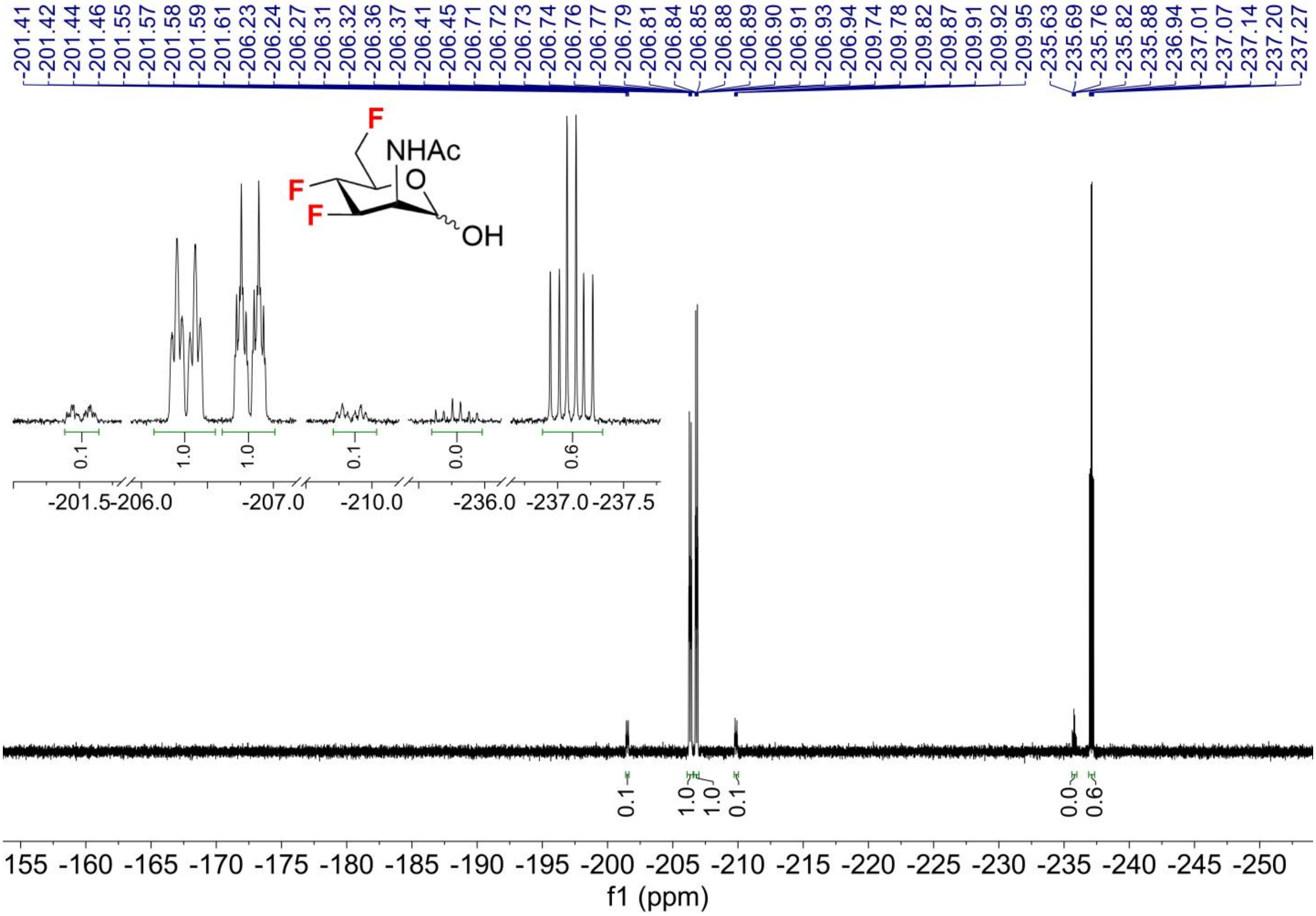

^{1}H-^{1}H COSY (400 MHz, CD3OD) 11

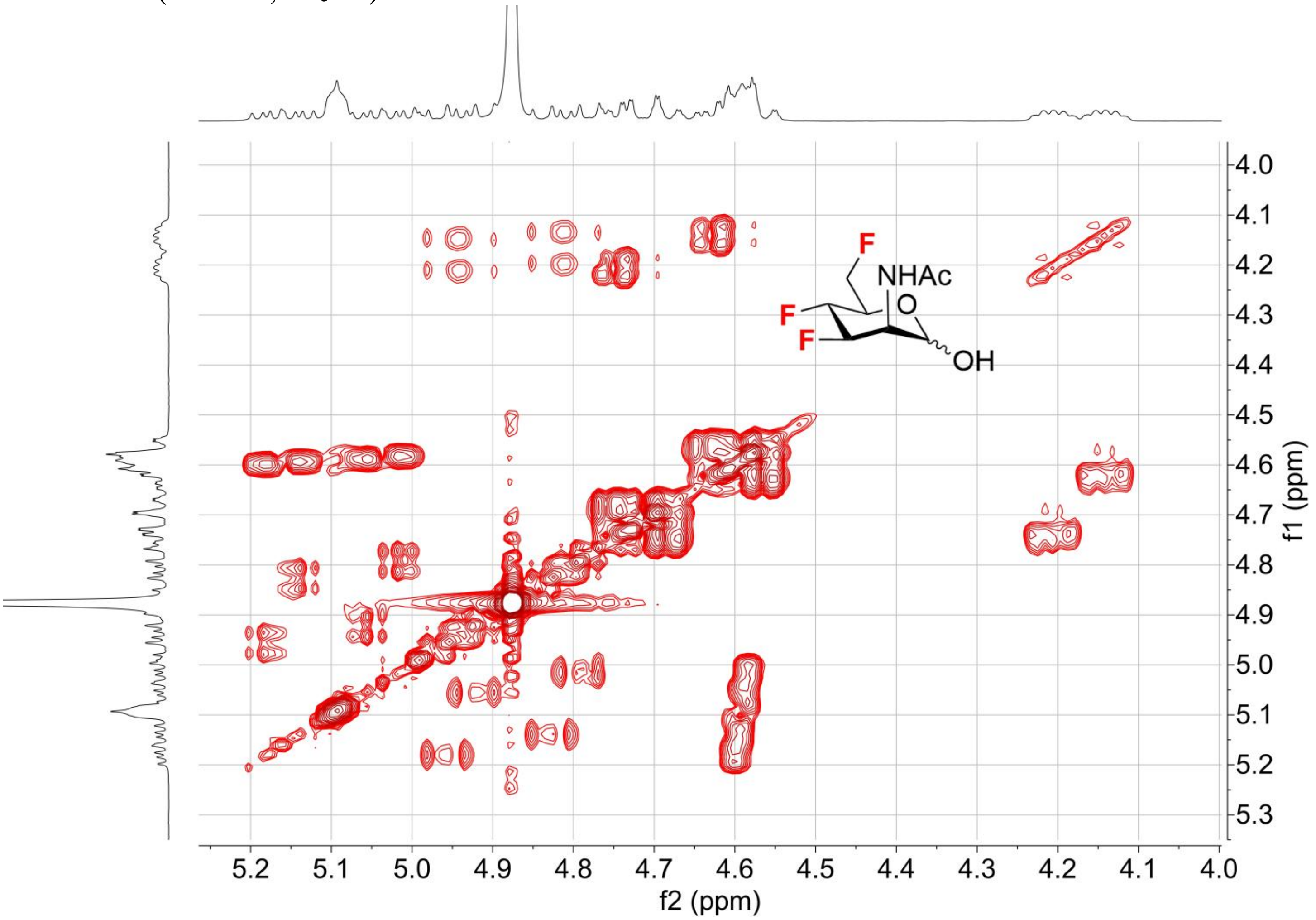

^1H-^{13}C HSQC (^1H/^{13}C 400/101 MHz, CD_3OD) 11

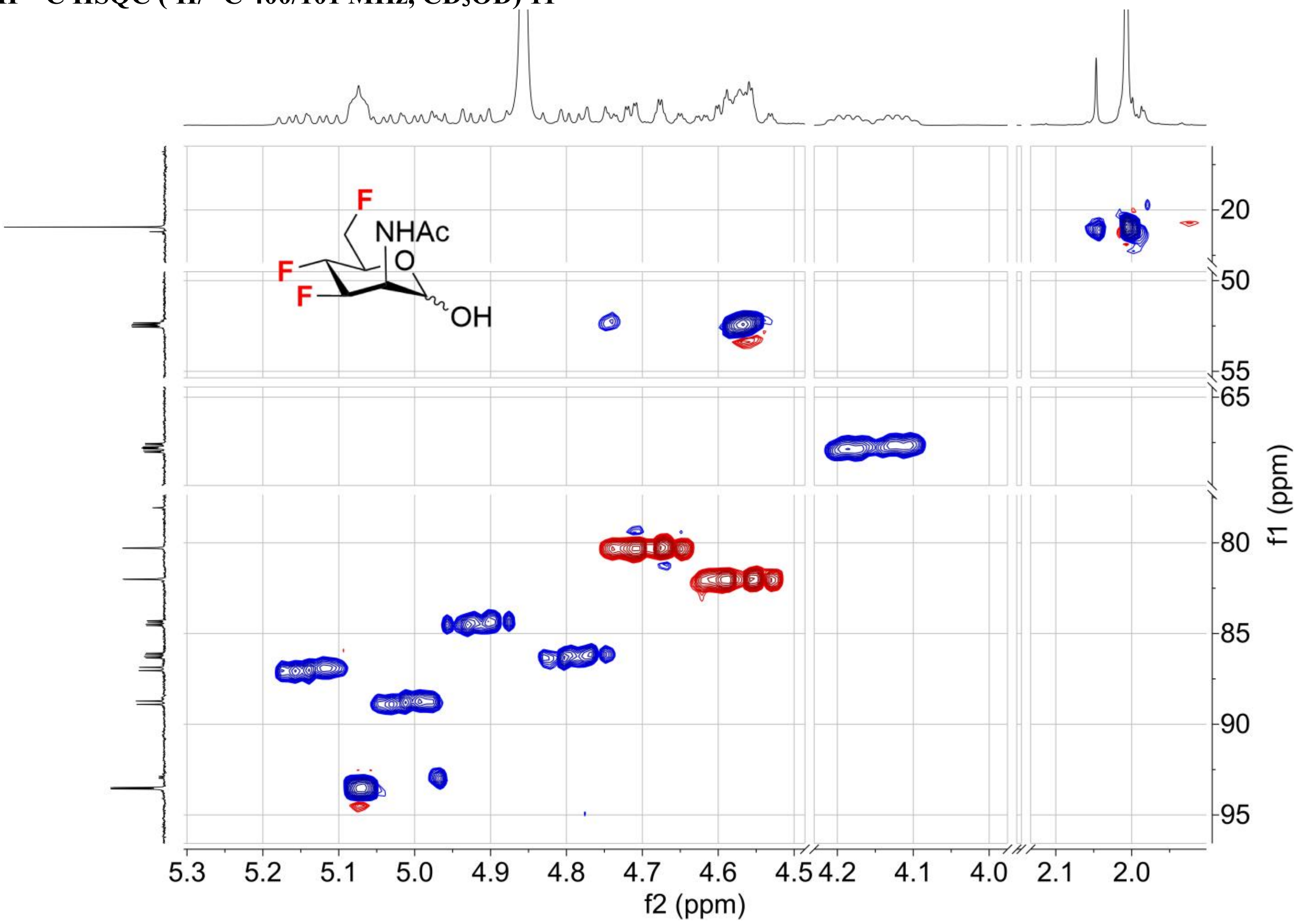

^1H-^{13}C HMBC (^1H/^{13}C 400/101 MHz, CD_3OD) 11

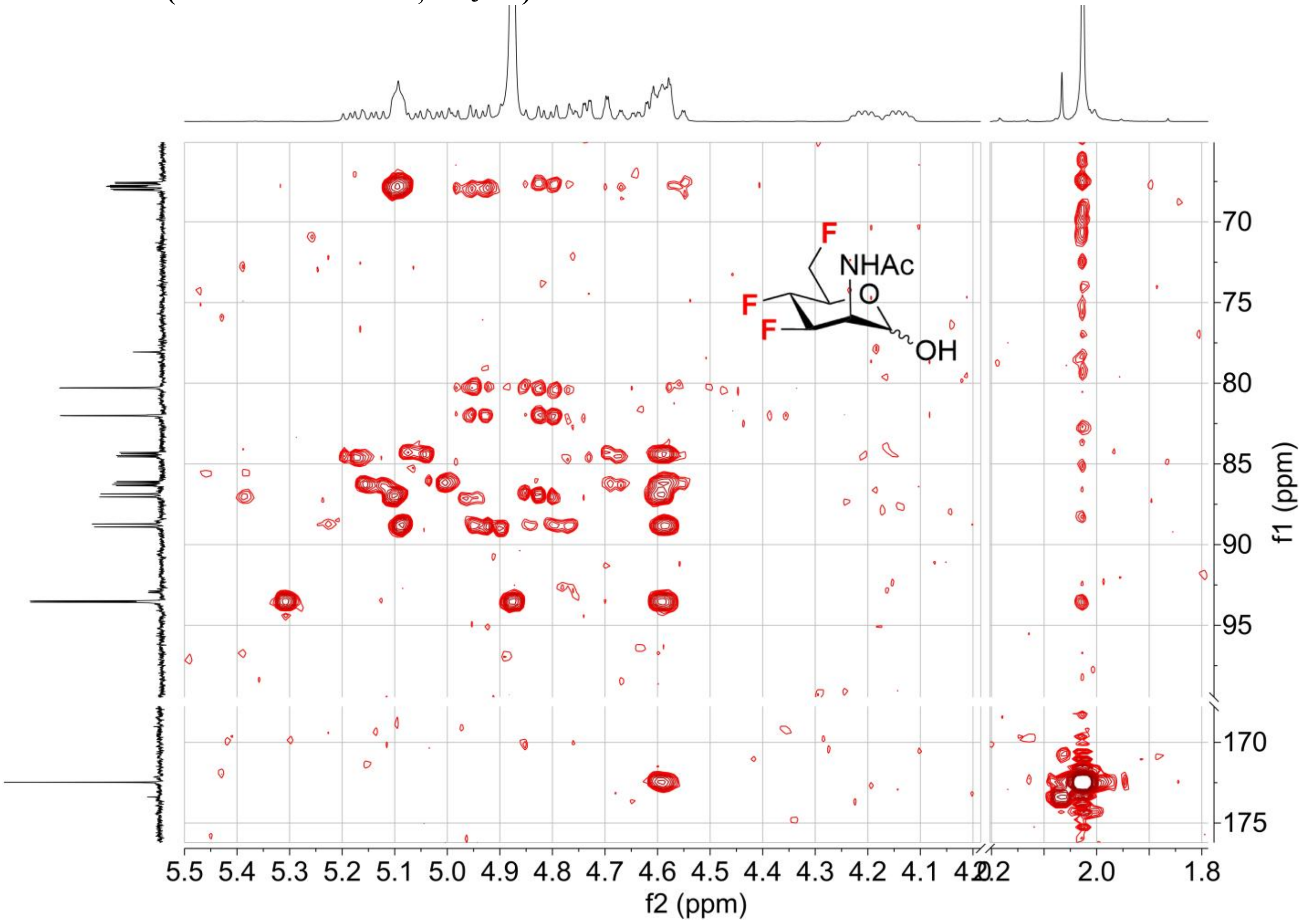

NMR COMPOUND 17 (containing ca 10% of compound 18)

^{1}H NMR (400 MHz, $CDCl_3$) 17 (containing ca 10% of compound 18)

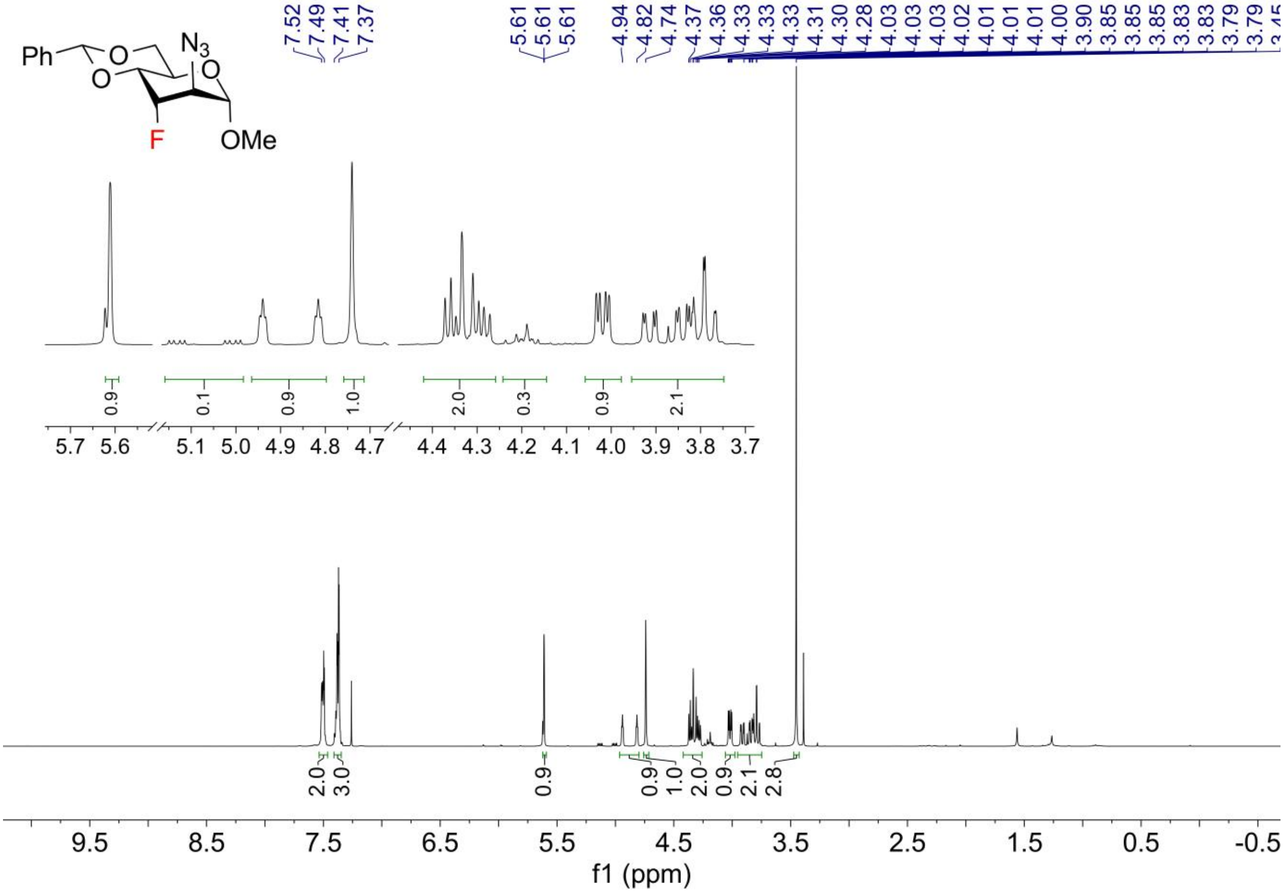

$^{13}C\{^{1}H\}$ NMR (101 MHz, $CDCl_3$) 17 (containing ca 10% of compound 18)

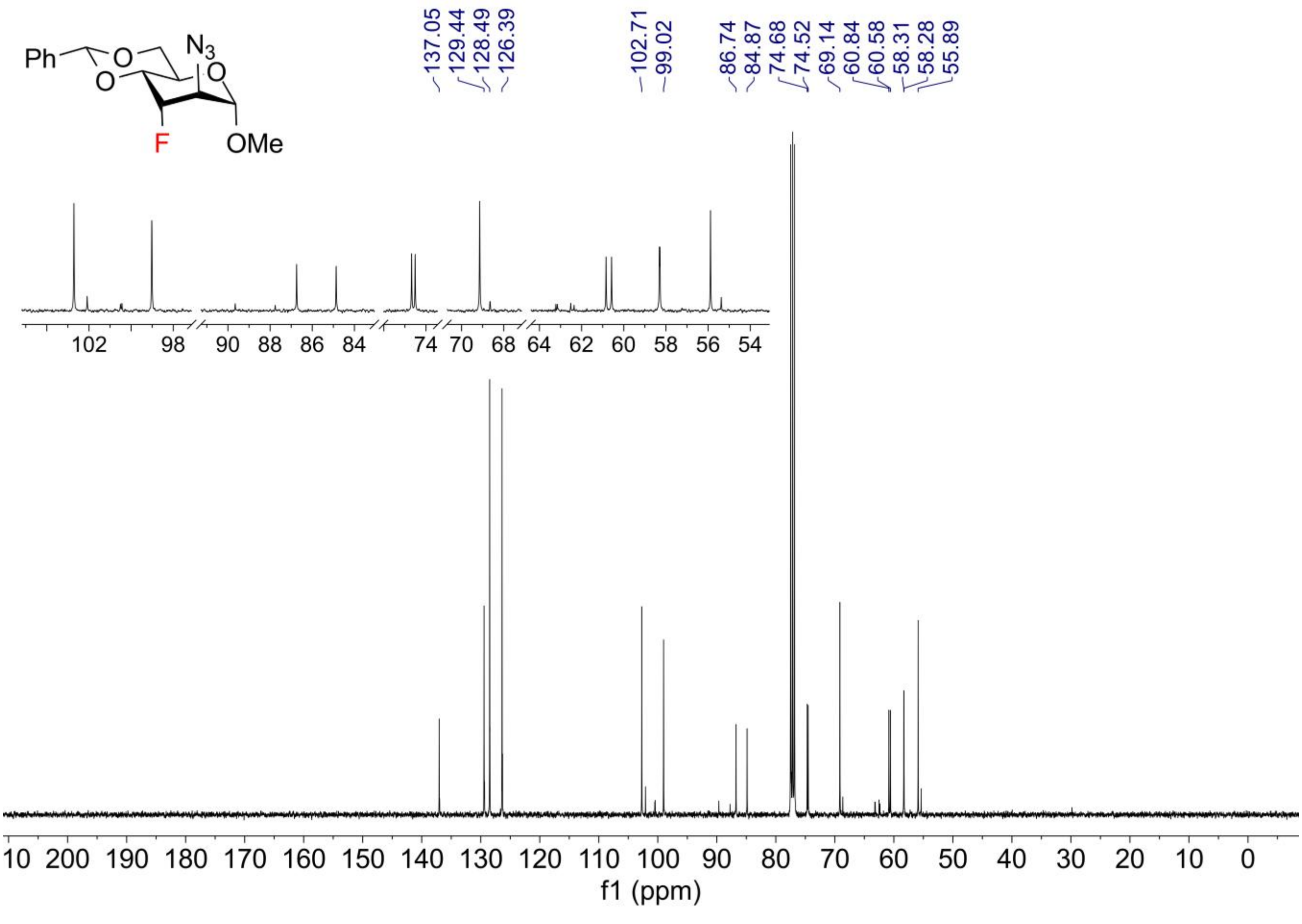

^{19}F NMR (376 MHz, $CDCl_3$) 17 (containing ca 10% of compound 18)

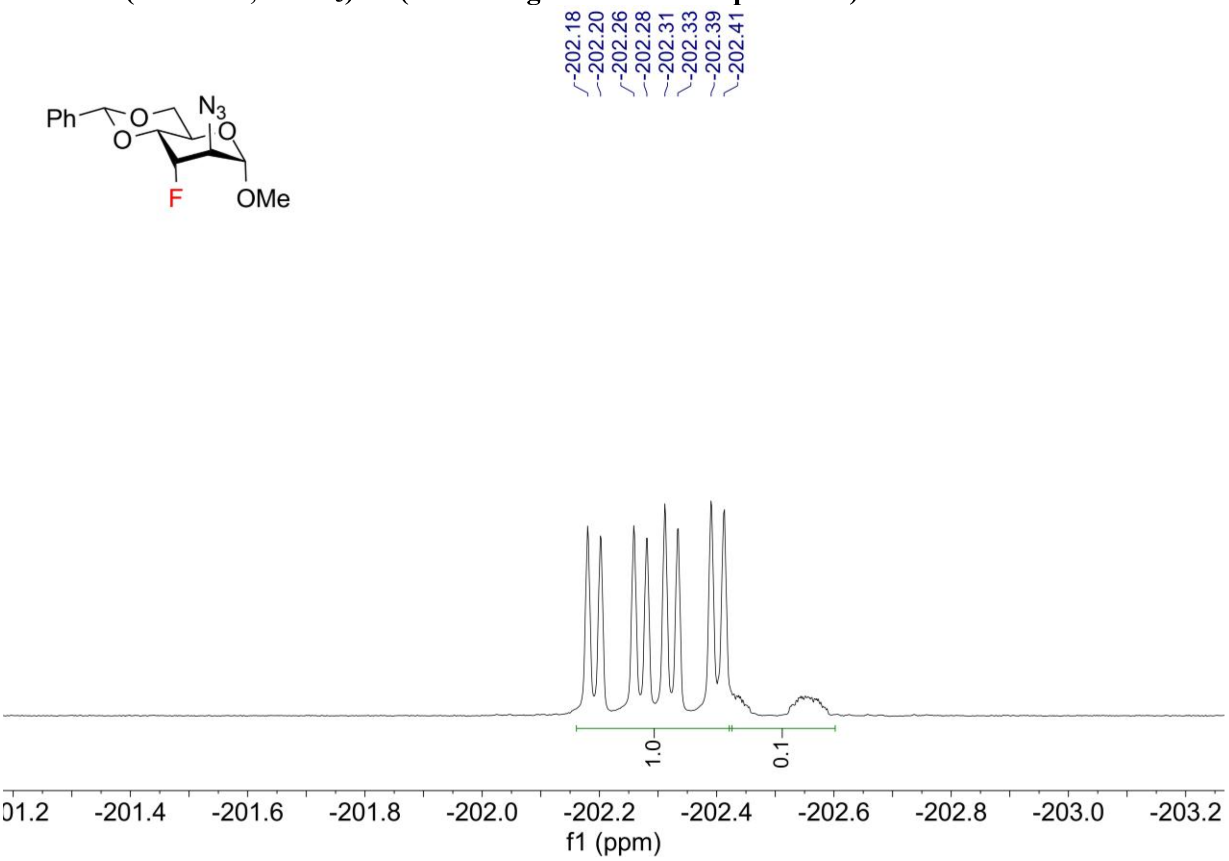

^{1}H-^{1}H COSY (400 MHz, $CDCl_3$) 17 (containing ca 10% of compound 18)

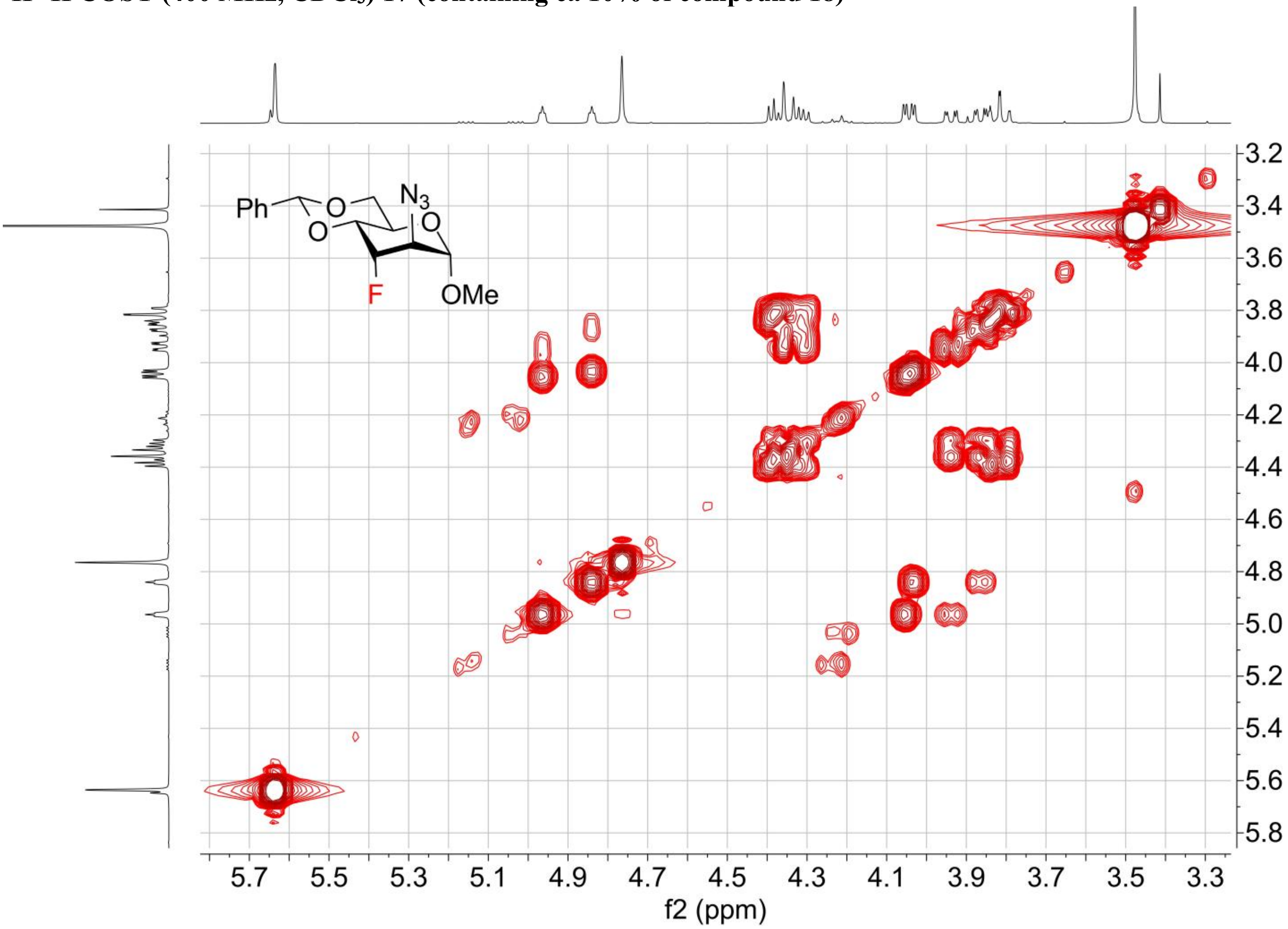

^{1}H-^{13}C HSQC ($^{1}H/^{13}C$ 400/101 MHz, $CDCl_3$) 17 (containing ca 10% of compound 18)

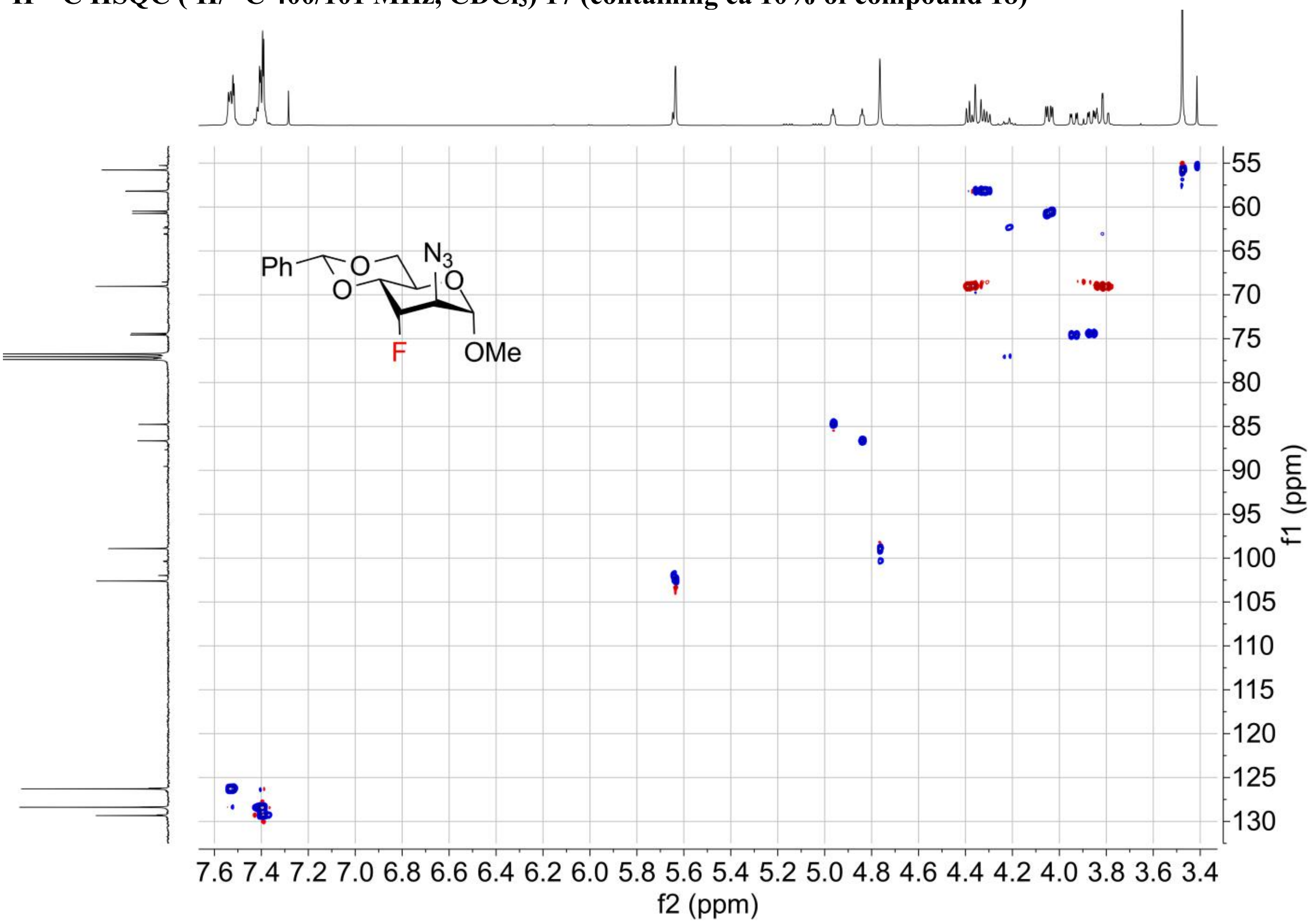

^{1}H-^{13}C HMBC ($^{1}H/^{13}C$ 400/101 MHz, $CDCl_3$) 17 (containing ca 10% of compound 18)

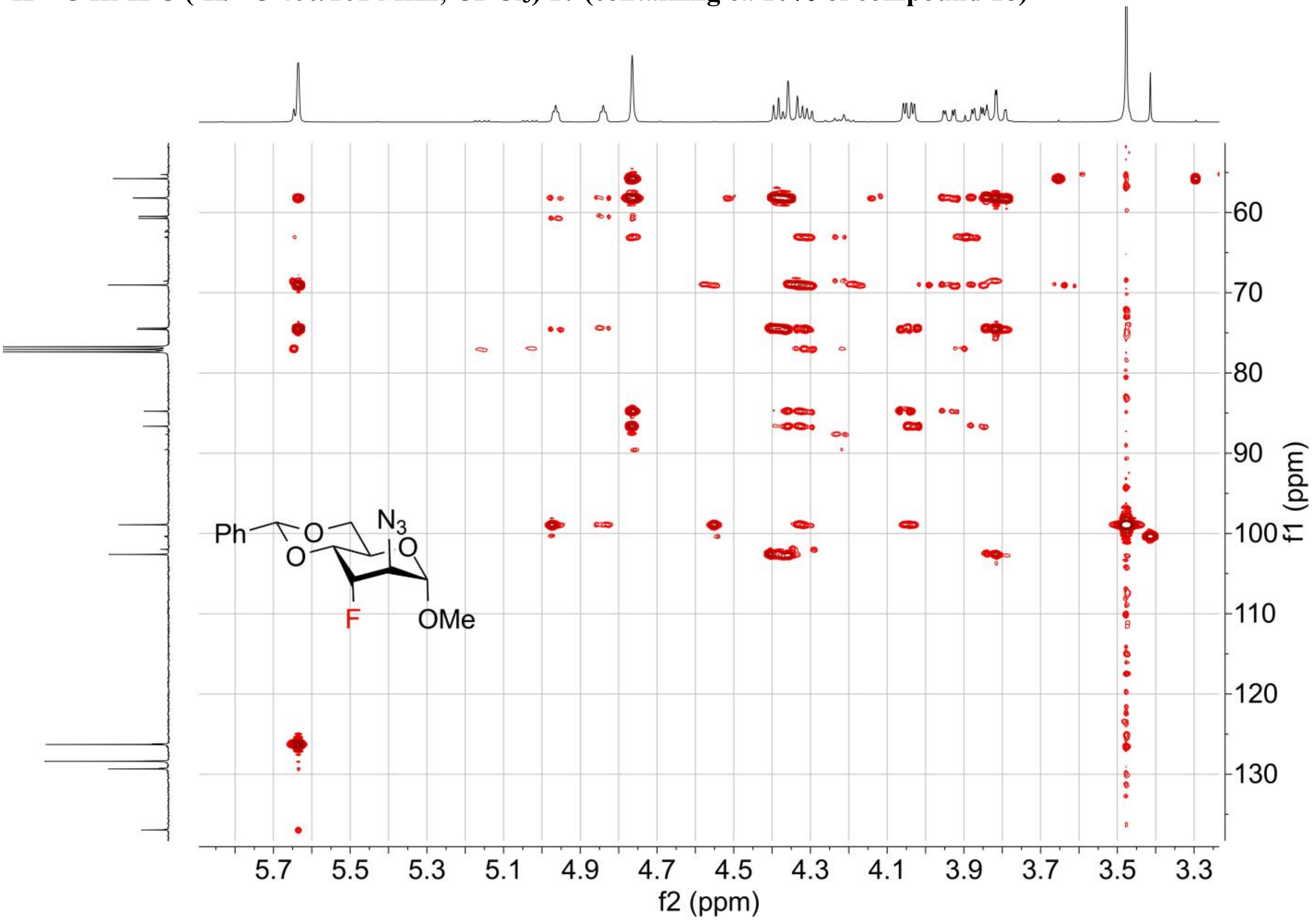

NMR COMPOUND 18

^{1}H NMR (400 MHz, CDCl$_3$) 18

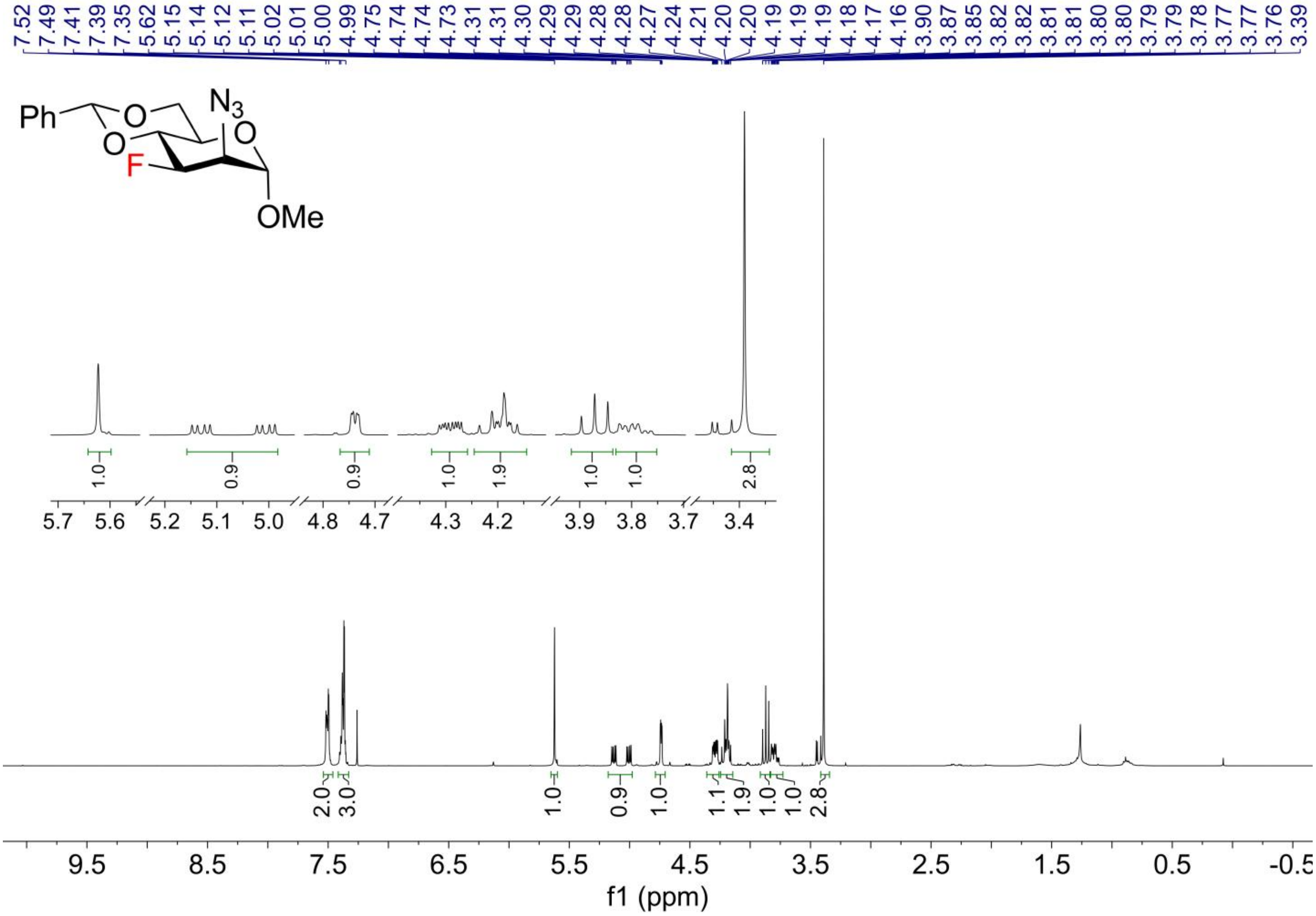

^{13}C{^{1}H} NMR (101 MHz, CDCl$_3$) 18

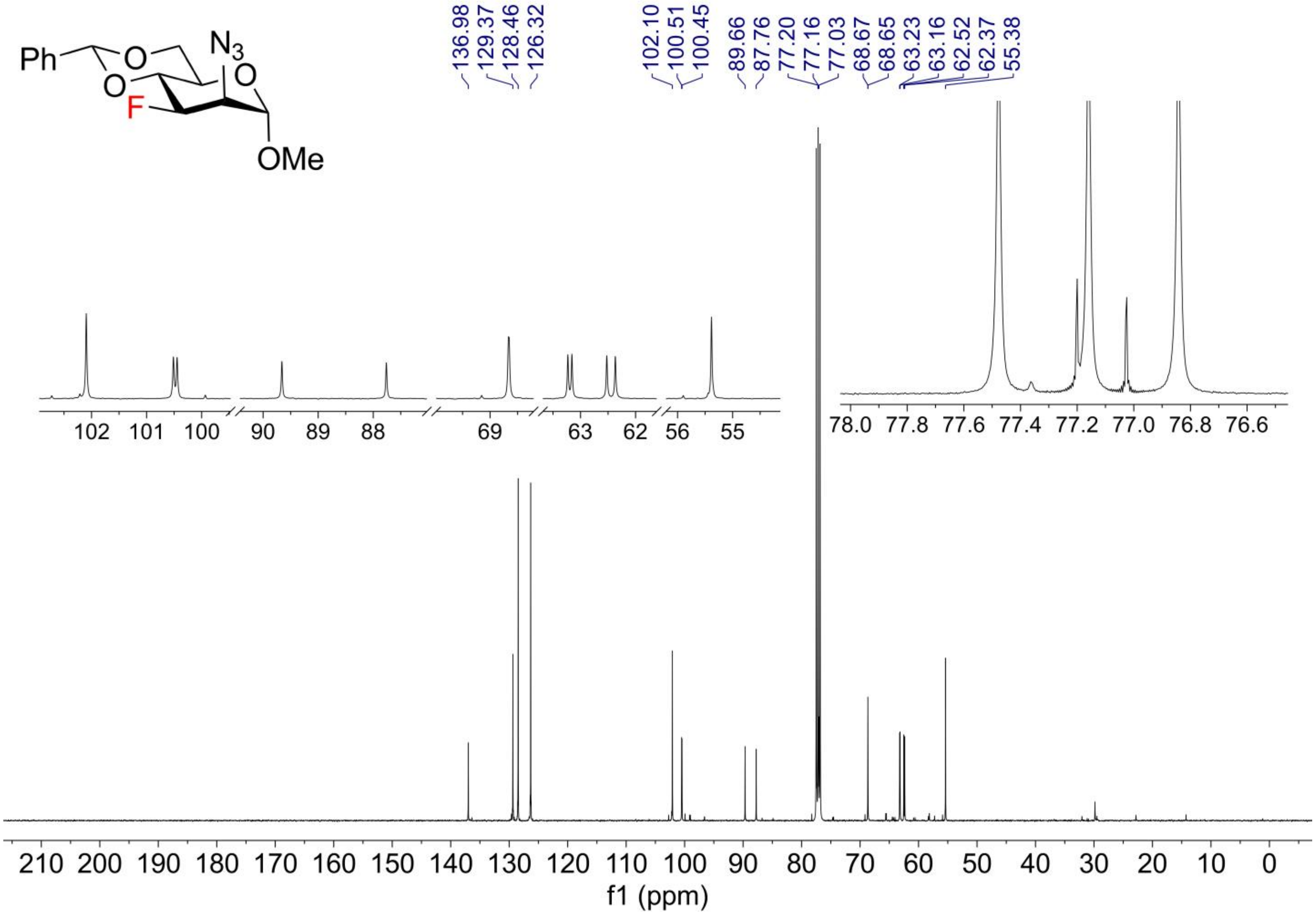

^{19}F NMR (376 MHz, CDCl$_3$) 18

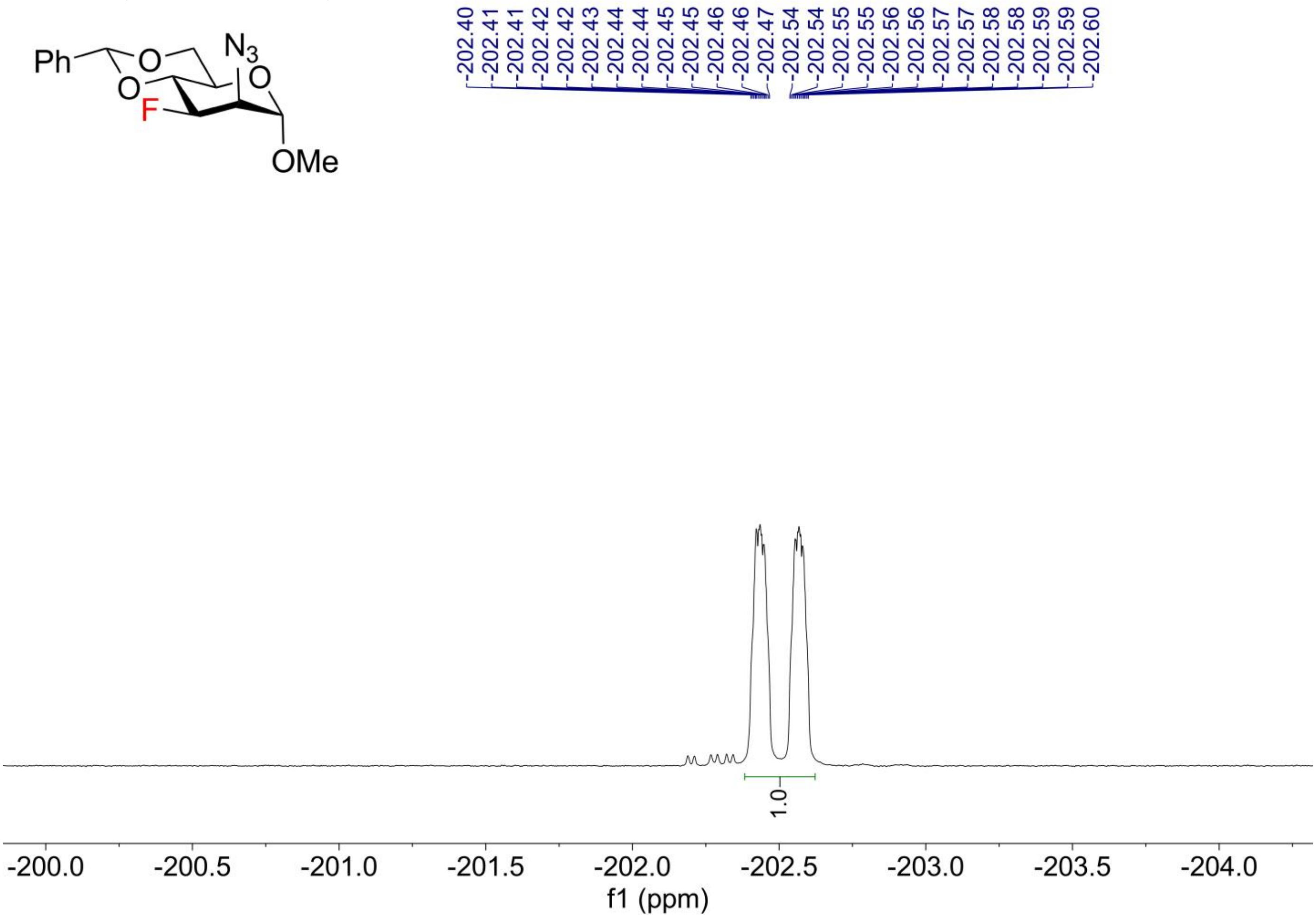

^{1}H-^{1}H COSY (400 MHz, CDCl$_3$) 18

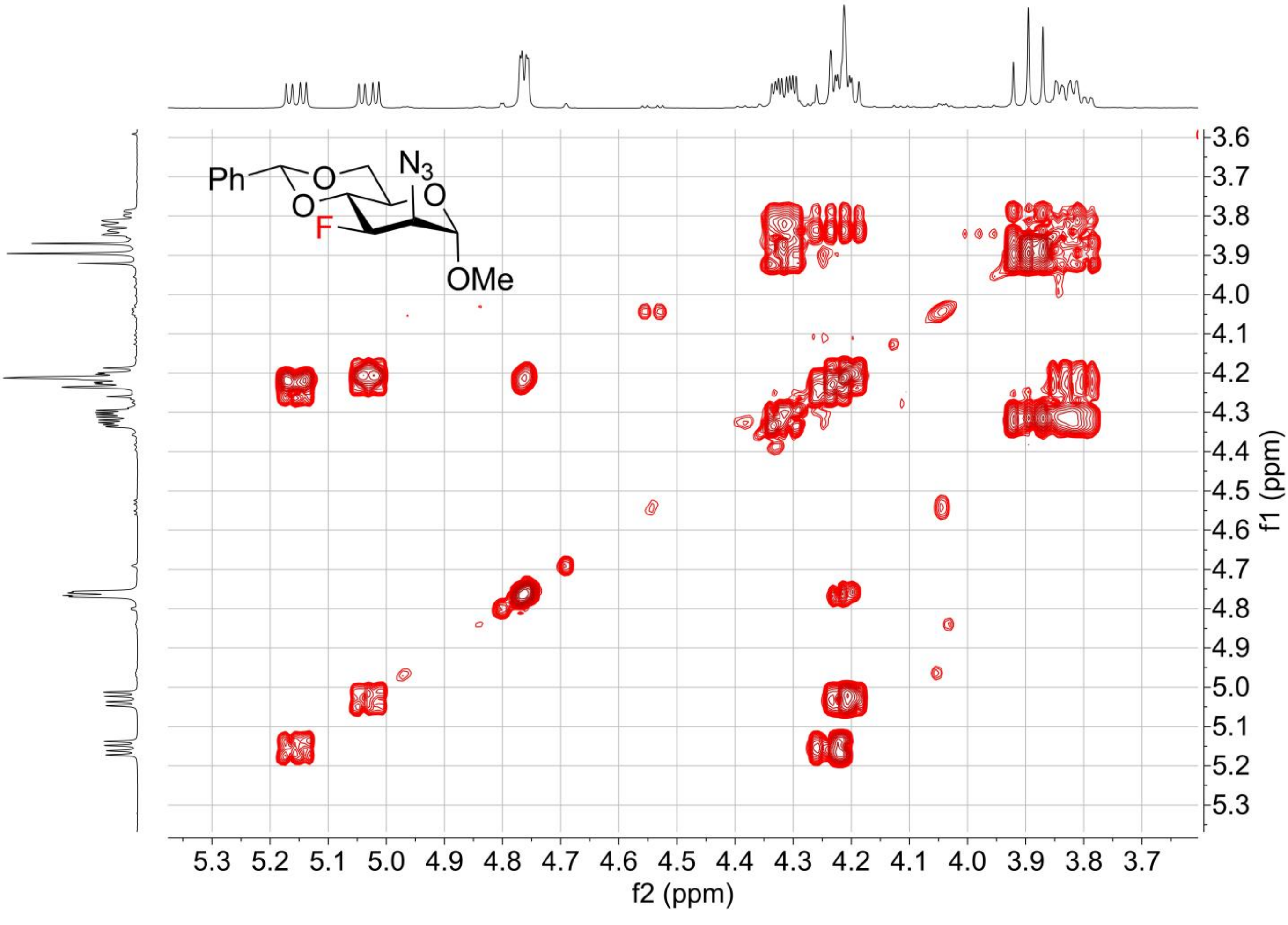

^{1}H-^{13}C HSQC (^{1}H/^{13}C 400/101 MHz, $CDCl_3$) 18

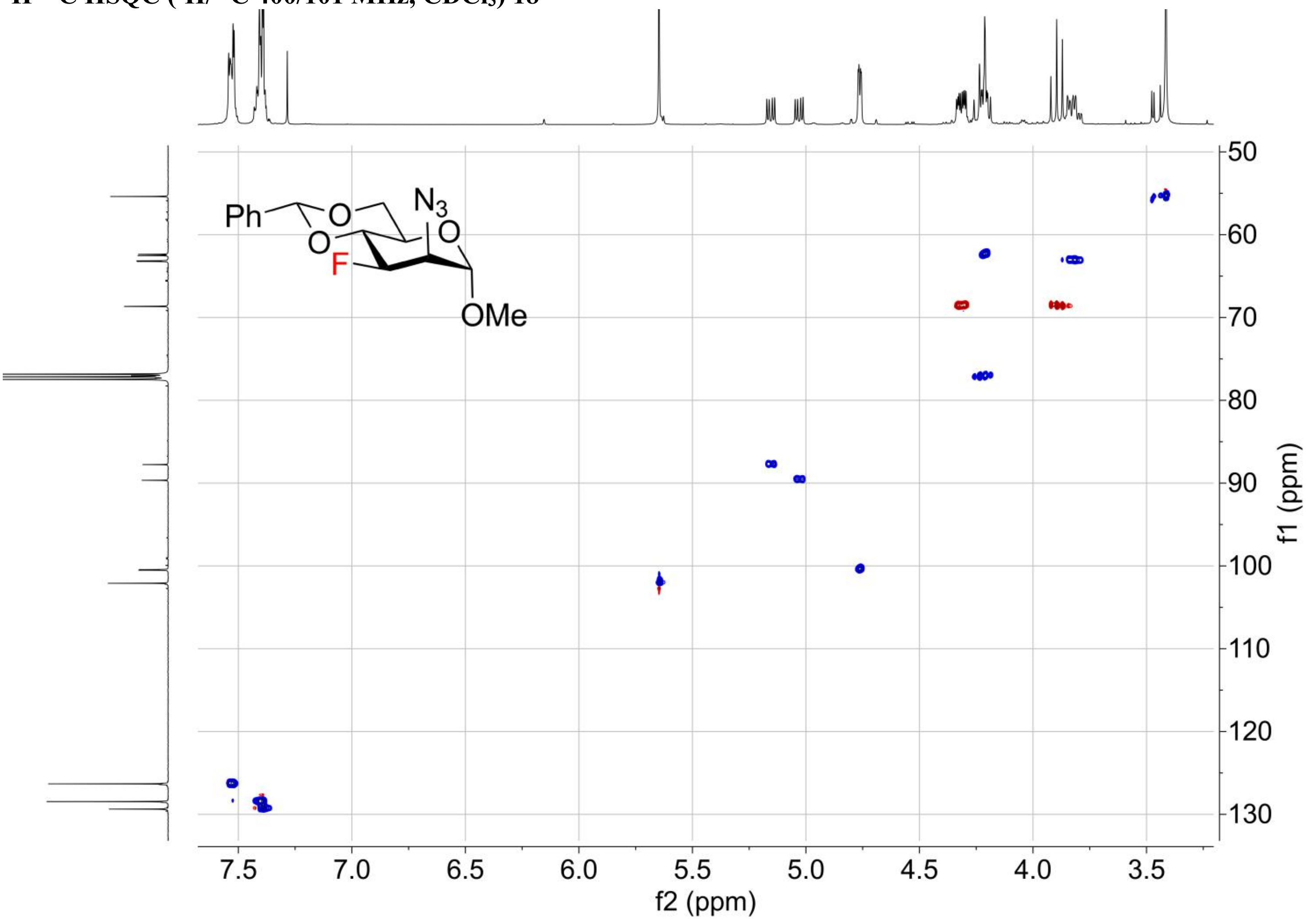

^{1}H-^{13}C HMBC (^{1}H/^{13}C 400/101 MHz, $CDCl_3$) 18

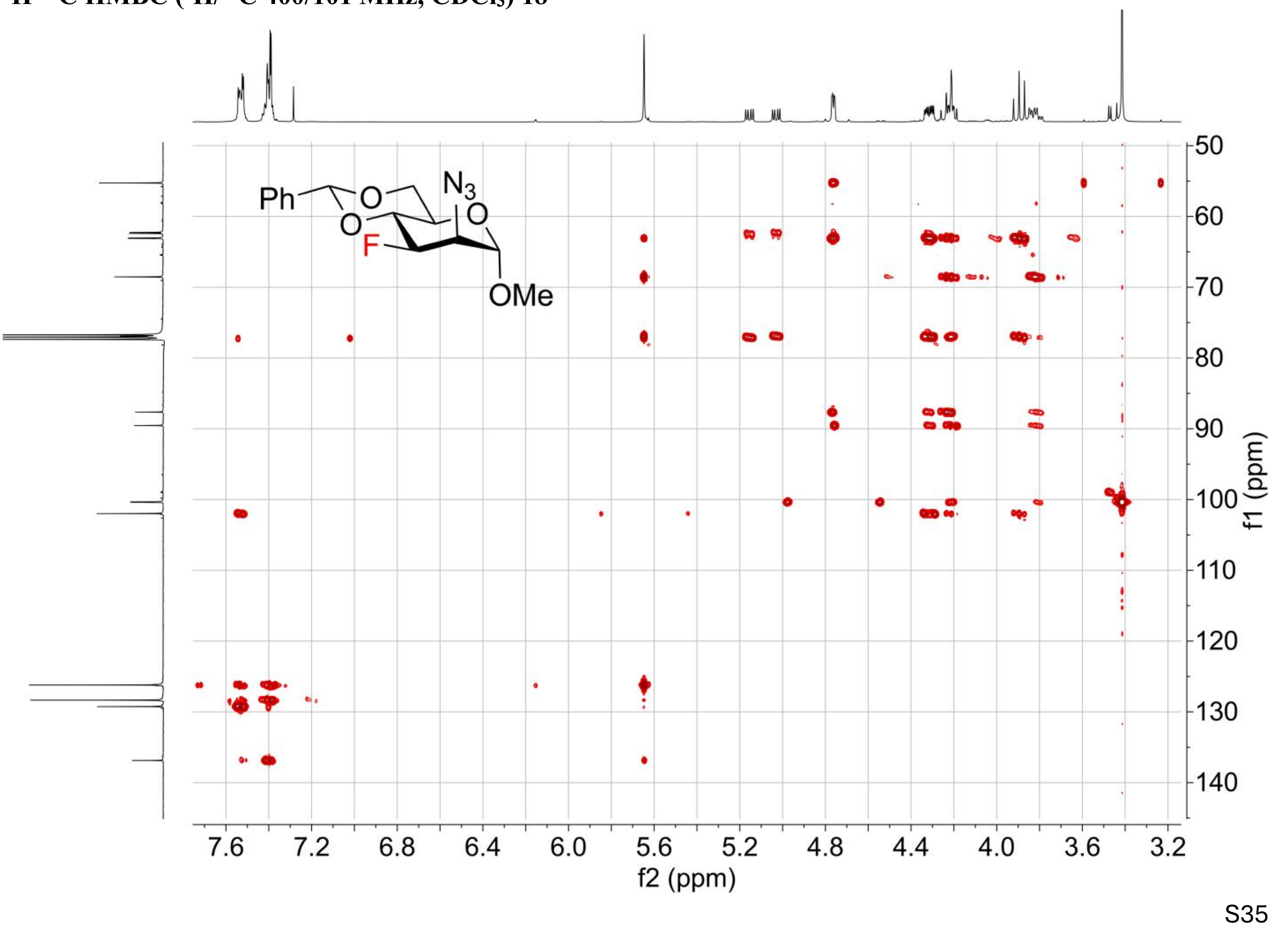

NMR COMPOUND 19

^{1}H NMR (400 MHz, $CDCl_3$) 19

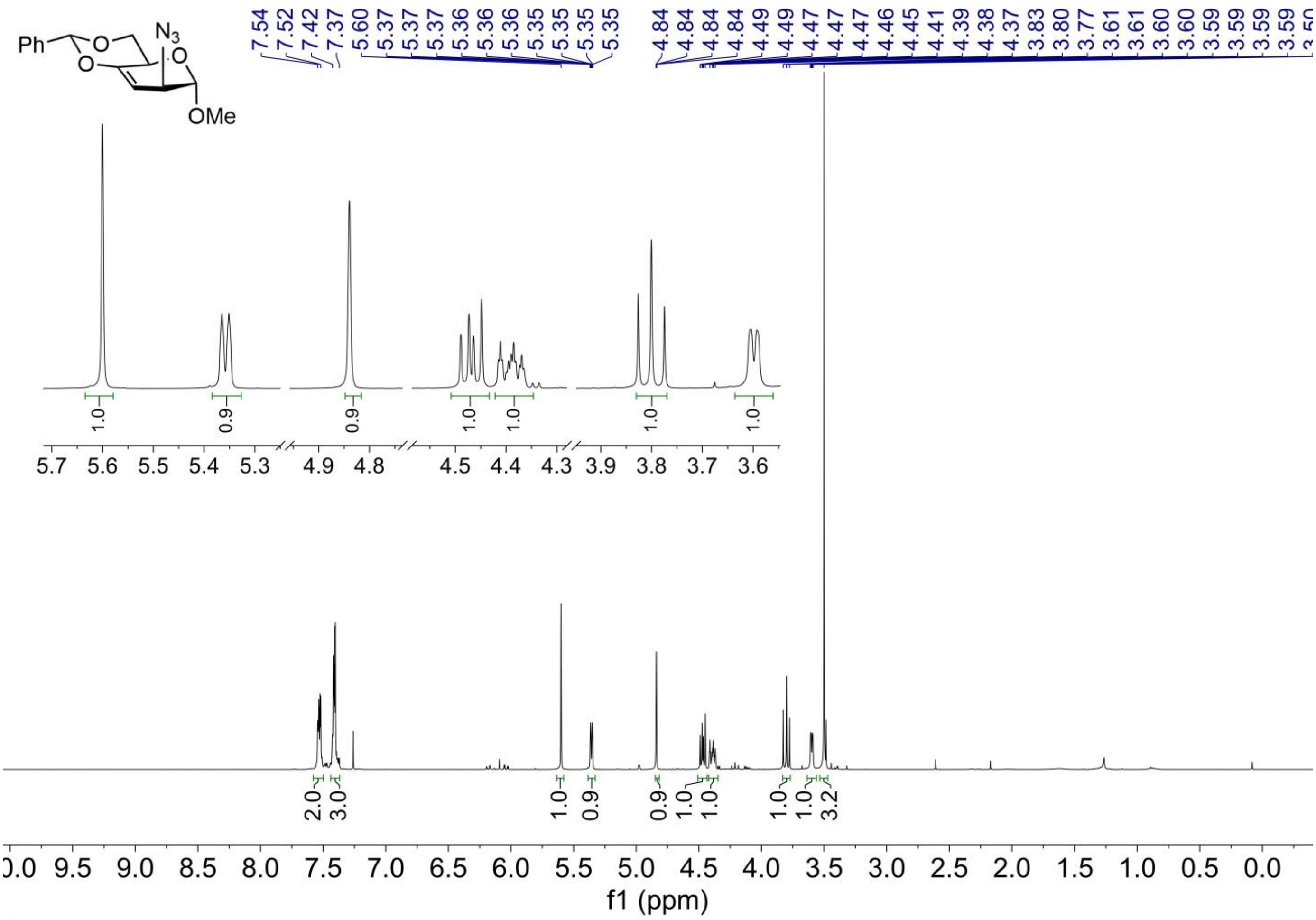

^{13}C{^{1}H} NMR (101 MHz, $CDCl_3$) 19

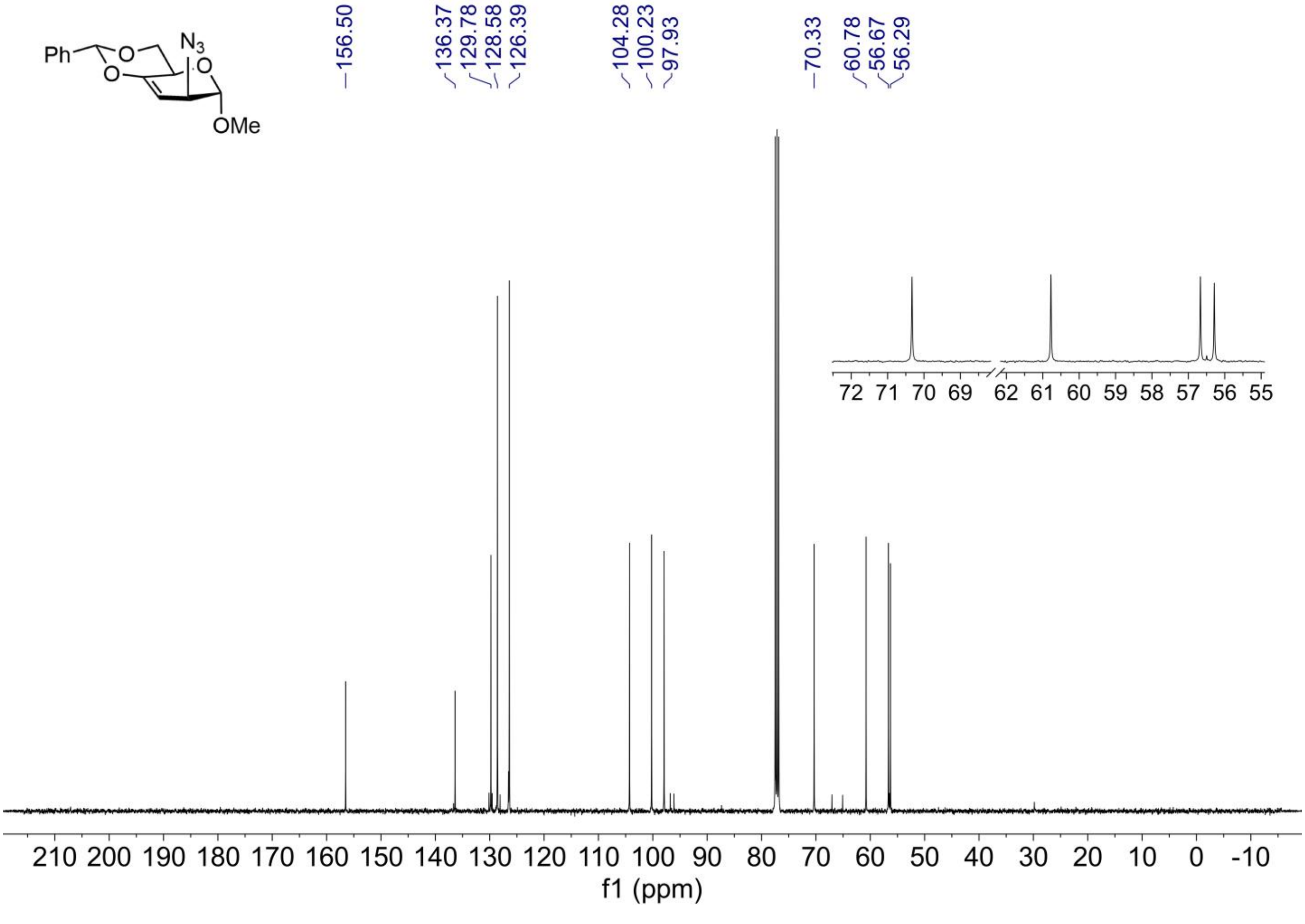

^{1}H-^{1}H COSY (400 MHz, $CDCl_3$) 19

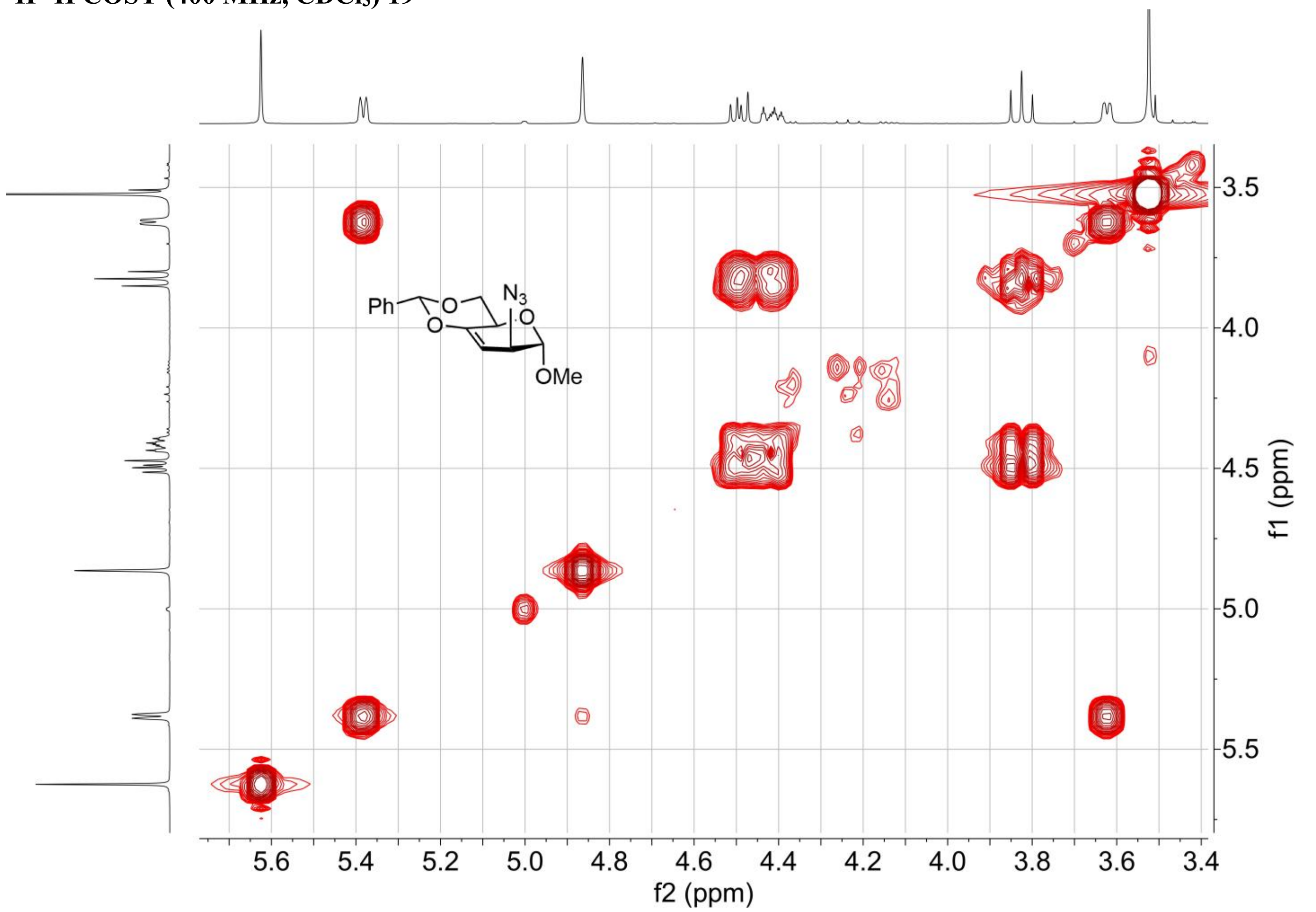

^{1}H-^{13}C HSQC (^{1}H/^{13}C 400/101 MHz, $CDCl_3$) 19

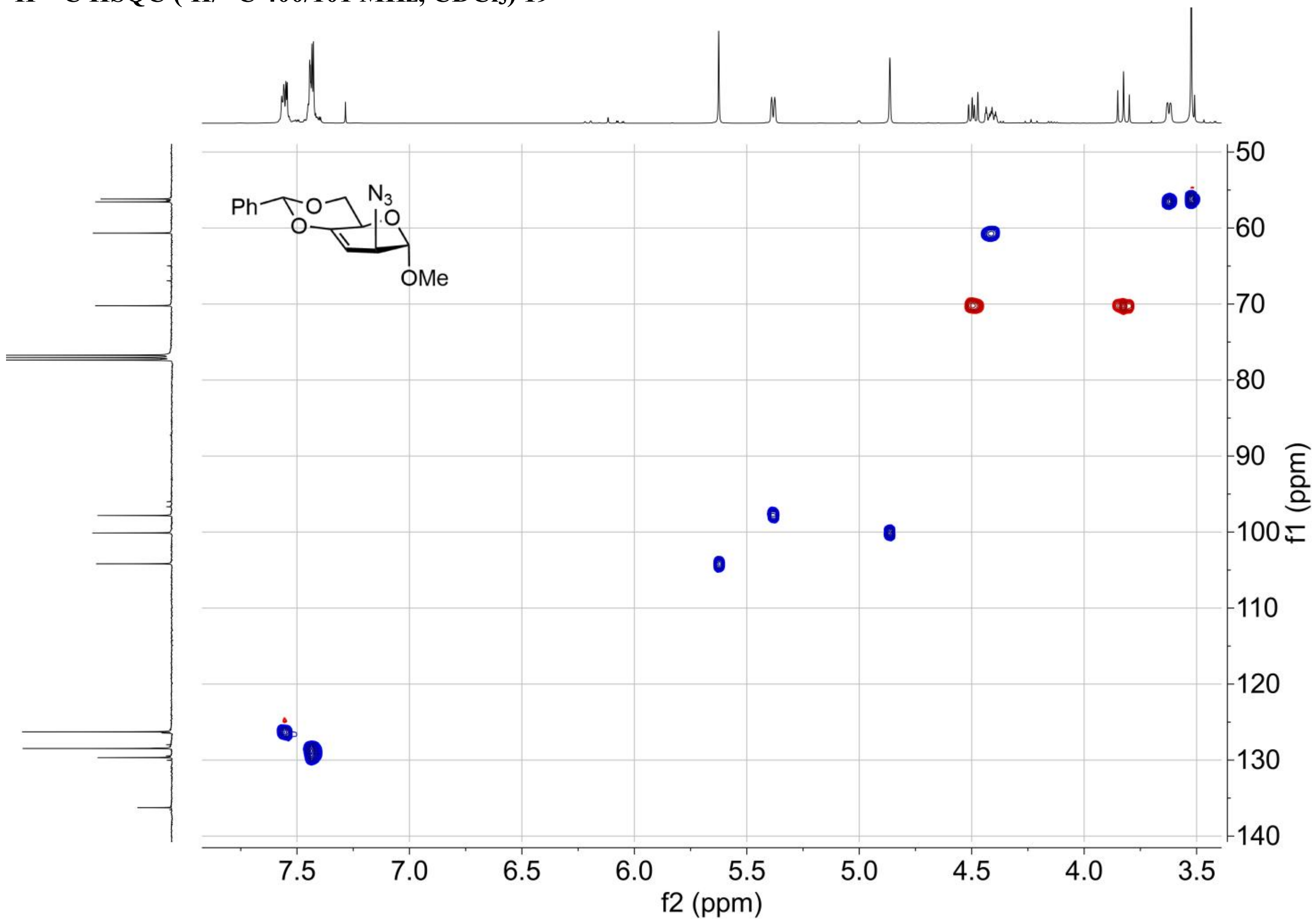

^{1}H-^{13}C HMBC (^{1}H/^{13}C 400/101 MHz, $CDCl_3$) 19

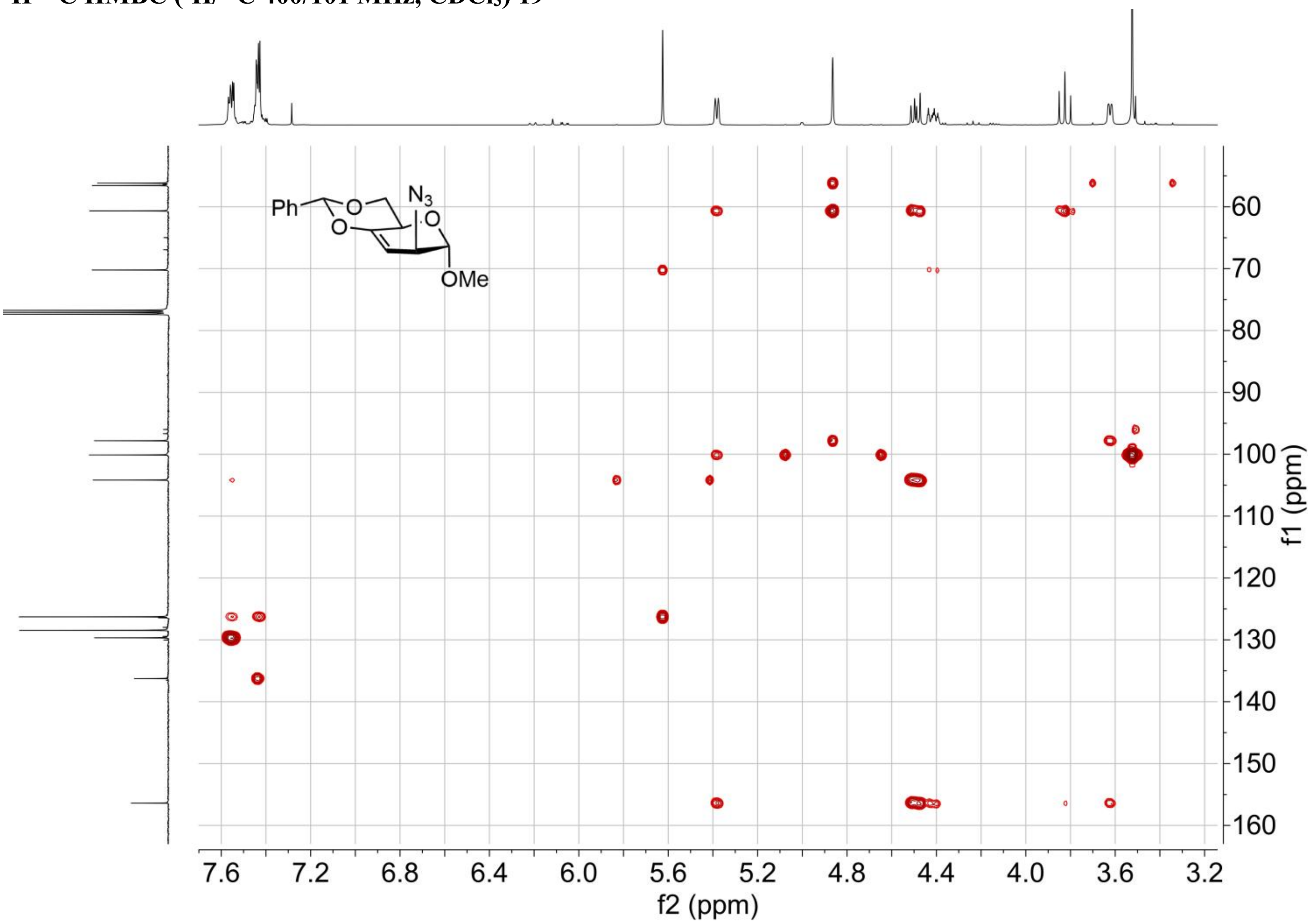

NMR COMPOUND 21

^{1}H NMR (400 MHz, $CDCl_3$) 21

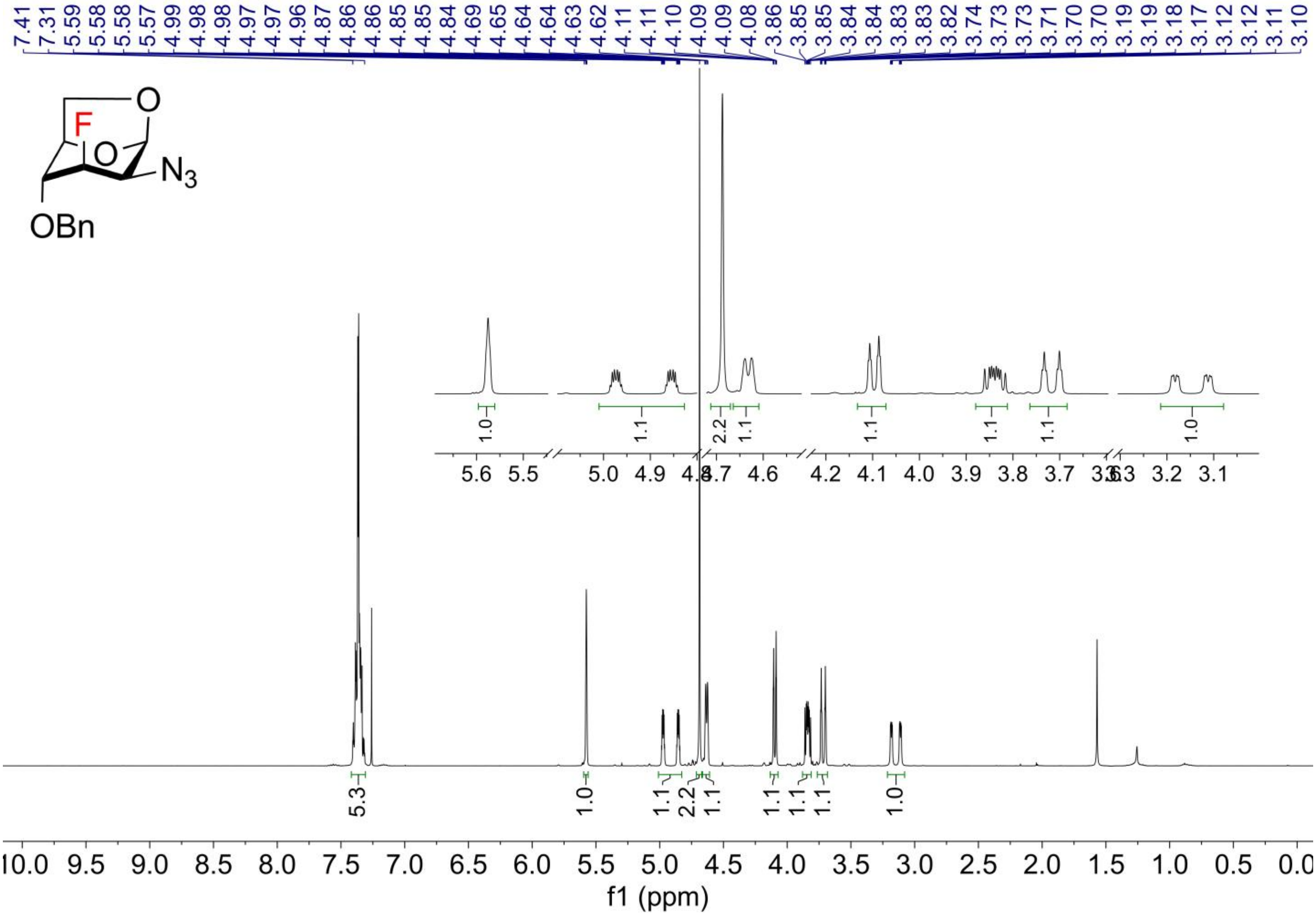

^{13}C{^{1}H} NMR (101 MHz, $CDCl_3$) 21

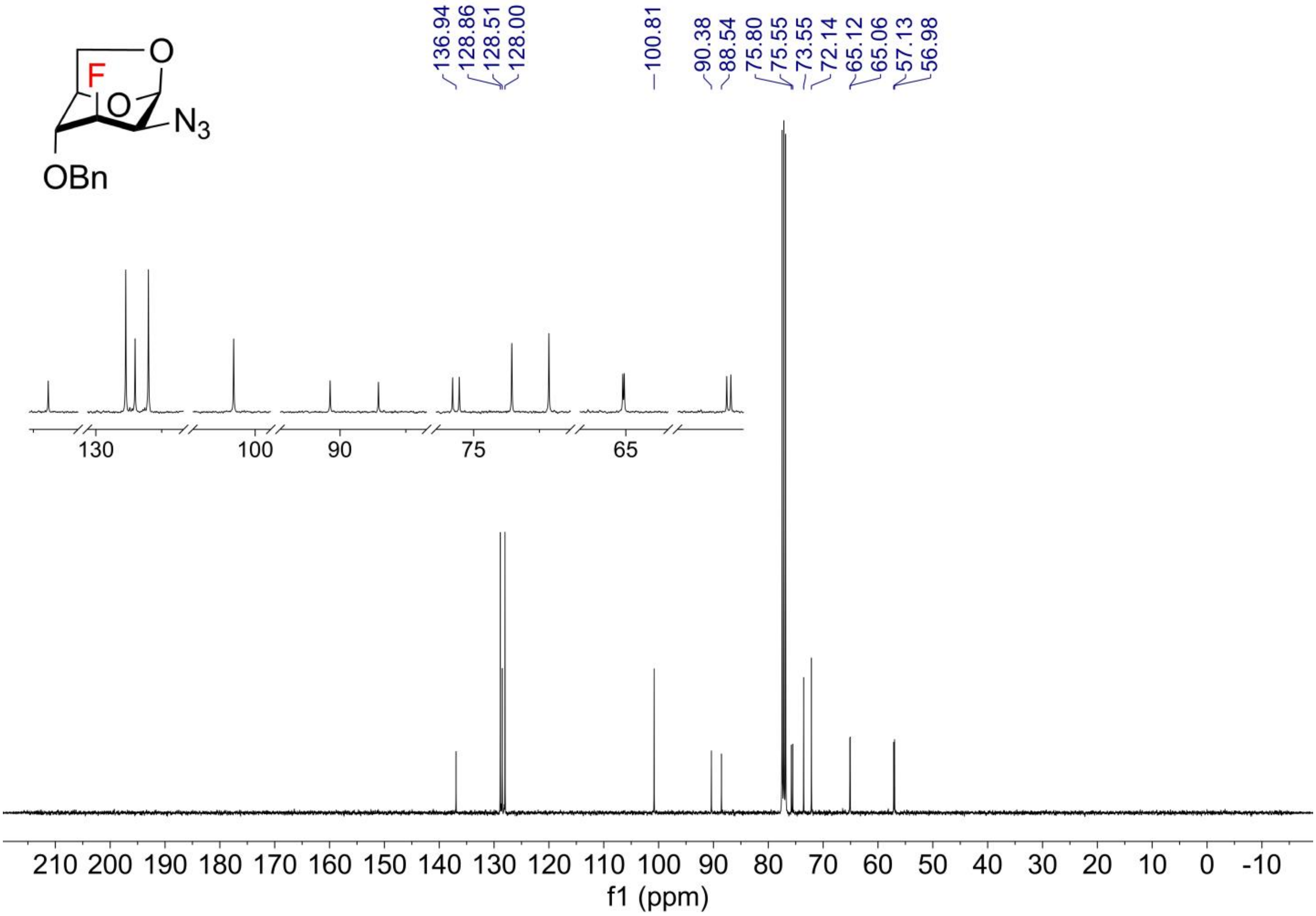

^{19}F NMR (376 MHz, $CDCl_3$) 21

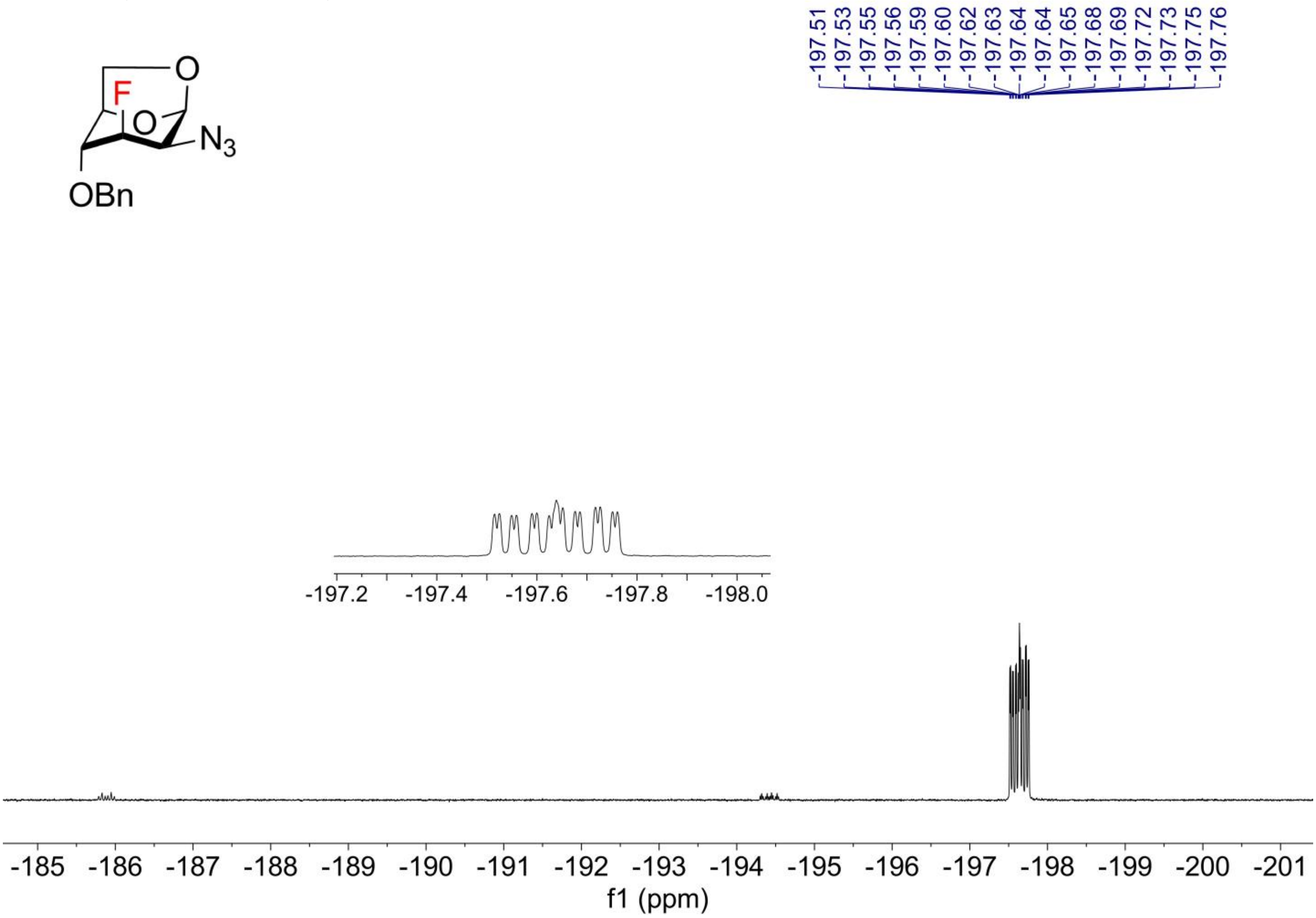

^{1}H-^{1}H COSY (400 MHz, $CDCl_3$) 21

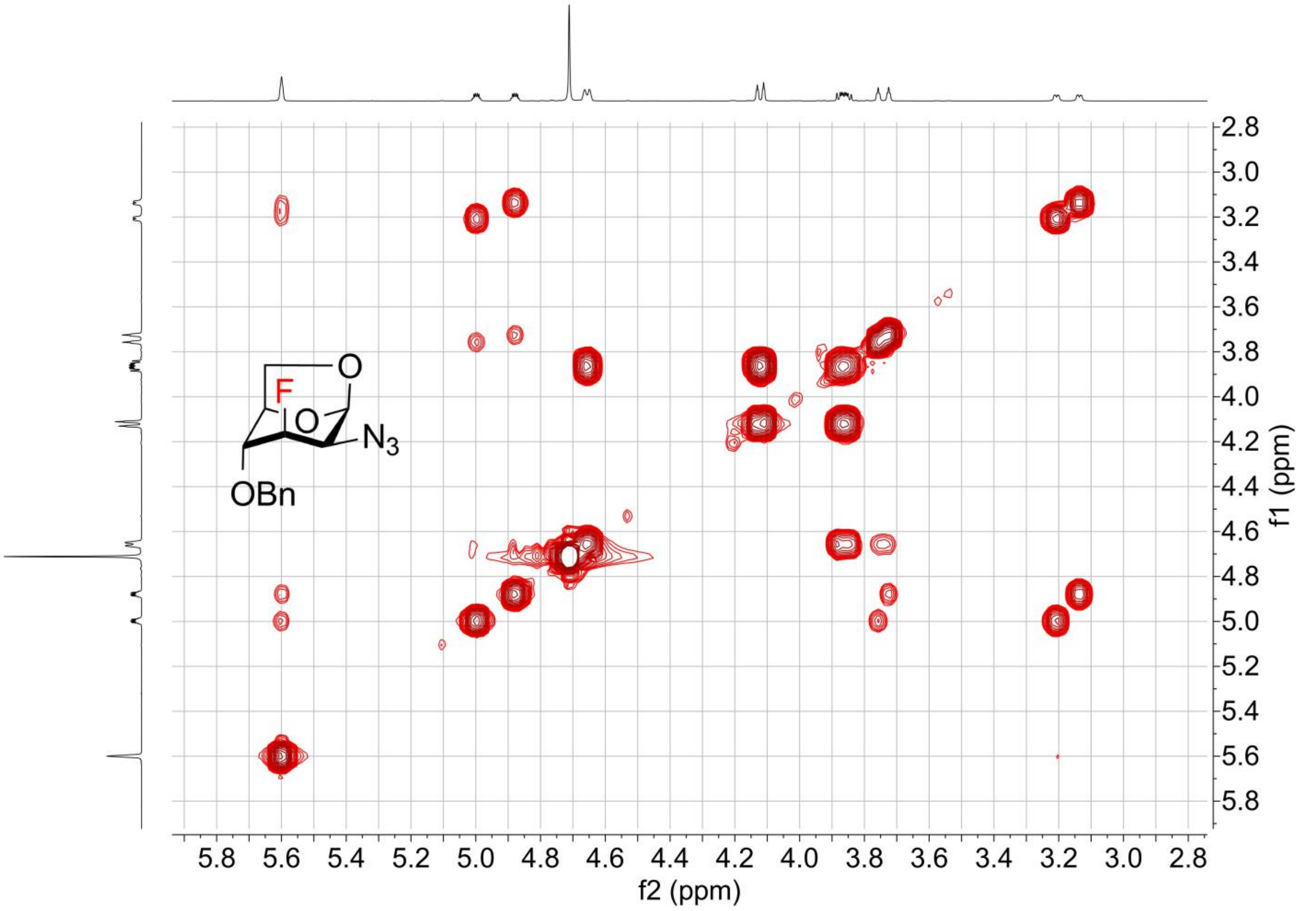

^{1}H-^{13}C HSQC ($^{1}H/^{13}C$ 400/101 MHz, $CDCl_3$) 21

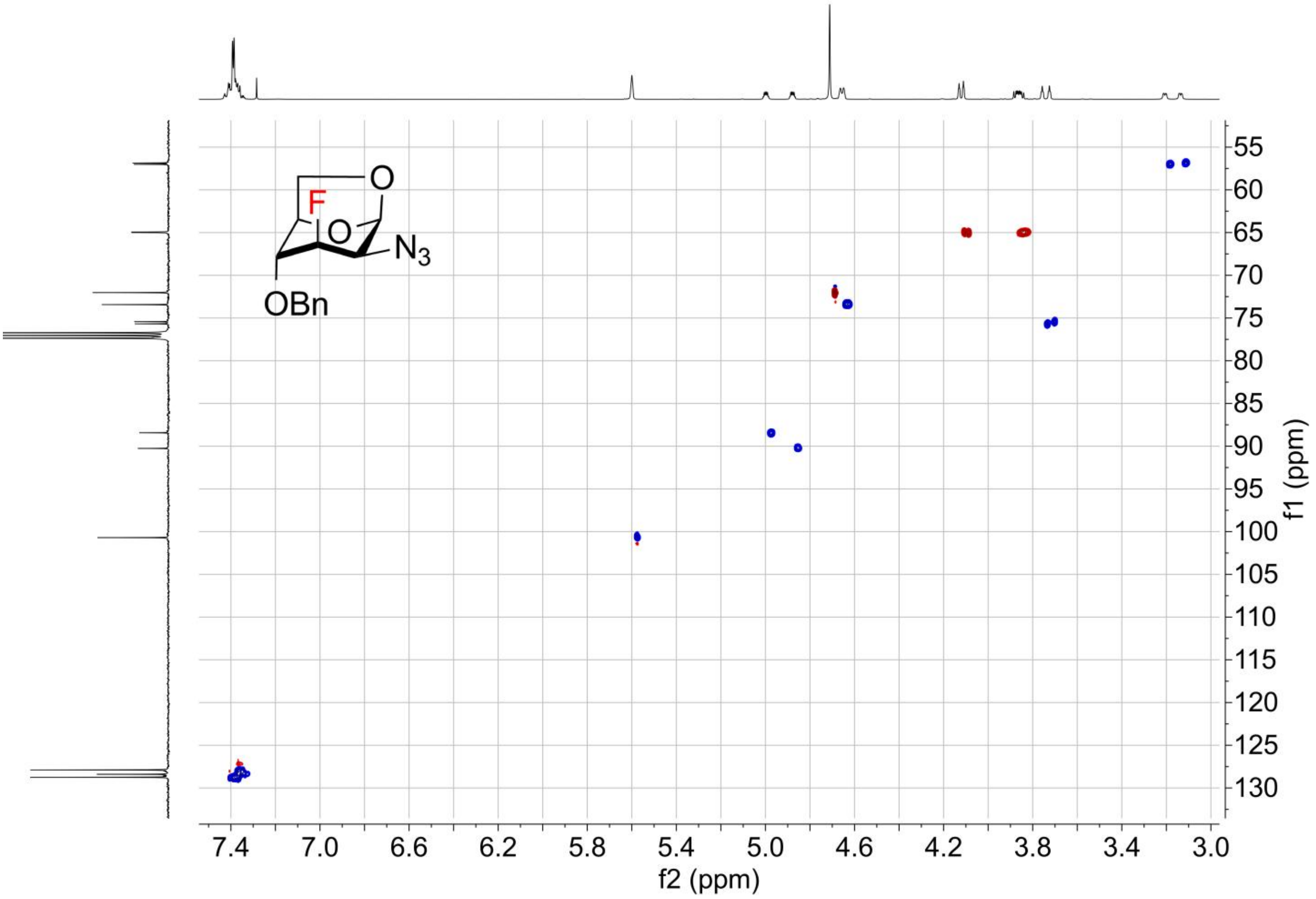

^{1}H-^{13}C HMBC ($^{1}H/^{13}C$ 400/101 MHz, $CDCl_3$) 21

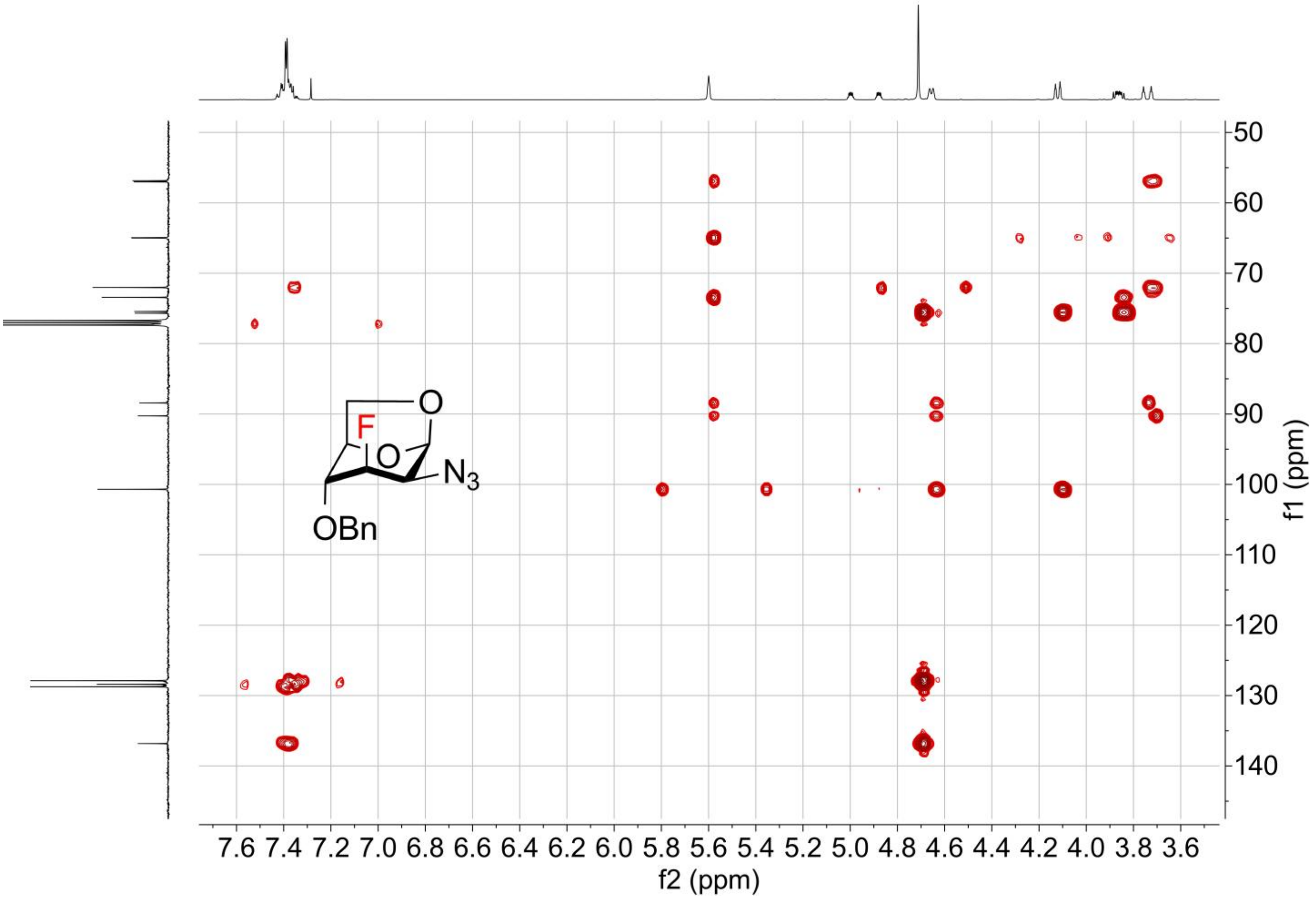

NMR COMPOUND 22

^{1}H NMR (400 MHz, CDCl$_3$) 22

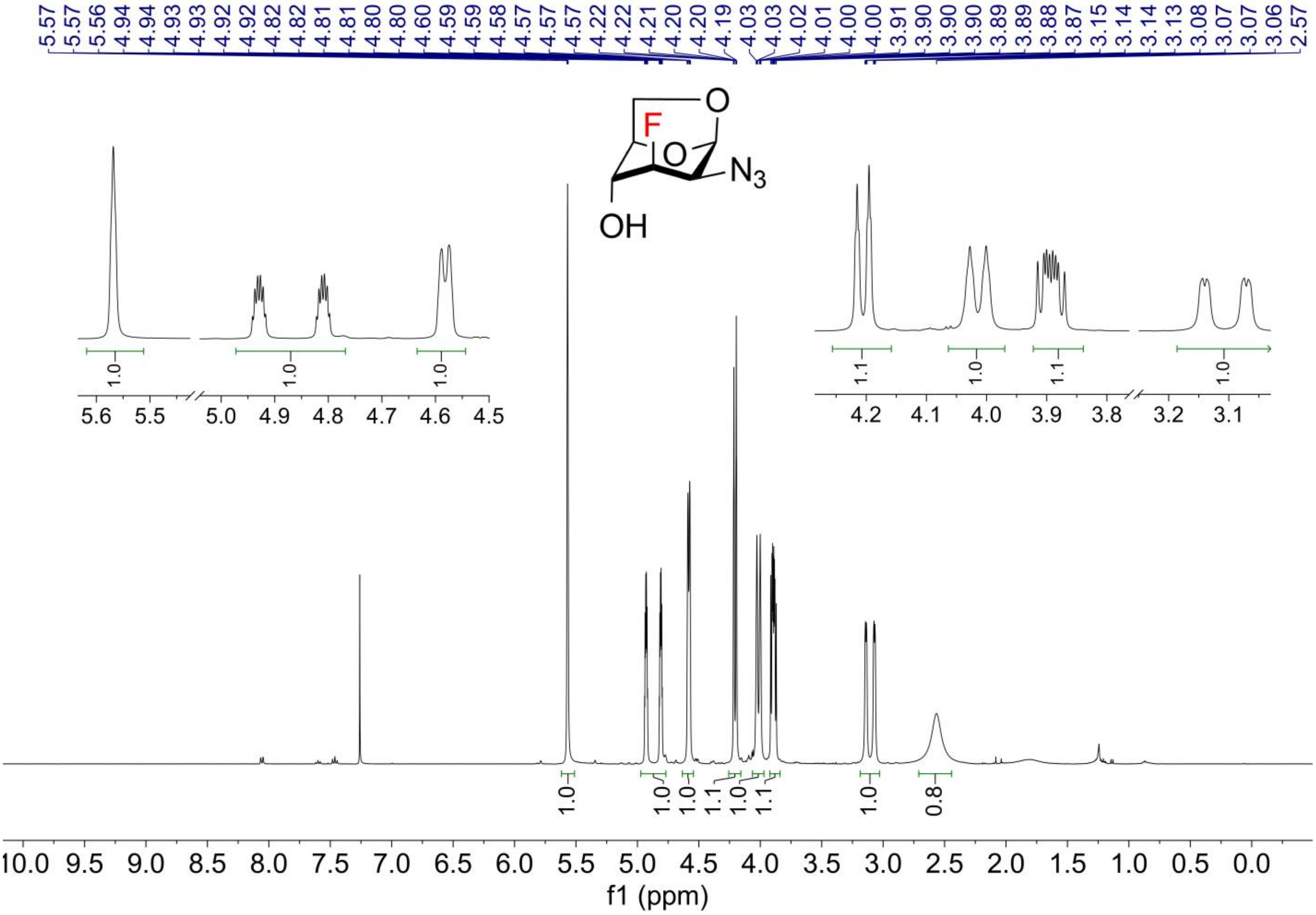

^{13}C{^{1}H}NMR (101 MHz, CDCl$_3$) 22

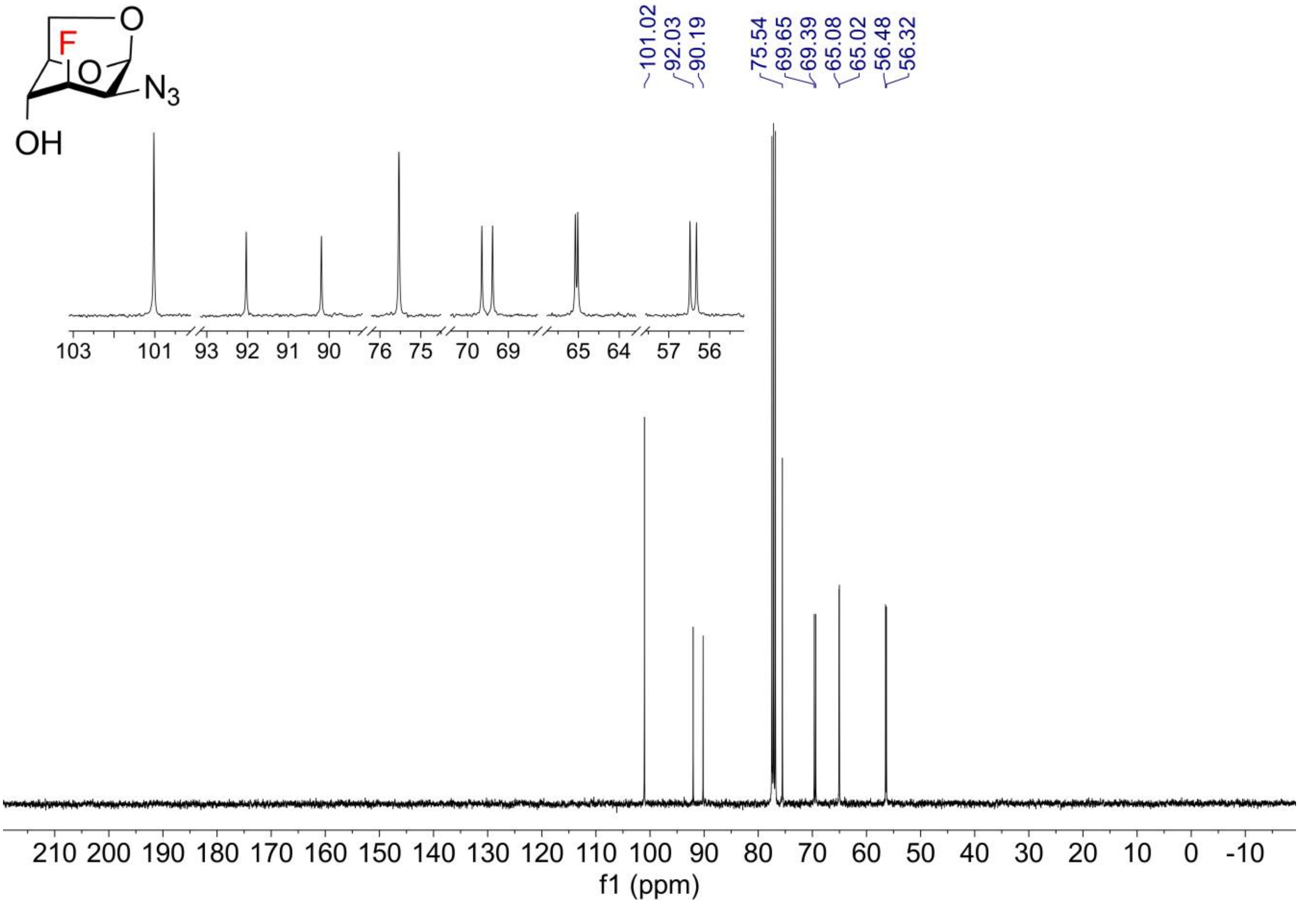

^{19}F NMR (376 MHz, CDCl₃) 22

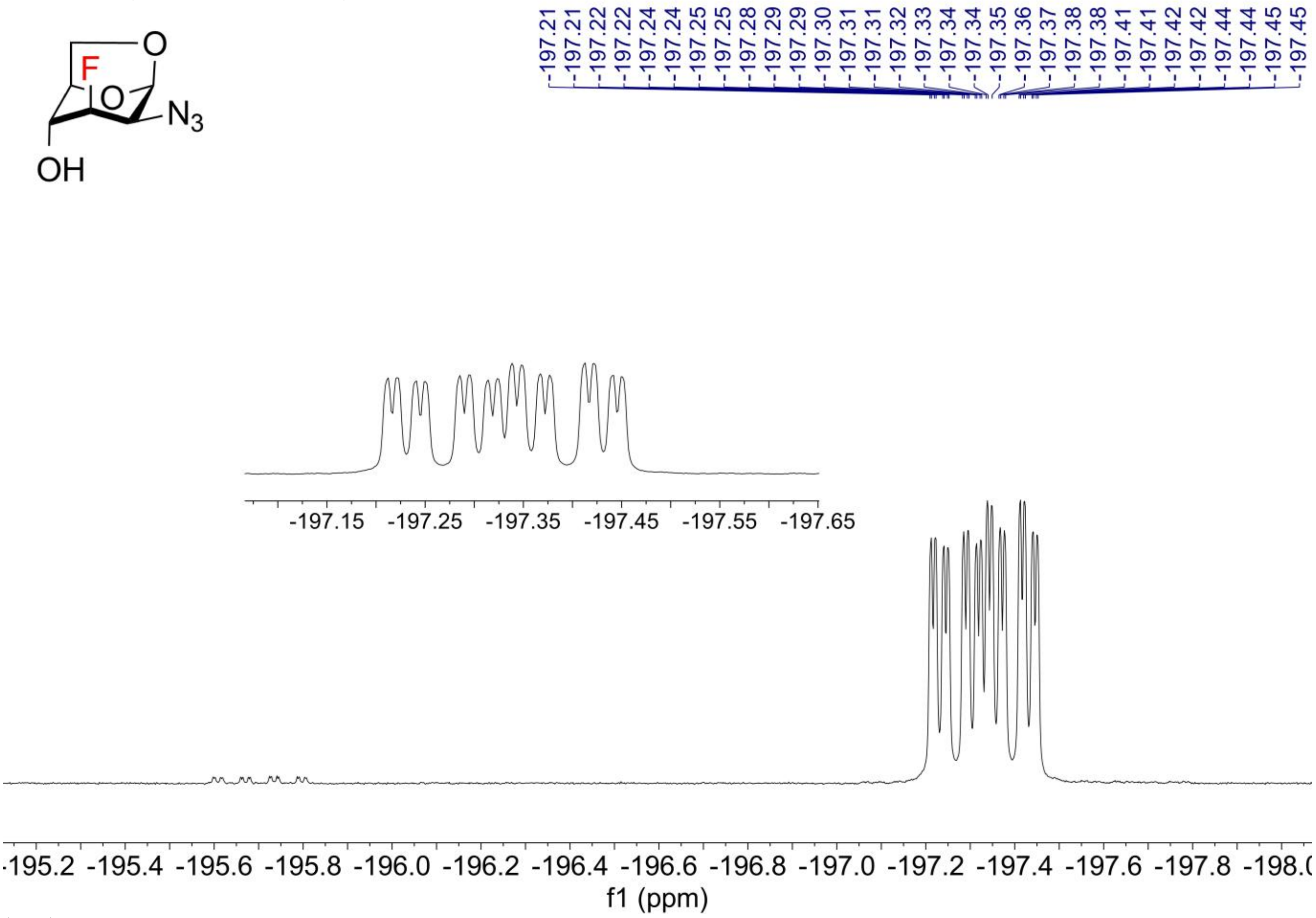

^{1}H-^{1}H COSY (400 MHz, CDCl₃) 22

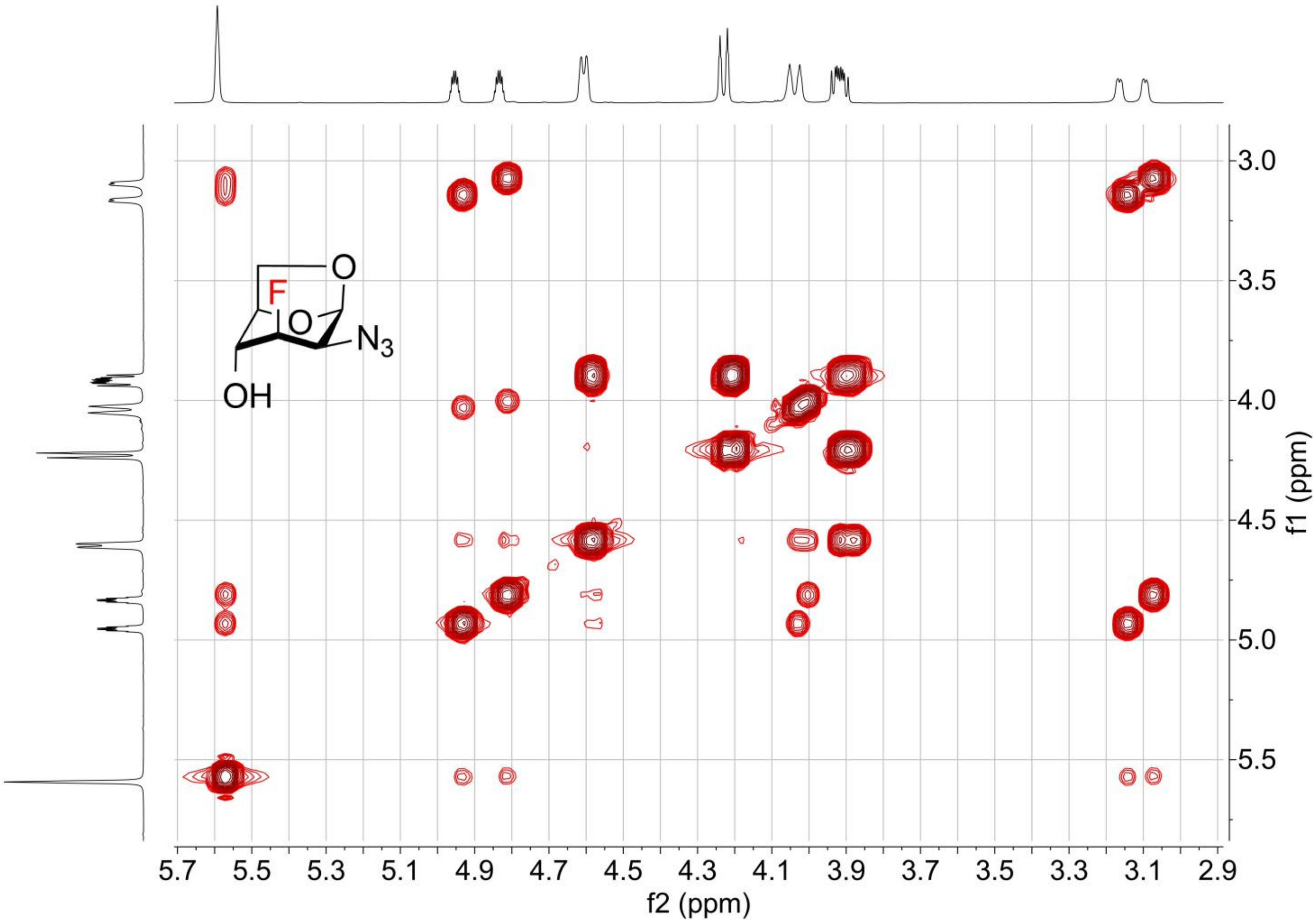

^{1}H-^{13}C HSQC (^{1}H/^{13}C 400/101 MHz, $CDCl_3$) 22

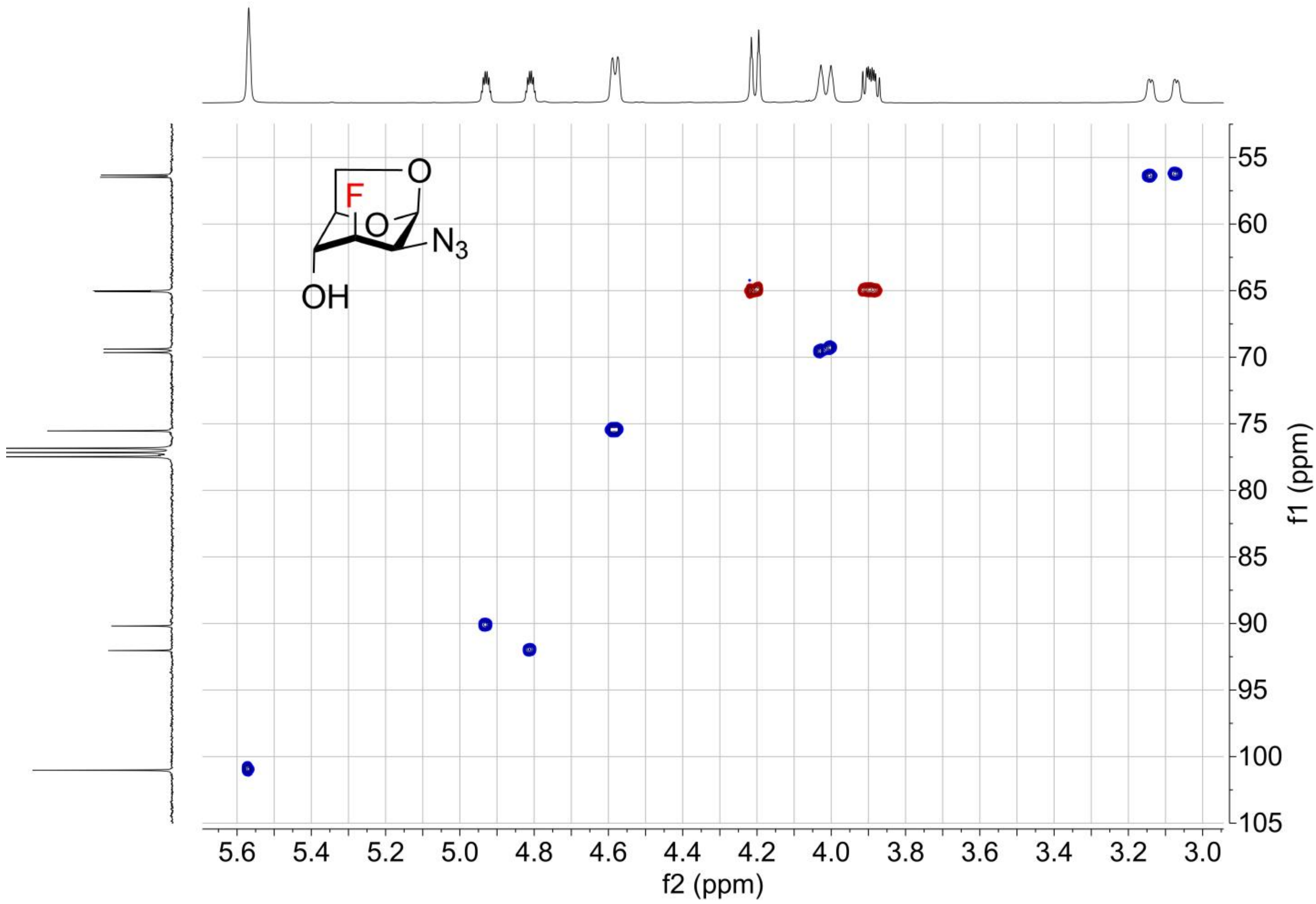

^{1}H-^{13}C HMBC (^{1}H/^{13}C 400/101 MHz, $CDCl_3$) 22

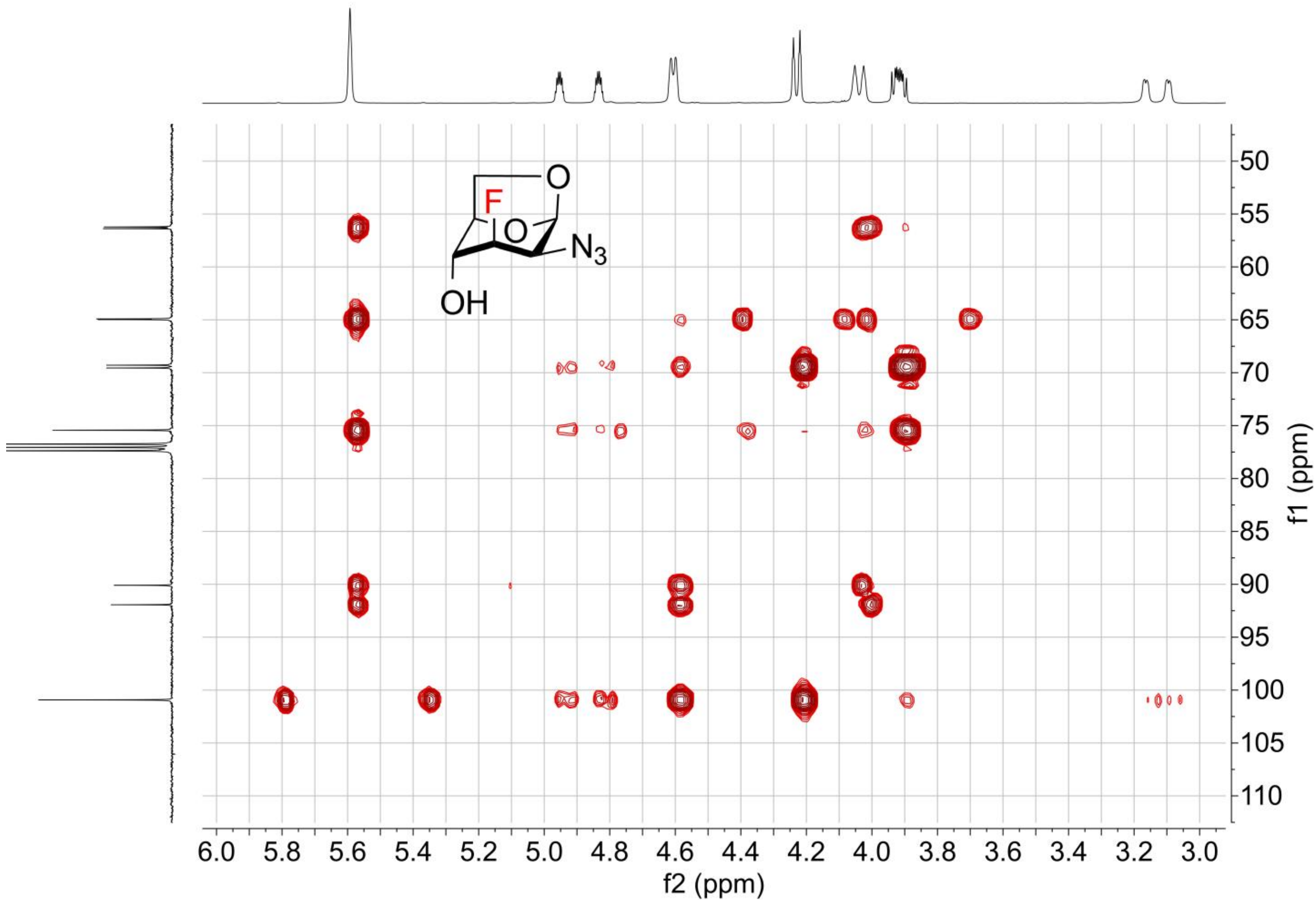

NMR COMPOUND α-23

^{1}H NMR (400 MHz, $CDCl_3$) α-23

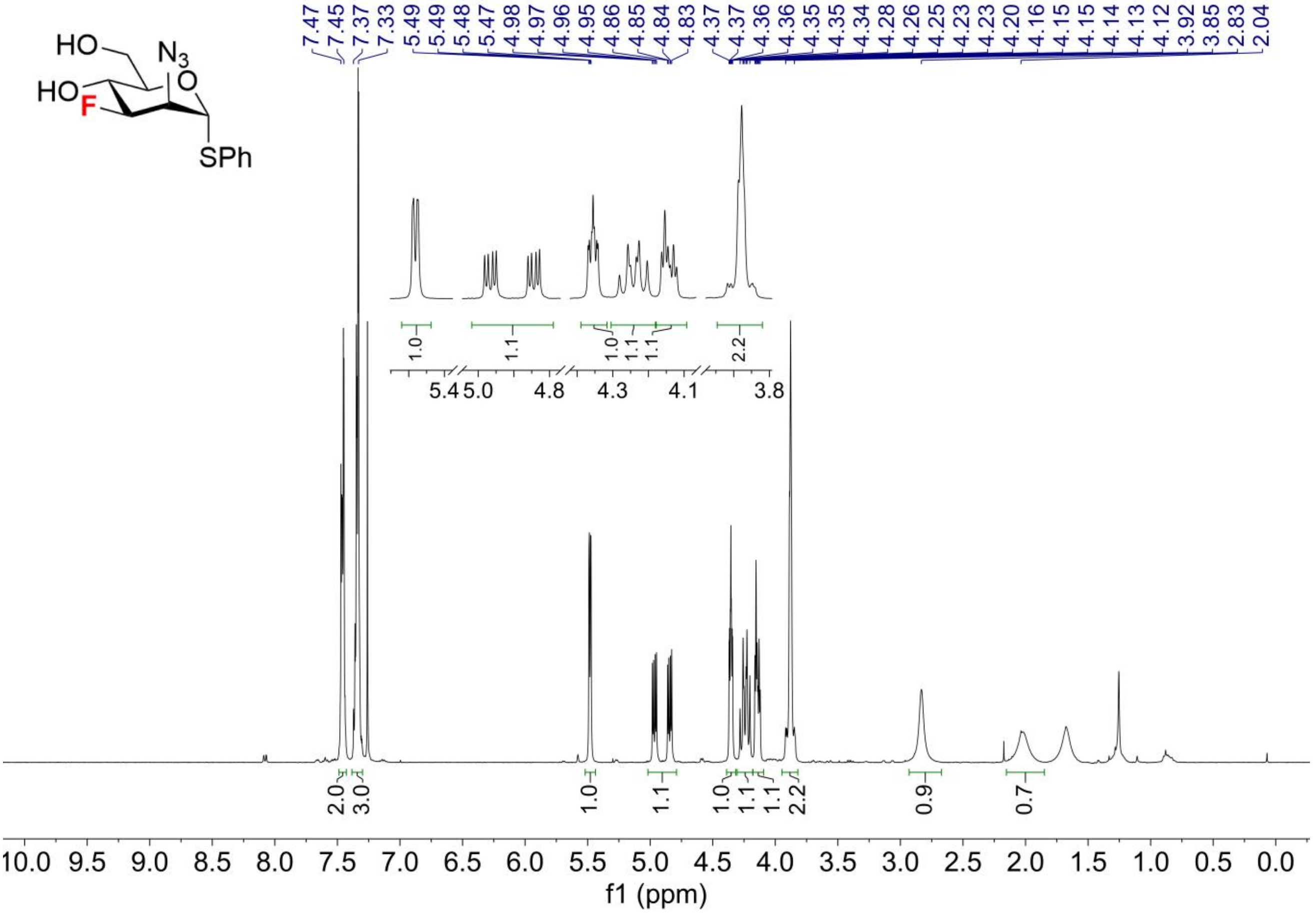

^{13}C{^{1}H} NMR (101 MHz, $CDCl_3$) α-23

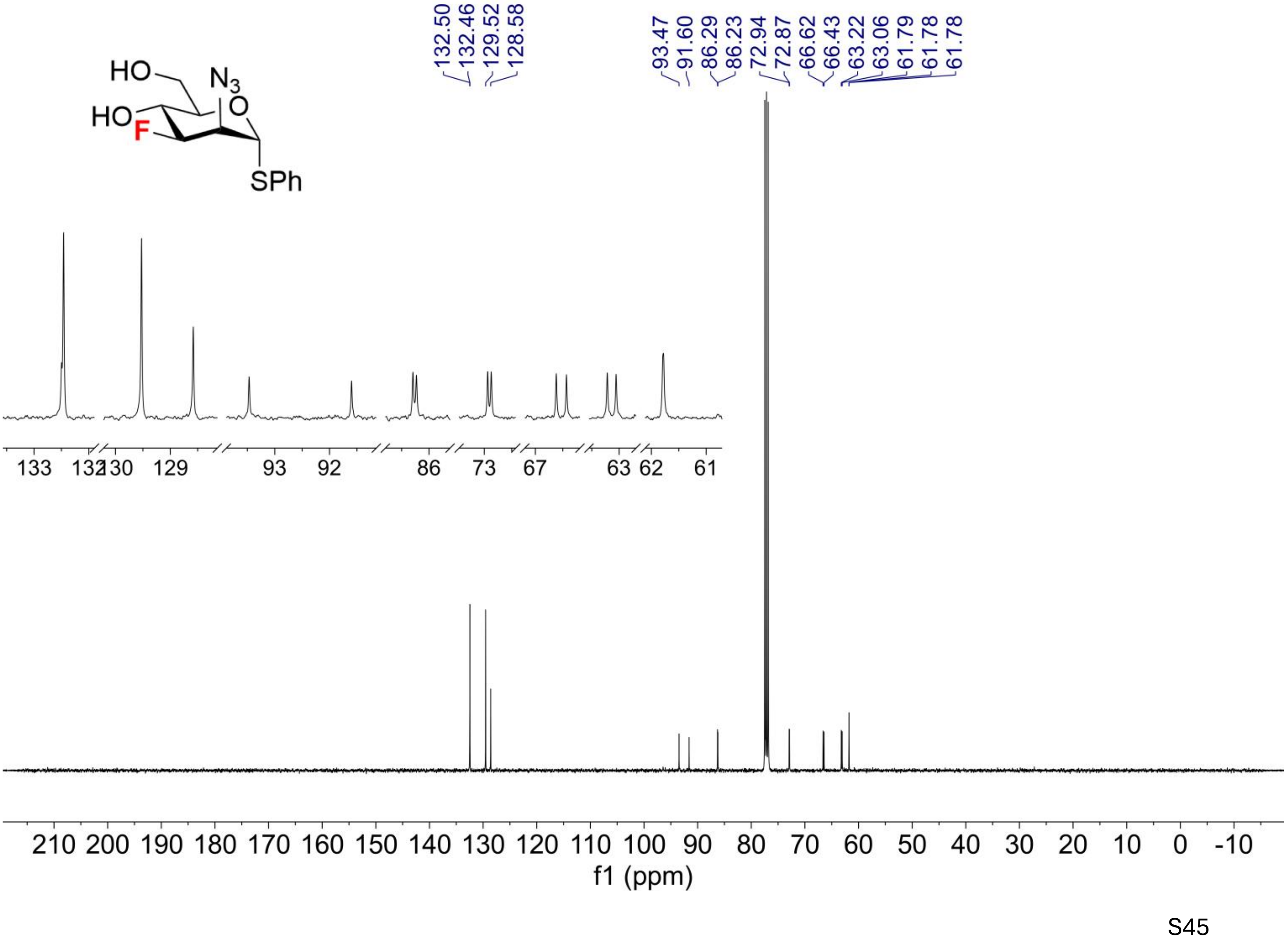

^{19}F NMR (376 MHz, CDCl$_3$) α-23

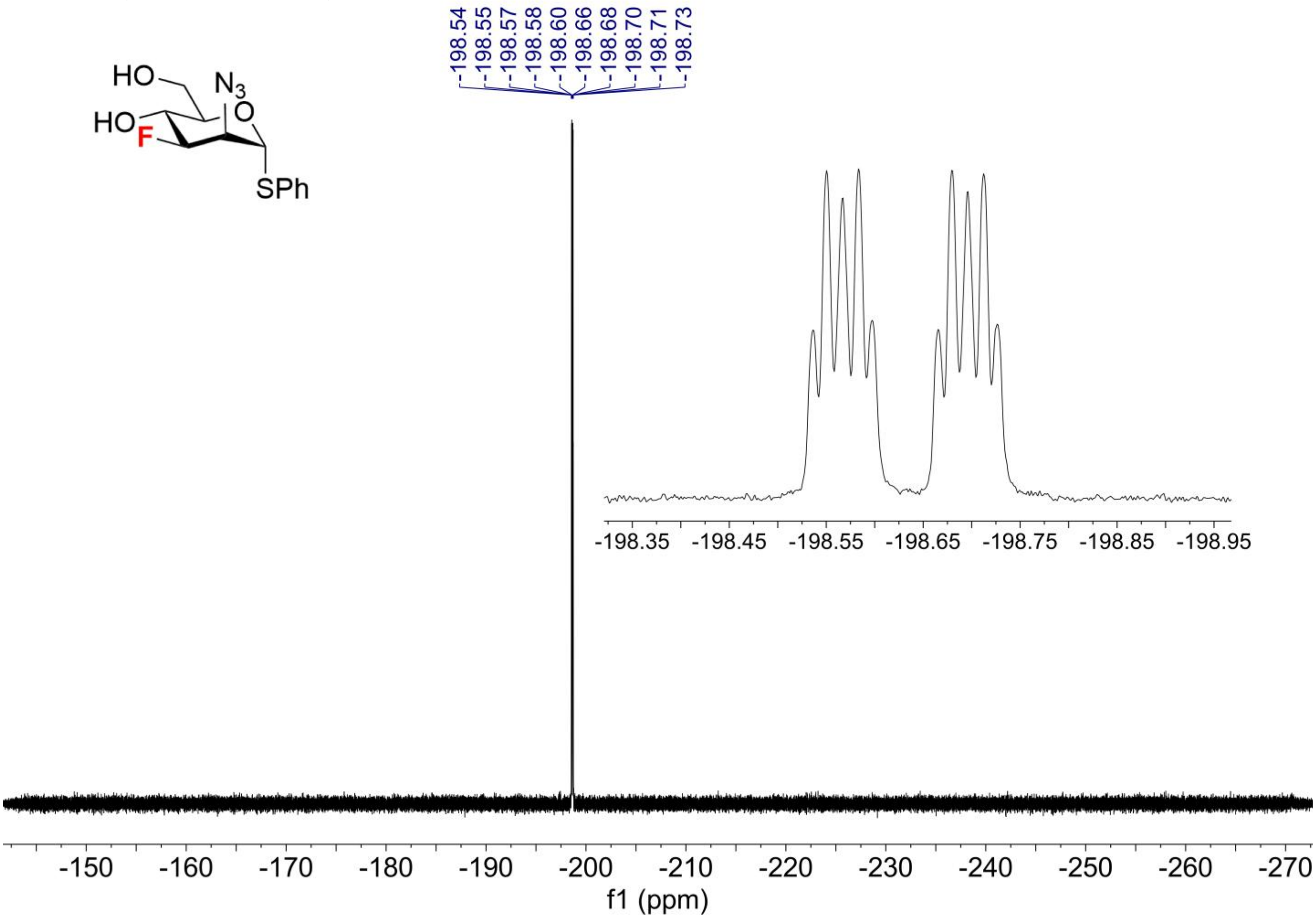

^{1}H-^{1}H COSY (400 MHz, CDCl$_3$) α-23

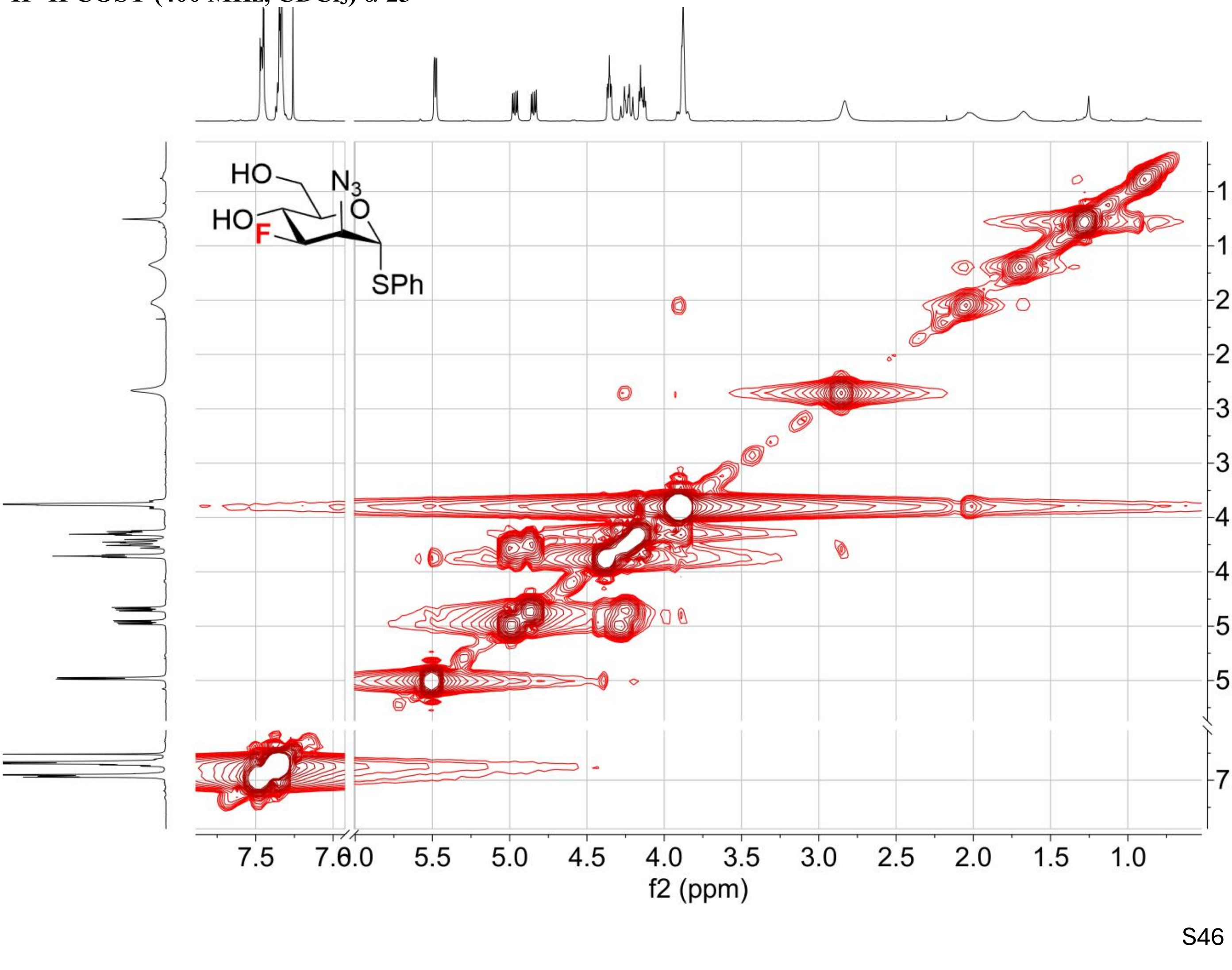

^{1}H-^{13}C HSQC (^{1}H/^{13}C 400/101 MHz, $CDCl_3$) α-23

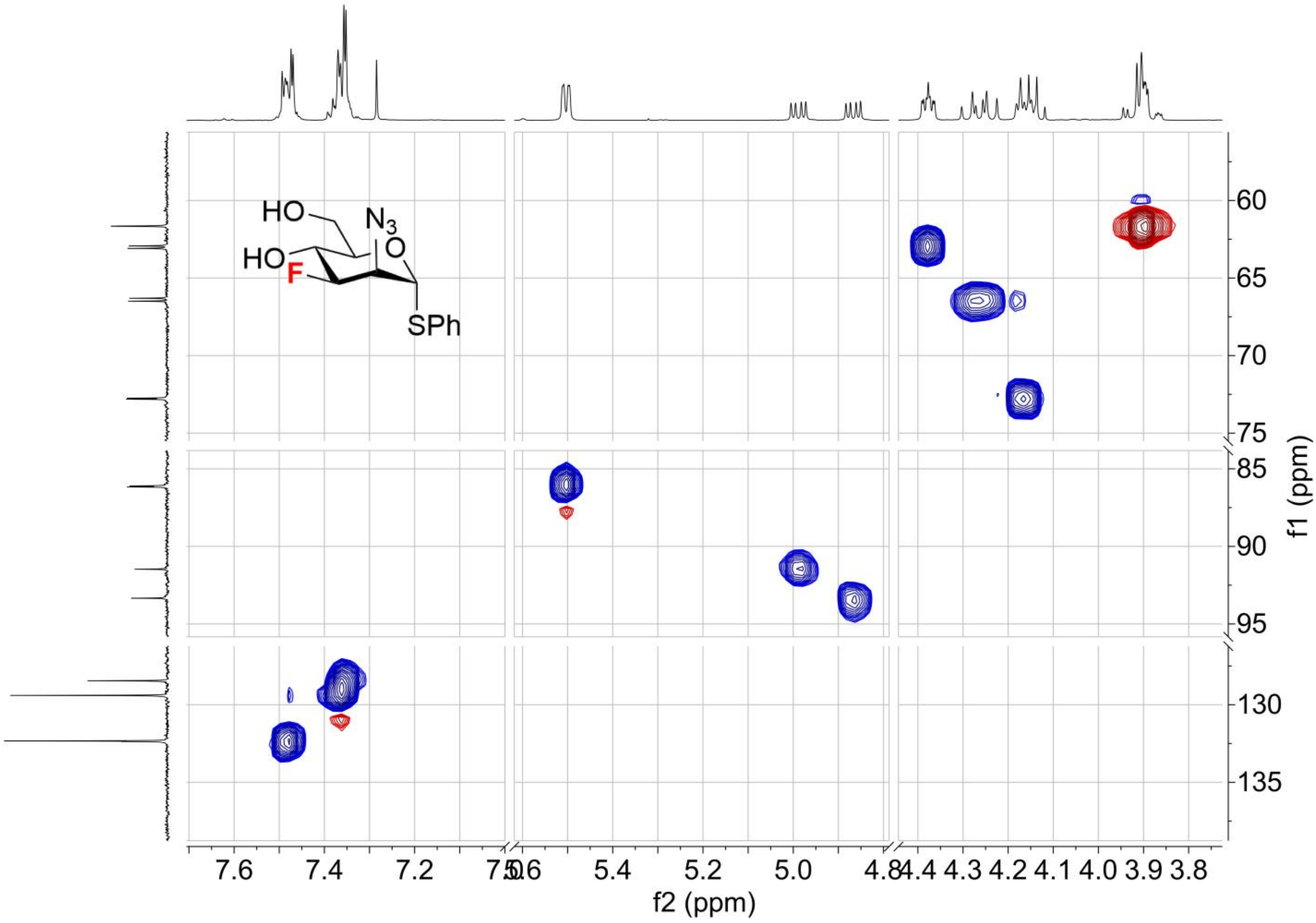

NMR COMPOUND β-23

^{1}H NMR (400 MHz, CDCl$_3$) β-23

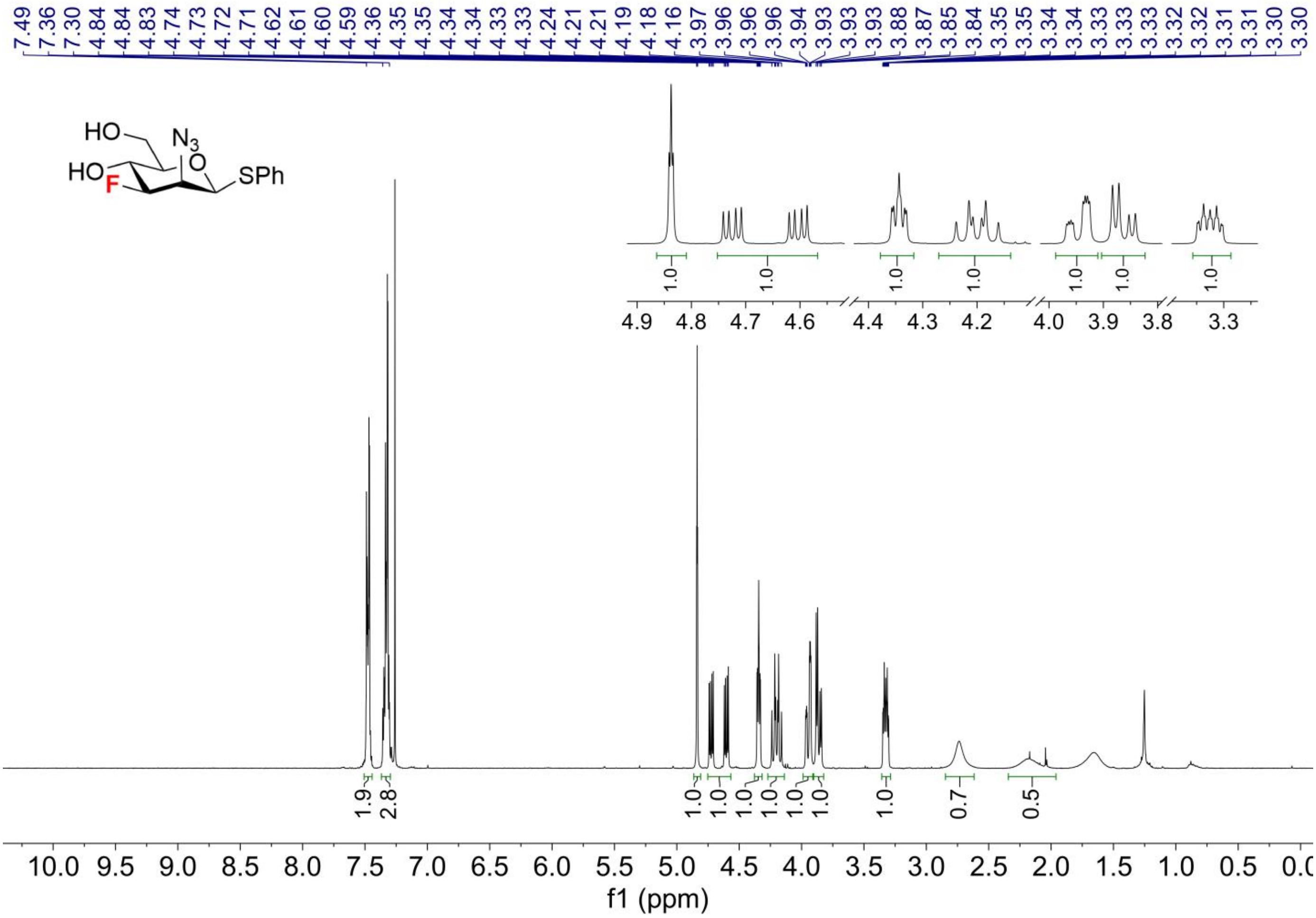

^{13}C{^{1}H} NMR (101 MHz, CDCl$_3$) β-23

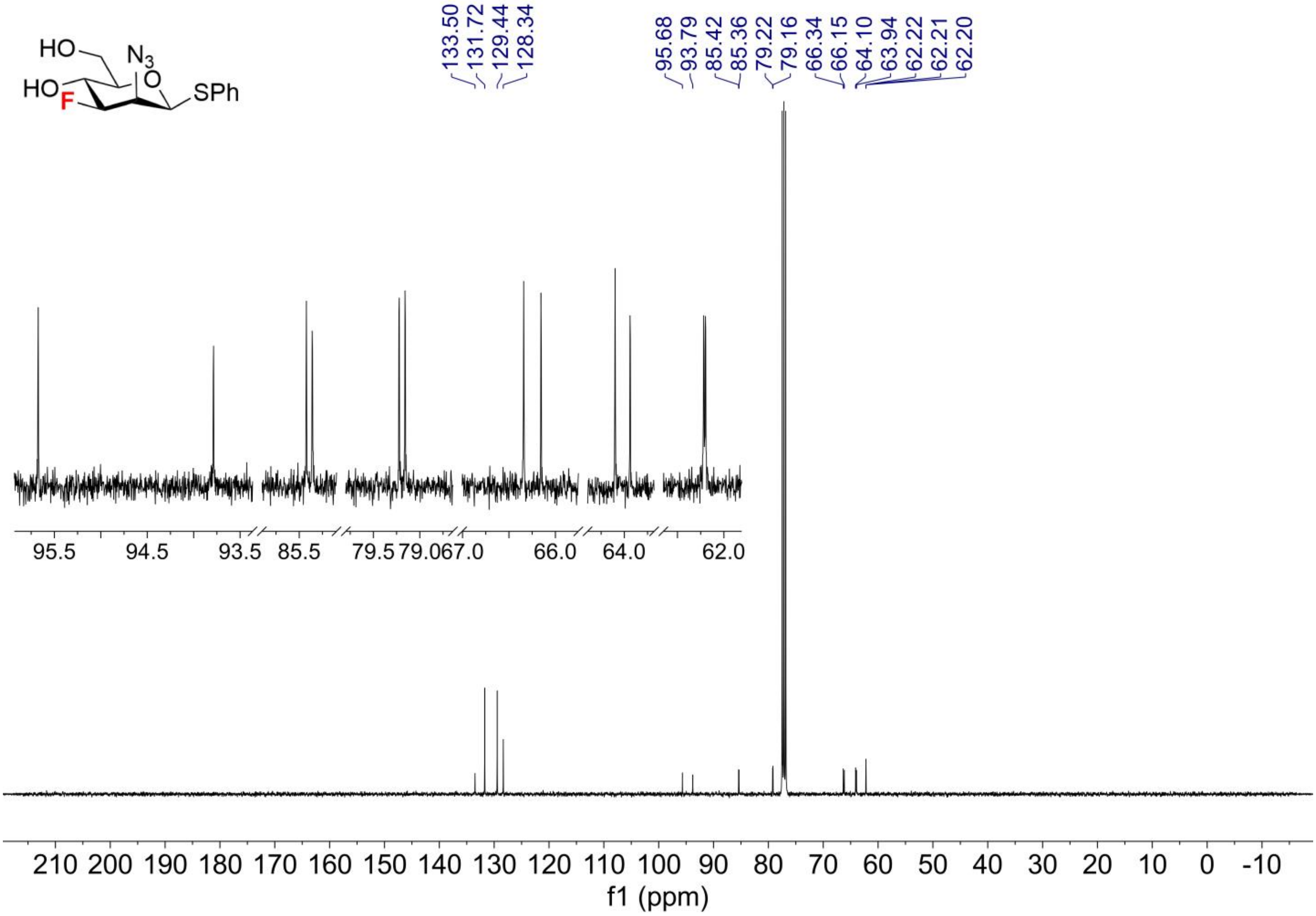

^{19}F NMR (376 MHz, $CDCl_3$) β-23

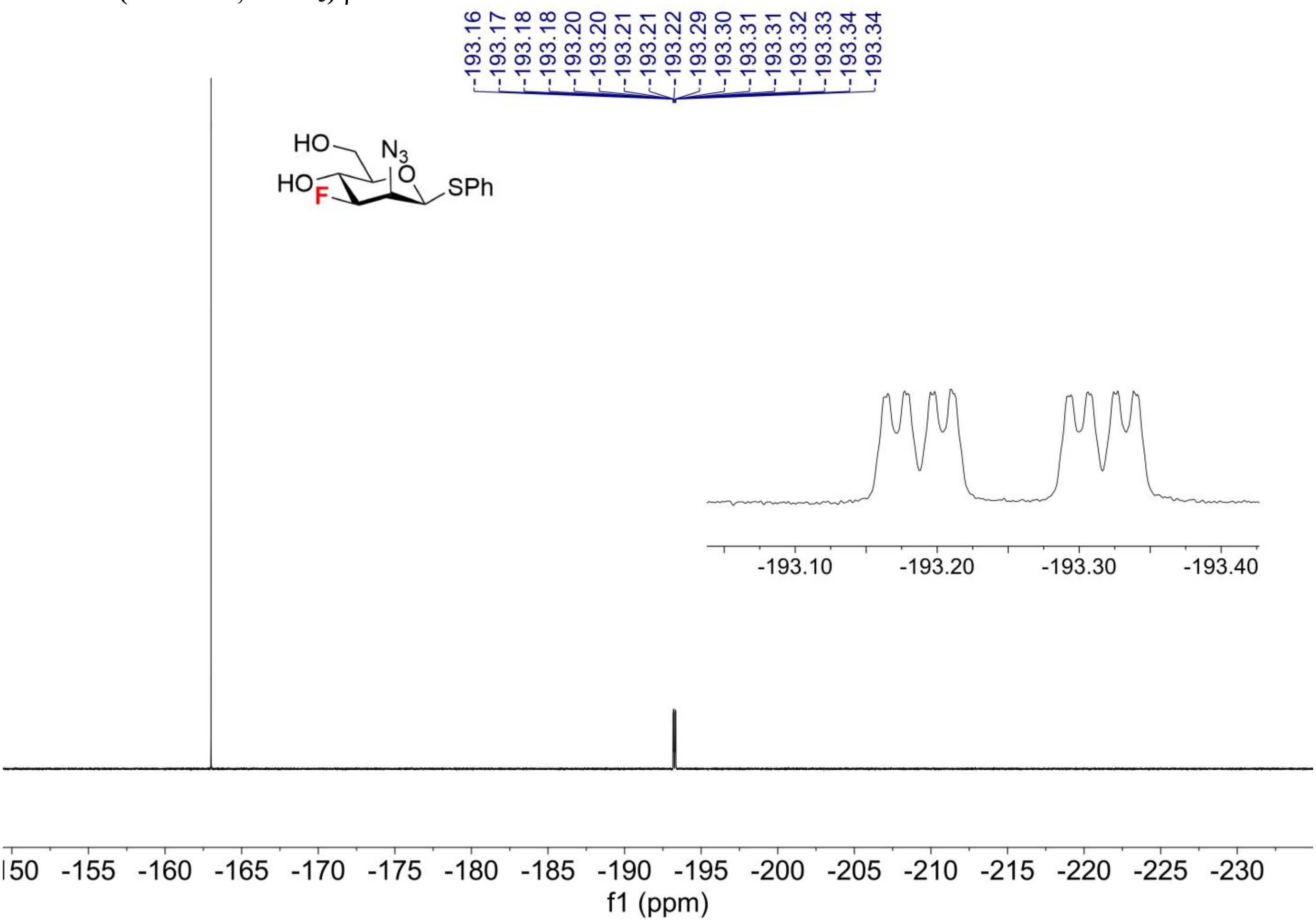

^{1}H-^{1}H COSY (400 MHz, $CDCl_3$) β-23

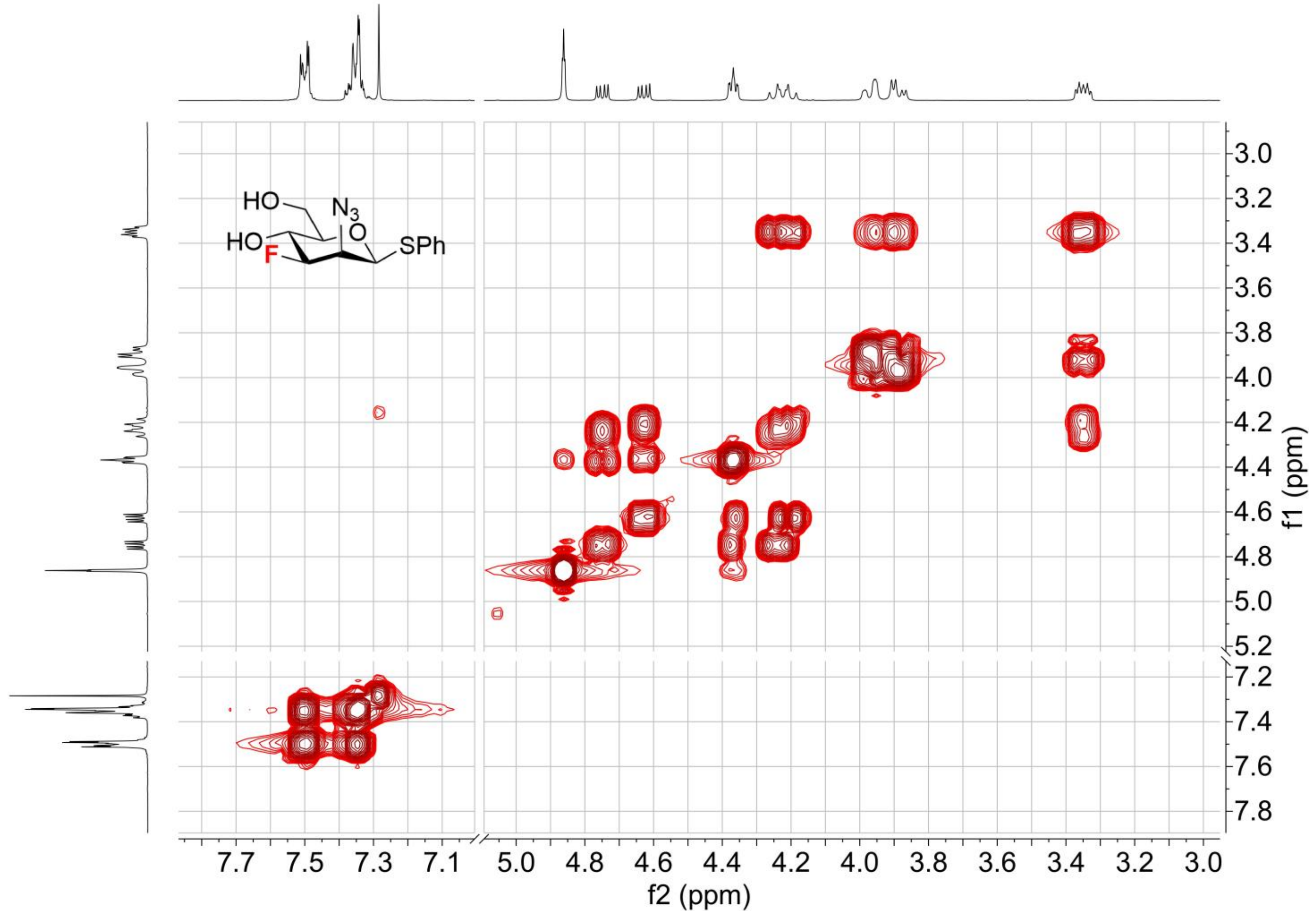

^{1}H-^{13}C HSQC (^{1}H/^{13}C 400/101 MHz, $CDCl_3$) β-23

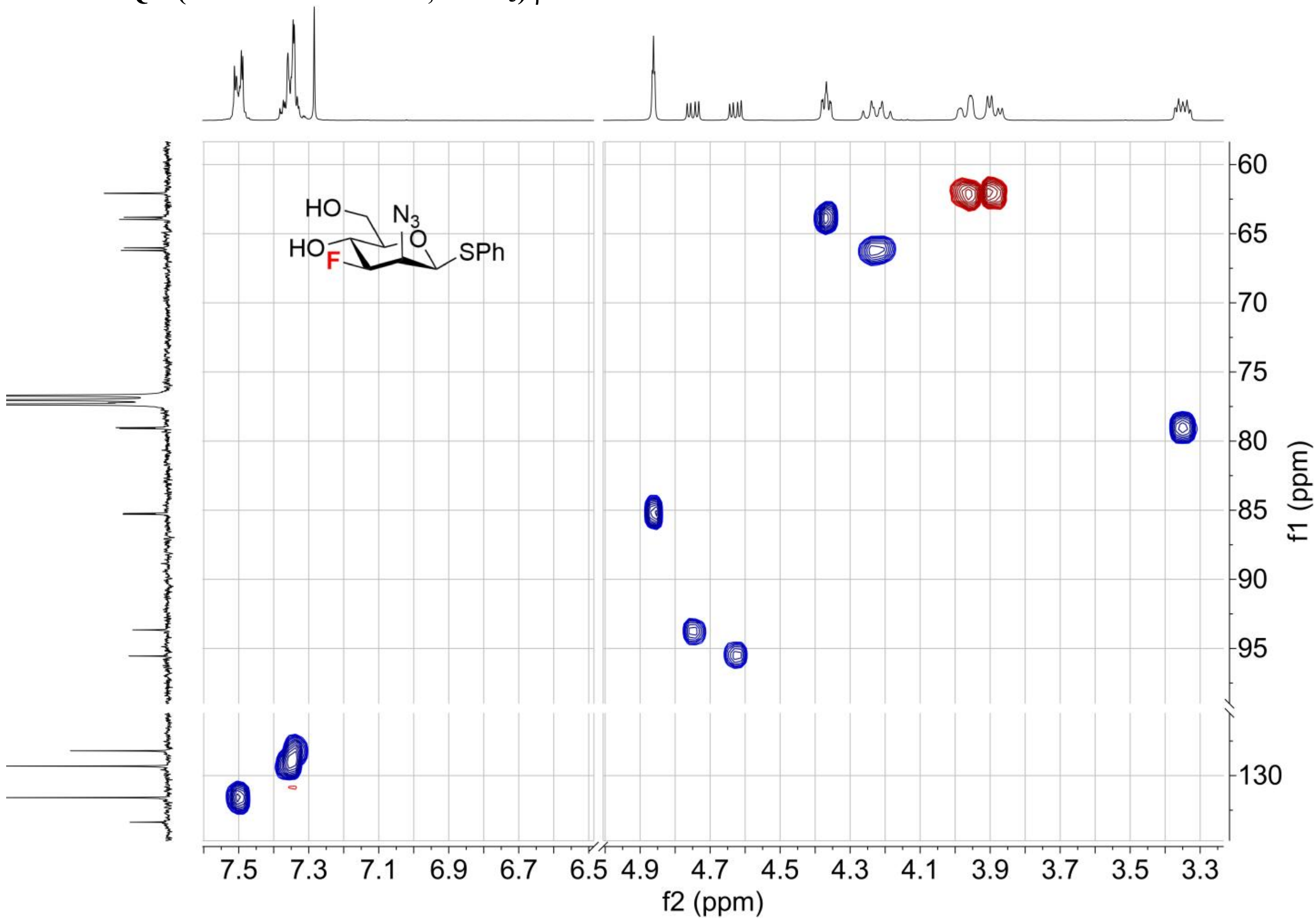

NMR COMPOUND 24

^{1}H NMR (400 MHz, CDCl$_3$) 24

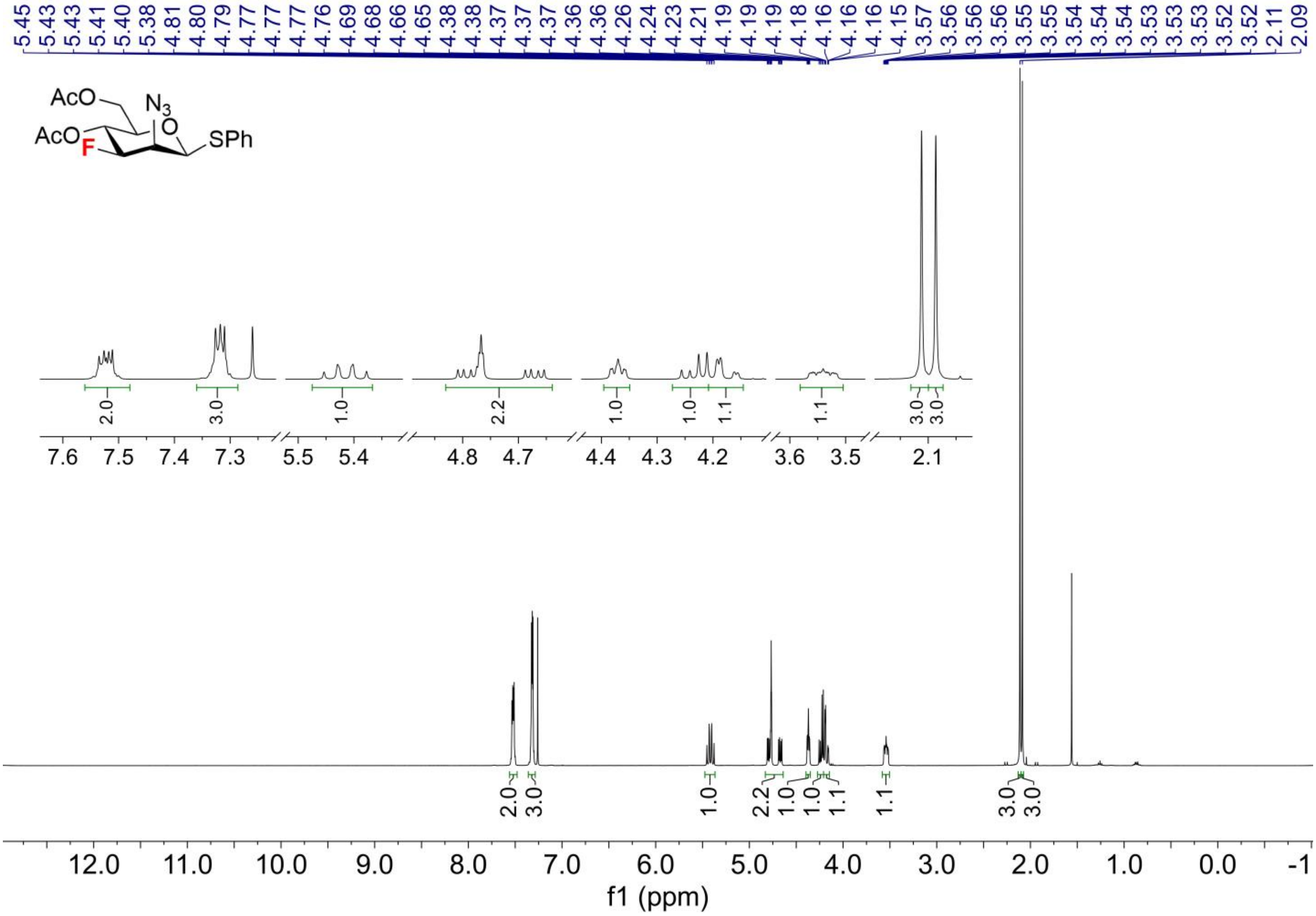

^{13}C{^{1}H} NMR (101 MHz, CDCl$_3$) 24

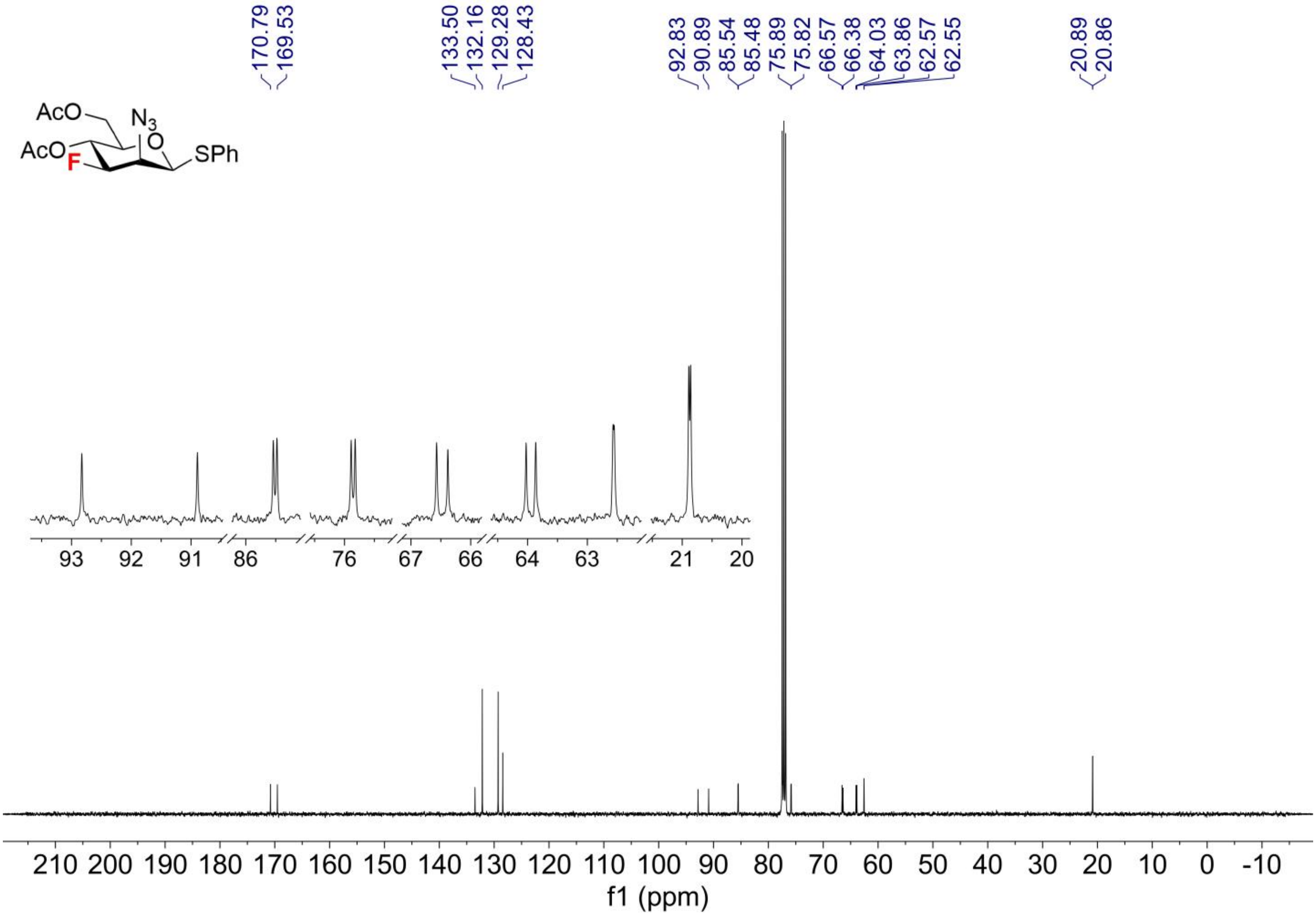

^{19}F NMR (376 MHz, CDCl$_3$) 24

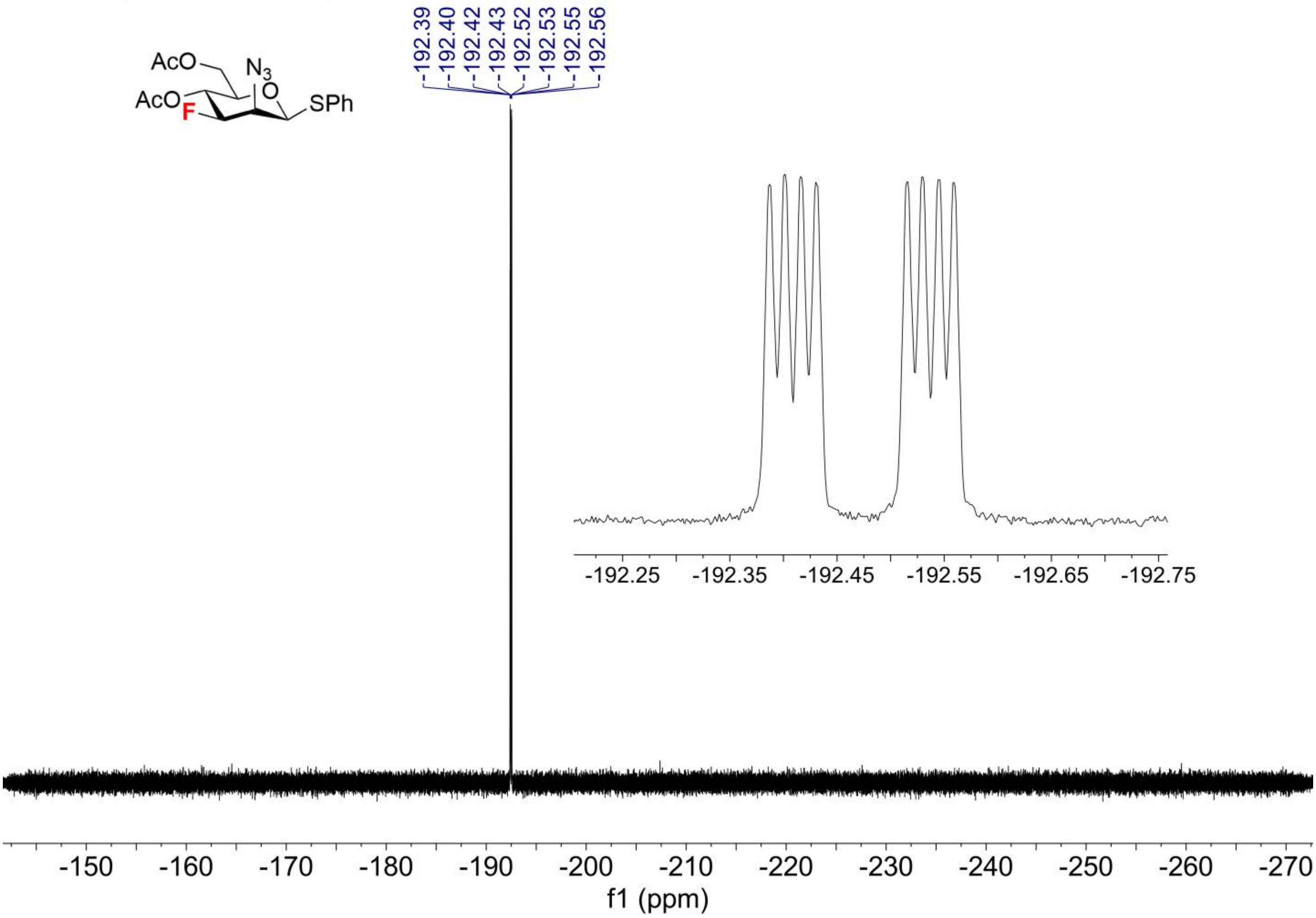

^{1}H-^{1}H COSY (400 MHz, CDCl$_3$) 24

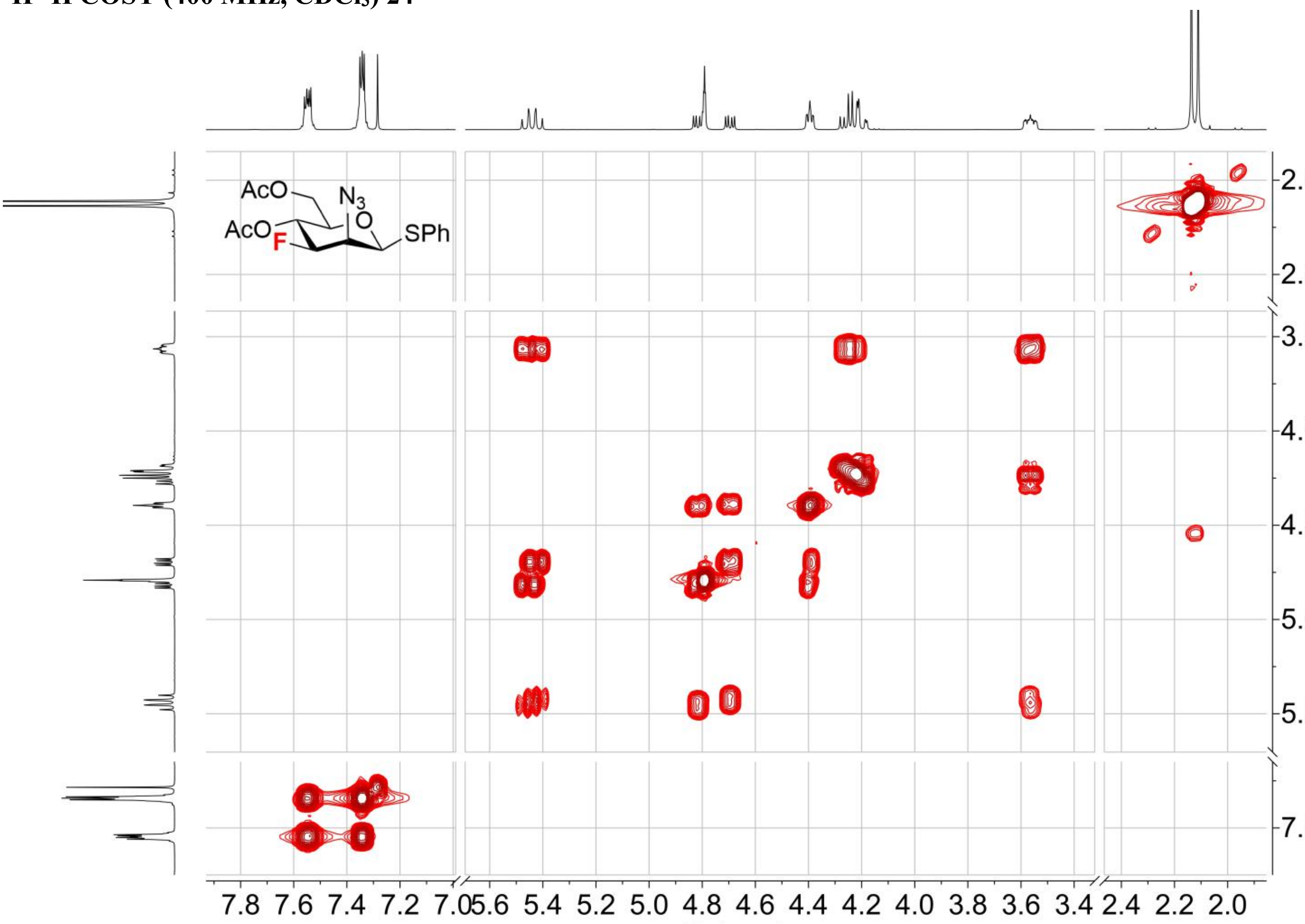

^{1}H-^{13}C HSQC (^{1}H/^{13}C 400/101 MHz, $CDCl_3$) 24

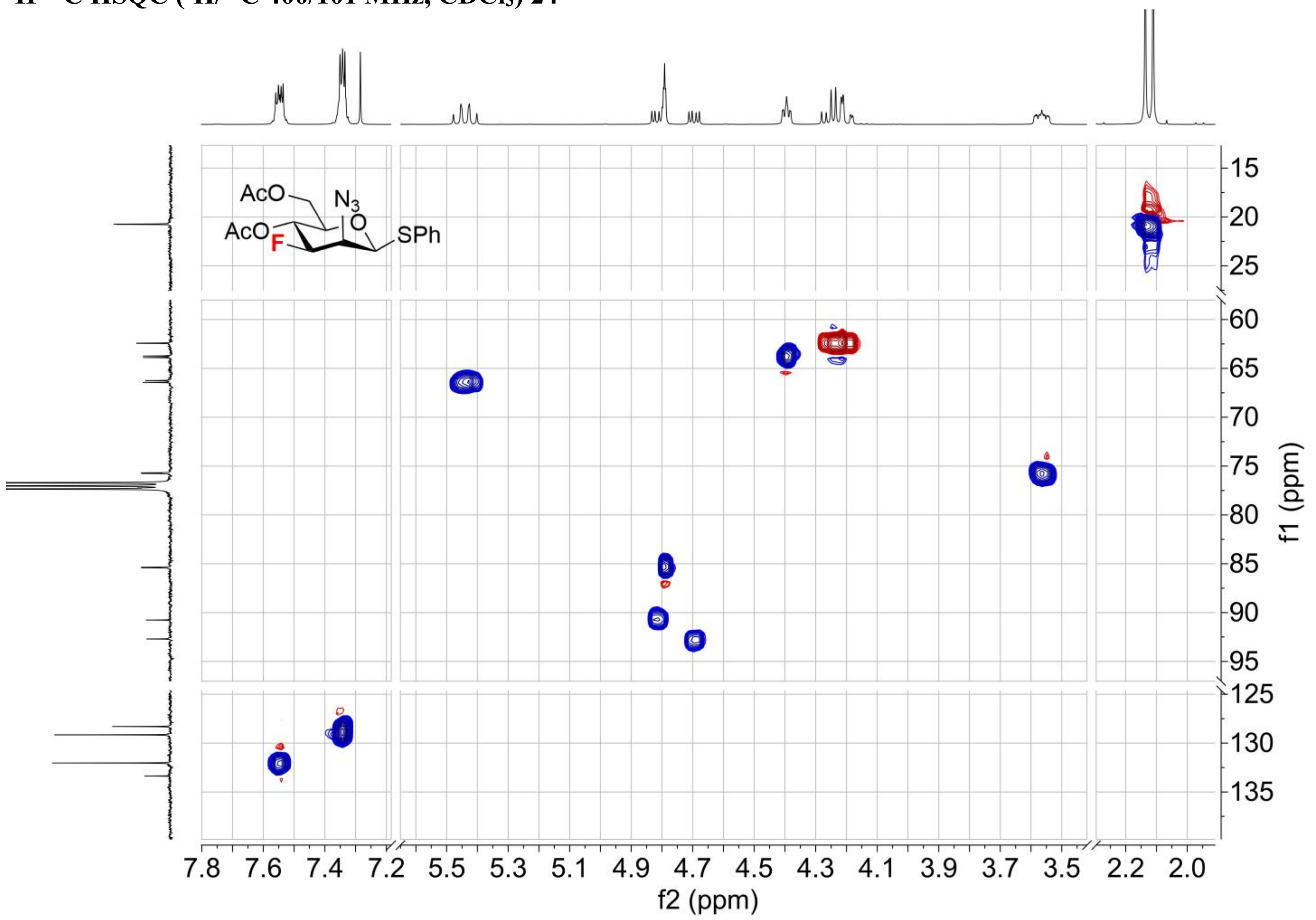

^{1}H-^{13}C HMBC (^{1}H/^{13}C 400/101 MHz, $CDCl_3$) 24

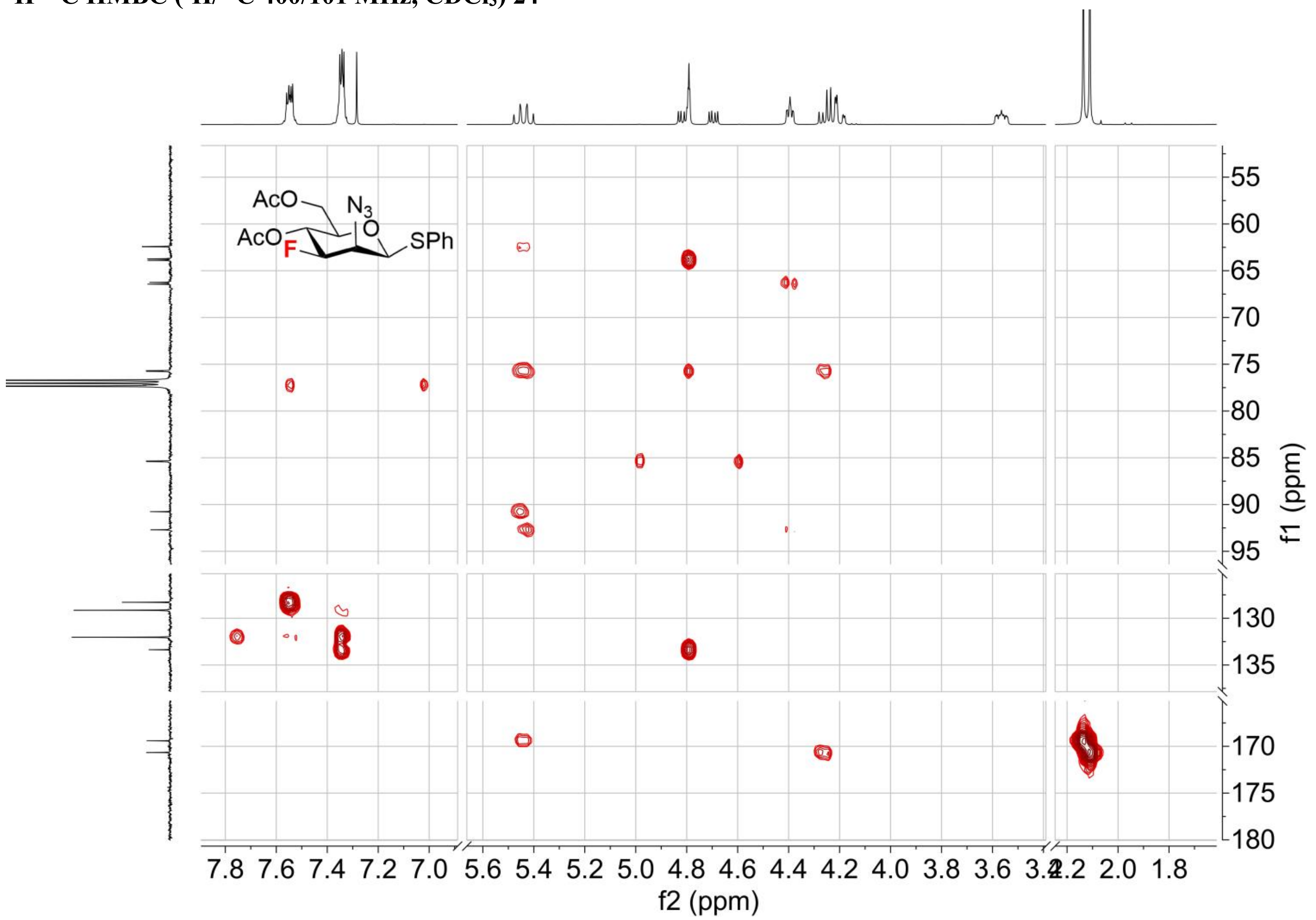

NMR COMPOUND 25

^{1}H NMR (400 MHz, $CDCl_3$) 25

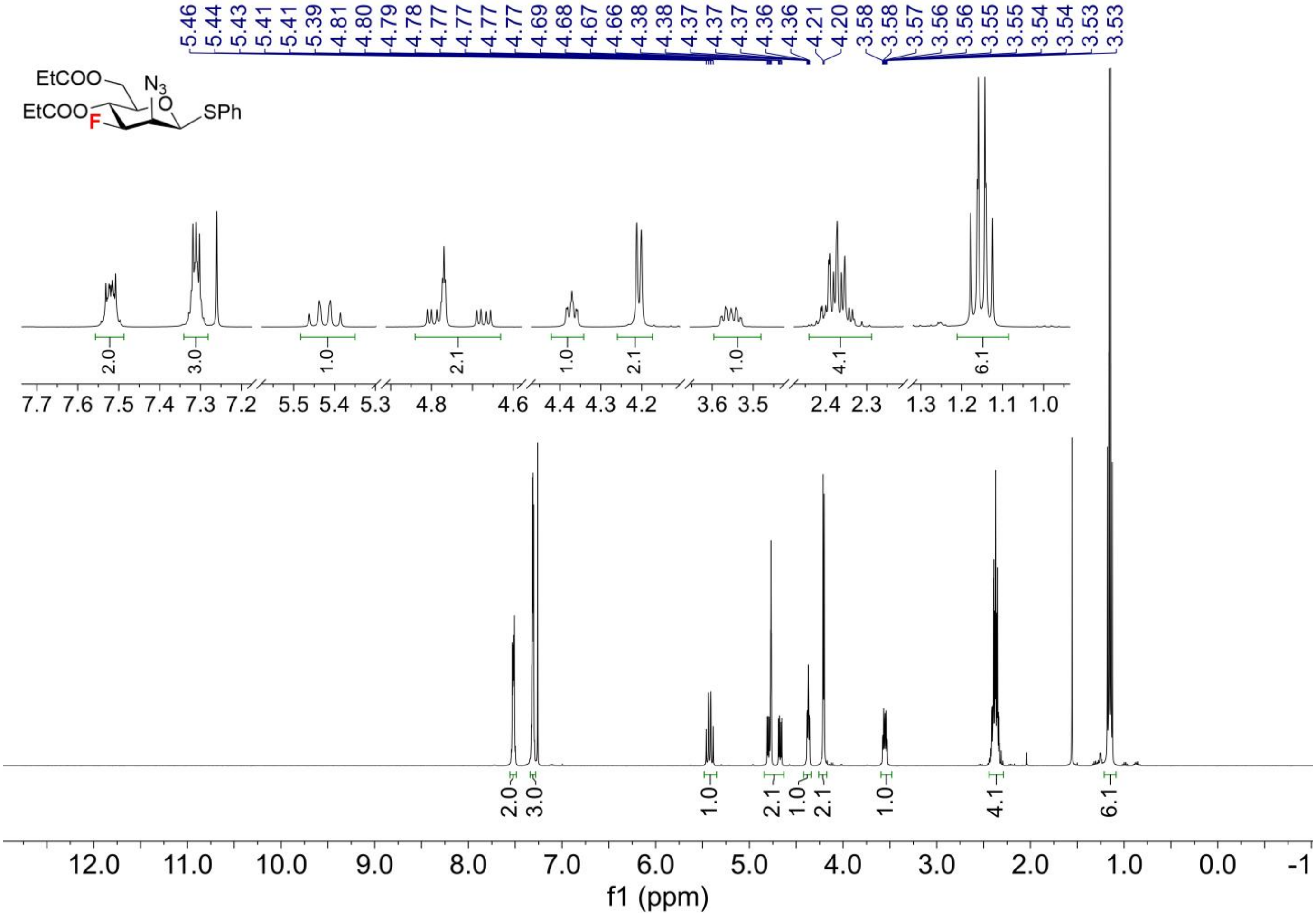

^{13}C{^{1}H} NMR (101 MHz, $CDCl_3$) 25

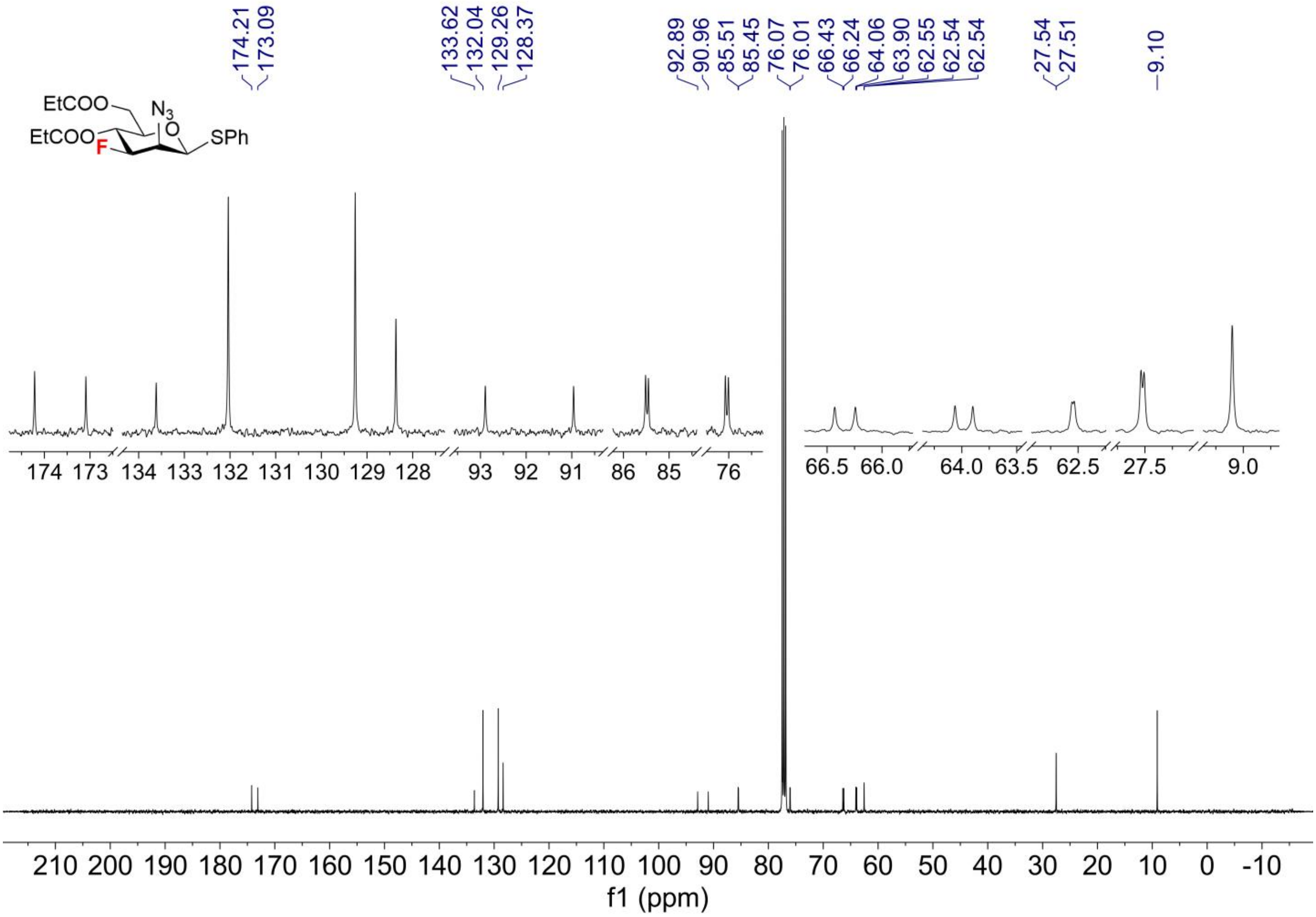

^{19}F NMR (376 MHz, CDCl$_3$) 25

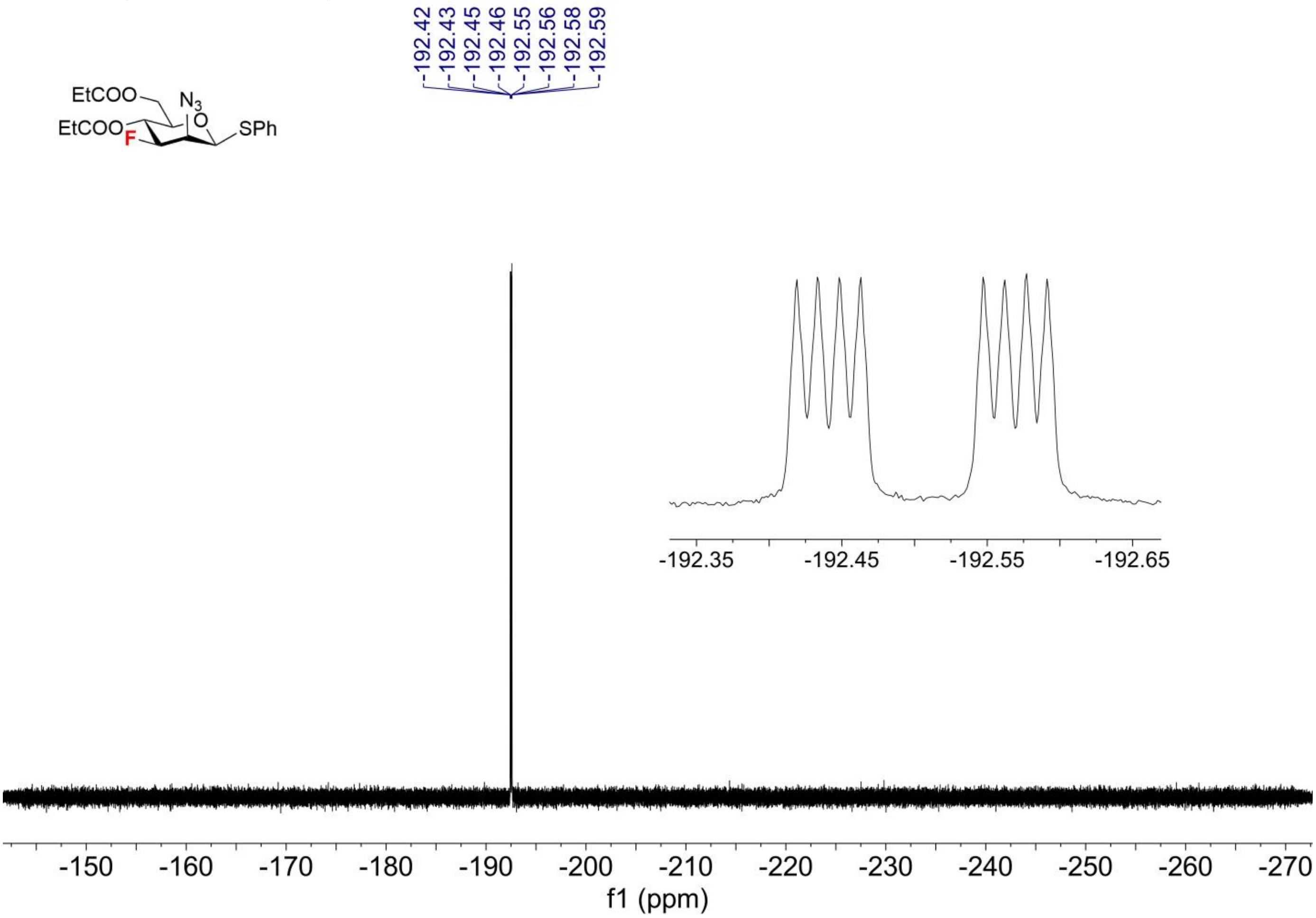

^{1}H-^{1}H COSY (400 MHz, CDCl$_3$) 25

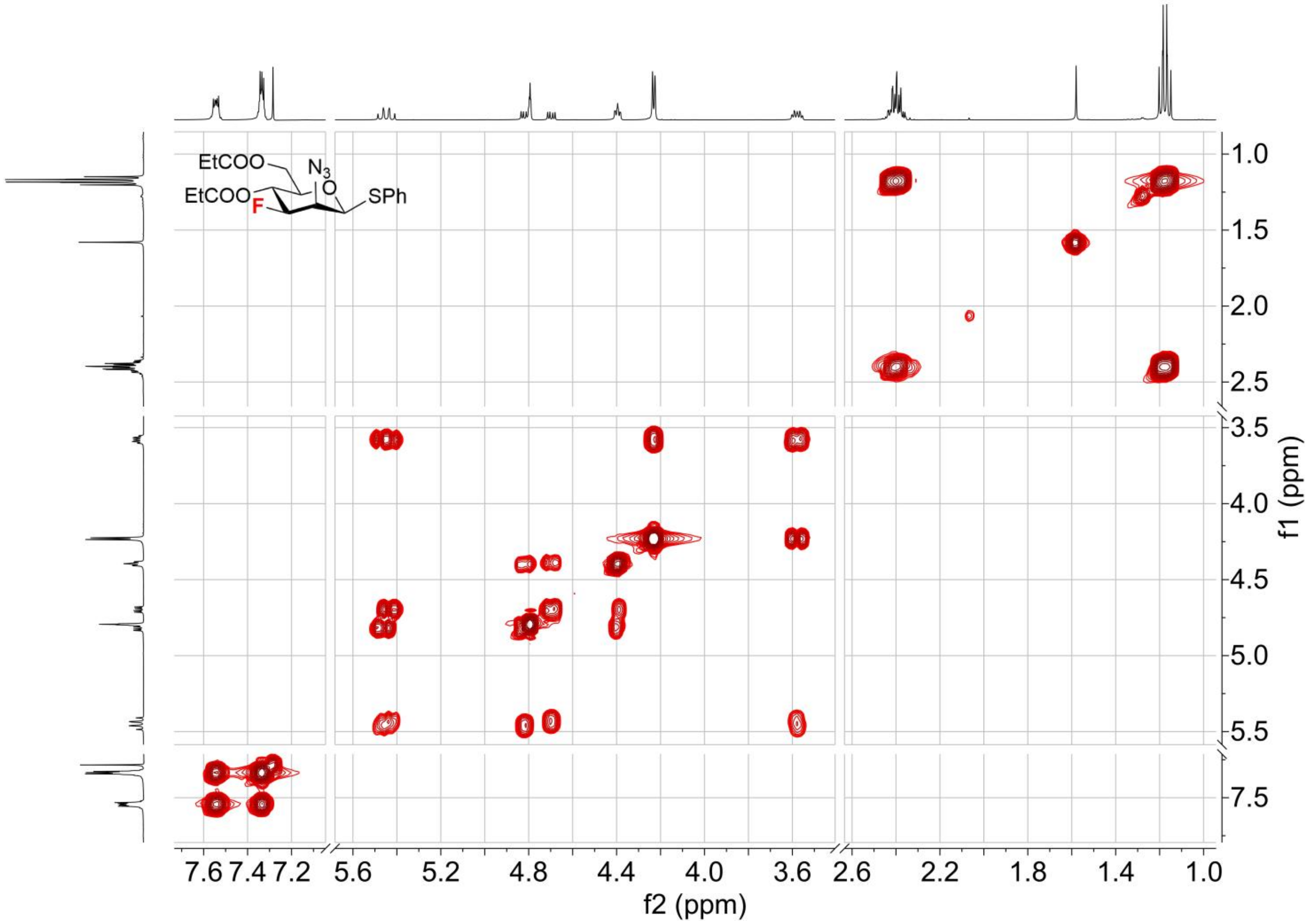

^{1}H-^{13}C HSQC (^{1}H/^{13}C 400/101 MHz, $CDCl_3$) 25

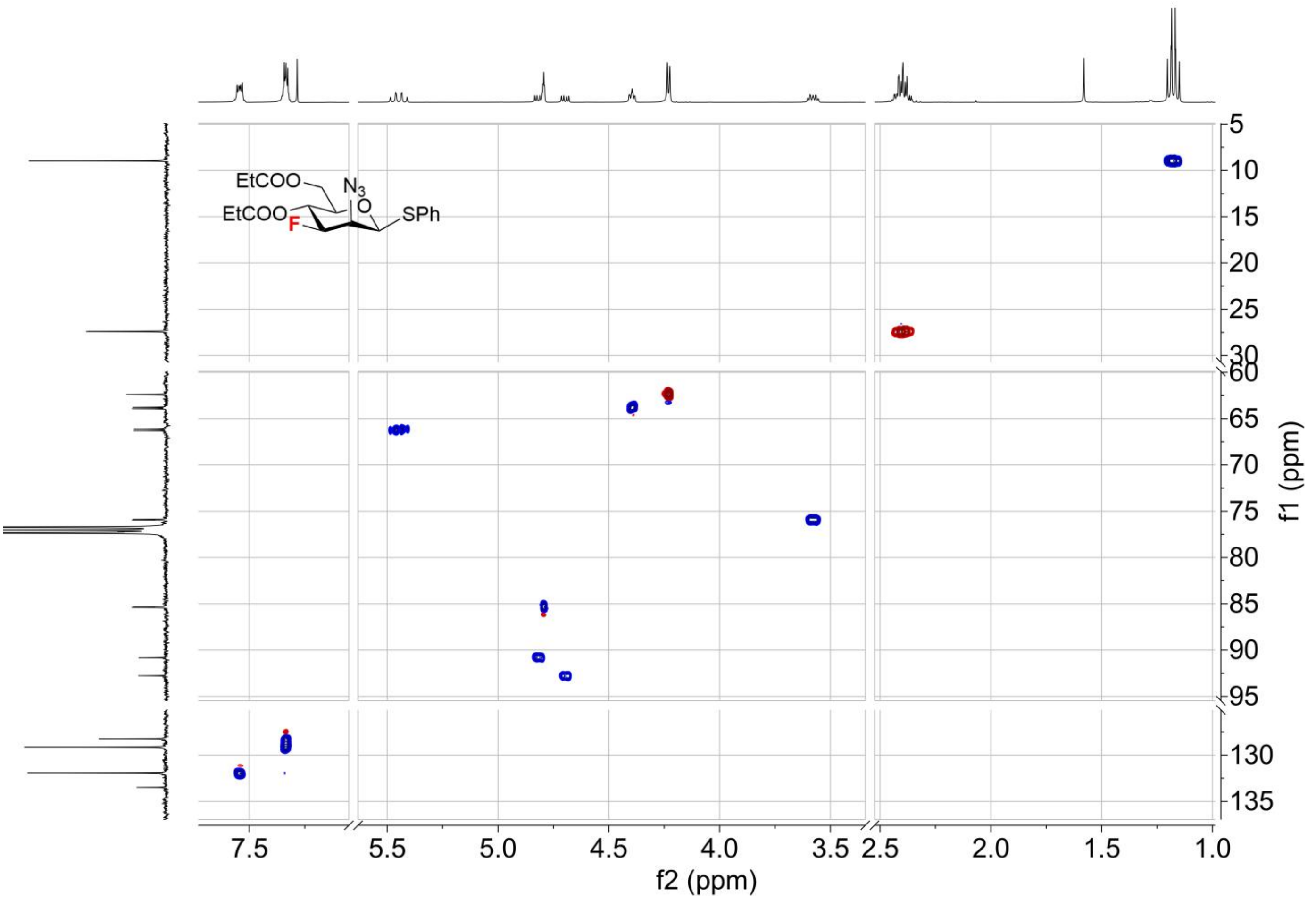

^{1}H-^{13}C HMBC (^{1}H/^{13}C 400/101 MHz, $CDCl_3$) 25

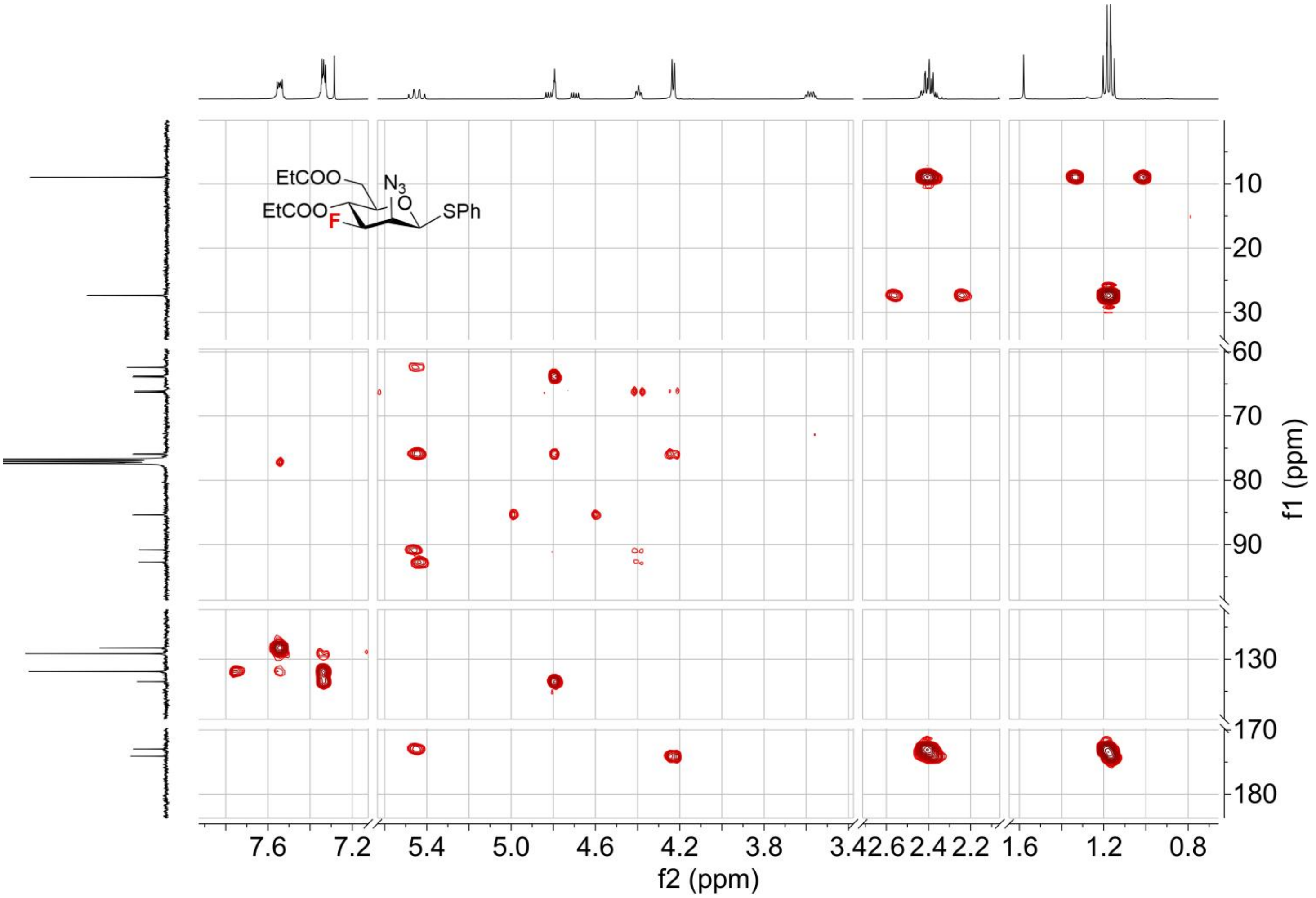

NMR COMPOUND 26

^{1}H NMR (400 MHz, $CDCl_3$) 26

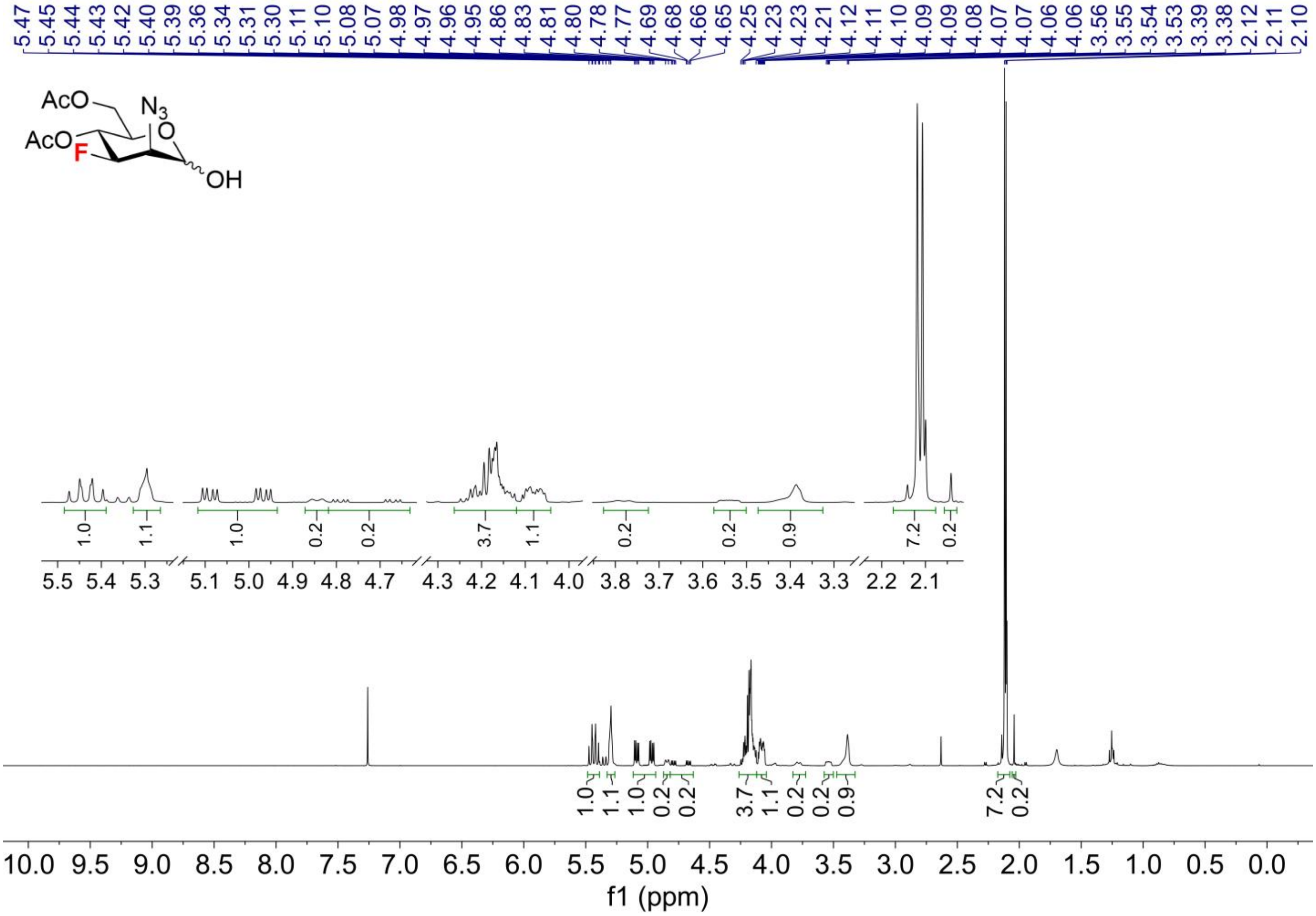

^{13}C{^{1}H} NMR (101 MHz, $CDCl_3$) 26

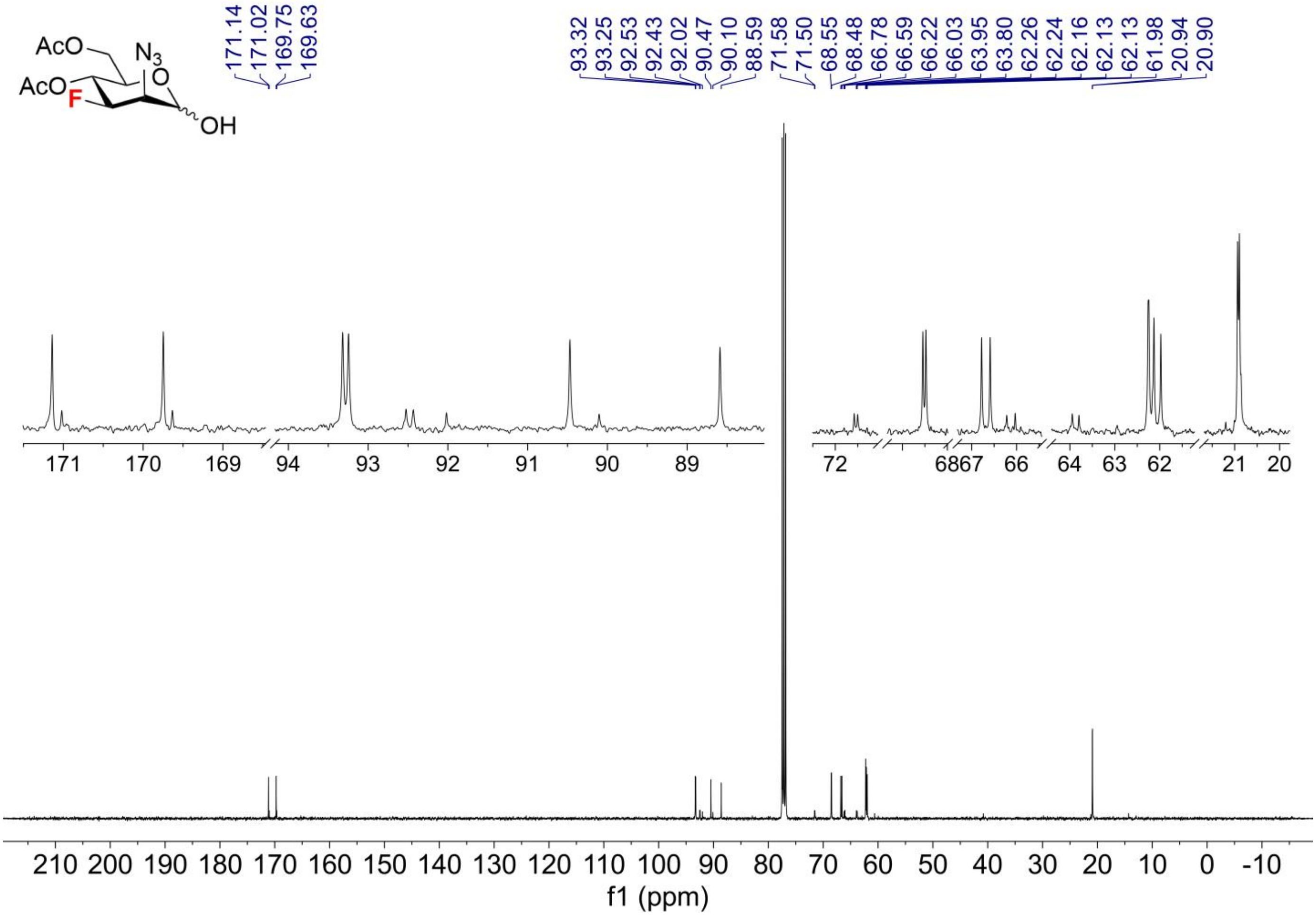

^{19}F NMR (376 MHz, CDCl$_3$) 26

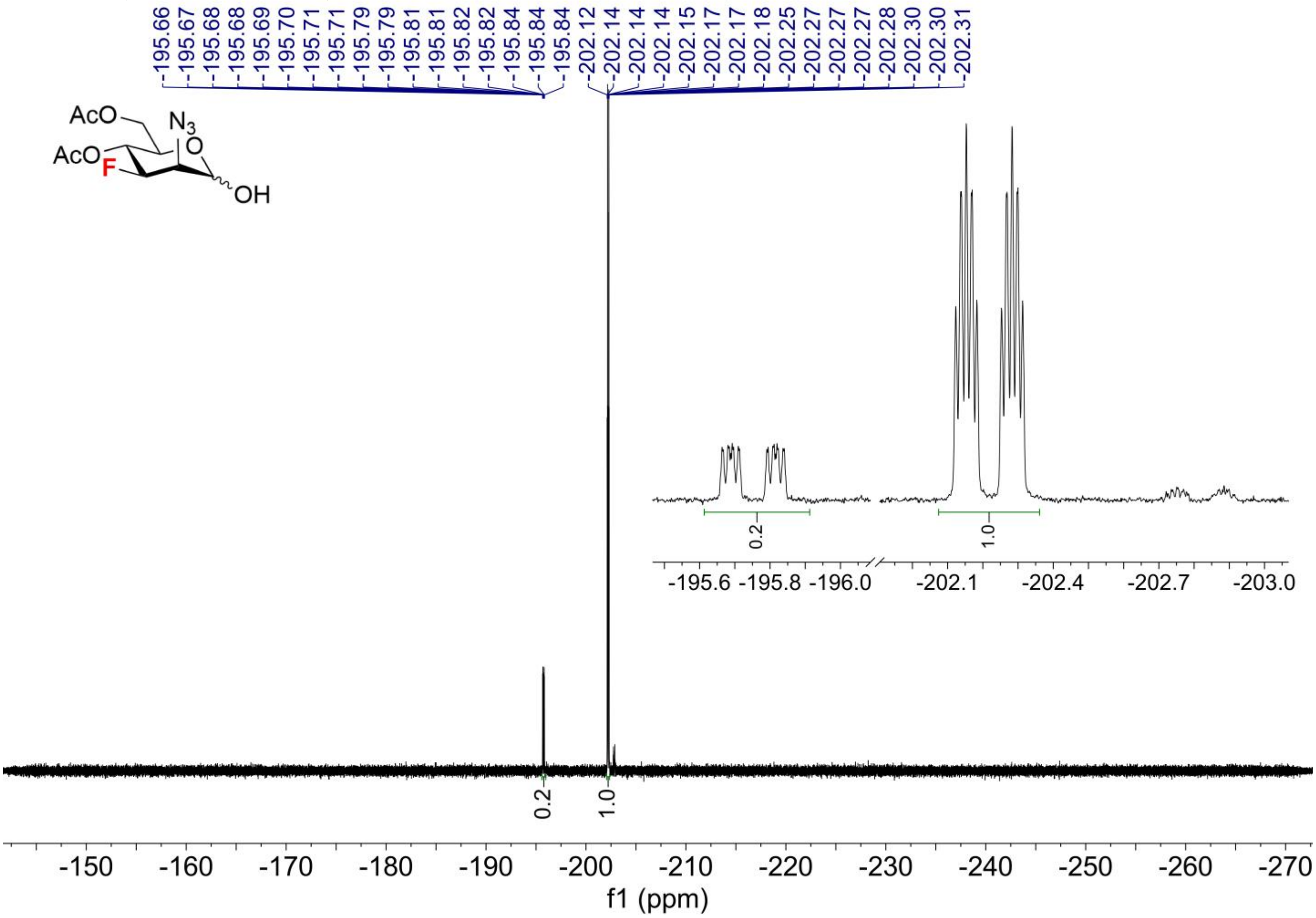

^{1}H-^{1}H COSY (400 MHz, CDCl$_3$) 26

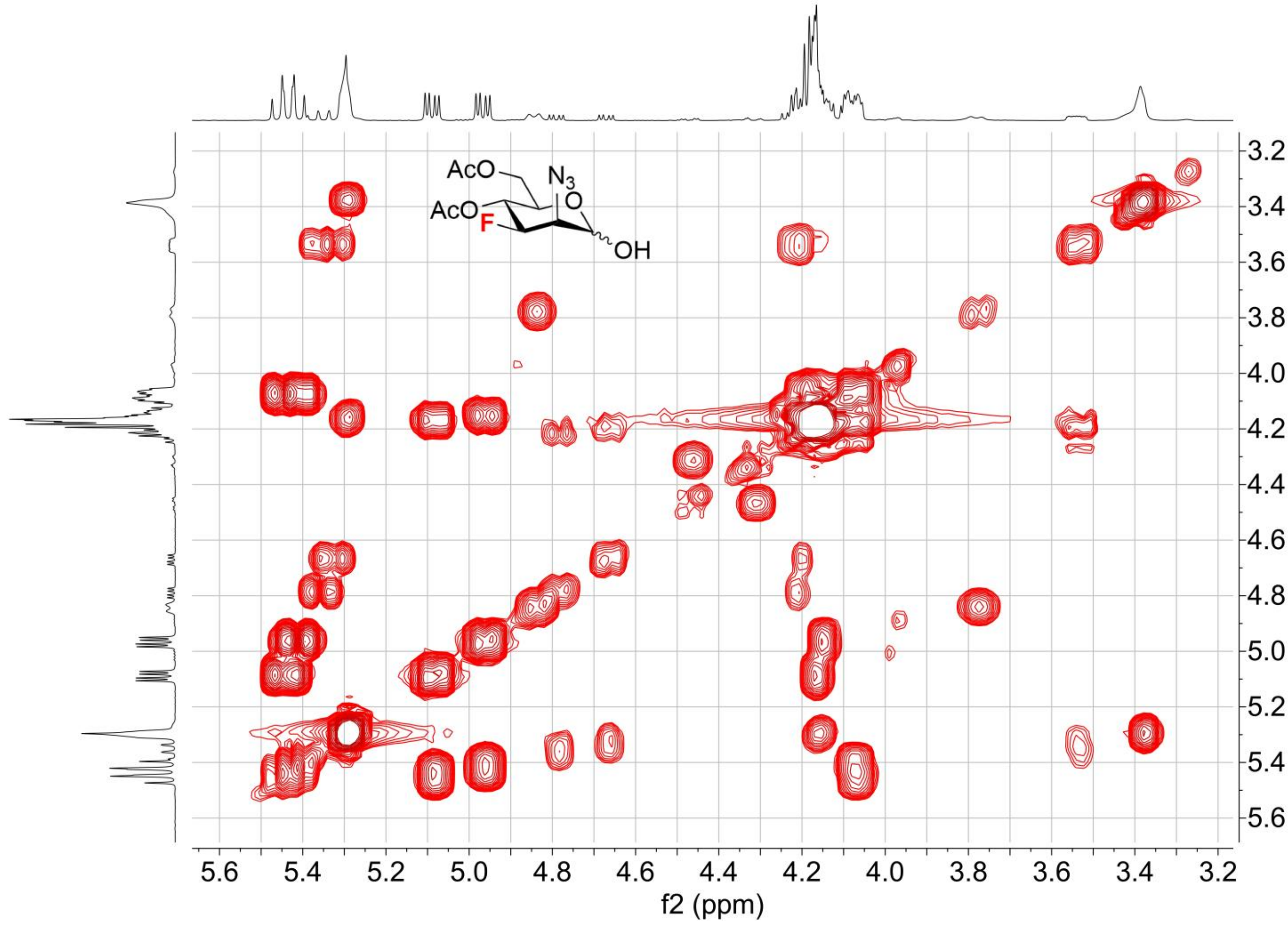

^{1}H-^{13}C HSQC (^{1}H/^{13}C 400/101 MHz, CDCl$_3$) 26

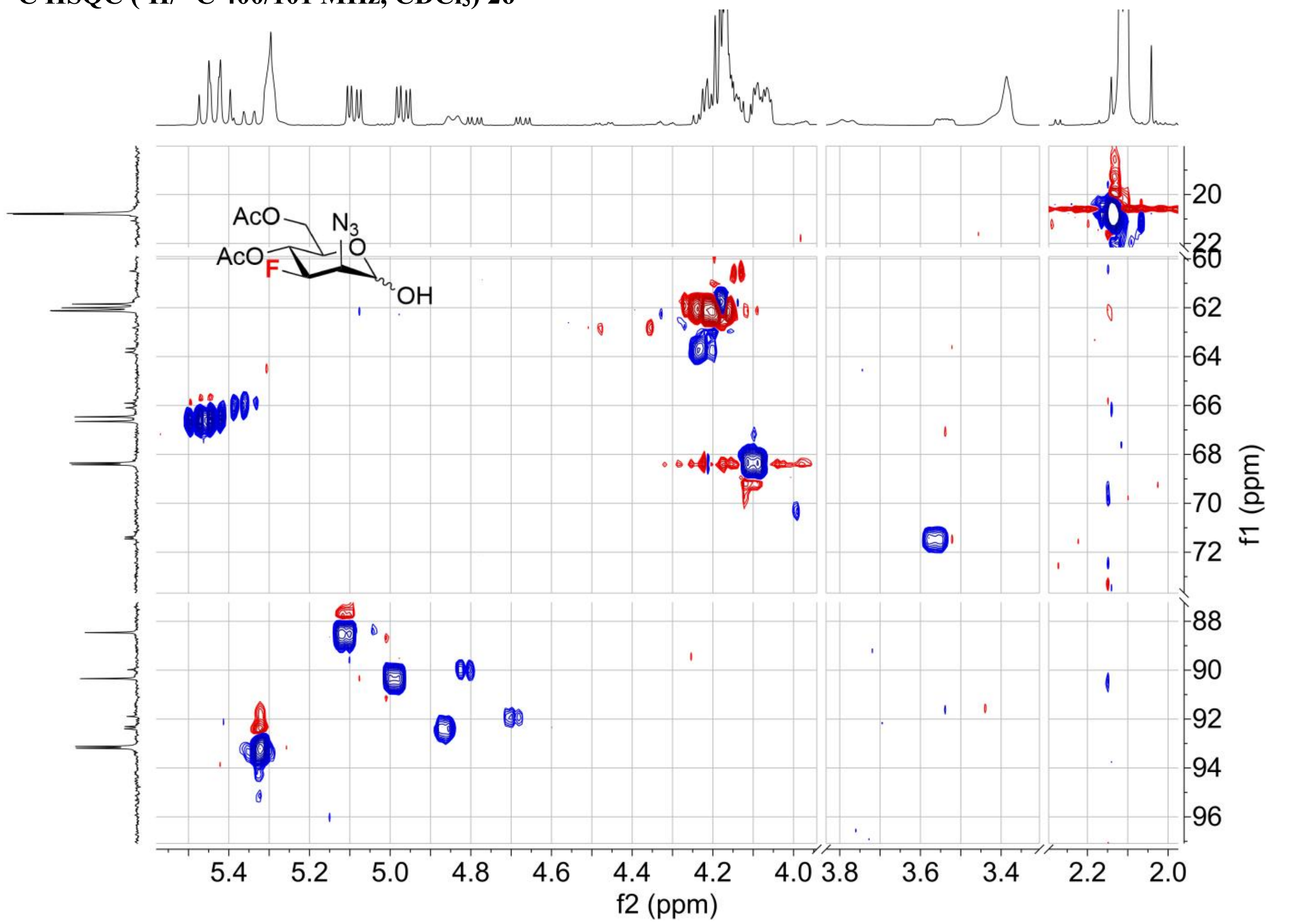

^{1}H-^{13}C HMBC (^{1}H/^{13}C 400/101 MHz, CDCl$_3$) 26

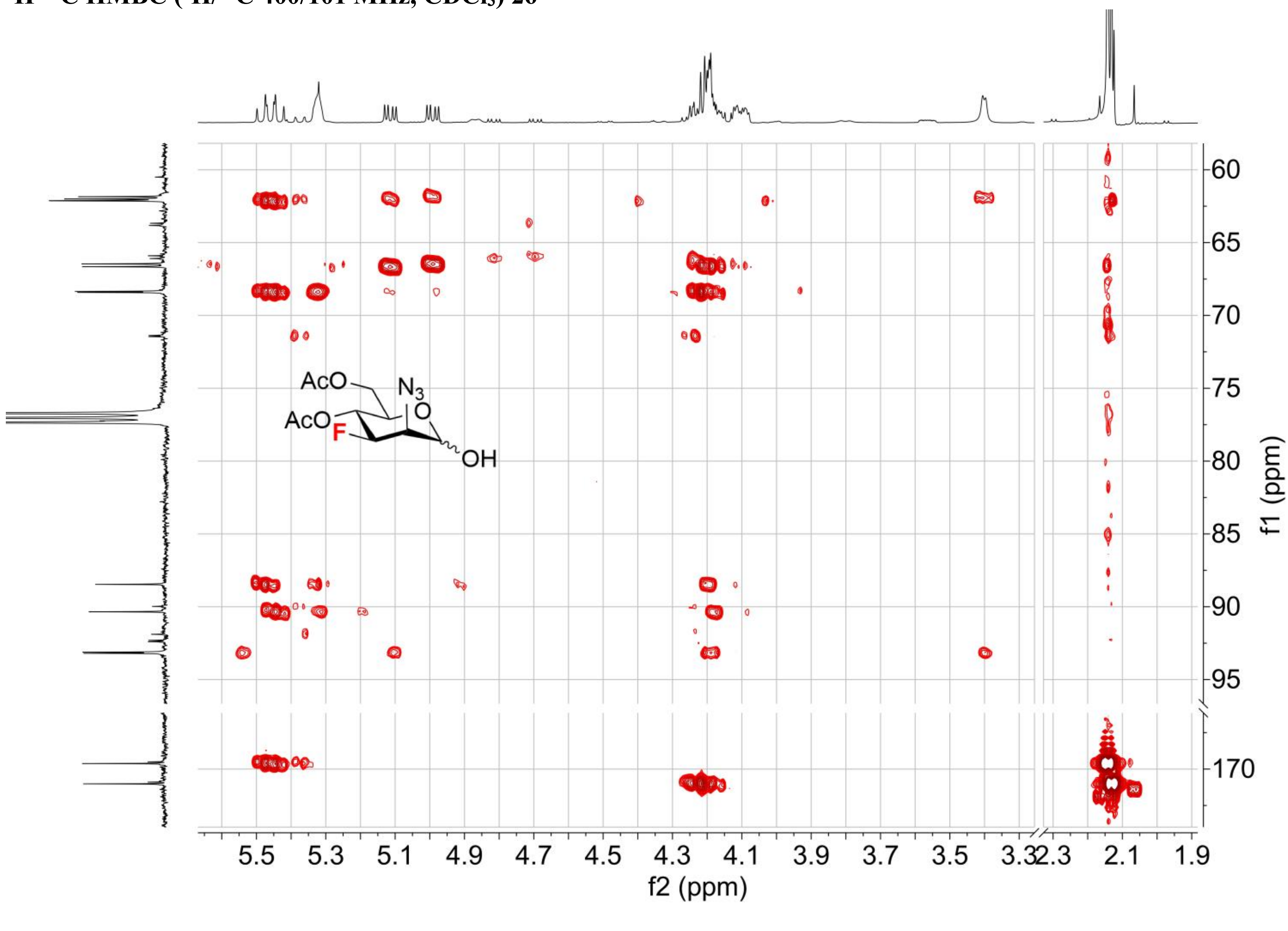

NMR COMPOUND 27

^{1}H NMR (400 MHz, CDCl$_3$) 27

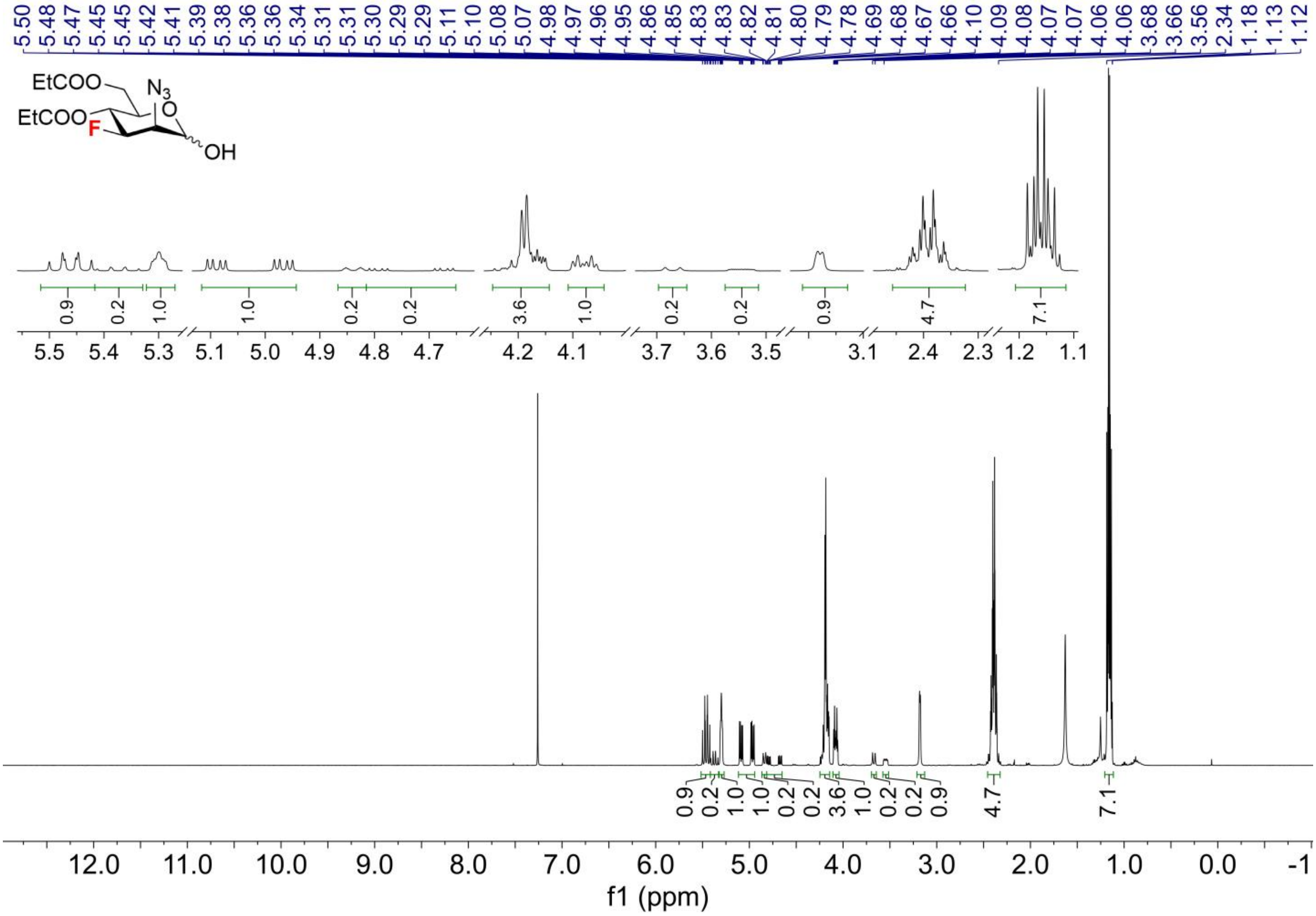

^{13}C{^{1}H} NMR (101 MHz, CDCl$_3$) 27

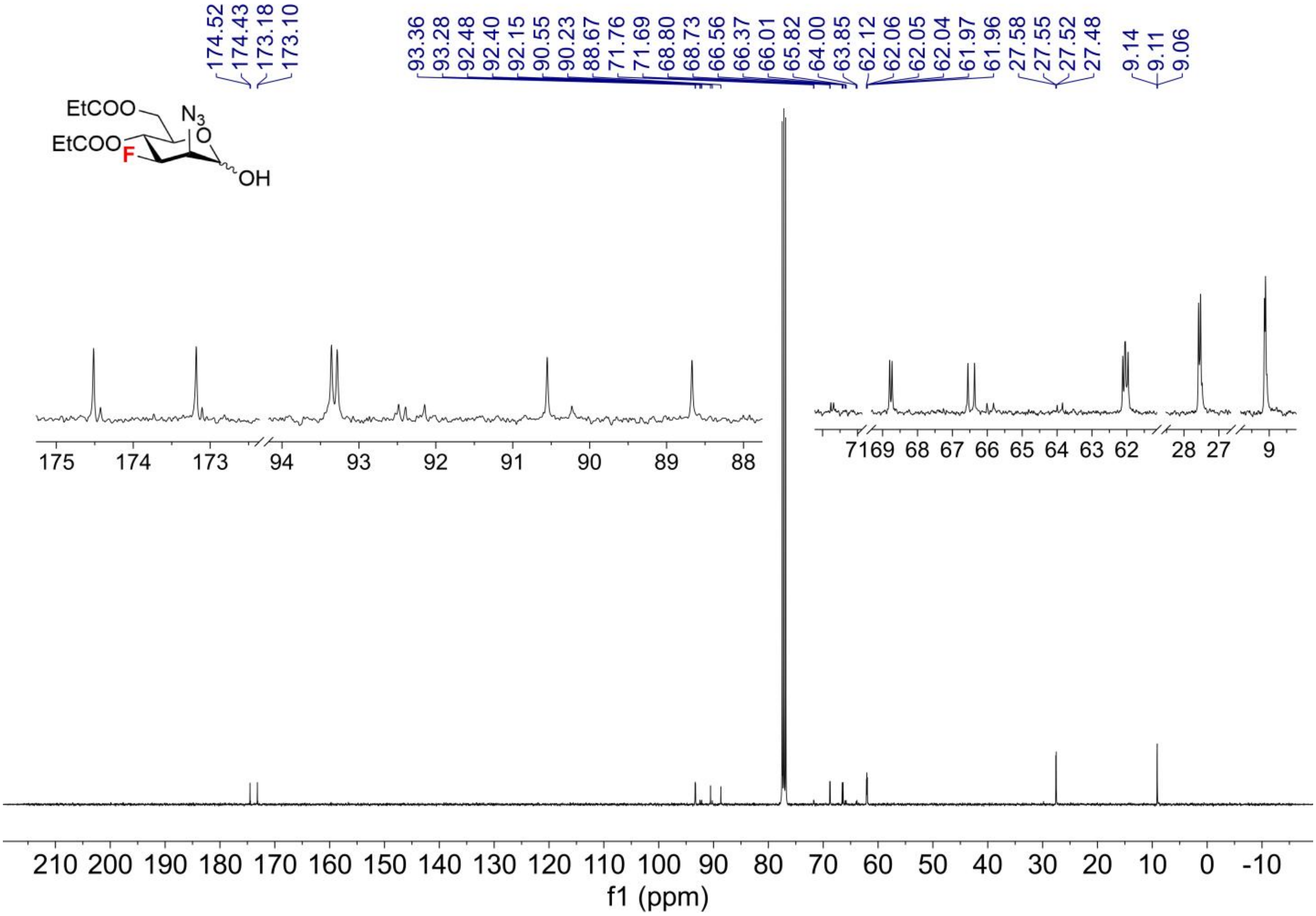

^{19}F NMR (376 MHz, $CDCl_3$) 27

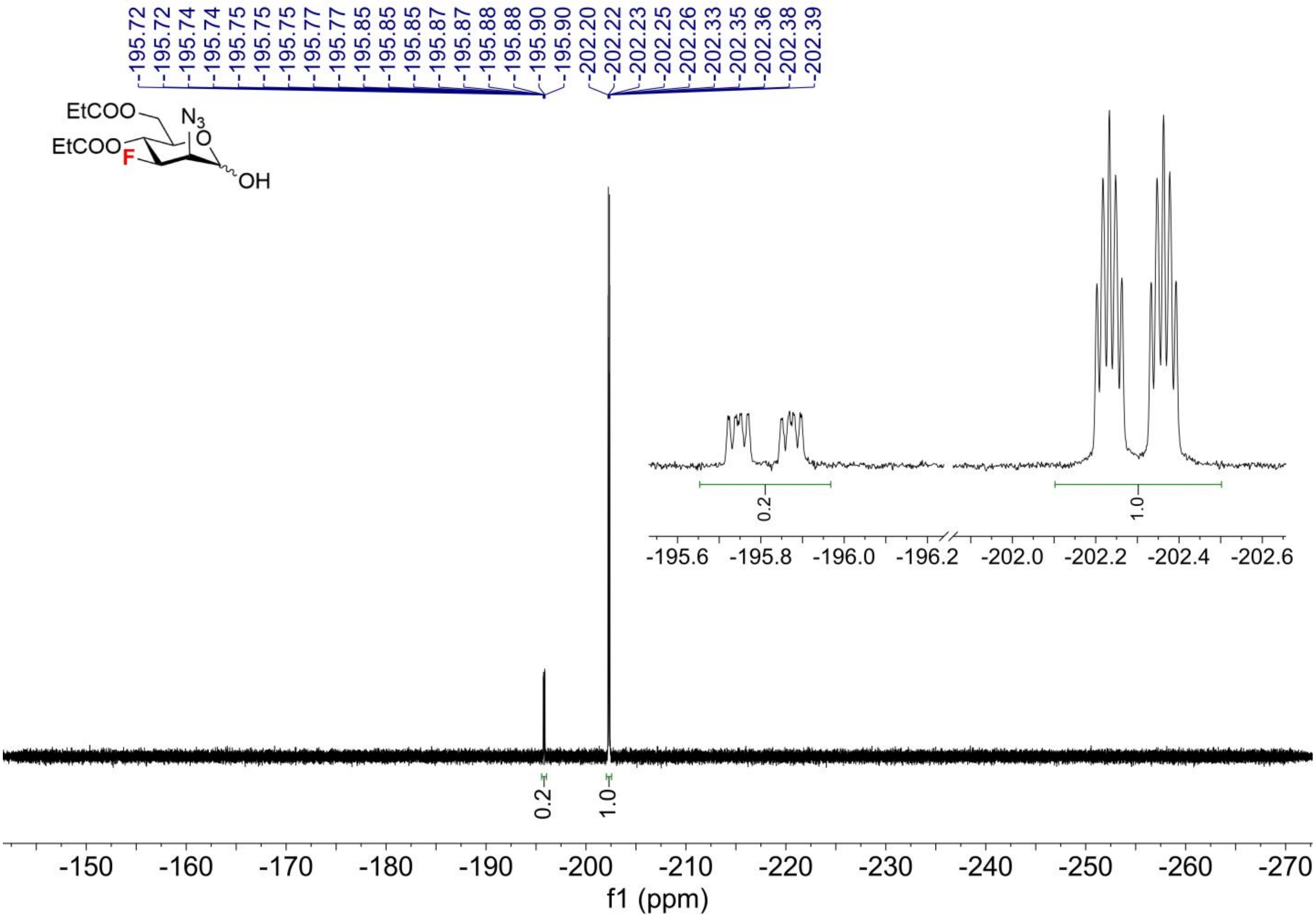

^{1}H-^{1}H COSY (400 MHz, $CDCl_3$) 27

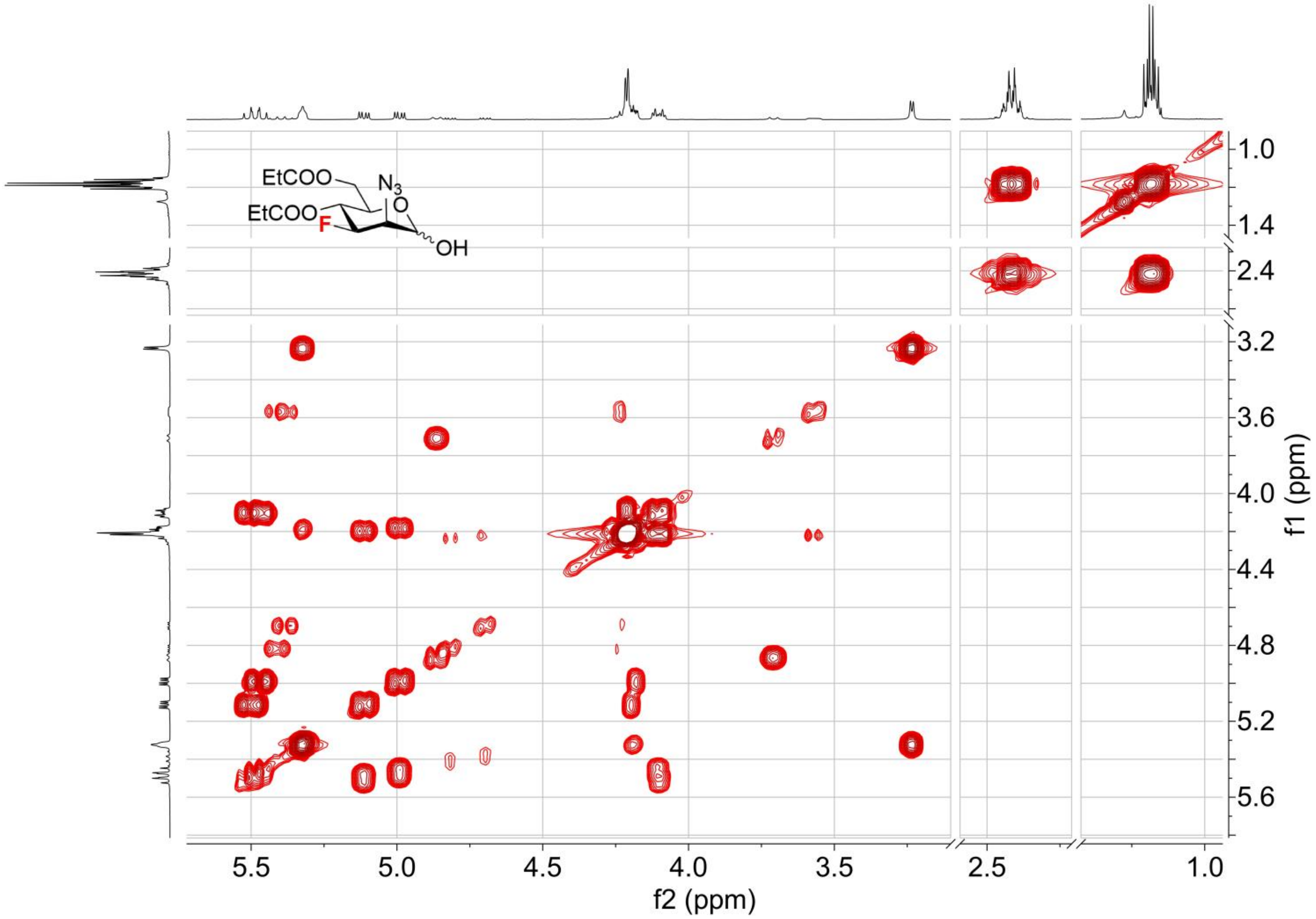

^{1}H-^{13}C HSQC (^{1}H/^{13}C 400/101 MHz, CDCl$_3$) 27

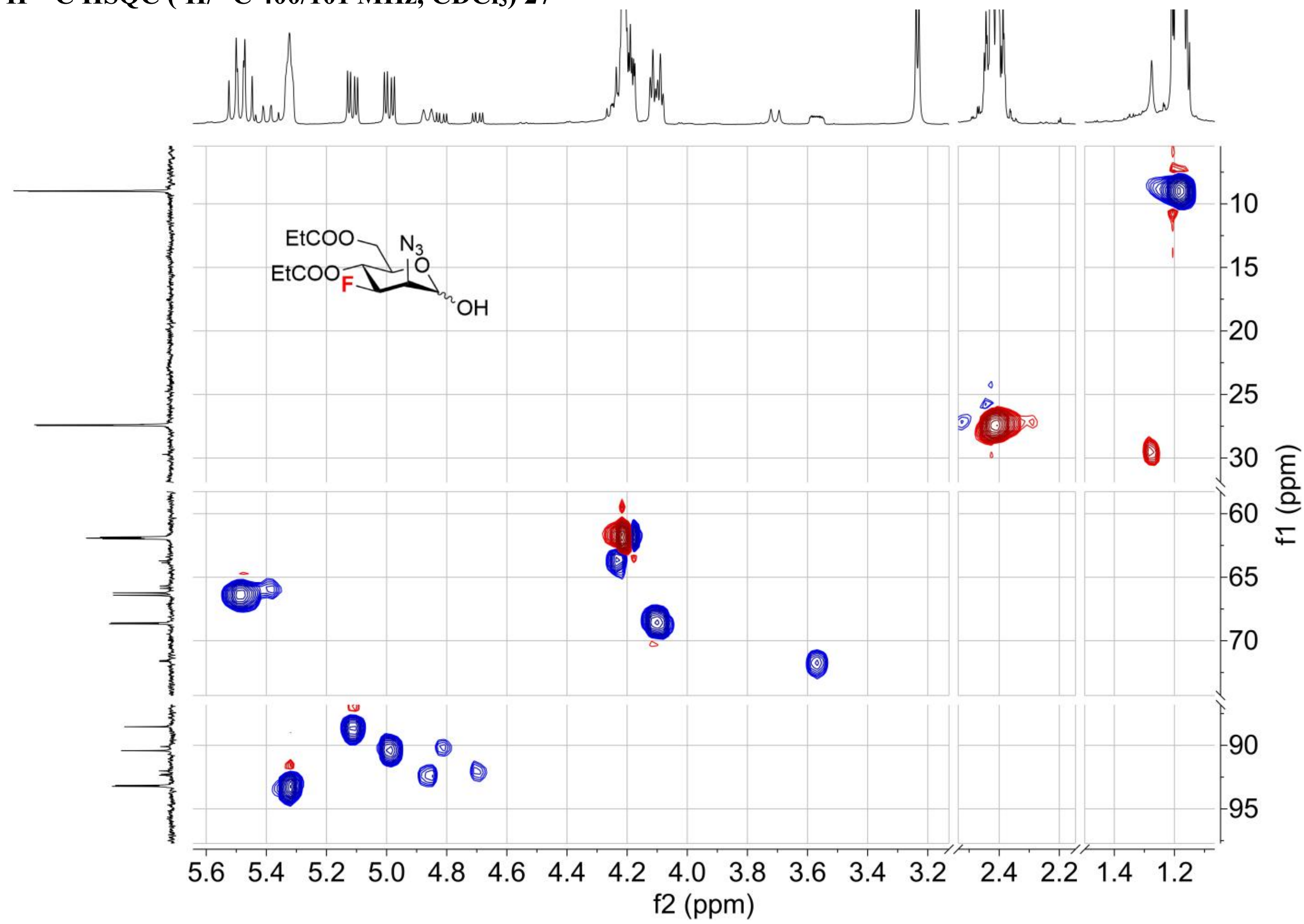

^{1}H-^{13}C HMBC (^{1}H/^{13}C 400/101 MHz, CDCl$_3$) 27

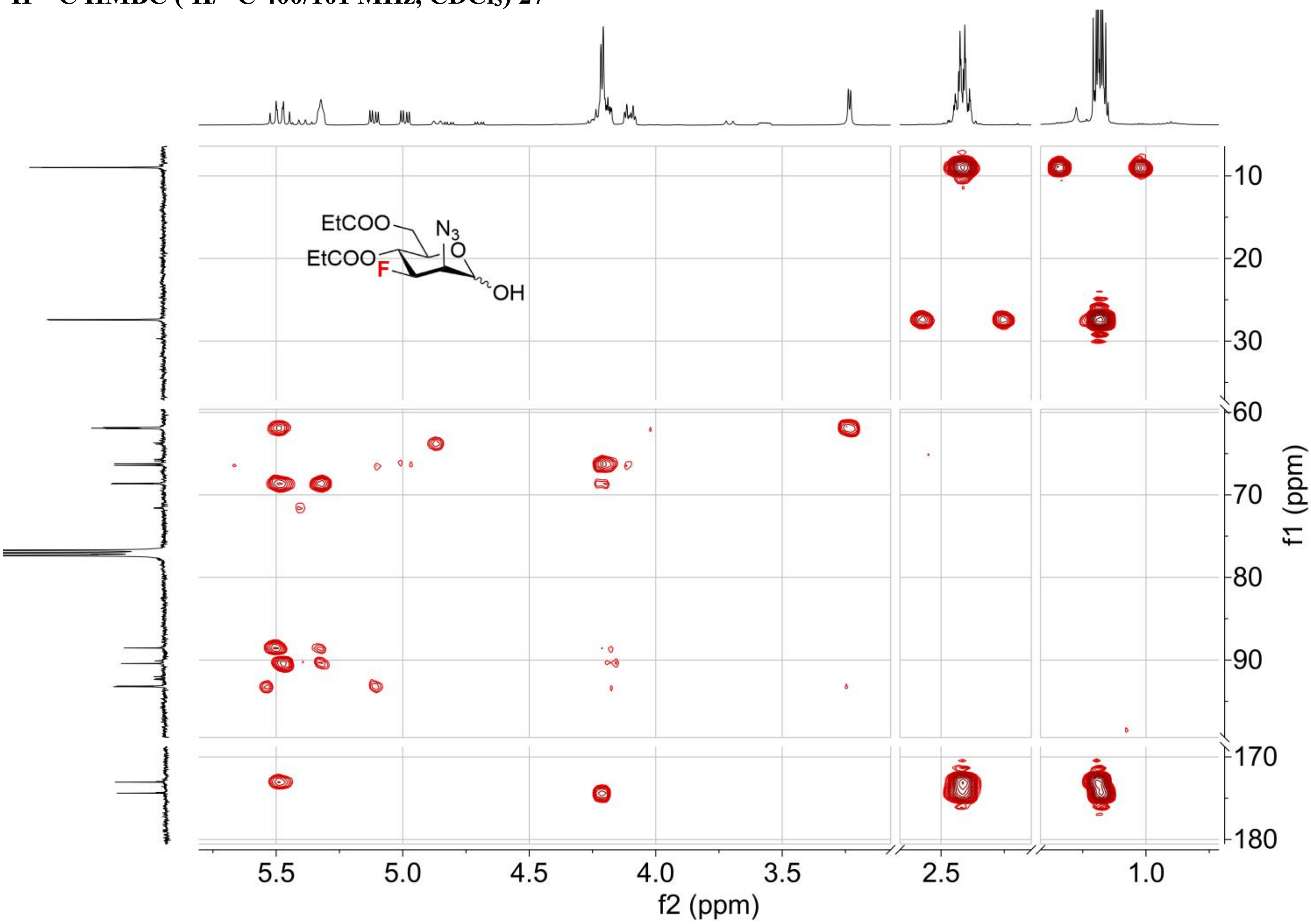

NMR COMPOUND 28

^{1}H NMR (400 MHz, CDCl$_3$) 28

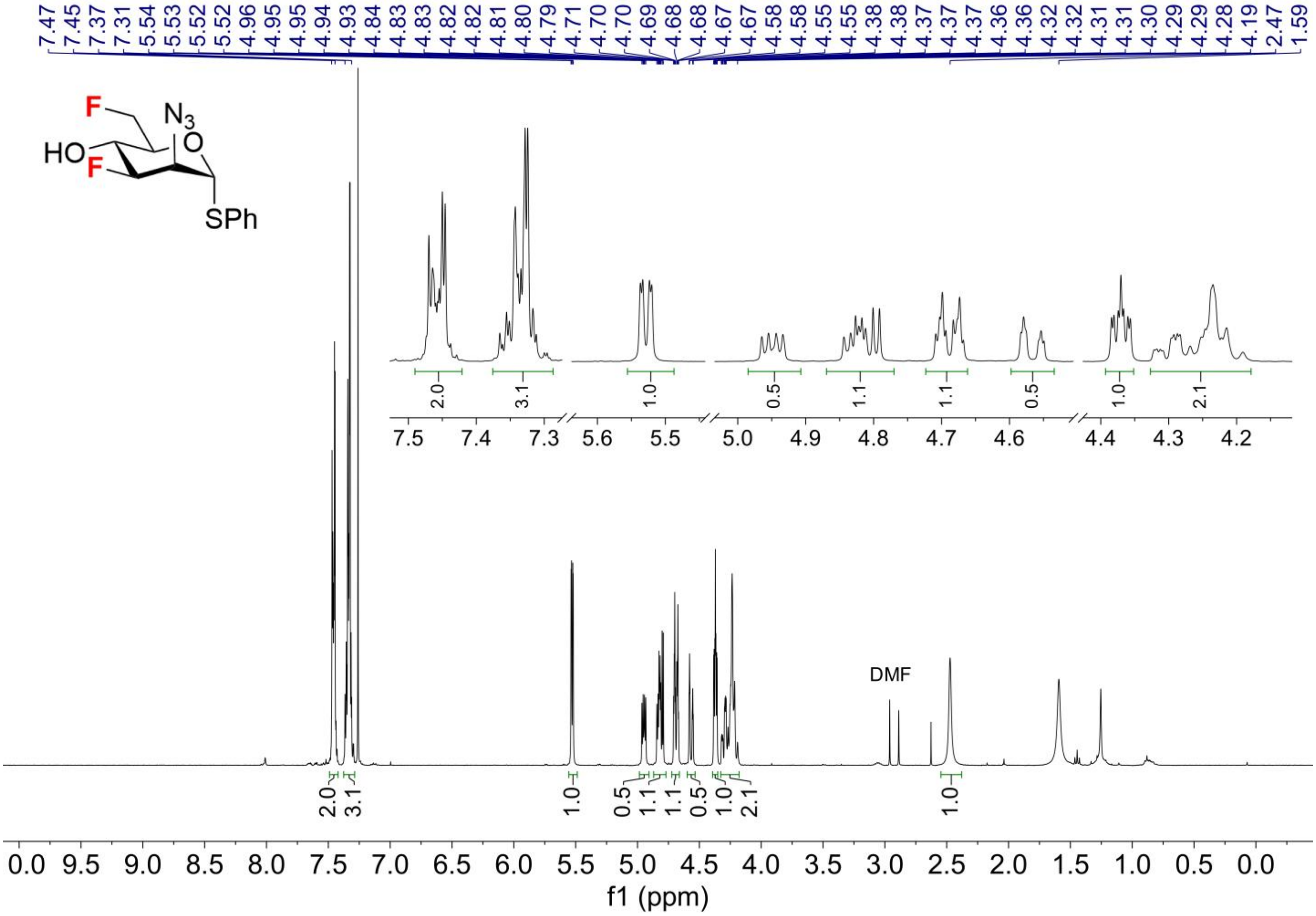

^{13}C{^{1}H} NMR (101 MHz, CDCl$_3$) 28

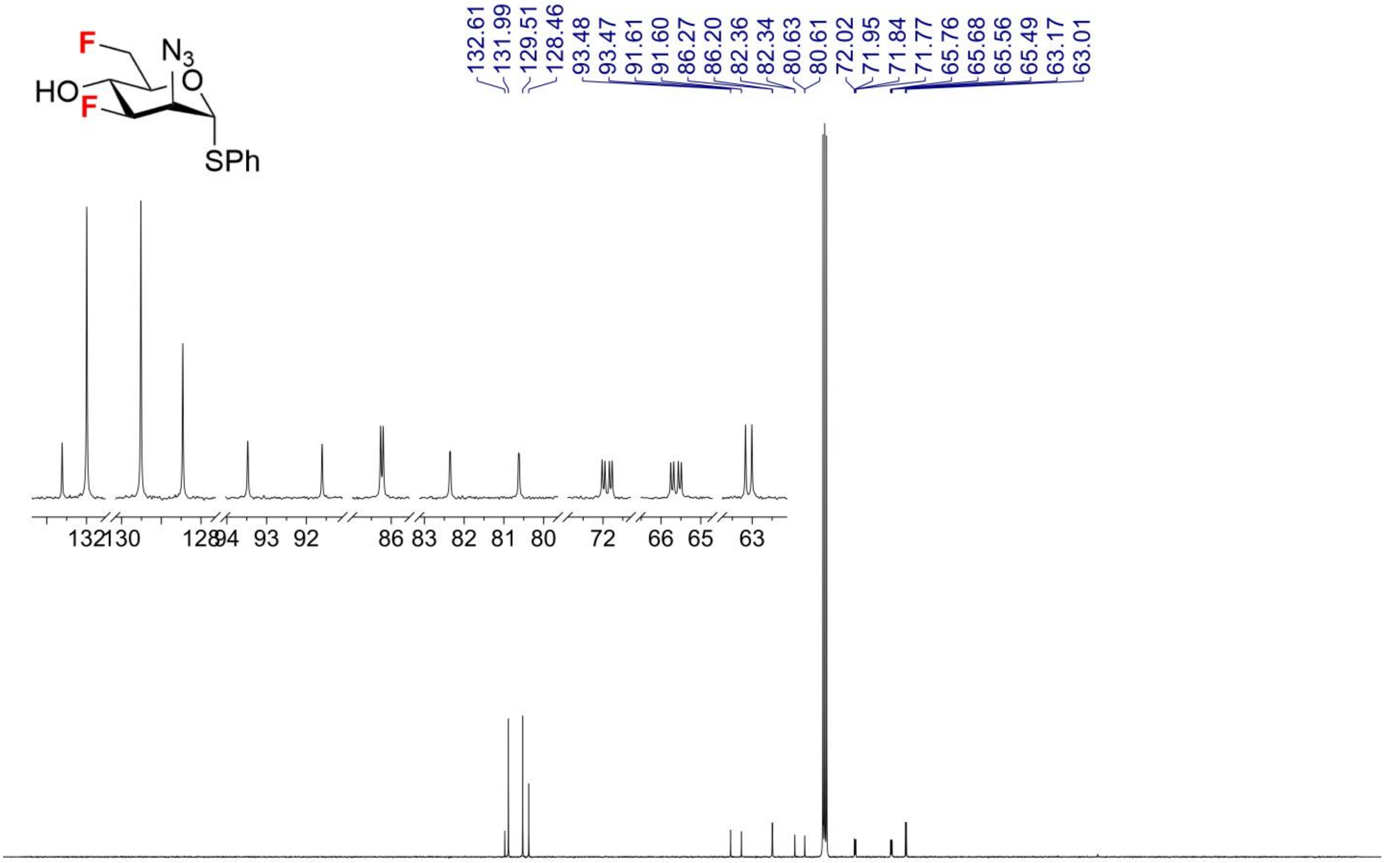

^{19}F NMR (376 MHz, $CDCl_3$) 28

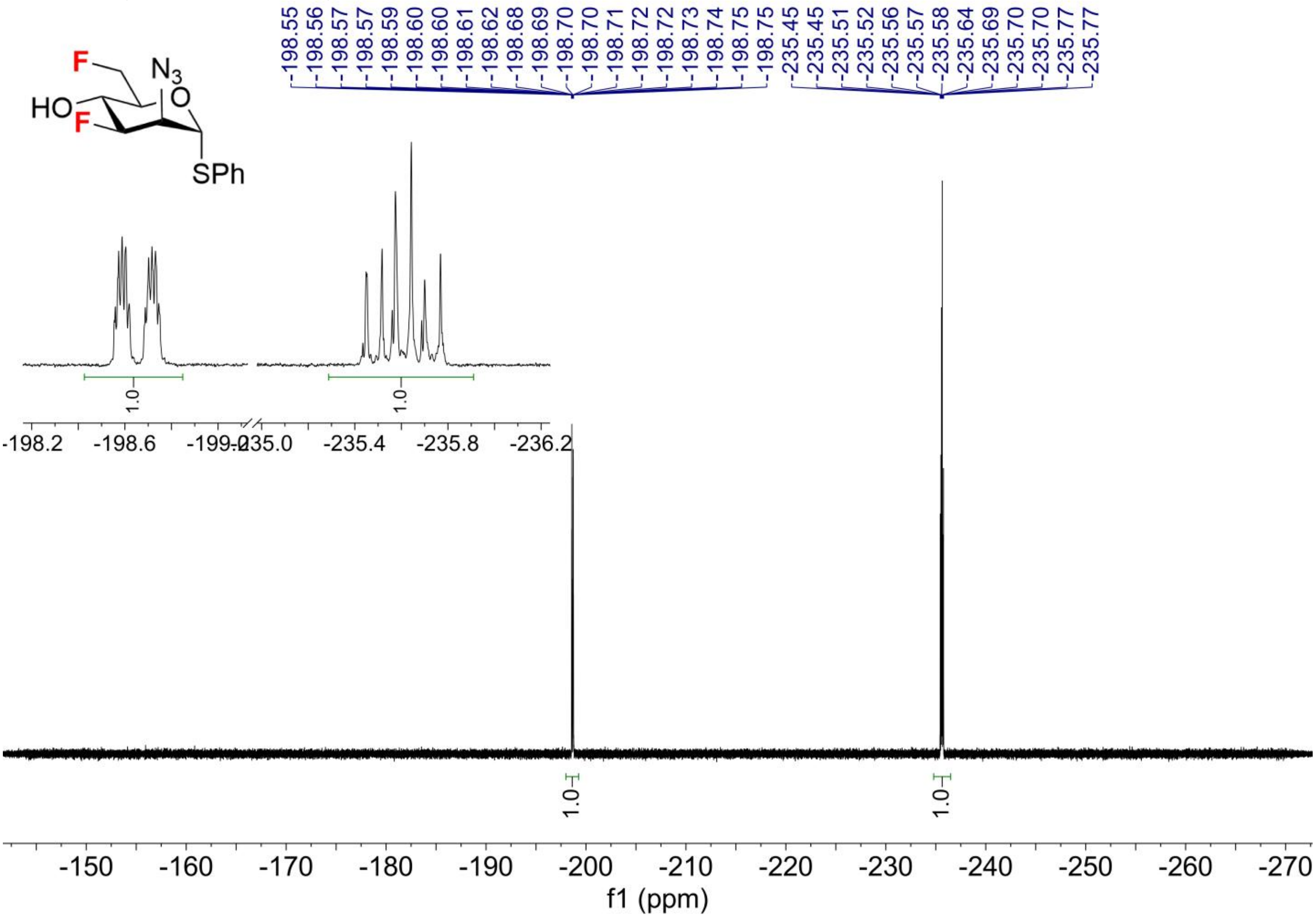

^{1}H-^{1}H COSY (400 MHz, $CDCl_3$) 28

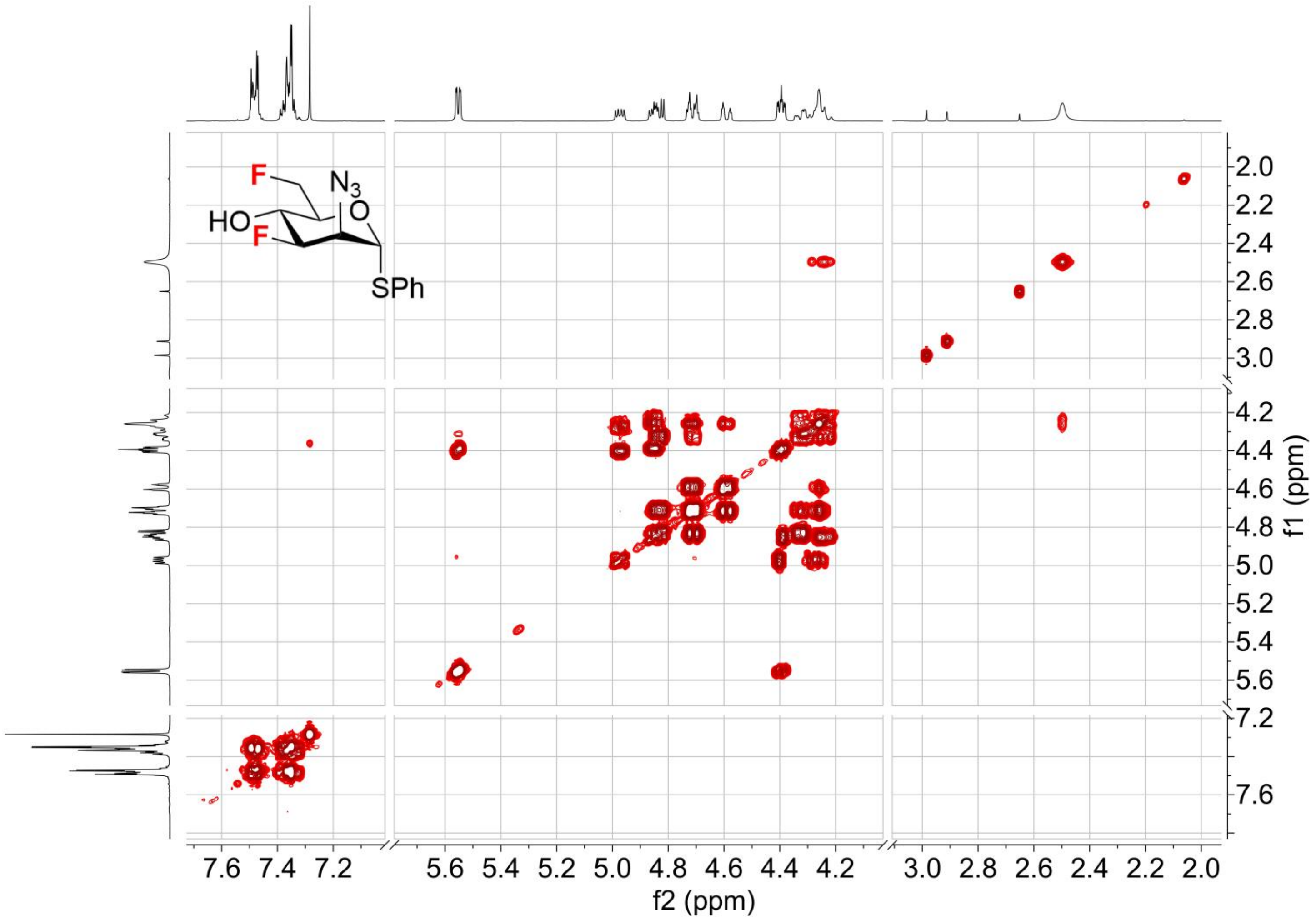

^{1}H-^{13}C HSQC (^{1}H/^{13}C 400/101 MHz, CDCl$_3$) 28

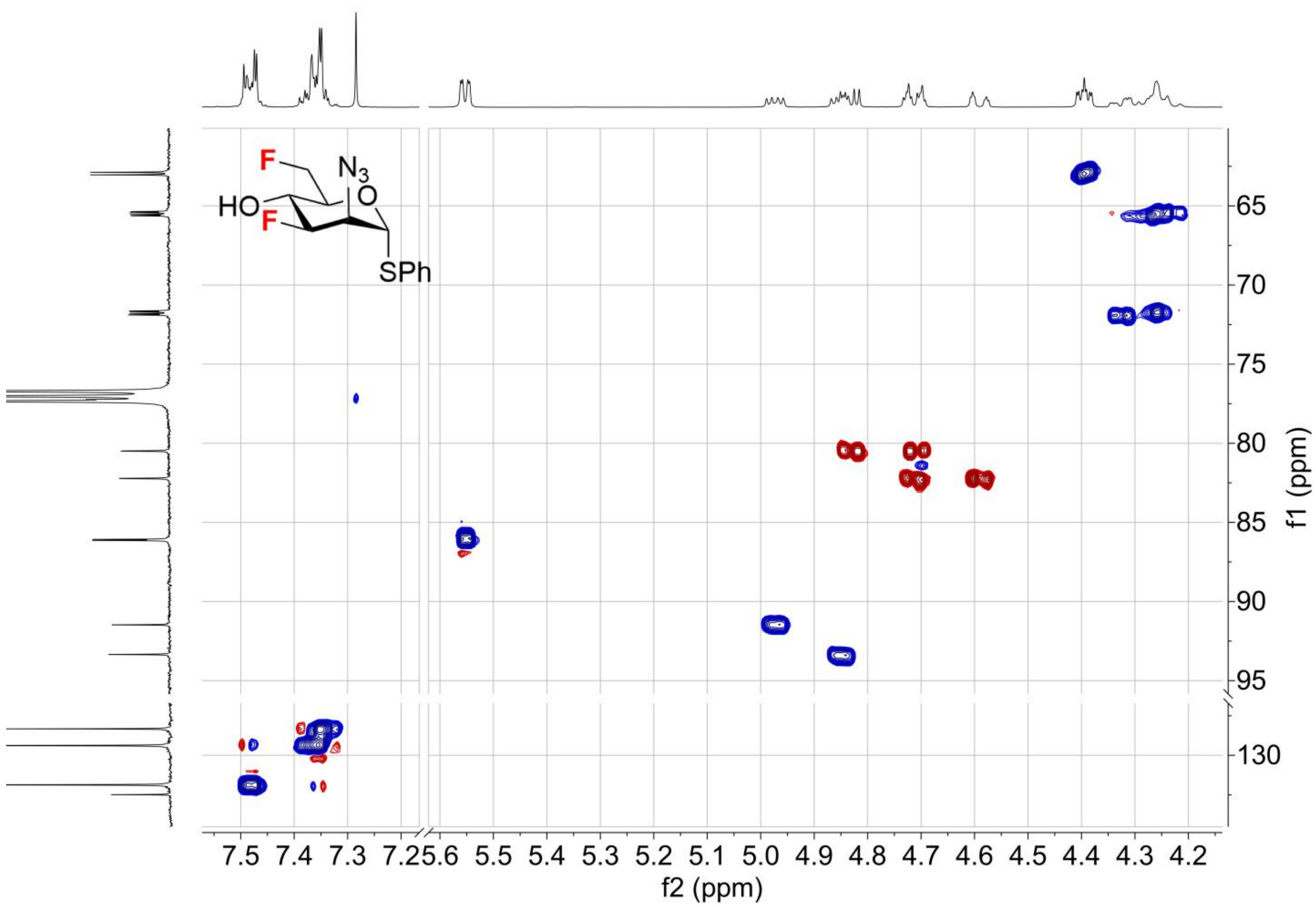

^{1}H-^{13}C HMBC (^{1}H/^{13}C 400/101 MHz, CDCl$_3$) 28

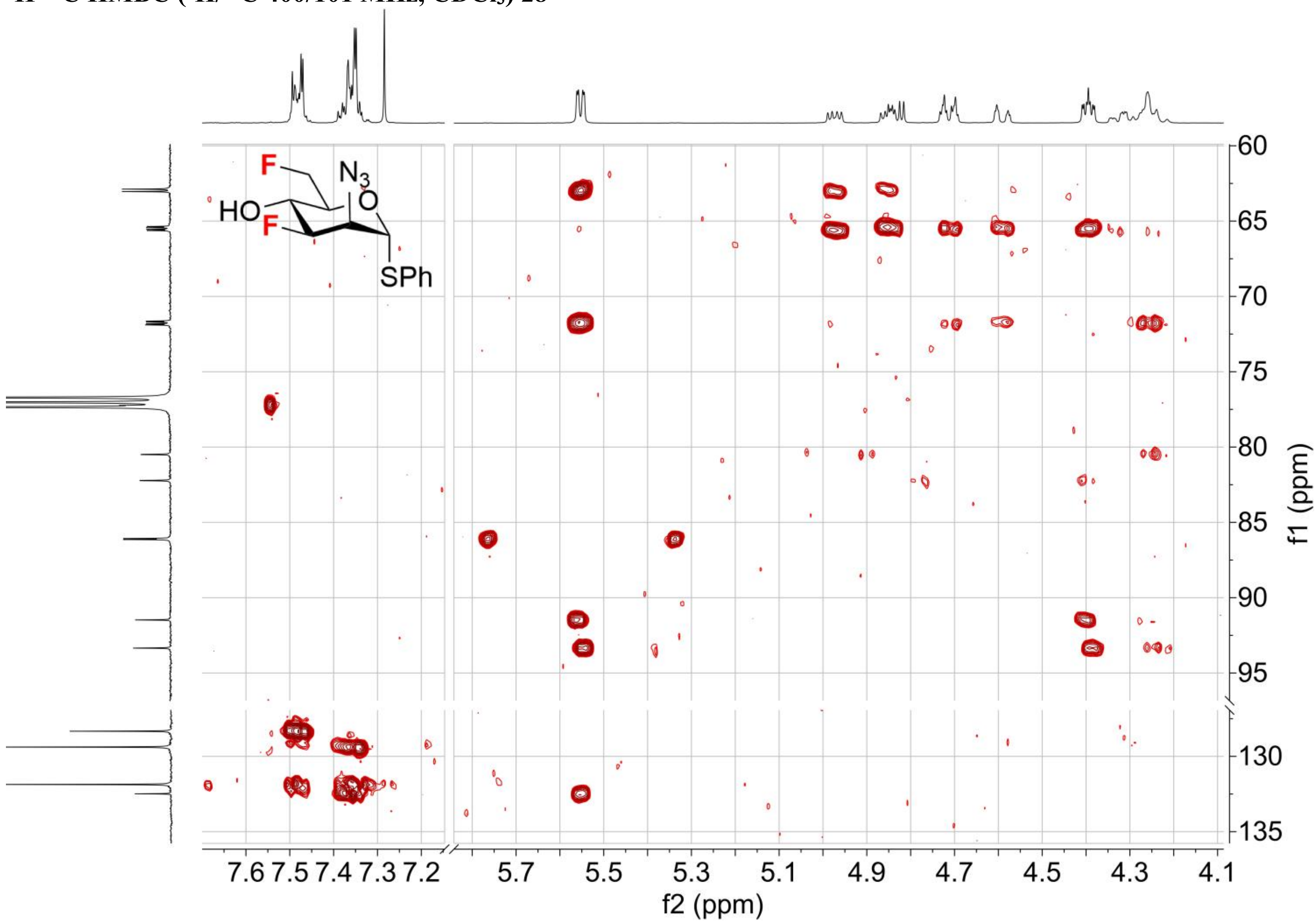

NMR COMPOUND 29

^{1}H NMR (400 MHz, $CDCl_3$) 29

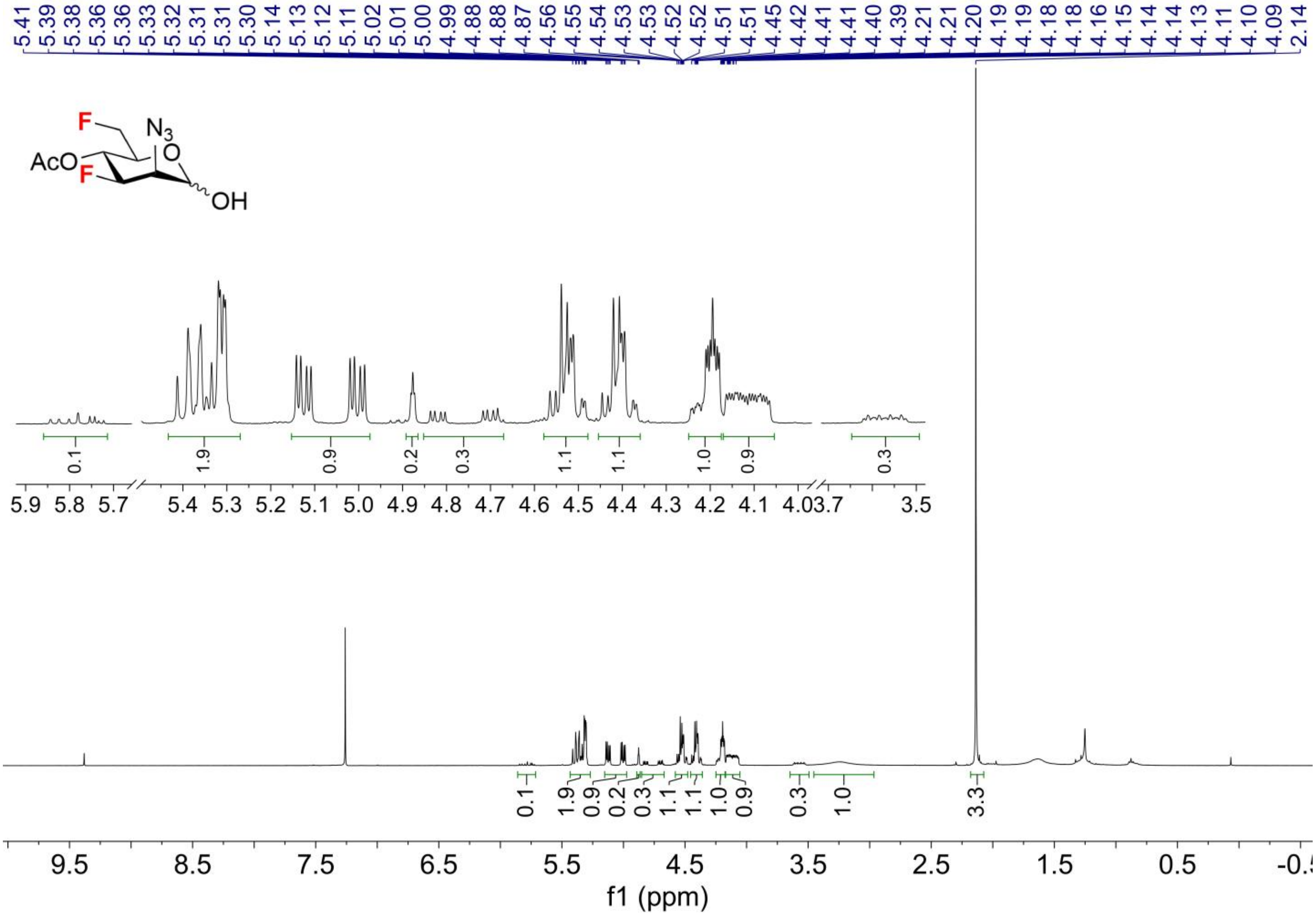

^{13}C{^{1}H} NMR (101 MHz, $CDCl_3$) 29

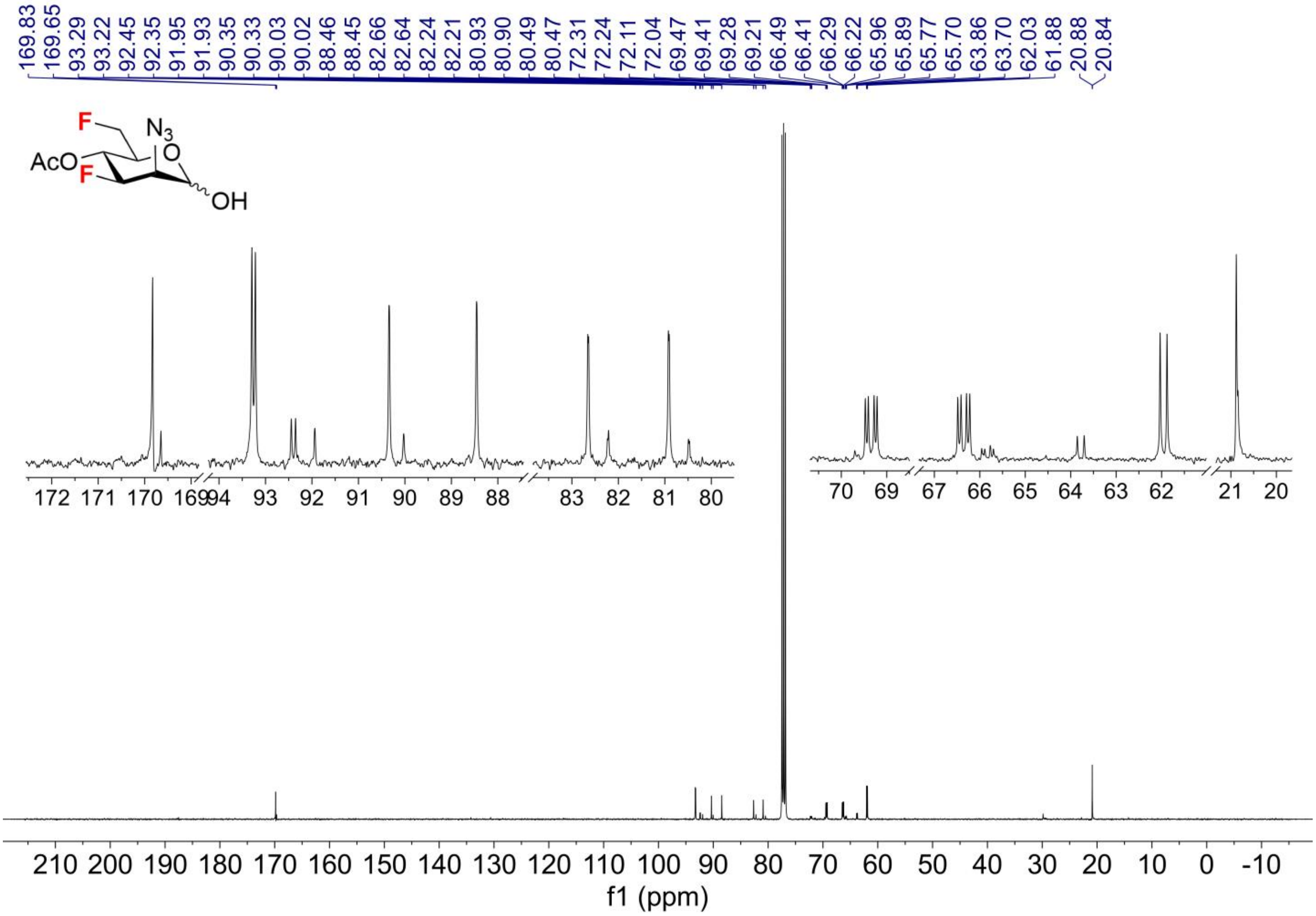

^{19}F NMR (376 MHz, CDCl$_3$) 29

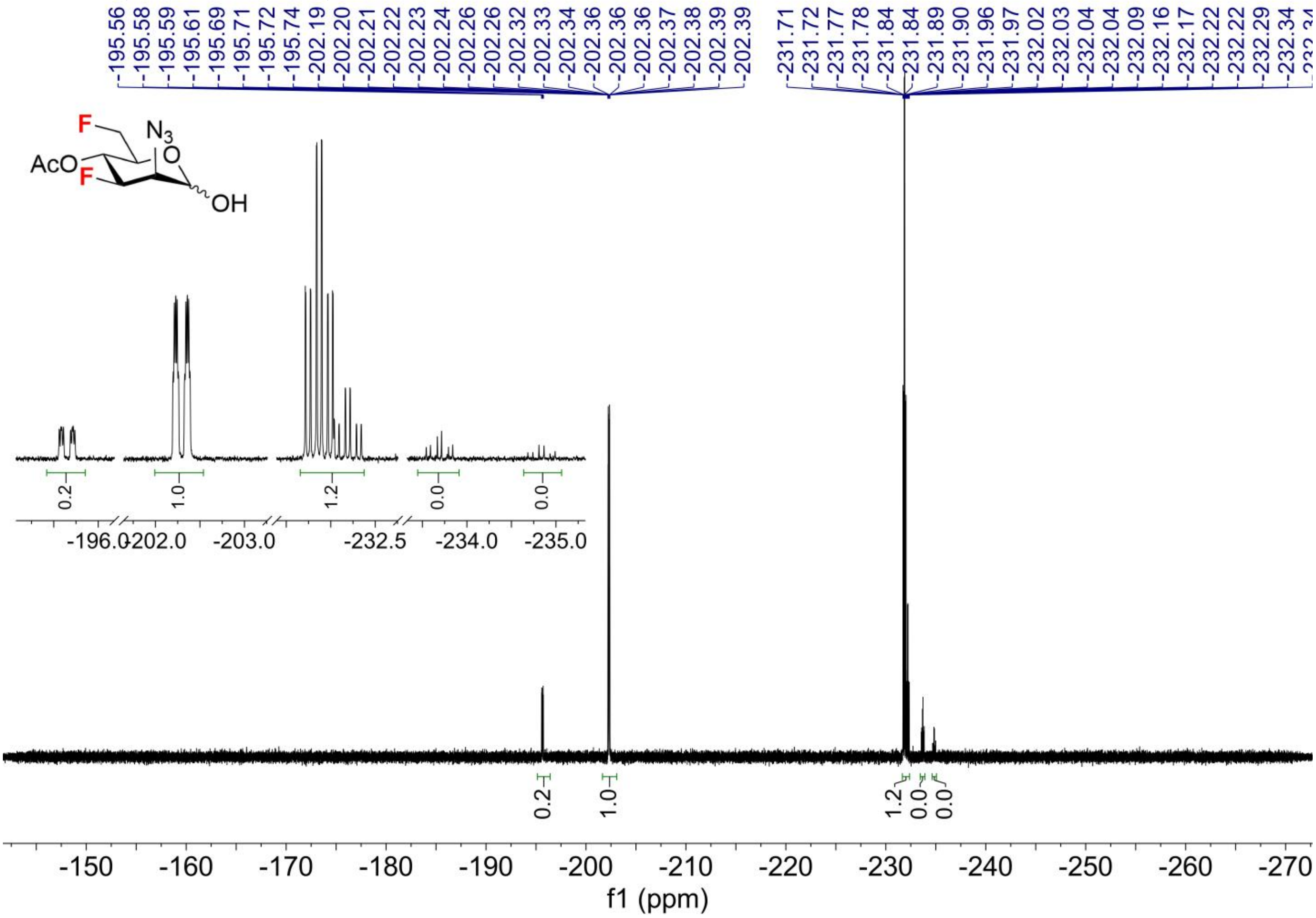

^{1}H-^{1}H COSY (400 MHz, CDCl$_3$) 29

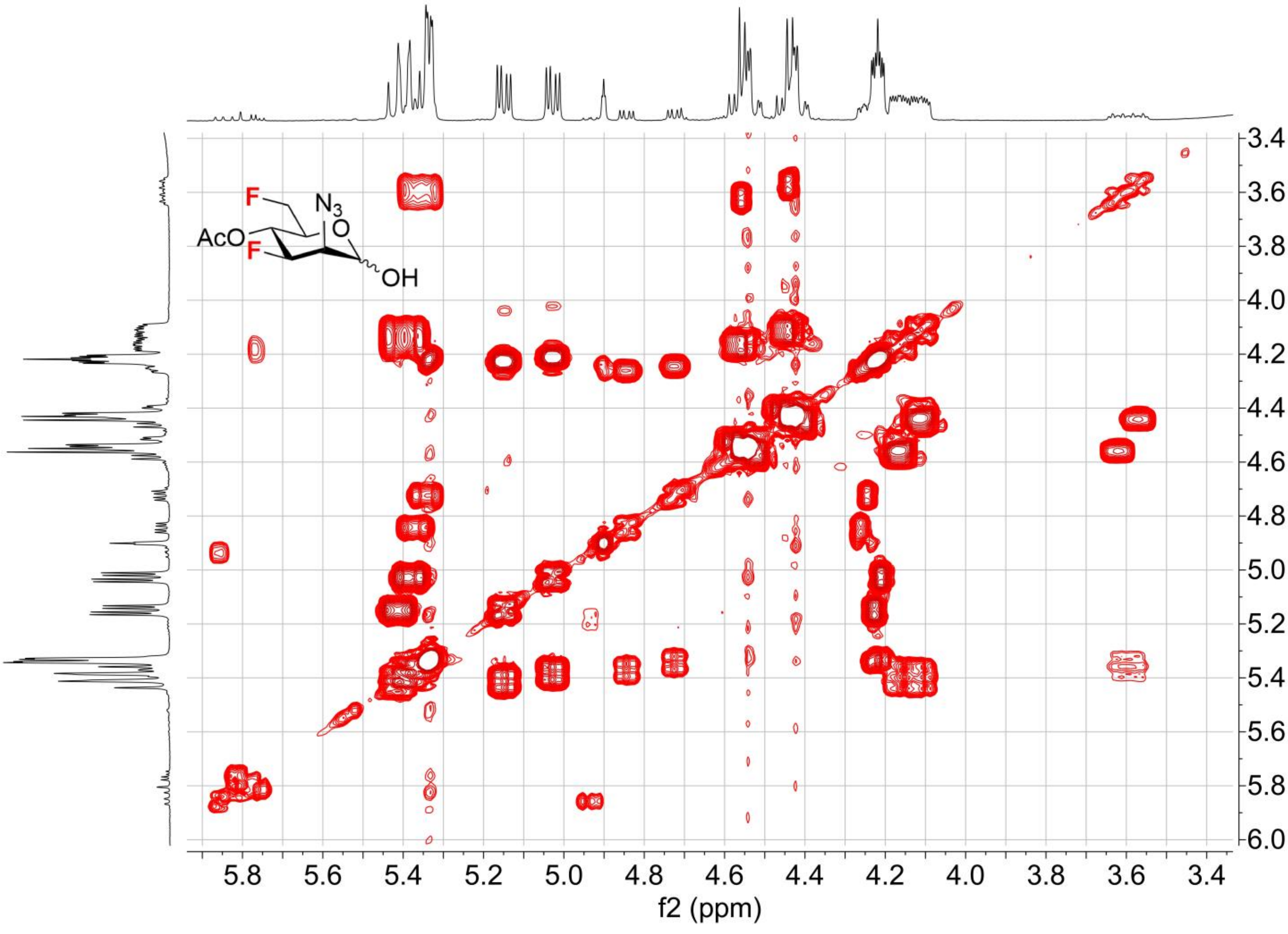

^1H-^{13}C HSQC (^1H/^{13}C 400/101 MHz, $CDCl_3$) 29

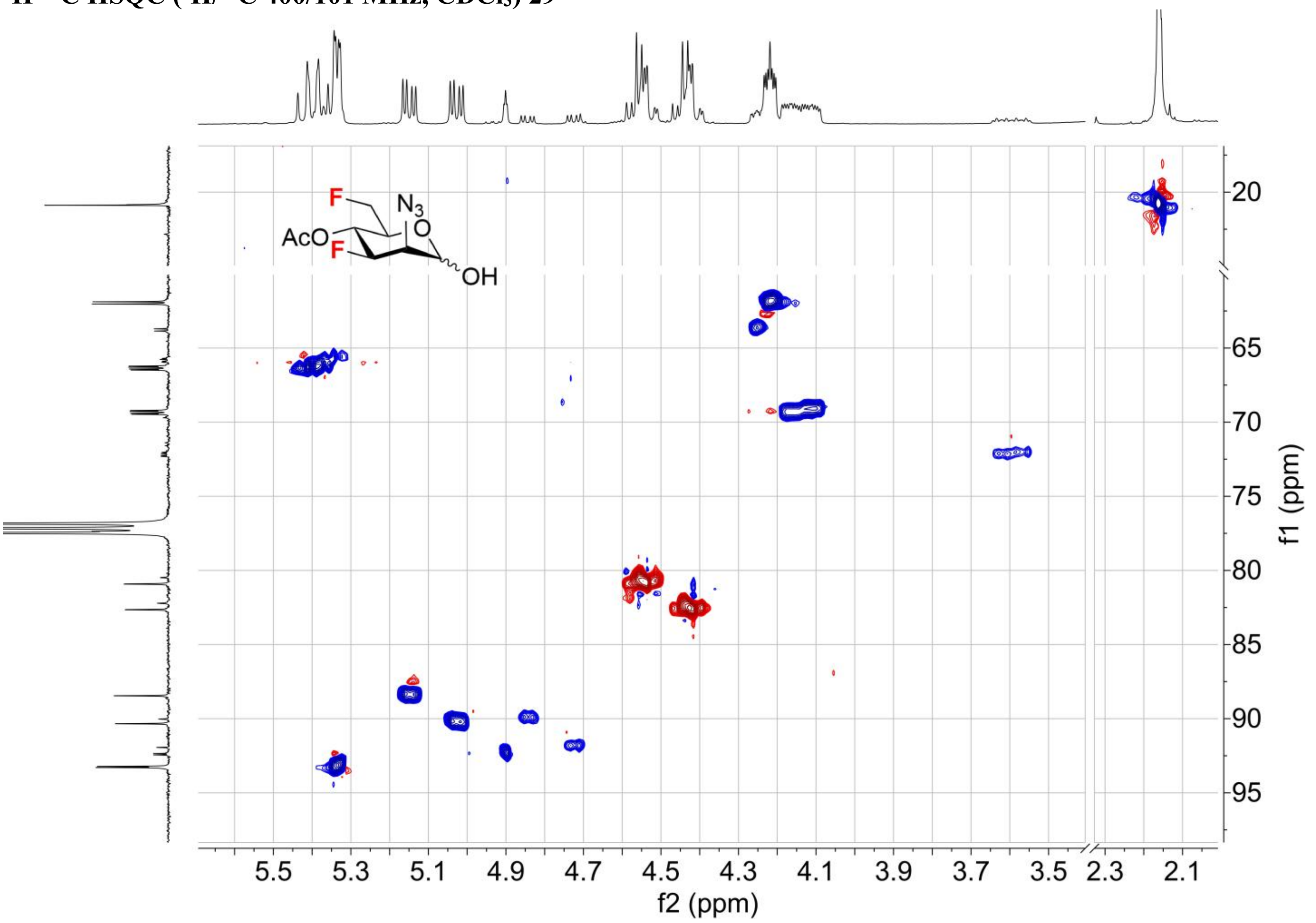

^1H-^{13}C HMBC (^1H/^{13}C 400/101 MHz, $CDCl_3$) 29

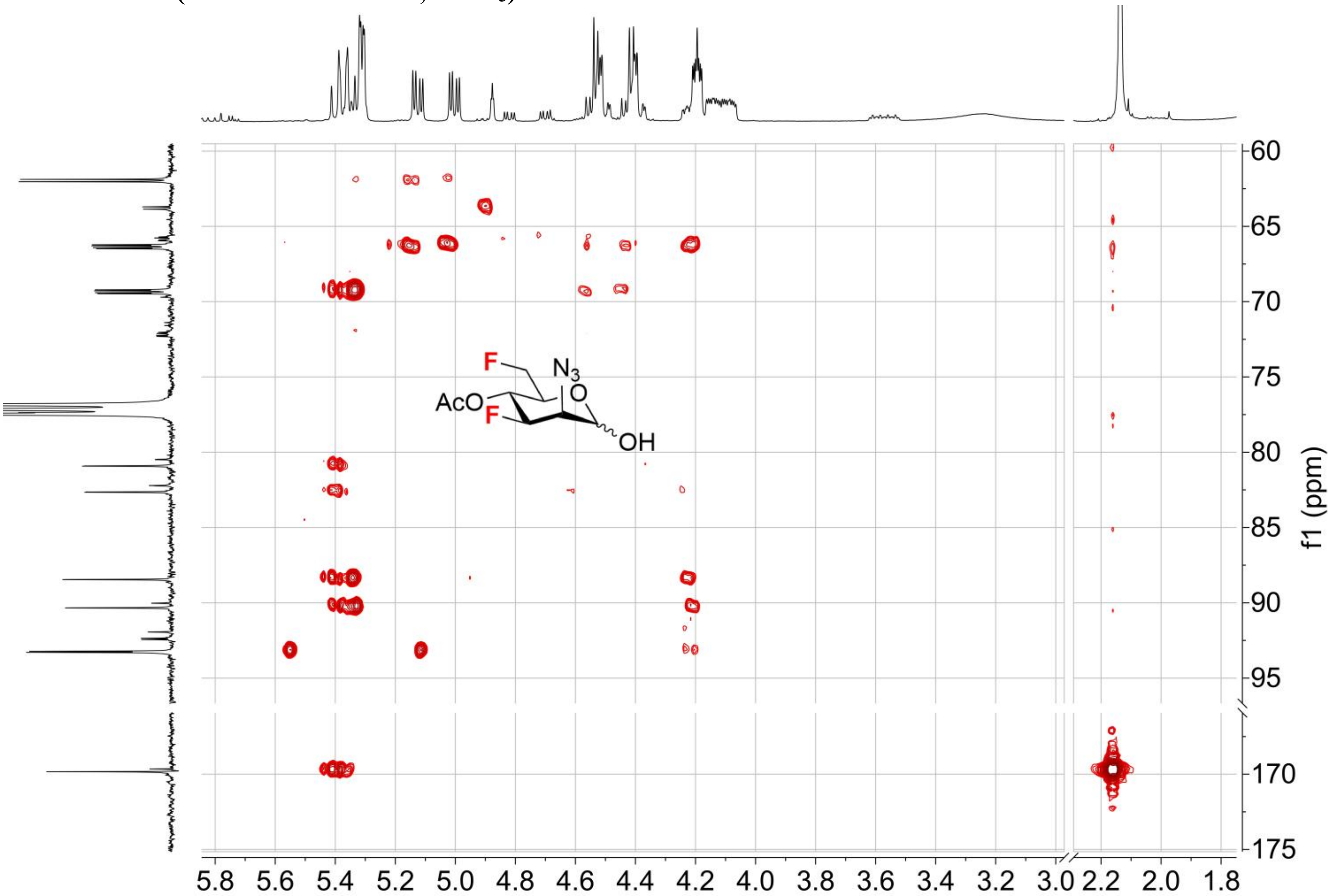

NMR COMPOUND 31

^{1}H NMR (400 MHz, CDCl$_3$) 31

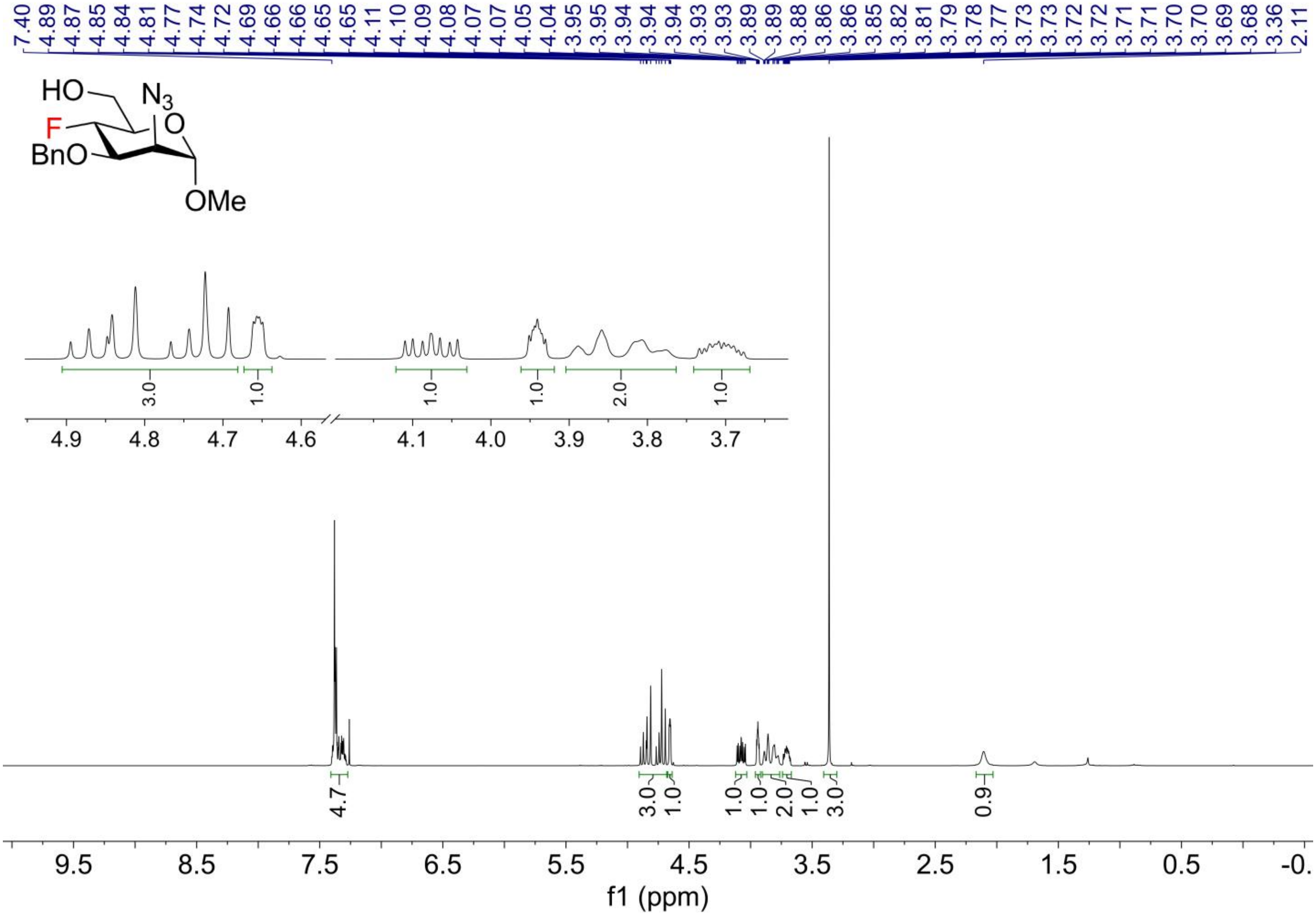

^{13}C{^{1}H} NMR (101 MHz, CDCl$_3$) 31

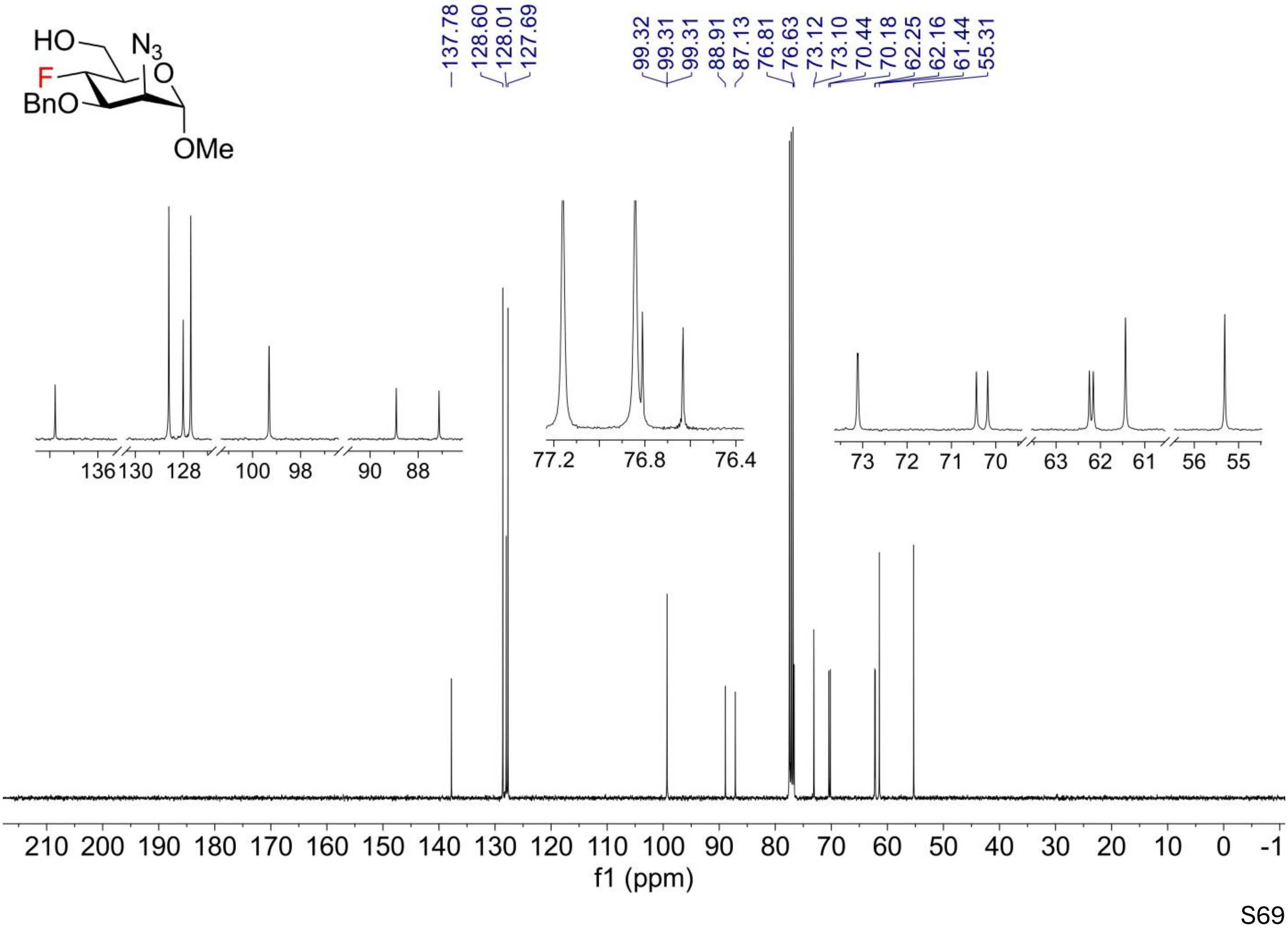

^{19}F NMR (376 MHz, CDCl3) 31

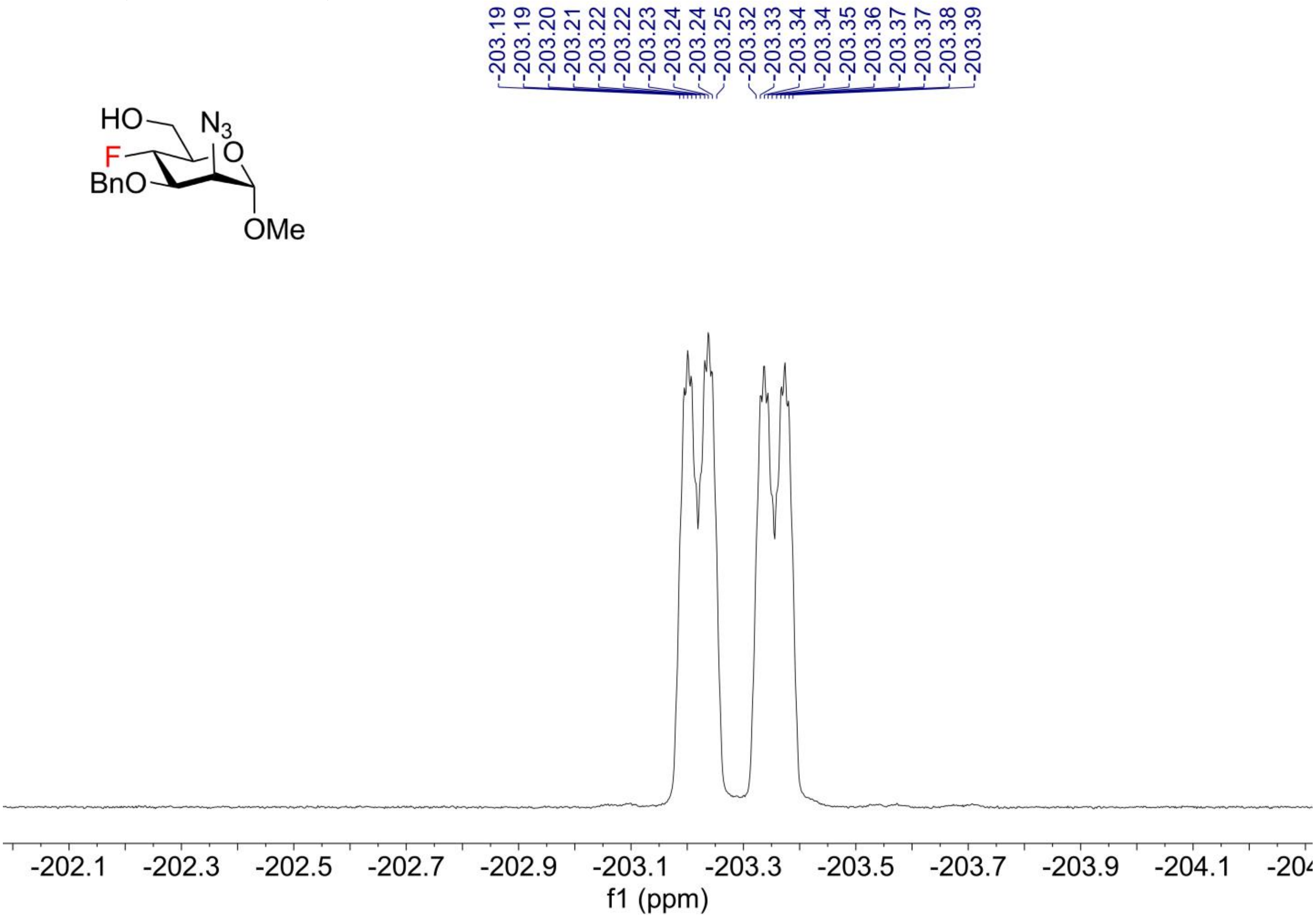

^{1}H-^{1}H COSY (400 MHz, CDCl3) 31

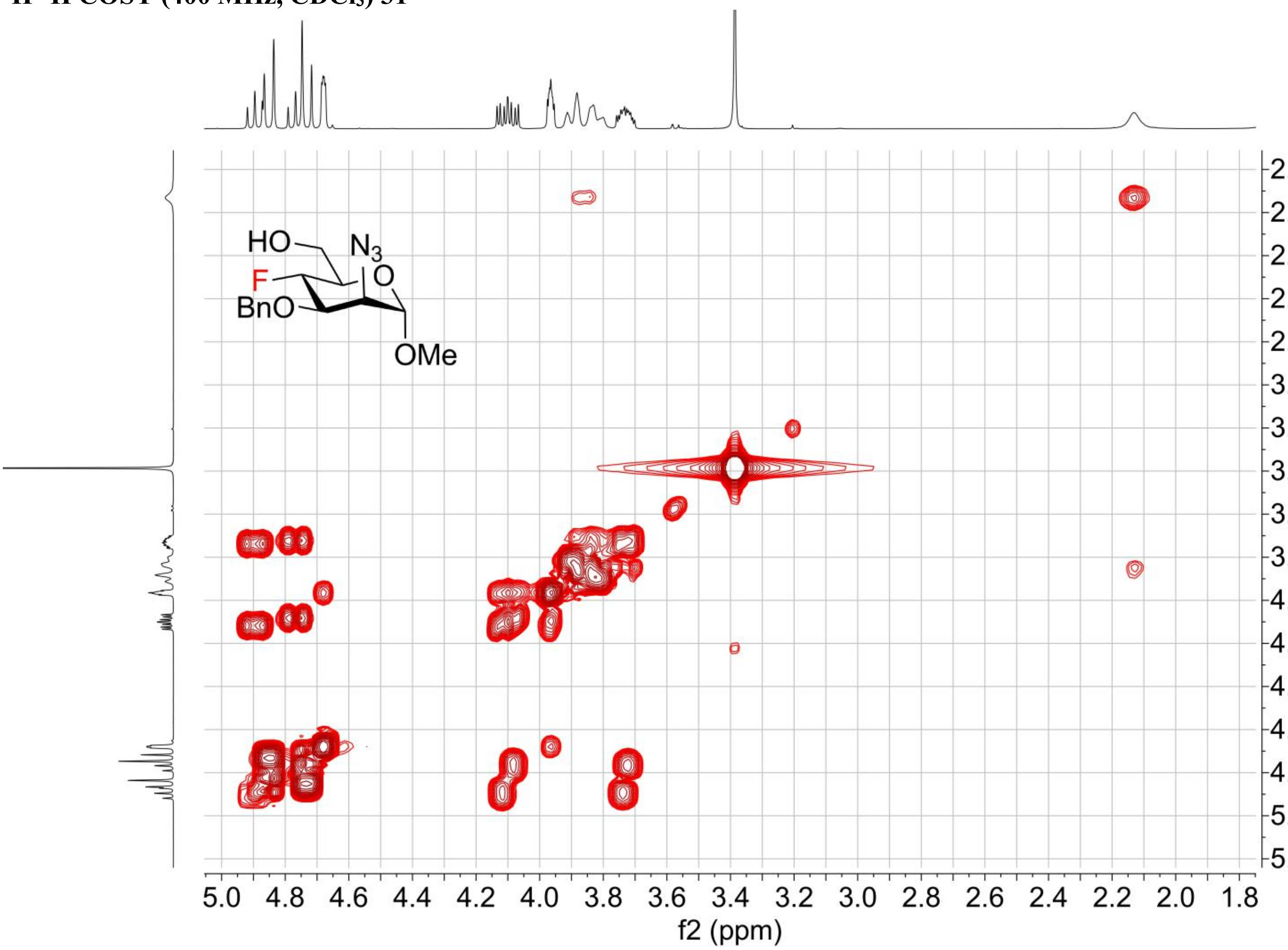

^{1}H-^{13}C HSQC ($^{1}H/^{13}C$ 400/101 MHz, $CDCl_3$) 31

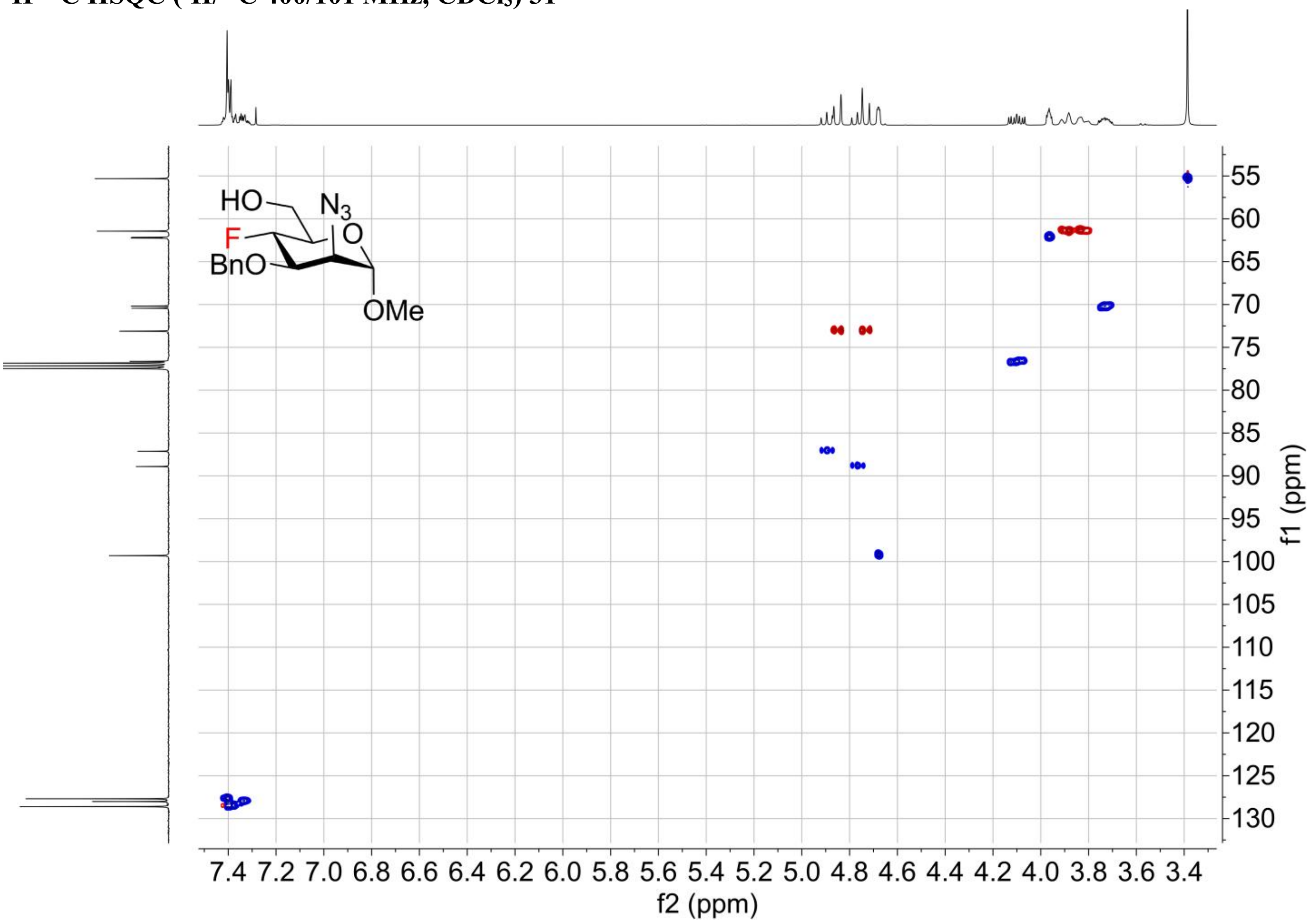

NMR COMPOUND 32

^{1}H NMR (400 MHz, $CDCl_3$) 32

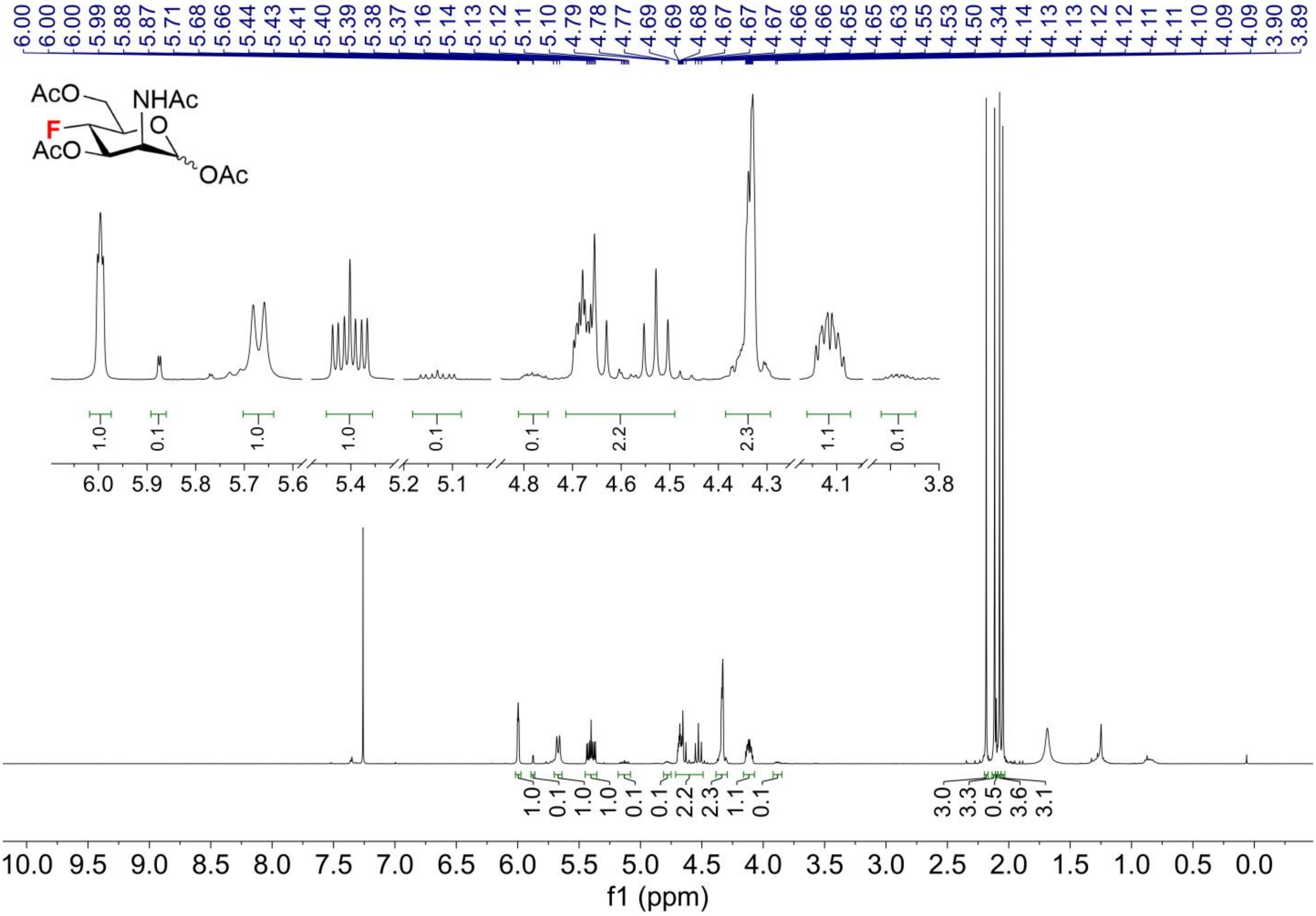

^{13}C{^{1}H} NMR (101 MHz, $CDCl_3$) 32

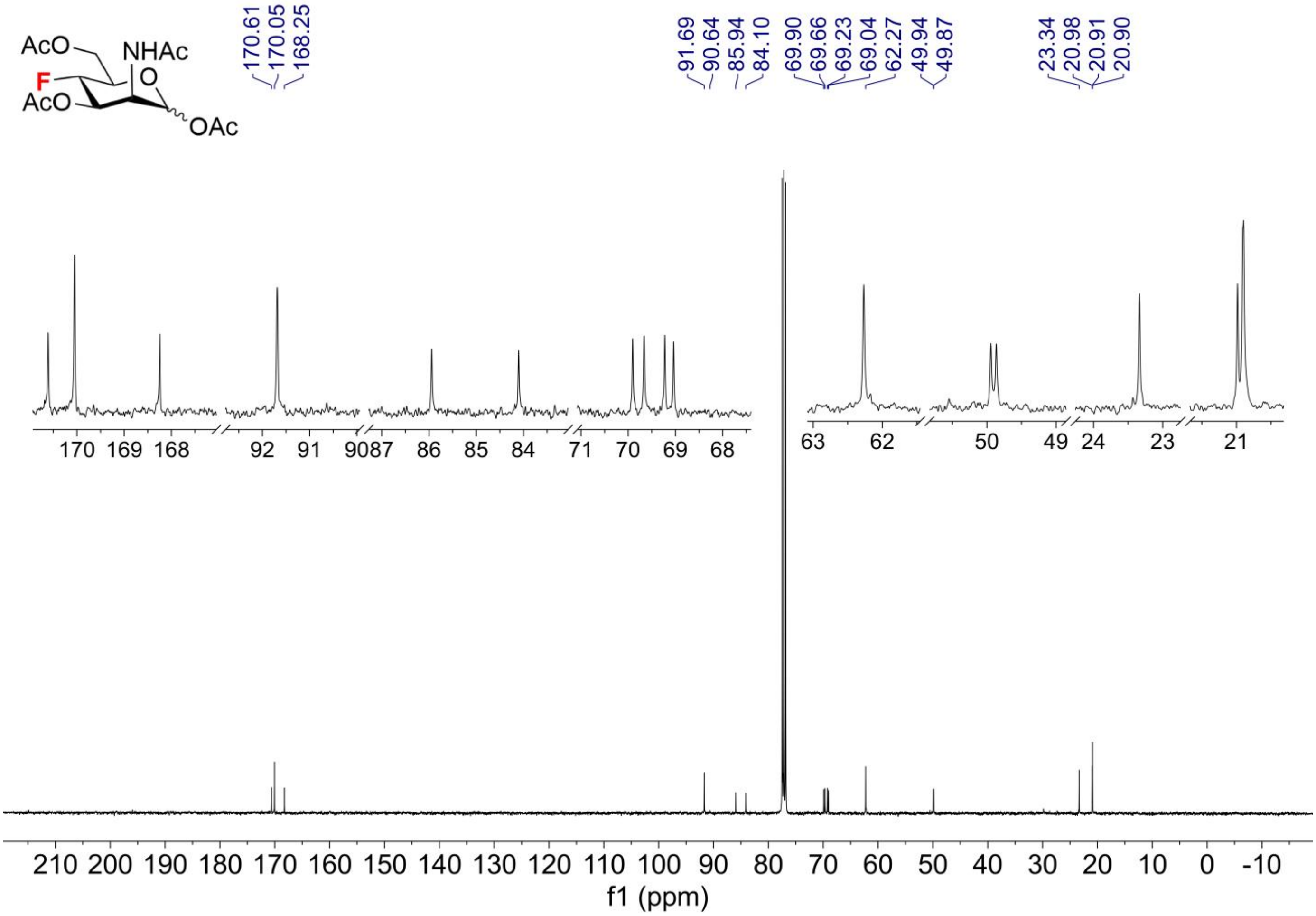

^{19}F NMR (376 MHz, CDCl$_3$) 32

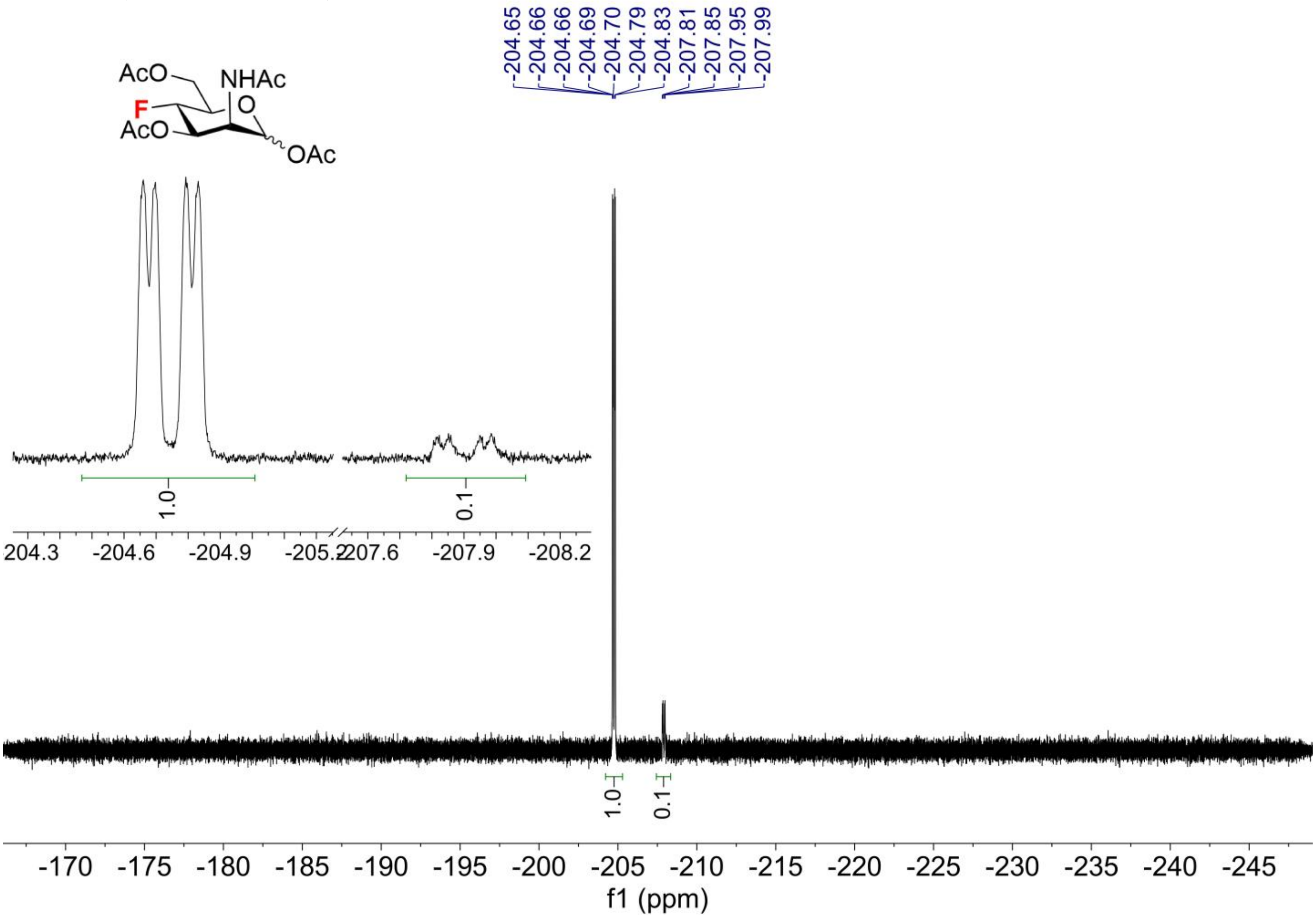

^{1}H-^{1}H COSY (400 MHz, CDCl$_3$) 32

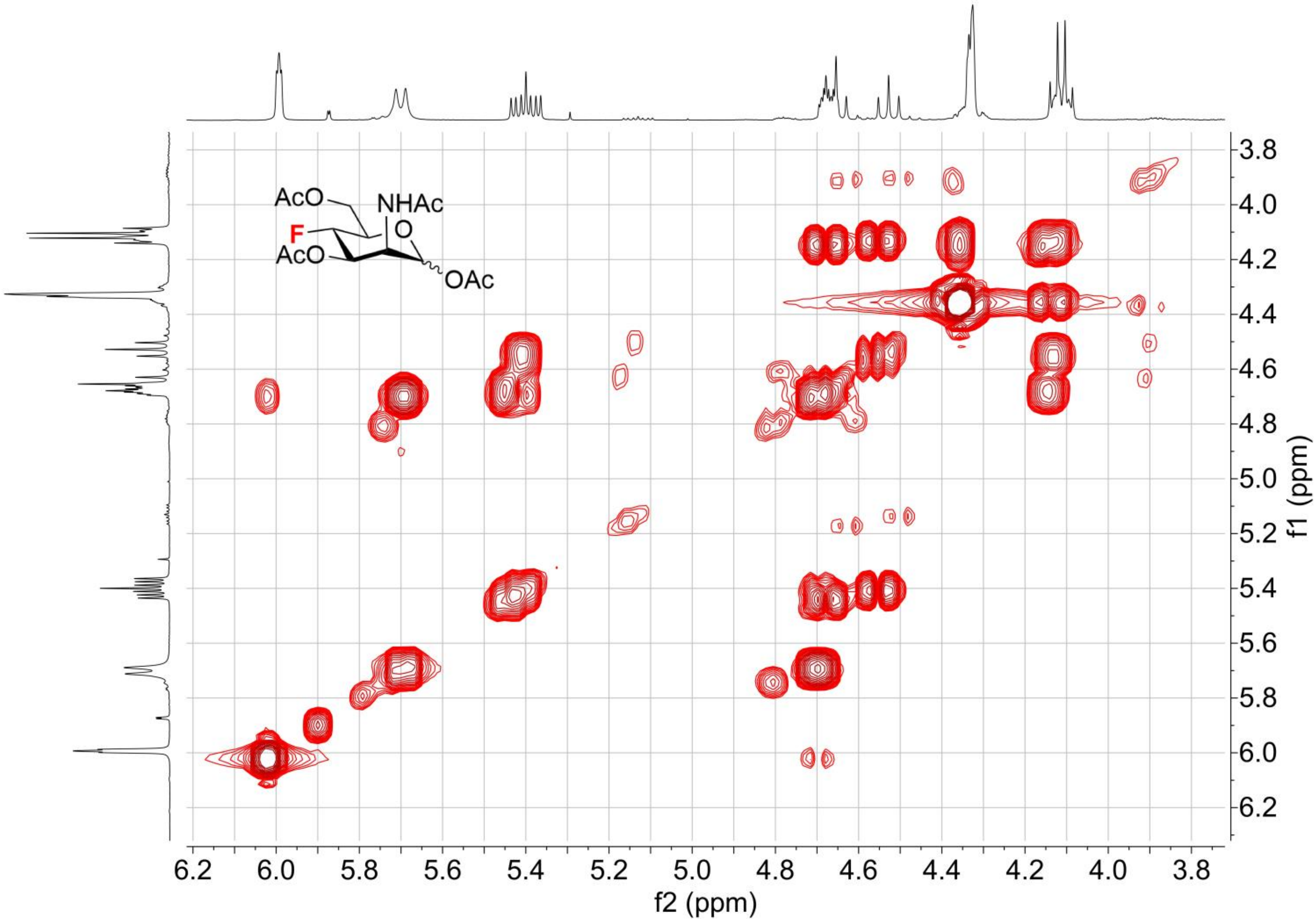

^{1}H-^{13}C HSQC (^{1}H/^{13}C 400/101 MHz, $CDCl_3$) 32

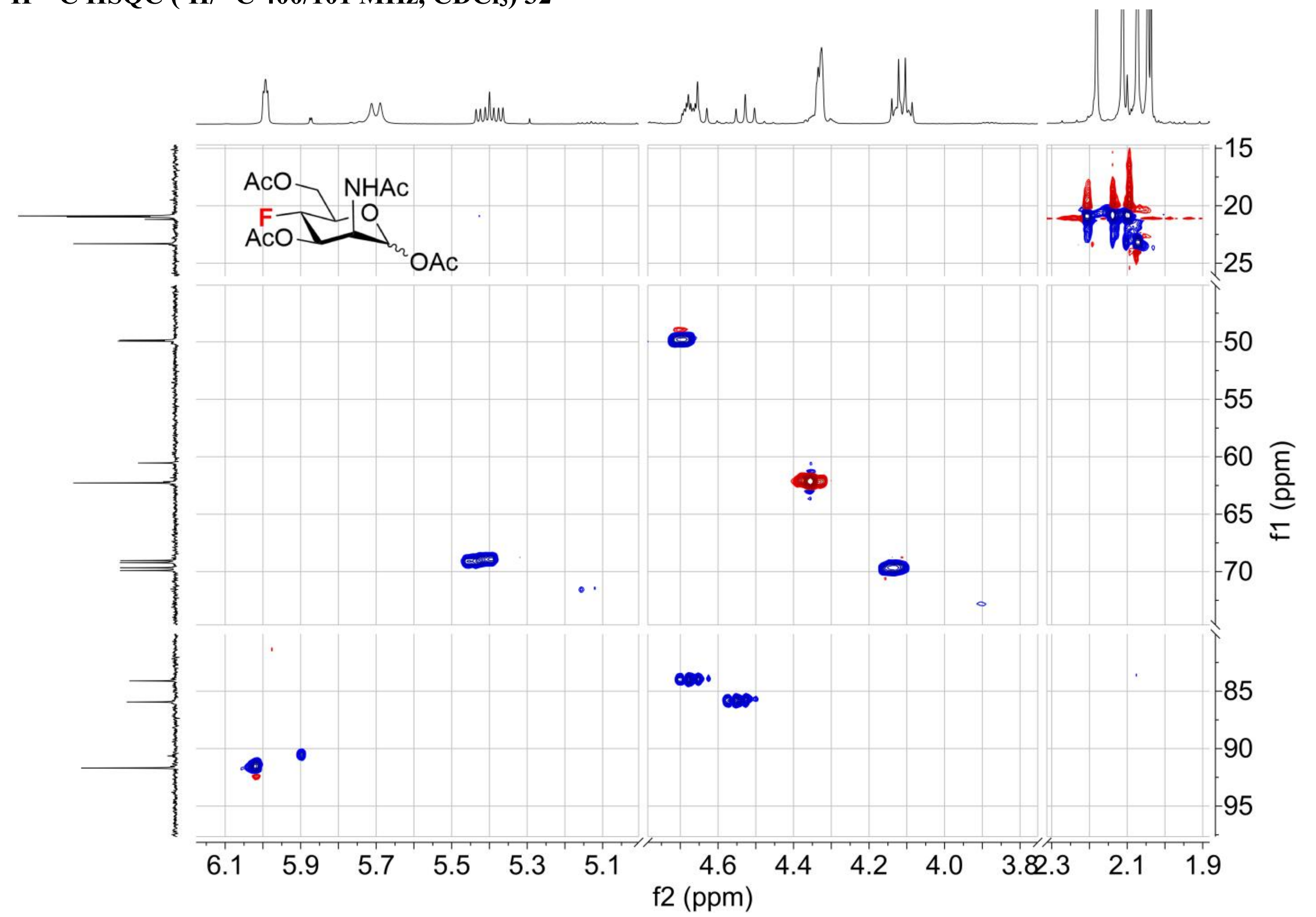

^{1}H-^{13}C HMBC (^{1}H/^{13}C 400/101 MHz, $CDCl_3$) 32

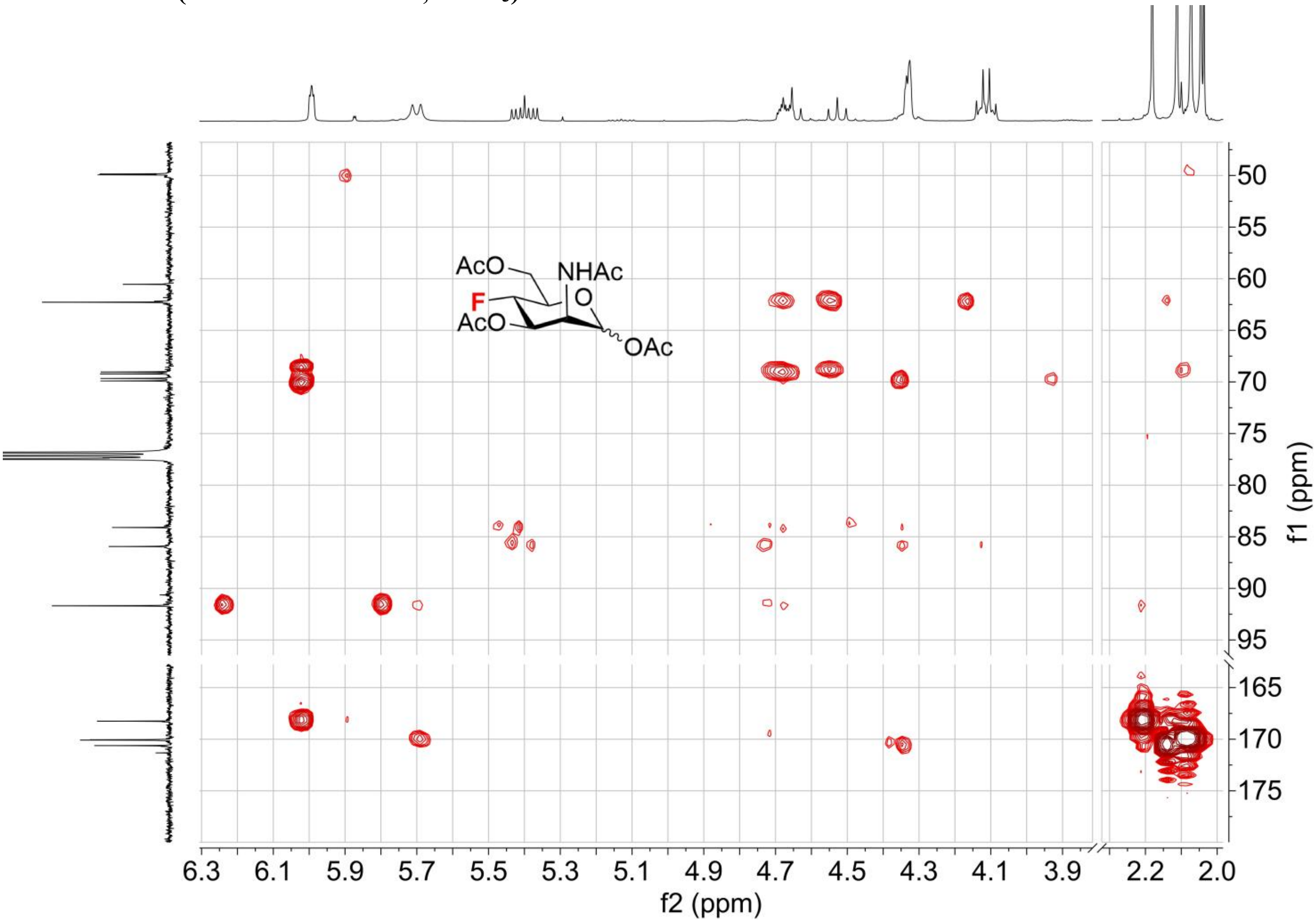

NMR COMPOUND 33

^{1}H NMR (400 MHz, CDCl3) 33

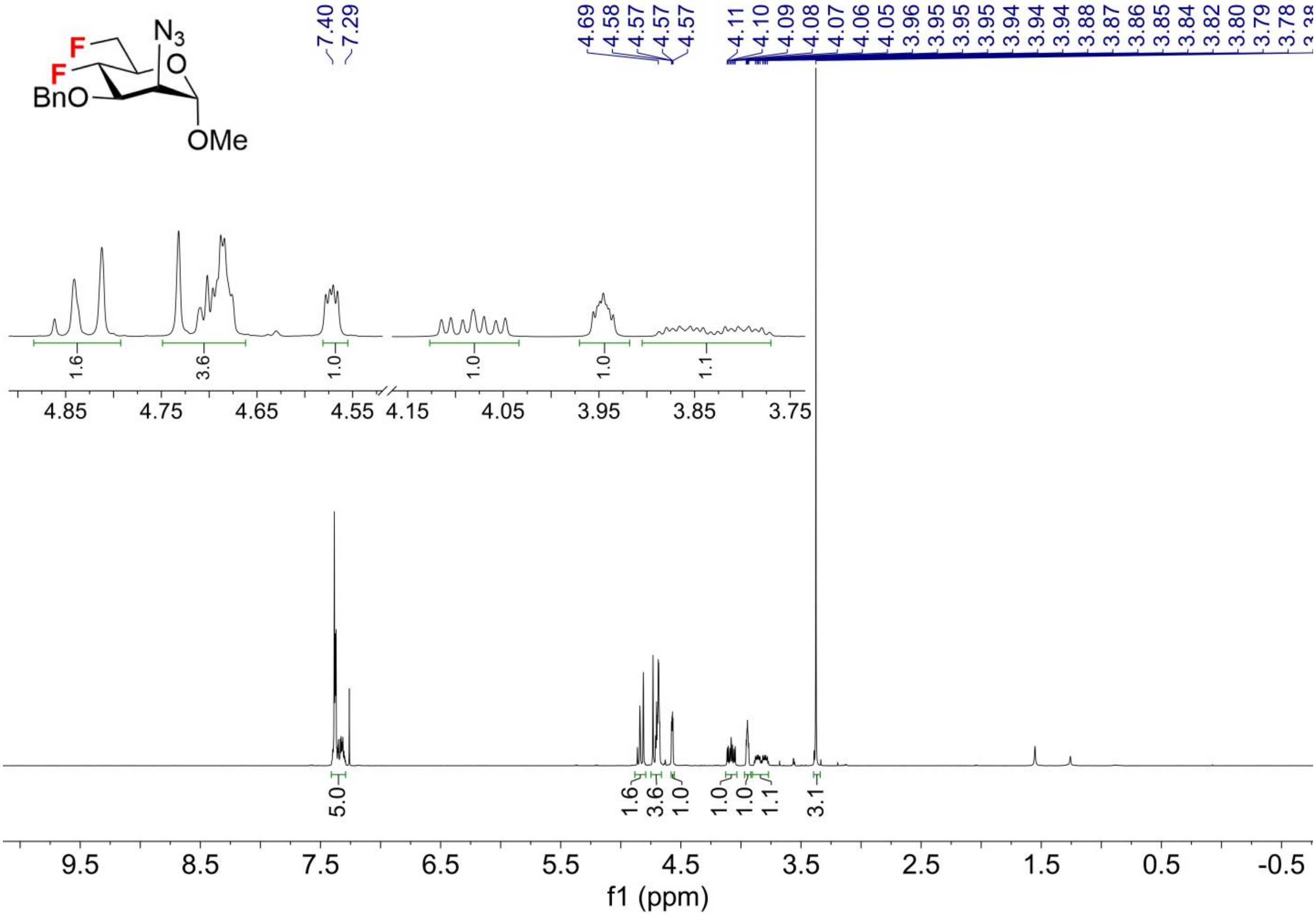

^{13}C{^{1}H} NMR (101 MHz, CDCl3) 33

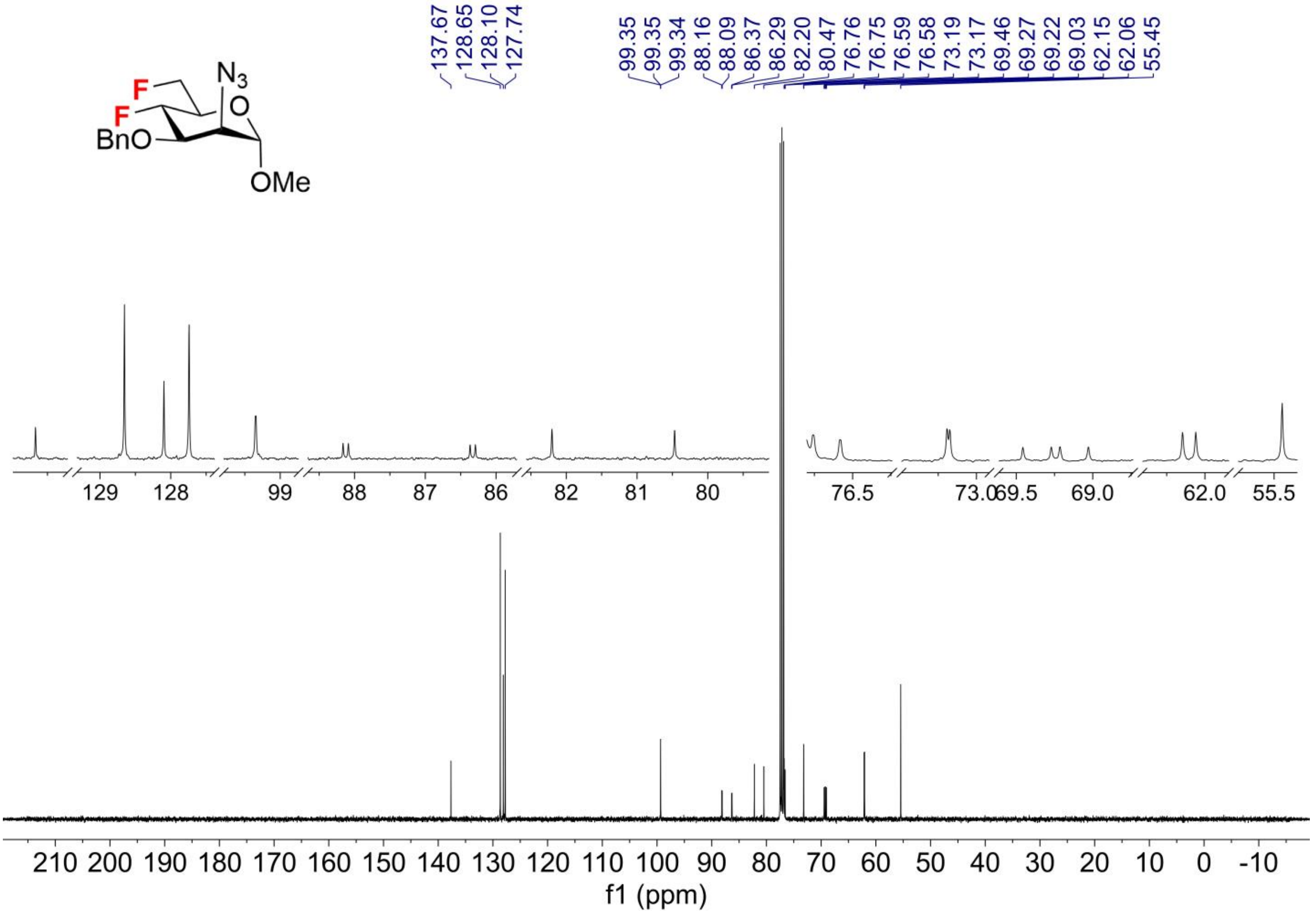

^{19}F NMR (376 MHz, CDCl$_3$) 33

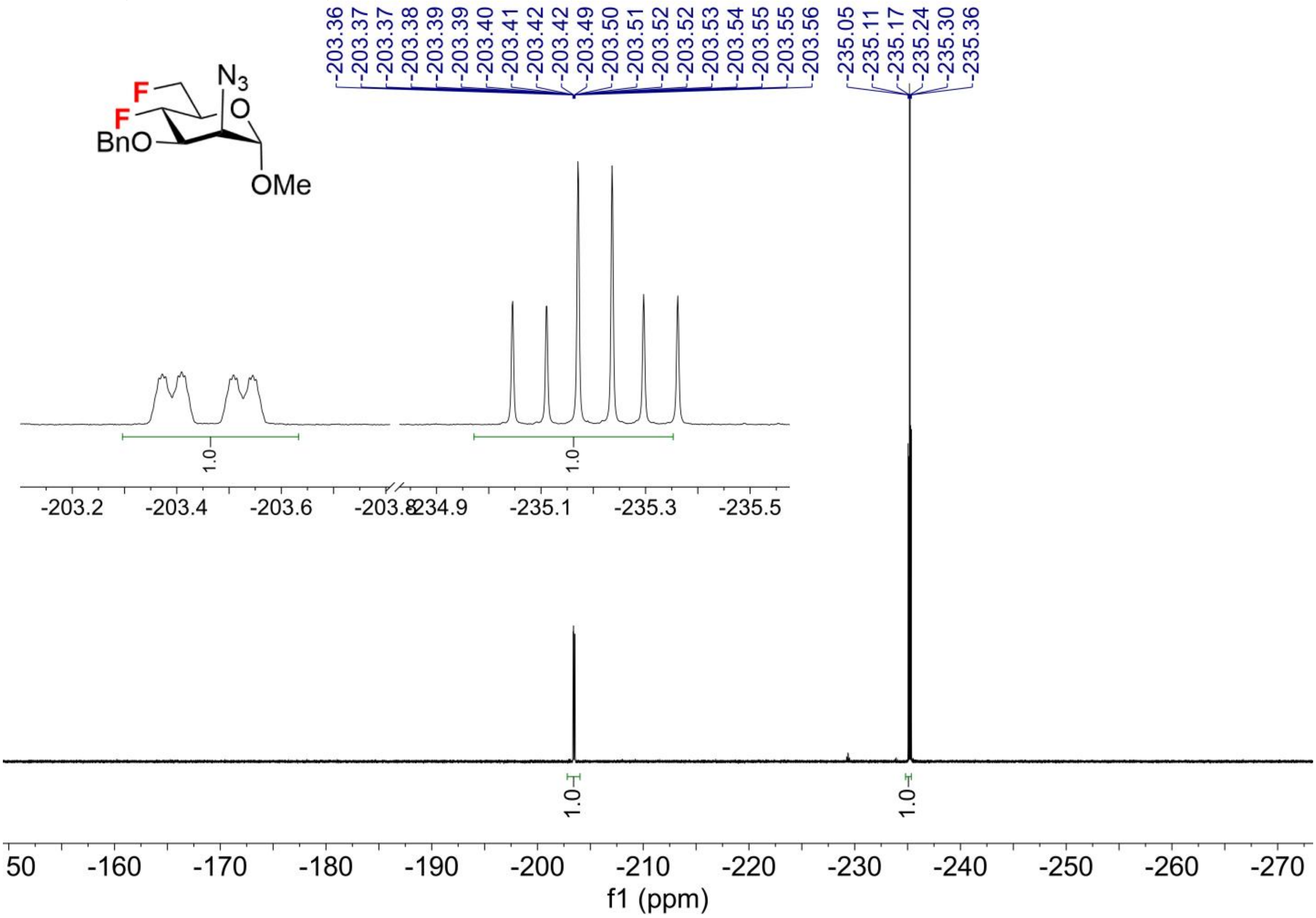

^{1}H-^{1}H COSY (400 MHz, CDCl$_3$) 33

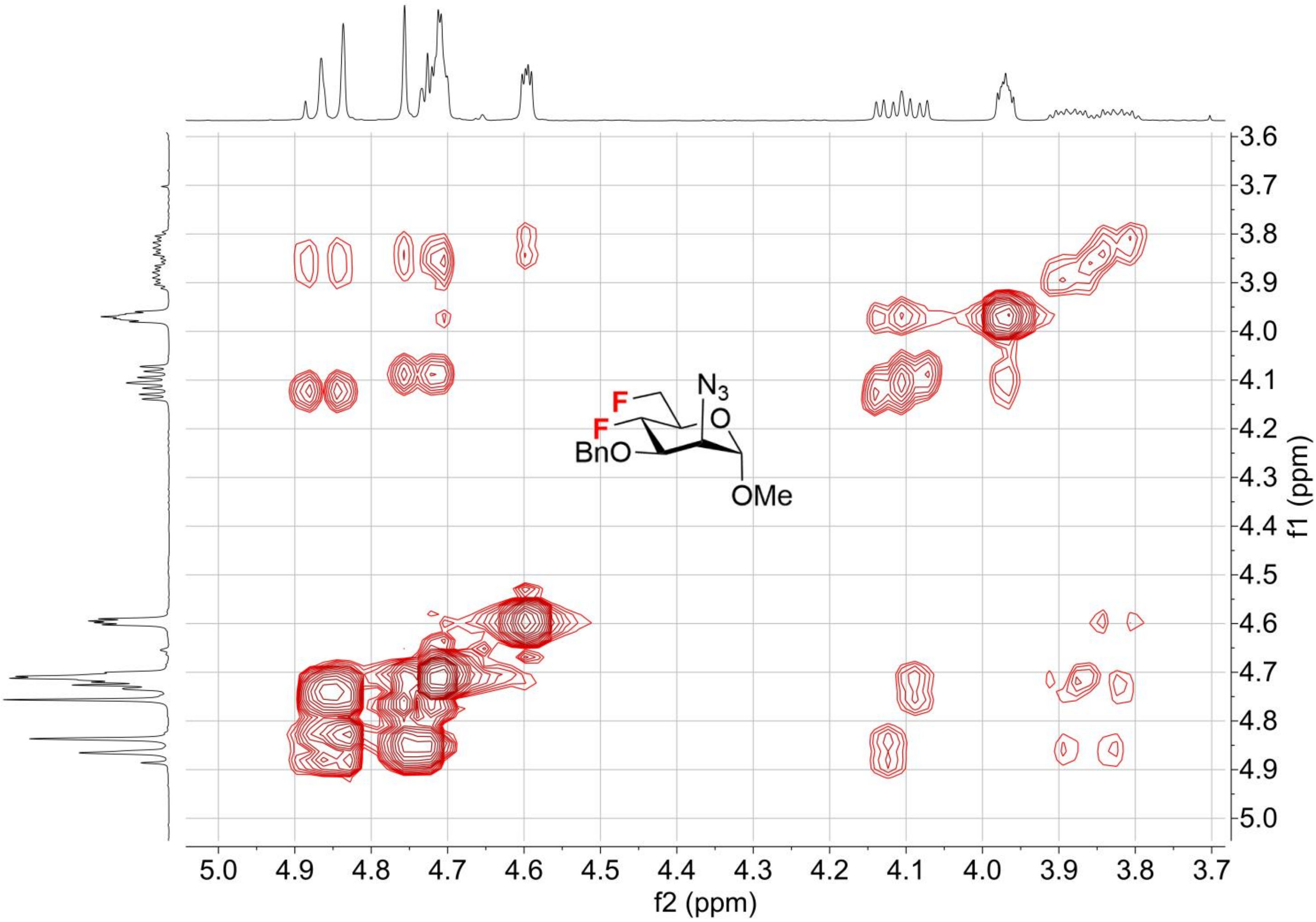

^{1}H-^{13}C HSQC (^{1}H/^{13}C 400/101 MHz, CDCl$_3$) 33

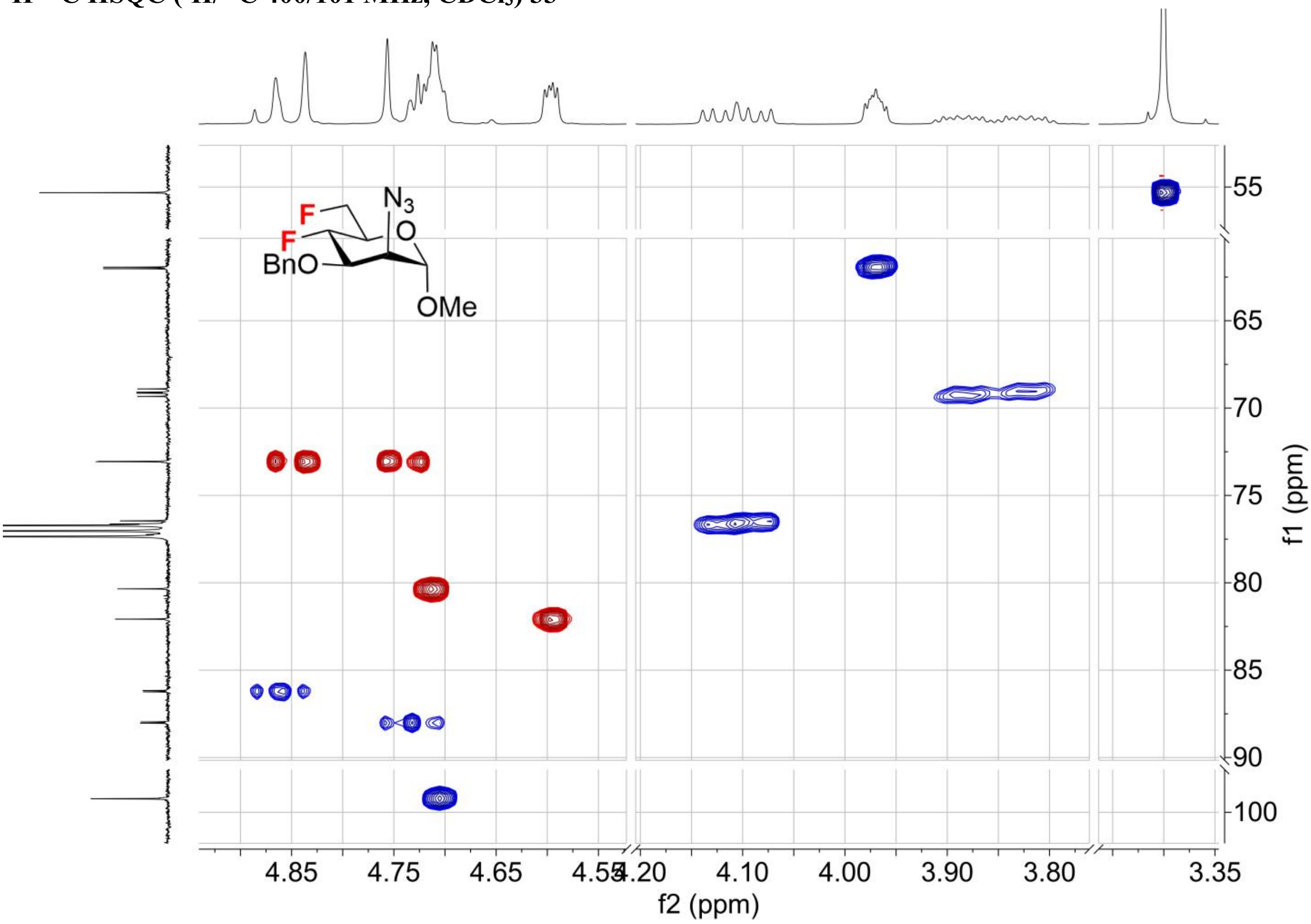

NMR COMPOUND 34

^{1}H NMR (400 MHz, $CDCl_3$) 34

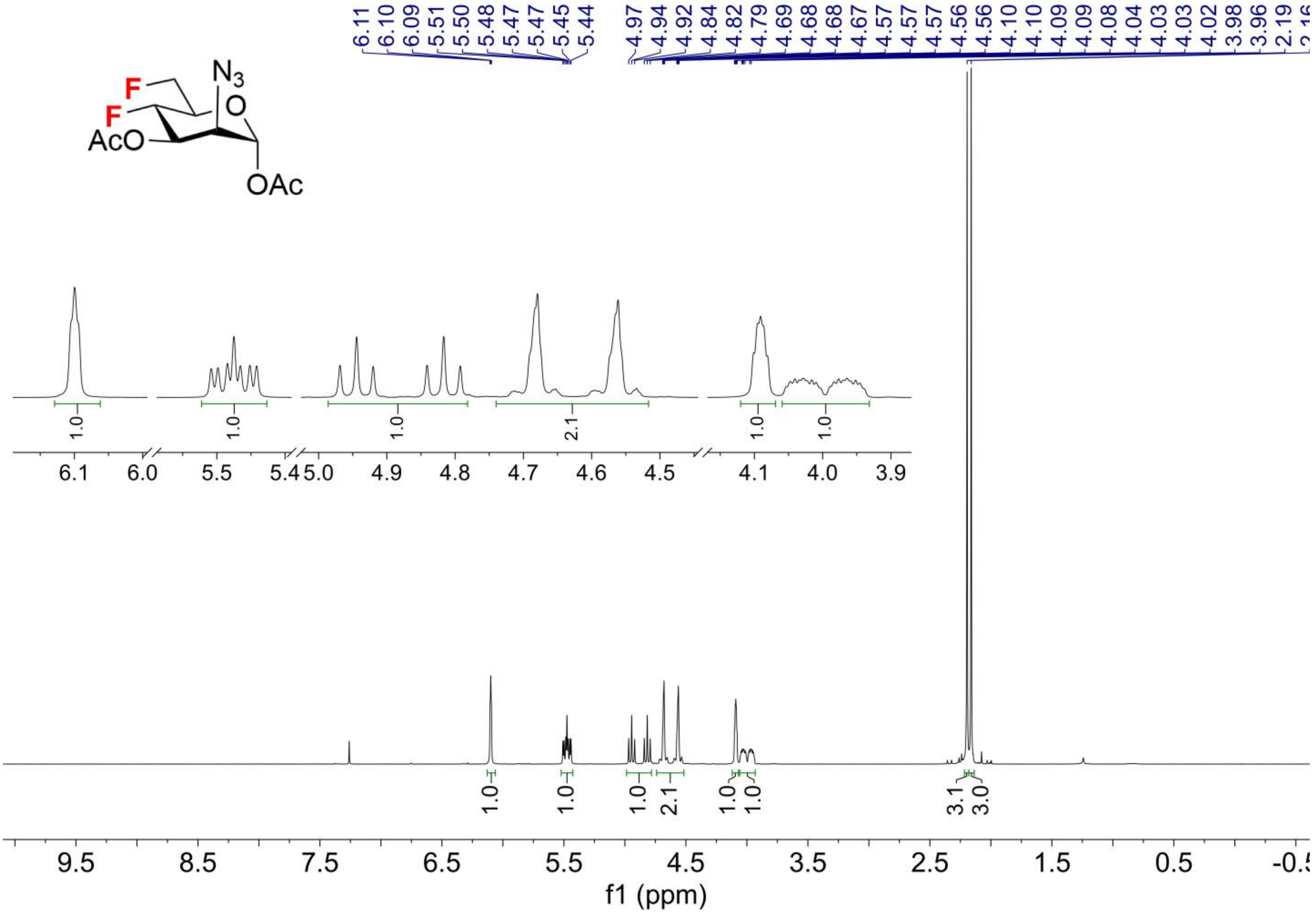

$^{13}C\{^1H\}$ NMR (101 MHz, $CDCl_3$) 34

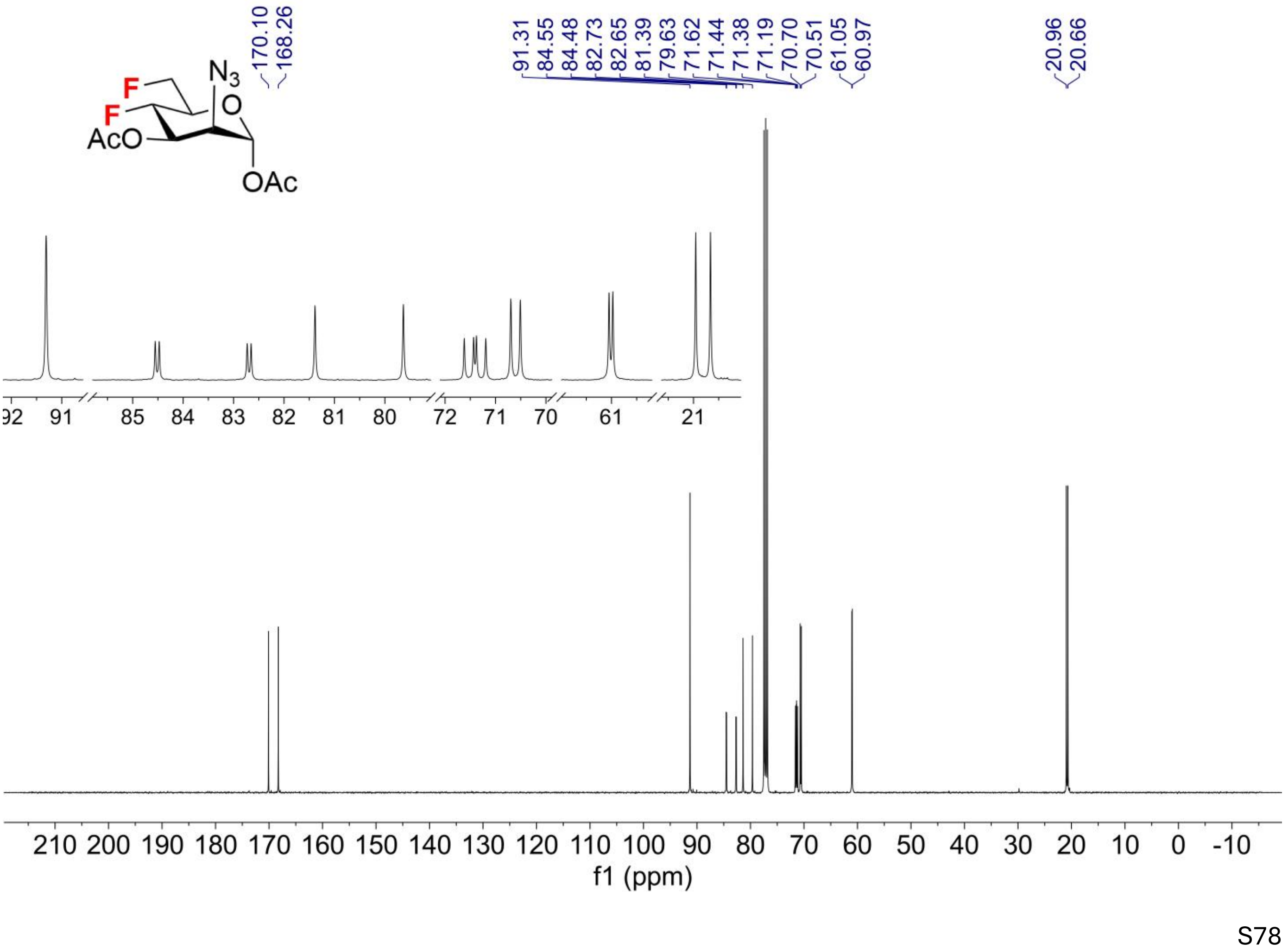

^{19}F NMR (376 MHz, CDCl$_3$) 34

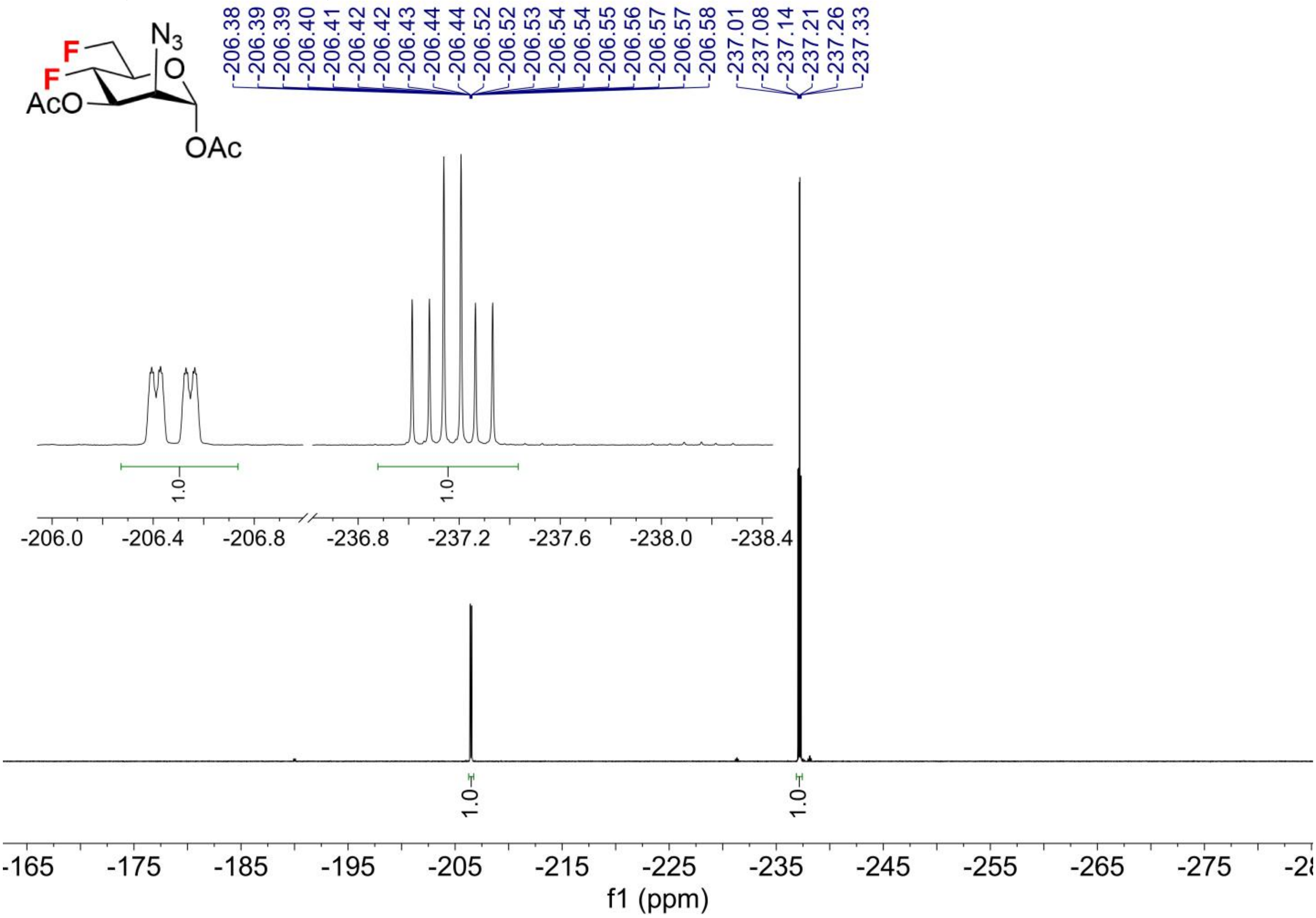

^{1}H-^{1}H COSY (400 MHz, CDCl$_3$) 34

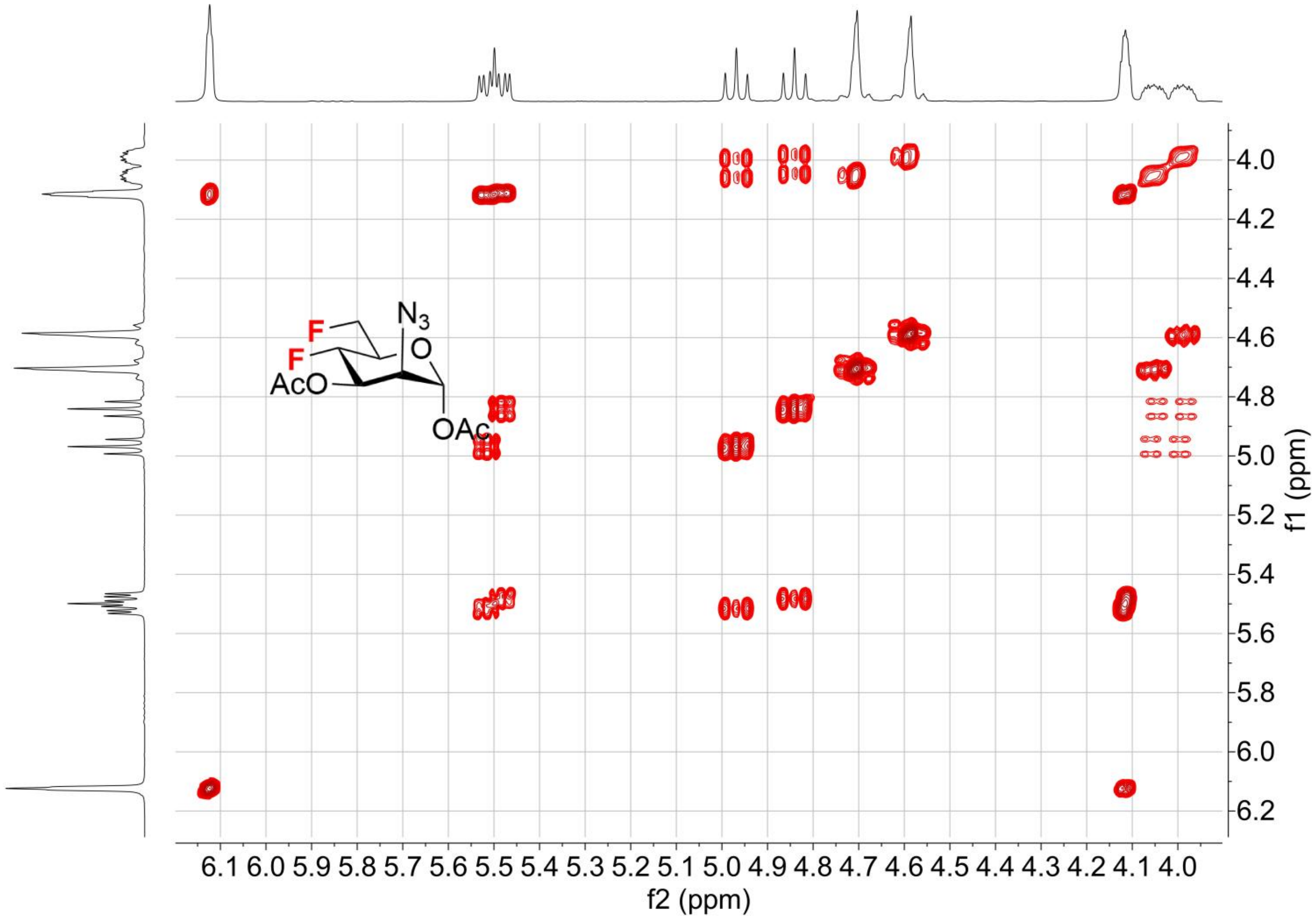

^{1}H-^{13}C HSQC (^{1}H/^{13}C 400/101 MHz, CDCl$_3$) 34

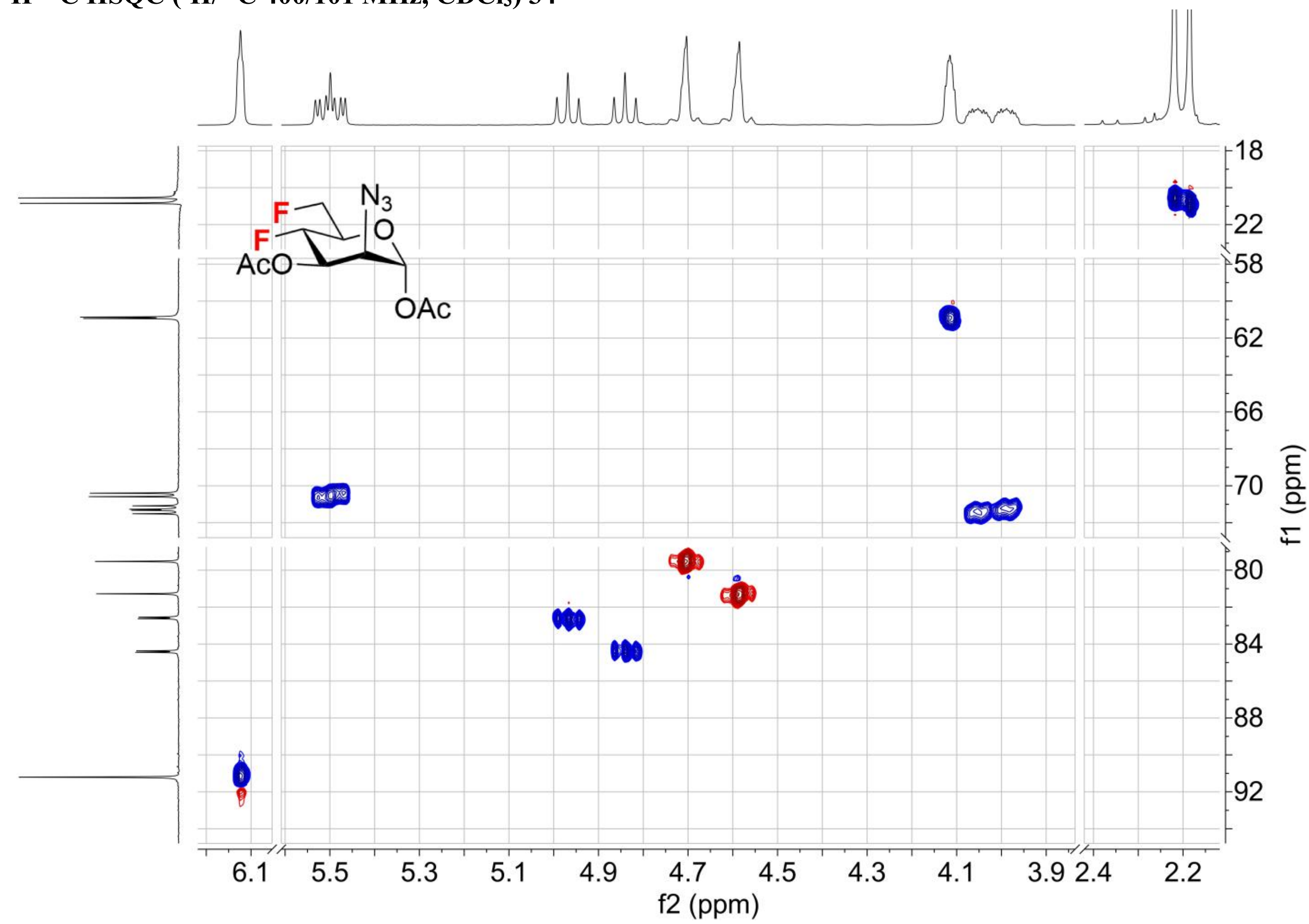

^{1}H-^{13}C HMBC (^{1}H/^{13}C 400/101 MHz, CDCl$_3$) 34

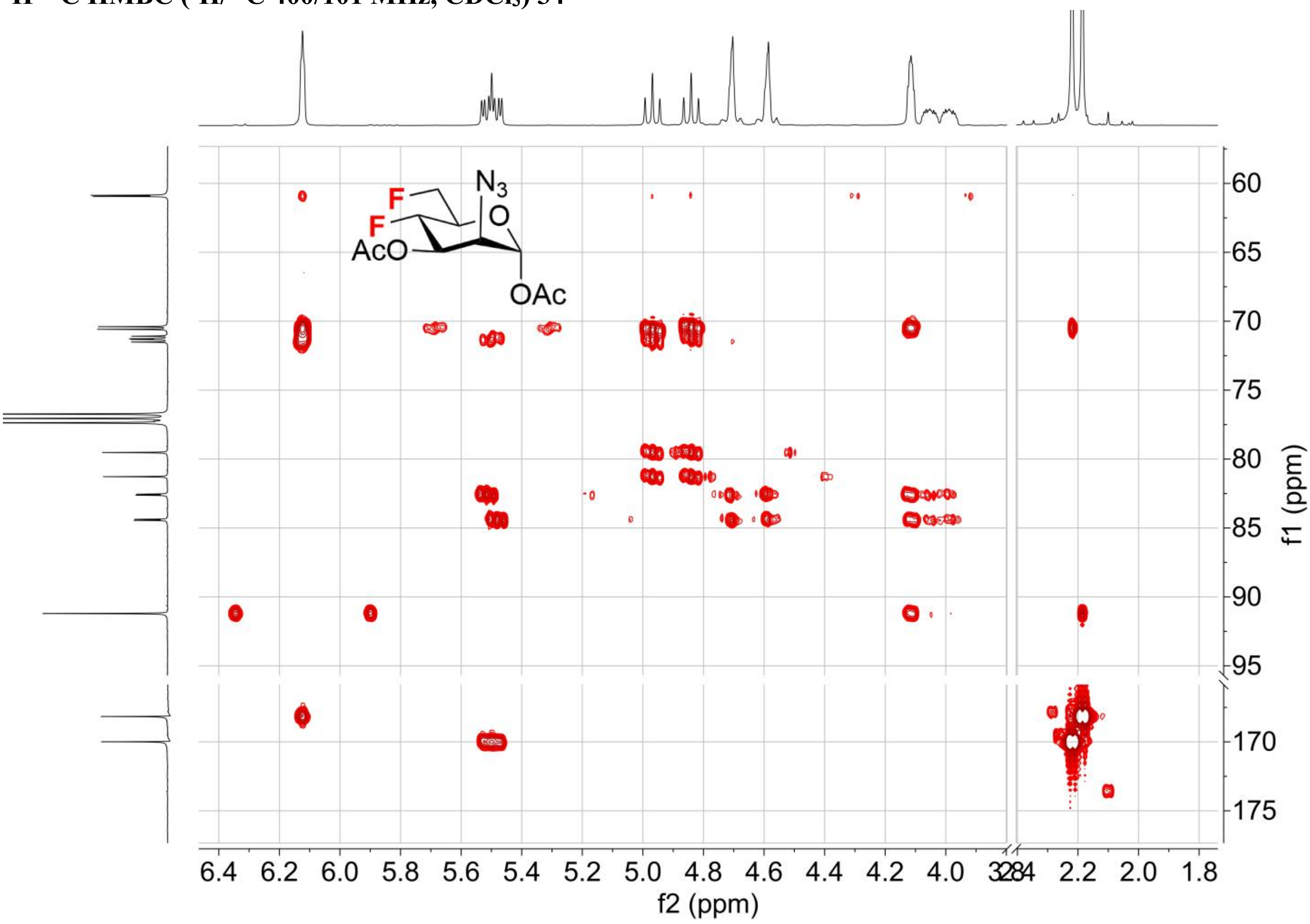

NMR COMPOUND 36

^{1}H NMR (400 MHz, $CDCl_3$) 36

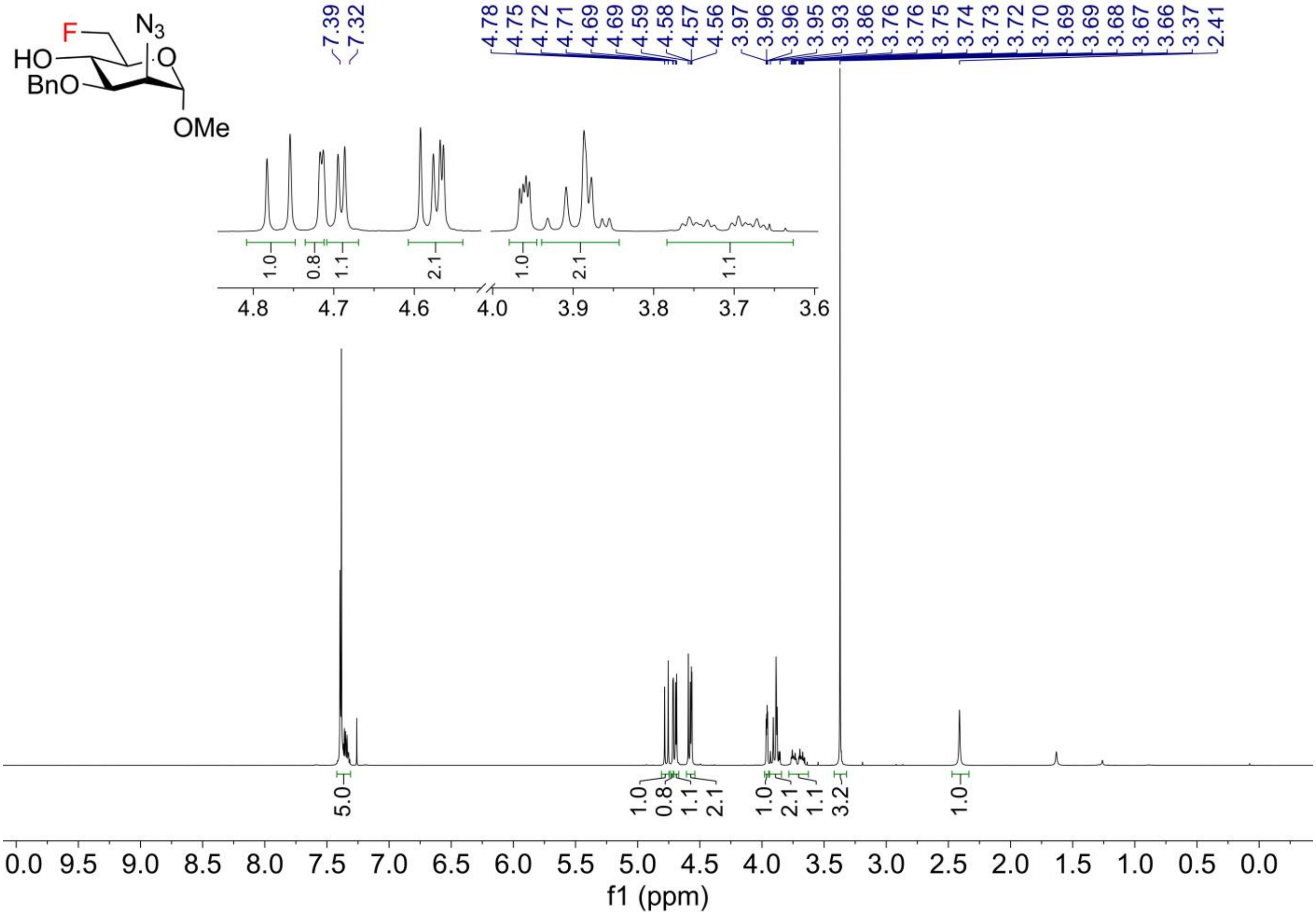

^{13}C{^{1}H} NMR (101 MHz, $CDCl_3$) 36

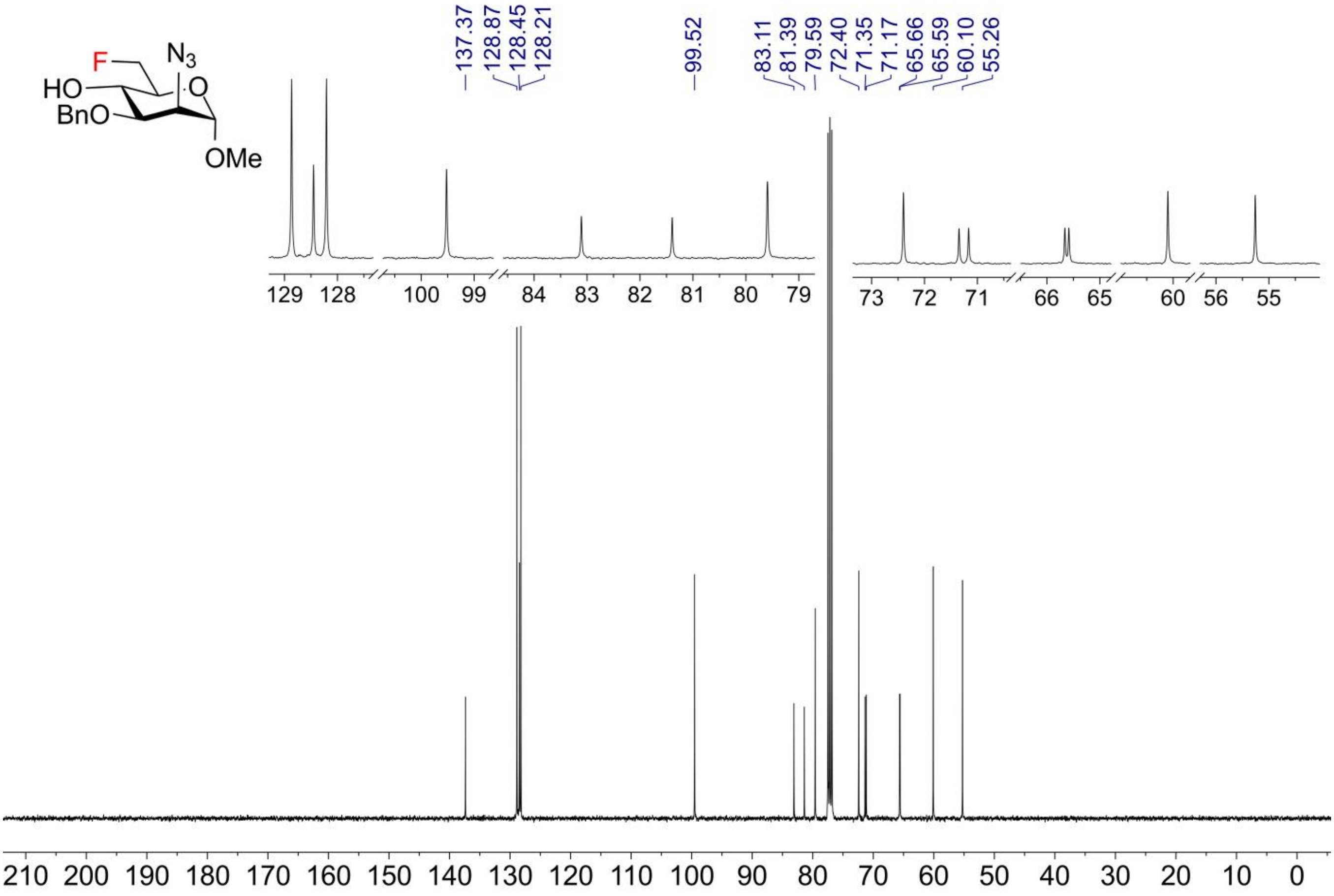

^{19}F NMR (376 MHz, CDCl$_3$) 36

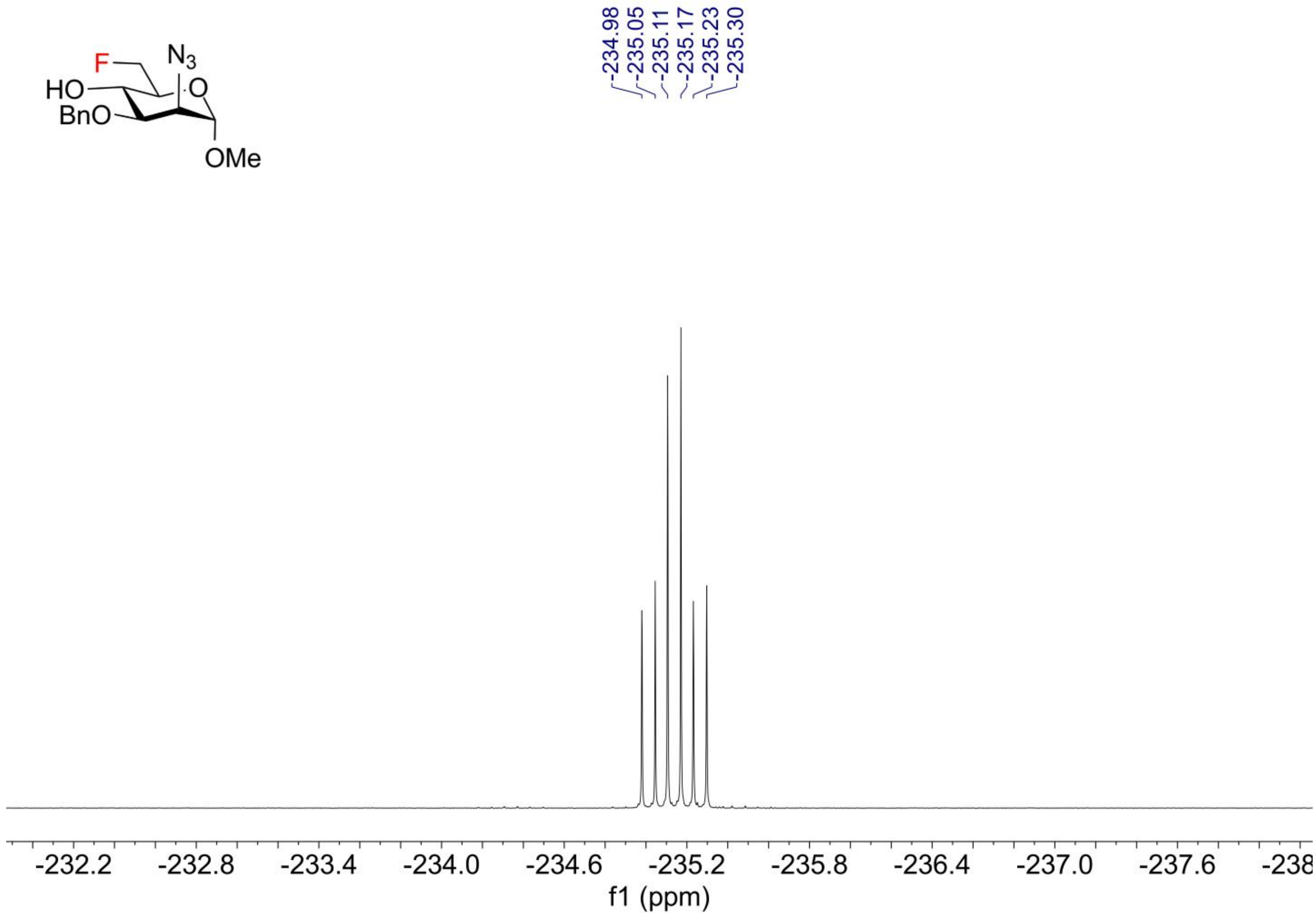

^{1}H-^{1}H COSY (400 MHz, CDCl$_3$) 36

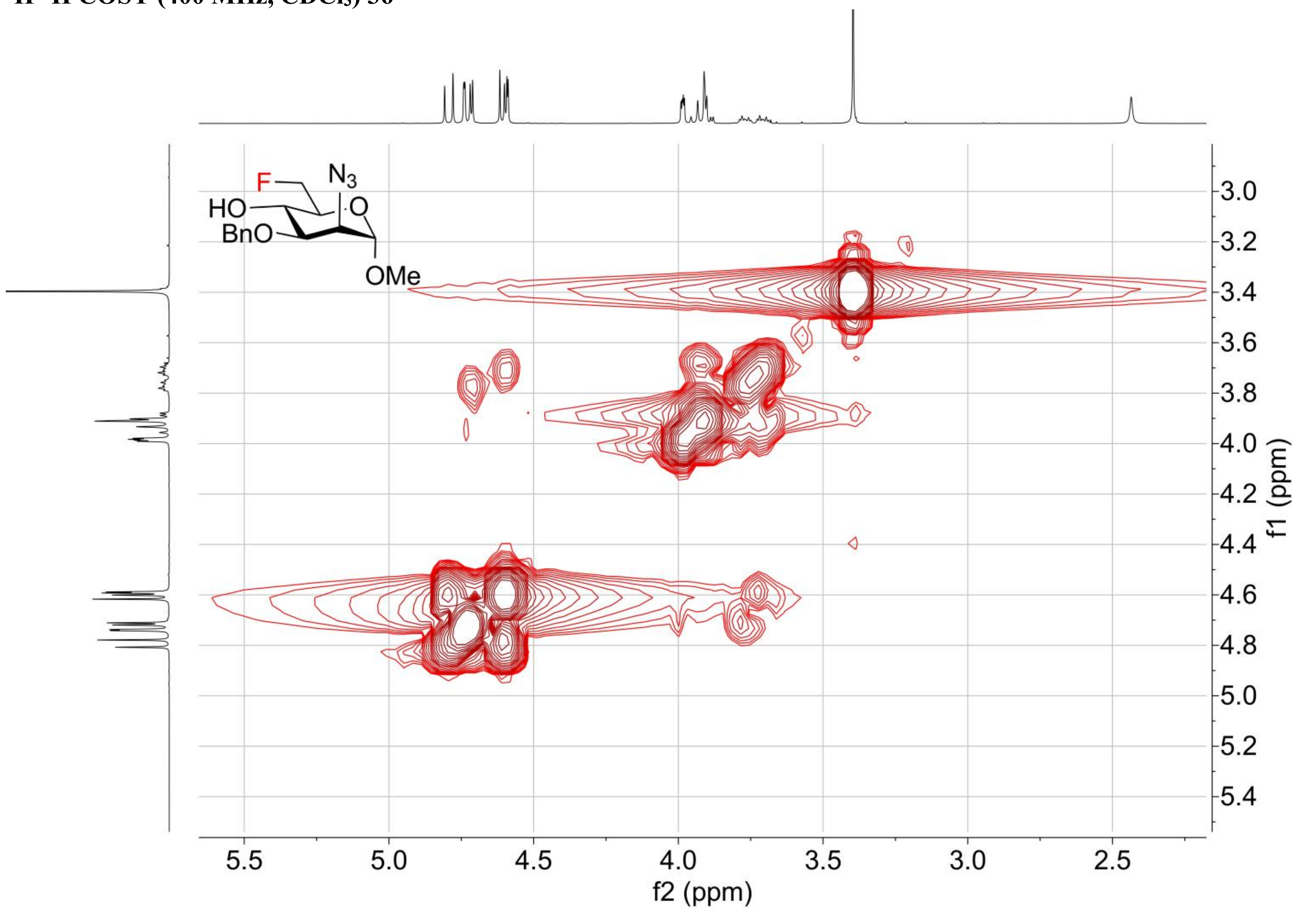

^{1}H-^{13}C HSQC (^{1}H/^{13}C 400/101 MHz, $CDCl_3$) 36

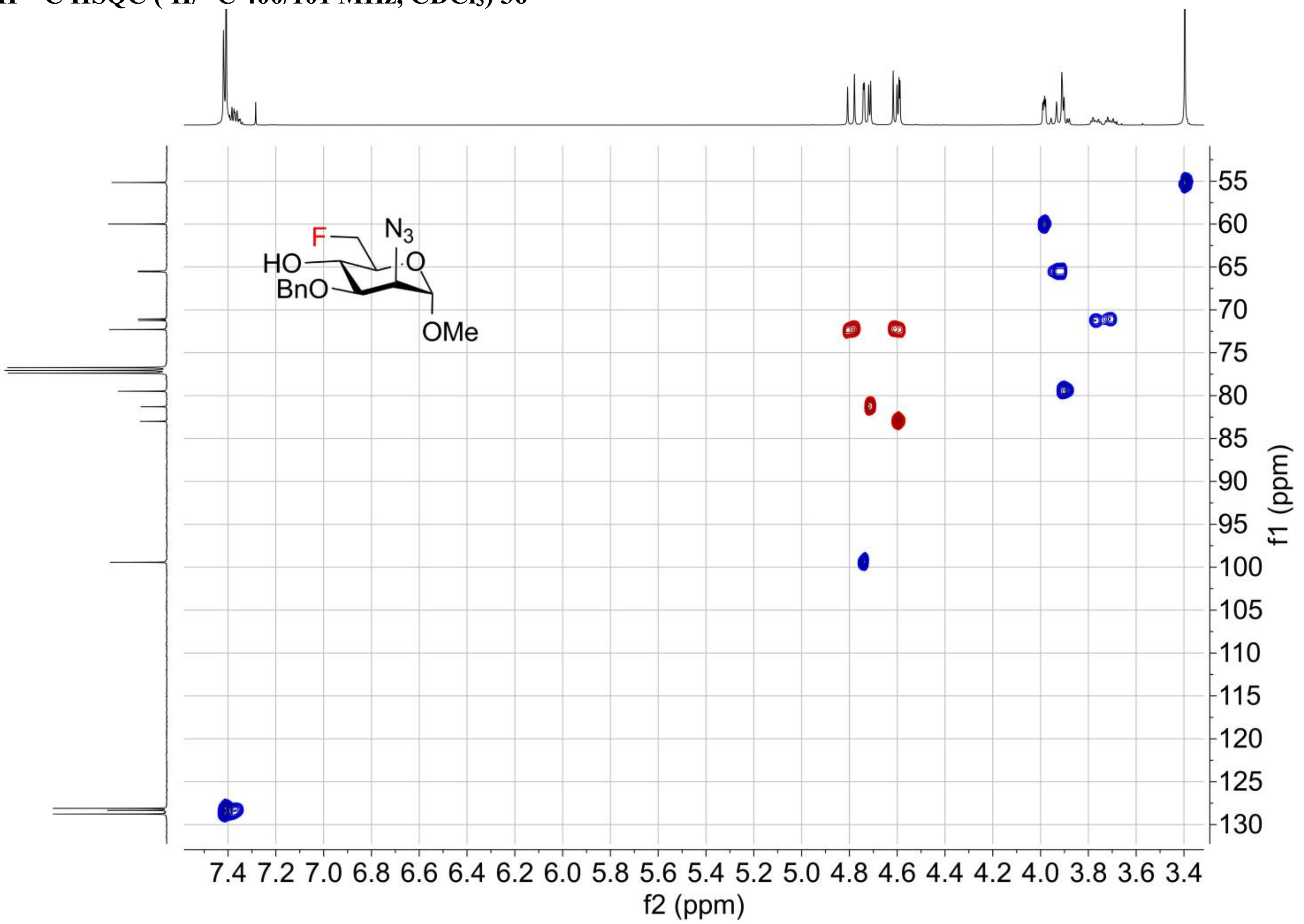

^{1}H-^{13}C HMBC (^{1}H/^{13}C 400/101 MHz, $CDCl_3$) 36

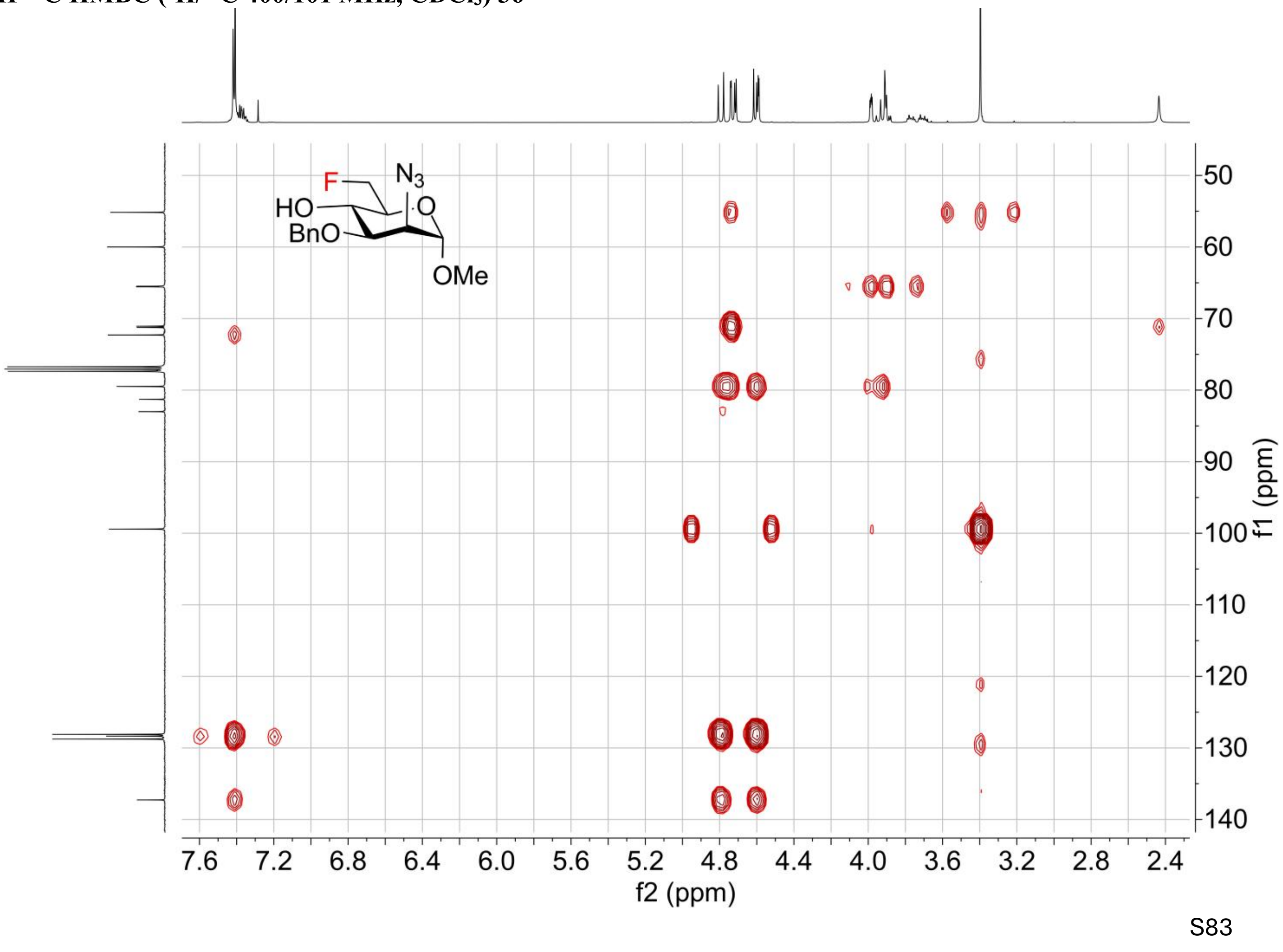

NMR COMPOUND 37

^{1}H NMR (400 MHz, $CDCl_3$) 37

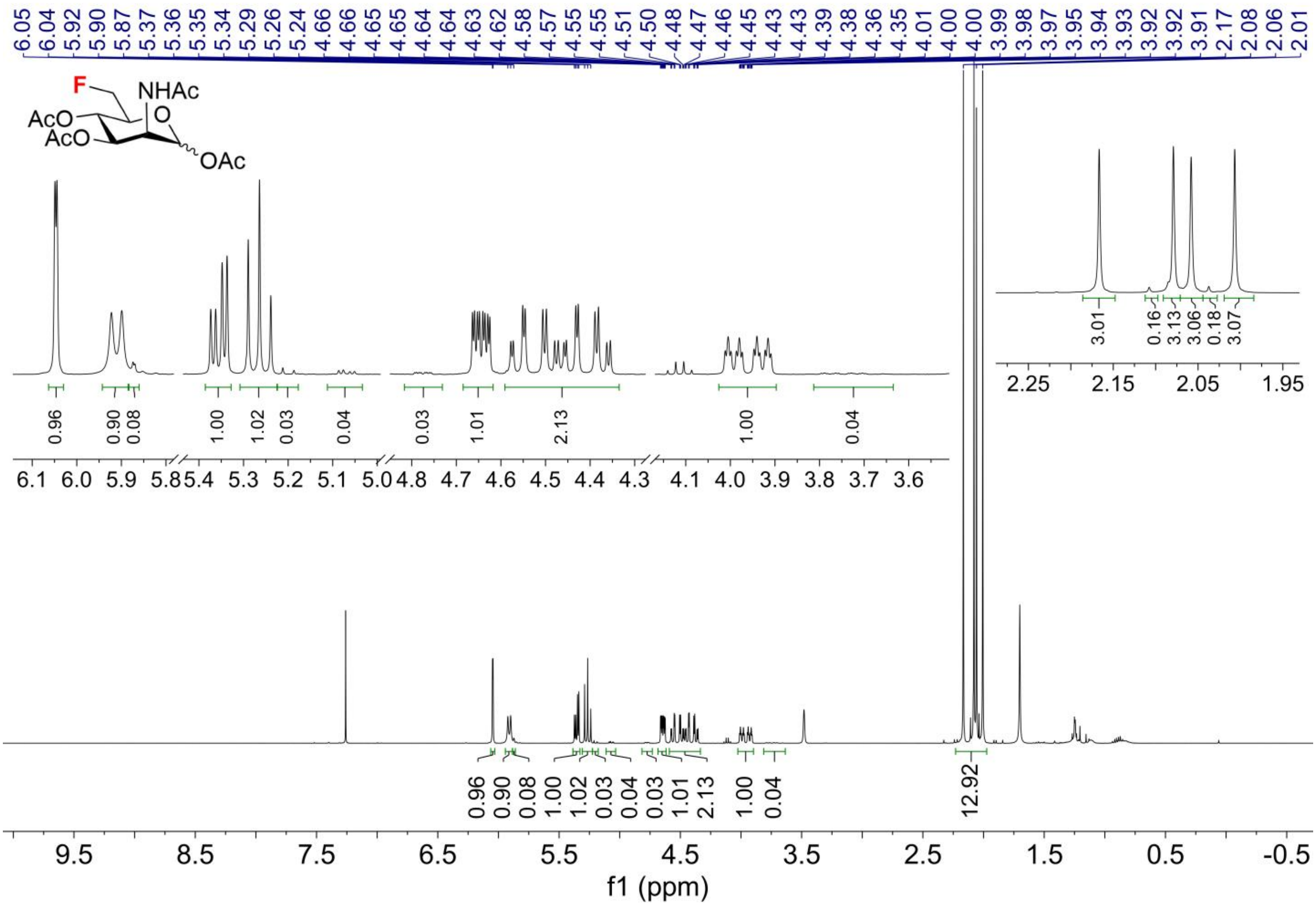

^{13}C{^{1}H} NMR (101 MHz, $CDCl_3$) 37

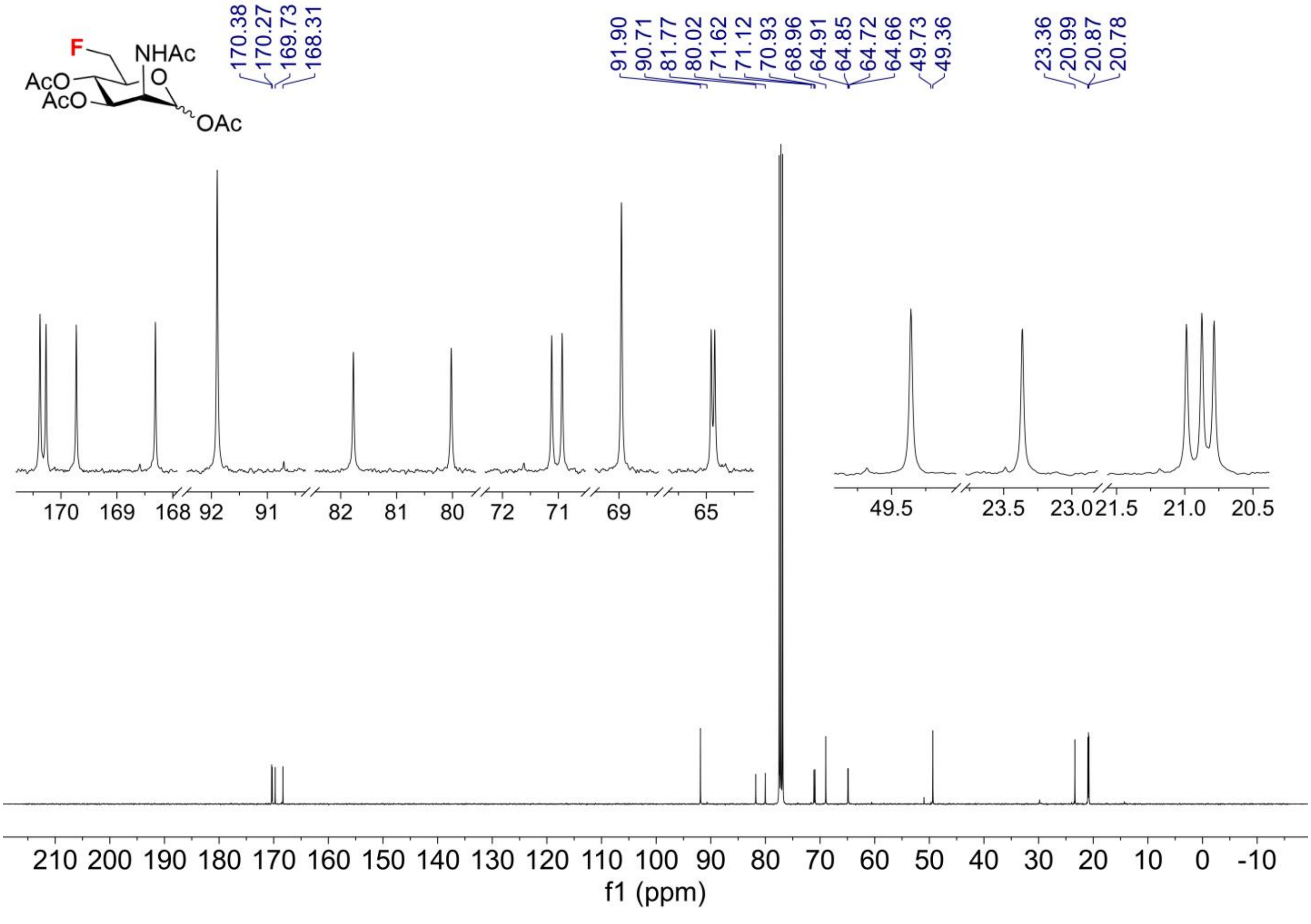

^{19}F NMR (376 MHz, CDCl$_3$) 37

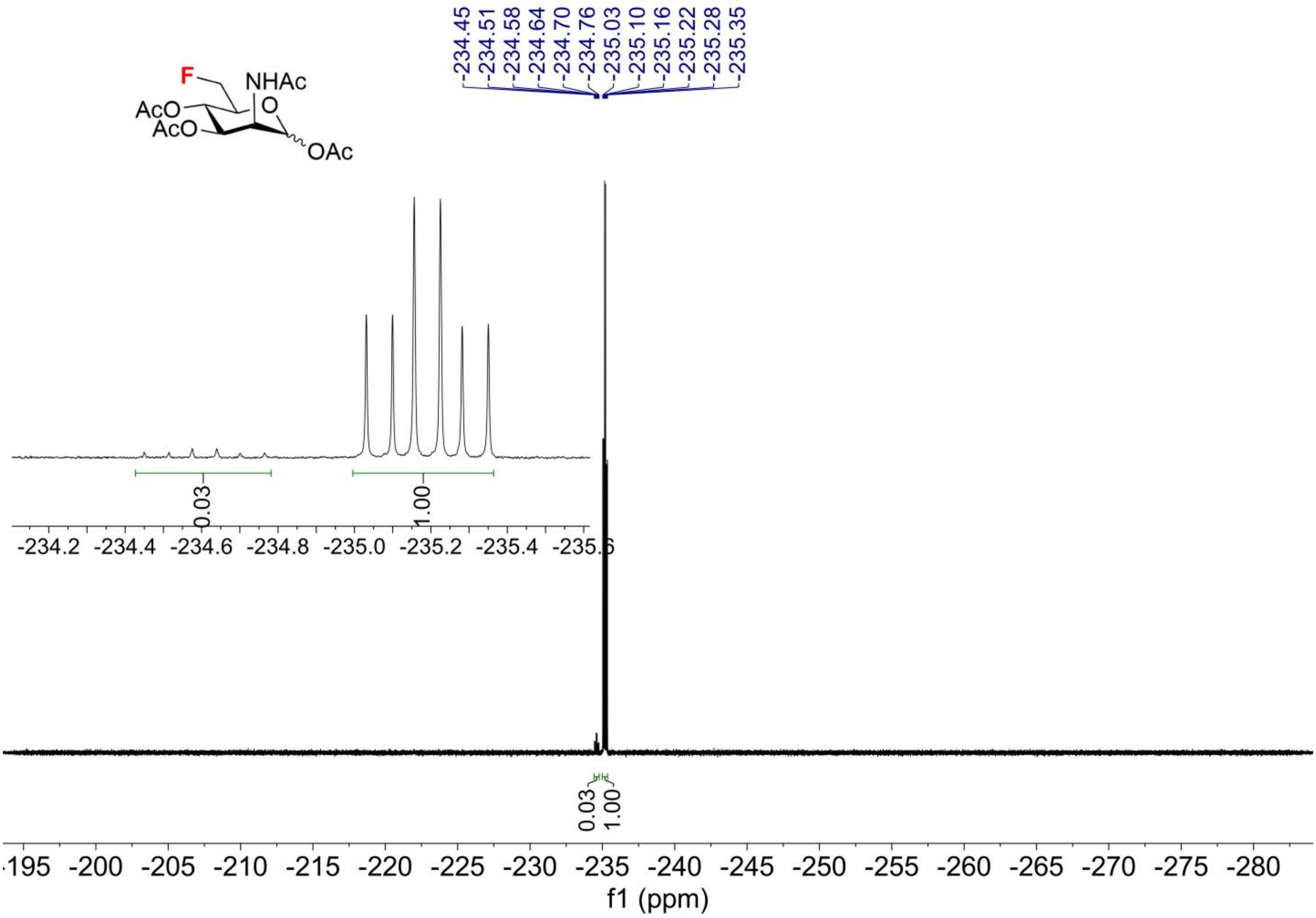

^{1}H-^{1}H COSY (400 MHz, CDCl$_3$) 37

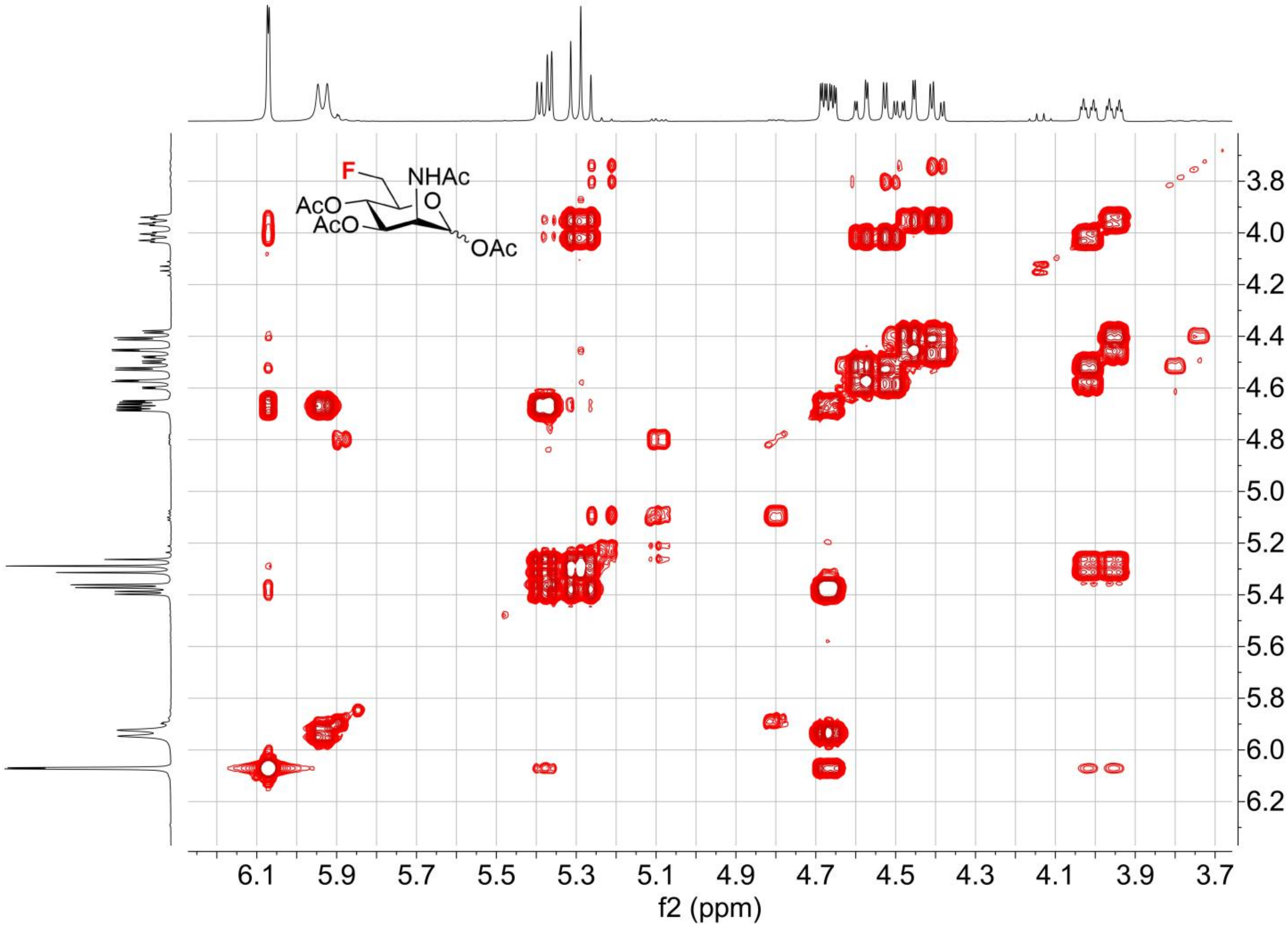

^{1}H-^{13}C HSQC (^{1}H/^{13}C 400/101 MHz, CDCl$_3$) 37

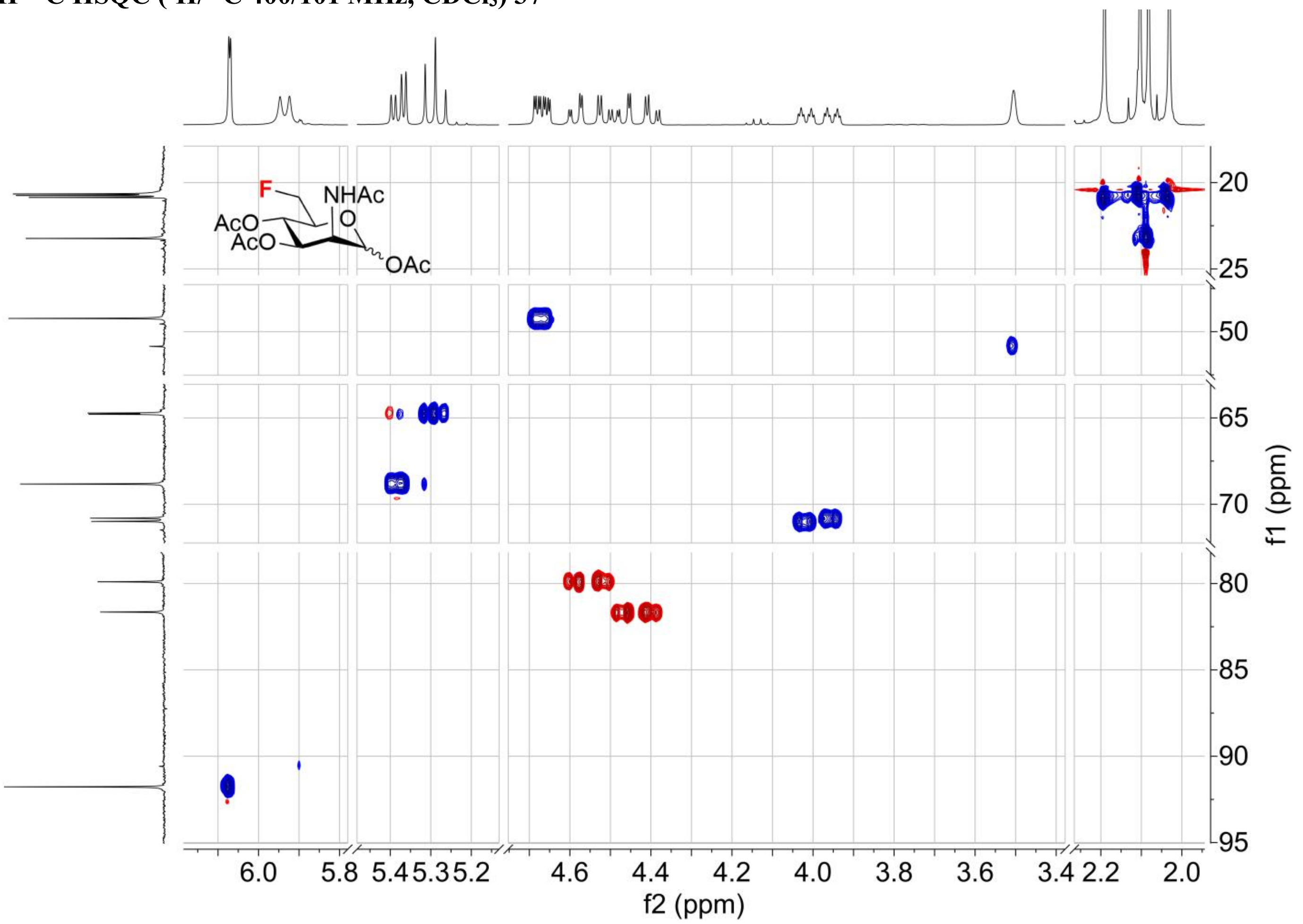

^{1}H-^{13}C HMBC (^{1}H/^{13}C 400/101 MHz, CDCl$_3$) 37

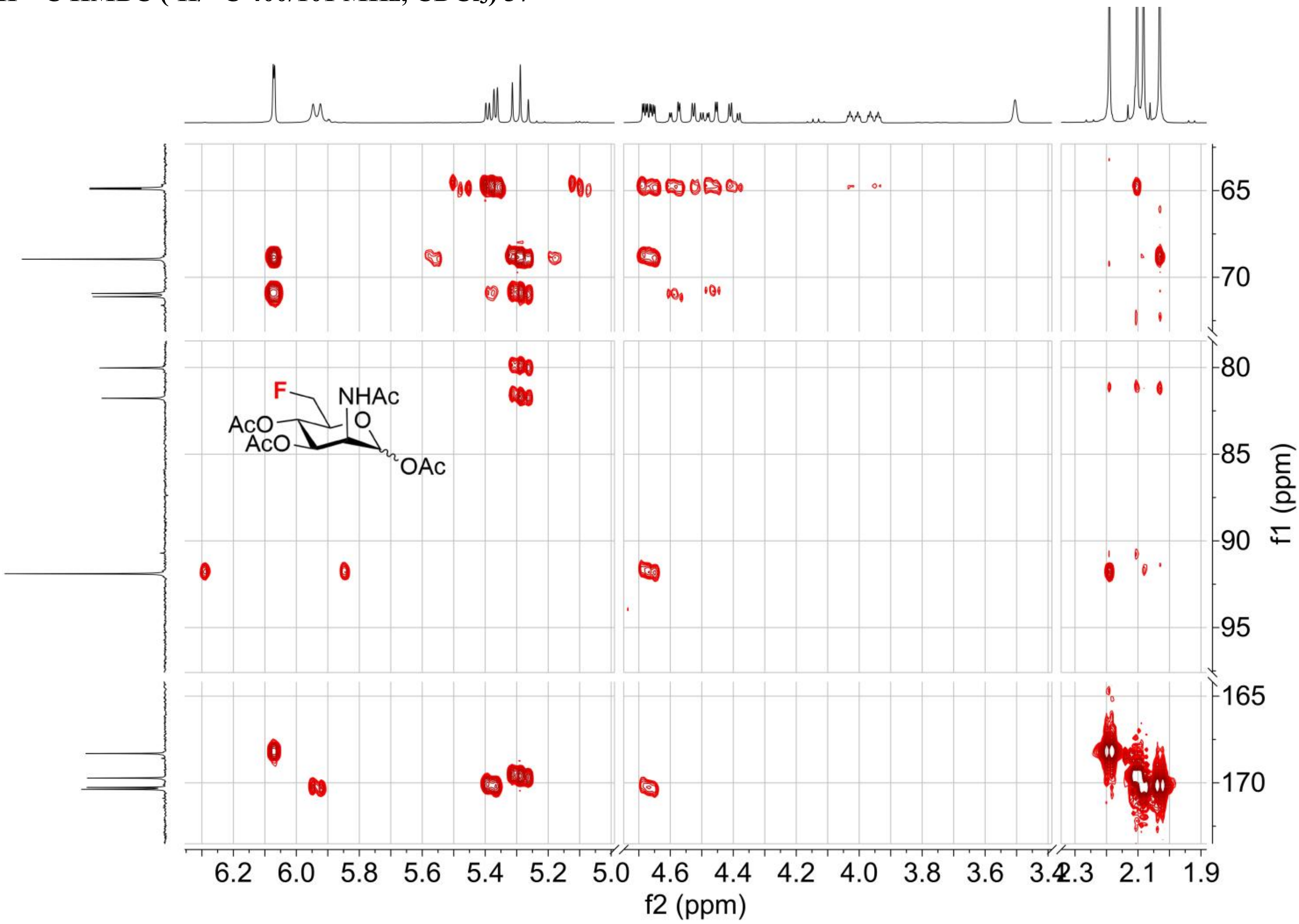

NMR COMPOUND 38

^{1}H NMR (400 MHz, $CDCl_3$) 38

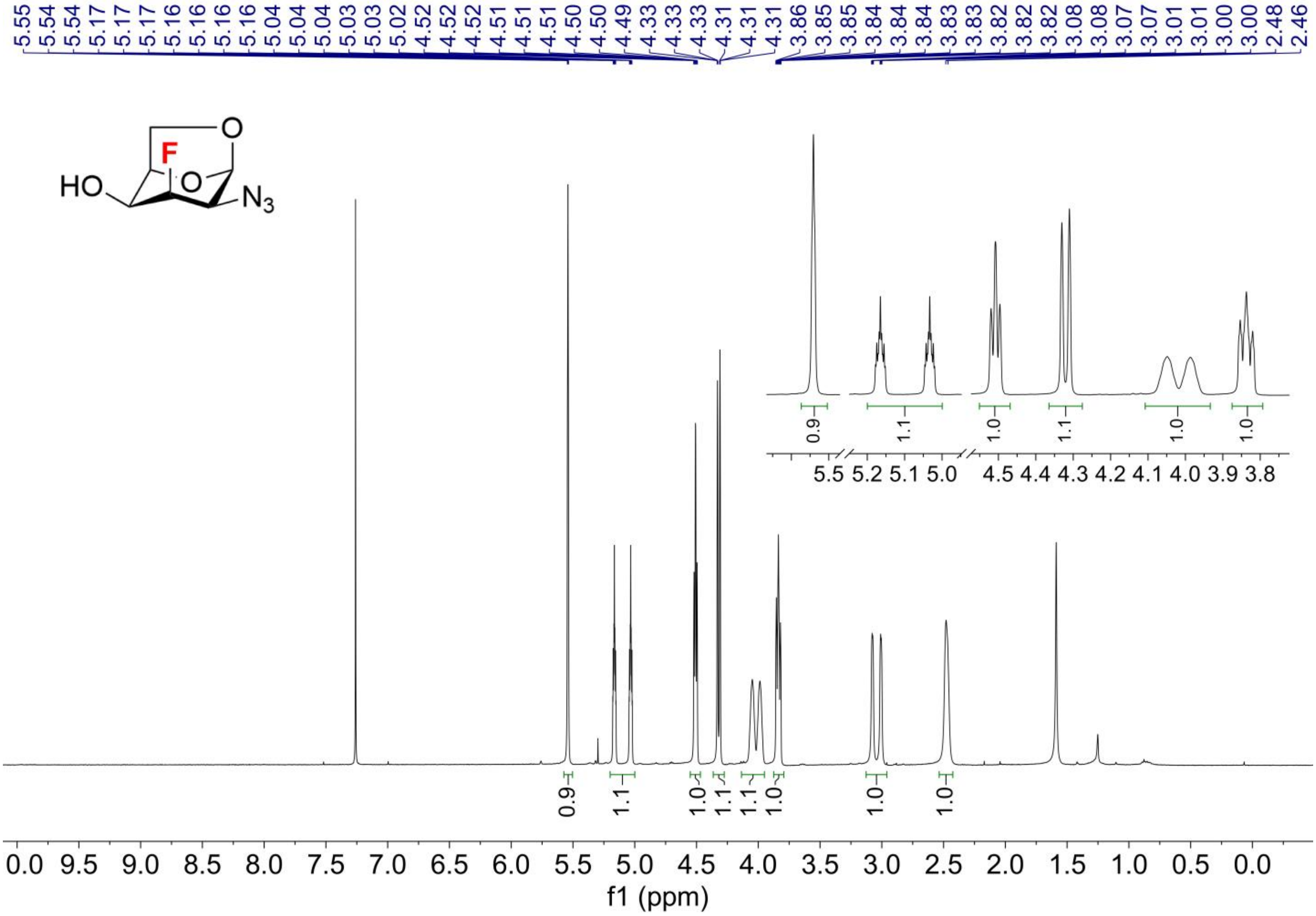

$^{13}C\{^{1}H\}$ NMR (101 MHz, $CDCl_3$) 38

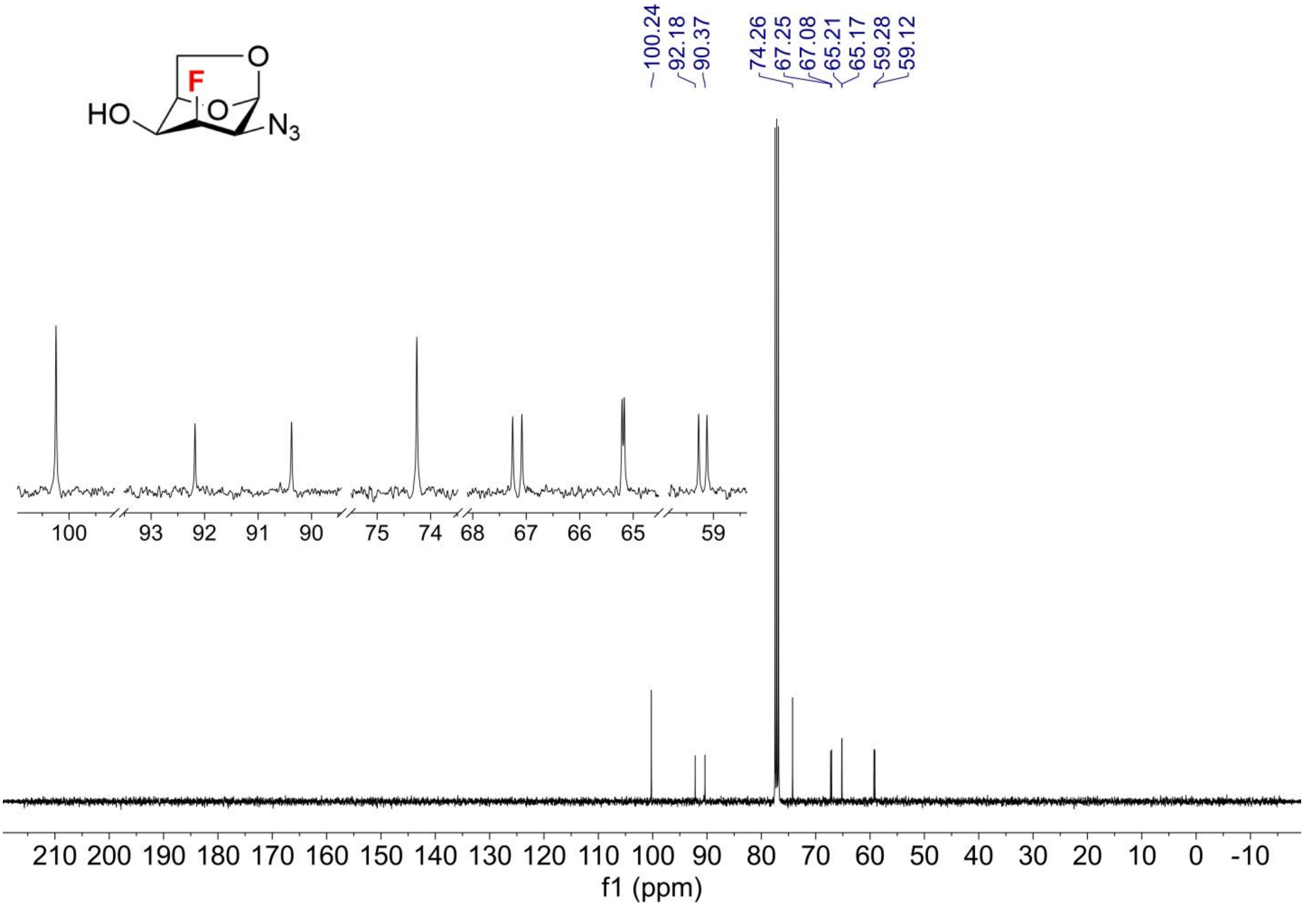

^{19}F NMR (376 MHz, CDCl$_3$) 38

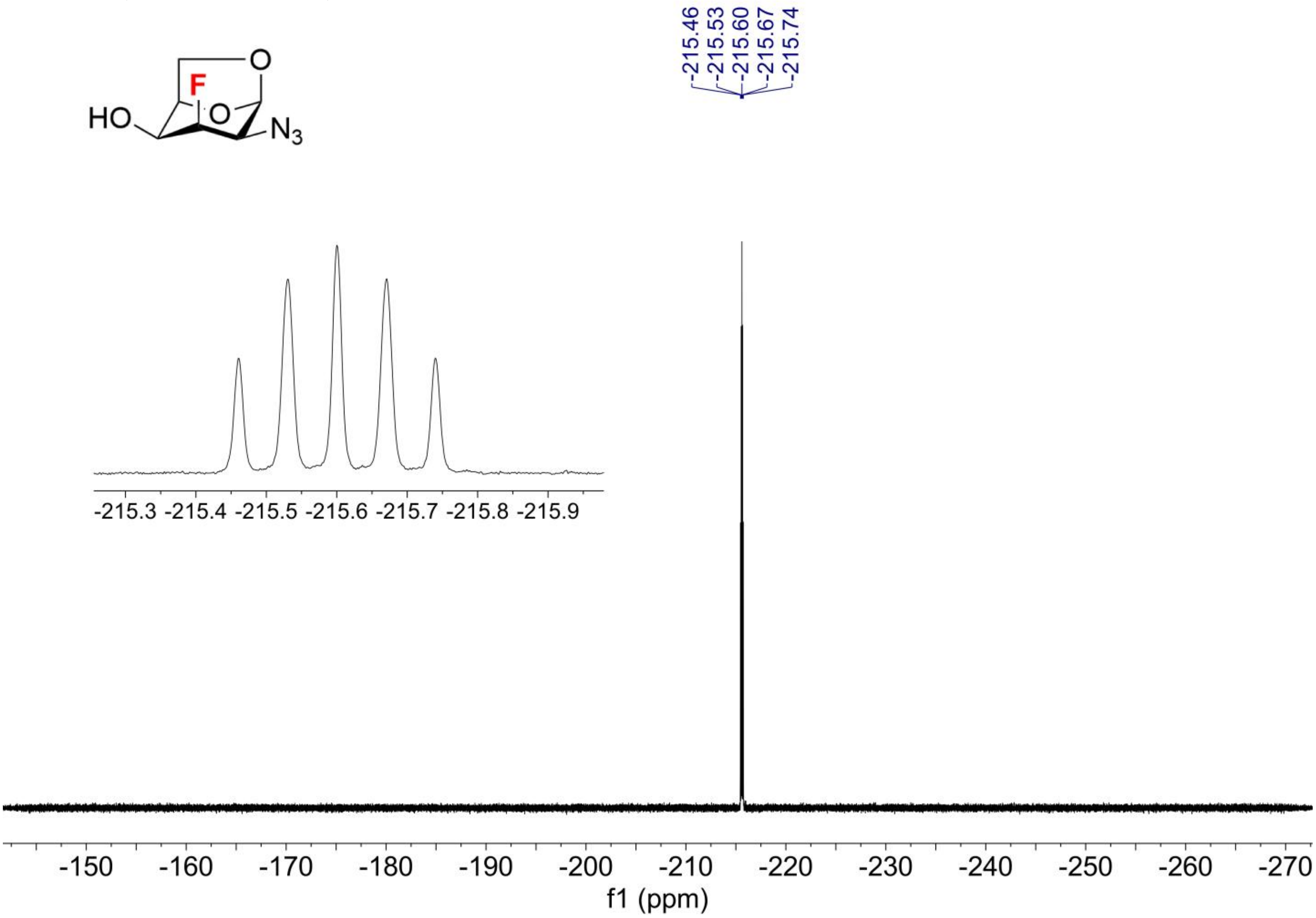

^{1}H-^{1}H COSY (400 MHz, CDCl$_3$) 38

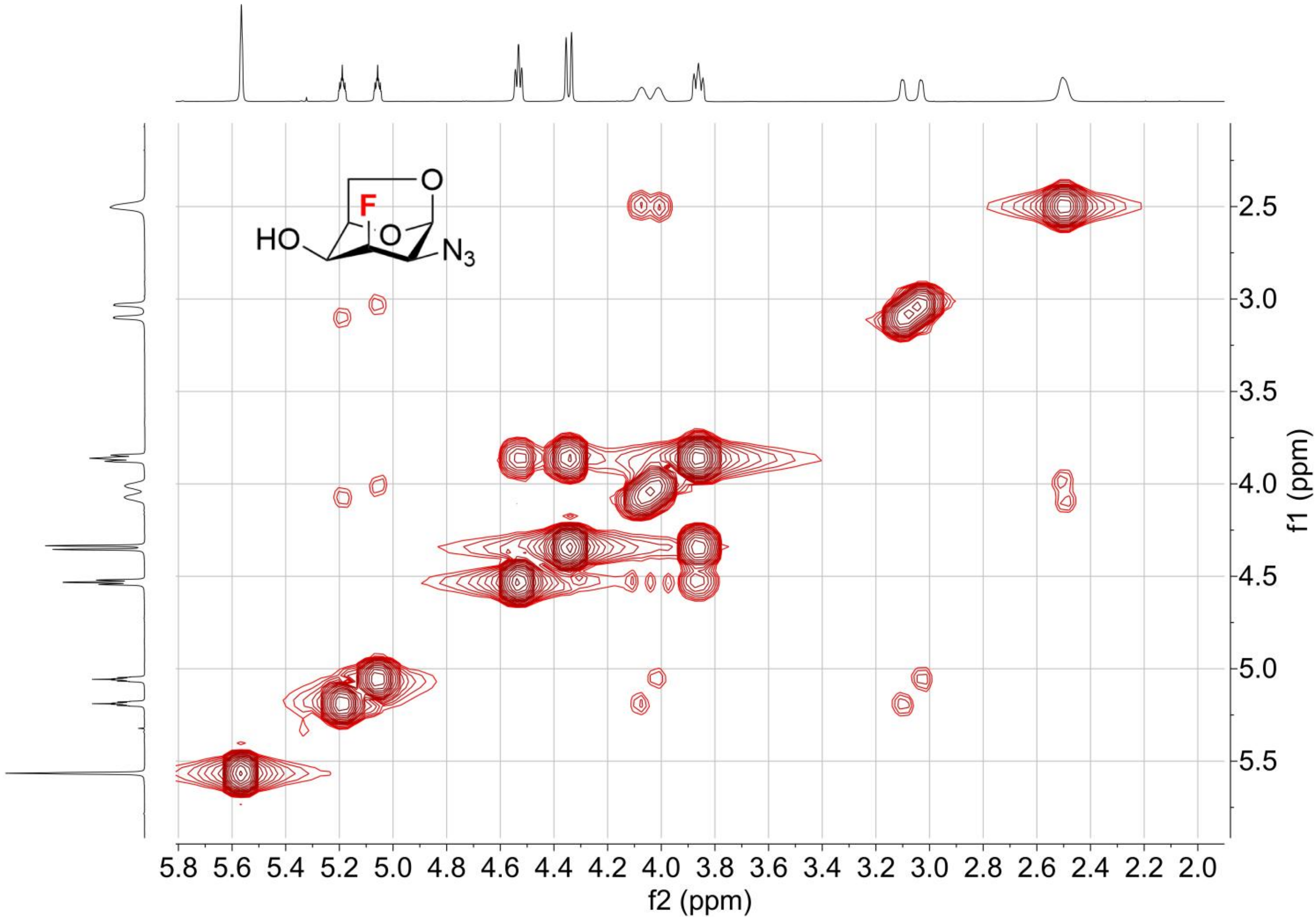

^{1}H-^{13}C HSQC (^{1}H/^{13}C 400/101 MHz, CDCl$_3$) 38

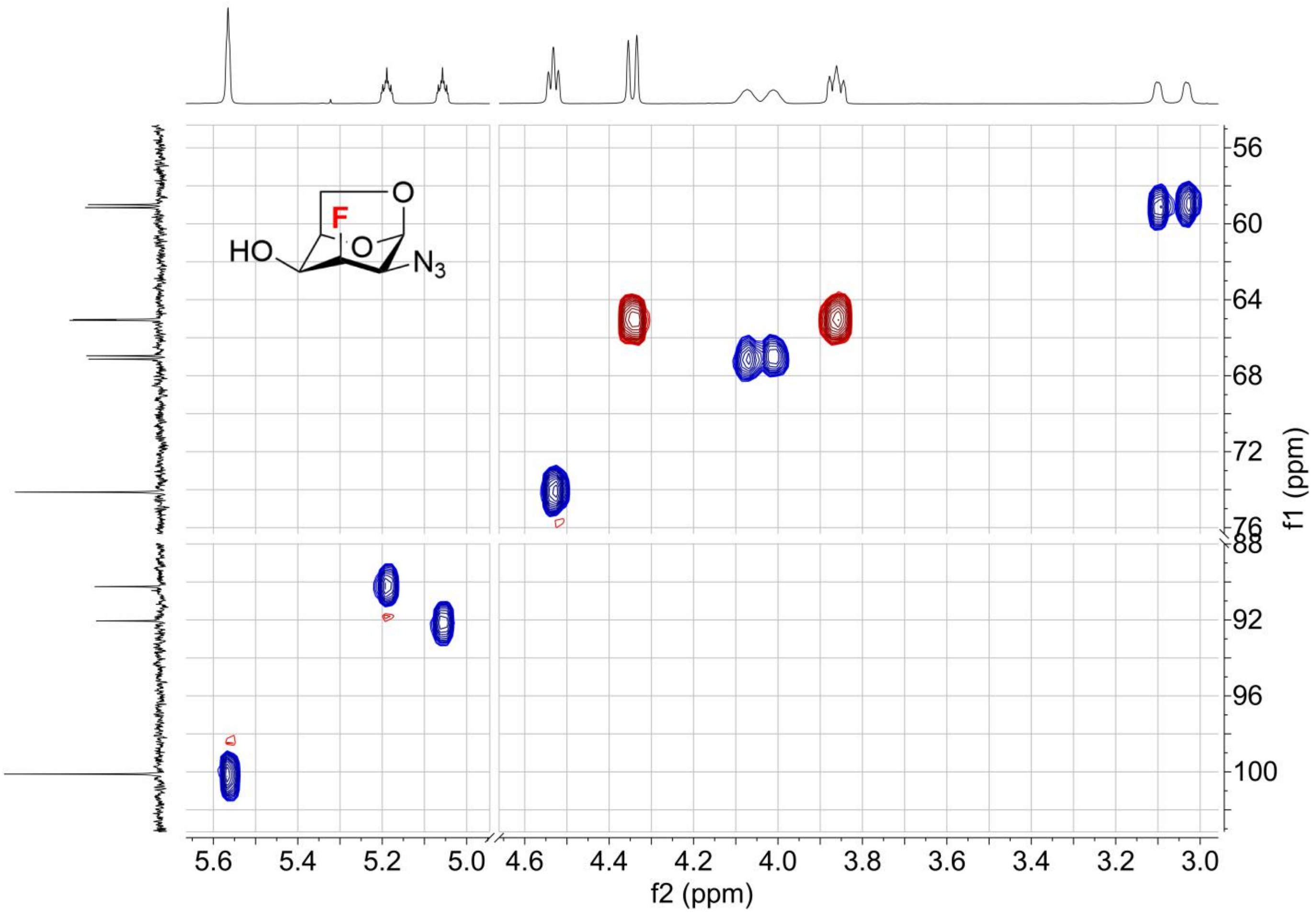

NMR COMPOUND 39

^{1}H NMR (400 MHz, $CDCl_3$) 39

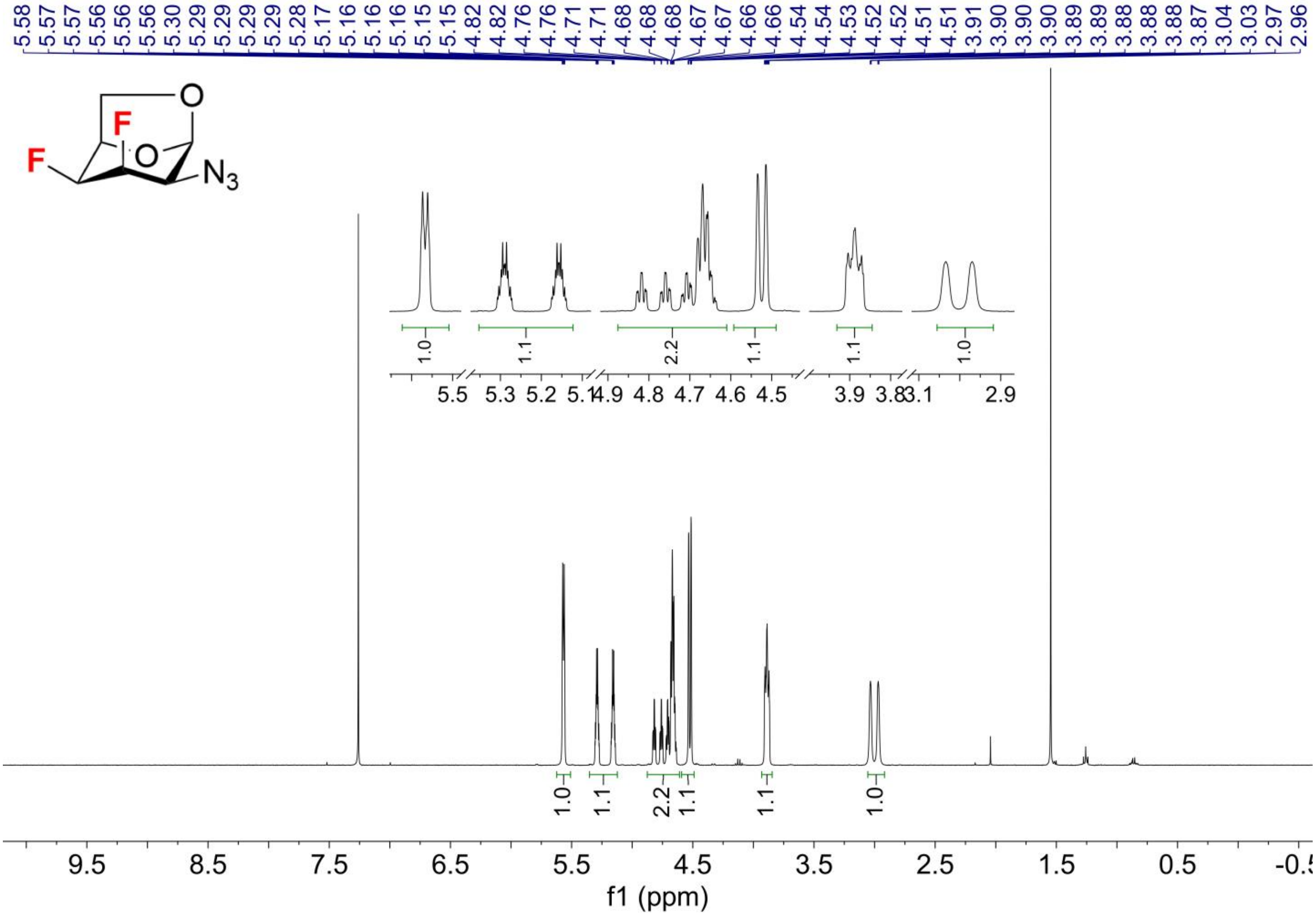

$^{13}C\{^{1}H\}$ NMR (101 MHz, $CDCl_3$) 39

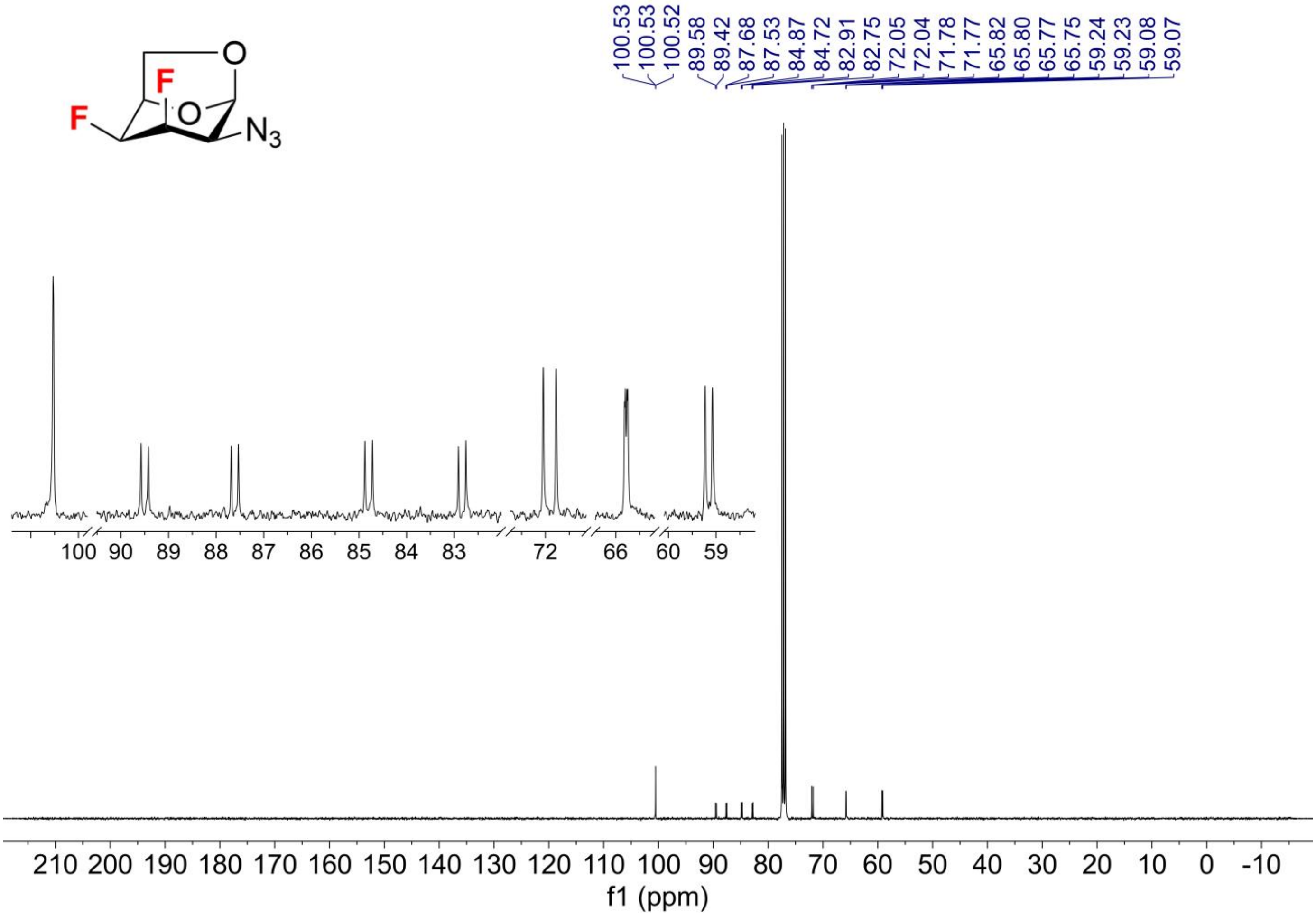

^{19}F NMR (376 MHz, $CDCl_3$) 39

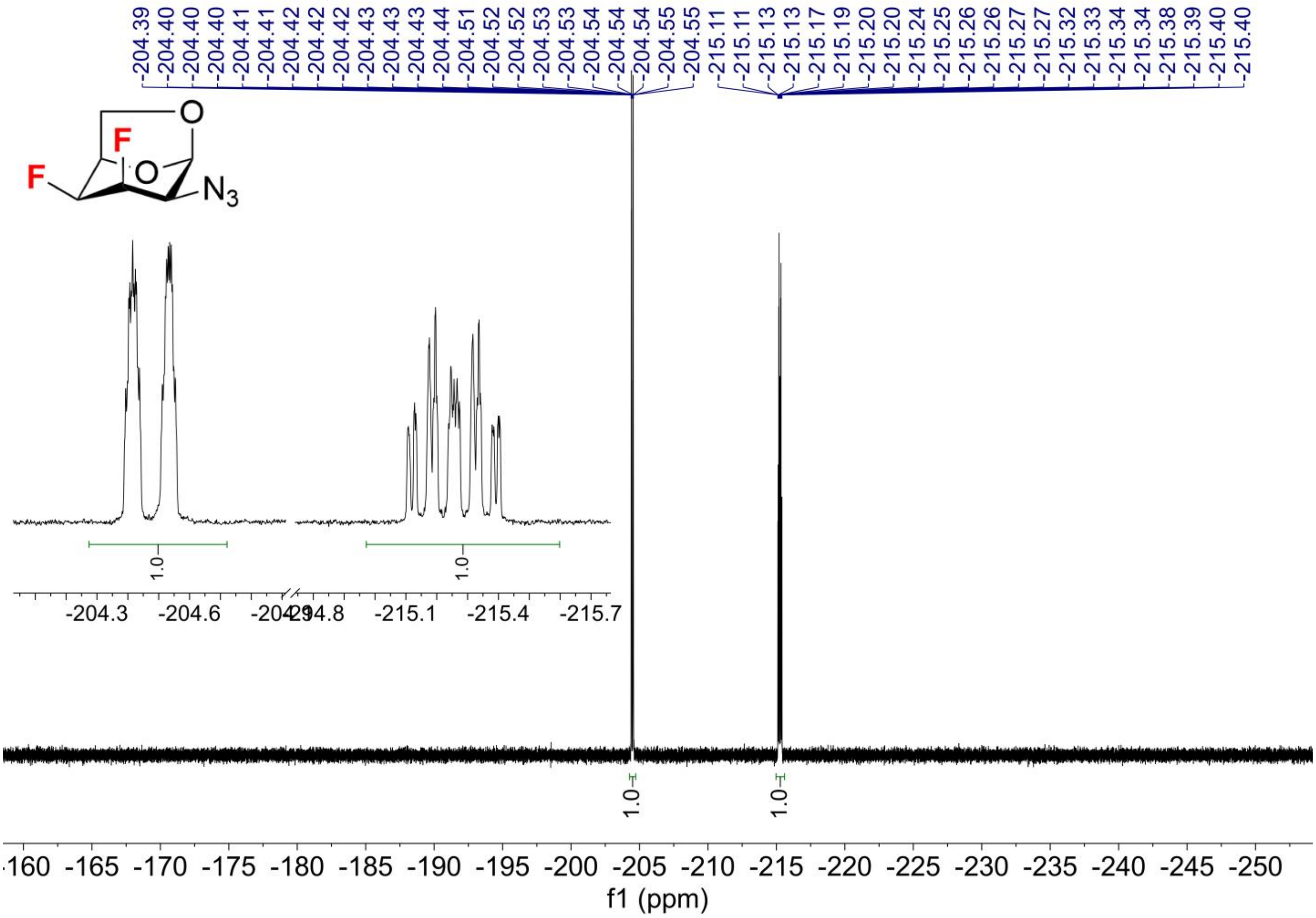

^{1}H-^{1}H COSY (400 MHz, $CDCl_3$) 39

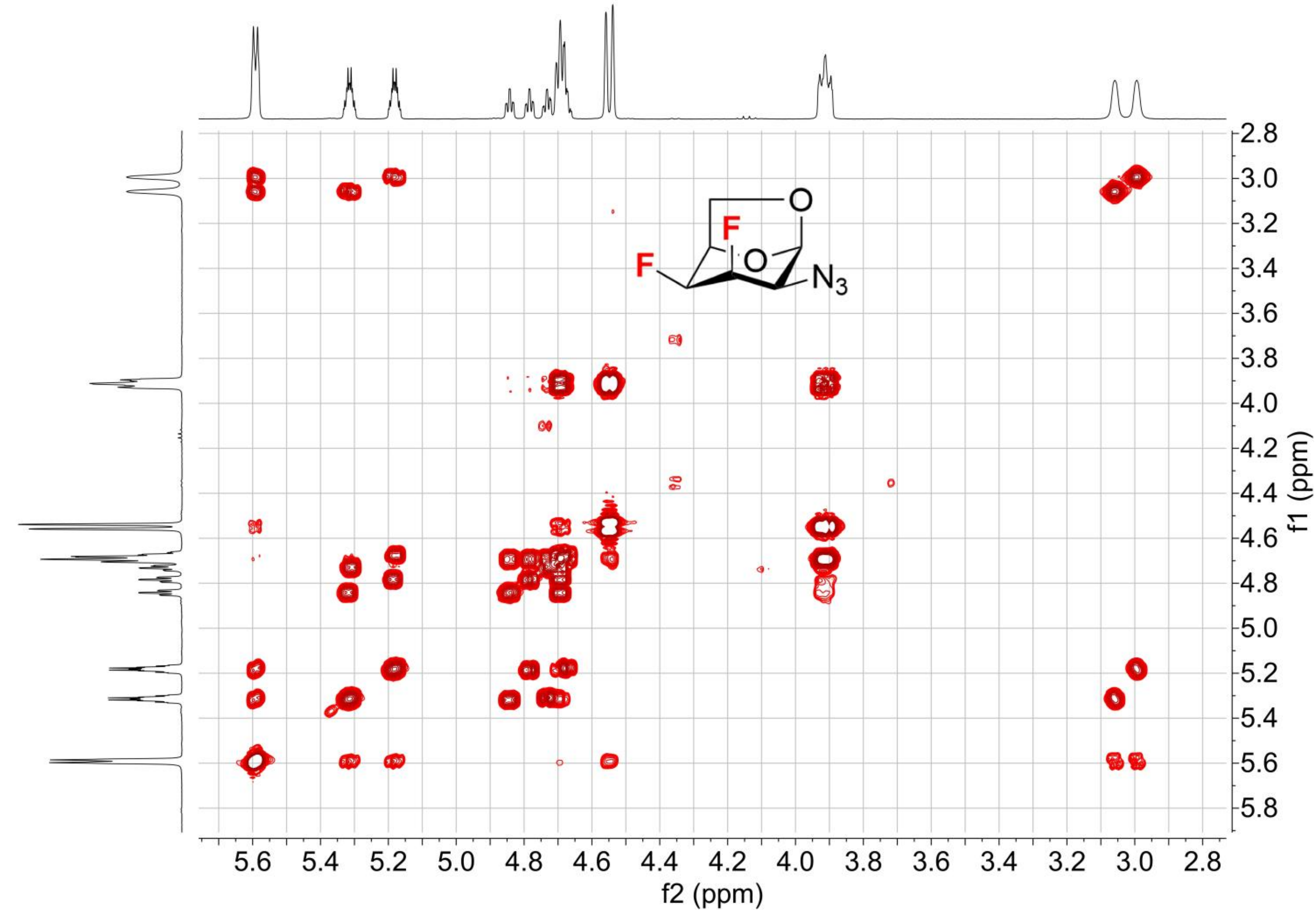

^{1}H-^{13}C HSQC (^{1}H/^{13}C 400/101 MHz, $CDCl_3$) 39

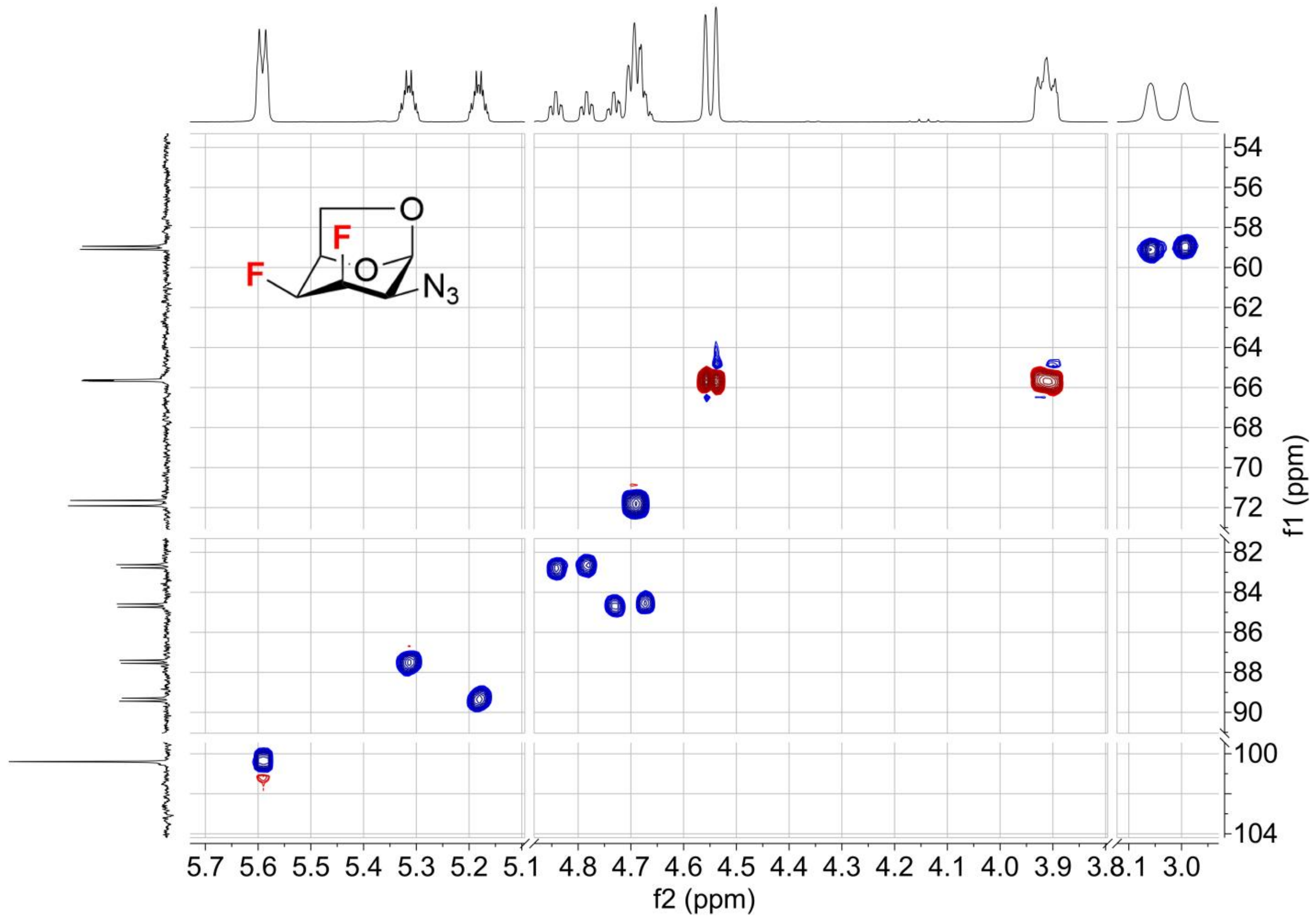

^{1}H-^{13}C HMBC (^{1}H/^{13}C 400/101 MHz, $CDCl_3$) 39

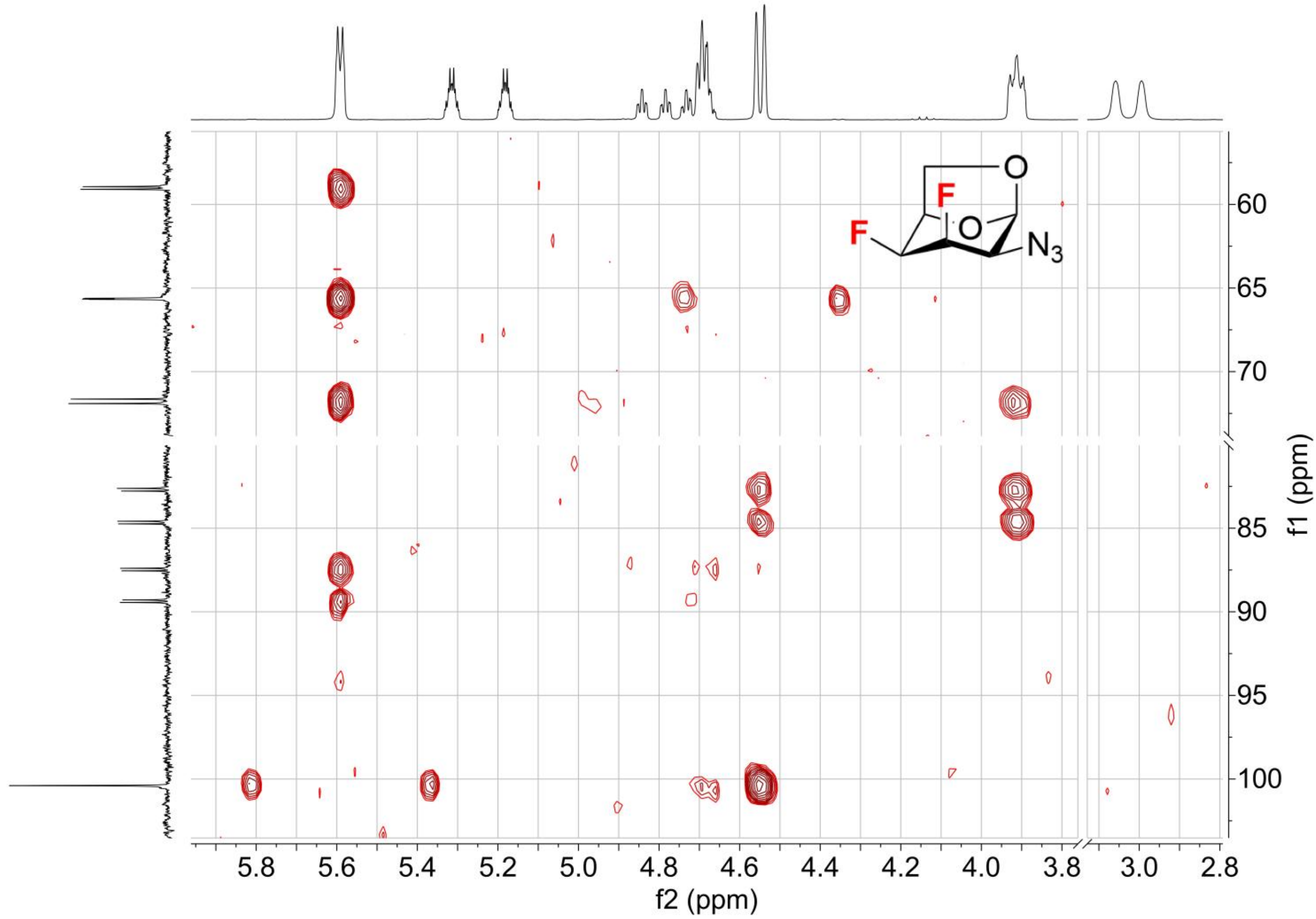

NMR COMPOUND α-48

^{1}H NMR (400 MHz, $CDCl_3$) α-48

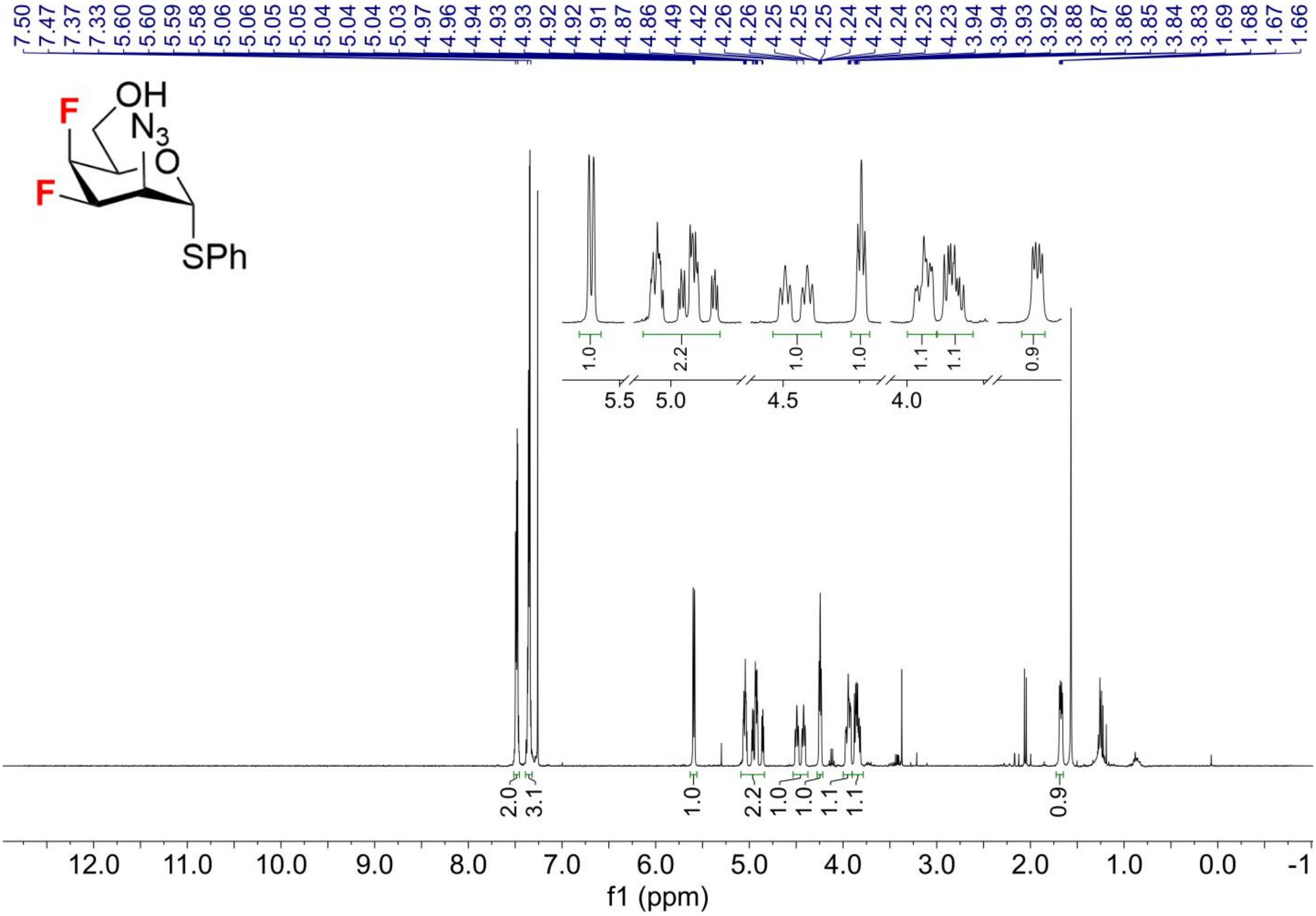

^{13}C{^{1}H} NMR (101 MHz, $CDCl_3$) α-48

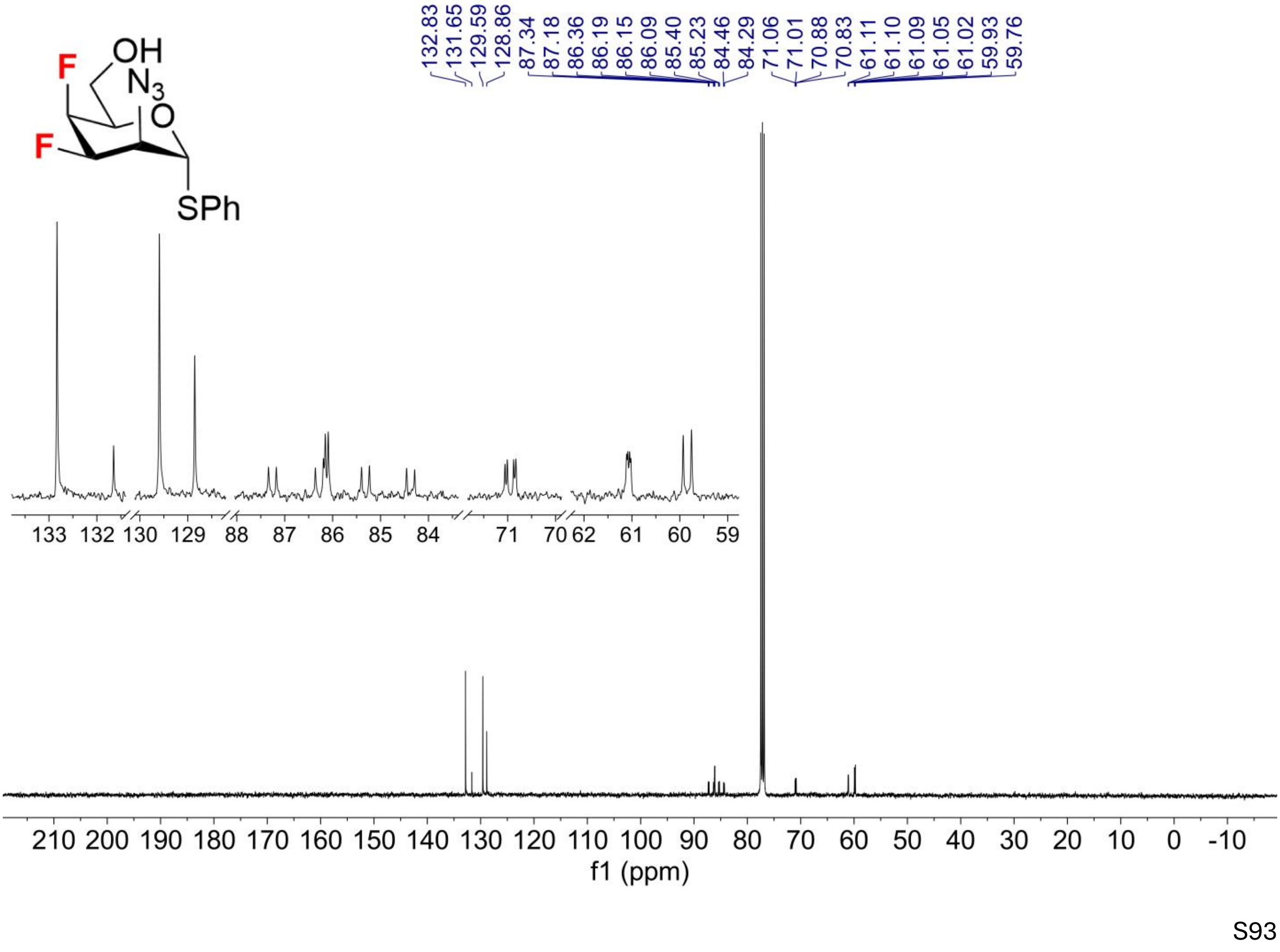

^{19}F NMR (376 MHz, $CDCl_3$) α-48

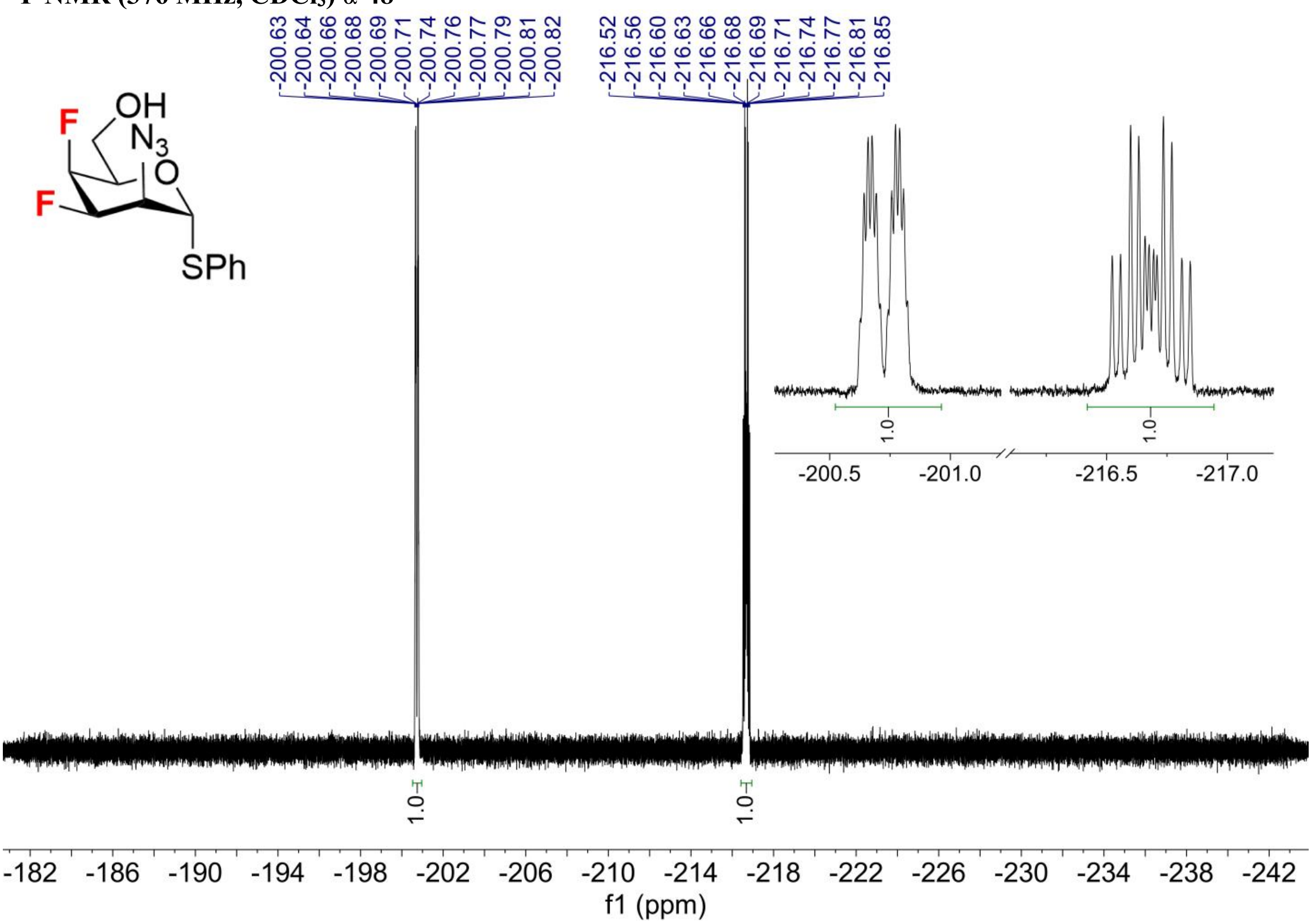

^{1}H-^{1}H COSY (400 MHz, $CDCl_3$) α-48

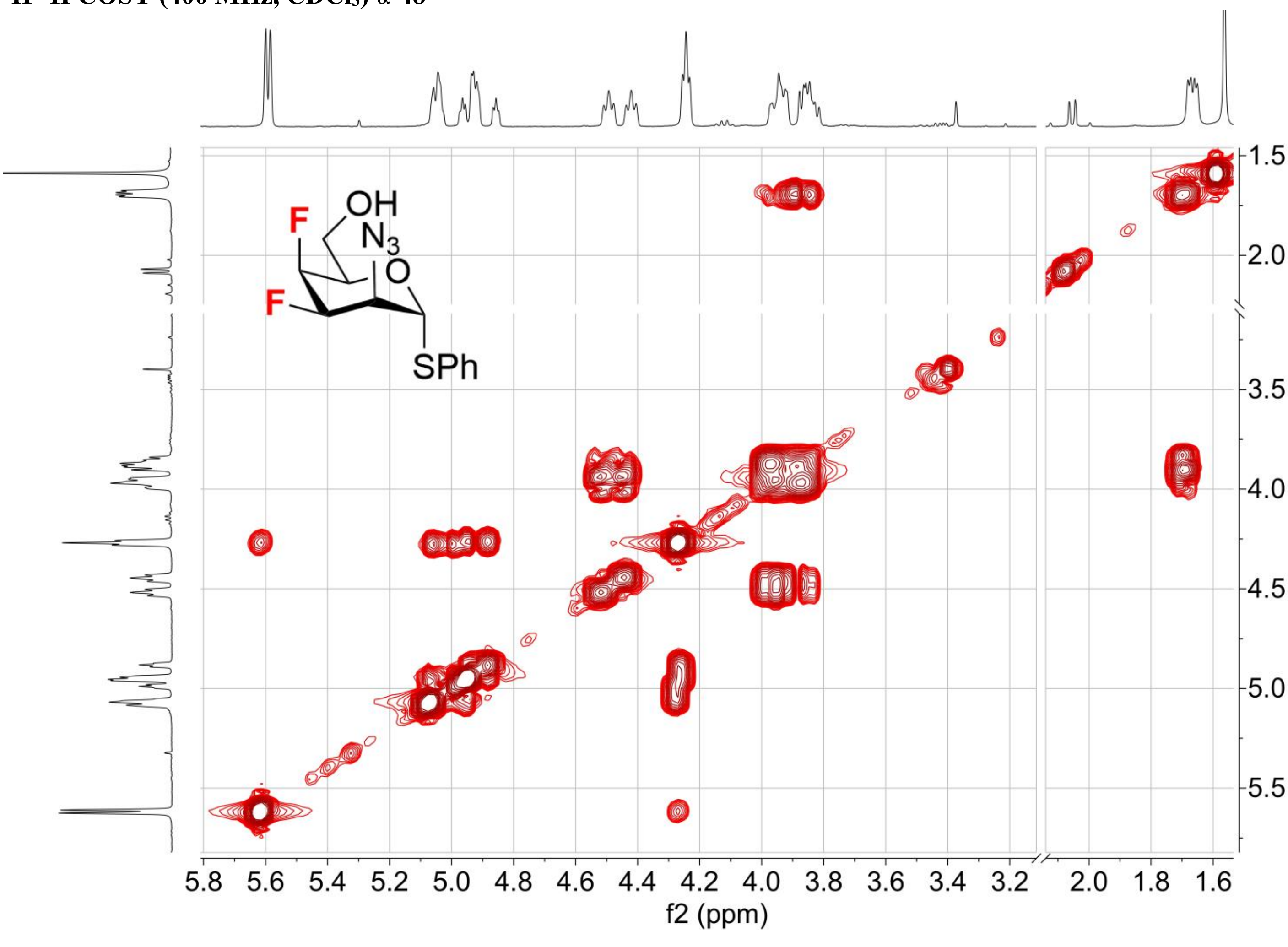

^1H-^{13}C HSQC (^1H/^{13}C 400/101 MHz, $CDCl_3$) α-48

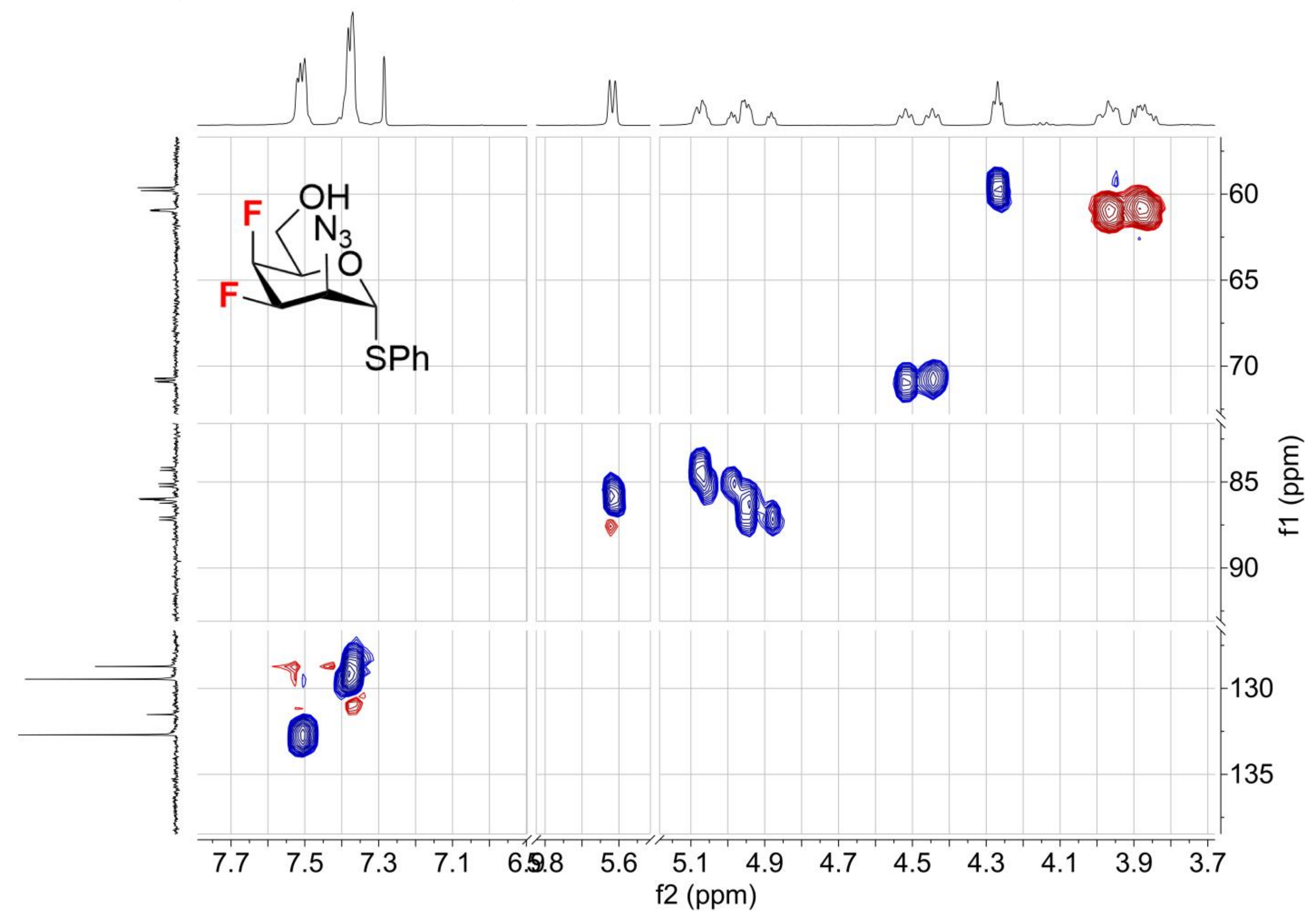

^1H-^{13}C HMBC (^1H/^{13}C 400/101 MHz, $CDCl_3$) α-48

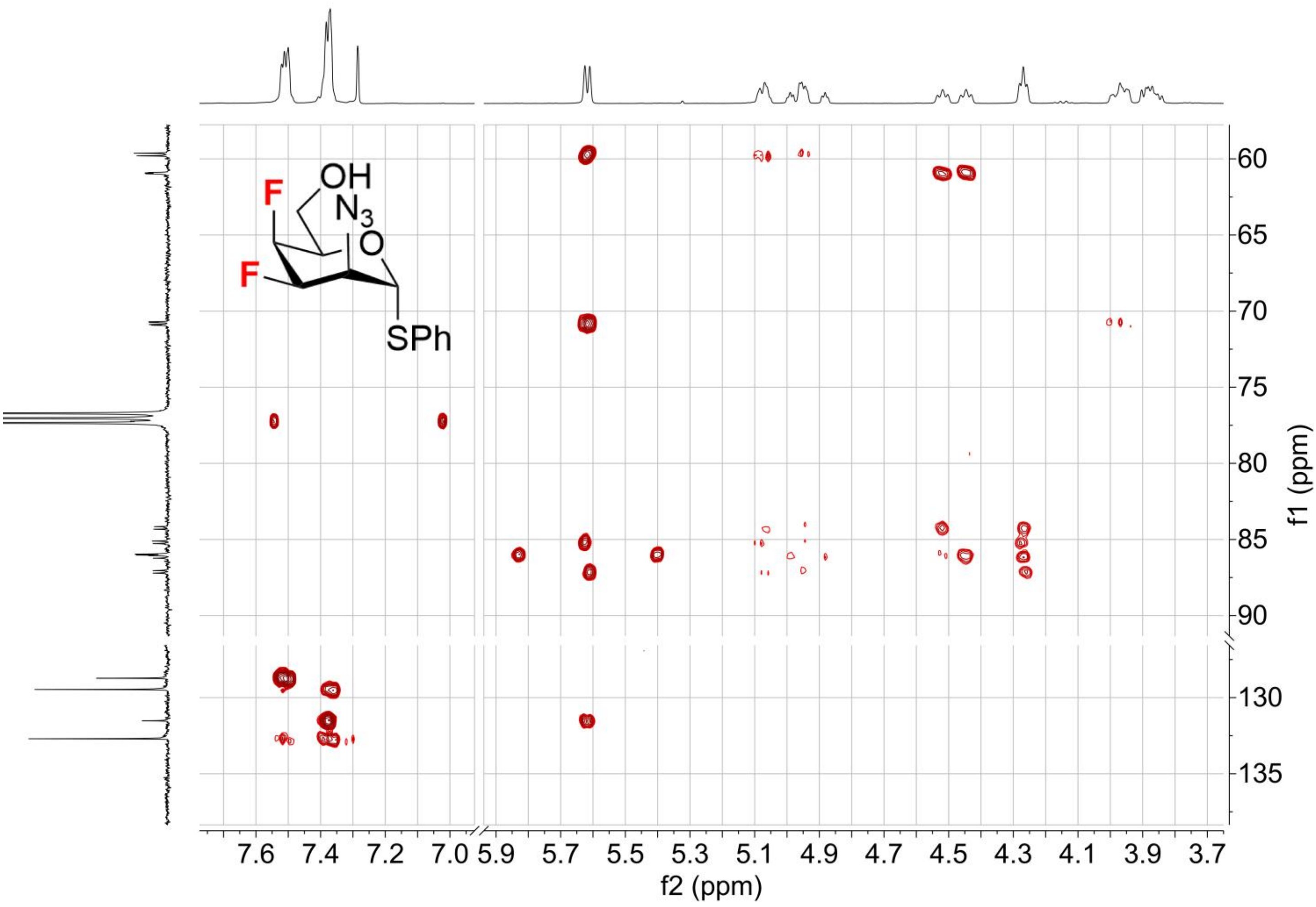

NMR COMPOUND β-48

^{1}H NMR (400 MHz, CDCl$_3$) β-48

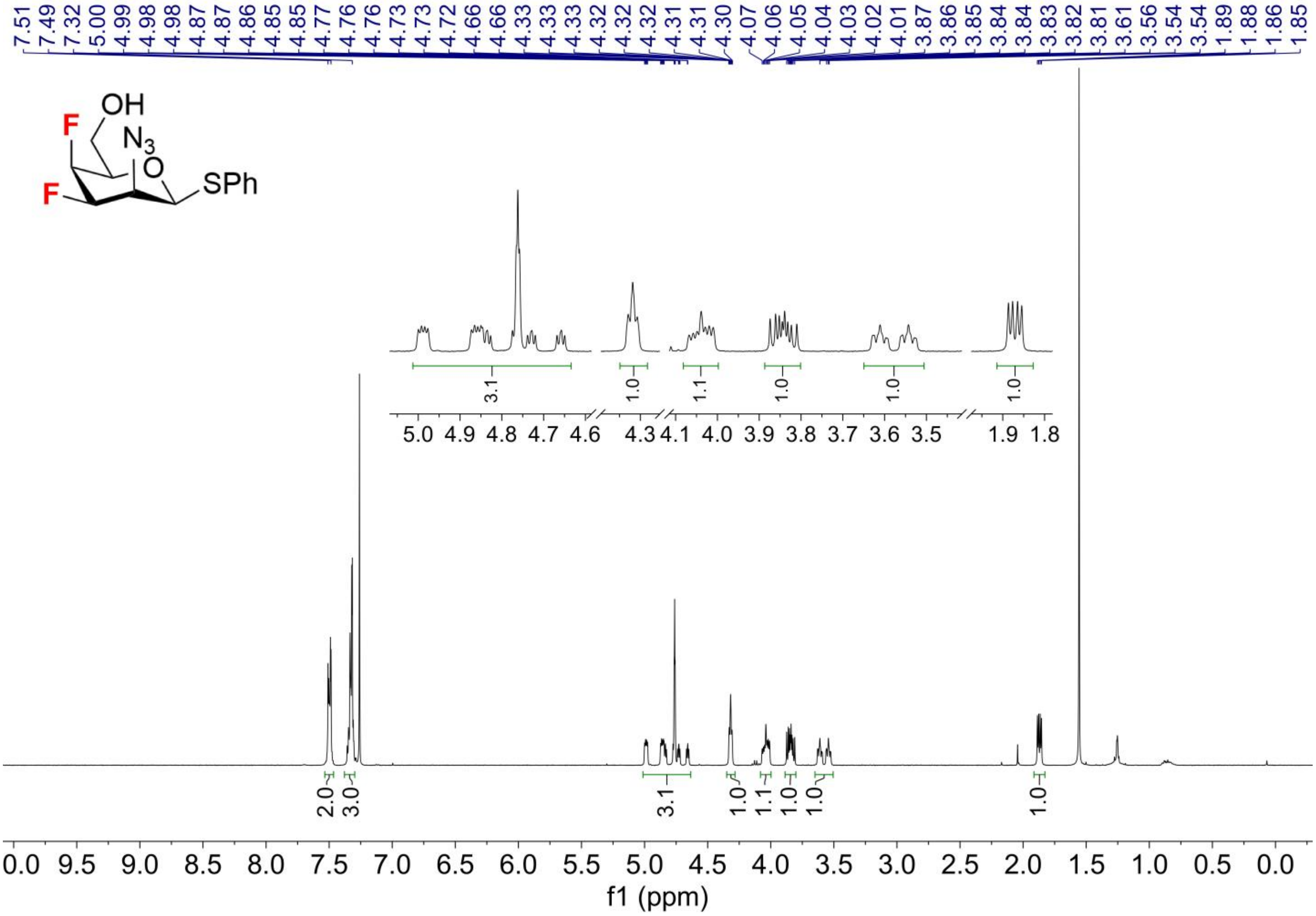

^{13}C{^{1}H} NMR (101 MHz, CDCl$_3$) β-48

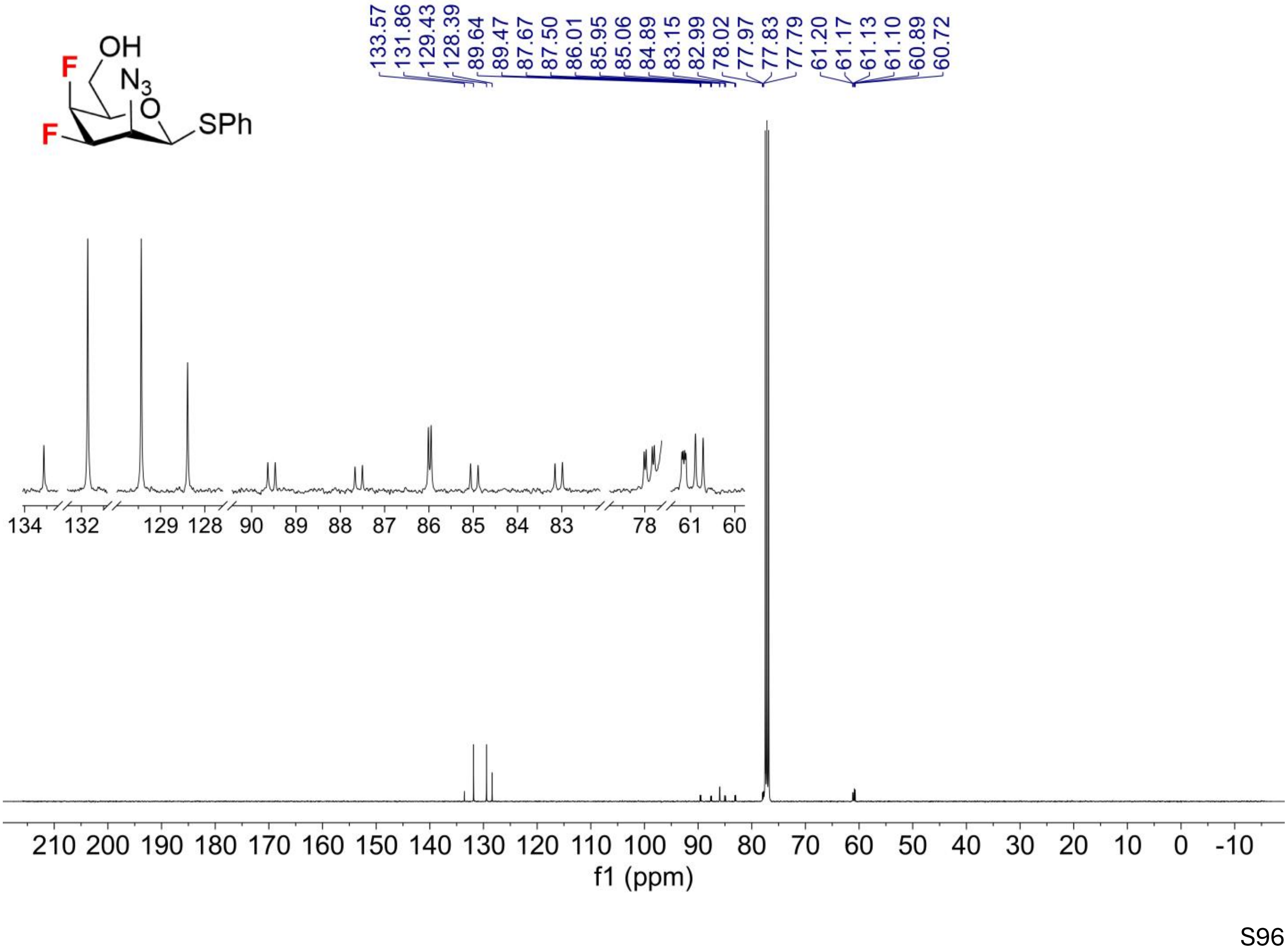

^{19}F NMR (376 MHz, CDCl$_3$) β-48

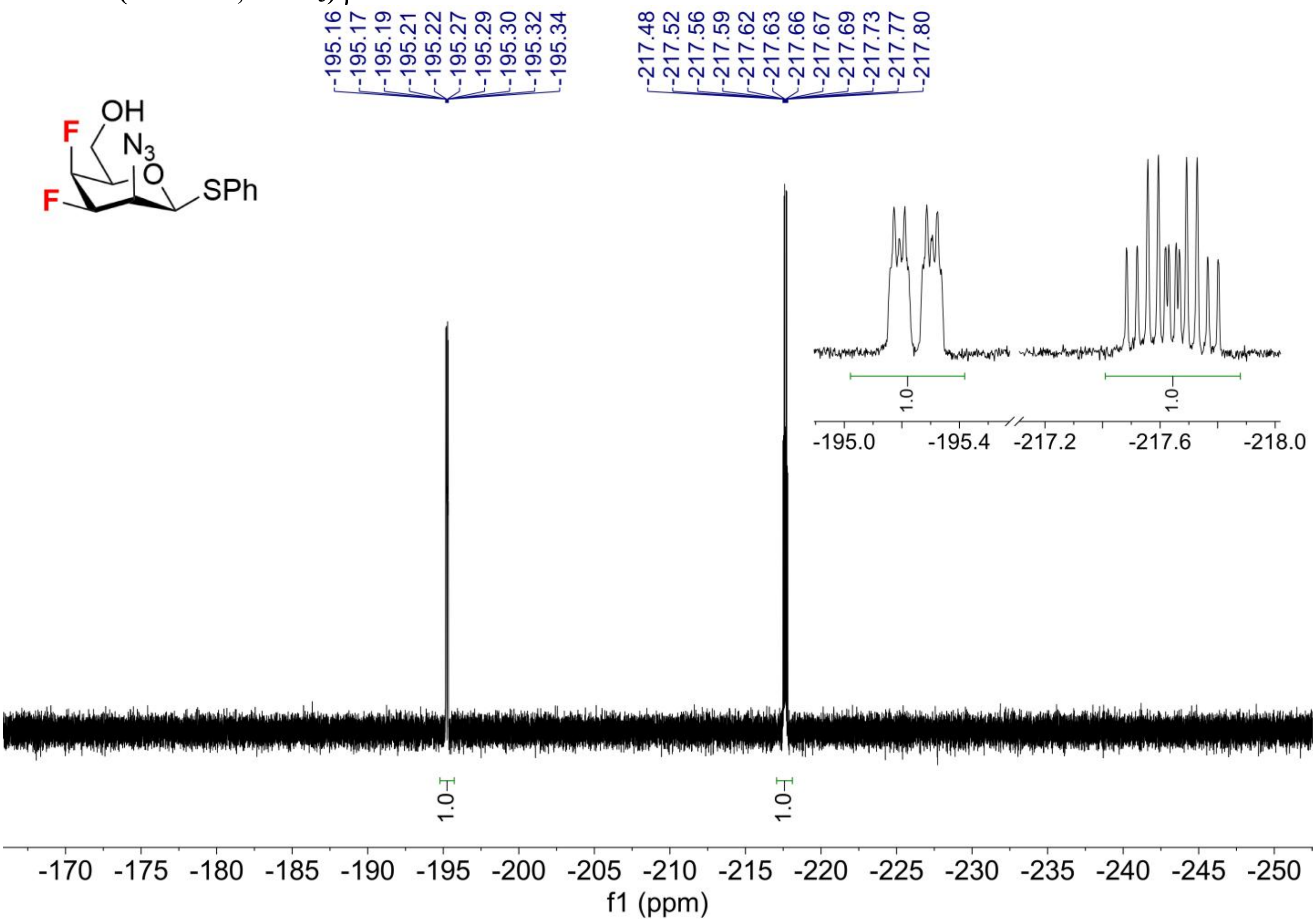

^{1}H-^{1}H COSY (400 MHz, CDCl$_3$) β-48

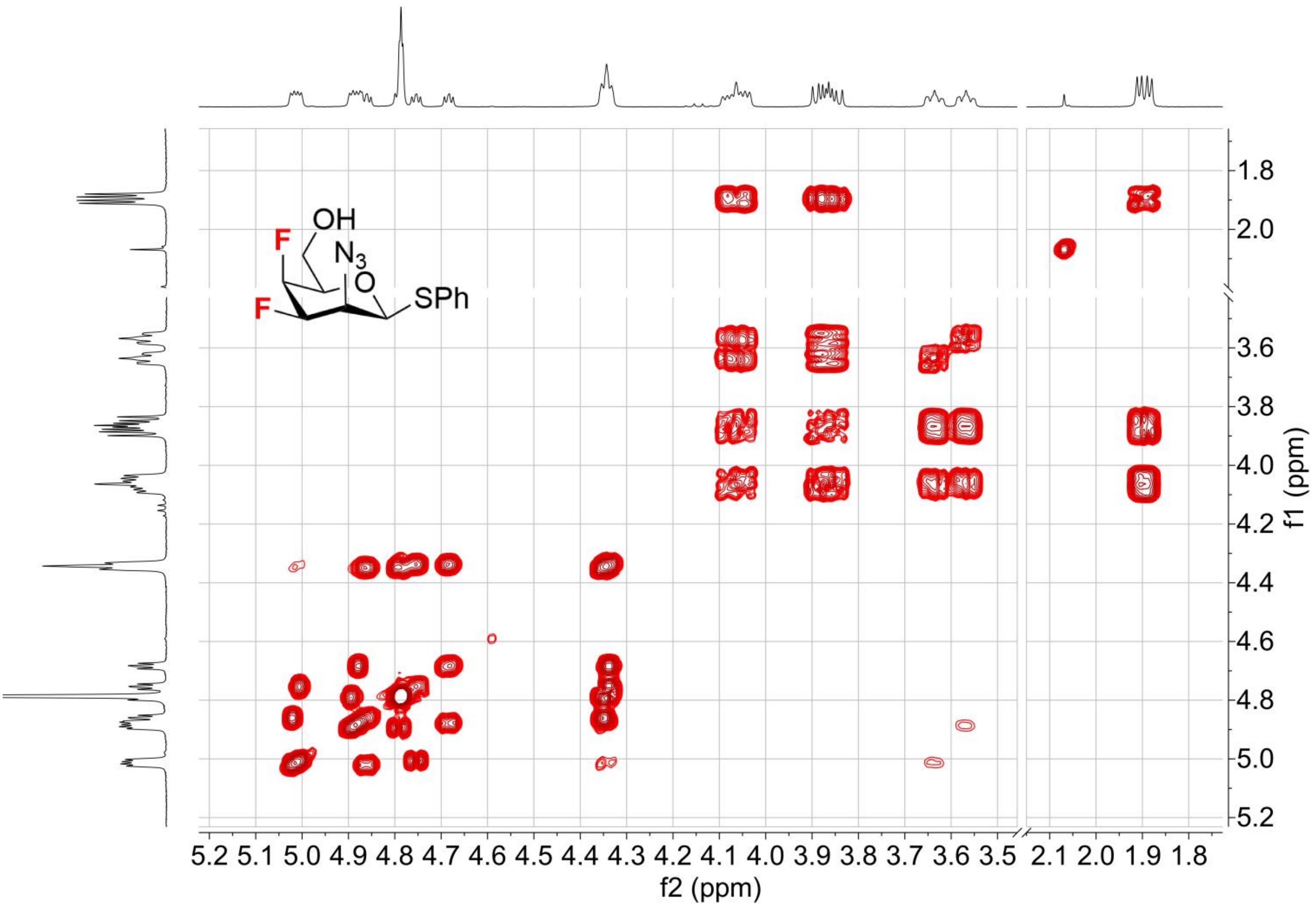

^{1}H-^{13}C HSQC (^{1}H/^{13}C 400/101 MHz, CDCl$_3$) β-48

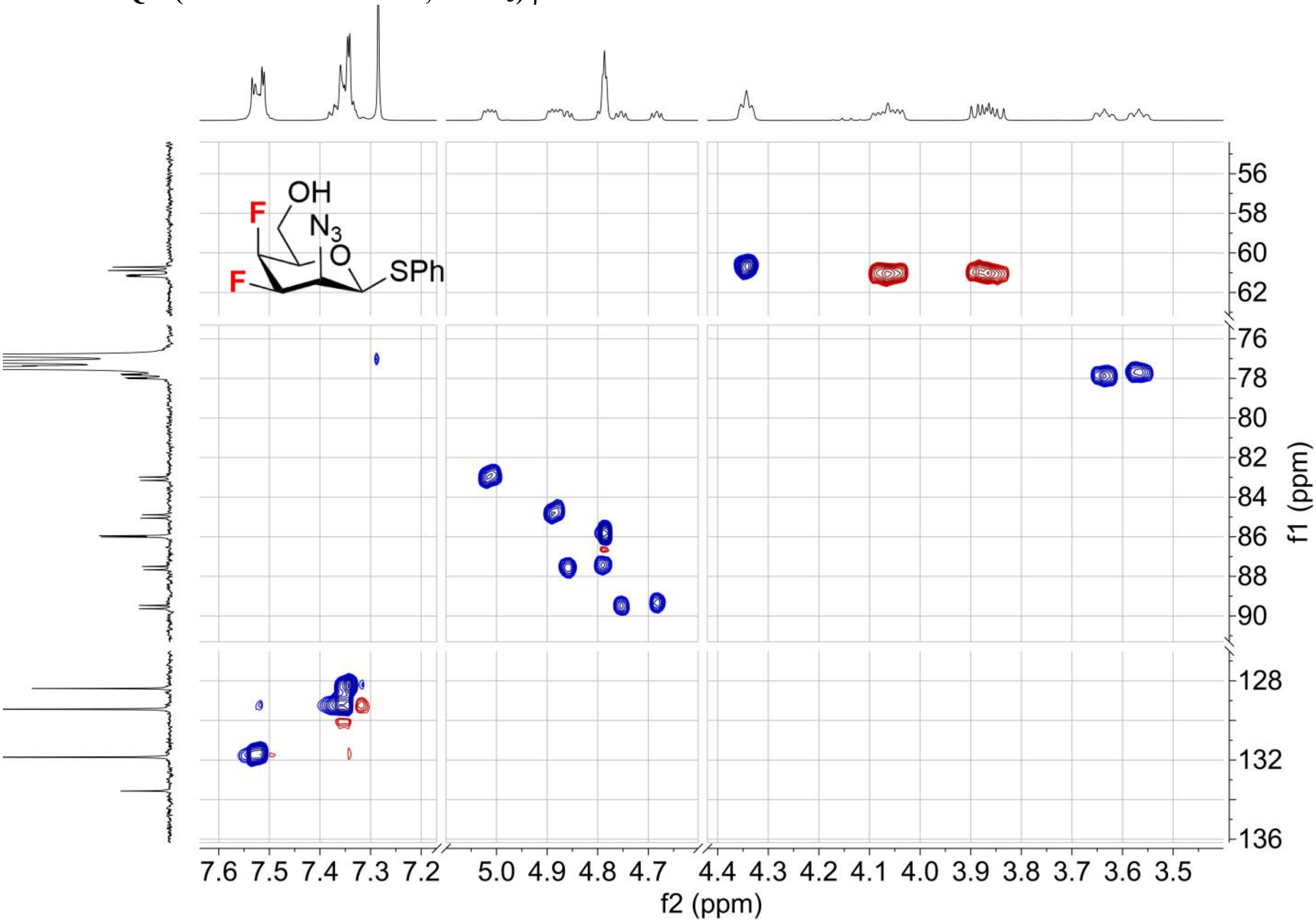

NMR COMPOUND 49

^{1}H NMR (400 MHz, $CDCl_3$) 49

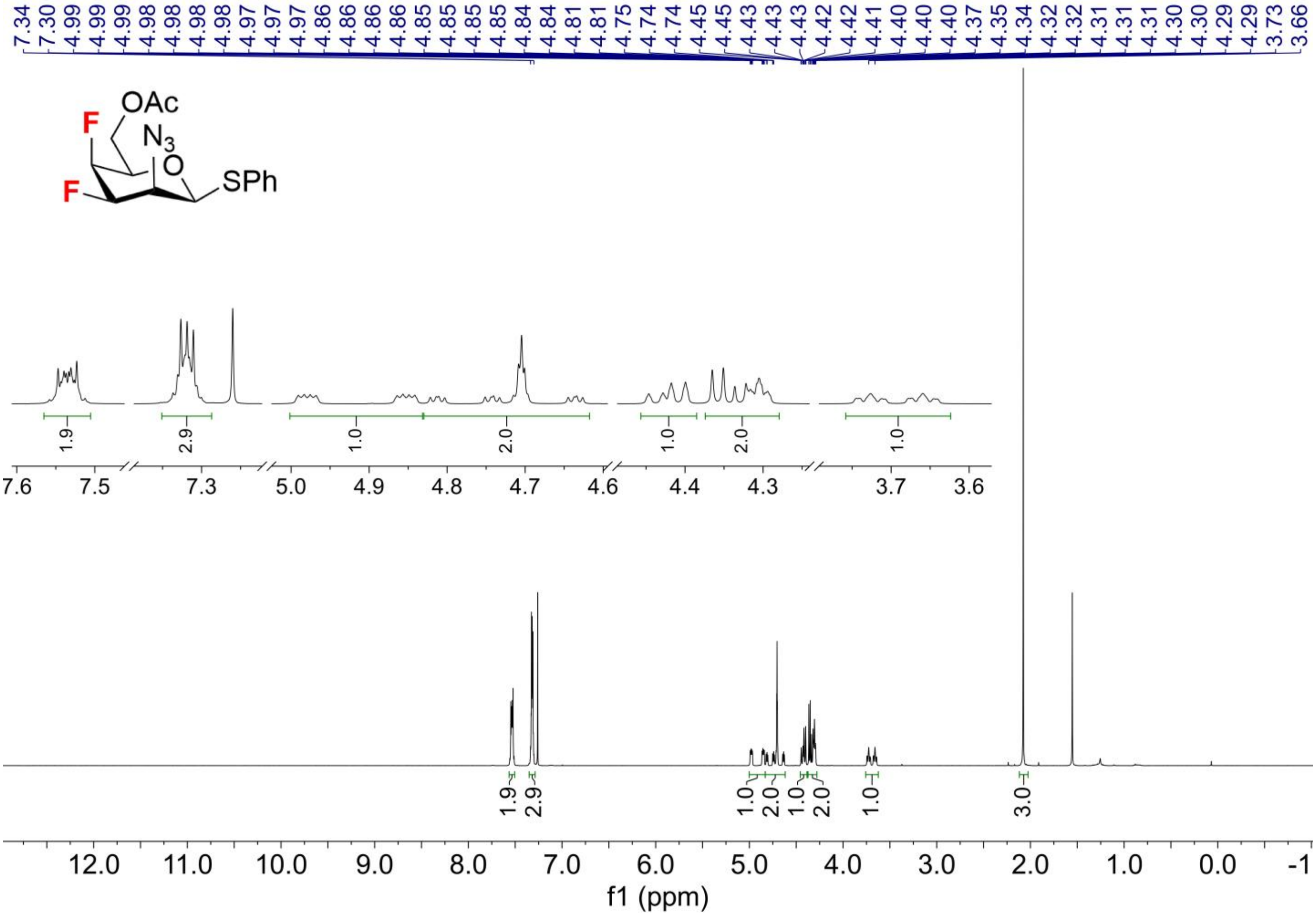

^{13}C{^{1}H} NMR (101 MHz, $CDCl_3$) 49

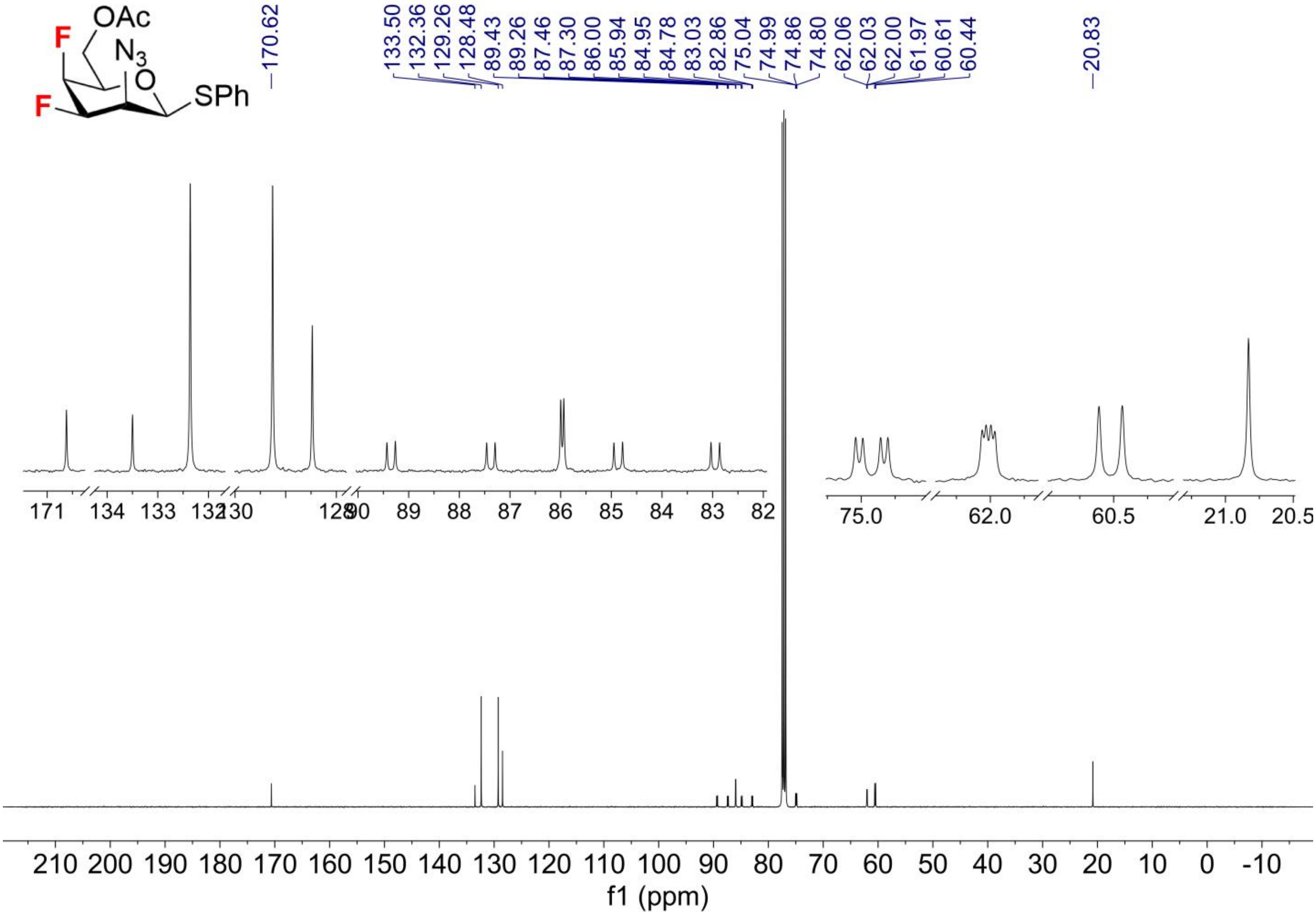

^{19}F NMR (376 MHz, $CDCl_3$) 49

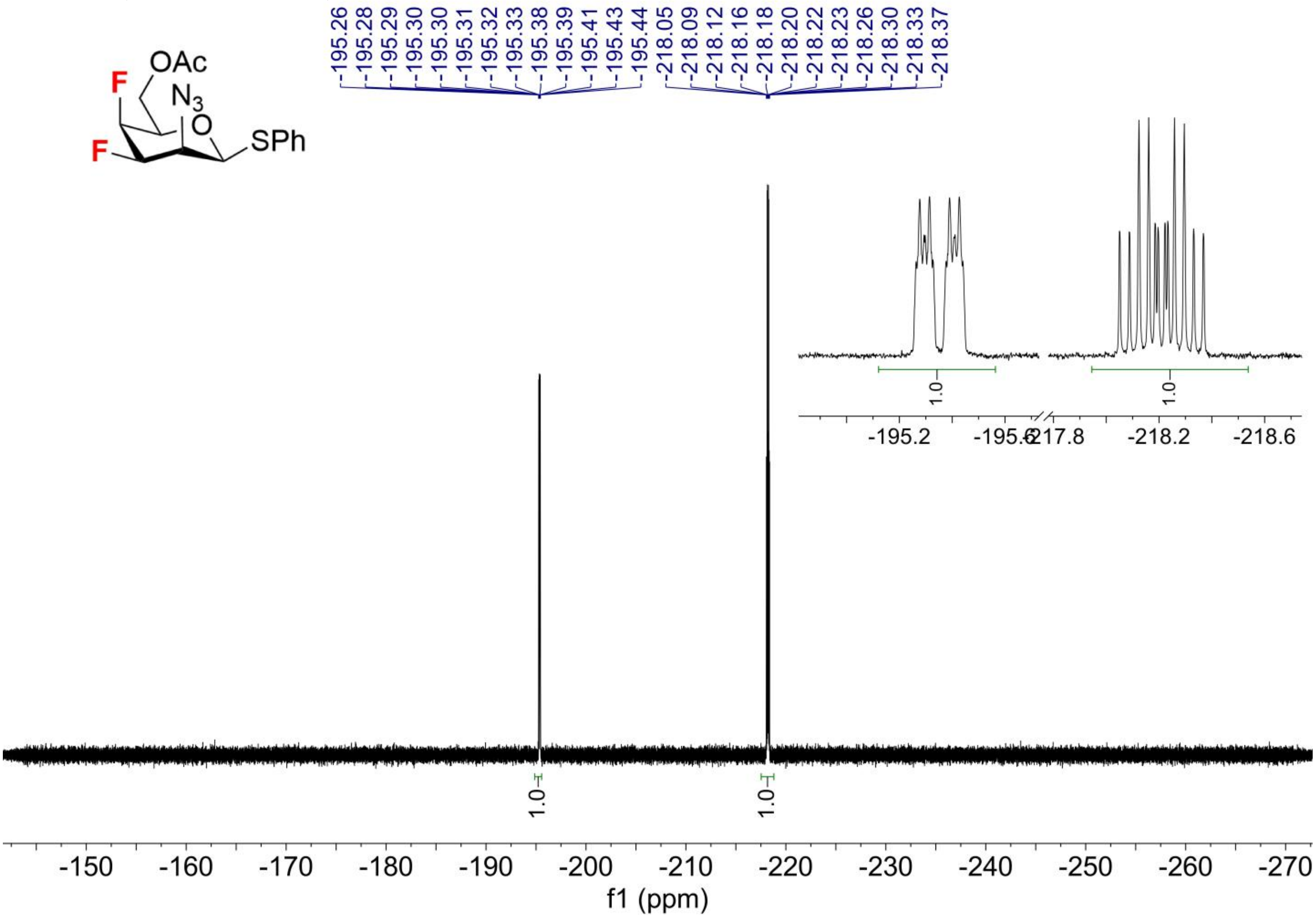

^{1}H-^{1}H COSY (400 MHz, $CDCl_3$) 49

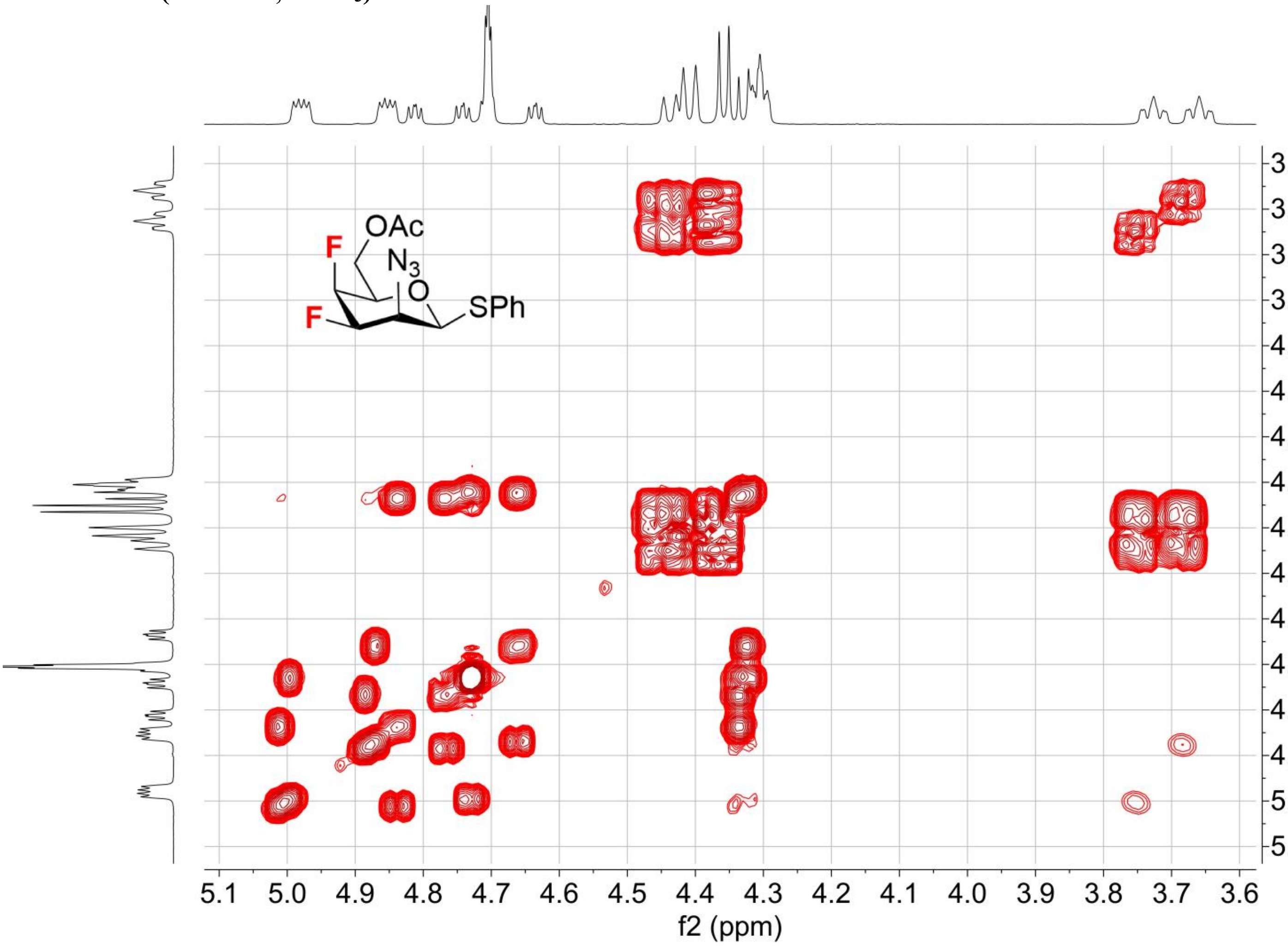

^{1}H-^{13}C HSQC (^{1}H/^{13}C 400/101 MHz, CDCl$_3$) 49

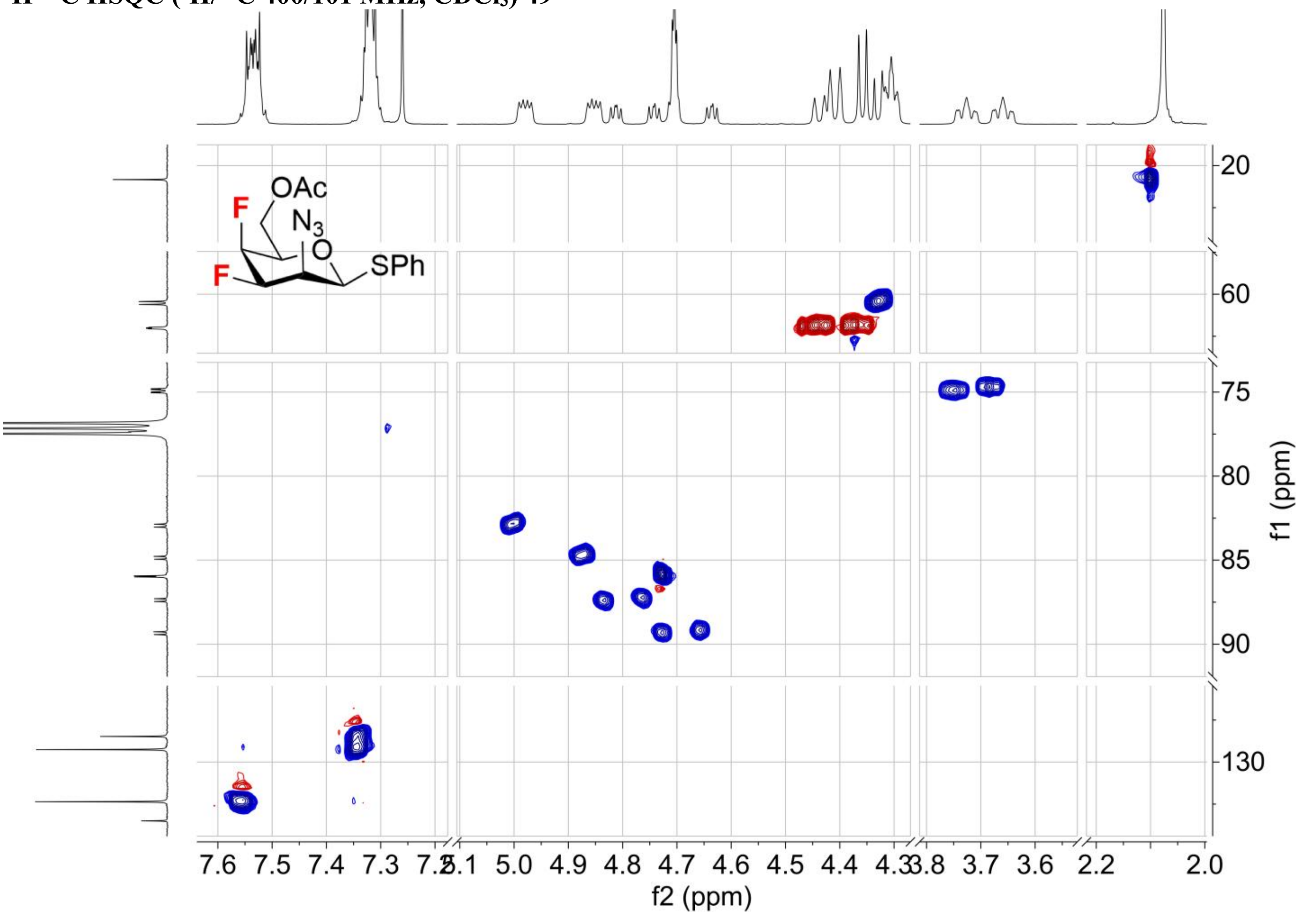

^{1}H-^{13}C HMBC (^{1}H/^{13}C 400/101 MHz, CDCl$_3$) 49

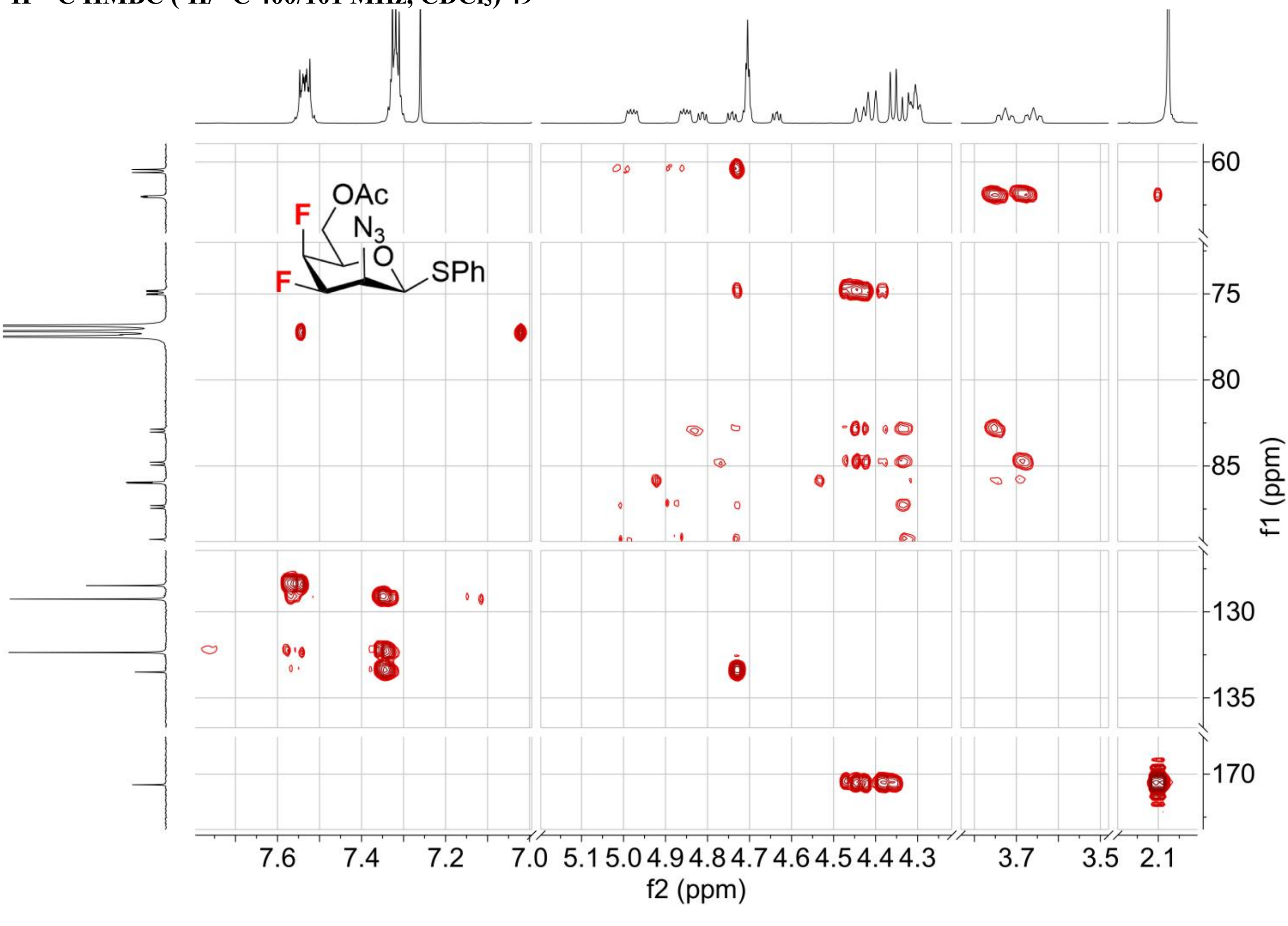

NMR COMPOUND 50

^{1}H NMR (400 MHz, CD_3OD)50

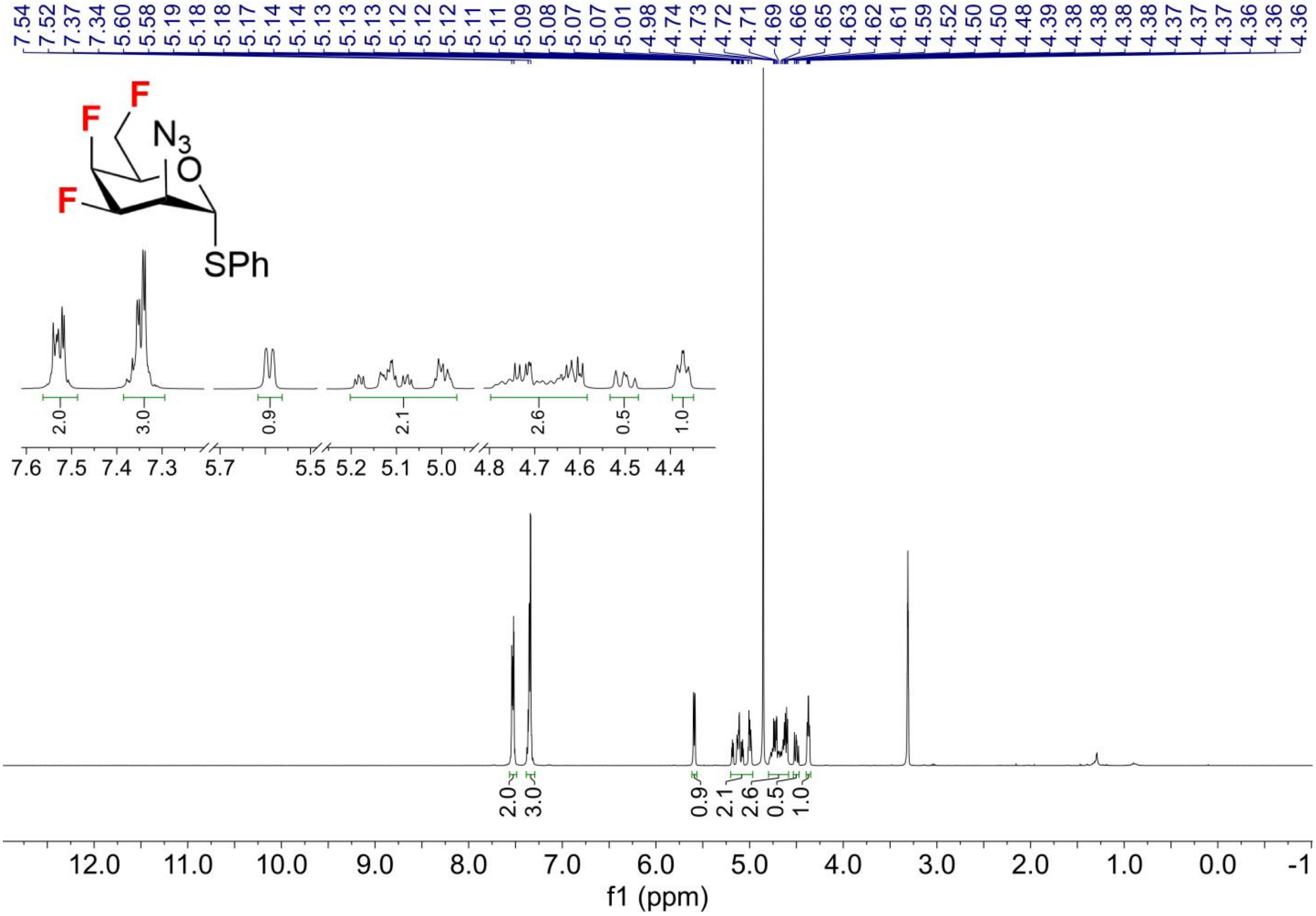

$^{13}C\{^{1}H\}$ NMR (101 MHz, CD_3OD) 50

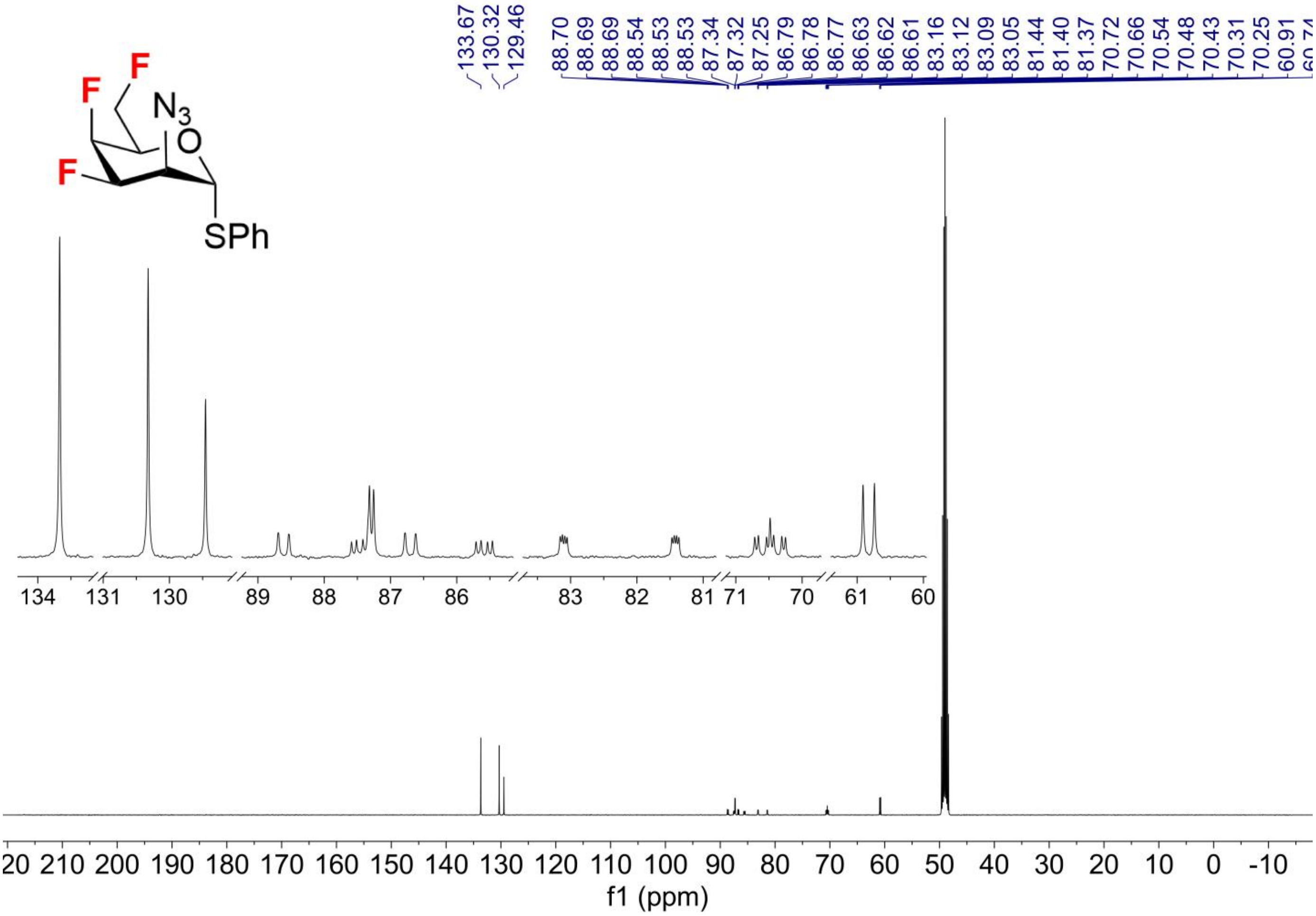

^{19}F NMR (376 MHz, CD_3OD) 50

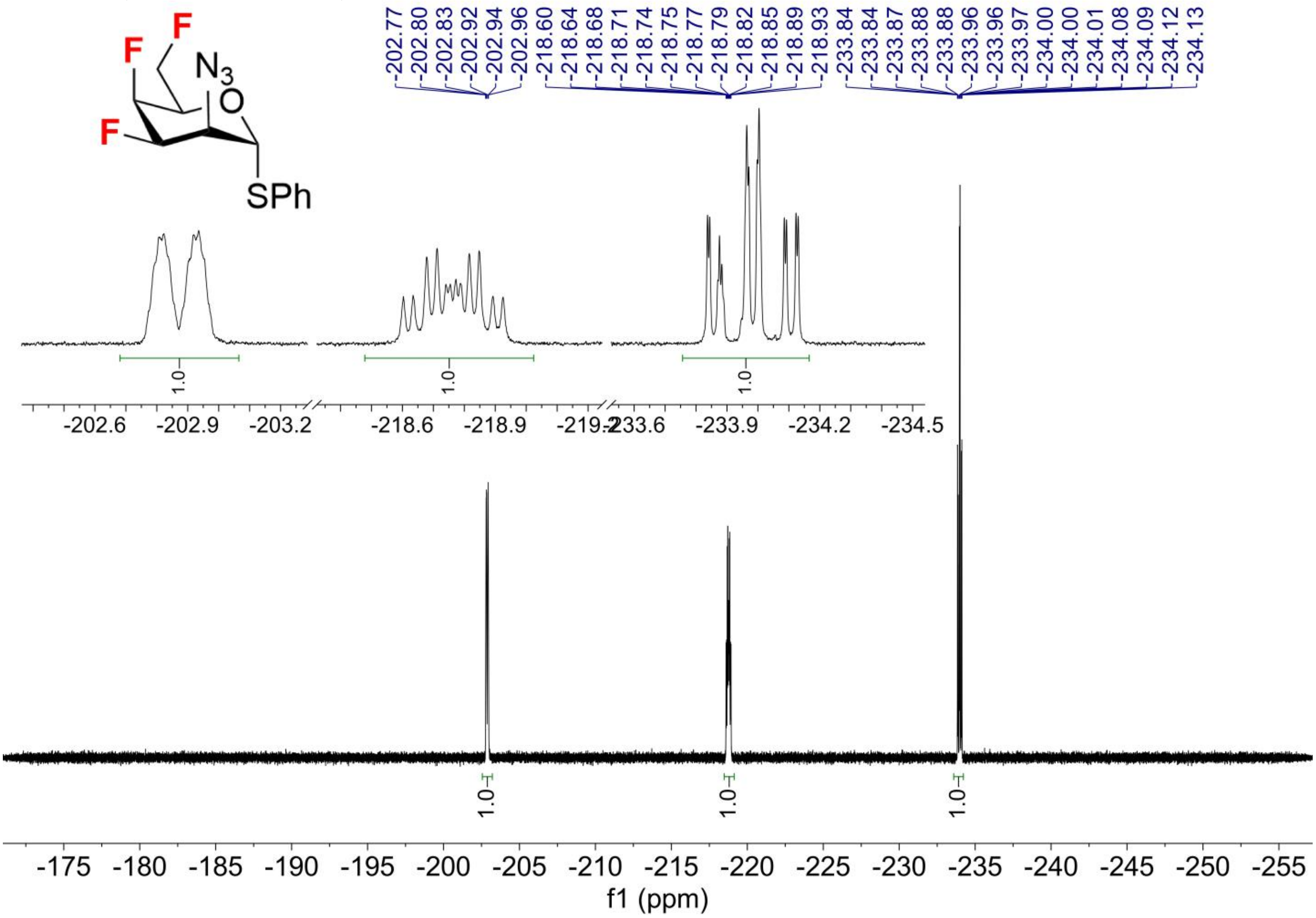

^{1}H-^{1}H COSY (400 MHz, CD_3OD) 50

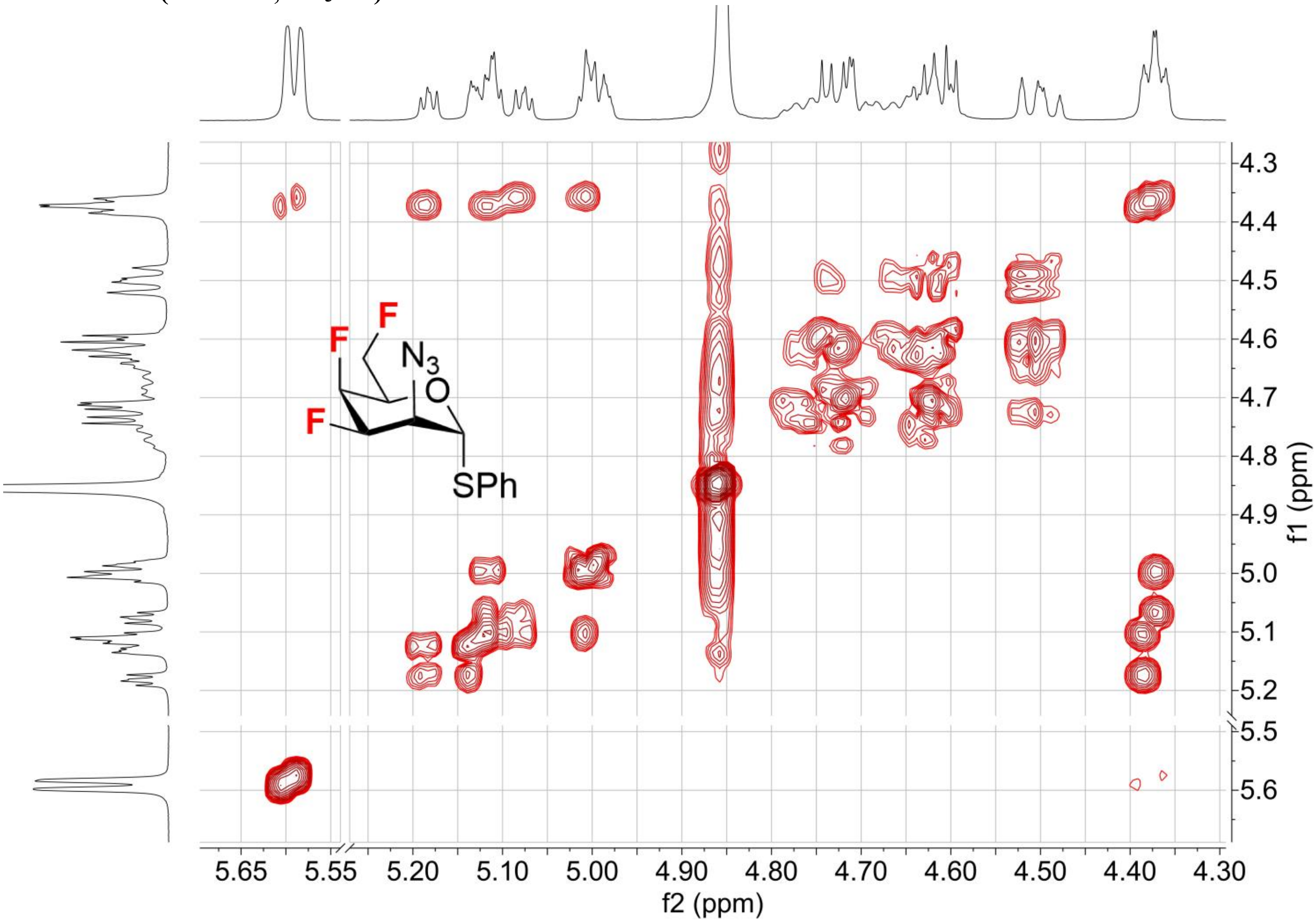

^{1}H-^{13}C HSQC (^{1}H/^{13}C 400/101 MHz, CD_3OD) 50

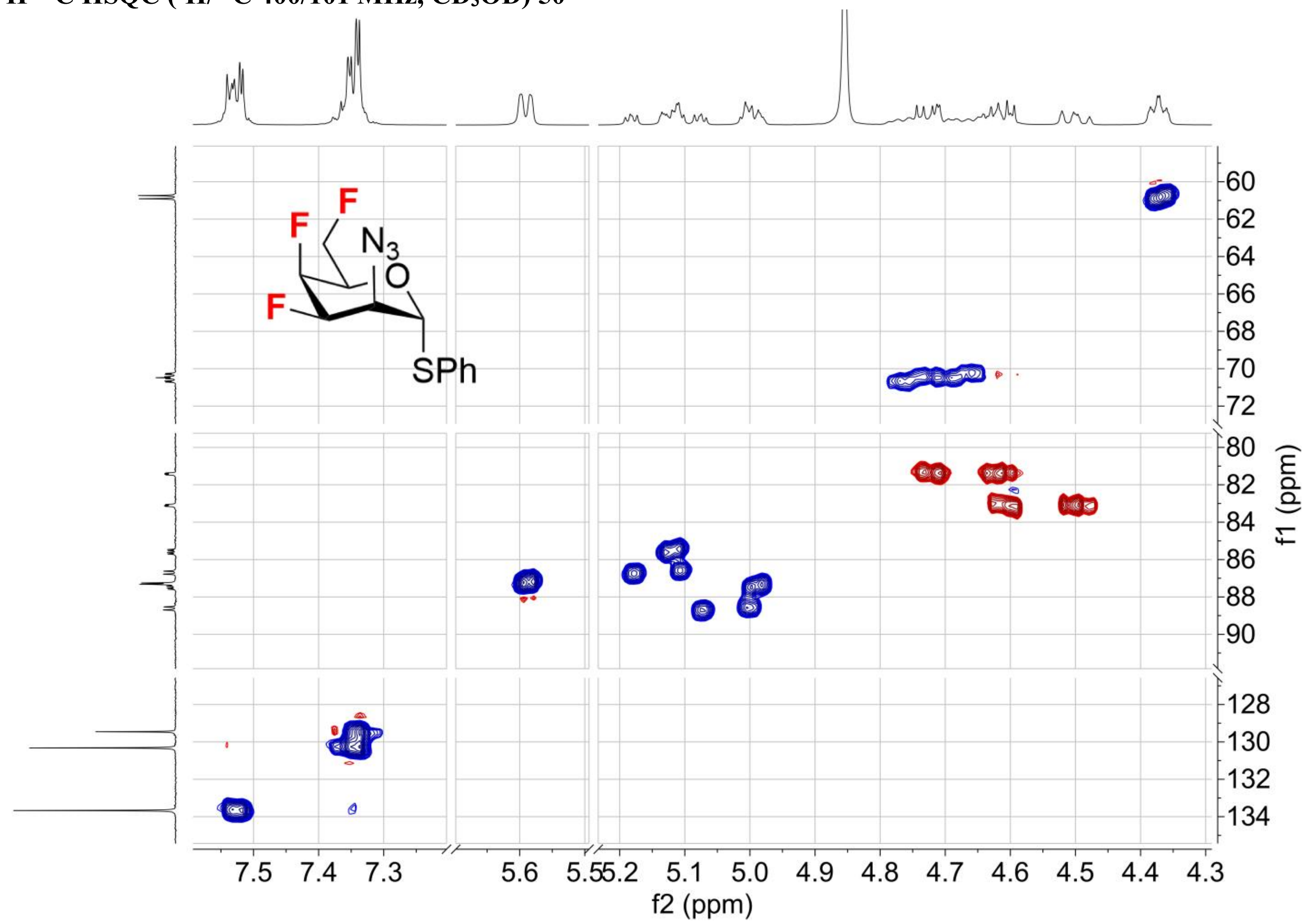

^{1}H-^{13}C HMBC (^{1}H/^{13}C 400/101 MHz, CD_3OD) 50

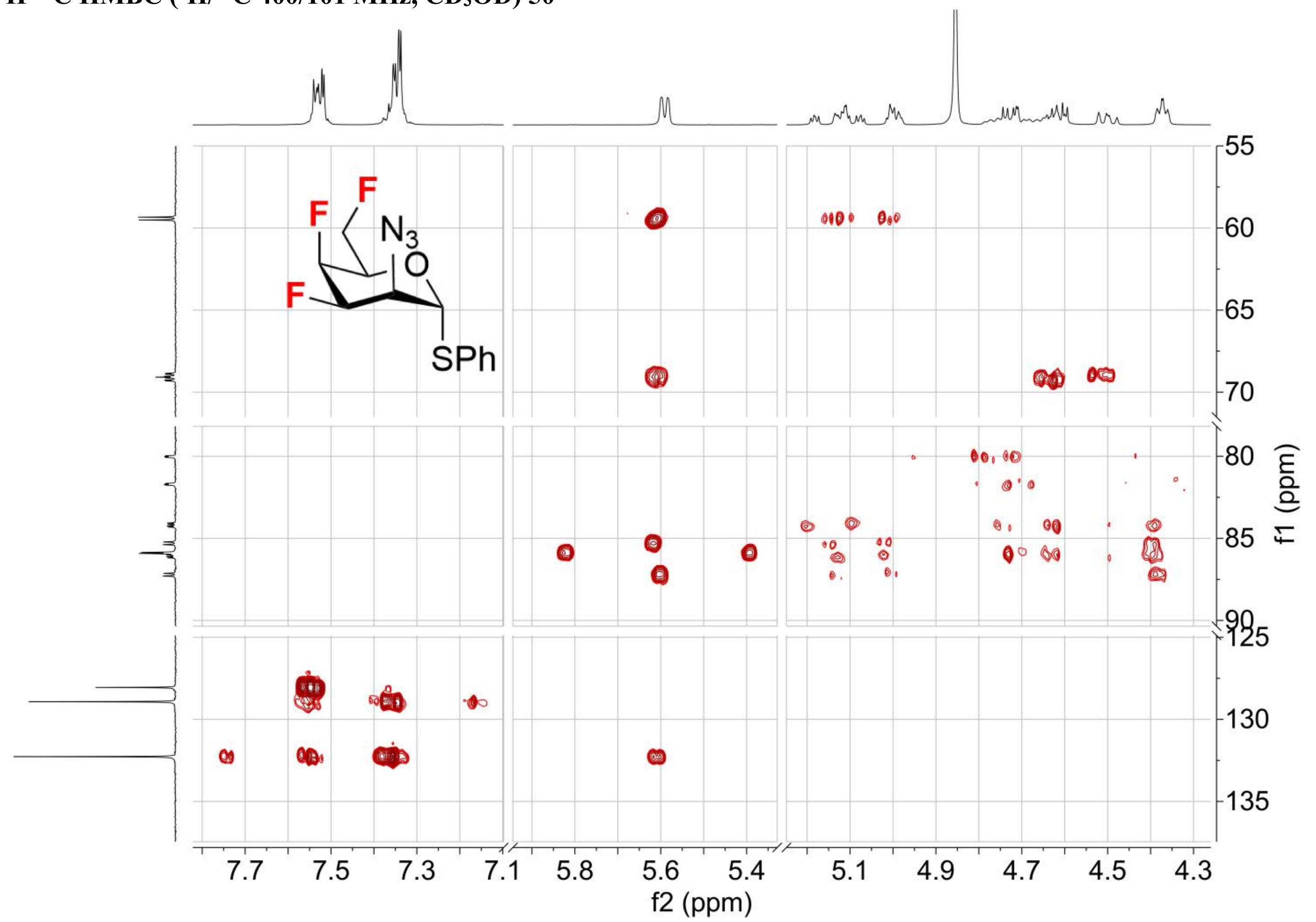

NMR COMPOUND 51

^{1}H NMR (400 MHz, CD_3OD) 51

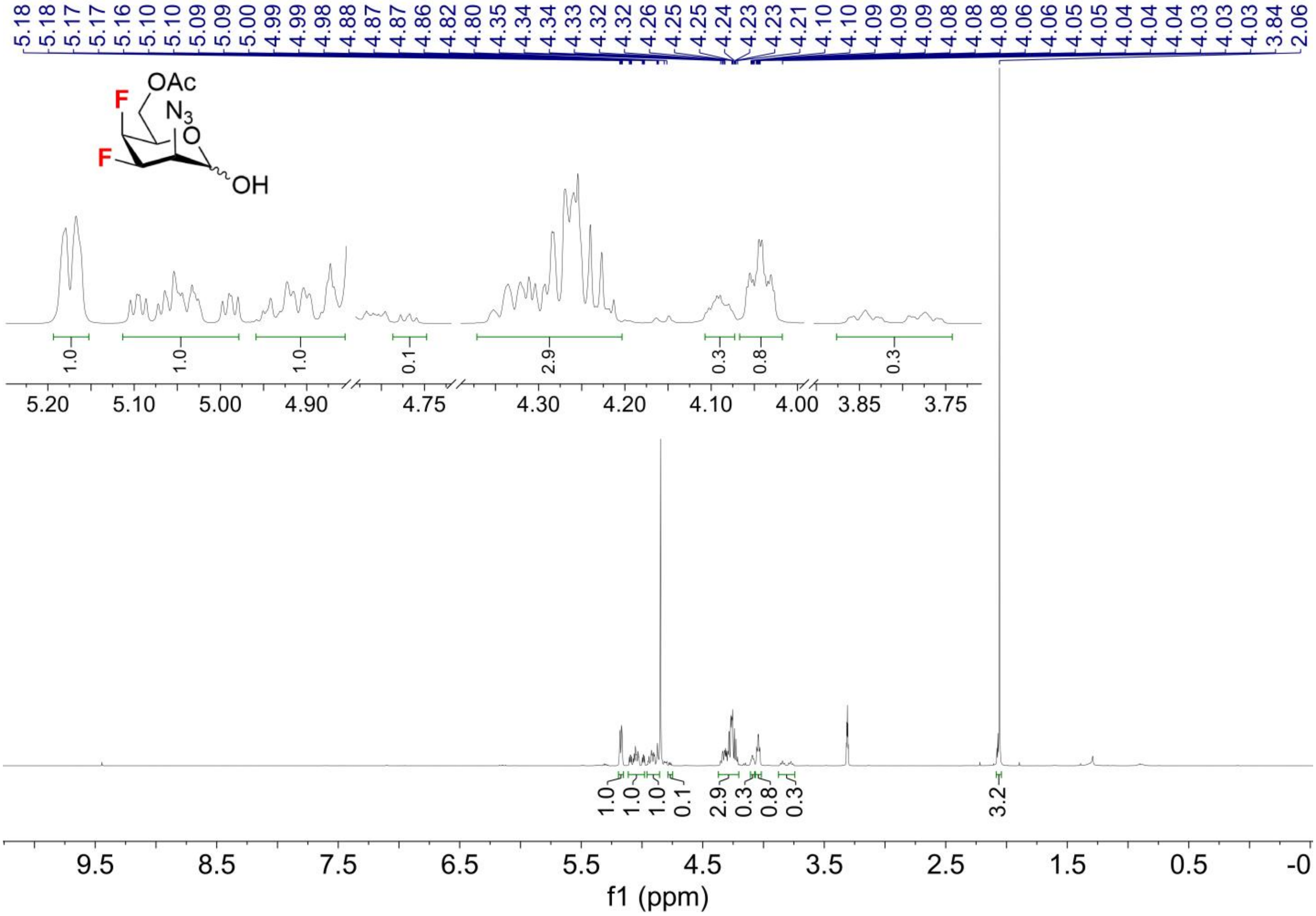

^{13}C{^{1}H} NMR (101 MHz, CD_3OD) 51

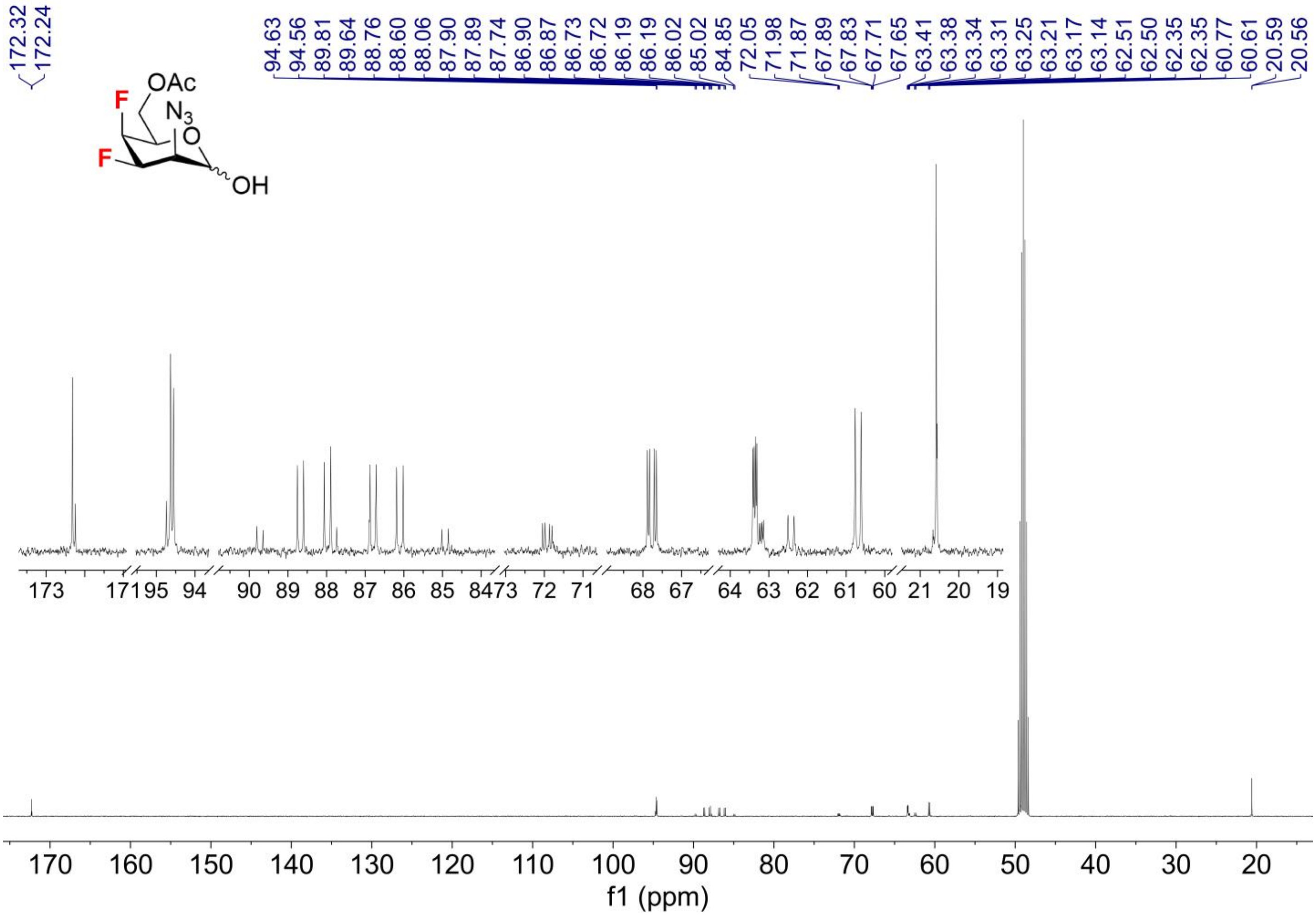

^{19}F NMR (376 MHz, CD_3OD) 51

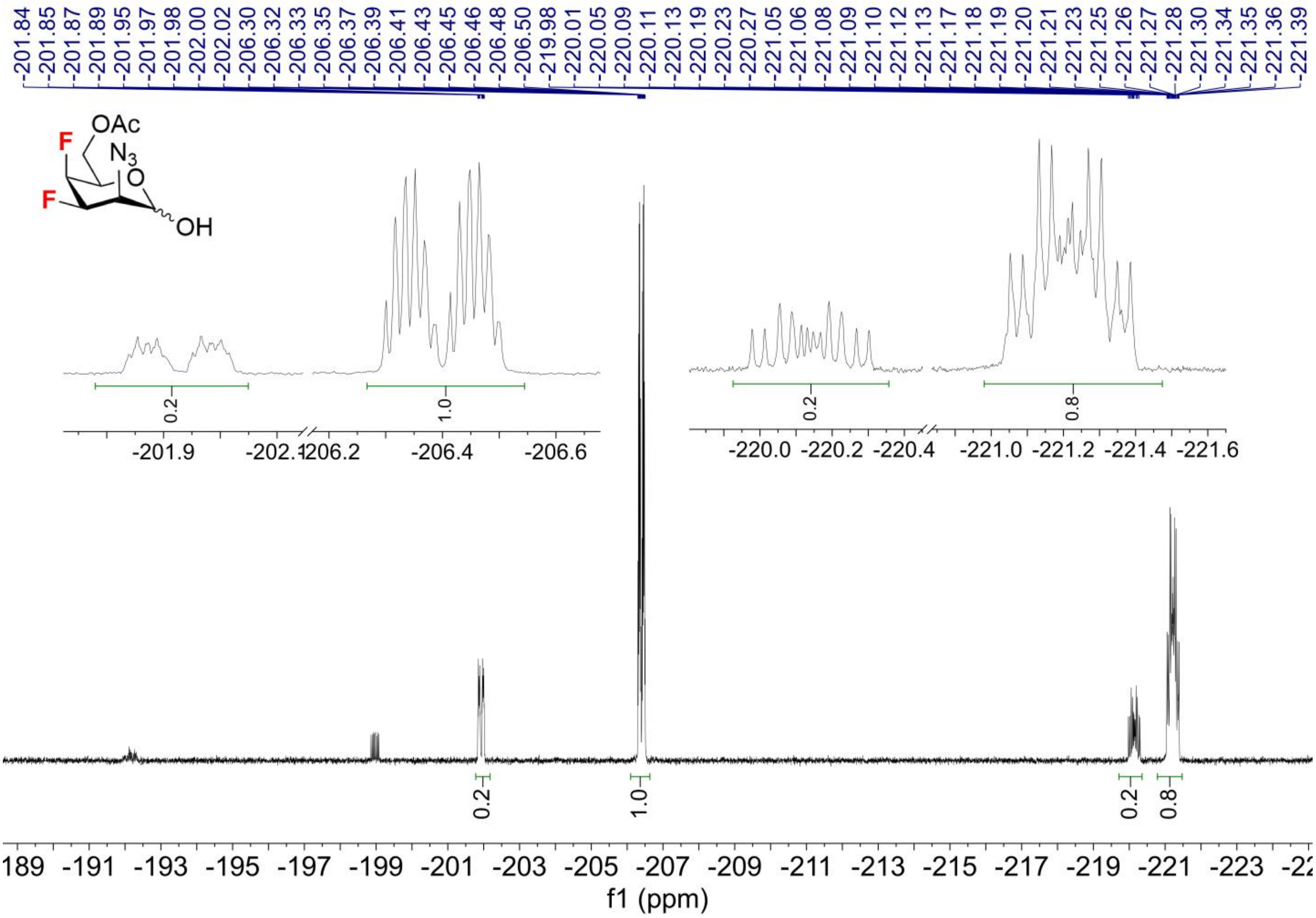

^{1}H-^{1}H COSY (400 MHz, CD_3OD) 51

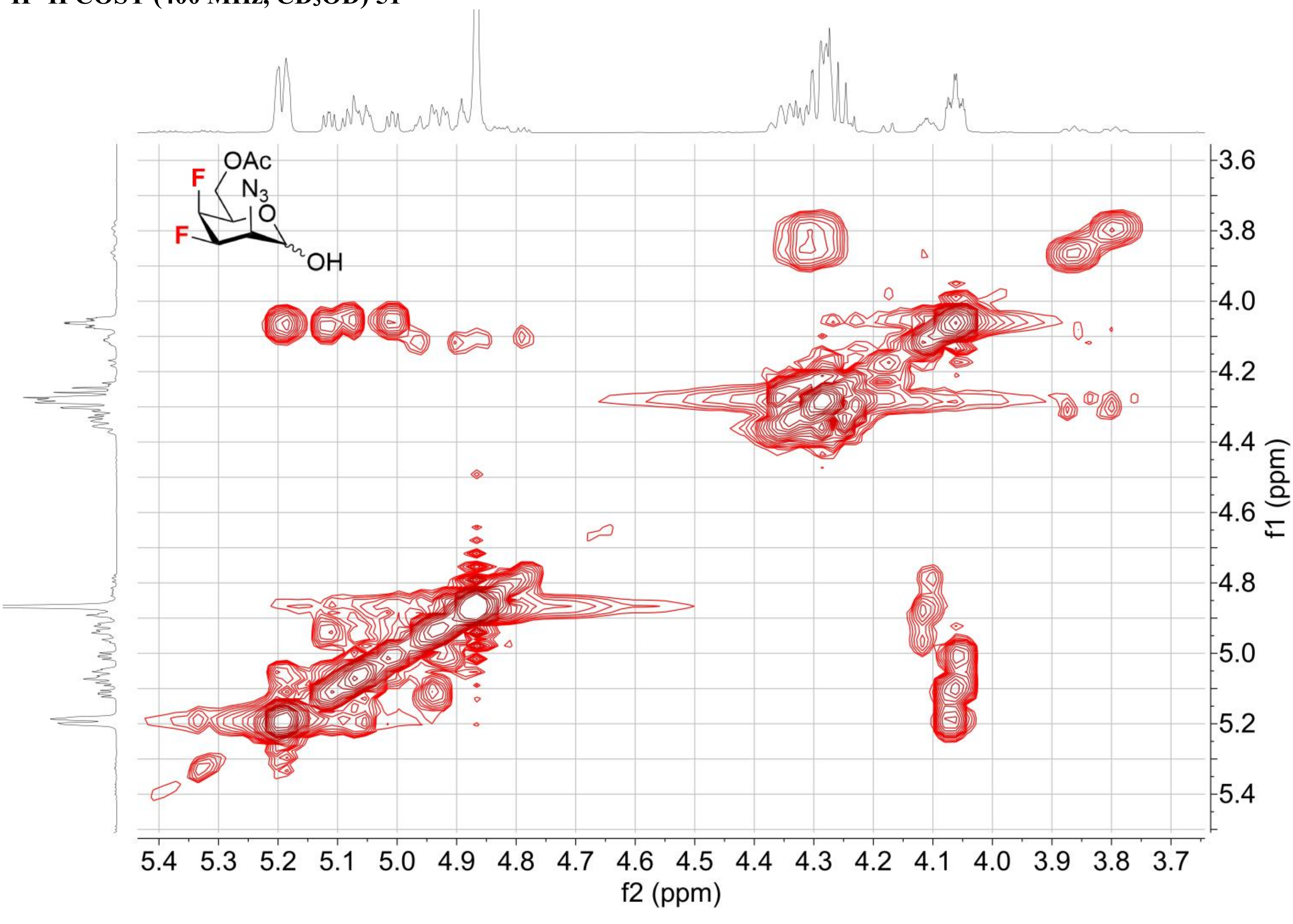

^{1}H-^{13}C HSQC ($^{1}H/^{13}C$ 400/101 MHz, CD_3OD) 51

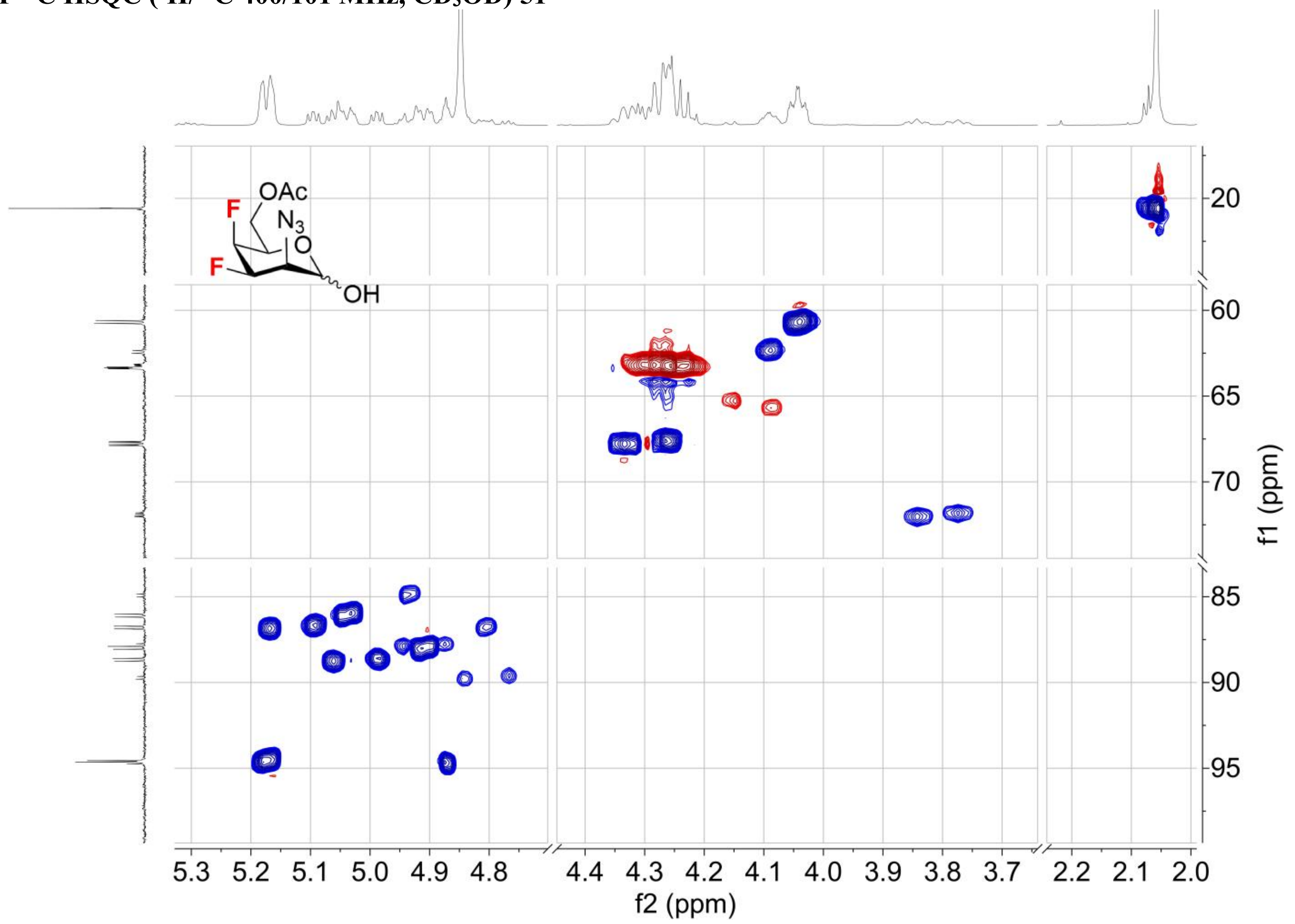

^{1}H-^{13}C HMBC ($^{1}H/^{13}C$ 400/101 MHz, CD_3OD) 51

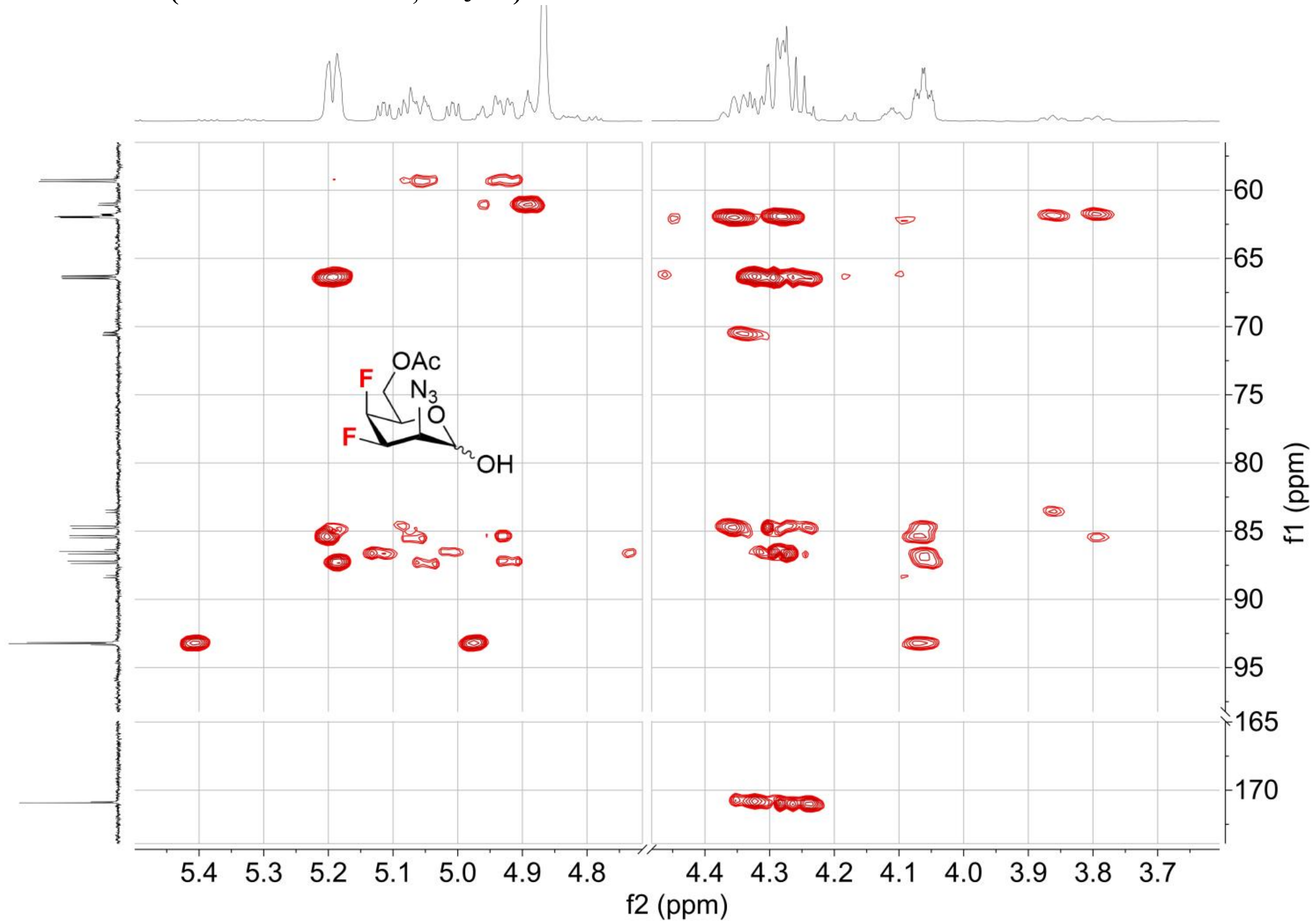

NMR COMPOUND 53

^{1}H NMR (400 MHz, $CDCl_3$) 53

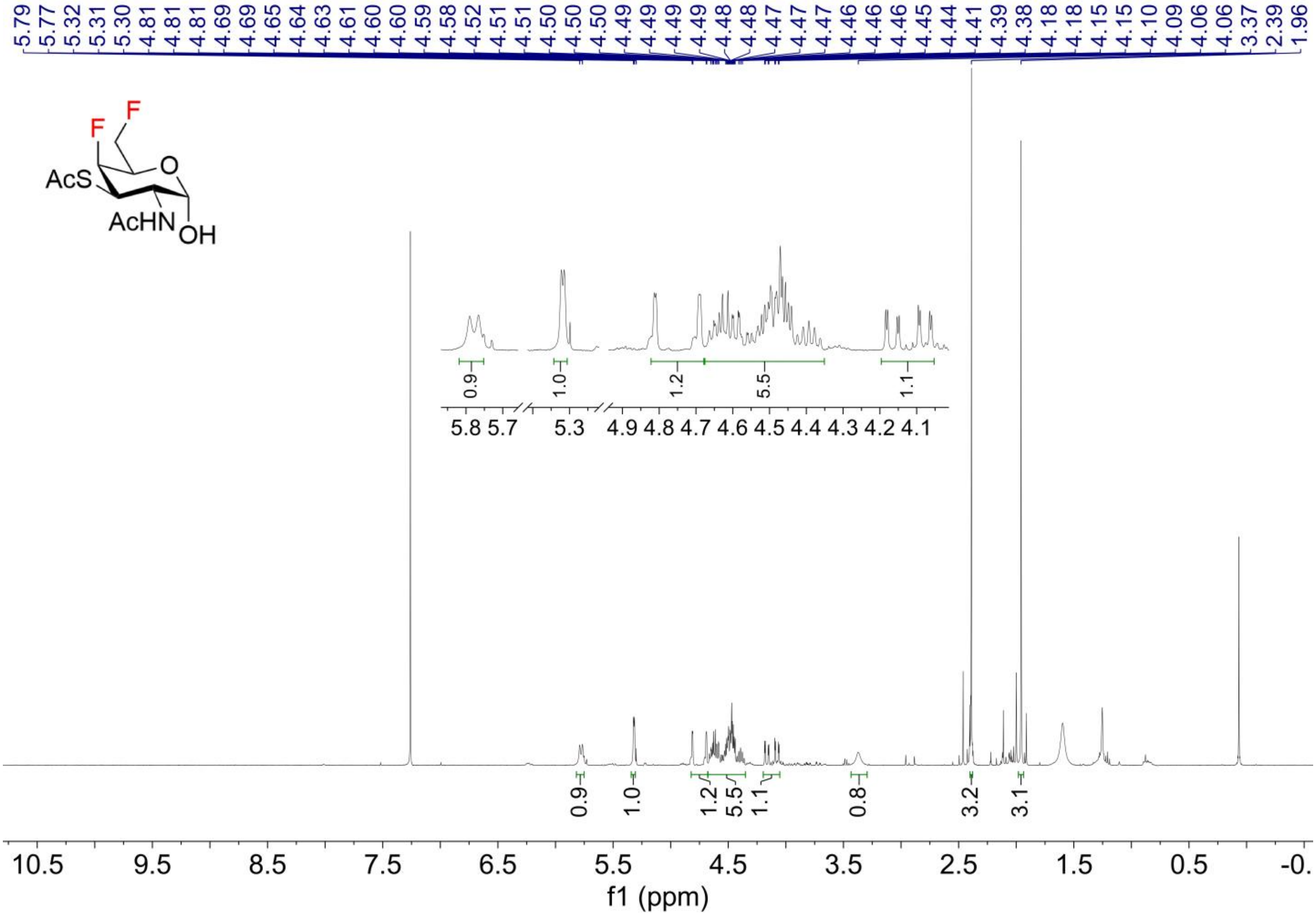

$^{13}C\{^1H\}$ NMR (101 MHz, $CDCl_3$) 53

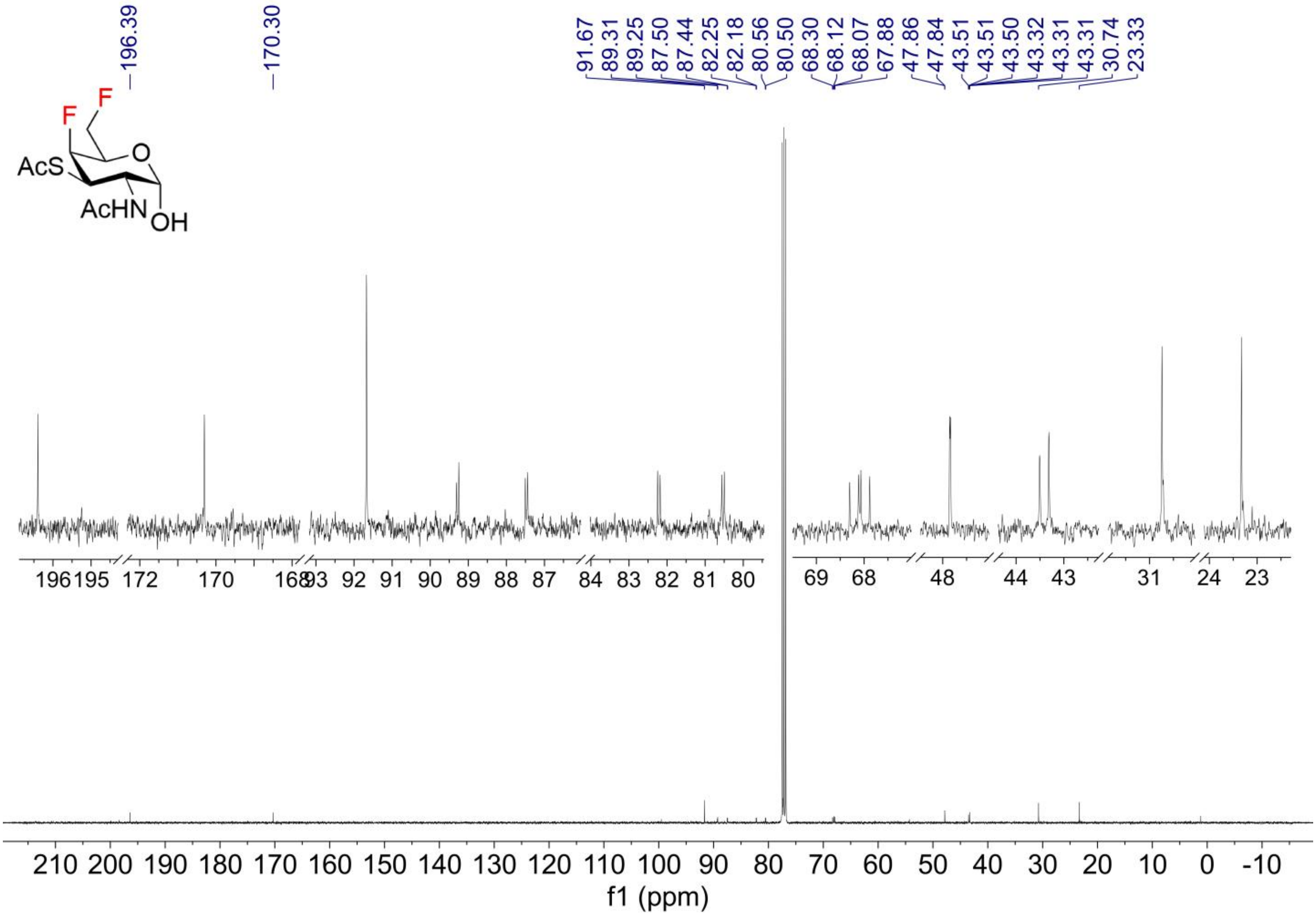

^{19}F NMR (376 MHz, CDCl$_3$) 53

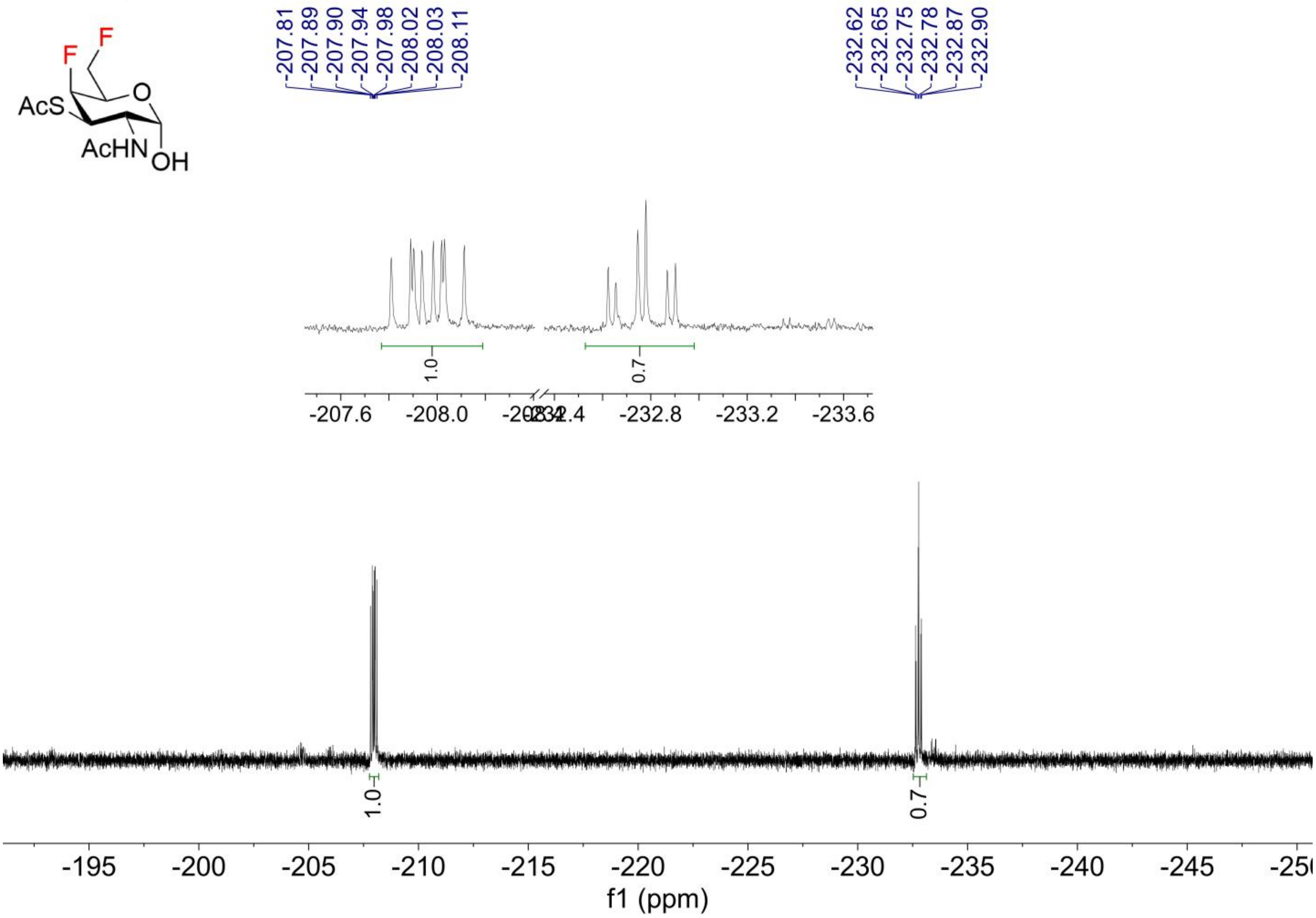

^{1}H-^{1}H COSY (400 MHz, CDCl$_3$) 53

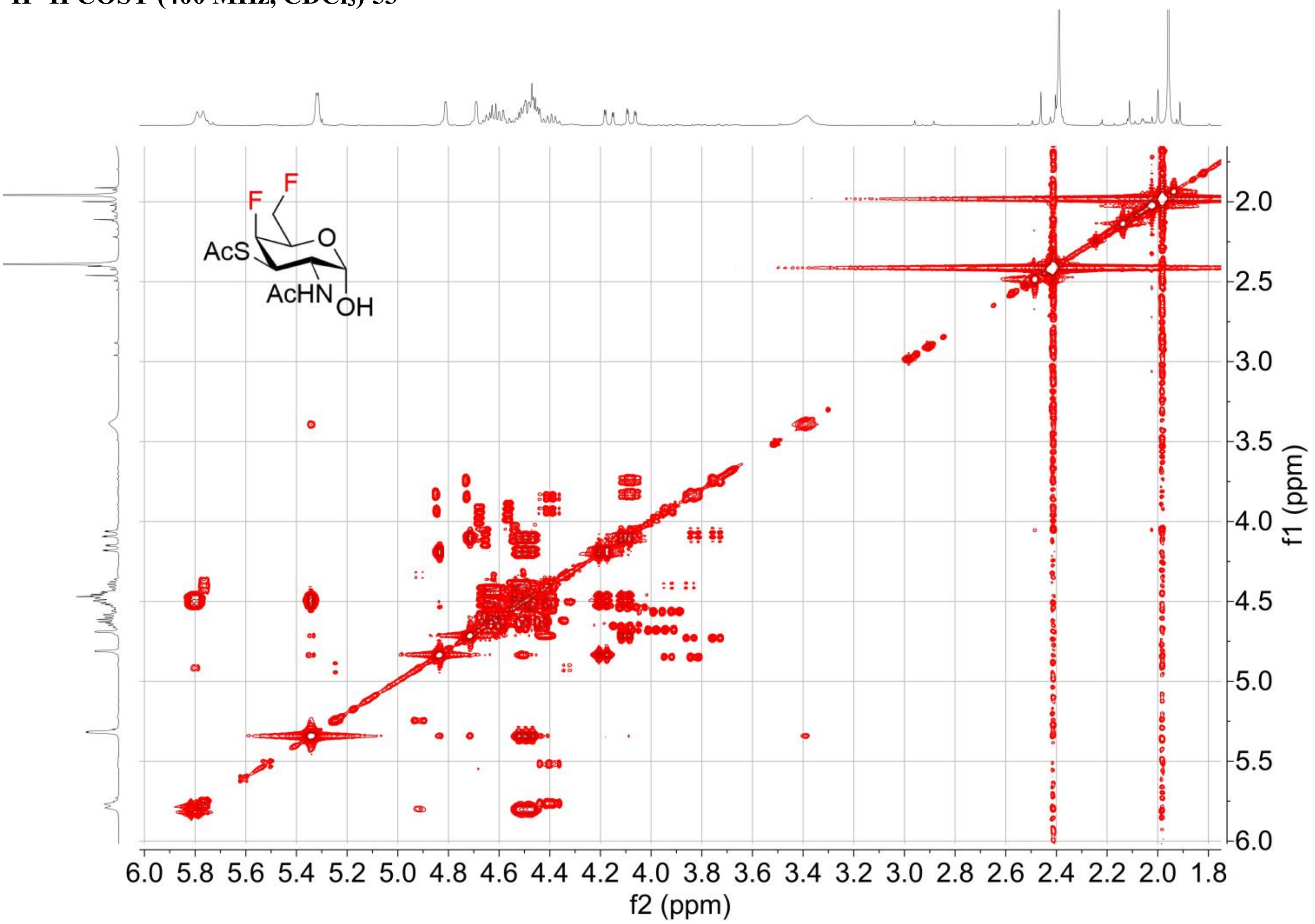

^{1}H-^{13}C HSQC (^{1}H/^{13}C 400/101 MHz, CDCl$_3$) 53

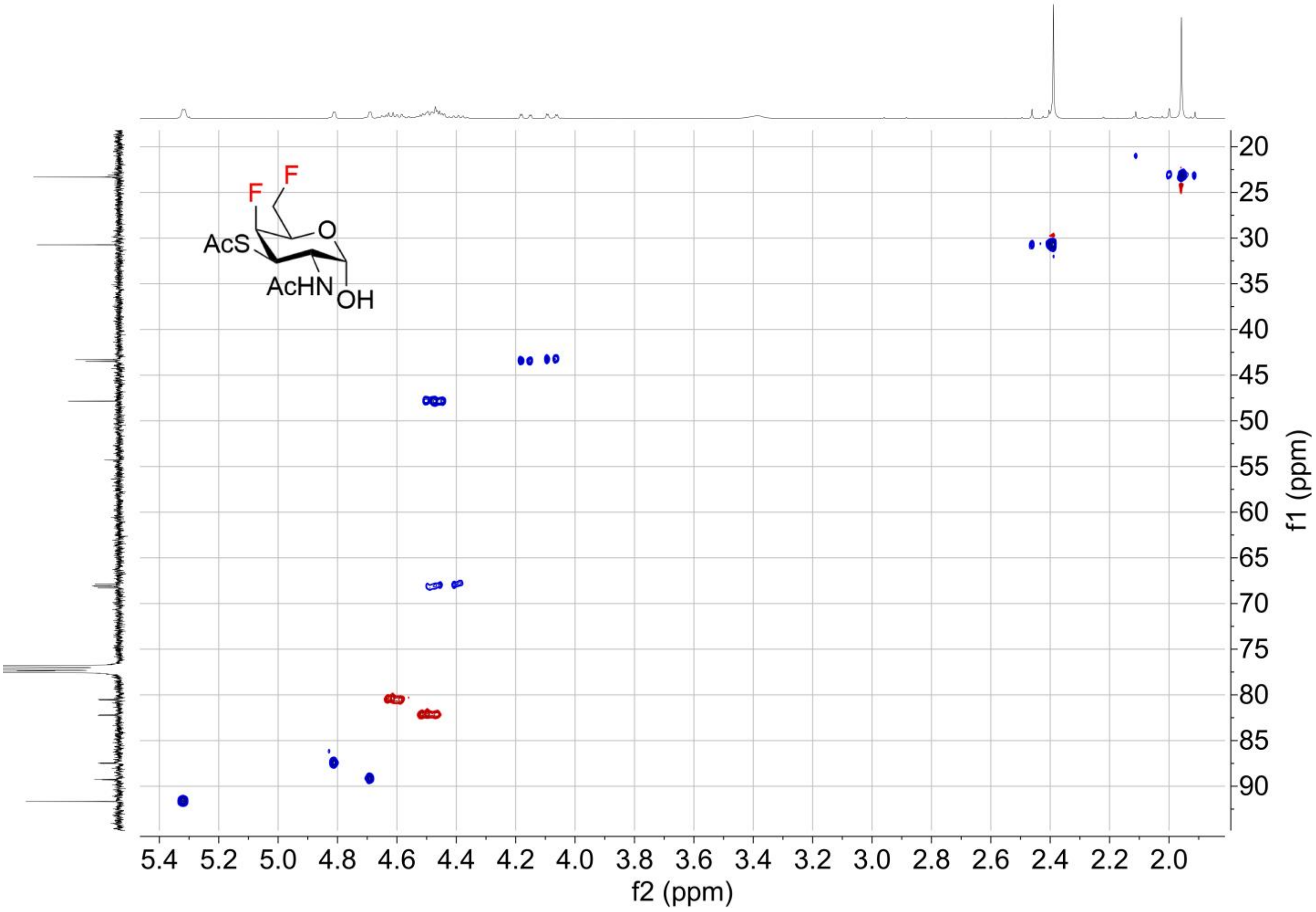

NMR COMPOUND 55

^{1}H NMR (400 MHz, $CDCl_3$) 55

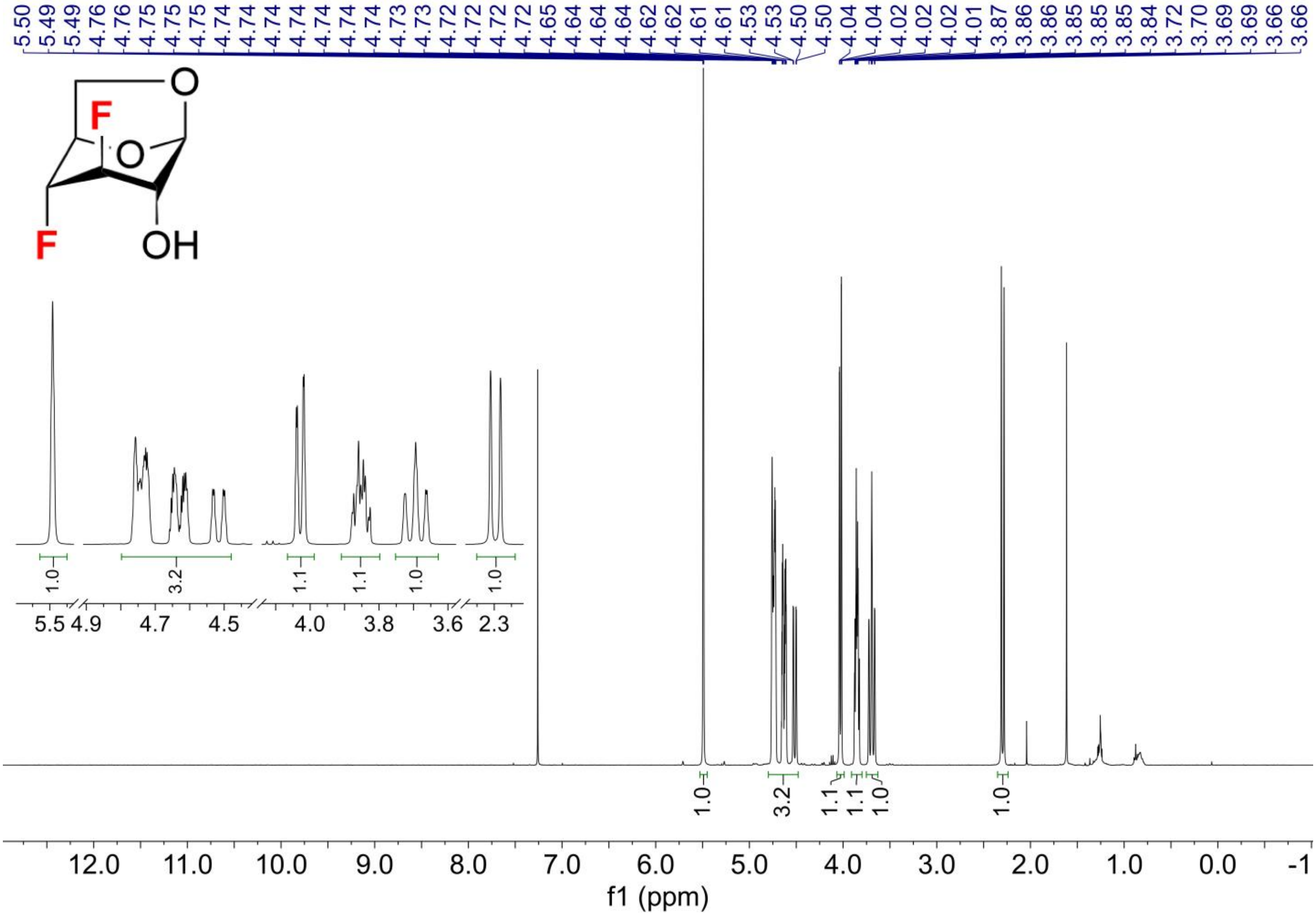

^{13}C{^{1}H} NMR (101 MHz, $CDCl_3$) 55

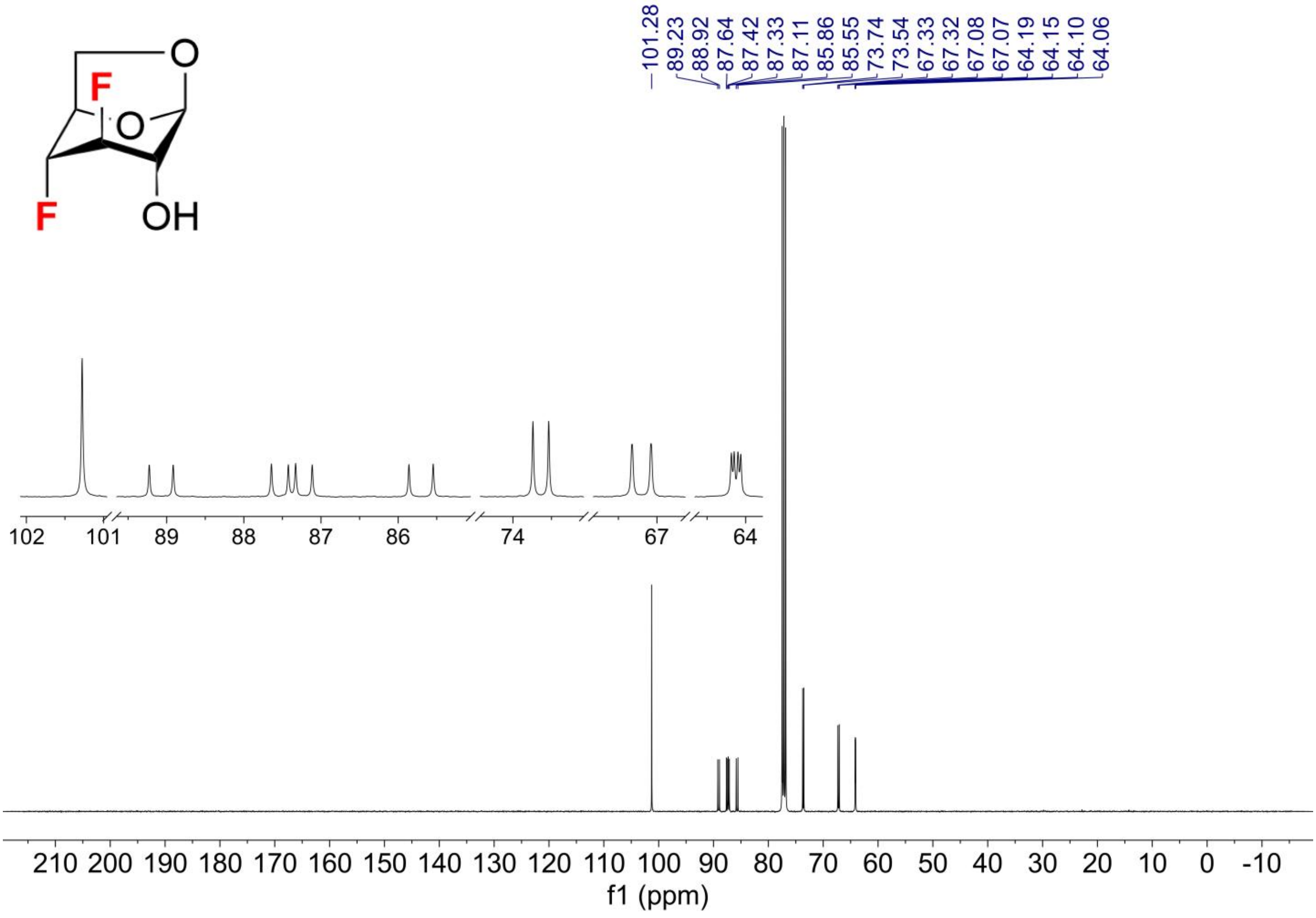

^{19}F NMR (376 MHz, CDCl$_3$) 55

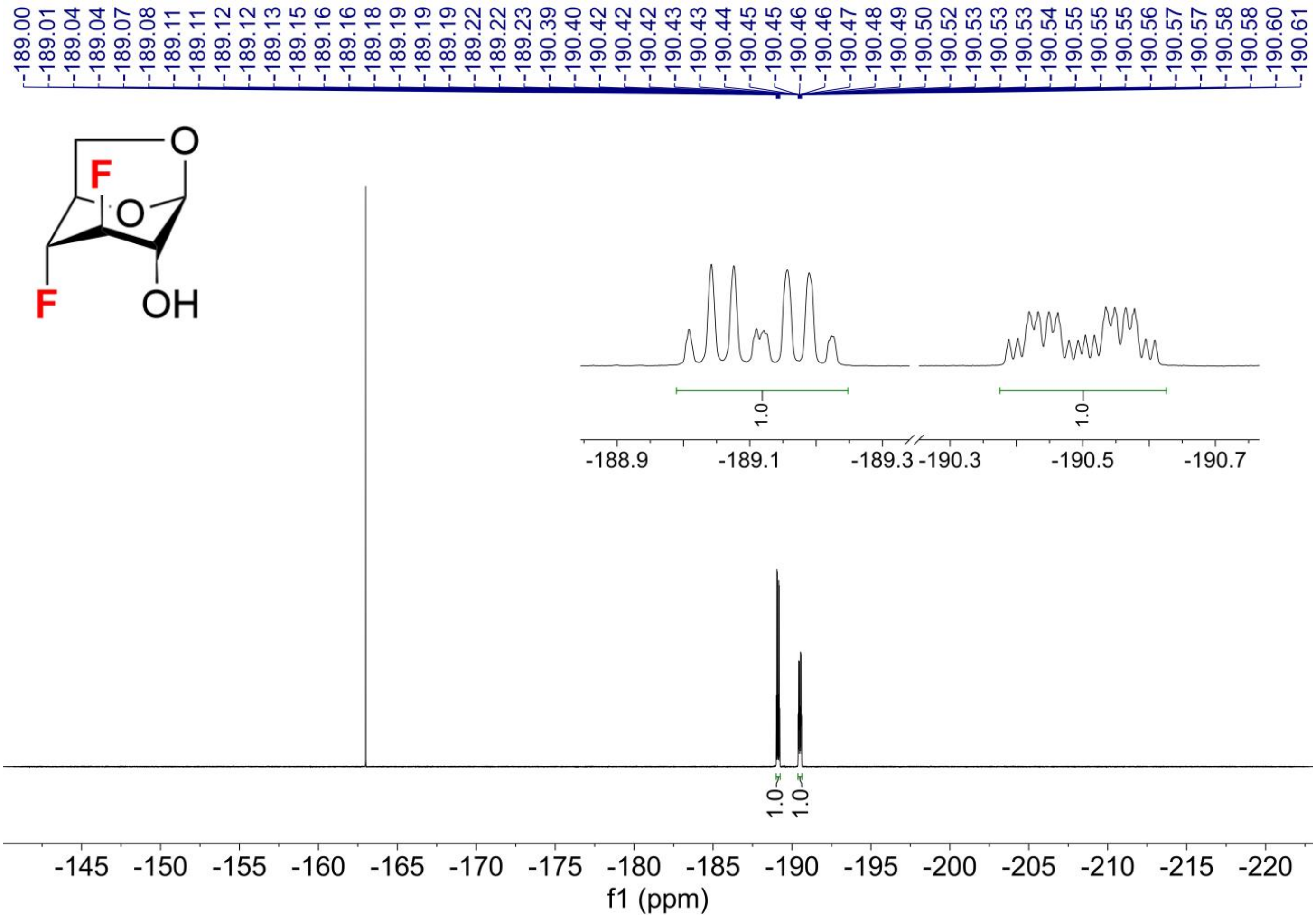

^{1}H-^{1}H COSY (400 MHz, CDCl$_3$) 55

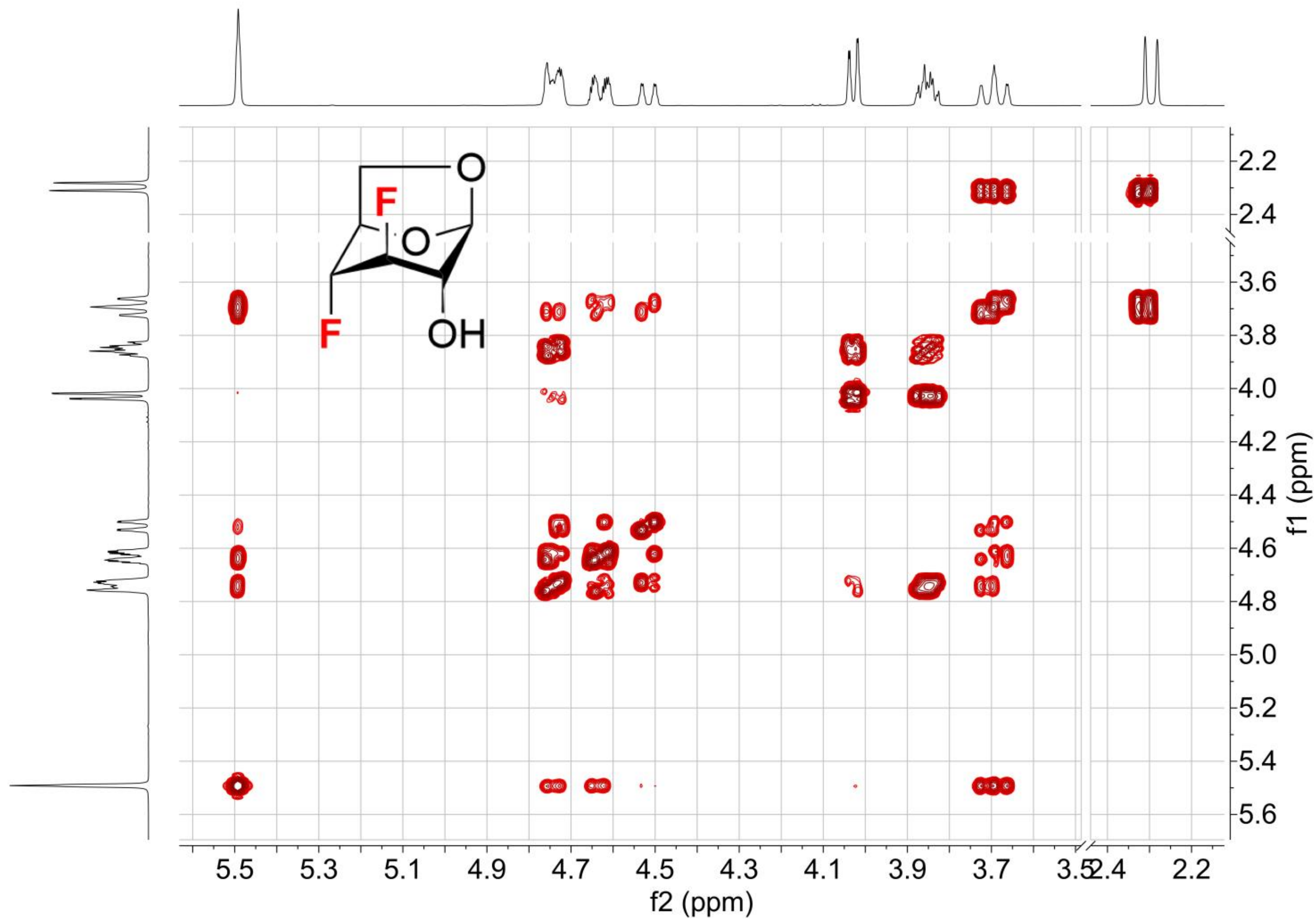

^{1}H-^{13}C HSQC (^{1}H/^{13}C 400/101 MHz, CDCl$_3$) 55

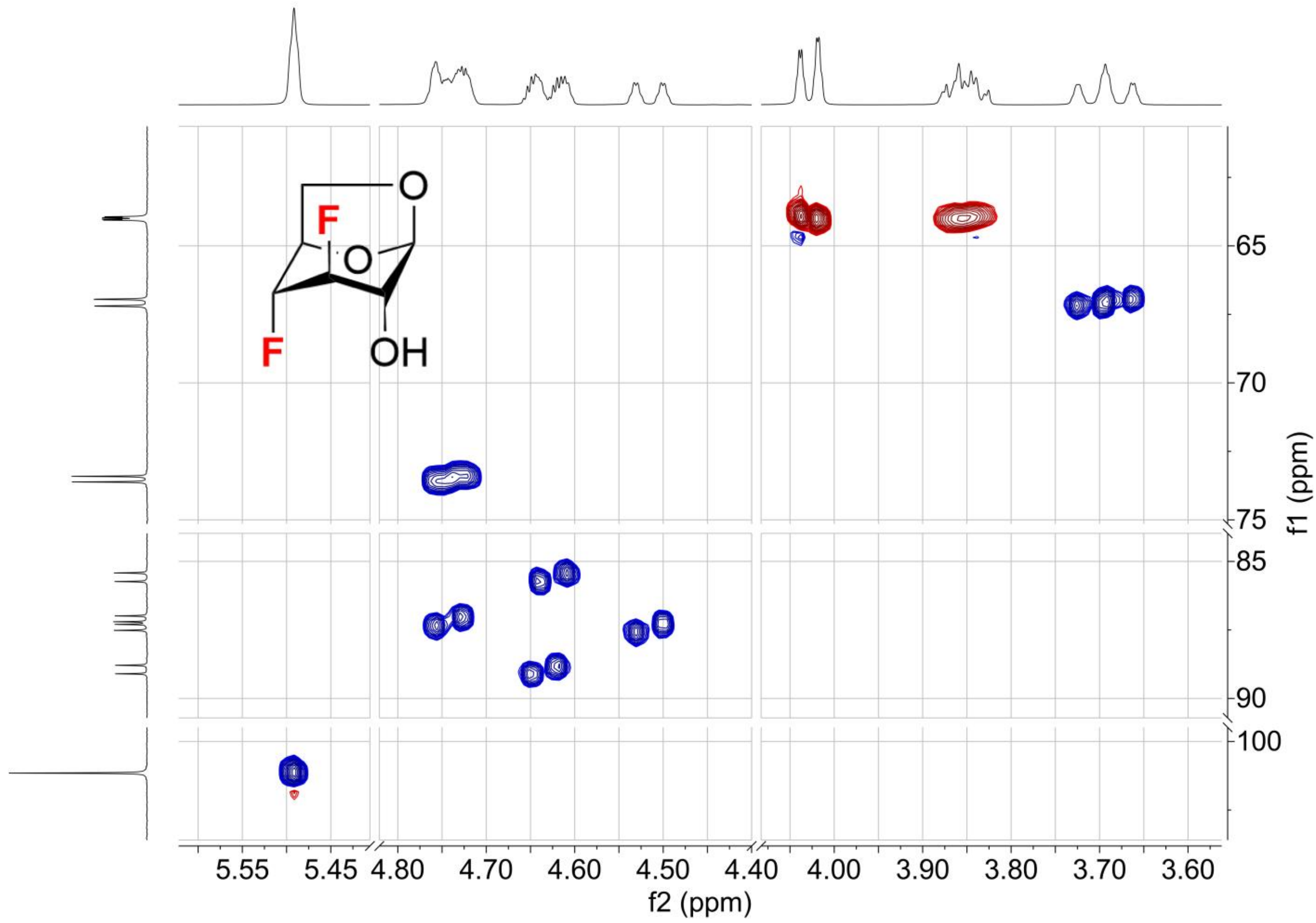

^{1}H-^{13}C HMBC (^{1}H/^{13}C 400/101 MHz, CDCl$_3$) 55

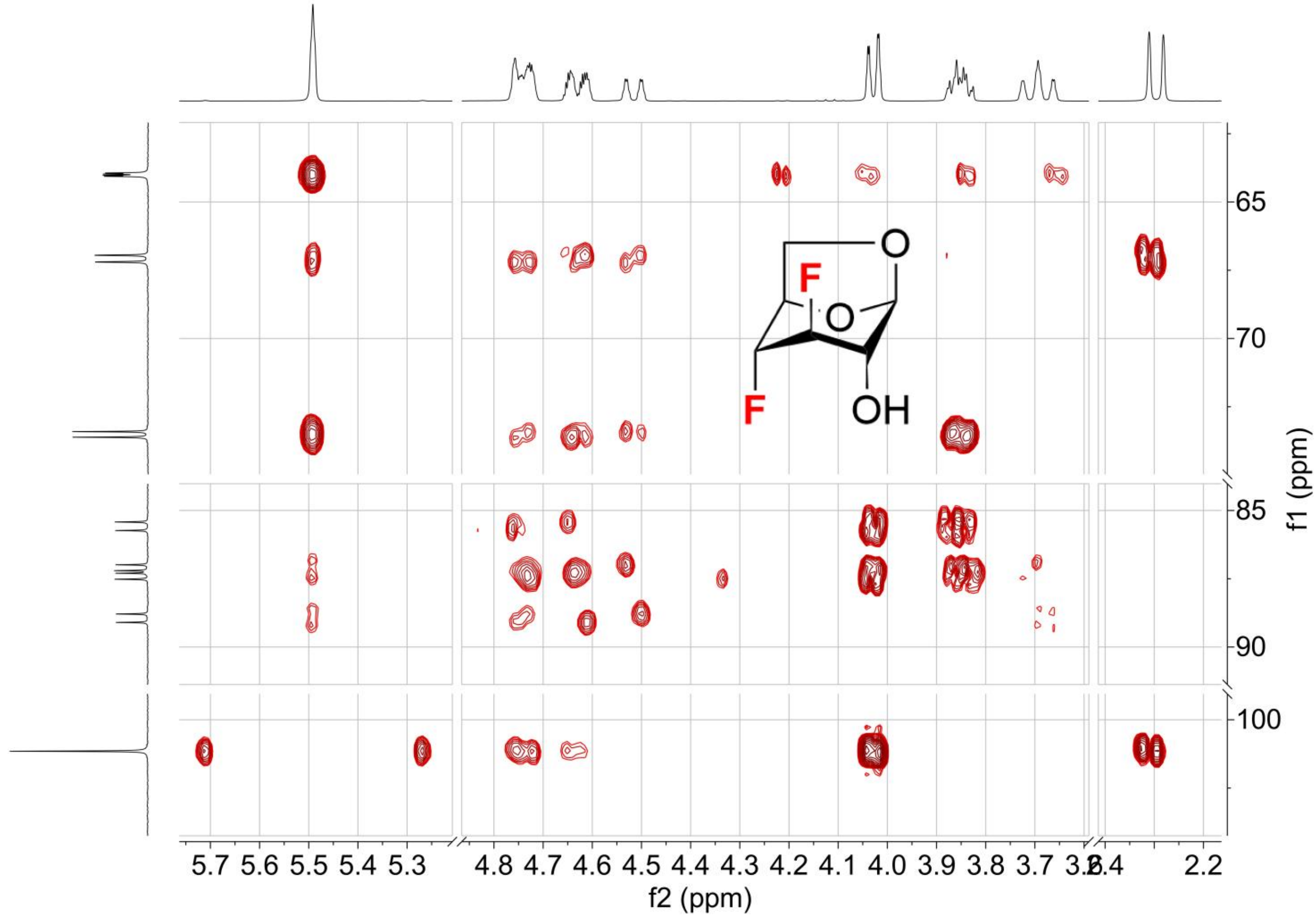

NMR COMPOUND 56

^{1}H NMR (400 MHz, CDCl$_3$) 56

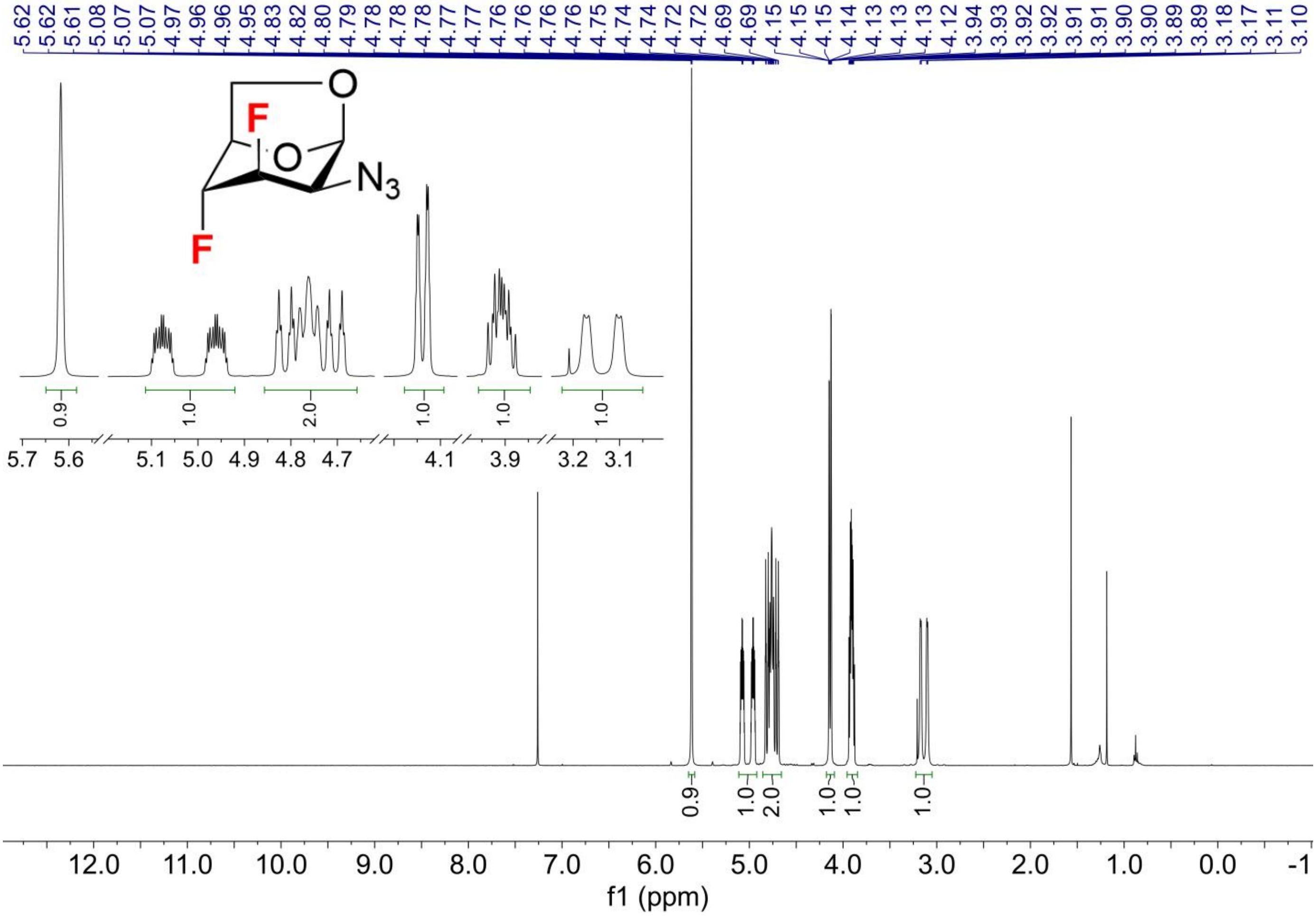

^{13}C{^{1}H} NMR (101 MHz, CDCl$_3$) 56

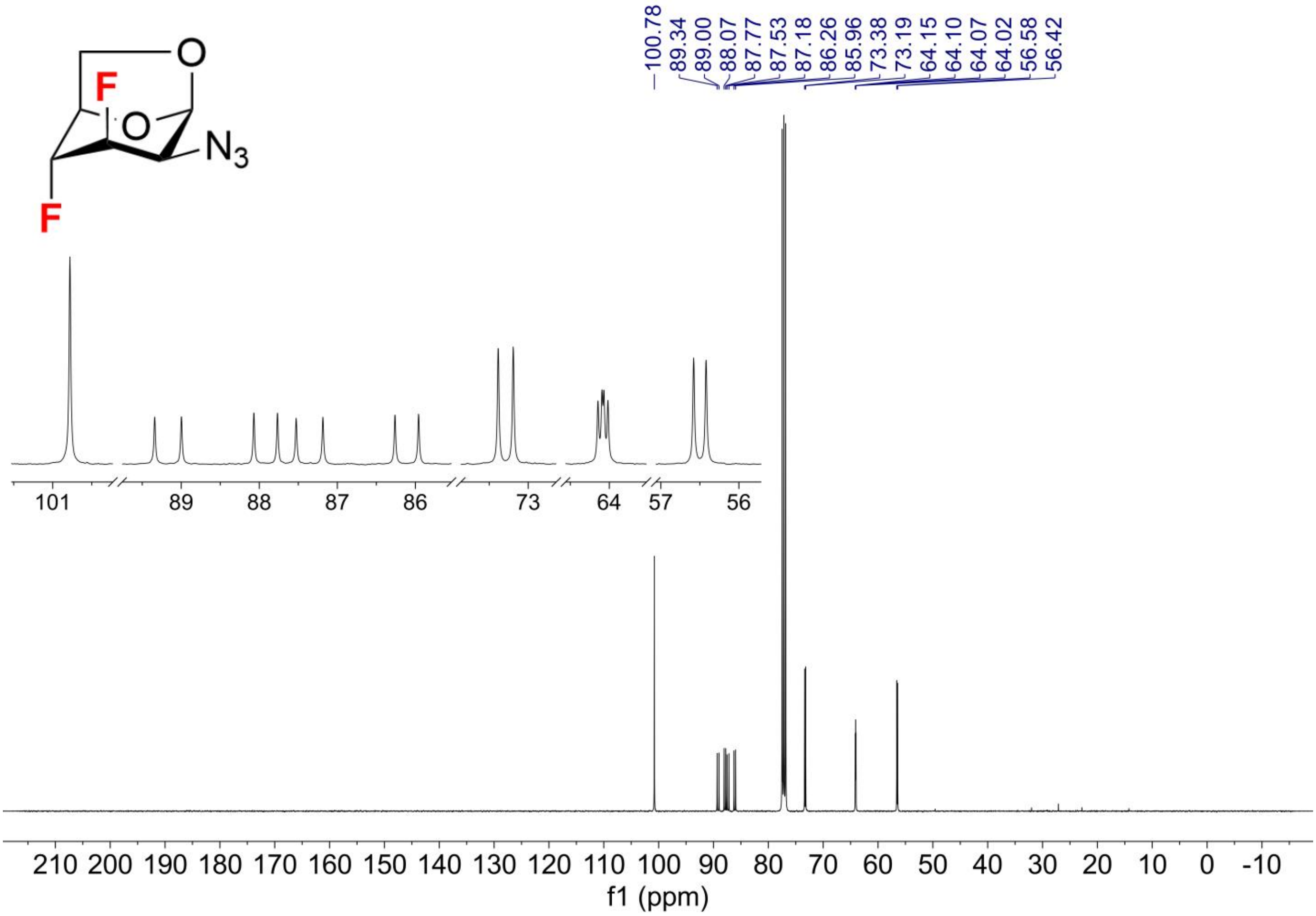

^{19}F NMR (376 MHz, CDCl$_3$) 56

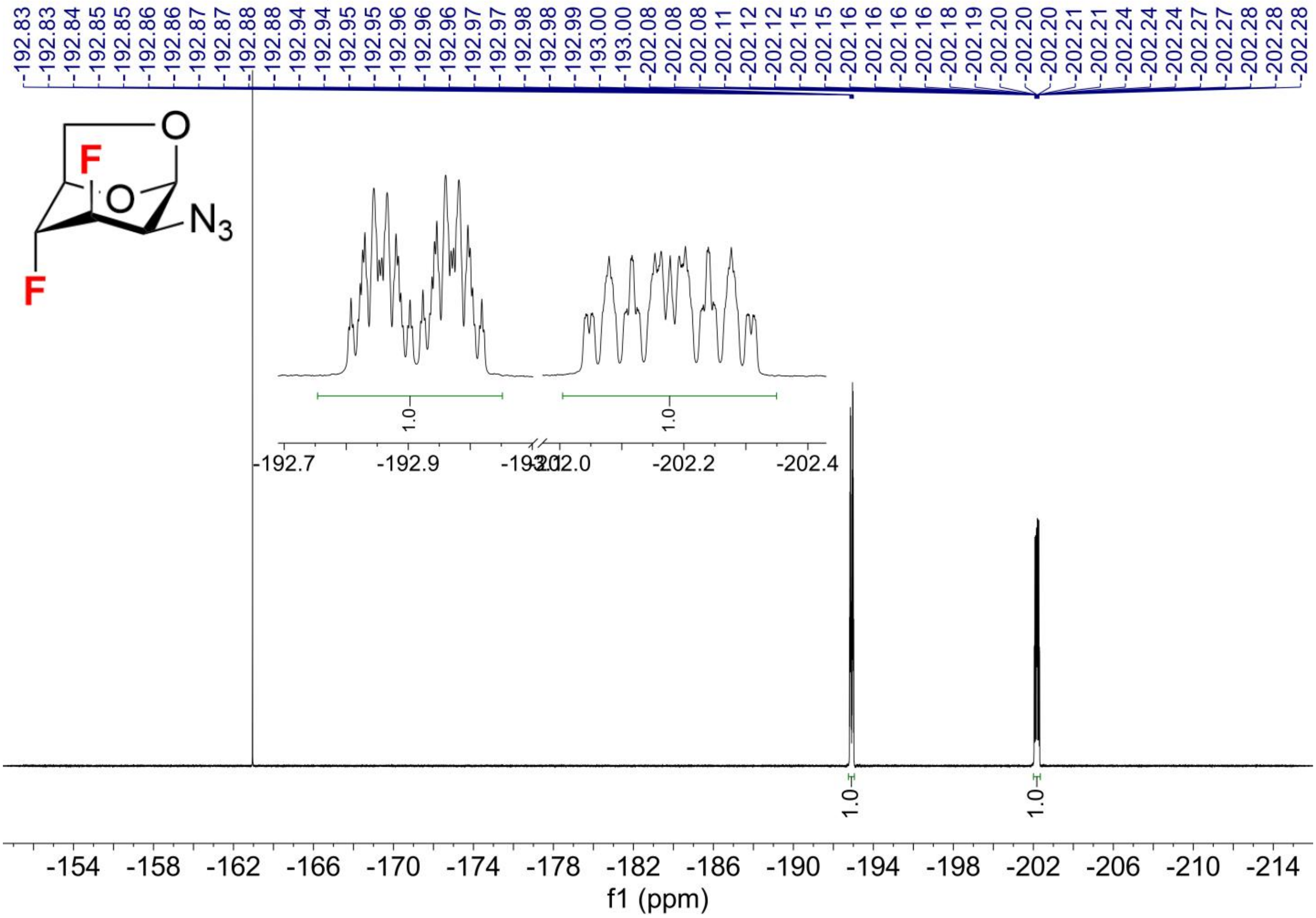

^{1}H-^{1}H COSY (400 MHz, CDCl$_3$) 56

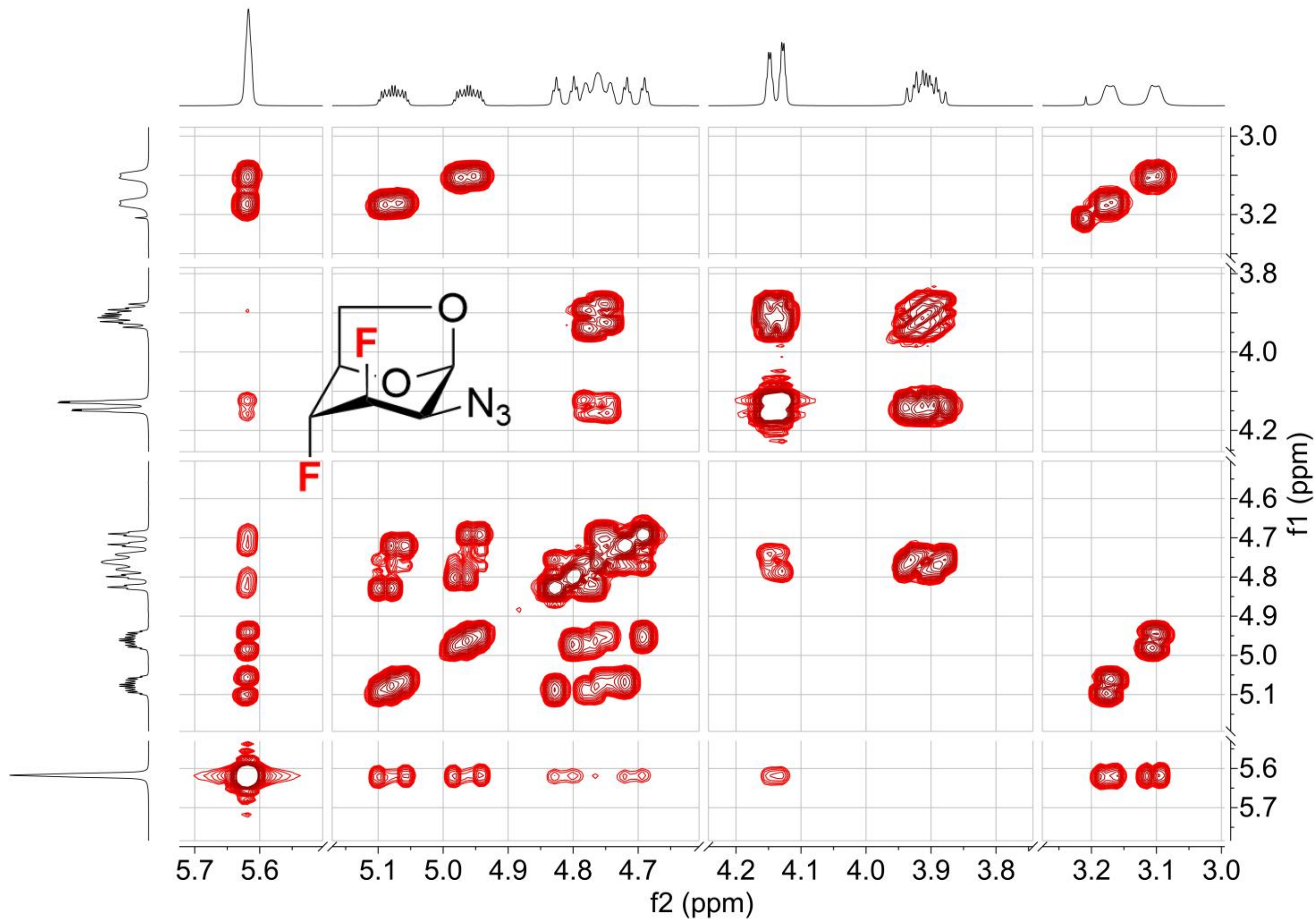

^1H-^{13}C HSQC (^1H/^{13}C 400/101 MHz, $CDCl_3$) 56

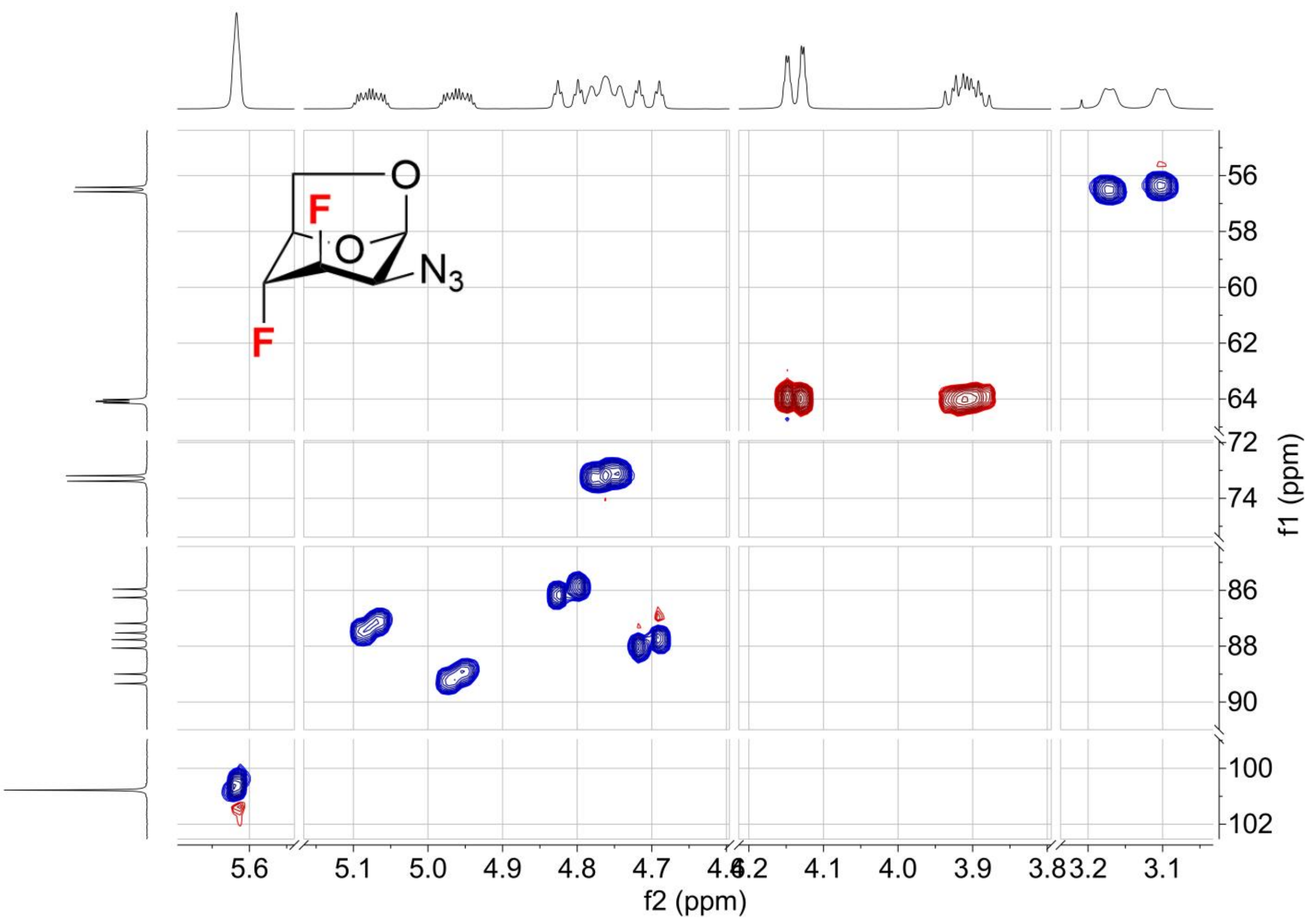

^1H-^{13}C HMBC (^1H/^{13}C 400/101 MHz, $CDCl_3$) 56

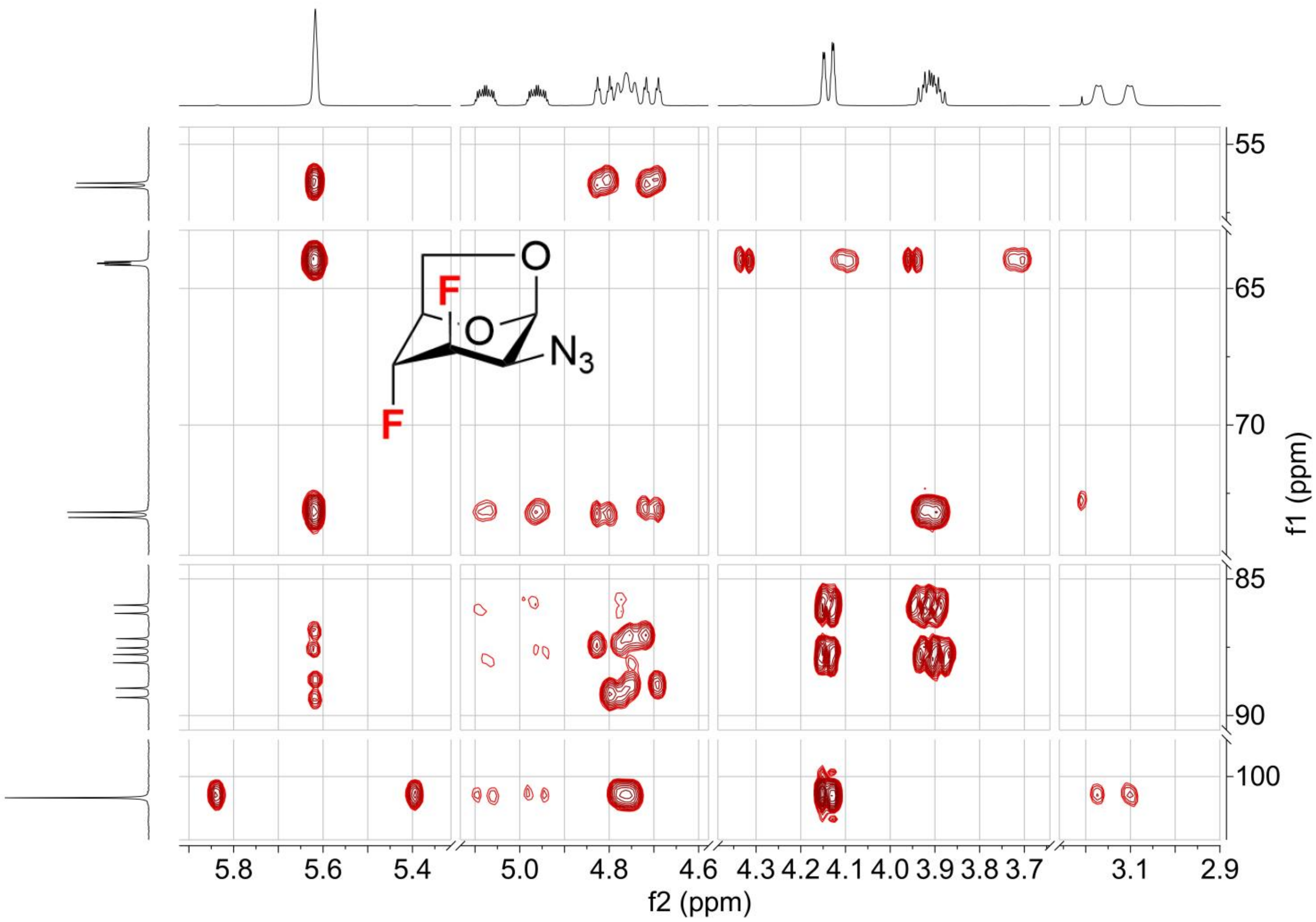

NMR COMPOUND β-57

^{1}H NMR (400 MHz, $CDCl_3$) β-57

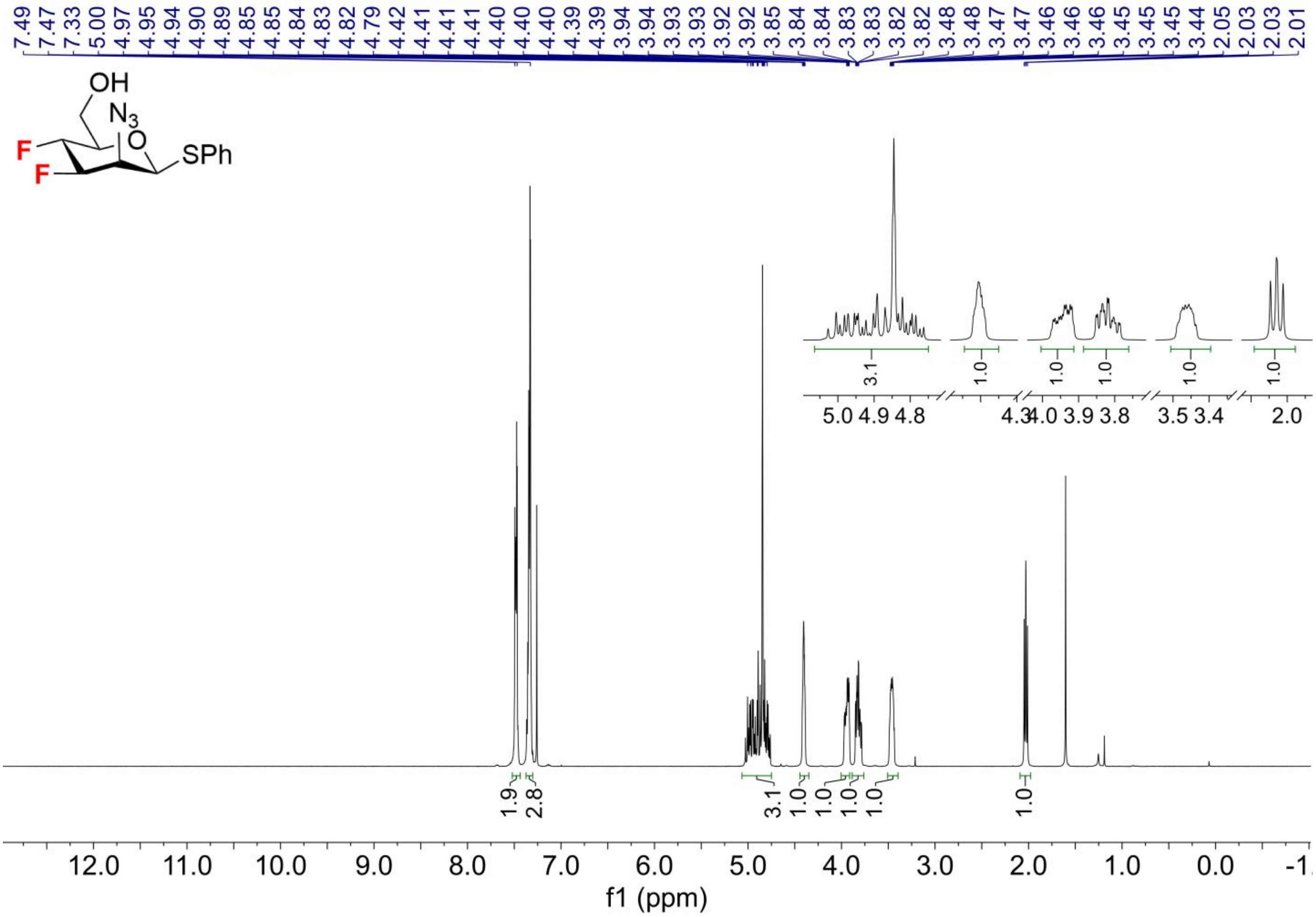

^{13}C{^{1}H} NMR (101 MHz, $CDCl_3$) β-57

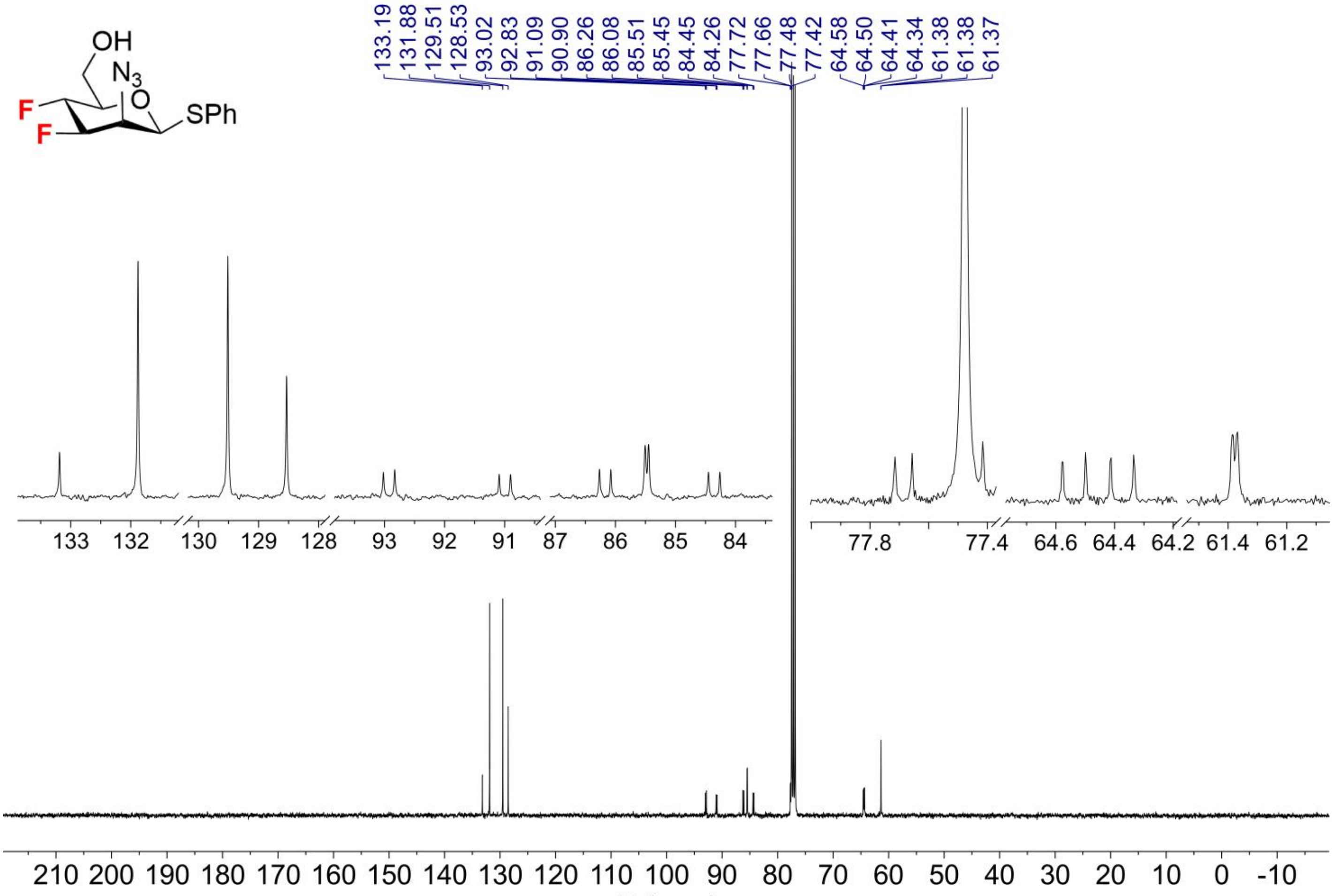

^{19}F NMR (376 MHz, CDCl₃) β-57

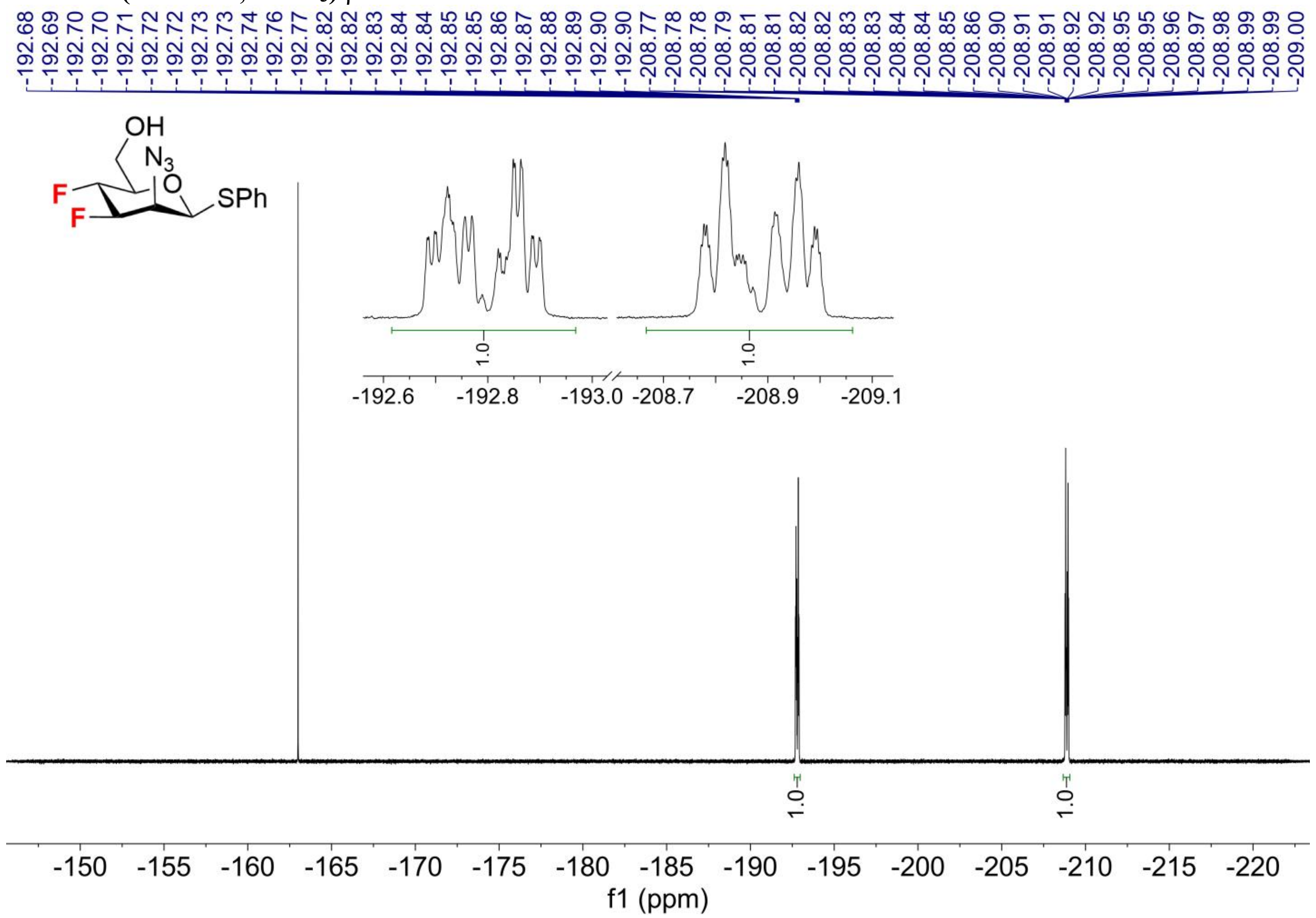

^{1}H-^{1}H COSY (400 MHz, CDCl₃) β-57

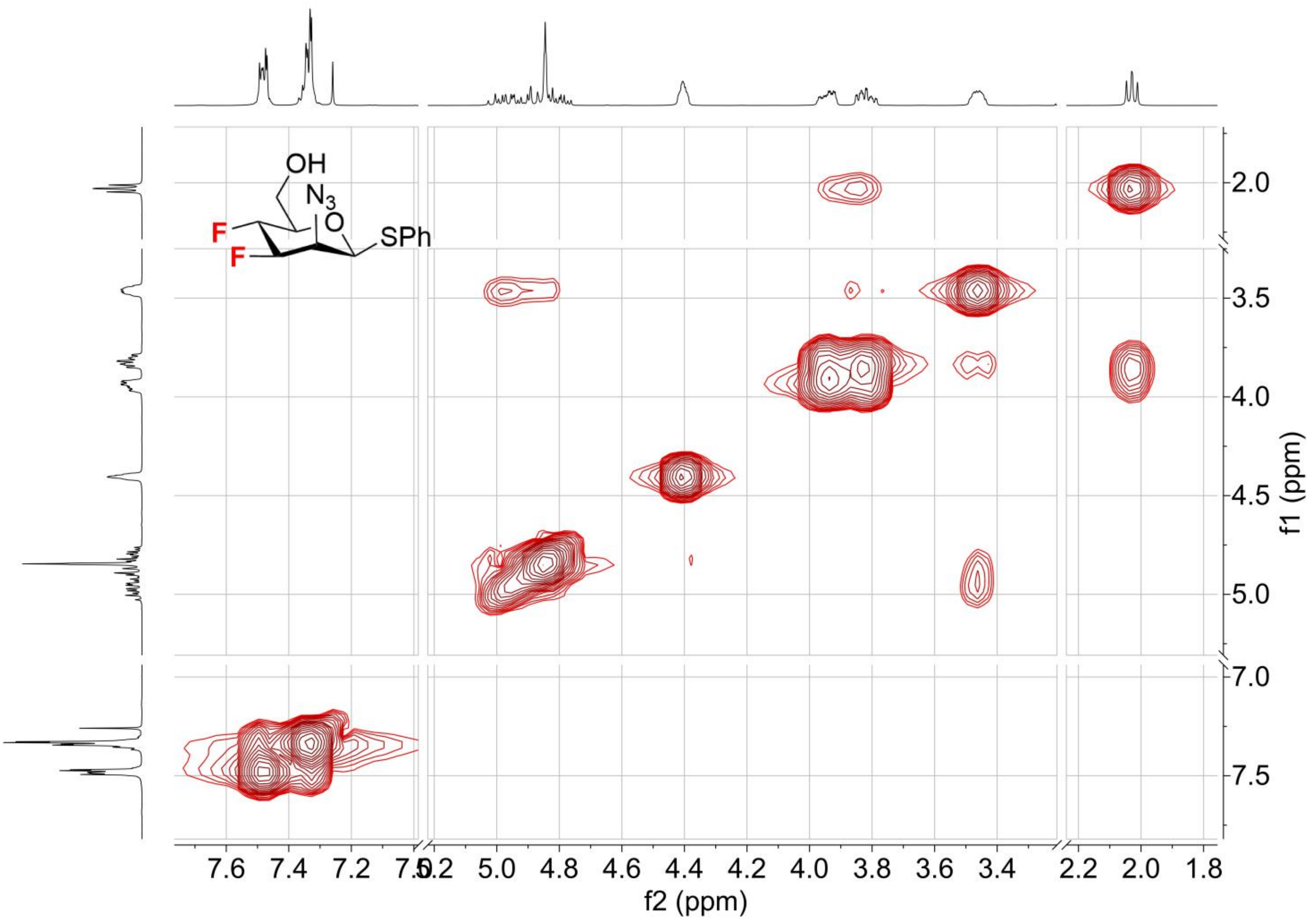

^{1}H-^{13}C HSQC (^{1}H/^{13}C 400/101 MHz, $CDCl_3$) β-57

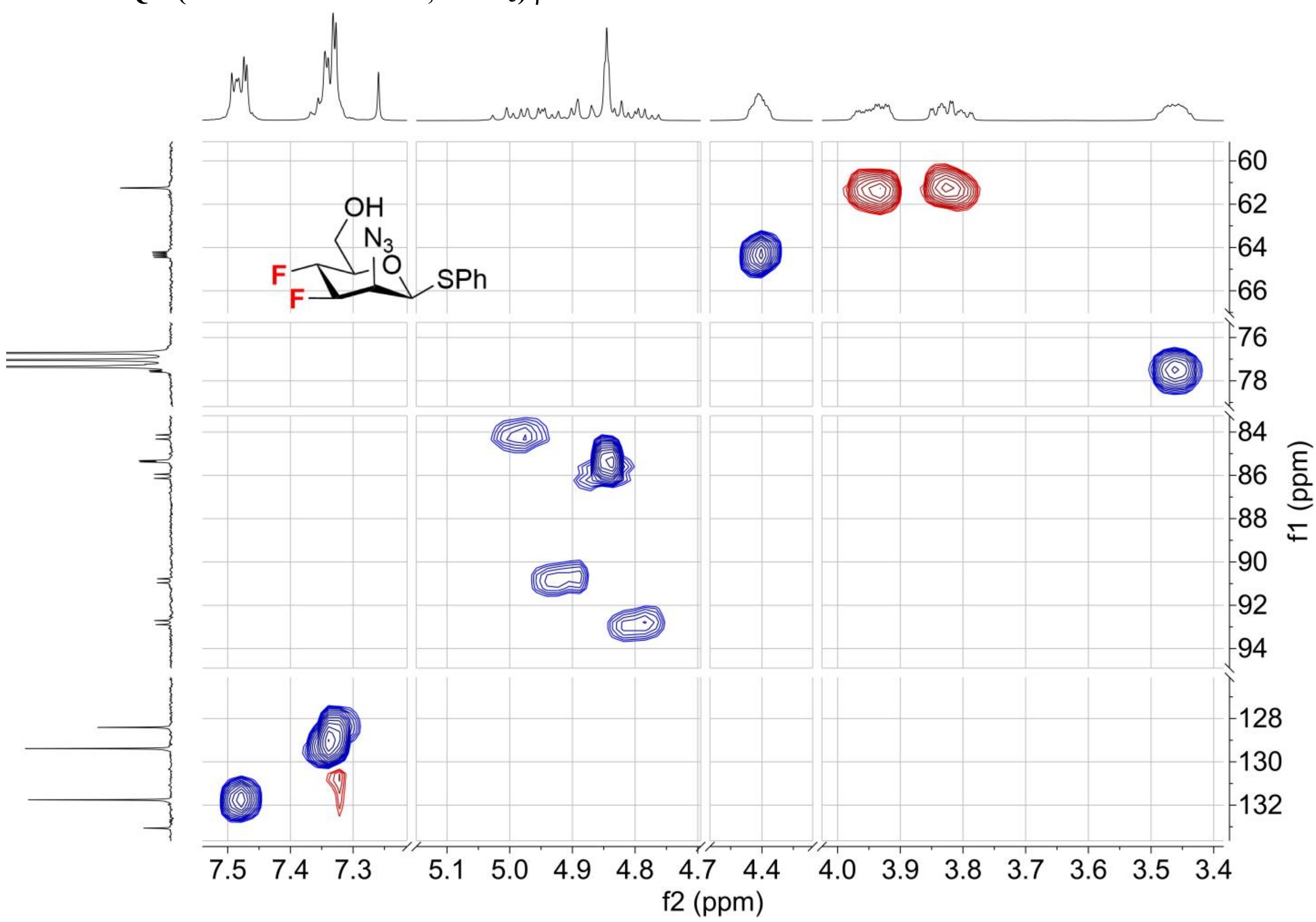

^{1}H-^{13}C HMBC (^{1}H/^{13}C 400/101 MHz, $CDCl_3$) β-57

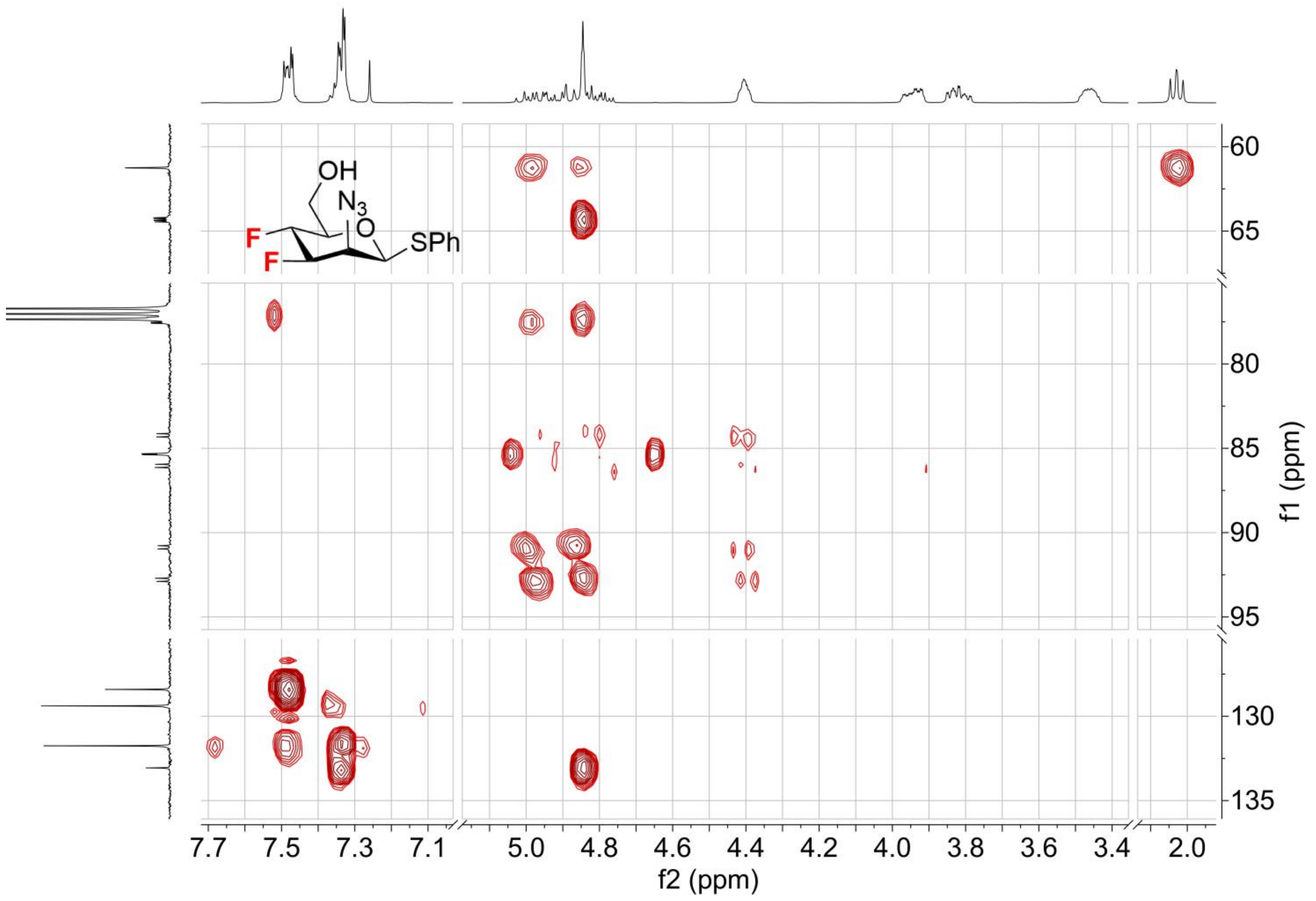

NMR COMPOUND 58

^{1}H NMR (400 MHz, $CDCl_3$) 58

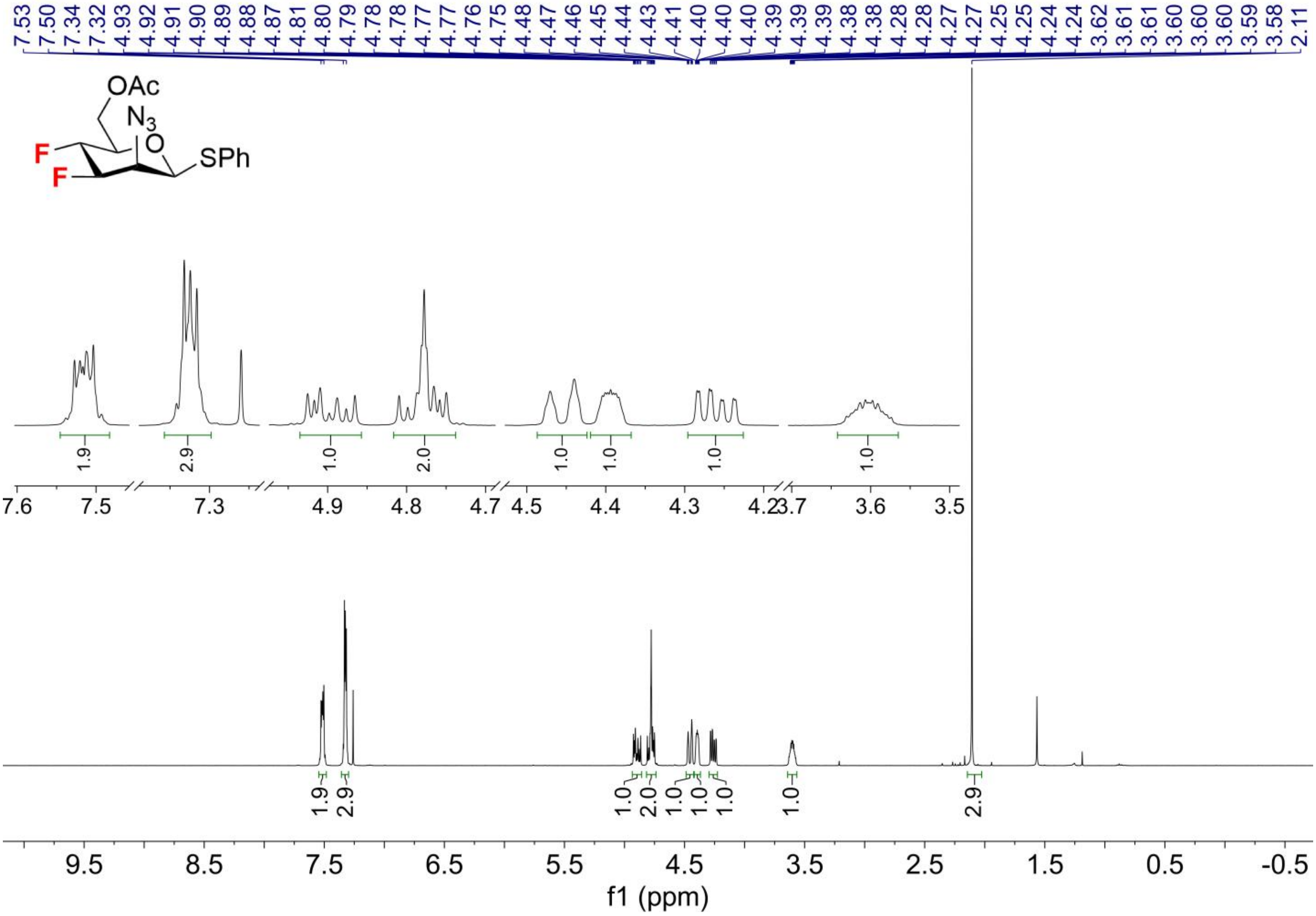

^{13}C{^{1}H} NMR (101 MHz, $CDCl_3$) 58

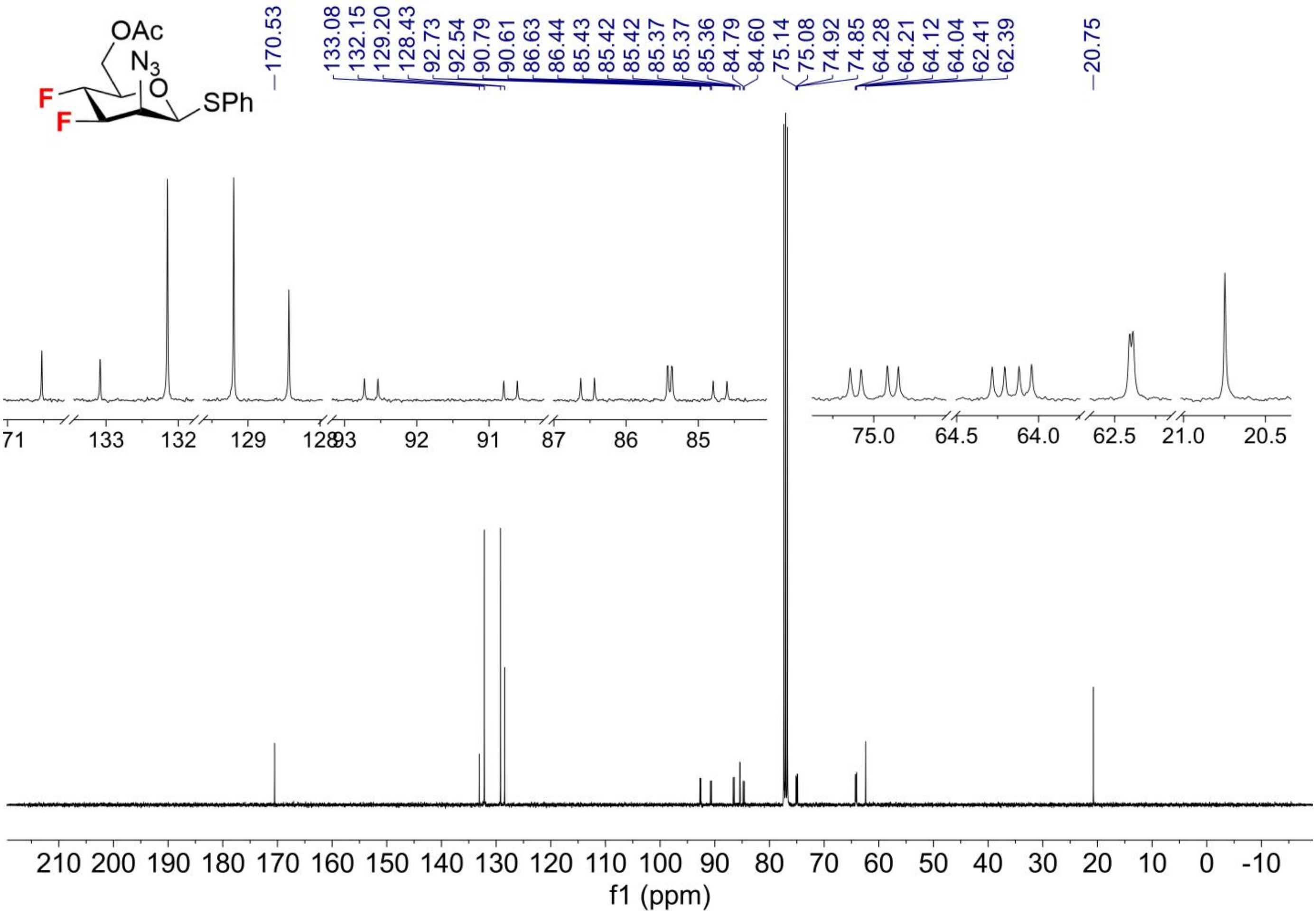

^{19}F NMR (376 MHz, CDCl$_3$) 58

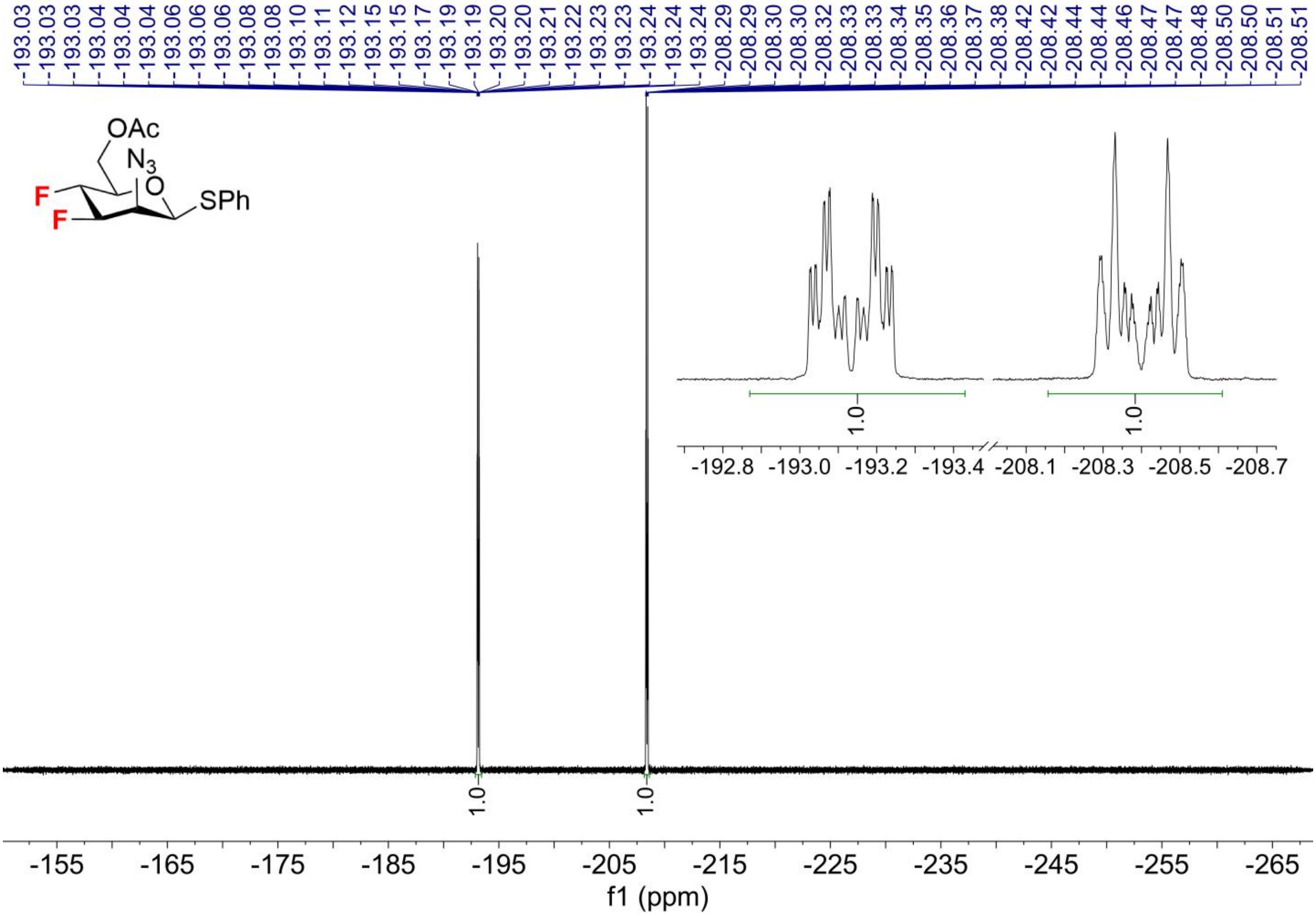

^{1}H-^{1}H COSY (400 MHz, CDCl$_3$) 58

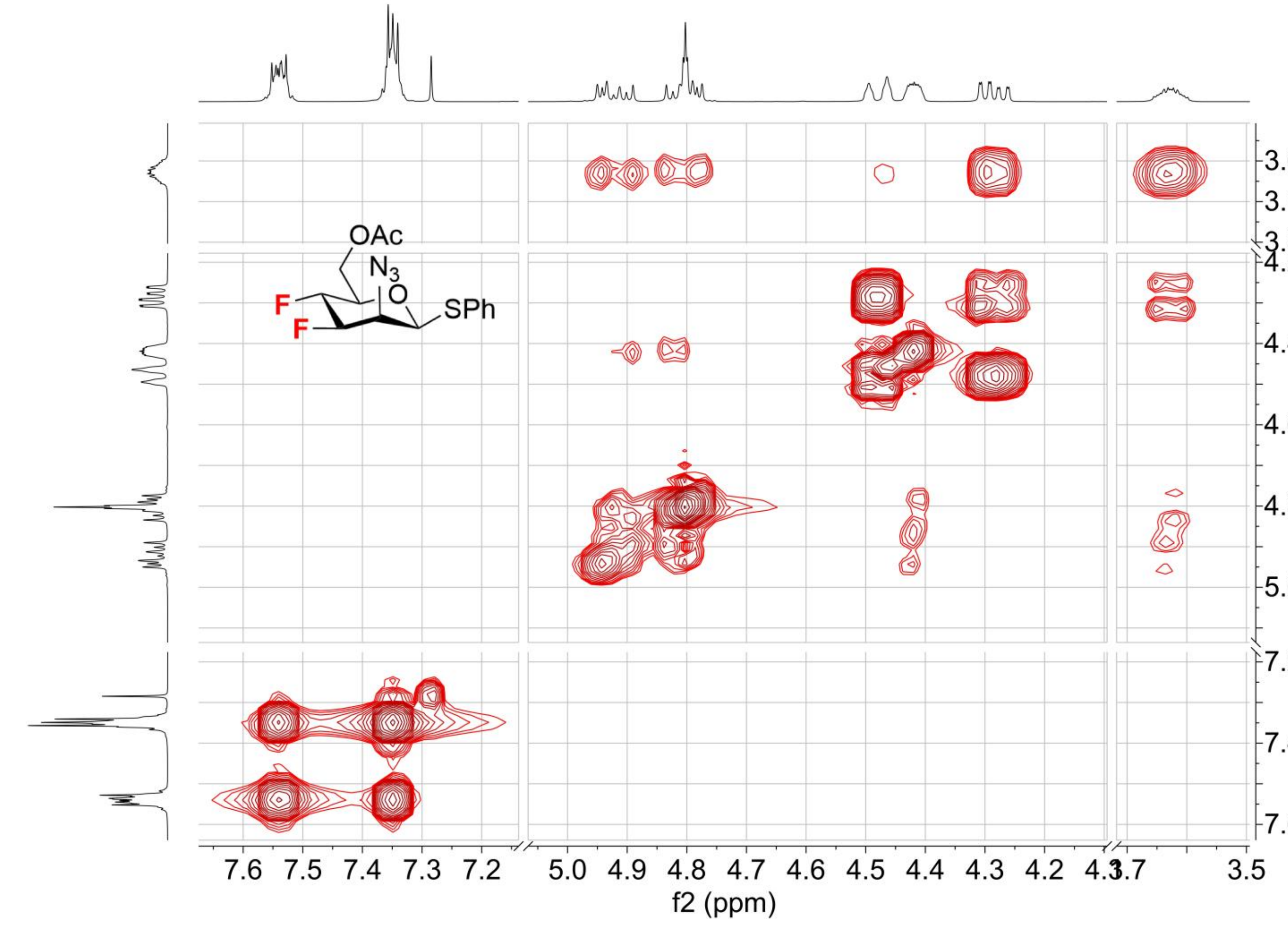

^{1}H-^{13}C HSQC (^{1}H/^{13}C 400/101 MHz, $CDCl_3$) 58

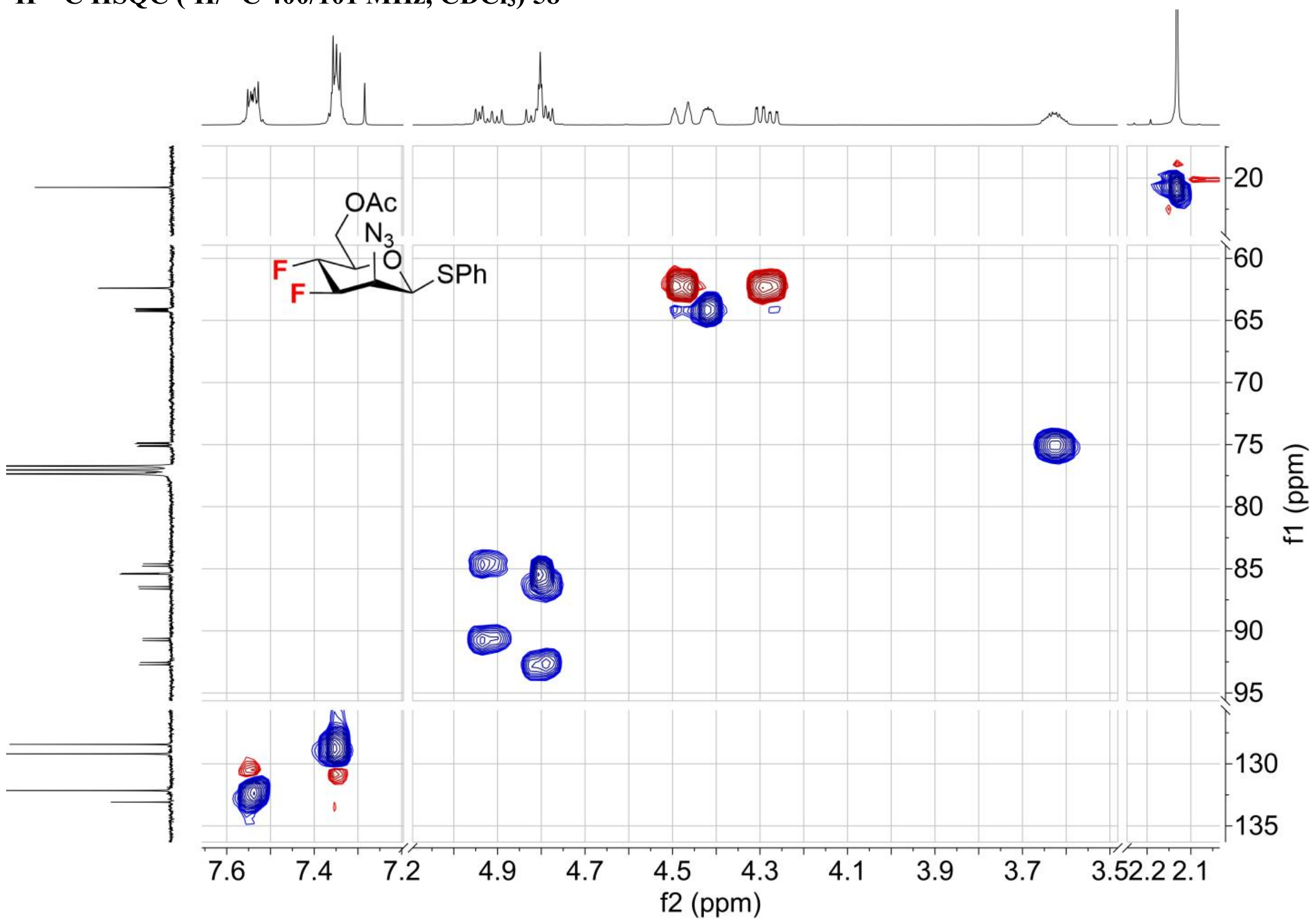

^{1}H-^{13}C HMBC (^{1}H/^{13}C 400/101 MHz, $CDCl_3$) 58

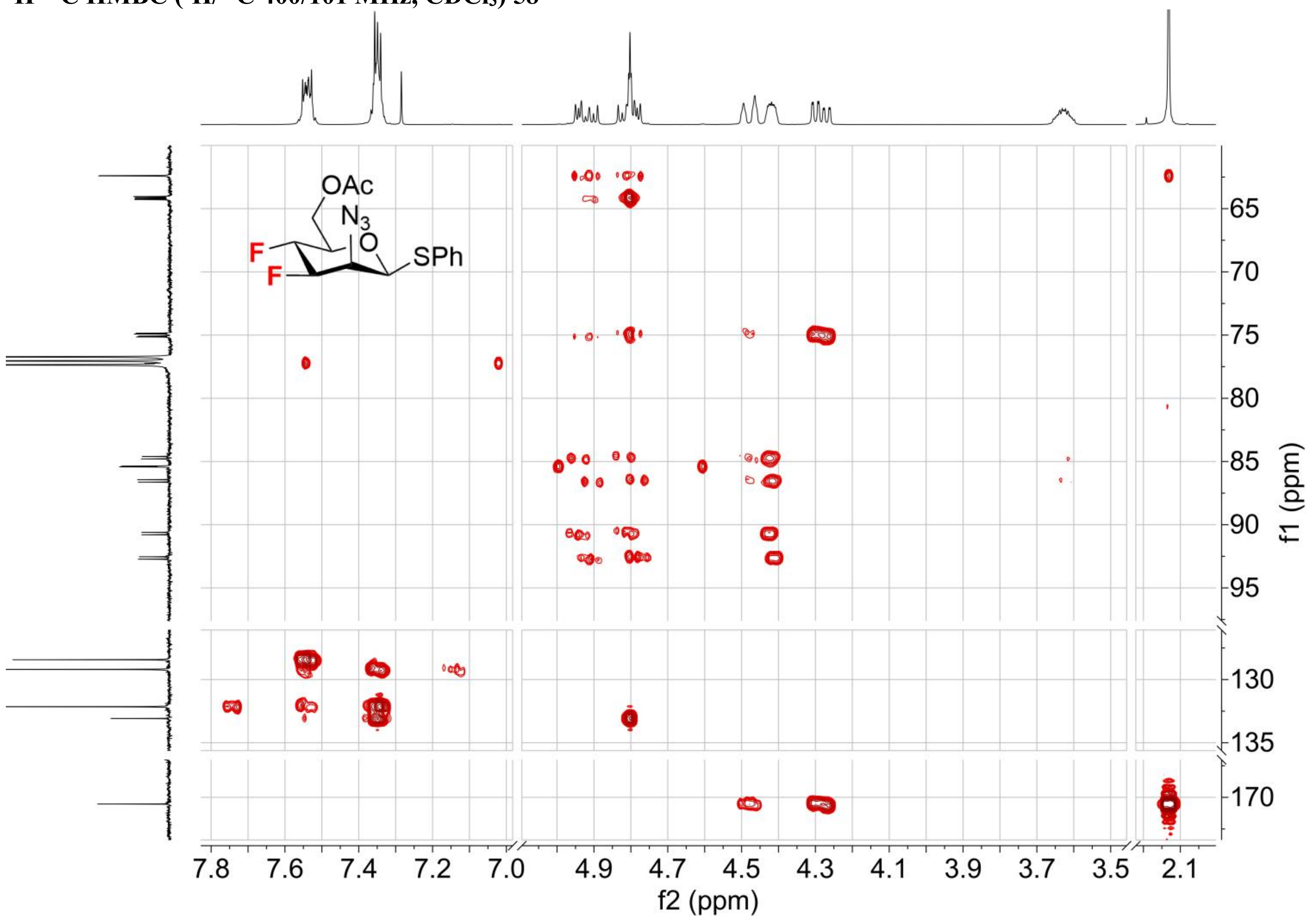

NMR COMPOUND 59

^{1}H NMR (400 MHz, $CDCl_3$) 59

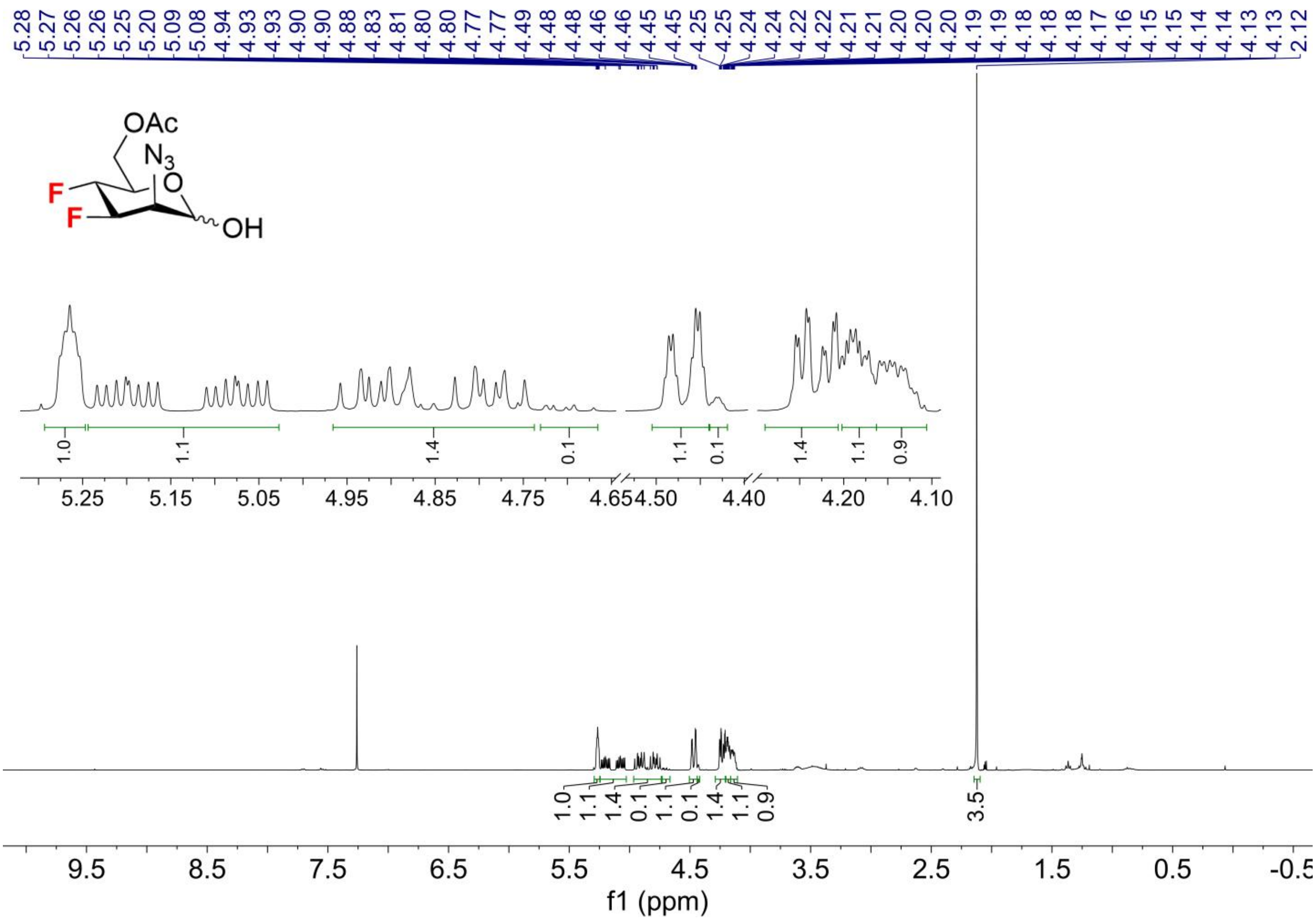

^{13}C{^{1}H} NMR (101 MHz, $CDCl_3$) 59

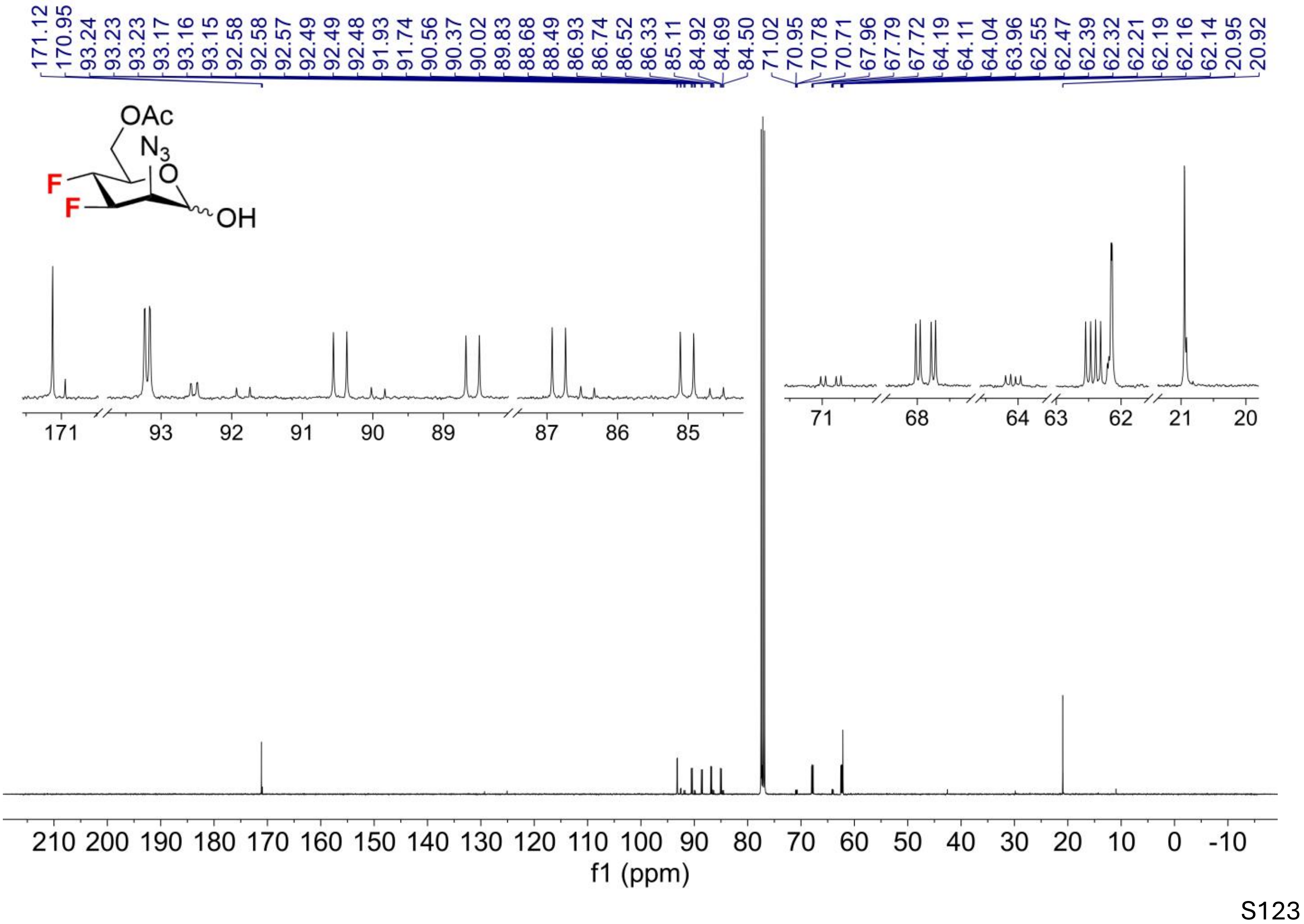

^{19}F NMR (376 MHz, CDCl₃) 59

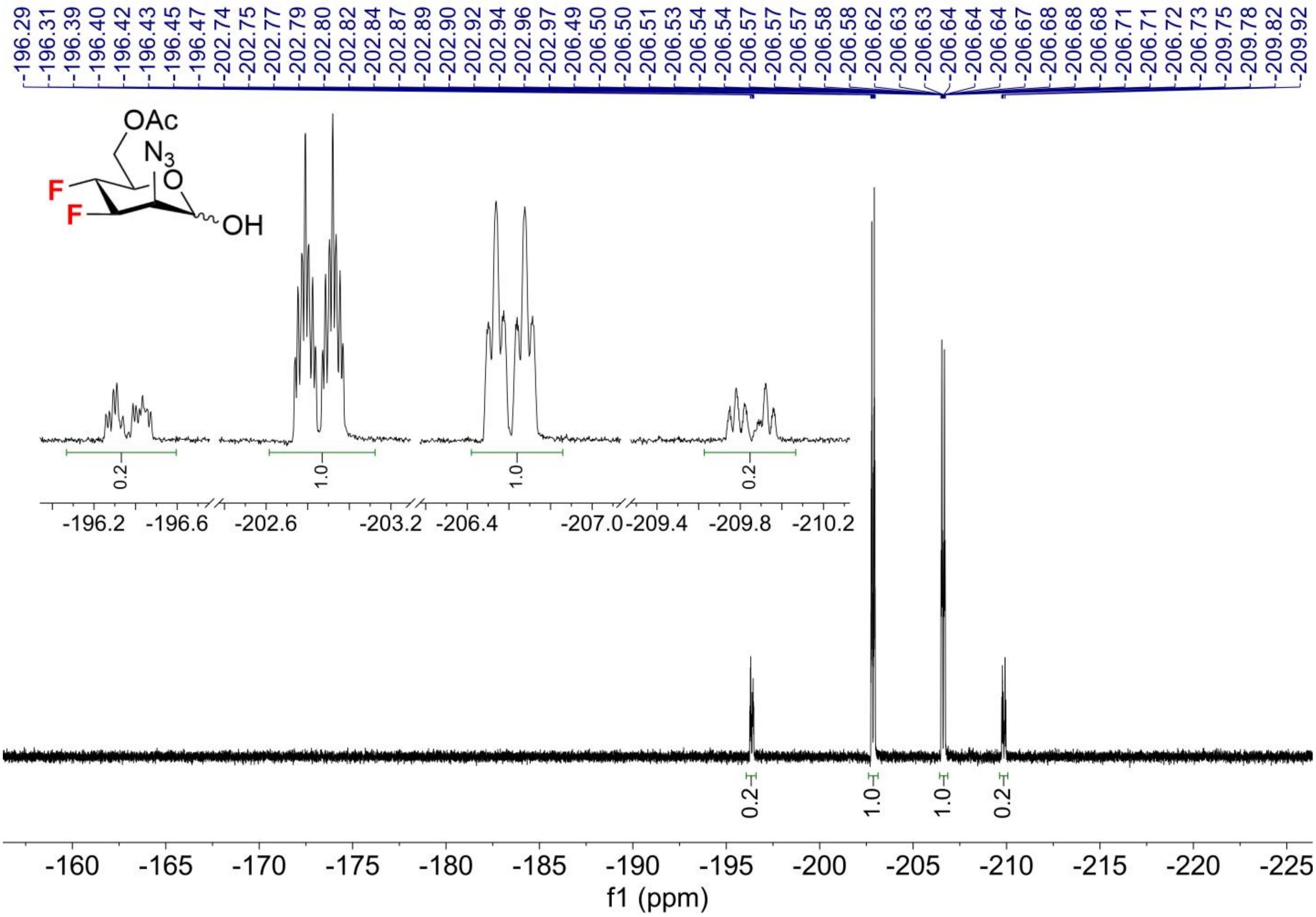

^{1}H-^{1}H COSY (400 MHz, CDCl₃) 59

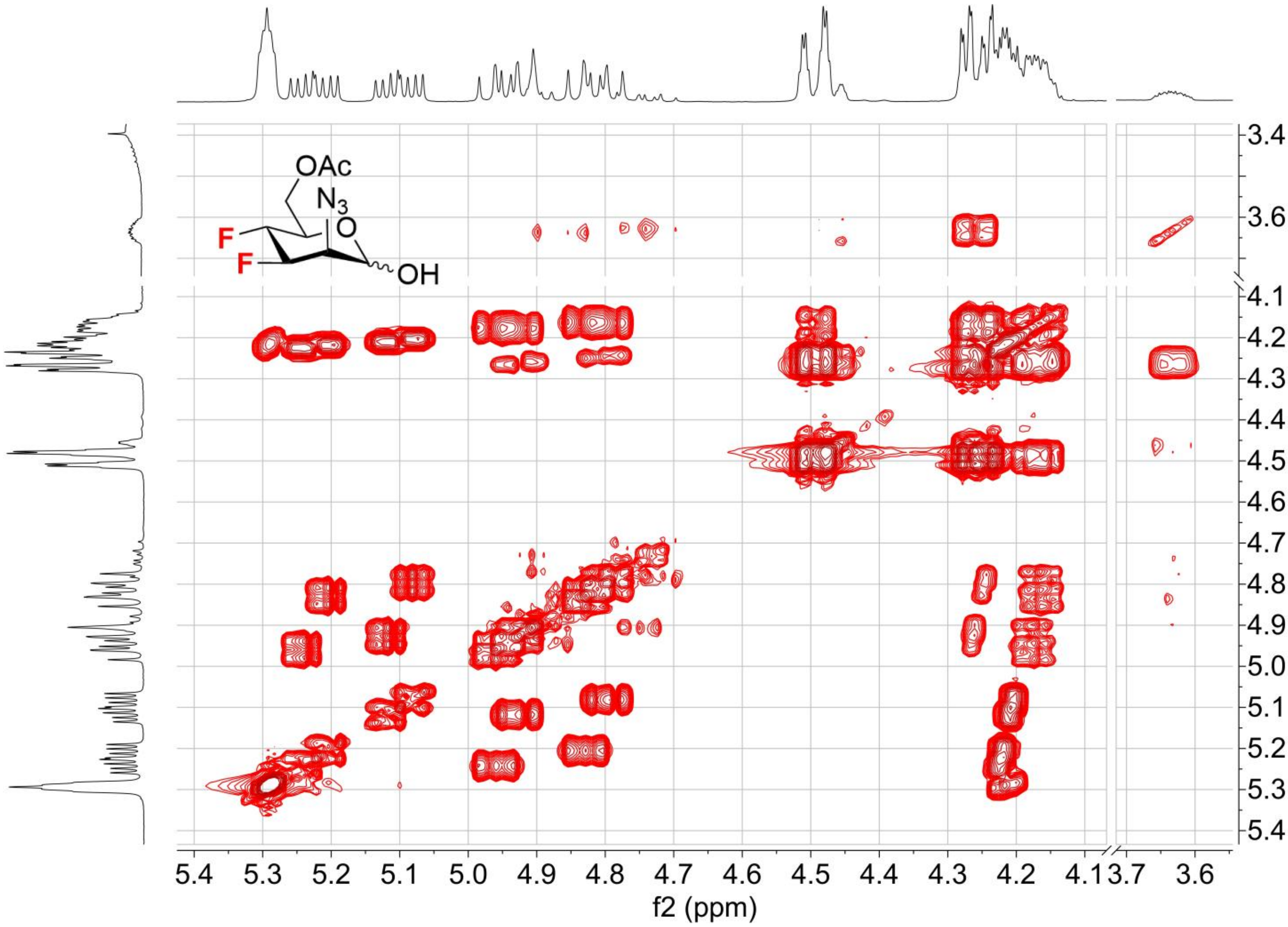

^{1}H-^{13}C HSQC (^{1}H/^{13}C 400/101 MHz, $CDCl_3$) 59

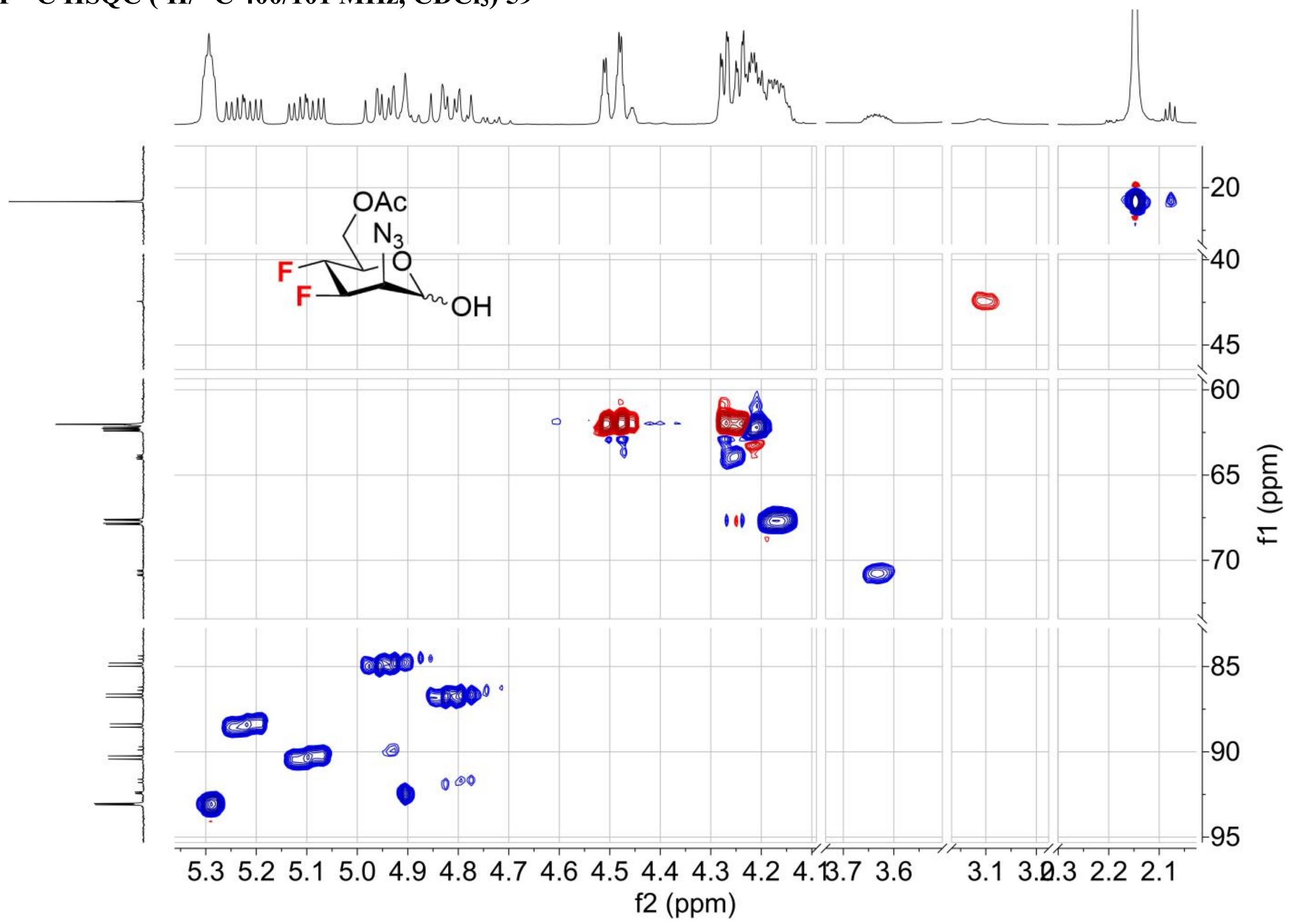

^{1}H-^{13}C HMBC (^{1}H/^{13}C 400/101 MHz, $CDCl_3$) 59

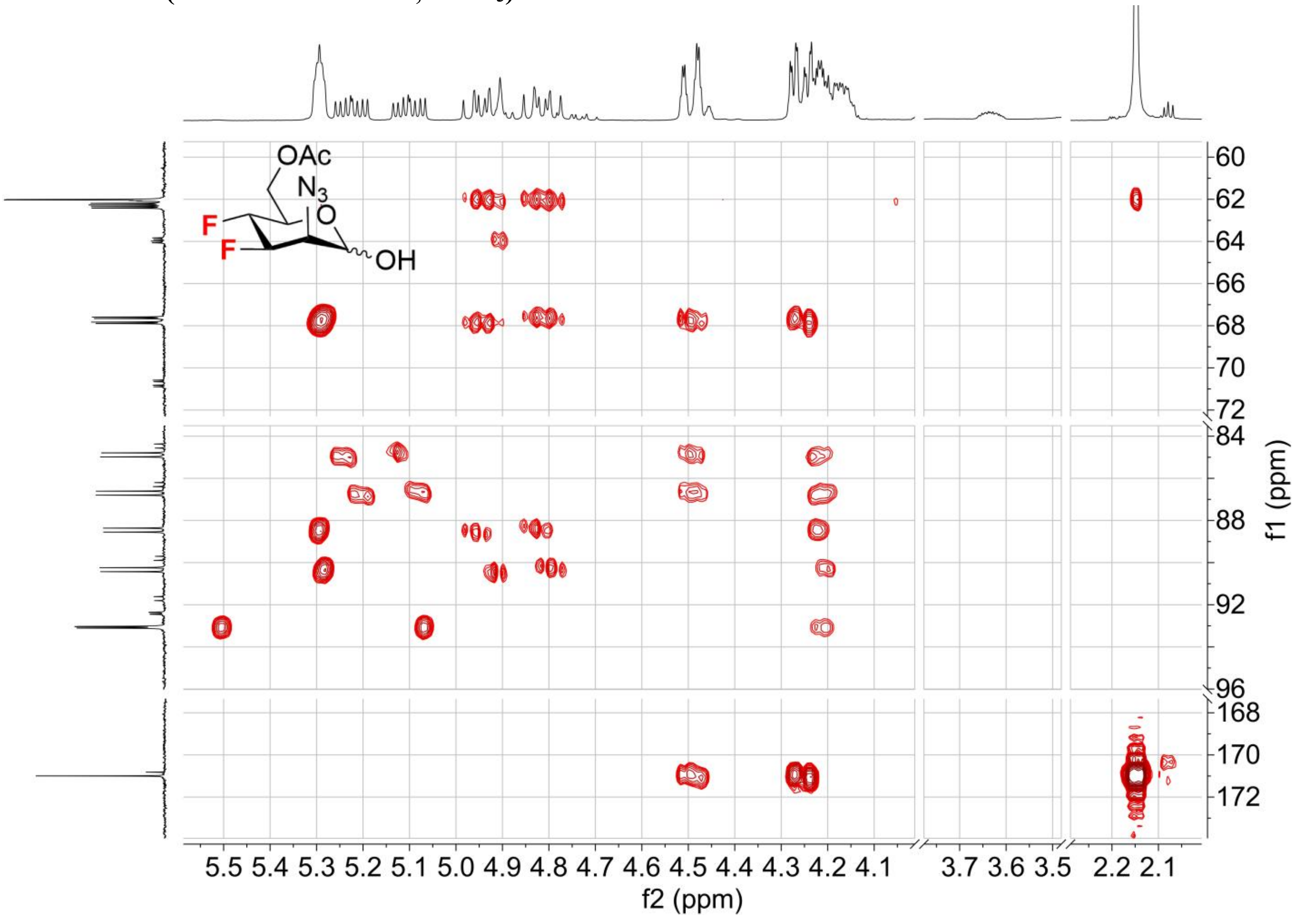

NMR COMPOUND 60

^{1}H NMR (400 MHz, CDCl$_3$) 60

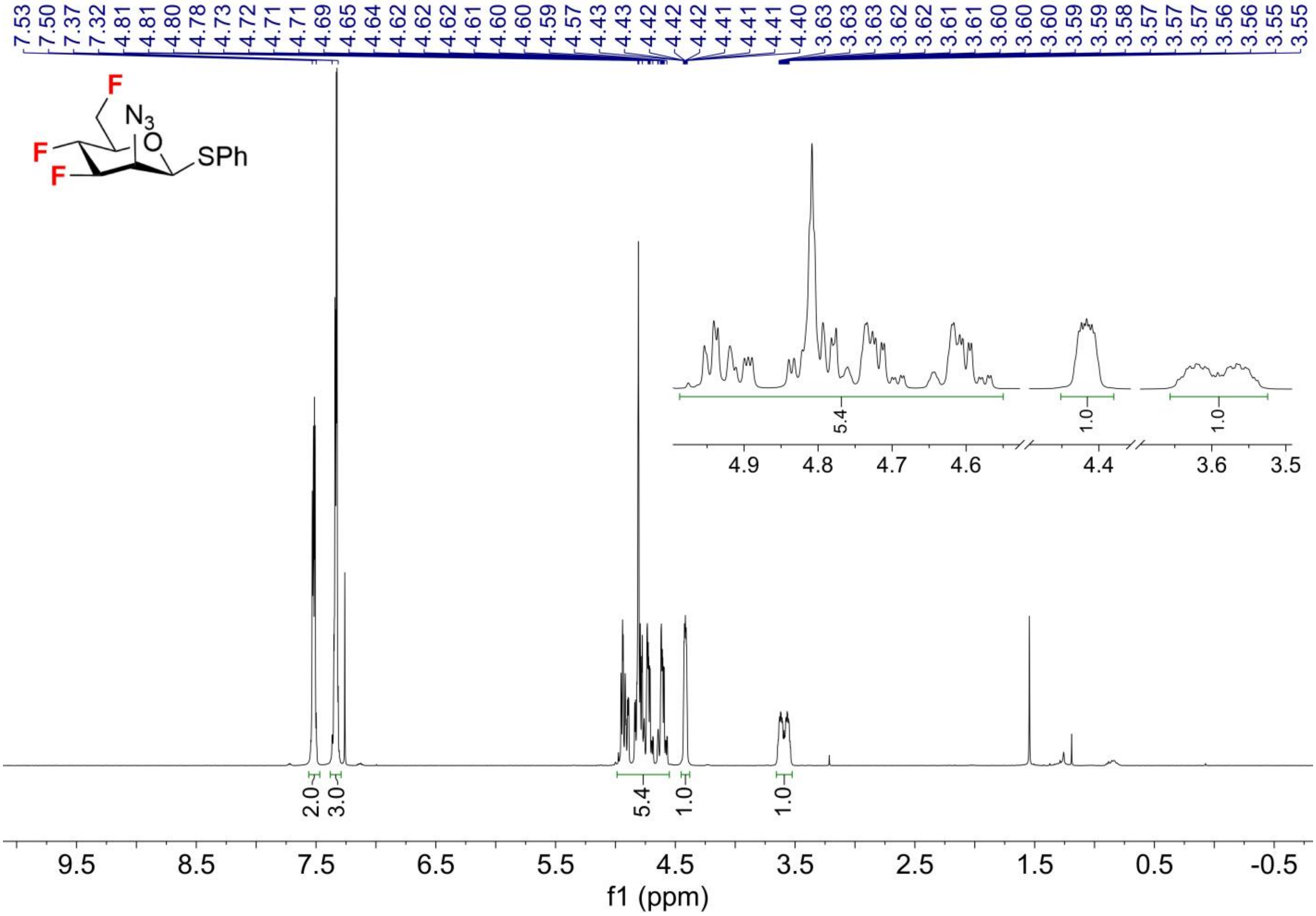

^{13}C{^{1}H} NMR (101 MHz, CDCl$_3$) 60

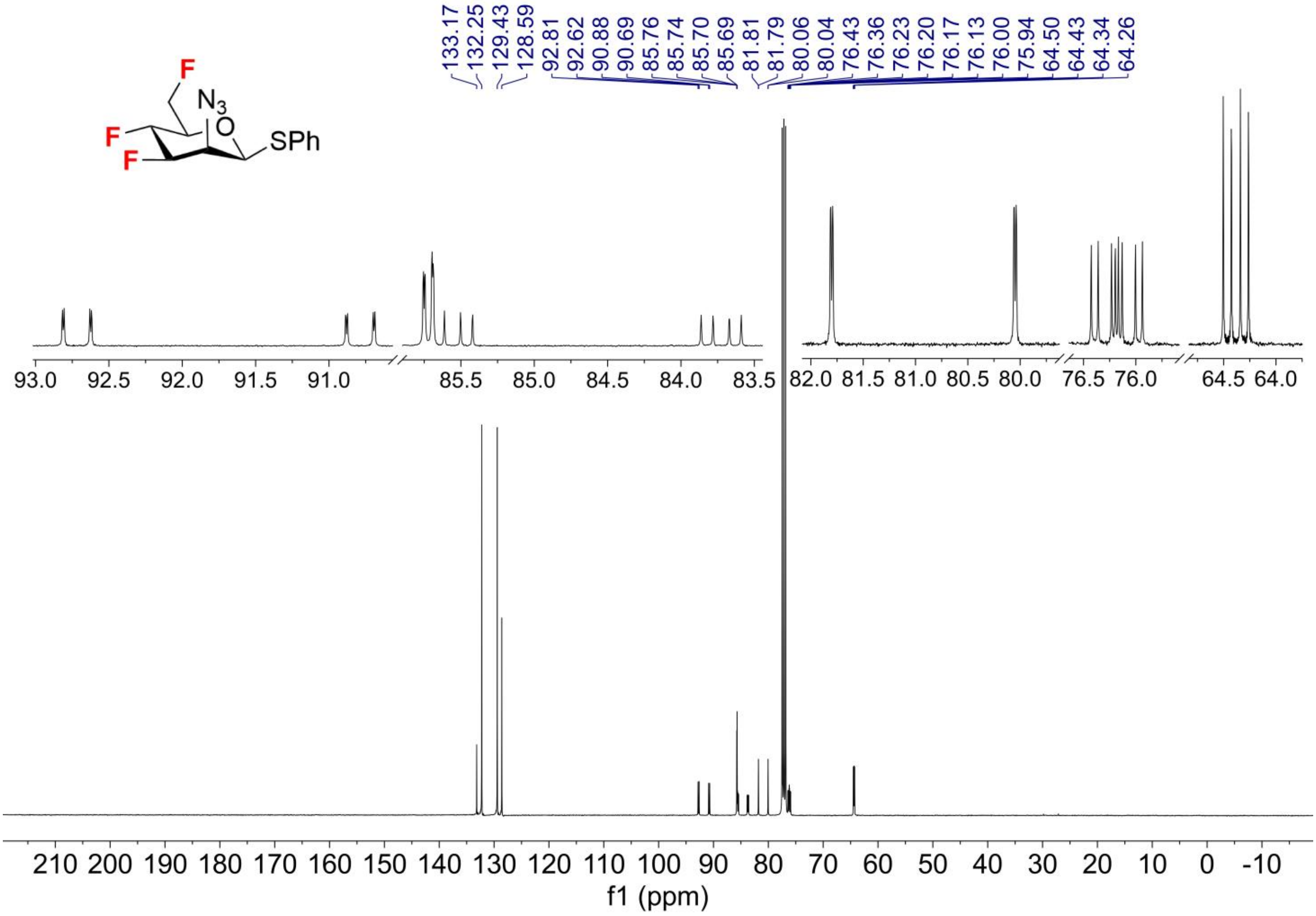

^{19}F NMR (376 MHz, CDCl$_3$) 60

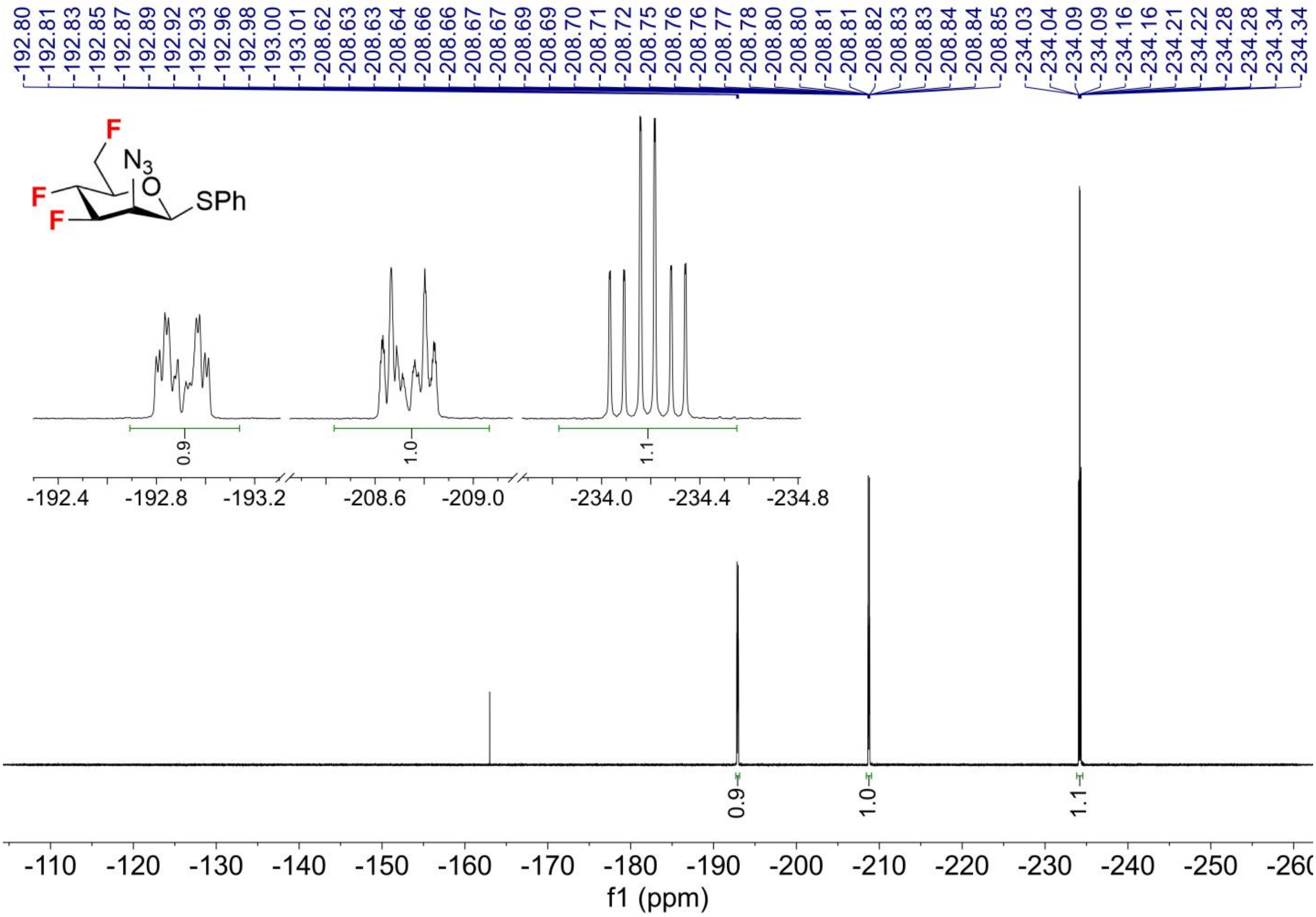

^{1}H-^{1}H COSY (400 MHz, CDCl$_3$) 60

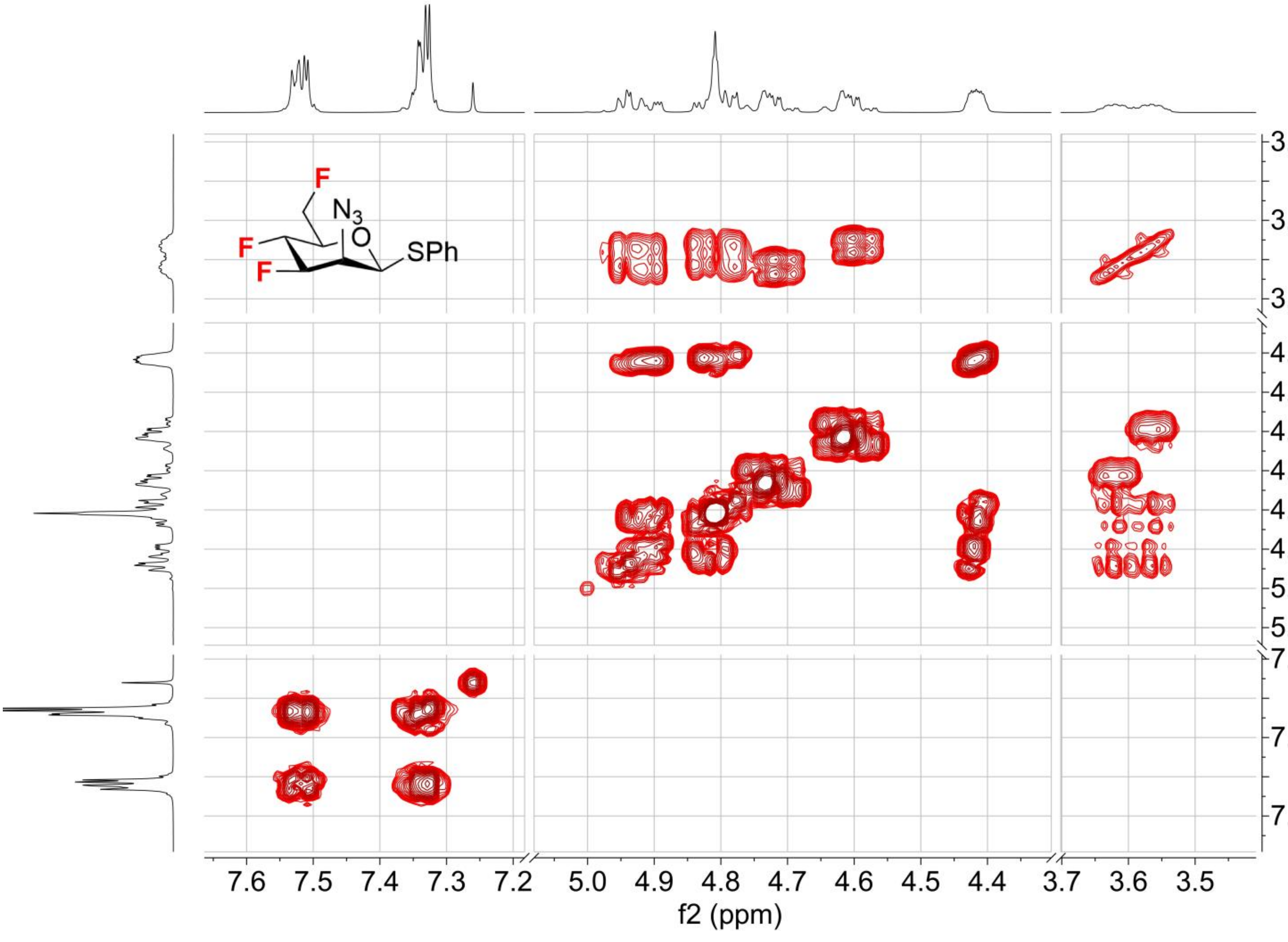

^{1}H-^{13}C HSQC (^{1}H/^{13}C 400/101 MHz, $CDCl_3$) 60

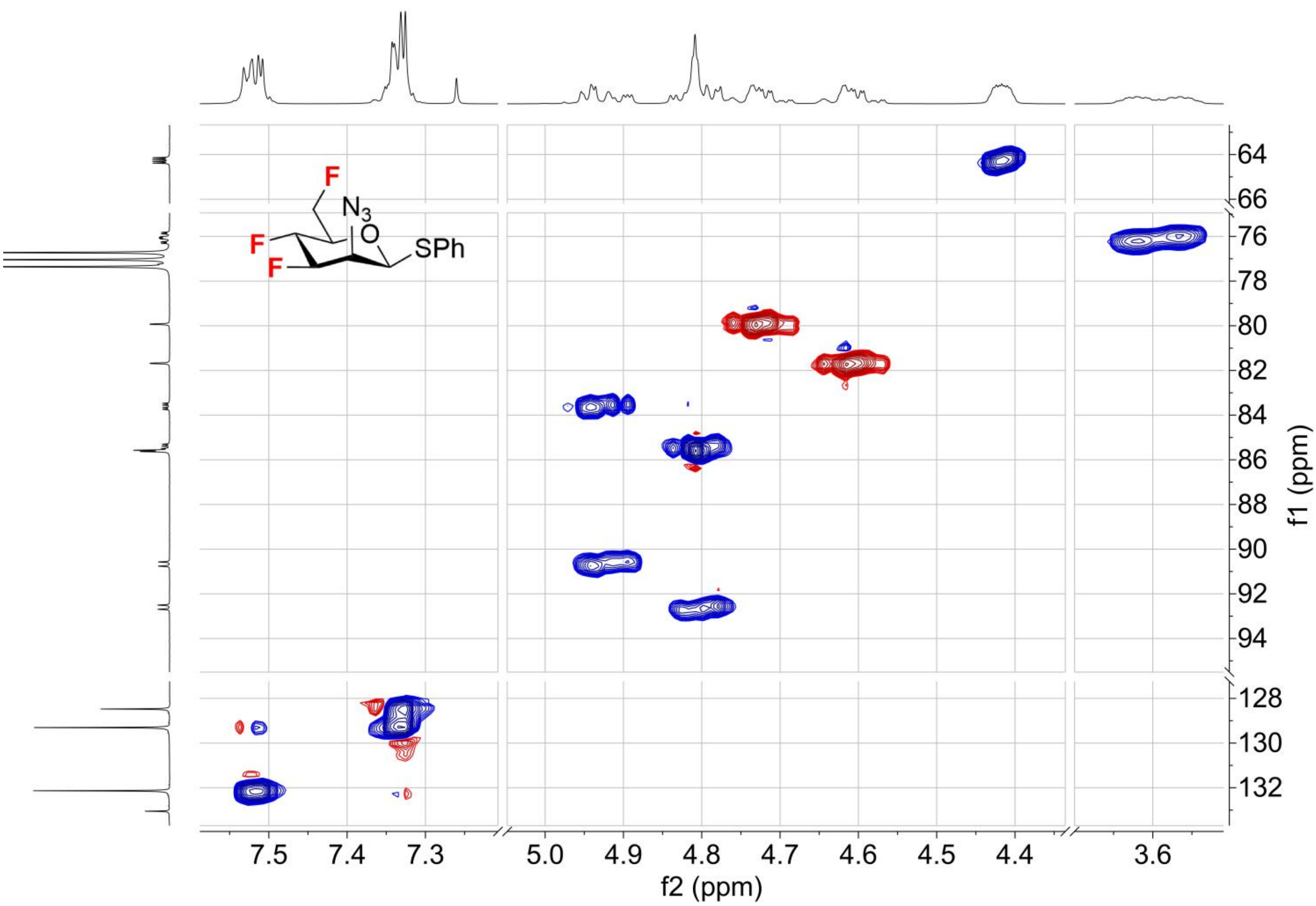

^{1}H-^{13}C HMBC (^{1}H/^{13}C 400/101 MHz, $CDCl_3$) 60

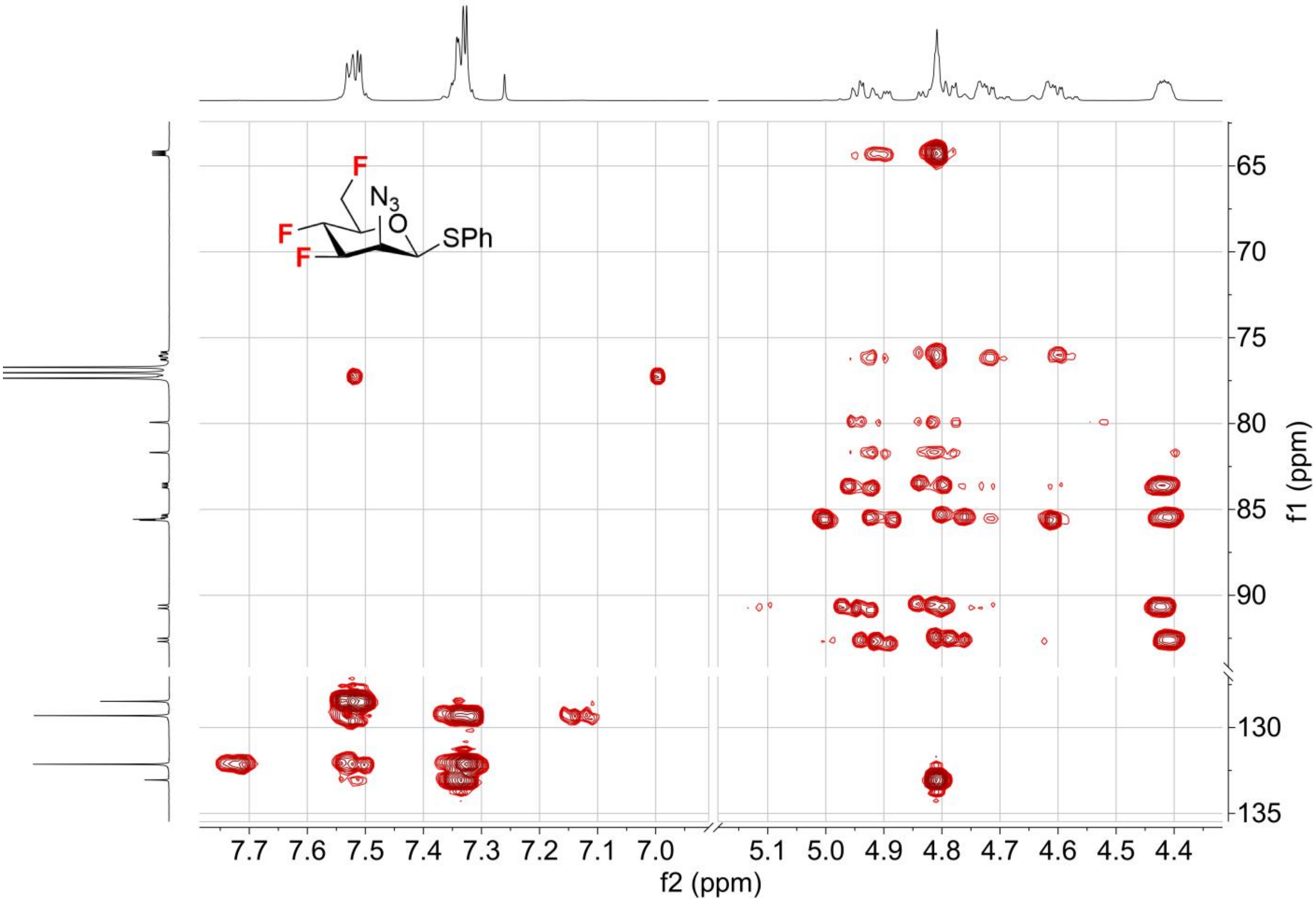

NMR COMPOUND 61

^{1}H NMR (400 MHz, $CDCl_3$) 61

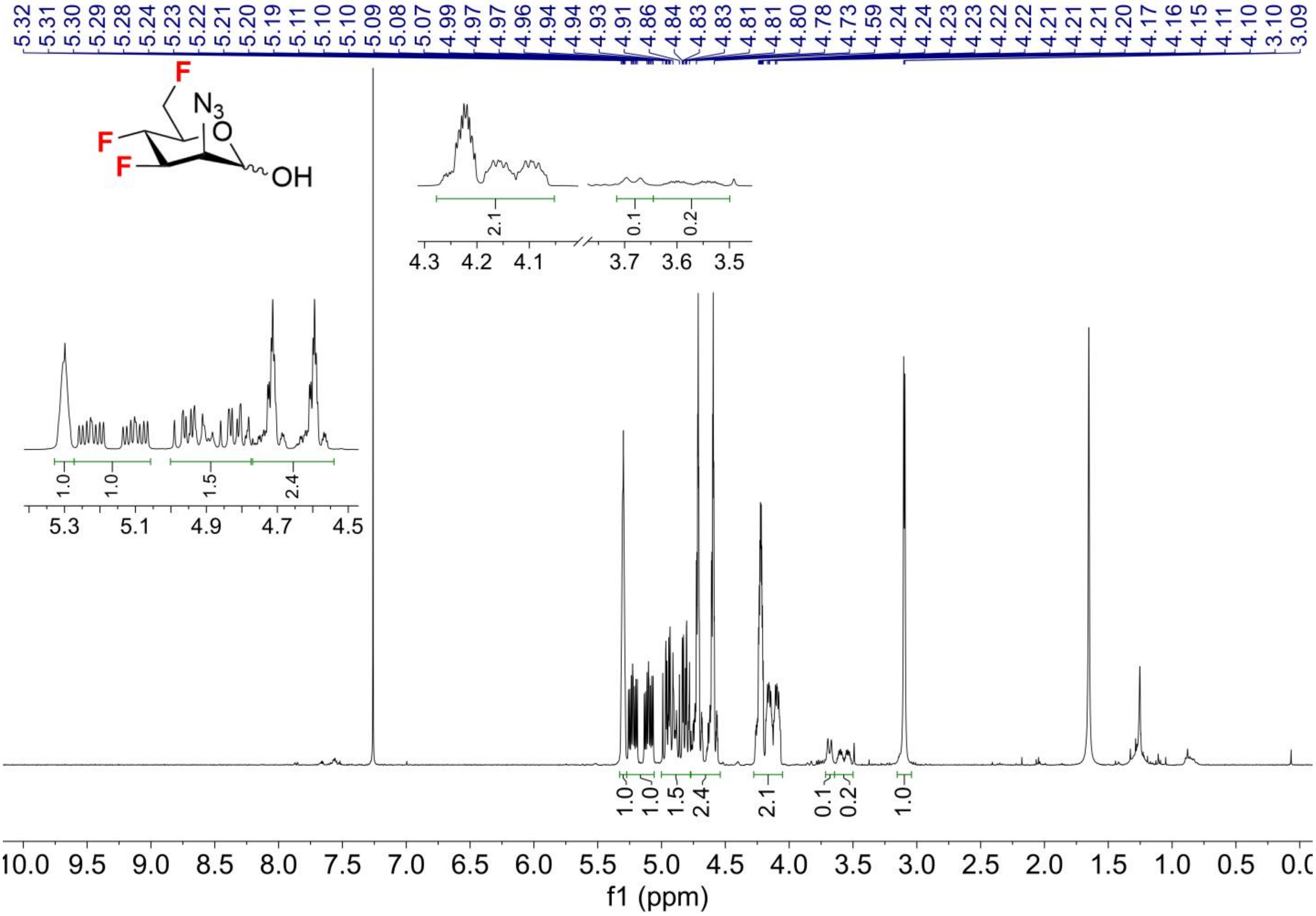

^{13}C{^{1}H} NMR (101 MHz, $CDCl_3$) 61

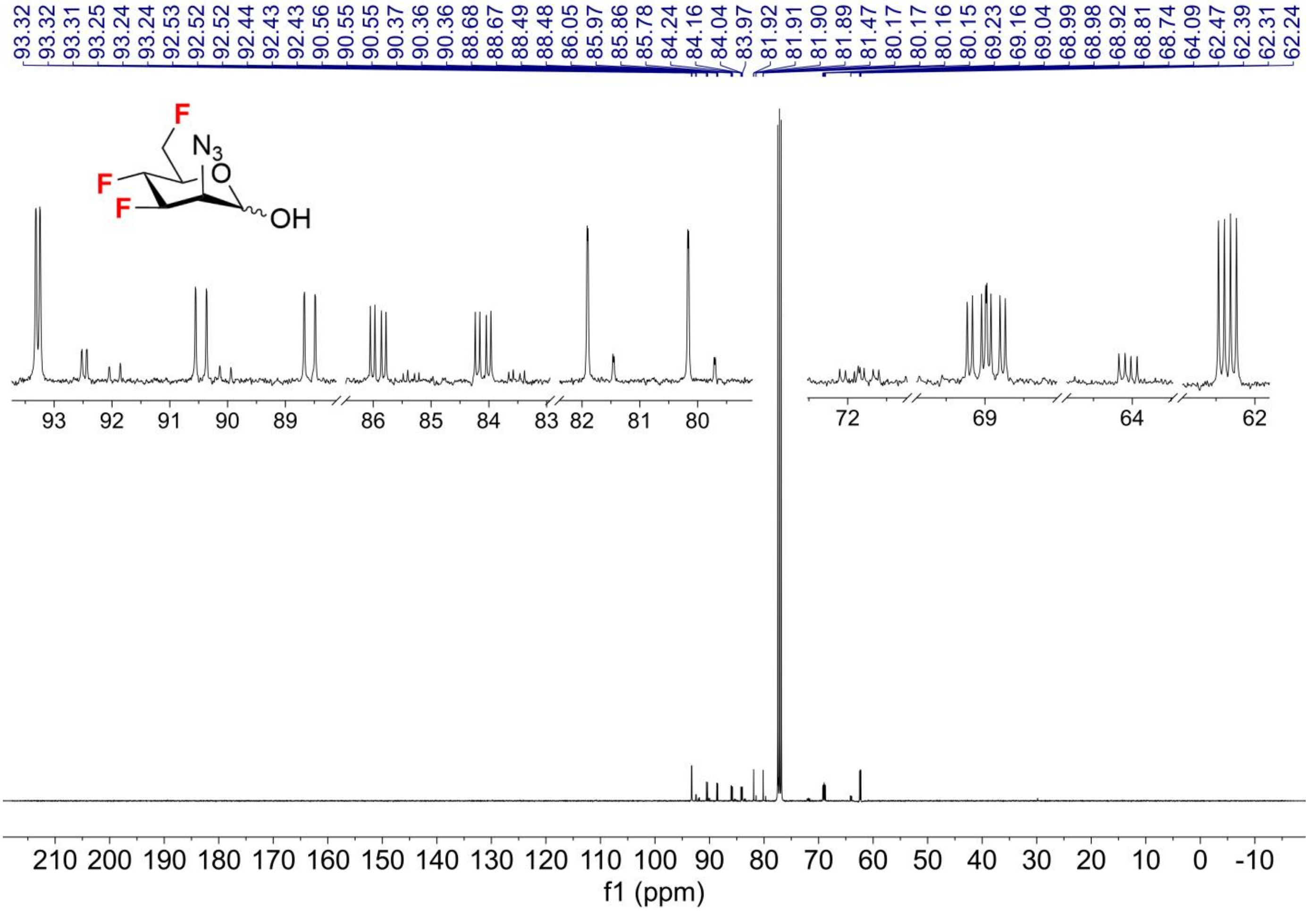

^{19}F NMR (376 MHz, CDCl$_3$) 61

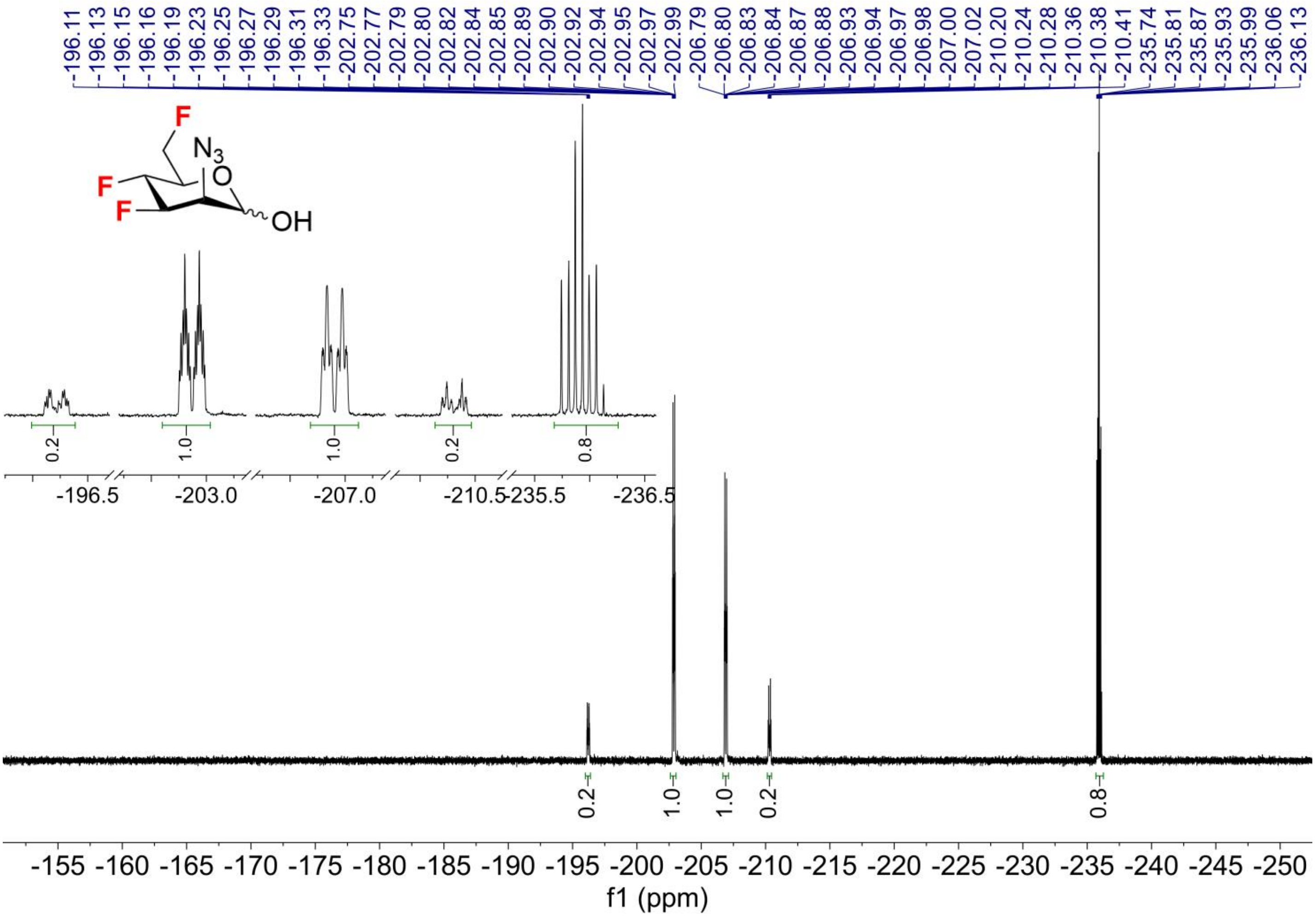

^{1}H-^{1}H COSY (400 MHz, CDCl$_3$) 61

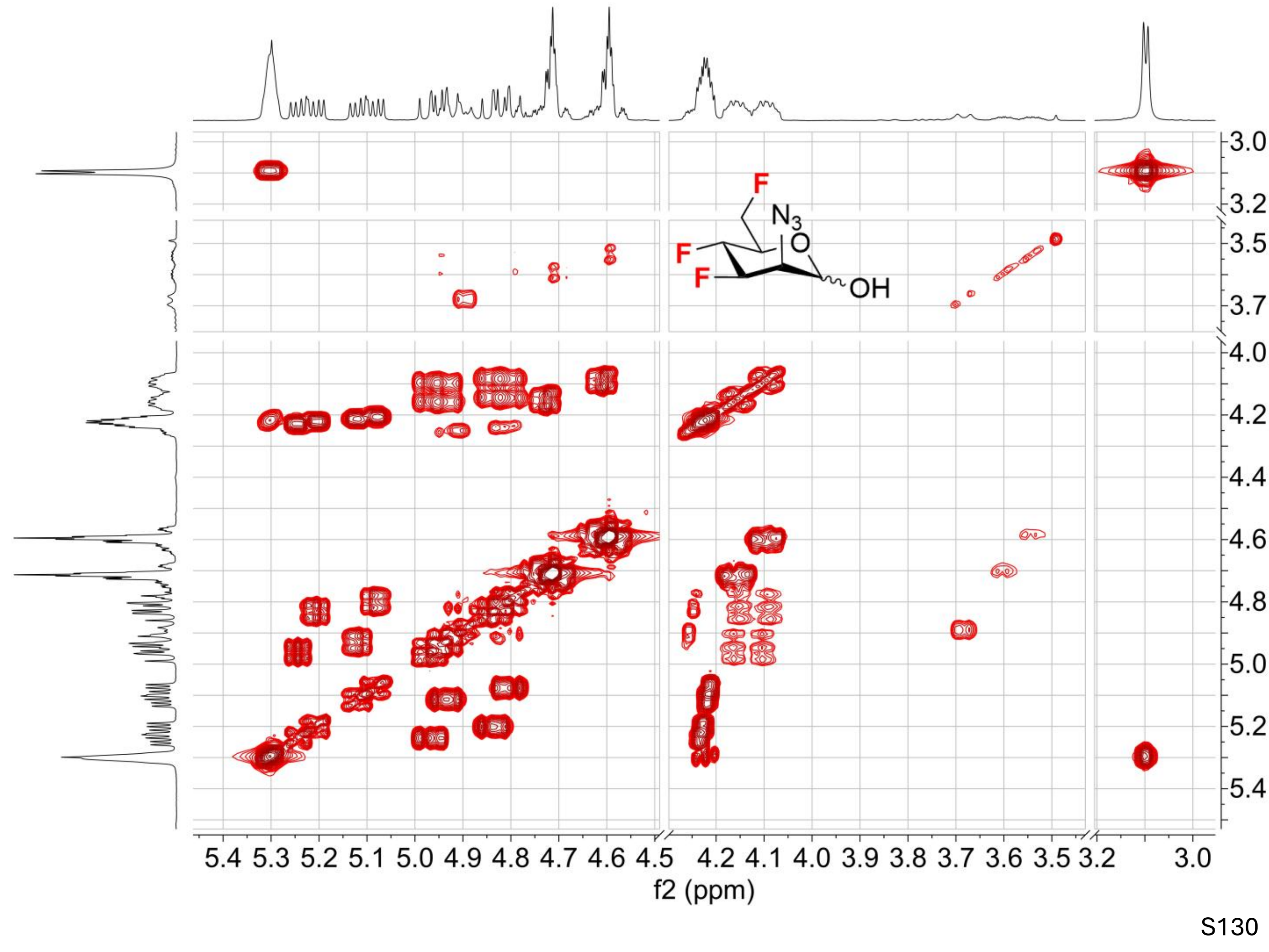

^{1}H-^{13}C HSQC (^{1}H/^{13}C 400/101 MHz, $CDCl_3$) 61

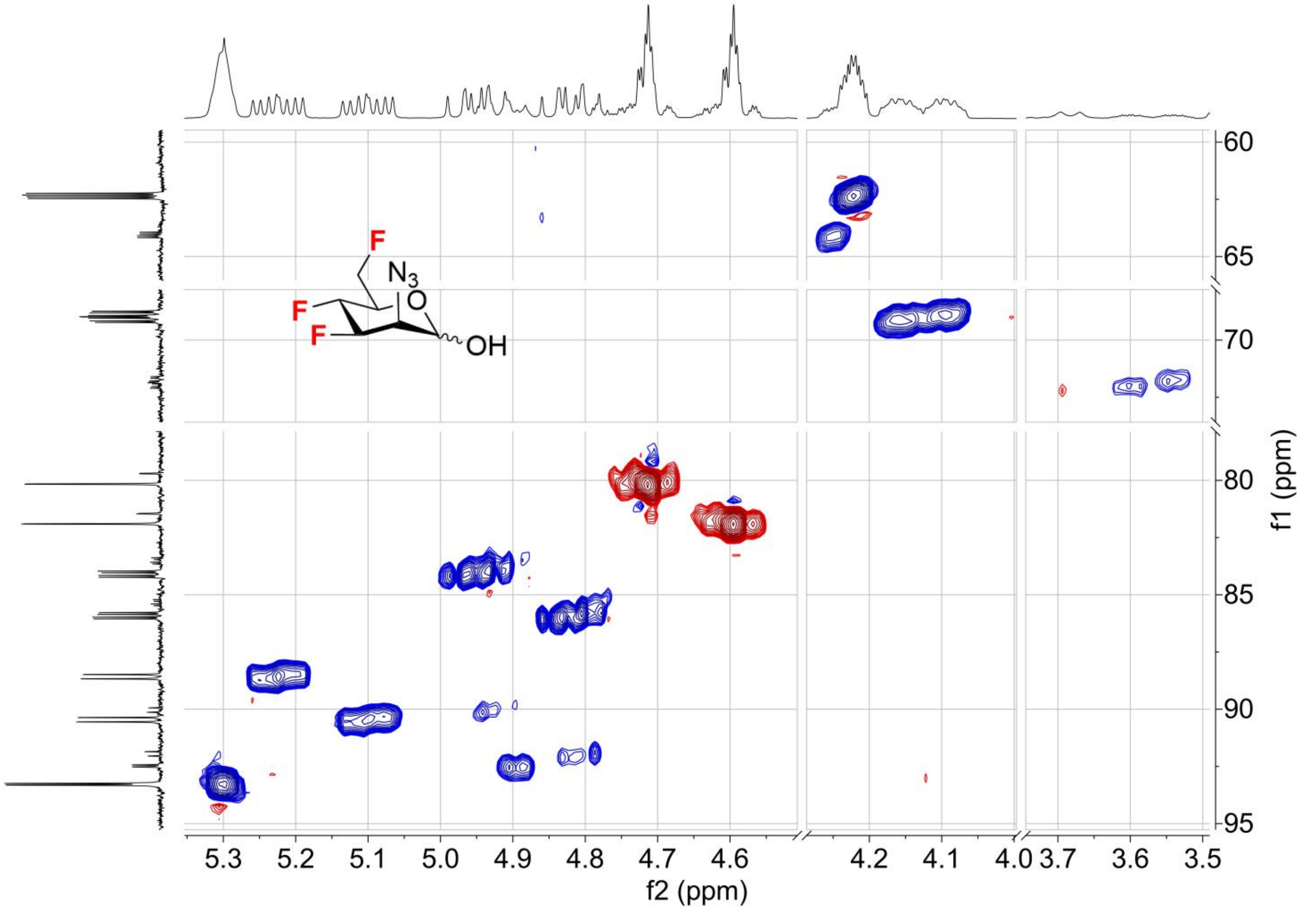

^{1}H-^{13}C HMBC (^{1}H/^{13}C 400/101 MHz, $CDCl_3$) 61

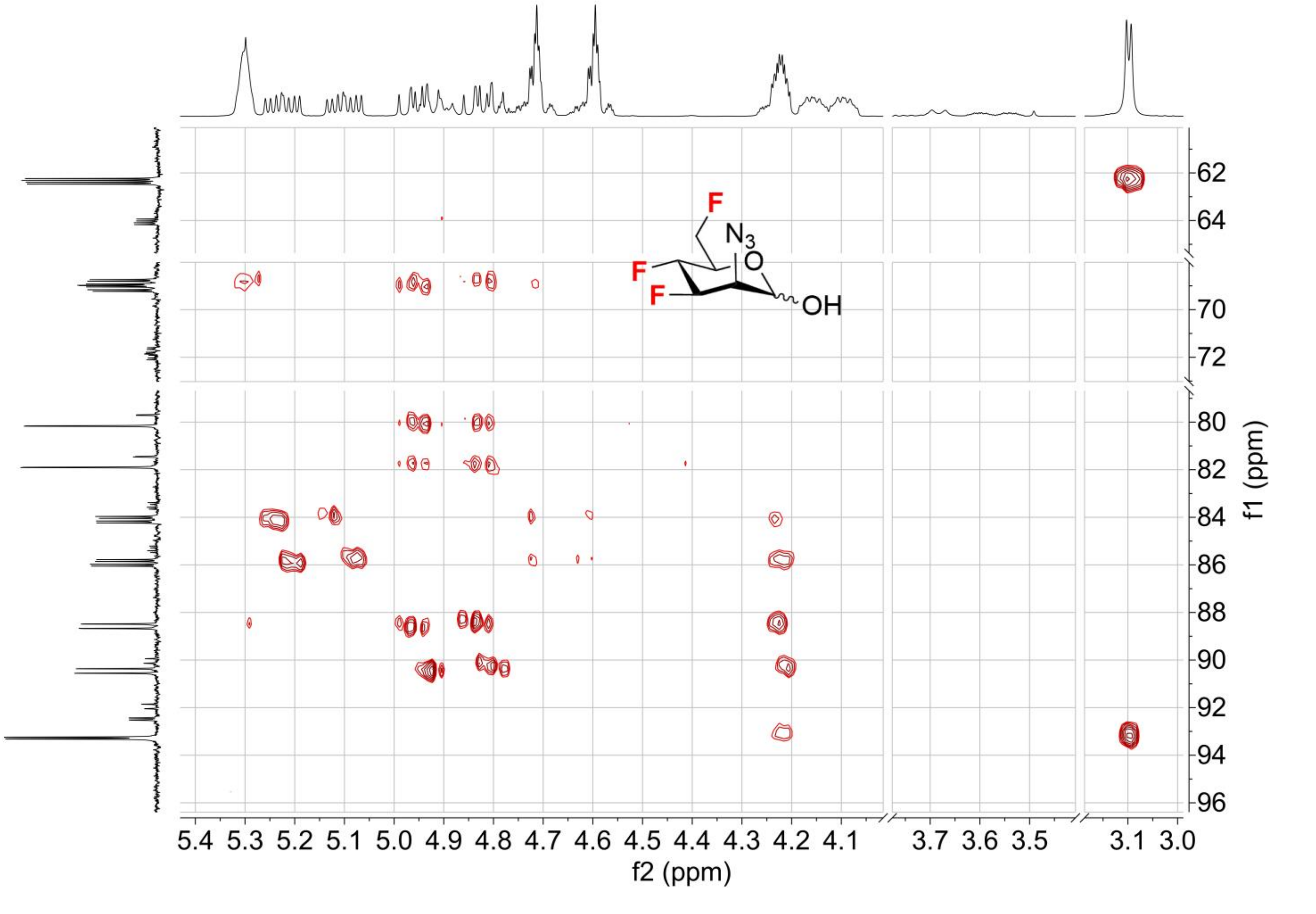

Supplement: Supplementary file 2 [file jo5c03084_si_002.pdf]
